# Rapid Asymmetric Synthesis of Disubstituted Allenes by Coupling of Flow‐Generated Diazo Compounds and Propargylated Amines

**DOI:** 10.1002/anie.201611067

**Published:** 2017-01-11

**Authors:** Jian‐Siang Poh, Szabolcs Makai, Timo von Keutz, Duc N. Tran, Claudio Battilocchio, Patrick Pasau, Steven V. Ley

**Affiliations:** ^1^ Department of ChemistryUniversity of CambridgeLensfield RoadCambridgeCB2 1EWUK; ^2^ UCB Biopharma SPRLChemical Research R5Chemin du Foriest1420Braine-L'AlleudBelgium

**Keywords:** allenes, asymmetric catalysis, copper, diazo compounds, flow chemistry

## Abstract

We report herein the asymmetric coupling of flow‐generated unstabilized diazo compounds and propargylated amine derivatives, using a new pyridinebis(imidazoline) ligand, a copper catalyst and base. The reaction proceeds rapidly, generating chiral allenes in 10–20 minutes with high enantioselectivity (89–98 % de/ee), moderate yields and a wide functional group tolerance.

Allenes continue to hold a general interest due to their cumulated, orthogonal π bonds. Since their initial discovery, they have been found in natural products and incorporated into various functional materials.^1^, ^2^ Perhaps most importantly, however, is their utilization in a wide range of chemical transformations, especially cycloadditions^3^ and cyclization reactions.^4^

The asymmetric synthesis of allenes remains an underdeveloped area, with most precedent literature proceeding via S_N_2′ type reactions with enantiopure propargyl derivatives.^5^ Enantioselective coupling reactions of two simpler fragments using catalytic quantities of chiral ligand are even scarcer, due to the difficulty in controlling the stereochemistry of a three‐carbon axially chiral skeleton. Existing reports utilizing chiral ligands include the coupling of aldehydes and terminal alkynols in the presence of (*R*,*R*)‐*N*‐PINAP^6^ and the coupling of stabilized α‐diazoesters with terminal alkynes using chiral guanidinium salts (Scheme 1).^7^ Very recently, an asymmetric process was described by Wang et al. to generate trisubstituted allenes using unstabilized ketone‐derived diazo compounds (72–98 % *ee*).^8^

**Scheme 1:**
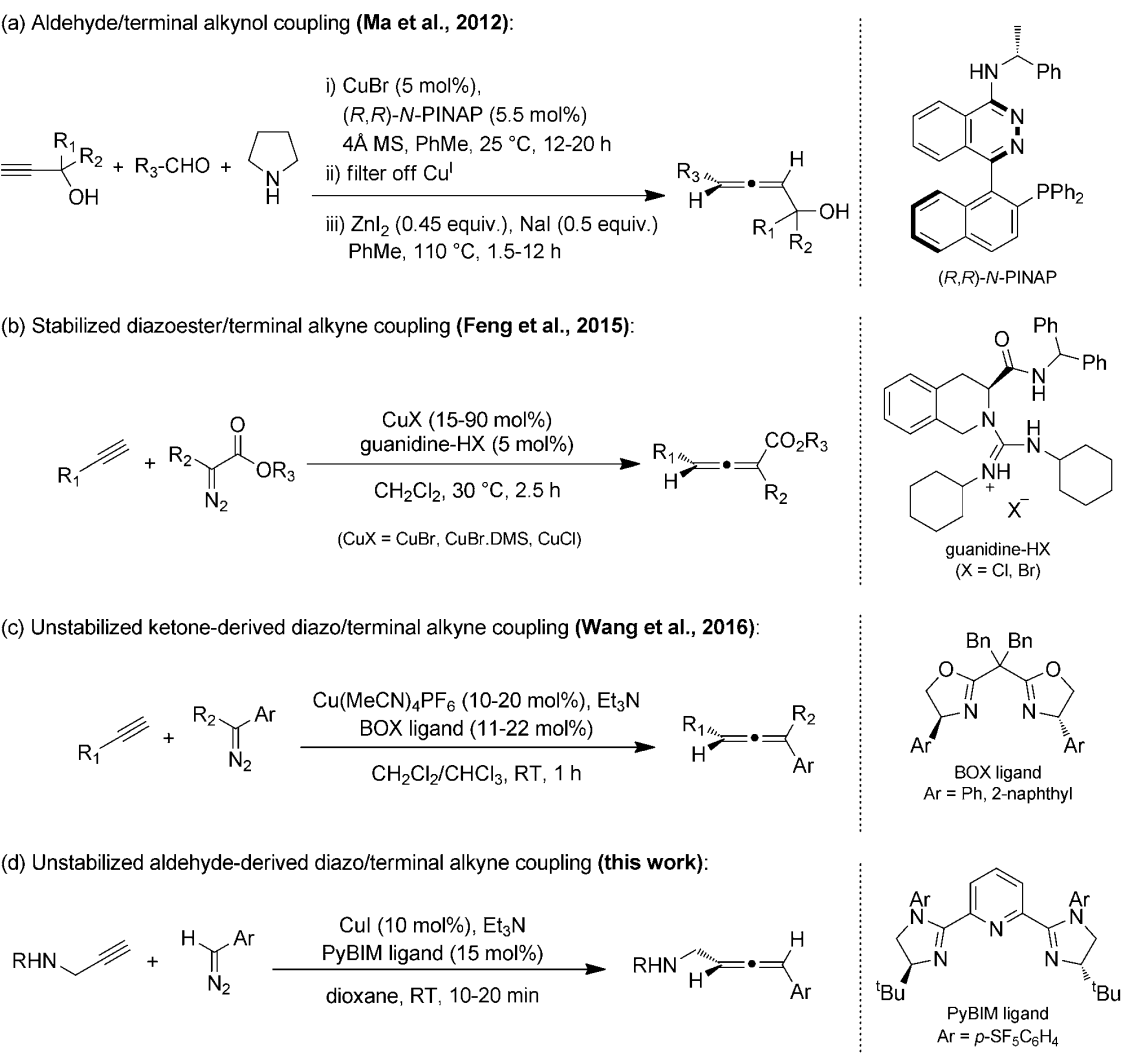
Asymmetric coupling reactions for allene synthesis using catalytic chiral ligand.

We reported earlier the room‐temperature racemic coupling of unstabilized flow‐generated diazo compounds with terminal alkynes and found the process to be extremely general, tolerating a large range of functional groups as a consequence of the mild conditions utilized for the diazo partner generation.^9^ It would therefore be highly desirable to assess whether these same benefits^10^, ^11^ would allow an enantioselective variant of the process, in the presence of an appropriate chiral ligand. We report herein a method to access chiral disubstituted allenes via flow‐generated aldehyde‐derived diazo compounds and terminal alkynes, using a new pyridinebis(imidazoline) ligand designed for this purpose.

With the knowledge that hydrazone **1 a** is able to generate the corresponding diazo compound **2 a** in the presence of activated MnO_2_ under flow conditions, an initial ligand screen was developed for the coupling with alkyne **3 a** (Table 1). We found that tridentate ligands were suitable for inducing enantioselectivity, whereas mono‐ and bidentate ligands did not result in any selectivity (see the Supporting Information (SI) for further details). These results suggest that our tridentate ligand‐mediated process for disubstituted allene synthesis is complementary to the bidentate ligand‐mediated trisubstituted allene process described by Wang et al.^8^ Interestingly, the commercially available (*S*)‐^i^Pr‐PyBOX (**L1**) provided coupling products **4 a** and **4 a′**, in a 38:62 ratio with 68 % *ee* (entry 1). Attempts at changing the structure of the PyBOX ligand offered some improvement in enantioselectivity (84 % *ee*, entry 3), though frustratingly, the desired allene remained a minor product of the reaction.

**Table 1:** Ligand screening for asymmetric allene synthesis.^[a]^

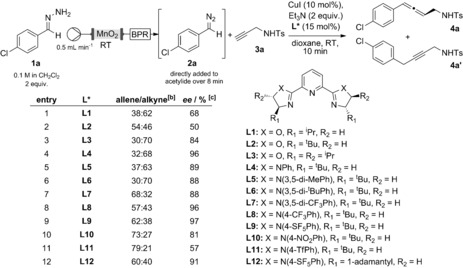

[a] Using 0.2 mmol of alkyne **3 a**, 0.02 mmol of CuI, 0.03 mmol of ligand and 0.4 mmol of Et_3_N at RT in 2 mL 1,4‐dioxane, with 0.4 mmol of hydrazone **1 a** (with DIPEA as buffer) flowed through activated MnO_2_ then a back pressure regulator (BPR). All reactions proceeded in >95 % conversion. [b] Allene/alkyne ratio determined by ^1^H NMR analysis of the crude reaction mixture. [c] *ee* determined by chiral HPLC.

Fortunately, switching the flanking oxazoline units to imidazolines (PyBIMs) proved fruitful, allowing an improvement in the enantioselectivity (96 % *ee*, entry 4). In particular, *para*‐substituted electron‐withdrawing groups on the *N*‐aryl substituent proved essential to providing allene **4 a** as the major product, with the 4‐SF_5_ group providing the best result (entry 9). Further increases in the electron deficiency (4‐nitro and 4‐triflyl, entries 10 and 11) of the aromatic ring provided moderate enantioselectivities, presumably due to poorer binding to the copper center. We believe that the ambidentate oxazoline units of the more commonly used PyBOX ligands are the origin of this differing efficiency in enantioselectivity, as coordination to the oxygen atom could lead to poorer enantioselection (see SI for a detailed discussion). In contrast, PyBIM ligands were superior as they offer only a single binding site on the imidazoline units. Furthermore, modulation of electronic effects was critical to achieve correct coupling selectivity. To the best of our knowledge, this is the first rationalization of the performance of PyBOX versus PyBIM ligands,^12^ which could be important for future development of new catalytic asymmetric reactions.

With the new ligand in hand, we then tested a variety of propargylated amine derivatives and flow‐generated diazo compounds for the asymmetric allenylation reaction (Table 2). Overall although yields in general are moderate (30–57 %), the enantioselectivity of the reaction is excellent (89–98 % *de*/*ee*) and proceeds rapidly under mild conditions. The remaining mass balance was mainly due to the alkyne cross‐coupled product, which could generally be removed by careful column chromatography. A myriad of sensitive functional groups were found to be compatible with the reaction conditions, including ketones (**4 c**), aldehydes (**4 d**), epoxides (**4 e**), unprotected alcohols (**4 i**), esters (**4 l**, **4 u**) and terminal/internal olefins (**4 s**, **4 t** and **4 v**), all with excellent enantioselectivities. Notably, several heterocyclic examples including thiophenes (**4 f**), furans (**4 j**), indoles (**4 ac**) and pyrroles (**4 ad**) were also tolerated despite their potential to interfere with the catalytic cycle, all with good enantioselectivity. Variation in the electronic properties of the diazo compound had a small effect on the yield and enantioselectivity, with more electron‐withdrawing substituents such as 4‐fluoro (**4 o**) and 3‐cyano (**4 q**) leading to slightly lower *ee* values (91 % *ee* and 89 % *ee*, respectively, compared to 94–96 % *ee* for **4 m**, **4 n**, **4 p** and **4 r**). It was also easy to scale up the asymmetric allenylation to 5 mmol of propargylamide for allene **4 w** in our flow system without the need to store excessive quantities of diazo compound, which provided 0.89 g of chiral material in similar yield and *ee* to the smaller scale run.

**Table 2:** Scope of asymmetric allenylation reaction.^[a]^

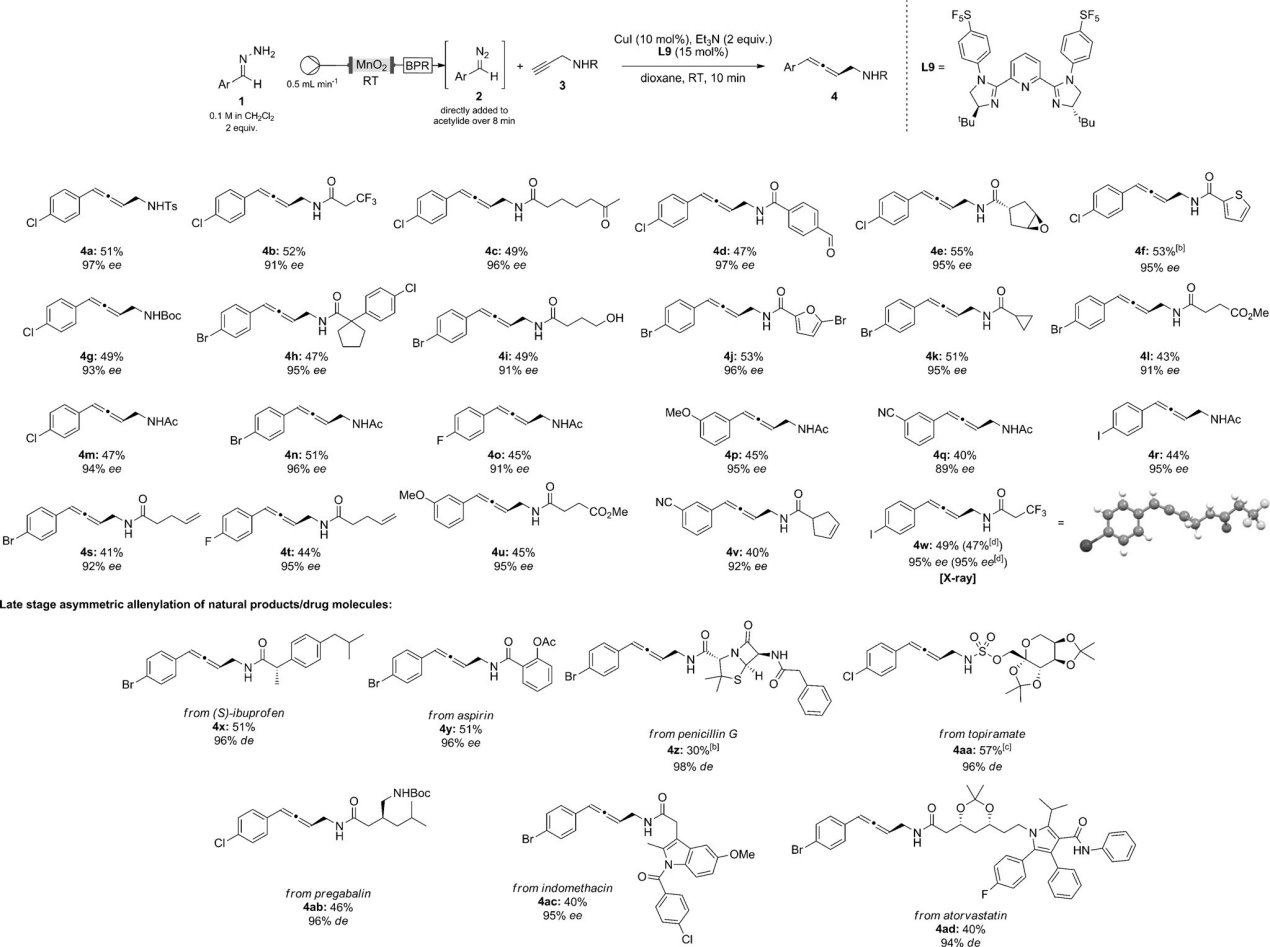

[a] Standard reaction conditions: 0.2 mmol of alkyne, 0.02 mmol of CuI, 0.03 mmol of ligand **L9** and 0.4 mmol of Et_3_N at RT in 2 mL 1,4‐dioxane, with 0.4 mmol of hydrazone (with DIPEA as buffer) flowed through activated MnO_2_ then a back pressure regulator (BPR), see SI for more detail; yields stated are of isolated product; *de/ee* determined by chiral HPLC; absolute configuration of allene **4 w** determined by X‐ray crystallography, others assigned by analogy. [b] Stirred for 20 min after addition of diazo compound. [c] Yield by ^1^H NMR using 1,4‐dinitrobenzene as internal standard. [d] Conducted on 5 mmol scale with respect to propargylamide.

To further probe the functional group tolerance of this procedure, we were able to conduct the late‐stage asymmetric allenylation of various propargylated amine derivatives of seven drug molecules/natural products (**4 x**–**4 ad**), including (*S*)‐ibuprofen, aspirin, penicillin G, topiramate, pregabalin, indomethacin and atorvastatin, again all proceeding with high *de/ee* values. Surprisingly, the presence of the thioether functionality on penicillin G was not detrimental to the stereoselectivity.

We were able to demonstrate the utility of the chiral allenes generated in a medicinal chemistry context by conducting the silver‐mediated cyclization^13^ of allene **4 a** to its corresponding 3‐pyrroline **5** (Scheme 2), with good chirality transfer and excellent yield (97 % *ee* to 95 % *ee*). The process can therefore overall be regarded as a formal enantioselective sp^2^–sp^3^ coupling, with a functional handle (internal olefin) for potential further derivatization.

**Scheme 2:**
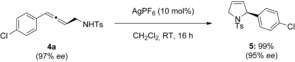
Silver‐mediated cyclization of allene **4 a** to 3‐pyrroline **5**.

We anticipate that the reaction mechanism proceeds as depicted below (Scheme 3). In the presence of base and the copper catalyst, the initially generated ligand–copper acetylide complex **7** undergoes an enantioselective concerted Cu−C bond insertion with the diazo compound **2**. The approach of this species is controlled by the bulky ^t^Bu arms of the PyBIM ligand, with the hydrogen substituent of the diazo compound on the same side as the ligand ^t^Bu groups. Selective 1,3‐protonation (most likely via the N−H group from the amide/sulfonyl group) of the transient copper species **8** from the front face of the catalyst then generates the desired allene **4** and regenerating the ligand–copper complex **6**. It appears that the copper species **8** is fairly labile and can dissociate leading to the anionic species **9**, which subsequently protonates to give the undesired alkyne cross‐coupled product **4′**.^14^

**Scheme 3:**
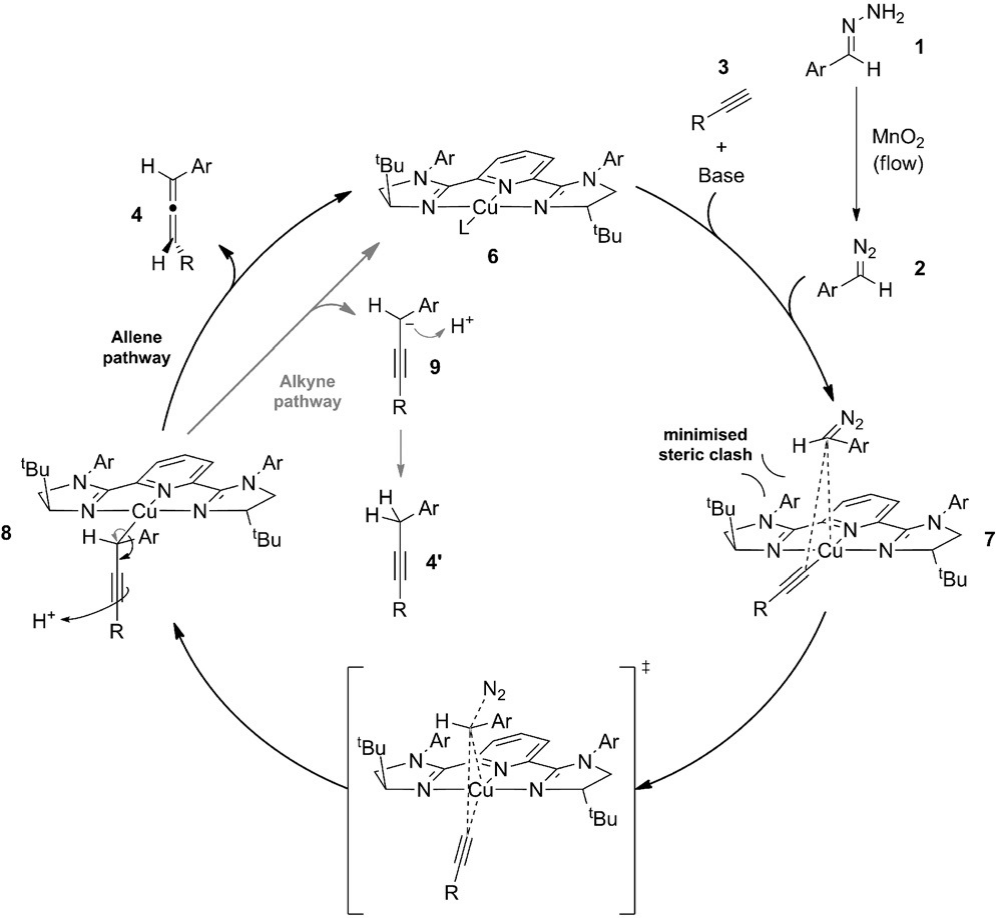
Proposed reaction mechanism for asymmetric diazo–alkyne coupling reaction.

In summary, we have reported a new flow‐enabled catalytic asymmetric coupling reaction of aldehyde‐derived unstabilized diazo compounds with propargylamine derivatives, proceeding rapidly in moderate yields and high enantioselectivities. Given the underdeveloped methods for enantioselective allene synthesis, our results represent an important achievement in this field, particularly due to the high level of functional group tolerance and potential for late‐stage functionalization. Further investigations into generalizing the scope and the development of new bifunctional ligands are currently ongoing in our laboratories.

## Conflict of interest

The authors declare no conflict of interest.

## Supporting information

As a service to our authors and readers, this journal provides supporting information supplied by the authors. Such materials are peer reviewed and may be re‐organized for online delivery, but are not copy‐edited or typeset. Technical support issues arising from supporting information (other than missing files) should be addressed to the authors.

Supplementary
