# Supplementary material for: Rapid Asymmetric Synthesis of Disubstituted Allenes by Coupling of Flow‐Generated Diazo Compounds and Propargylated Amines
Source: Angew Chem Int Ed Engl. 2017 Jan 11;56(7):1864–8. doi: 10.1002/anie.201611067 (PMC5363227; doi:10.1002/anie.201611067)
Supplement: Supplementary file 1 — Supplementary [file ANIE-56-1864-s001.pdf]

Angewandte Chemie
Eine Zeitschrift der Gesellschaft Deutscher Chemiker

Supporting Information

Rapid Asymmetric Synthesis of Disubstituted Allenes by Coupling of Flow-Generated Diazo Compounds and Propargylated Amines

*Jian-Siang Poh, Szabolcs Makai, Timo von Keutz, Duc N. Tran, Claudio Battilocchio, Patrick Pasau, and Steven V. Ley**

anie_201611067_sm_miscellaneous_information.pdf

Contents

1. General experimental details....3
2. Synthetic procedures and characterisation for new ligands....5
   2.1. PyBOX ligands....5
   2.2. PyBIM ligands....15
3. Synthetic procedures and characterisation for alkyne starting materials....35
4. Synthetic procedures and characterisation for hydrazone starting materials....47
5. Optimisation and discussion....50
   5.1. Ligand list....50
   5.2. Optimisation table....52
6. Synthetic procedures and characterisation for asymmetric allenylation....53
7. Synthetic procedure and characterisation for silver-mediated cyclisation....88
8. NMR spectra....89
   8.1. NMR spectra for PyBOX ligands....89
   8.2. NMR spectra for PyBIM ligands....107
   8.3. NMR spectra for alkyne starting materials....150
   8.4. NMR spectra for hydrazone starting materials....174
   8.5. NMR spectra for chiral allenes....181
   8.6. NMR spectra for alkyne cross-products....217
   8.7. NMR spectra for pyrroline....253
9. HPLC spectra....254
References....286

1. General experimental details

All batch reactions were performed using oven-dried glassware (200 °C) under an atmosphere of argon unless otherwise stated. All flow reactions were performed using a Uniqsis FlowSyn platform[1] or a Vapourtec R2+R4 system.[2] Solvents were freshly distilled over sodium benzophenone ketyl (THF, Et_2O) or calcium hydride (acetone, MeCN, MeOH, CH_2Cl_2, $CHCl_3$, toluene, hexane and EtOAc). Additional anhydrous solvents were obtained from commercial sources and used directly (DMF, DMA, DMSO and 1,4-dioxane). DIPEA and Et_3N were freshly distilled over calcium hydride and stored over 4 Å molecular sieves. All reagents were obtained from commercial sources and used without further purification.

Flash column chromatography was performed using high-purity grade silica gel (Merck grade 9385) with a pore size 60 Å and 230–400 mesh particle size under air pressure. Analytical thin layer chromatography (TLC) was performed using silica gel 60 F_{254} pre-coated glass backed plates and visualized by ultraviolet radiation (254 nm) and/or potassium permanganate solution as appropriate.

^{1}H NMR spectra were recorded on a 600 MHz Avance 600 BBI Spectrometer as indicated. Chemical shifts are reported in ppm with the resonance resulting from incomplete deuteration of the solvent as the internal standard ($CDCl_3$: 7.26 ppm). ^{13}C NMR spectra were recorded the same spectrometer with complete proton decoupling. Chemical shifts are reported in ppm with the solvent resonance as the internal standard ($^{13}CDCl_3$: 77.16 ppm, t). ^{19}F NMR spectra were recorded on a 376 MHz Avance III HD Spectrometer with complete proton decoupling. Chemical shifts are reported in ppm with $CFCl_3$ as the external standard ($CFCl_3$: 0.00 ppm). Data are reported as follows: chemical shift δ/ppm, integration (^{1}H only), multiplicity (s = singlet, d = doublet, t = triplet, q = quartet, qn = quintet, sept = septet, oct = octet, br = broad, m = multiplet or combinations thereof; ^{13}C signals are singlets unless otherwise stated), coupling constants *J* in Hz, assignment. Spectra are assigned as fully as possible, using ^{1}H-COSY, DEPT-135, HMQC and HMBC where appropriate to facilitate structural determination. Signals that cannot be unambiguously assigned are reported with all possible assignments separated by a slash (e.g. H1/H2) or descriptions of their environments (e.g. ArH, NH, OH). Multiple signals arising from diastereotopic or (pseudo)axial/equatorial positions are suffixed alphabetically (e.g. H1a, H1b). Overlapping signals that cannot be resolved are reported with their assignments denoted in list format (e.g. H1, H2 and H3). ^{1}H NMR signals are reported to 2 decimal places and ^{13}C signals to 1 decimal place unless rounding would produce a value identical to another signal. In this case, an additional decimal place is reported for both signals concerned.

Infrared spectra were recorded neat as thin films on a Perkin-Elmer Spectrum One FTIR spectrometer and selected peaks are reported (s = strong, m = medium, w = weak, br = broad).

High resolution mass spectrometry (HRMS) was performed using positive electrospray ionisation (ESI+), on either a Waters Micromass LCT Premier spectrometer or performed by the Mass Spectrometry Service for the Chemistry Department at the University of Cambridge. All *m/z* values are reported to 4 decimal places and are within ± 5 ppm of theoretical values.

Specific optical rotation was recorded on a Perkin-Elmer Model 343 digital polarimeter, using a Na/Hal lamp set at 589 nm and with a path length of 100 mm. $[\alpha]_D$ values were measured using spectroscopy grade solvent at the specified concentration (in g/100 mL) and temperature, with units of deg 10^{-1} cm^2 g^{-1}.

Melting points were recorded on a Stanford Research Systems OptiMelt Automated Melting Point System calibrated against vanillin (m.p. 83 °C), phenacetin (m.p. 136 °C) and caffeine (m.p. 237 °C).

Chiral HPLC analysis was conducted on an Agilent 1100 Series Chromatography system using mixtures of hexane/isopropanol as eluent on Chiralpak AS, Chiralpak OD-H, ChiralART SA or ChiralART SC columns.

2. Synthetic procedures and characterisation for new ligands

2.1. PyBOX ligands

General procedure for PyBOX synthesis:[3] A solution of the appropriate 2,6-pyridinedicarbonitrile (1.0 mmol, 1 equiv.), the appropriate amino alcohol (2.0 mmol, 2 equiv.) and zinc(II) triflate (36 mg, 0.1 mmol, 0.1 equiv.) in toluene (15 mL) was heated under reflux for 48 h. The reaction mixture was then cooled to r.t. and diluted with EtOAc (10 mL). The organic layer was washed with brine (3 × 25 mL), saturated aqueous $NaHCO_3$ solution (3 × 25 mL), dried ($MgSO_4$) and evaporated under reduced pressure. The residue was further purified by silica gel column chromatography or recrystallisation as appropriate.

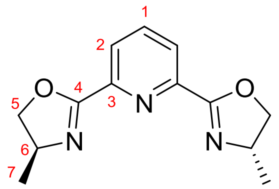

2,6-bis((*S*)-4-methyl-4,5-dihydrooxazol-2-yl)pyridine: Following the general procedure for PyBOX synthesis, using 2,6-pyridinedicarbonitrile (0.129 g, 1.0 mmol, 1 equiv.) and (*S*)-2-aminopropan-1-ol (0.286 g, 2.0 mmol, 2 equiv.), purified by silica gel column chromatography (eluent: 5% $MeOH/CH_2Cl_2$) provided the title compound as a white amorphous solid (0.022 g, 0.09 mmol, 9%), m.p. 148-151 °C (lit. m.p.[4] 163.0-165.5 °C, on material with 0.25 mol H_2O). Data are consistent with a reported example.[4]

^{1}H NMR (600 MHz, $CDCl_3$): δ 8.17 (d, *J* = 7.8 Hz, 2 H, H2), 7.86 (t, *J* = 7.8 Hz, 1 H, H1), 4.61 (dd, *J* = 9.5, 8.3 Hz, 2 H, H5a), 4.43 (ddq, *J* = 9.5, 8.3, 6.7 Hz, 2 H, H6), 4.07 (t, *J* = 8.3 Hz, 2 H, H5b), 1.37 (d, *J* = 6.7 Hz, 6 H, H7).
^{13}C NMR (150 MHz, $CDCl_3$): δ 162.4 (C4), 147.0 (C3), 137.4 (C1), 125.8 (C2), 74.9 (C5), 62.4 (C6), 21.5 (C7).
FTIR (ν_{max}, cm^{-1}): 2971 (m), 2900 (w), 1641 (s), 1575 (s), 1531 (w), 1459 (m), 1385 (m), 1305 (w), 1269 (w), 1246 (w), 1171 (w), 1119 (m), 1068 (s), 973 (s), 945 (m), 831 (w).
HRMS (ESI): calculated for $C_{13}H_{16}N_3O_2$ $[M+H]^+$ 246.1237, found 246.1231.
R_f = 0.26 (5% $MeOH/CH_2Cl_2$).
$[\alpha]_D^{28.3}$ = -127.6 ($CHCl_3$, c = 0.5); lit.[4] $[\alpha]_D$ = -131.8 ($CHCl_3$, c = 0.72).

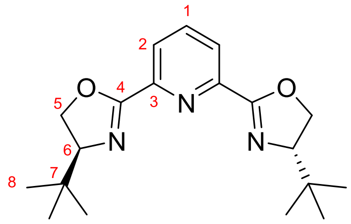

2,6-bis((S)-4-(*tert*-butyl)-4,5-dihydrooxazol-2-yl)pyridine (L2): Following the general procedure for PyBOX synthesis, using 2,6-pyridinedicarbonitrile (0.387 g, 3.0 mmol, 1 equiv.) and L-*tert*-leucinol (0.703 g, 6.0 mmol, 2 equiv.), after extraction and evaporation provided the title compound as a white crystalline solid (0.970 g, 2.9 mmol, 98%), m.p. 236-238 °C. Data are consistent with a reported example.[5]

^{1}H NMR (600 MHz, $CDCl_3$): δ 8.25 (d, *J* = 7.8 Hz, 2 H, H2), 7.84 (t, *J* = 7.8 Hz, 1 H, H1), 4.47 (dd, *J* = 10.3, 8.6 Hz, 2 H, H5a), 4.32 (t, *J* = 8.6 Hz, 2 H, H6), 4.10 (dd, *J* = 10.3, 8.6 Hz, 2 H, H5b), 0.96 (s, 18 H, H8).
^{13}C NMR (150 MHz, $CDCl_3$): δ 162.3 (C4), 147.1 (C3), 137.2 (C1), 126.0 (C2), 76.5 (C6), 69.6 (C5), 34.1 (C7), 26.1 (C8).
FTIR (ν_{max}, cm^{-1}): 2957 (m), 2902 (w), 2869 (w), 1641 (s), 1590 (w), 1569 (m), 1475 (m), 1459 (m), 1417 (w), 1395 (m), 1377 (s), 1363 (s), 1319 (w), 1276 (m), 1212 (m), 1195 (w), 1153 (w), 1106 (s), 1075 (m), 1057 (m), 1032 (m), 997 (m), 967 (m), 956 (m), 931 (m), 899 (w), 865 (w), 841 (m), 757 (m).
HRMS (ESI): calculated for $C_{19}H_{27}N_3O_2Na$ $[M+Na]^+$ 352.1995, found 352.2005.
$[\boldsymbol{\alpha}]_D^{24.0}$ = -115.0 (CH_2Cl_2, c = 1.0); lit.[5] $[\boldsymbol{\alpha}]_D^{27}$ = -118 (CH_2Cl_2, c = 0.5).

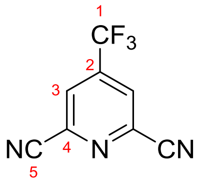

4-(trifluoromethyl)pyridine-2,6-dicarbonitrile: To a 20 mL microwave vial was added 2,6-dichloro-4-(trifluoromethyl)pyridine (1.080 g, 5.0 mmol, 1.0 equiv.), zinc(II) cyanide (0.646 g, 5.5 mmol, 1.1 equiv.), zinc powder (65 mg, 1.0 mmol, 0.2 equiv.), palladium(II) trifluoroacetate (83 mg, 0.25 mmol, 0.05 equiv.), *rac*-2-(di-*tert*-butylphosphino)-1,1'-binaphthyl (0.199 g, 0.50 mmol, 0.1 equiv.) and anhydrous DMA (20 mL). The mixture was placed under vacuum and backfilled with argon (three cycles), stirred at r.t. for 20 min, then subsequently heated to 95 °C for 16 h. The mixture was then cooled to r.t. and filtered through a pad of Celite, eluting with EtOAc. The solvents were removed by vacuum distillation (6-10 mmHg) and the residue purified by silica gel column chromatography (eluent: 10% EtOAc/hexane) to provide the title compound as a pale yellow amorphous solid (0.620 g, 3.1 mmol, 63%), m.p. 103-104 °C.

^{1}H NMR (600 MHz, $CDCl_3$): δ 8.12 (q, *J* = 0.6 Hz, 2 H, H3).
^{13}C NMR (150 MHz, $CDCl_3$): δ 142.0 (q, *J* = 36.8 Hz, C2), 136.7 (C4), 127.0 (q, *J* = 3.4 Hz, C3), 120.9 (q, *J* = 274.8 Hz, C1), 114.5 (C5).
^{19}F NMR (376 MHz, $CDCl_3$): δ -64.8 (s, 3 F, F1).
FTIR (ν_{max}, cm^{-1}): 3069 (w), 1870 (w), 1567 (w), 1416 (w), 1403 (m), 1352 (m), 1304 (w), 1250 (w), 1222 (m), 1212 (m), 1153 (s), 1105 (m), 981 (w), 925 (m), 863 (m), 792 (w).
HRMS (ESI): calculated for $C_8H_3F_3N_3$ $[M+H]^+$ 198.0274, found 198.0265.
$\boldsymbol{R_f}$ = 0.27 (10% EtOAc/hexane).

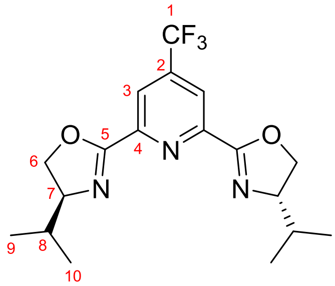

(4*S*,4'*S*)-2,2'-(4-(trifluoromethyl)pyridine-2,6-diyl)bis(4-isopropyl-4,5-dihydrooxazole): Following the general procedure for PyBOX synthesis, using 4-(trifluoromethyl)pyridine-2,6-dicarbonitrile (0.197 g, 1.0 mmol, 1 equiv.) and L-valinol (0.206 g, 2.0 mmol, 2 equiv.),

purified by silica gel column chromatography (eluent: 5% acetone/CH_2Cl_2) provided the title compound as an off-white amorphous solid (0.179 g, 0.48 mmol, 48%), m.p. 90-93 °C.

^{1}H NMR (600 MHz, $CDCl_3$): δ 8.42 (s, 2 H, H3), 4.55 (dd, *J* = 9.8, 8.5 Hz, 2 H, H6a), 4.25 (t, *J* = 8.5 Hz, 2 H, H6b), 4.16 (ddd, *J* = 9.8, 8.5, 6.7 Hz, 2 H, H7), 1.92 – 1.82 (oct, *J* = 6.7 Hz, 2 H, H8), 1.04 (d, *J* = 6.7 Hz, 6 H, H9/H10), 0.93 (d, *J* = 6.7 Hz, 6 H, H9/H10).
^{13}C NMR (150 MHz, $CDCl_3$): δ 161.5 (C5), 148.4 (C4), 139.9 (q, *J* = 35.0 Hz, C2), 122.3 (q, *J* = 273.7 Hz, C1), 121.6 (q, *J* = 3.5 Hz, C3), 73.2 (C7), 71.4 (C6), 32.9 (C8), 19.1 (C9/C10), 18.4 (C9/C10).
^{19}F NMR (376 MHz, $CDCl_3$): δ -64.6 (s, 3 F, F1).
FTIR (ν_{max}, cm^{-1}): 2961 (w), 2874 (w), 1670 (w), 1644 (m), 1608 (w), 1574 (w), 1467 (w), 1443 (w), 1413 (w), 1386 (w), 1368 (w), 1356 (w), 1337 (w), 1294 (m), 1277 (m), 1259 (m), 1174 (m), 1141 (s), 1112 (m), 1041 (w), 1020 (w), 978 (m), 961 (m), 938 (m), 907 (m), 819 (w), 768 (w), 753 (w).
HRMS (ESI): calculated for $C_{18}H_{23}F_3N_3O_2$ $[M+H]^+$ 370.1737, found 370.1742.
R_f = 0.29 (5% acetone/CH_2Cl_2).
$[\alpha]_D^{28.3}$ = -124.8 ($CHCl_3$, c = 1.0).

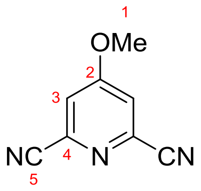

4-methoxypyridine-2,6-dicarbonitrile: To a 20 mL microwave vial was added 2,6-dichloro-4-methoxypyridine (0.890 g, 5.0 mmol, 1.0 equiv.), zinc(II) cyanide (0.646 g, 5.5 mmol, 1.1 equiv.), zinc powder (65 mg, 1.0 mmol, 0.2 equiv.), palladium(II) trifluoroacetate (83 mg, 0.25 mmol, 0.05 equiv.), *rac*-2-(di-*tert*-butylphosphino)-1,1'-binaphthyl (0.199 g, 0.50 mmol, 0.1 equiv.) and anhydrous DMA (20 mL). The mixture was placed under vacuum and backfilled with argon three times, stirred at r.t. for 20 min, then subsequently heated to 95 °C for 16 h. The mixture was then cooled to r.t. and filtered through a pad of Celite, eluting with EtOAc. The solvents were removed by vacuum distillation (6-10 mmHg) and the residue purified by silica gel column chromatography (eluent: 40% EtOAc/hexane) to provide the title compound as a white amorphous solid (0.730 g, 4.6 mmol, 92%), m.p. 147-149 °C. Data are consistent with a reported example.[6]

^{1}H NMR (600 MHz, $CDCl_3$): δ 7.39 (s, 2 H, H3), 3.99 (s, 3 H, H1).
^{13}C NMR (150 MHz, $CDCl_3$): δ 167.0 (C2), 136.3 (C4), 117.9 (C3), 115.7 (C5), 56.9 (C1).
FTIR (ν_{max}, cm^{-1}): 3087 (w), 3074 (w), 2243 (w), 1589 (s), 1558 (m), 1474 (m), 1437 (m), 1338 (s), 1275 (w), 1211 (w), 1194 (m), 1172 (w), 1155 (s), 1046 (s), 984 (s), 943 (w), 916 (m), 891 (s).
HRMS (ESI): calculated for $C_8H_6N_3O$ $[M+H]^+$ 160.0505, found 160.0502.
R_f = 0.43 (40% EtOAc/hexane).

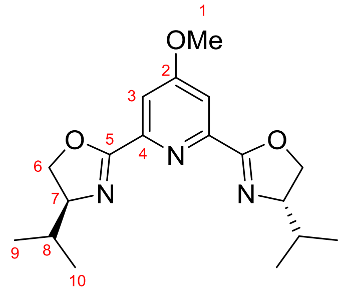

(4*S*,4'*S*)-2,2'-(4-methoxypyridine-2,6-diyl)bis(4-isopropyl-4,5-dihydrooxazole): Following the general procedure for PyBOX synthesis, using 4-methoxypyridine-2,6-dicarbonitrile (0.159 g, 1.0 mmol, 1 equiv.) and L-valinol (0.206 g, 2.0 mmol, 2 equiv.), purified by silica gel column chromatography (eluent: 3% MeOH/CH_2Cl_2) provided the title compound as a white amorphous solid (0.216 g, 0.65 mmol, 65%), m.p. 60-62 °C (lit. m.p. [7] 83-84 °C, on material with 0.5 mol H_2O). Data are consistent with a reported example.[7]

^{1}H NMR (600 MHz, CDCl$_3$): δ 7.72 (s, 2 H, H3), 4.50 (dd, *J* = 9.8, 8.5 Hz, 2 H, H6a), 4.20 (t, *J* = 8.5 Hz, 2 H, H6b), 4.11 (ddd, *J* = 9.8, 8.5, 6.7 Hz, 2 H, H7), 3.93 (s, 3 H, H1), 1.85 (oct, *J* = 6.7 Hz, 2 H, H8), 1.02 (d, *J* = 6.7 Hz, 6 H, H9/H10), 0.91 (d, *J* = 6.7 Hz, 6 H, H9/H10).
^{13}C NMR (150 MHz, CDCl$_3$): δ 166.6 (C2), 162.4 (C5), 148.4 (C4), 111.8 (C3), 72.9 (C7), 71.0 (C6), 56.0 (C1), 32.9 (C8), 19.2 (C9/C10), 18.3 (C9/C10).
FTIR (ν_{max}, cm^{-1}): 2964 (w), 2876 (w), 1673 (w), 1584 (m), 1533 (w), 1471 (w), 1443 (w), 1380 (w), 1346 (w), 1296 (w), 1245 (m), 1214 (m), 1182 (w), 1162 (m), 1143 (w), 1106 (w), 1082 (w), 1045 (m), 970 (s), 939 (w), 886 (w), 864 (m), 806 (w), 758 (m).
HRMS (ESI): calculated for $C_{18}H_{26}N_3O_3$ $[M+H]^+$ 332.1969, found 332.1967.
R_f = 0.28 (3% MeOH/CH_2Cl_2).
$[\alpha]_D^{28.3}$ = -99.9 ($CHCl_3$, c = 1.0); lit.[7] $[\alpha]_D$ = -83.6 (CH_2Cl_2, c = 0.53).

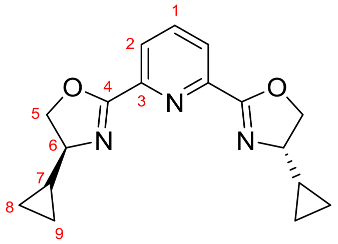

2,6-bis((*S*)-4-cyclopropyl-4,5-dihydrooxazol-2-yl)pyridine: Following the general procedure for PyBOX synthesis, using 2,6-pyridinedicarbonitrile (0.129 g, 1.0 mmol, 1 equiv.) and (*S*)-2-amino-2-cyclopropylethan-1-ol (0.202 g, 2.0 mmol, 2 equiv.), purified by silica gel column chromatography (eluent: 30% acetone/CH_2Cl_2) provided the title compound as a yellow amorphous solid (0.096 g, 0.32 mmol, 32%), m.p. 82-85 °C.

^{1}H NMR (600 MHz, CDCl$_3$): δ 8.22 (d, *J* = 7.8 Hz, 2 H, H2), 7.85 (t, *J* = 7.8 Hz, 1 H, H1), 4.60 (dd, *J* = 9.8, 8.3 Hz, 2 H, H5a), 4.28 (t, *J* = 8.3 Hz, 2 H, H5b), 3.82 (dt, *J* = 9.8, 8.3 Hz, 2 H, H6), 1.01 – 0.94 (m, 2 H, H7), 0.66 – 0.60 (m, 2 H, H8/H9), 0.57 – 0.51 (m, 2 H, H8/H9), 0.49 – 0.44 (m, 2 H, H8/H9), 0.36 – 0.31 (m, 2 H, H8/H9).
^{13}C NMR (150 MHz, CDCl$_3$): δ 162.9 (C4), 147.0 (C3), 137.3 (C1), 126.0 (C2), 73.4 (C5), 71.3 (C6), 16.0 (C7), 3.3 (C8/C9), 2.3 (C8/C9).
FTIR (ν_{max}, cm^{-1}): 3403 (w), 3084 (w), 3007 (w), 2900 (w), 1647 (m), 1573 (m), 1533 (w), 1447 (w), 1380 (m), 1355 (m), 1331 (m), 1250 (m), 1205 (w), 1170 (m), 1147 (w), 1106 (w), 1083 (w), 1062 (m), 1051 (m), 1025 (m), 973 (s), 939 (m), 895 (m), 828 (s), 819 (s).

HRMS (ESI): calculated for $C_{17}H_{20}N_3O_2$ $[M+H]^+$ 298.1550, found 298.1556.
R_f = 0.43 (30% acetone/CH_2Cl_2).
$[\alpha]_D^{28.3}$ = -189.4 ($CHCl_3$, c = 1.0).

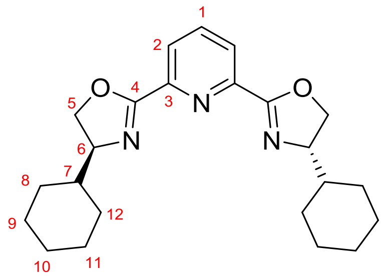

2,6-bis((*S*)-4-cyclohexyl-4,5-dihydrooxazol-2-yl)pyridine: Following the general procedure for PyBOX synthesis, using 2,6-pyridinedicarbonitrile (0.129 g, 1.0 mmol, 1 equiv.) and (*S*)-2-amino-2-cyclohexylethan-1-ol (0.286 g, 2.0 mmol, 2 equiv.), purified by recrystallisation from EtOAc provided the title compound as a white flaky solid (0.123 g, 0.32 mmol, 32%), m.p. 204-206 °C. Compound has been prepared previously,[8] but NMR spectra were recorded in CD_2Cl_2.

^{1}H NMR (600 MHz, $CDCl_3$): δ 8.19 (d, *J* = 7.9 Hz, 2 H, H2), 7.84 (t, *J* = 7.9 Hz, 1 H, H1), 4.51 (dd, *J* = 9.7, 8.5 Hz, 2 H, H5a), 4.24 (t, *J* = 8.5 Hz, 2H, H5b), 4.13 (ddd, *J* = 9.7, 8.5, 7.0 Hz, 2 H, H6), 2.00 (br d, *J* = 12.8 Hz, 2 H, H8a/H12a), 1.80 – 1.72 (m, 4 H, H9a and H11a), 1.68 (br d, *J* = 12.1 Hz, 2 H, H10a), 1.61 (br d, *J* = 12.6 Hz, 2 H, H8a/H12a), 1.57 – 1.49 (m, 2 H, H7), 1.32 – 1.16 (m, 6 H, H9b, H10b and H11b), 1.13 – 1.01 (m, 4 H, H8b and H12b).
^{13}C NMR (150 MHz, $CDCl_3$): δ 162.3 (C4), 147.1 (C3), 137.3 (C1), 125.8 (C2), 72.2 (C6), 71.3 (C5), 43.0 (C7), 29.8 (C8/C12), 29.1 (C8/C12), 26.6 (C10), 26.2 (C9 and C11).
FTIR (ν_{max}, cm^{-1}): 2919 (m), 2850 (m), 1629 (s), 1569 (w), 1451 (w), 1414 (w), 1379 (s), 1326 (w), 1282 (w), 1228 (w), 1101 (s), 1072 (s), 1057 (w), 998 (w), 980 (w), 962 (s), 941 (w), 910 (w), 885 (w), 843 (w), 756 (w).
HRMS (ESI): calculated for $C_{23}H_{32}N_3O_2$ $[M+H]^+$ 382.2489, found 382.2498.
$[\alpha]_D^{28.3}$ = -162.8 ($CHCl_3$, c = 1.0).

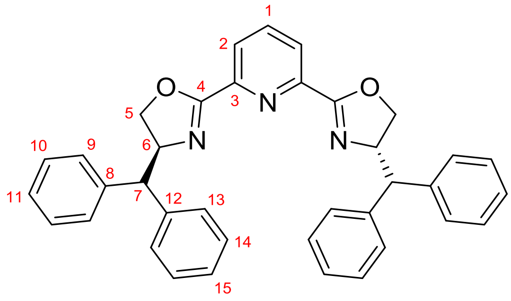

2,6-bis((*S*)-4-benzhydryl-4,5-dihydrooxazol-2-yl)pyridine: Following the general procedure for PyBOX synthesis, using 2,6-pyridinedicarbonitrile (0.129 g, 1.0 mmol, 1 equiv.) and (*S*)-2-amino-3,3-diphenylpropan-1-ol (0.455 g, 2.0 mmol, 2 equiv.), purified by recrystallisation from toluene and washed on the filter with cold EtOAc (2 mL) provided the title compound as an off-white flaky solid (0.195 g, 0.35 mmol, 35%), m.p. 262-264 °C.

^{1}H NMR (600 MHz, $CDCl_3$): δ 8.17 (d, *J* = 7.9 Hz, 2 H, H2), 7.78 (t, *J* = 7.9 Hz, 1 H, H1), 7.37 – 7.13 (m, 20 H, ArH), 5.17 (td, *J* = 9.3, 8.5 Hz, 2 H, H6), 4.54 (appears t, *J* = 9.3 Hz, 2 H, H5a), 4.20 (appears t, *J* = 8.5 Hz, 2 H, H5b), 4.07 (d, *J* = 9.3 Hz, 2 H, H7).

^{13}C NMR (150 MHz, $CDCl_3$): δ 163.0 (C4), 146.9 (C3), 142.2 (C8/C12), 142.0 (C8/C12), 137.2 (C1), 128.9 (ArCH), 128.8 (ArCH), 128.7 (ArCH), 128.5 (ArCH), 127.0 (C11/C15), 126.6 (C11/C15), 126.3 (C2), 72.3 (C5), 70.5 (C6), 57.1 (C7).
FTIR (ν_{max}, cm^{-1}): 1638 (m), 1571 (w), 1495 (w), 1452 (w), 1379 (s), 1276 (w), 1108 (s), 1073 (m), 1031 (w), 975 (w), 830 (w).
HRMS (ESI): calculated for $C_{37}H_{32}N_3O_2$ $[M+H]^+$ 550.2489, found 550.2506.
$[\alpha]_D^{28.3}$ = -93.1 ($CHCl_3$, c = 1.0).

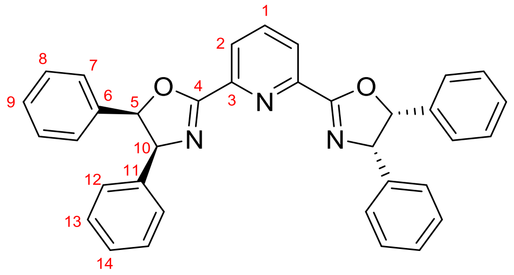

2,6-bis((4*S*,5*R*)-4,5-diphenyl-4,5-dihydrooxazol-2-yl)pyridine: Following the general procedure for PyBOX synthesis, using 2,6-pyridinedicarbonitrile (0.129 g, 1.0 mmol, 1 equiv.) and (1*R*,2*S*)-2-amino-1,2-diphenylethan-1-ol (0.427 g, 2.0 mmol, 2 equiv.), purified by silica gel column chromatography (eluent: 2% $MeOH/CH_2Cl_2$) and then recrystallisation from CH_2Cl_2/hexane provided the title compound as a white crystalline solid (0.091 g, 0.18 mmol, 18%), m.p. 233-236 °C (lit. m.p.[9] 225-226 °C). Data are consistent with a reported example.[9]

^{1}H NMR (600 MHz, $CDCl_3$): δ 8.44 (d, *J* = 7.9 Hz, 2 H, H2), 8.04 (t, *J* = 7.9 Hz, 1 H, H1), 7.08 – 6.95 (m, 20 H, ArH), 6.14 (d, *J* = 10.3 Hz, 2 H, H5), 5.83 (d, *J* = 10.3 Hz, 2 H, H10).
^{13}C NMR (150 MHz, $CDCl_3$): δ 164.1 (C4), 147.2 (C3), 137.8 (C1), 137.3 (C6/C11), 136.1 (C6/C11), 128.0 (C7/C8/C12/C13), 127.81 (C7/C8/C12/C13), 127.76 (C7/C8/C12/C13), 127.6 (C9/C14), 127.2 (C9/C14), 126.7 (C7/C8/C12/C13), 126.6 (C2), 86.4 (C5), 74.5 (C10).
FTIR (ν_{max}, cm^{-1}): 1664 (w), 1630 (m), 1572 (m), 1495 (w), 1453 (m), 1357 (w), 1321 (m), 1261 (w), 1244 (w), 1211 (w), 1169 (m), 1141 (m), 1108 (m), 1077 (s), 1028 (w), 995 (w), 968 (s), 921 (m), 888 (w), 859 (w), 821 (m), 765 (s).
HRMS (ESI): calculated for $C_{35}H_{28}N_3O_2$ $[M+H]^+$ 522.2176, found 522.2181.
R_f = 0.18 (2% $MeOH/CH_2Cl_2$).
$[\alpha]_D^{28.3}$ = -293.7 ($CHCl_3$, c = 1.0); lit.[9] $[\alpha]_D$ = -305 ($CHCl_3$, c = 0.5).

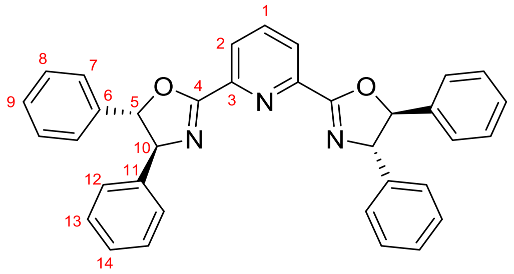

2,6-bis((4*S*,5*S*)-4,5-diphenyl-4,5-dihydrooxazol-2-yl)pyridine: Following the general procedure for PyBOX synthesis, using 2,6-pyridinedicarbonitrile (0.129 g, 1.0 mmol, 1 equiv.) and (1*S*,2*S*)-2-amino-1,2-diphenylethan-1-ol (0.427 g, 2.0 mmol, 2 equiv.), purified by silica gel column chromatography (eluent: 2% $MeOH/CH_2Cl_2$) provided the title compound as a white amorphous solid (0.088 g, 0.17 mmol, 17%), m.p. 200-201 °C (lit. m.p.[10] 201-202 °C). Data are consistent with a reported example.[10]

^{1}H NMR (600 MHz, $CDCl_3$): δ 8.41 (d, *J* = 7.9 Hz, 2 H, H2), 7.98 (t, *J* = 7.9 Hz, 1 H, H1), 7.41 – 7.30 (m, 20 H, ArH), 5.56 (d, *J* = 8.4 Hz, 2 H, H5), 5.35 (d, *J* = 8.4 Hz, 2 H, H10).
^{13}C NMR (150 MHz, $CDCl_3$): δ 163.0 (C4), 147.1 (C3), 141.3 (C6/C11), 139.8 (C6/C11), 137.5 (C1), 128.9 (C7/C8/C12/C13), 128.9 (C7/C8/C12/C13), 128.6 (C9/C14), 127.9 (C9/C14), 126.9 (C7/C8/C12/C13), 126.7 (C2), 126.3 (C7/C8/C12/C13), 90.1 (C5), 79.1 (C10).
FTIR (ν_{max}, cm^{-1}): 1648 (m), 1600 (w), 1568 (m), 1493 (w), 1448 (m), 1341 (w), 1288 (m), 1258 (m), 1222 (w), 1162 (m), 1123 (m), 1080 (m), 1025 (w), 1012 (w), 978 (s), 956 (s), 915 (w), 890 (w), 846 (m), 758 (s).
HRMS (ESI): calculated for $C_{35}H_{27}N_3O_2Na$ $[M+Na]^+$ 544.1995, found 544.2006.
R_f = 0.26 (2% MeOH/CH_2Cl_2).
$[\alpha]_D^{28.3}$ = -37.8 ($CHCl_3$, c = 1.0); lit.[10] $[\alpha]_D^{20}$ = -41.4 ($CHCl_3$, c = 0.5).

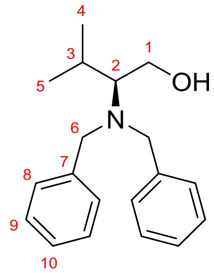

(*S*)-2-(dibenzylamino)-3-methylbutan-1-ol: A mixture of L-valinol (2.06 g, 20.0 mmol, 1 equiv.), benzyl bromide (5.24 mL, 44.0 mmol, 2.2 equiv.), potassium carbonate (8.30 g, 60.0 mmol, 3 equiv.) and tetrabutylammonium iodide (2.22 g, 6.0 mmol, 0.3 equiv.) in acetonitrile (200 mL) was heated under reflux for 16 h. The solvent was removed under reduced pressure and the residue diluted with EtOAc (300 mL) and water (300 mL). The organic layer was separated and the aqueous layer extracted further with EtOAc (3 × 100 mL). The combined organic extracts were dried ($MgSO_4$) and evaporated under reduced pressure. The residue was then purified by silica gel column chromatography (eluent: 15% EtOAc/hexane) to provide the title compound as a colourless oil (4.75 g, 16.8 mmol, 84%). Data are consistent with a reported example.[11]

^{1}H NMR (600 MHz, $CDCl_3$): δ 7.33 – 7.28 (m, 4 H, H9), 7.26 – 7.22 (m, 6 H, H8 and H10), 3.89 (d, *J* = 13.2 Hz, 2 H, H6a), 3.69 (d, *J* = 13.2 Hz, 2 H, H6b), 3.57 (dd, *J* = 10.7, 4.6 Hz, 1 H, H1a), 3.44 (dd, *J* = 10.7, 9.8 Hz, 1 H, H1b), 2.95 (br s, 1 H, OH), 2.54 (ddd, *J* = 9.8, 7.8, 4.6 Hz, 1 H, H2), 2.12 – 2.01 (m, 1 H, H3), 1.14 (d, *J* = 6.8 Hz, 3 H, H4/H5), 0.89 (d, *J* = 6.7 Hz, 3 H, H4/H5).
^{13}C NMR (150 MHz, $CDCl_3$): δ 139.8 (C7), 129.4 (C8), 128.6 (C9), 127.3 (C10), 64.8 (C2), 59.4 (C1), 54.4 (C6), 27.7 (C3), 22.9 (C4/C5), 20.3 (C4/C5).
FTIR (ν_{max}, cm^{-1}): 3443 (br w), 3064 (w), 3028 (w), 2956 (m), 2871 (m), 1703 (w), 1603 (w), 1494 (m), 1453 (s), 1362 (m), 1322 (w), 1247 (w), 1206 (w), 1137 (w), 1099 (m), 1064 (s), 1028 (s), 1009 (s), 910 (m), 858 (w), 827 (w), 787 (w).
HRMS (ESI): calculated for $C_{19}H_{26}NO$ $[M+H]^+$ 284.2009, found 284.2011.
R_f = 0.28 (15% EtOAc/hexane).
$[\alpha]_D^{27.7}$ = +23.1 ($CHCl_3$, c = 1.0); lit.[11] $[\alpha]_D^{20}$ = +24.5 ($CHCl_3$, c = 0.8).

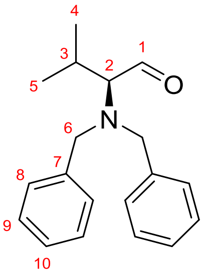

(*S*)-2-(dibenzylamino)-3-methylbutanal: To a solution of oxalyl chloride (1.92 mL, 22.5 mmol, 1.5 equiv.) in anhydrous CH_2Cl_2 (20 mL) at -78 °C was added slowly dropwise a solution of DMSO (2.34 mL, 33.0 mmol, 2.2 equiv.) in anhydrous CH_2Cl_2 (20 mL), keeping the internal temperature below -60 °C. The mixture was stirred further for 5 min. A solution of (*S*)-2-(dibenzylamino)-3-methylbutan-1-ol (4.25 g, 15.0 mmol, 1 equiv.) in anhydrous CH_2Cl_2 (20 mL) was added slowly dropwise and stirred further for 15 min. Triethylamine (10.4 mL, 75.0 mmol, 5 equiv.) was then added, stirred at -78 °C for 30 min, warmed to r.t. and quenched with water (75 mL). The organic layer was separated and the aqueous layer extracted with CH_2Cl_2 (3 × 25 mL). The combined organic extracts were washed with saturated aqueous NH_4Cl solution (50 mL), dried ($MgSO_4$) and evaporated under reduced pressure. The residue was purified by silica gel column chromatography (eluent: 5% EtOAc/hexane) to provide the title compound as a pale yellow turbid oil (3.71 g, 13.2 mmol, 88%). Data are consistent with a reported example.[12]

^{1}H NMR (600 MHz, $CDCl_3$): δ 9.86 (d, *J* = 3.4 Hz, 1 H, H1), 7.37 (d, *J* = 7.3 Hz, 4 H, H8), 7.33 (t, *J* = 7.3 Hz, 4 H, H9), 7.26 (t, *J* = 7.3 Hz, 2 H, H10), 4.03 (d, *J* = 13.8 Hz, 2 H, H6a), 3.72 (d, *J* = 13.8 Hz, 2 H, H6b), 2.73 (dd, *J* = 10.0, 3.4 Hz, 1 H, H2), 2.29 (d sept, *J* = 10.0, 6.7 Hz, 1 H, H3), 1.09 (d, *J* = 6.7 Hz, 3 H, H4/H5), 0.88 (d, *J* = 6.7 Hz, 3 H, H4/H5).
^{13}C NMR (150 MHz, $CDCl_3$): δ 205.3 (C1), 139.4 (C7), 128.9 (C8), 128.5 (C9), 127.3 (C10), 71.7 (C2), 54.7 (C6), 26.2 (C3), 20.3 (C4/C5), 20.0 (C4/C5).
FTIR (ν_{max}, cm^{-1}): 3063 (w), 3030 (w), 2960 (w), 2809 (w), 2716 (w), 1716 (s), 1602 (w), 1494 (m), 1454 (m), 1368 (w), 1308 (w), 1241 (w), 1208 (w), 1155 (w), 1112 (w), 1091 (w), 1074 (m), 1049 (w), 1028 (m), 971 (w), 908 (w), 846 (w), 824 (w).
HRMS (ESI): calculated for $C_{19}H_{24}NO$ $[M+H]^+$ 282.1852, found 282.1850.
R_f = 0.36 (5% EtOAc/hexane).
$[\alpha]_D^{23.7}$ = -63.4 ($CHCl_3$, c = 1.0); lit.[12] $[\alpha]_D^{25}$ = -62.2 ($CHCl_3$, c = 1.0).

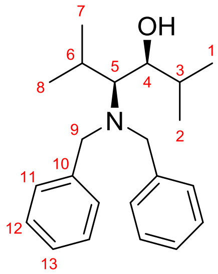

(3*S*,4*S*)-4-(dibenzylamino)-2,5-dimethylhexan-3-ol: To a solution of (*S*)-2-(dibenzylamino)-3-methylbutanal (2.81 g, 10.0 mmol, 1 equiv.) in anhydrous toluene (40 mL) was added a solution of diisopropylzinc (30 mL, 1.0 M in PhMe, 30.0 mmol, 3 equiv.) at 0 °C and the reaction mixture stirred further for 16 h. The reaction was quenched with saturated aqueous NH_4Cl solution (50 mL) and extracted with CH_2Cl_2 (3 × 50 mL). The combined organic extracts were dried ($MgSO_4$) and evaporated under reduced pressure. The residue was purified by silica gel column chromatography (eluent: 5% EtOAc/hexane) to provide the title compound as a yellow oil (3.08 g, 9.46 mmol, 95%).

^{1}H NMR (600 MHz, $CDCl_3$): δ 7.32 – 7.29 (m, 4 H, H12), 7.27 (d, *J* = 7.0 Hz, 4 H, H11), 7.26 – 7.21 (m, 2 H, H13), 4.60 (br s, 1 H, OH), 3.93 (d, *J* = 13.0 Hz, 2 H, H9a), 3.66 (dd, *J* = 9.9, 1.5 Hz, 1 H, H4), 3.42 (d, *J* = 13.0 Hz, 2 H, H9b), 2.51 (dd, *J* = 9.9, 2.2 Hz, 1 H, H5), 2.24 (sept d, *J* = 7.4, 2.2 Hz, 1 H, H6), 1.60 (sept d, *J* = 6.7, 1.5 Hz, 1 H, H3), 1.04 (m, 9 H, H1/H2, H7 and H8), 0.44 (d, *J* = 6.7 Hz, 3 H, H1/H2).
^{13}C NMR (150 MHz, $CDCl_3$): δ 139.0 (C10), 129.4 (C11), 128.5 (C12), 127.3 (C13), 70.5 (C4), 61.9 (C5), 53.8 (C9), 30.0 (C3), 25.0 (C6), 23.9 (C1/C2/C7/C8), 21.7 (C1/C2/C7/C8), 19.3 (C1/C2/C7/C8), 13.5 (C1/C2).
FTIR (ν_{max}, cm^{-1}): 3354 (br w), 3064 (w), 3029 (w), 2959 (s), 2874 (m), 1604 (w), 1495 (m), 1454 (s), 1414 (w), 1389 (m), 1362 (m), 1241 (w), 1208 (w), 1179 (w), 1137 (w), 1095 (m), 1072 (s), 1028 (m), 1005 (s), 972 (s), 947 (m), 911 (w), 867 (w), 847 (w), 810 (w).
HRMS (ESI): calculated for $C_{22}H_{32}NO$ $[M+H]^+$ 326.2478, found 326.2483.
$\boldsymbol{R_f}$ = 0.28 (5% EtOAc/hexane).
$[\alpha]_D^{26.0}$ = +21.0 ($CHCl_3$, c = 1.0).

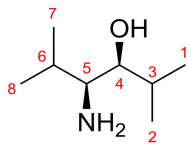

(3*S*,4*S*)-4-amino-2,5-dimethylhexan-3-ol: A mixture of (3*S*,4*S*)-4-(dibenzylamino)-2,5-dimethylhexan-3-ol (1.63 g, 5.0 mmol, 1 equiv.), 20% palladium(II) hydroxide on carbon (0.5 g) and acetic acid (0.34 mL, 6.0 mmol, 1.2 equiv.) in ethanol (50 mL) was stirred under H_2 (balloon) at 50 °C for 16 h. A further portion of 20% palladium(II) hydroxide on carbon (0.5 g) was added, then stirred under H_2 (balloon) at 50 °C for a further 16 h. The mixture was filtered through a pad of Celite, eluting with ethanol, and the solvent removed under reduced pressure. The residue was dissolved in CH_2Cl_2 (25 mL) and 1 M aqueous NaOH solution (25 mL) was added. The organic layer was separated and the aqueous layer extracted further with CH_2Cl_2 (3 × 25 mL). The combined organic extracts were dried ($MgSO_4$) and evaporated under reduced pressure to provide the title product as a white amorphous solid (0.486 g, 3.3 mmol, 67%), m.p. 55-57 °C.

^{1}H NMR (600 MHz, $CDCl_3$): δ 3.15 (t, *J* = 5.3 Hz, 1 H, H4), 2.52 (t, *J* = 5.3 Hz, 1 H, H5), 1.81 (br s, 3 H, NH_2 and OH), 1.76 (sept d, *J* = 6.8, 5.3 Hz, 1 H, H6), 1.69 (sept d, *J* = 6.8, 5.3 Hz, 1 H, H3), 0.95 (d, *J* = 6.8 Hz, 3 H, H1/H2), 0.94 (d, *J* = 6.8 Hz, 3 H, H7/H8), 0.91 (d, *J* = 6.8 Hz, 3 H, H1/H2), 0.86 (d, *J* = 6.8 Hz, 3 H, H7/H8).
^{13}C NMR (150 MHz, $CDCl_3$): δ 75.7 (C4), 57.7 (C5), 30.8 (C3), 30.0 (C6), 20.4 (C7/C8), 20.0 (C1/C2), 16.91 (C1/C2/C7/C8), 16.86 (C1/C2/C7/C8).
FTIR (ν_{max}, cm^{-1}): 3348 (w), 3300 (w), 3165 (br w), 2962 (m), 2932 (m), 2910 (m), 2874 (m), 1619 (m), 1585 (w), 1470 (m), 1389 (w), 1361 (w), 1338 (w), 1315 (w), 1299 (w), 1252 (w), 1189 (w), 1134 (m), 1113 (w), 1057 (m), 1021 (w), 1001 (s), 960 (m), 947 (m), 926 (m), 903 (s), 806 (m), 766 (m).
HRMS (ESI): calculated for $C_8H_{20}NO$ $[M+H]^+$ 146.1539, found 146.1534.
$[\alpha]_D^{25.2}$ = +6.6 ($CHCl_3$, c = 1.0).

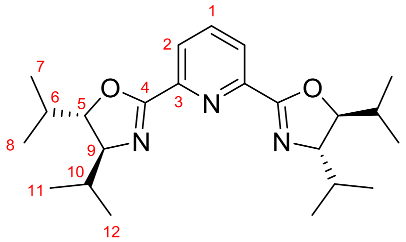

2,6-bis((4*S*,5*S*)-4,5-diisopropyl-4,5-dihydrooxazol-2-yl)pyridine (L3): Following the general procedure for PyBOX synthesis, using 2,6-pyridinedicarbonitrile (0.129 g, 1.0 mmol, 1 equiv.) and (3*S*,4*S*)-4-amino-2,5-dimethylhexan-3-ol (0.290 g, 2.0 mmol, 2 equiv.), purified by silica gel column chromatography (eluent: 3% MeOH/CH_2Cl_2) provided the title compound as a yellow gum (0.065 g, 0.17 mmol, 17%).

^{1}H NMR (600 MHz, $CDCl_3$): δ 8.06 (d, J = 7.8 Hz, 2 H, H2), 7.83 (t, J = 7.8 Hz, 1 H, H1), 4.24 (t, J = 5.9 Hz, 2 H, H5), 3.82 (t, J = 5.9 Hz, 2 H, H9), 1.89 – 1.76 (m, 4 H, H6 and H10), 0.99 – 0.94 (m, 24 H, H7, H8, H11 and H12).
^{13}C NMR (150 MHz, $CDCl_3$): δ 161.7 (C4), 147.2 (C3), 137.3 (C1), 125.5 (C2), 87.9 (C5), 74.9 (C9), 32.9 (C6/C10), 32.7 (C6/C10), 18.8 (C7/C8/C11/C12), 18.4 (C7/C8/C11/C12), 17.9 (C7/C8/C11/C12), 17.3 (C7/C8/C11/C12).
FTIR (ν_{max}, cm^{-1}): 2961 (s), 2933 (m), 2875 (m), 1666 (m), 1575 (m), 1523 (w), 1465 (s), 1386 (m), 1370 (m), 1261 (m), 1239 (m), 1170 (m), 1108 (m), 1079 (m), 979 (s), 955 (m), 929 (w), 904 (w), 832 (w), 763 (w).
HRMS (ESI): calculated for $C_{23}H_{36}N_3O_2$ $[M+H]^+$ 386.2802, found 386.2803.
R_f = 0.34 (3% MeOH/CH_2Cl_2).
$[\alpha]_D^{28.4}$ = -93.2 ($CHCl_3$, c = 1.0).

2.2. PyBIM ligands

General procedure for PyBIM synthesis:[13] To a mixture of the appropriate amide precursor (5.0 mmol, 1 equiv.) in anhydrous $CHCl_3$ (35 mL) was added thionyl chloride (2.1 mL, 28.8 mmol, 5.8 equiv.) at 0 °C. The mixture was then heated under reflux for 5 h. Phosphorus pentachloride (2.19 g, 10.5 mmol, 2.1 equiv.) was then added in one portion and the mixture stirred under reflux for 16 h. The solution was then stripped of volatiles and the residue redissolved in anhydrous $CHCl_3$ (35 mL) to use as a stock solution of the imidoyl chloride intermediate.

(N.B. The imidoyl chlorides are highly water sensitive, reacting with adventitious water from the atmosphere to form the corresponding amides – it is recommended to use the solution immediately. The quality of the solution can be checked by taking a small aliquot, ca. 0.02 mL, removing all solvent under high vacuum and analysing by ^{1}H NMR.)

To a 5 mL microwave vial was added a solution of the imidoyl chloride in $CHCl_3$ (3.5 mL, 0.5 mmol, 1 equiv.), triethylamine (0.52 mL, 3.6 mmol, 7.2 equiv.) and the appropriate aniline/amine (1.1 mmol, 2.2 equiv.). The vial was sealed and stirred at r.t. or 60 °C for the allotted time. The mixture was diluted with CH_2Cl_2 (10 mL) then quenched with 1 M aqueous NaOH solution (10 mL). The organic layer was separated and the aqueous layer extracted further with CH_2Cl_2 (3 × 10 mL). The combined organic extracts were dried ($MgSO_4$) and evaporated under reduced pressure. The residue was then purified by silica gel column chromatography.

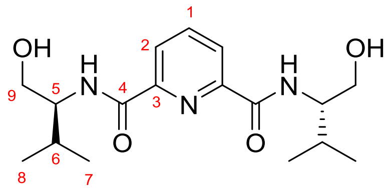

***N*^{2},*N*^{6}-bis((*S*)-1-hydroxy-3-methylbutan-2-yl)pyridine-2,6-dicarboxamide:** To a stirred solution of L-valinol (2.06 g, 20.0 mmol, 2 equiv.) and triethylamine (2.79 mL, 20.0 mmol, 2 equiv.) in anhydrous CH_2Cl_2 (50 mL) was added portionwise pyridine-2,6-dicarbonyl chloride (2.04 g, 10.0 mmol, 1 equiv.) at 0 °C. The mixture was warmed to r.t. and stirred further for 16 h. Water (50 mL) was added and the organic layer separated. The aqueous layer was extracted further with CH_2Cl_2 (3 × 25 mL) and the combined organic extracts dried ($MgSO_4$) and evaporated under reduced pressure. The residue was recrystallised from CH_2Cl_2 to provide the title compound as a white crystalline solid (2.20 g, 6.5 mmol, 65%), m.p. 120-121 °C (lit. m.p.[14] 118-120 °C). Data are consistent with a reported example.[15]

^{1}H NMR (600 MHz, $CDCl_3$): δ 8.36 (d, *J* = 7.8 Hz, 2 H, H2), 8.06 (t, *J* = 7.8 Hz, 1 H, H1), 8.00 (br d, *J* = 8.1 Hz, 2 H, NH), 3.99 – 3.93 (m, 2 H, H5), 3.89 – 3.81 (m, 4 H, H9), 2.53 (br s, 2 H, OH), 2.09 (oct, *J* = 6.8 Hz, 2 H, H6), 1.06 (d, *J* = 6.8 Hz, 6 H, H7/H8), 1.04 (d, *J* = 6.8 Hz, 6 H, H7/H8).
^{13}C NMR (150 MHz, $CDCl_3$): δ 164.2 (C4), 148.8 (C3), 139.4 (C1), 125.3 (C2), 64.2 (C9), 57.4 (C5), 29.5 (C6), 19.8 (C7/C8), 18.7 (C7/C8).
FTIR (ν_{max}, cm^{-1}): 3401 (w), 3304 (br m), 2964 (w), 2878 (w), 1680 (m), 1644 (s), 1592 (w), 1538 (s), 1477 (m), 1442 (w), 1389 (w), 1368 (w), 1341 (w), 1302 (w), 1238 (w), 1171 (w), 1145 (w), 1094 (w), 1066 (s), 1017 (s), 1000 (w), 970 (w), 956 (w), 934 (w), 899 (w), 872 (w), 844 (m), 800 (w).
HRMS (ESI): calculated for $C_{17}H_{27}N_3O_4Na$ $[M+Na]^+$ 360.1894, found 360.1892.

$[\alpha]_D^{25.9}$ = -37.5 ($CHCl_3$, c = 1.0); lit.[16] $[\alpha]_D^{25}$ = -82.3 (acetone, c = 0.15).

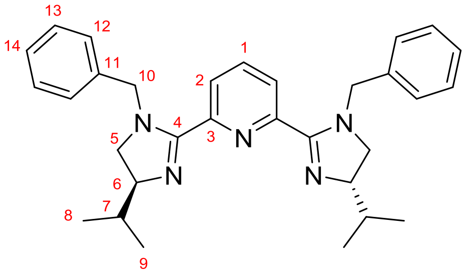

2,6-bis((*S*)-1-benzyl-4-isopropyl-4,5-dihydro-1*H*-imidazol-2-yl)pyridine: Following the general procedure for PyBIM synthesis, using N^2,N^6-bis((*S*)-1-hydroxy-3-methylbutan-2-yl)pyridine-2,6-dicarboxamide and benzylamine (0.12 mL, 1.1 mmol, 2.2 equiv.) at 60 °C for 16 h, purified by silica gel column chromatography (eluent: 10% MeOH/CH_2Cl_2) provided the title compound as a brown foam (113.1 mg, 0.236 mmol, 47%). Compound has been prepared previously,[13] but NMR spectra were recorded in CD_3OD.

^{1}H NMR (600 MHz, $CDCl_3$): δ 8.21 (d, *J* = 7.9 Hz, 2 H, H2), 7.95 (t, *J* = 7.9 Hz, 1 H, H1), 7.28 – 7.23 (m, 6 H, H13 and H14), 7.14 – 7.09 (m, 4 H, H12), 4.57 (AB q, *J* = 15.3 Hz, 4 H, H10a and H10b), 3.96 (ddd, *J* = 10.7, 9.6, 6.7 Hz, 2 H, H6), 3.49 (dd, *J* = 10.7, 9.6 Hz, 2 H, H5a), 3.09 (t, *J* = 9.6 Hz, 2 H, H5b), 1.86 (oct, *J* = 6.7 Hz, 2 H, H7), 0.94 (d, *J* = 6.7 Hz, 6 H, H8/H9), 0.84 (d, *J* = 6.7 Hz, 6 H, H8/H9).
^{13}C NMR (150 MHz, $CDCl_3$): δ 162.2 (C4), 148.3 (C3), 138.3 (C1), 137.1 (C11), 128.6 (C13), 127.6 (C12), 127.5 (C14), 127.1 (C2), 68.3 (C6), 53.0 (C5), 51.7 (C10), 32.9 (C7), 18.8 (C8/C9), 17.8 (C8/C9).
FTIR (ν_{max}, cm^{-1}): 3030 (w), 2957 (m), 2871 (m), 1596 (s), 1563 (s), 1495 (m), 1454 (m), 1385 (w), 1363 (m), 1312 (w), 1263 (m), 1151 (w), 1110 (w), 1077 (w), 1029 (w), 995 (w), 925 (w), 831 (m).
HRMS (ESI): calculated for $C_{31}H_{38}N_5$ $[M+H]^+$ 480.3122, found 480.3138.
R_f = 0.07 (10% MeOH/CH_2Cl_2).
$[\alpha]_D^{27.8}$ = -130.6 ($CHCl_3$, c = 1.0); lit.[13] $[\alpha]_D^{25}$ = -133.5 ($CHCl_3$, c = 0.39).

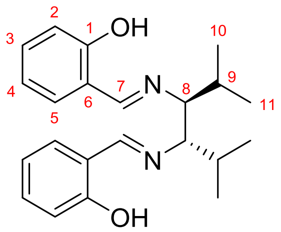

2,2'-((1*E*,1'*E*)-(((3*S*,4*S*)-2,5-dimethylhexane-3,4-diyl)bis(azanylylidene))bis(methanyl-ylidene))diphenol:[17] To a solution of (*R*,*R*)-1,2-bis(2-hydroxyphenyl)-1,2-diaminoethane (6.92 g, 28.3 mmol, 1 equiv.) in anhydrous toluene (75 mL) was added isobutyraldehyde (6.5 mL, 70.8 mmol, 2.5 equiv.). The mixture was heated under reflux with equipped Dean-Stark apparatus for 16 h. The mixture was then evaporated under reduced pressure and the residue triturated with MeOH. The product was collected by filtration to provide the title product as a yellow powder (8.49 g, 24.1 mmol, 85%), m.p. 189-191 °C (lit. m.p.[17] 188-189 °C). Data are consistent with a reported example.[17]

^{1}H NMR (600 MHz, $CDCl_3$): δ 13.54 (s, 2 H, OH), 8.17 (s, 2 H, H7), 7.28 – 7.24 (m, 2 H, H3), 7.15 (dd, *J* = 7.5, 1.7 Hz, 2 H, H5), 6.96 – 6.93 (m, 2 H, H2), 6.79 (td, *J* = 7.5, 1.1 Hz,

2 H, H4), 3.26 – 3.19 (m, 2 H, H8), 2.17 – 2.09 (m, 2 H, H9), 0.99 (d, *J* = 6.8 Hz, 6 H, H10/H11), 0.91 (d, *J* = 6.8 Hz, 6 H, H10/H11).
^{13}C NMR (150 MHz, $CDCl_3$): δ 165.8 (C7), 161.4 (C1), 132.3 (C3), 131.6 (C5), 118.57 (C4), 118.56 (C6), 117.1 (C2), 76.3 (C8), 28.6 (C9), 20.7 (C10/C11), 17.5 (C10/C11).
FTIR (ν_{max}, cm^{-1}): 2966 (w), 2927 (w), 2873 (w), 1627 (s), 1579 (m), 1499 (m), 1460 (m), 1418 (w), 1389 (w), 1364 (w), 1337 (w), 1315 (w), 1280 (m), 1207 (w), 1175 (w), 1150 (w), 1115 (w), 1083 (w), 1032 (w), 999 (w), 988 (w), 959 (w), 922 (w), 889 (w), 850 (w), 826 (w).
HRMS (ESI): calculated for $C_{22}H_{29}N_2O_2$ [M-H]$^-$ 351.2078, found 351.2077.
$[\alpha]_D^{28.4}$ = +150.0 ($CHCl_3$, c = 1.0); lit.[17] $[\alpha]_D^{26}$ = +144.8 ($CHCl_3$, c = 1.0).

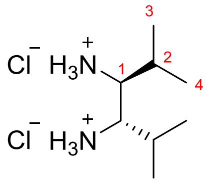

(3*S*,4*S*)-2,5-dimethylhexane-3,4-diammonium dichloride: To a solution of 2,2'-((1*E*,1'*E*)-(((3*S*,4*S*)-2,5-dimethylhexane-3,4-diyl)bis(azanylylidene))bis(methanylylidene))diphenol in THF (200 mL) was added 37% aqueous HCl (7.2 mL). The mixture was heated at 50 °C for 3 h, producing a white precipitate. The product was collected by filtration, providing the title product as a white crystalline solid (4.68 g, 21.5 mmol, 89%), m.p. 284-286 °C (sublim.) (lit. m.p.[17] >200 °C (sublim.)). Data are consistent with a reported example.[17]

^{1}H NMR (600 MHz, d_6-DMSO): δ 8.48 (br s, 6 H, NH), 3.21 (d, *J* = 5.4 Hz, 2 H, H1), 2.17 – 2.07 (m, 2 H, H2), 1.03 – 0.94 (m, 12 H, H3 and H4).
^{13}C NMR (150 MHz, d_6-DMSO): δ 56.2 (C1), 27.2 (C2), 19.7 (C3/C4), 17.3 (C3/C4).
FTIR (ν_{max}, cm^{-1}): 2824 (br m), 1588 (s), 1555 (m), 1532 (m), 1497 (s), 1458 (m), 1379 (w), 1035 (w), 983 (w).
HRMS (ESI): calculated for $C_8H_{21}N_2$ [of free base, M+H]$^+$ 145.1699, found 145.1696.
$[\alpha]_D^{28.4}$ = -4.8 (MeOH, c = 1.0); lit.[17] $[\alpha]_D^{27}$ = +5.4 (MeOH, c = 1.0) (N.B. we believe there is a sign error in the literature optical rotation, given that their other similar (*S*,*S*) diamine hydrochloride salts also have negative optical rotation values).

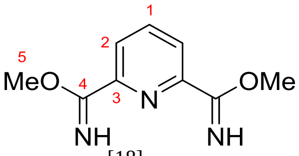

Dimethyl pyridine-2,6-bis(carbimidate):[18] To a solution of 2,6-pyridinedicarbonitrile (2.58 g, 20.0 mmol, 1 equiv.) in anhydrous MeOH (50 mL) was added sodium (60 mg, 2.5 mmol, 0.125 equiv.) and the mixture stirred at r.t. for 48 h. Acetic acid (0.15 mL, 2.5 mmol, 0.125 equiv.) was then added and the solvent removed under reduced pressure, which provided the title compound as a yellow amorphous solid (3.86 g, 20.0 mmol, 99%), m.p. >200 °C (dec.). Data are consistent with a reported example.[19]

^{1}H NMR (600 MHz, $CDCl_3$): δ 9.23 (br s, 2 H, NH), 7.93 – 7.91 (m, 3 H, H1 and H2), 4.03 (s, 6 H, H5).
^{13}C NMR (150 MHz, $CDCl_3$): δ 166.2 (C4), 147.1 (C3), 139.1 (C1), 122.7 (C2), 54.2 (C5).
FTIR (ν_{max}, cm^{-1}): 3289 (w), 3268 (m), 3008 (w), 2956 (w), 1654 (m), 1640 (m), 1571 (s), 1457 (m), 1434 (m), 1338 (s), 1268 (w), 1210 (m), 1194 (m), 1158 (w), 1080 (s), 998 (m), 946 (s), 914 (s), 874 (w), 824 (s), 751 (s).
HRMS (ESI): calculated for $C_9H_{11}N_3O_2Na$ [M+Na]$^+$ 216.0743, found 216.0749.

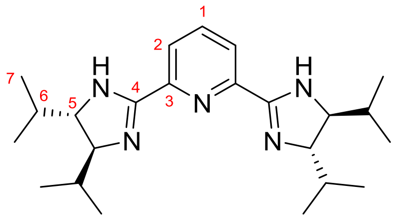

2,6-bis((4*S*,5*S*)-4,5-diisopropyl-4,5-dihydro-1*H*-imidazol-2-yl)pyridine: To a suspension of (3*S*,4*S*)-2,5-dimethylhexane-3,4-diammonium dichloride (1.82 g, 8.4 mmol, 2.1 equiv.) in CH_2Cl_2 (25 mL) was added 3 M aqueous NaOH solution (20 mL). The organic layer was separated and the aqueous layer extracted further with CH_2Cl_2 (3 × 10 mL). The combined organic extracts were dried ($MgSO_4$) and evaporated under reduced pressure to provide the corresponding free base (ca. 1.21 g, 8.4 mmol). The free base was then dissolved in CH_2Cl_2 (20 mL) and transferred into a 20 mL microwave vial charged with dimethyl pyridine-2,6-bis(carbimidate) (0.773 g, 4.0 mmol, 1 equiv.). The mixture was heated at 40 °C for 48 h. Water (20 mL) was then added and the organic layer separated. The aqueous layer was extracted further with CH_2Cl_2 (3 × 20 mL) and the combined organic extracts dried ($MgSO_4$) then evaporated under reduced pressure. The residue was purified by silica gel column chromatography (eluent: 10% MeOH/CH_2Cl_2) to provide the title product as a white foam (0.893 g, 2.33 mmol, 58%).

^{1}H NMR (600 MHz, $CDCl_3$): δ 8.25 (d, *J* = 7.8 Hz, 2 H, H2), 7.80 (t, *J* = 7.8 Hz, 1 H, H1), 5.90 (br s, 2 H, NH), 3.64 (br s, 4 H, H5), 1.75 (br s, 4 H, H6), 0.95 (d, *J* = 6.8 Hz, 24 H, H7).
^{13}C NMR (150 MHz, $CDCl_3$): δ 160.5 (C4), 147.8 (br, C3), 137.1 (C1), 124.1 (C2), 33.4 (C6), 18.5 (C7). (C5 too broadened to distinguish properly).
FTIR (ν_{max}, cm^{-1}): 3204 (br w), 2957 (m), 2871 (m), 1603 (m), 1565 (m), 1463 (s), 1385 (m), 1365 (m), 1320 (w), 1261 (m), 1240 (m), 1148 (w), 1077 (w), 992 (m), 938 (w), 832 (m).
HRMS (ESI): calculated for $C_{23}H_{38}N_5$ $[M+H]^+$ 384.3122, found 384.3130.
R_f = 0.15-0.45 (10% MeOH/CH_2Cl_2).
$[\alpha]_D^{28.3}$ = -162.4 ($CHCl_3$, c = 1.0).

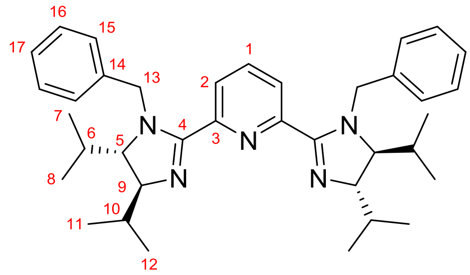

2,6-bis((4*S*,5*S*)-1-benzyl-4,5-diisopropyl-4,5-dihydro-1*H*-imidazol-2-yl)pyridine: To a suspension of NaH (64 mg, 60% dispersion in mineral oil, 1.6 mmol, 4 equiv.) in anhydrous DMF (3 mL) was added a solution of 2,6-bis((4*S*,5*S*)-4,5-diisopropyl-4,5-dihydro-1*H*-imidazol-2-yl)pyridine (0.153 g, 0.4 mmol, 1 equiv.) in anhydrous DMF (1 mL) at 0 °C. The mixture was stirred further for 30 min. A solution of benzyl bromide (0.12 mL, 1.0 mmol, 2.5 equiv.) was added slowly dropwise over 30 min and then the mixture was warmed to r.t. and stirred for 16 h. The mixture was quenched with water (20 mL), extracted with CH_2Cl_2 (3 × 25 mL) and the combined organic extracts evaporated under reduced pressure. The residue was redissolved in Et_2O (25 mL), washed with water (4 × 25 mL), brine (25 mL), dried ($MgSO_4$) and evaporated under reduced pressure. The residue was purified by silica gel

column chromatography (eluent: 10% MeOH/CH_2Cl_2) to provide the title product as a colourless resin (0.140 g, 0.25 mmol, 62%).

^{1}H NMR (600 MHz, $CDCl_3$): δ 8.03 (d, *J* = 7.8 Hz, 2 H, H2), 7.85 (t, *J* = 7.8 Hz, 1 H, H1), 7.24 – 7.17 (m, 6 H, H16 and H17), 7.12 – 7.09 (m, 4 H, H15), 5.11 (d, *J* = 15.4 Hz, 2 H, H13a), 4.24 (d, *J* = 15.4 Hz, 2 H, H13b), 3.56 (t, *J* = 5.2 Hz, 2 H, H9), 3.20 (dd, *J* = 5.2, 3.7 Hz, 2 H, H5), 1.93 – 1.84 (m, 2 H, H6), 1.53 – 1.43 (m, 2 H, H10), 0.83 (d, *J* = 6.9 Hz, 6 H, H7/H8), 0.82 (d, *J* = 6.9 Hz, 6 H, H11/H12), 0.80 (d, *J* = 6.9 Hz, 6 H, H7/H8), 0.77 (d, *J* = 6.9 Hz, 6 H, H11/H12).
^{13}C NMR (150 MHz, $CDCl_3$): δ 161.7 (C4), 149.9 (C3), 138.0 (C14), 137.7 (C1), 128.4 (C15/C16), 128.3 (C15/C16), 127.3 (C17), 126.0 (C2), 70.5 (C9), 66.9 (C5), 49.5 (C13), 33.8 (C10), 29.6 (C6), 18.9 (C11/C12), 17.8 (C11/C12), 17.5 (C7/C8), 16.0 (C7/C8).
FTIR (ν_{max}, cm^{-1}): 2957 (m), 2870 (m), 1594 (m), 1562 (s), 1463 (s), 1399 (w), 1385 (m), 1363 (w), 1327 (w), 1261 (w), 1210 (w), 1147 (m), 1074 (m), 1025 (w), 997 (w), 966 (m), 930 (w), 833 (s).
HRMS (ESI): calculated for $C_{37}H_{50}N_5$ $[M+H]^+$ 564.4061, found 564.4071.
R_f = 0.16 (10% MeOH/CH_2Cl_2).
$[\alpha]_D^{28.4}$ = -172.4 ($CHCl_3$, c = 1.0).

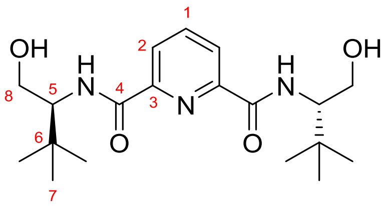

***N*^{2},*N*^{6}-bis((*S*)-1-hydroxy-3,3-dimethylbutan-2-yl)pyridine-2,6-dicarboxamide:** To a stirred solution of L-*tert*-leucinol (9.11 g, 77.7 mmol, 2 equiv.) and triethylamine (10.8 mL, 77.7 mmol, 2 equiv.) in anhydrous CH_2Cl_2 (450 mL) was added portionwise pyridine-2,6-dicarbonyl chloride (7.93 g, 38.8 mmol, 1 equiv.) at 0 °C. The mixture was warmed to r.t. and stirred further for 16 h. Water (450 mL) was added and the organic layer separated. The aqueous layer was extracted further with CH_2Cl_2 (3 × 200 mL) and the combined organic extracts dried ($MgSO_4$) and evaporated under reduced pressure, which provided the title product as a white foam (14.17 g, 38.8 mmol, 99%). Data are consistent with a reported example.[15]

^{1}H NMR (600 MHz, $CDCl_3$): δ 8.30 (d, *J* = 7.7 Hz, 2 H, H2), 8.04 (br d, *J* = 9.0 Hz, 2 H, NH), 8.01 (t, *J* = 7.7 Hz, 1 H, H1), 4.01 – 3.91 (m, 4 H, H5a and H8), 3.79 – 3.69 (m, 2 H, H5b), 2.77 (br s, 2 H, OH), 1.04 (s, 18 H, H7).
^{13}C NMR (150 MHz, $CDCl_3$): δ 164.4 (C4), 148.7 (C3), 139.4 (C1), 125.3 (C2), 63.2 (C5), 60.1 (C8), 34.1 (C6), 27.2 (C7).
FTIR (ν_{max}, cm^{-1}): 3392 (br w), 2960 (m), 1662 (s), 1525 (s), 1476 (m), 1445 (m), 1398 (w), 1367 (m), 1235 (m), 1174 (w), 1079 (m), 1050 (m), 1022 (w), 1000 (m), 933 (w), 843 (w), 754 (m).
HRMS (ESI): calculated for $C_{19}H_{31}N_3O_4Na$ $[M+Na]^+$ 388.2207, found 388.2198.
R_f = 0.18 (EtOAc).
$[\alpha]_D^{27.0}$ = -0.6 ($CHCl_3$, c = 1.0).

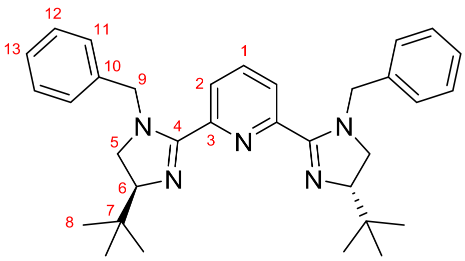

2,6-bis((*S*)-1-benzyl-4-(*tert*-butyl)-4,5-dihydro-1*H*-imidazol-2-yl)pyridine: Following the general procedure for PyBIM synthesis, using N^2,N^6-bis((*S*)-1-hydroxy-3,3-dimethylbutan-2-yl)pyridine-2,6-dicarboxamide and benzylamine (0.12 mL, 1.1 mmol, 2.2 equiv.) at 60 °C for 16 h, purified by silica gel column chromatography (eluent: 10% MeOH/EtOAc) provided the title compound as a brown gum (100.1 mg, 0.197 mmol, 39%).

^{1}H NMR (600 MHz, $CDCl_3$): δ 8.06 (br d, *J* = 7.7 Hz, 2 H, H2), 7.87 (t, *J* = 7.7 Hz, 1 H, H1), 7.29 – 7.23 (m, 6 H, H12 and H13), 7.17 – 7.13 (m, 4 H, H11), 4.60 (d, *J* = 15.2 Hz, 2 H, H9a), 4.53 (br d, *J* = 15.2 Hz, 2 H, H9b), 3.84 (appears t, *J* = 10.6 Hz, 2 H, H6), 3.40 (t, *J* = 10.6 Hz, 2 H, H5a), 3.07 (appears t, *J* = 9.9 Hz, 2 H, H5b), 0.88 (s, 18 H, H8).
^{13}C NMR (150 MHz, $CDCl_3$): δ 162.7 (C4), 137.9 (C1), 128.6 (C12), 127.9 (C11), 127.4 (C10), 127.3 (C11), 126.3 – 126.0 (br, C2), 73.6 – 73.1 (br, C6), 51.9 (C5/C9), 51.8 (C5/C9), 34.3 (C7), 26.2 (C8). (C3 too broadened to distinguish properly).
FTIR (ν_{max}, cm^{-1}): 3029 (w), 2952 (s), 2866 (m), 1594 (m), 1565 (s), 1495 (m), 1454 (s), 1390 (m), 1362 (s), 1275 (m), 1148 (m), 1076 (m), 1028 (m), 829 (m).
HRMS (ESI): calculated for $C_{33}H_{42}N_5$ $[M+H]^+$ 508.3435, found 508.3423.
R_f = 0.14 (15% MeOH/EtOAc).
$[\alpha]_D^{25.9}$ = -140.4 ($CHCl_3$, c = 1.0).

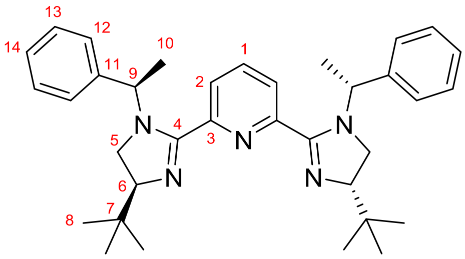

2,6-bis((*S*)-4-(*tert*-butyl)-1-((*R*)-1-phenylethyl)-4,5-dihydro-1*H*-imidazol-2-yl)pyridine: Following the general procedure for PyBIM synthesis, using N^2,N^6-bis((*S*)-1-hydroxy-3,3-dimethylbutan-2-yl)pyridine-2,6-dicarboxamide and (*R*)-1-phenylethan-1-amine (0.14 mL, 1.1 mmol, 2.2 equiv.) at 60 °C for 16 h, purified by silica gel column chromatography (eluent: 5% MeOH/CH_2Cl_2) provided the title compound as a brown gum (26.3 mg, 0.049 mmol, 10%).

^{1}H NMR (600 MHz, $CDCl_3$): δ 8.26 (br s, 2 H, H2), 8.05 (t, *J* = 7.7 Hz, 1 H, H1), 7.32 – 7.25 (m, 10 H, H12, H13 and H14), 5.67 (br s, 2 H, H9), 3.86 (t, *J* = 10.7 Hz, 2 H, H6), 3.29 (appears br t, *J* = 9.1 Hz, 2 H, H5a), 3.18 (appears t, *J* = 10.7 Hz, 2 H, H5b), 1.42 (d, *J* = 6.8 Hz, 6 H, H10), 0.97 (s, 18 H, H8).
^{13}C NMR (150 MHz, $CDCl_3$): δ 162.4 (C4), 138.9 (C1), 128.8 (C12/C13), 128.1 (br, C2), 127.8 (C14), 127.1 (C12/C13), 53.2 (C9), 46.1 (C5), 34.6 (C7), 25.9 (C8), 16.3 (C10). (C11 appears to be hidden behind other aromatic carbon peaks; C3, C6 too broadened to distinguish properly).
FTIR (ν_{max}, cm^{-1}): 2953 (m), 2866 (w), 1592 (m), 1562 (s), 1454 (m), 1391 (w), 1362 (m), 1279 (s), 1205 (m), 1174 (m), 1078 (w), 1024 (m), 994 (w), 827 (m), 787 (m).

HRMS (ESI): calculated for $C_{35}H_{46}N_5$ $[M+H]^+$ 536.3748, found 536.3737.
$\boldsymbol{R_f}$ = 0.06 (5% MeOH/CH_2Cl_2).
$[\alpha]_D^{26.1}$ = +37.2 ($CHCl_3$, c = 1.0).

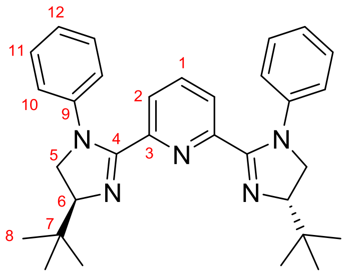

2,6-bis((*S*)-4-(*tert*-butyl)-1-phenyl-4,5-dihydro-1*H*-imidazol-2-yl)pyridine (L4): Following the general procedure for PyBIM synthesis, using N^2,N^6-bis((*S*)-1-hydroxy-3,3-dimethylbutan-2-yl)pyridine-2,6-dicarboxamide and aniline (0.10 mL, 1.1 mmol, 2.2 equiv.) at 60 °C for 16 h, purified by silica gel column chromatography (eluent: 5% MeOH/CH_2Cl_2) provided the title compound as a pale brown gum (213.0 mg, 0.444 mmol, 89%).

^{1}H NMR (600 MHz, $CDCl_3$): δ 7.67 – 7.60 (m, 3 H, H1 and H2), 7.09 (t, *J* = 7.6 Hz, 4 H, H11), 6.92 (t, *J* = 7.6 Hz, 2 H, H12), 6.60 (d, *J* = 7.6 Hz, 4 H, H10), 4.02 (dd, *J* = 10.9, 9.3 Hz, 2 H, H5a), 3.93 (dd, *J* = 10.9, 7.6 Hz, 2 H, H6), 3.60 (dd, *J* = 9.3, 7.6 Hz, 2 H, H5b), 0.91 (s, 18 H, H8).
^{13}C NMR (150 MHz, $CDCl_3$): δ 159.8 (C4), 149.9 (C3), 143.1 (C9), 136.7 (C1), 128.4 (C11), 124.6 (C2), 123.3 (C12), 122.8 (C10), 74.1 (C6), 55.1 (C5), 34.3 (C7), 26.0 (C8).
FTIR (ν_{max}, cm^{-1}): 2954 (w), 2868 (w), 1595 (m), 1569 (m), 1496 (s), 1480 (s), 1425 (w), 1392 (m), 1362 (m), 1299 (w), 1280 (w), 1216 (w), 1168 (w), 1082 (w), 1049 (w), 1018 (w), 994 (w), 931 (w), 825 (w).
HRMS (ESI): calculated for $C_{31}H_{38}N_5$ $[M+H]^+$ 480.3122, found 480.3109.
$\boldsymbol{R_f}$ = 0.11 (5% MeOH/CH_2Cl_2).
$[\alpha]_D^{25.9}$ = +35.3 ($CHCl_3$, c = 1.0).

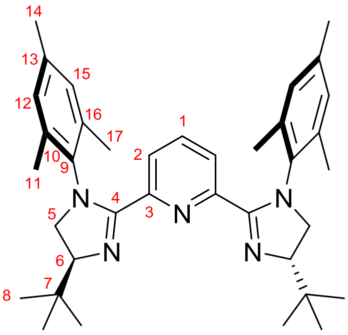

2,6-bis((*S*)-4-(*tert*-butyl)-1-mesityl-4,5-dihydro-1*H*-imidazol-2-yl)pyridine: Following the general procedure for PyBIM synthesis, using N^2,N^6-bis((*S*)-1-hydroxy-3,3-dimethylbutan-2-yl)pyridine-2,6-dicarboxamide and 2,4,6-trimethylaniline (0.16 mL, 1.1 mmol, 2.2 equiv.) at r.t. for 48 h, purified by silica gel column chromatography (eluent: 10% MeOH/CH_2Cl_2) provided the title compound as a pale brown gum (68.9 mg, 0.122 mmol, 24%).

^{1}H NMR (600 MHz, $CDCl_3$): δ 7.58 (br t, *J* = 7.9 Hz, 1 H, H1), 7.51 (d, *J* = 7.9 Hz, 2 H, H2), 6.82 (s, 2 H, H12/H15), 6.71 (s, 2 H, H12/H15), 4.21 (appears t, *J* = 11.3 Hz, 2 H, H6), 3.93 (t, *J* = 11.3 Hz, 2 H, H5a), 3.57 (appears t, *J* = 10.8 Hz, 2 H, H5b), 2.24 (s, 6 H, H11/H17), 2.18 (s, 6 H, H14), 1.82 (s, 6 H, H11/H17), 1.05 (s, 18 H, H8).

^{13}C NMR (150 MHz, $CDCl_3$): δ 162.5 (C4), 146.0 (C3), 138.3 (C9), 137.6 (C1), 136.1 (C10/C16), 134.8 (C10/C16), 134.1 (C13), 130.0 (C12/C15), 129.9 (C12/C15), 126.4 (C2), 70.7 (C6), 53.8 (C5), 34.3 (C7), 26.2 (C8), 20.9 (C14), 18.2 (C11/C17), 17.9 (C11/C17).
FTIR (ν_{max}, cm^{-1}): 2952 (s), 2867 (m), 1612 (m), 1558 (s), 1479 (s), 1362 (w), 1280 (m), 1172 (w), 1018 (w), 853 (w), 823 (w).
HRMS (ESI): calculated for $C_{37}H_{50}N_5$ $[M+H]^+$ 564.4061, found 564.4053.
$\boldsymbol{R_f}$ = 0.11 (10% MeOH/CH_2Cl_2).
$[\alpha]_D^{28.4}$ = +19.6 ($CHCl_3$, c = 1.0).

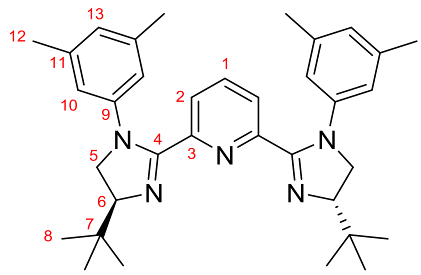

2,6-bis((*S*)-4-(*tert*-butyl)-1-(3,5-dimethylphenyl)-4,5-dihydro-1*H*-imidazol-2-yl)pyridine (L5): Following the general procedure for PyBIM synthesis, using *N*^{2},*N*^{6}-bis((*S*)-1-hydroxy-3,3-dimethylbutan-2-yl)pyridine-2,6-dicarboxamide and 3,5-dimethylaniline (0.16 mL, 1.1 mmol, 2.2 equiv.) at r.t. for 16 h, purified by silica gel column chromatography (eluent: 10% MeOH/CH_2Cl_2) provided the title compound as an orange resin (206.3 mg, 0.385 mmol, 77%).

^{1}H NMR (600 MHz, $CDCl_3$): δ 7.93 (br d, J = 7.7 Hz, 2 H, H2), 7.77 (br t, J = 7.7 Hz, 1 H, H1), 6.71 (s, 2 H, H13), 6.34 (s, 4 H, H10), 4.12 (t, J = 10.5 Hz, 2 H, H5a), 4.04 (dd, J = 10.5, 7.9 Hz, 2 H, H6), 3.76 (dd, J = 10.5, 7.9 Hz, 2 H, H5b), 2.16 (s, 12 H, H12), 0.94 (s, 18 H, H8).
^{13}C NMR (150 MHz, $CDCl_3$): δ 160.0 (C4), 146.2 – 145.8 (br, C3), 140.3 – 140.1 (C9), 138.7 (C11), 137.8 – 137.7 (br, C1), 127.7 – 127.5 (br, C2/C13), 127.5 – 127.3 (br, C2/C13), 121.3 (C10), 70.4 – 70.0 (br, C6), 55.7 (C5), 34.4 (C7), 25.6 (C8), 21.3 (C12).
FTIR (ν_{max}, cm^{-1}): 2953 (m), 1595 (s), 1567 (s), 1476 (s), 1363 (w), 1337 (w), 1275 (w), 1217 (w), 994 (w), 834 (w).
HRMS (ESI): calculated for $C_{35}H_{46}N_5$ $[M+H]^+$ 536.3748, found 536.3735.
$\boldsymbol{R_f}$ = 0.05 (10% MeOH/CH_2Cl_2).
$[\alpha]_D^{28.4}$ = -18.6 ($CHCl_3$, c = 1.0).

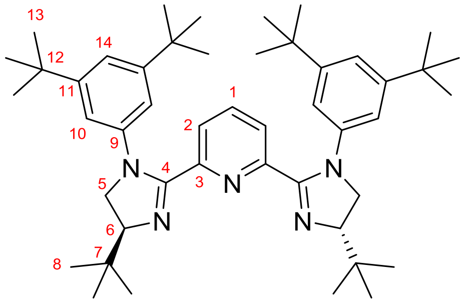

2,6-bis((*S*)-4-(*tert*-butyl)-1-(3,5-di-*tert*-butylphenyl)-4,5-dihydro-1*H*-imidazol-2-yl)pyridine (L6): Following the general procedure for PyBIM synthesis, using *N*^{2},*N*^{6}-bis((*S*)-1-hydroxy-3,3-dimethylbutan-2-yl)pyridine-2,6-dicarboxamide and 3,5-di-*tert*-butylaniline (0.226 g, 1.1 mmol, 2.2 equiv.) at r.t. for 16 h, purified by silica gel column chromatography

(eluent: 10% MeOH/CH_2Cl_2) provided the title compound as a yellow foam (201.3 mg, 0.286 mmol, 57%).

^{1}H NMR (600 MHz, d$_4$-MeOD): δ 7.80 (t, *J* = 8.0 Hz, 1 H, H1), 7.41 (d, *J* = 8.0 Hz, 2 H, H2), 7.36 (t, *J* = 1.5 Hz, 2 H, H14), 6.94 (d, *J* = 1.5 Hz, 4 H, H10), 4.39 (t, *J* = 10.7 Hz, 2 H, H5a), 4.21 (dd, *J* = 10.7, 8.1 Hz, 2 H, H6), 4.16 (dd, *J* = 10.7, 8.1 Hz, 2 H, H5b), 1.24 (s, 36 H, H13), 1.06 (s, 18 H, H8).
^{13}C NMR (150 MHz, d$_4$-MeOD): δ 162.2 (C4), 153.9 (C9), 147.3 (C3), 140.2 (C11), 139.1 (C1), 128.4 (C2), 122.3 (C14), 120.0 (C10), 70.9 (C6), 57.4 (C5), 35.9 (C12), 35.3 (C7), 31.7 (C13), 25.8 (C8).
FTIR (ν_{max}, cm^{-1}): 2954 (s), 2868 (m), 1594 (s), 1567 (m), 1479 (m), 1393 (w), 1362 (m), 1334 (w), 1248 (m), 1203 (w), 1168 (w), 1084 (w), 993 (w), 900 (w), 864 (w), 827 (w), 751 (m).
HRMS (ESI): calculated for $C_{47}H_{70}N_5$ $[M+H]^+$ 704.5626, found 704.5618.
R_f = 0.14 (10% MeOH/CH_2Cl_2).
$[\alpha]_D^{28.4}$ = -70.6 ($CHCl_3$, c = 1.0).

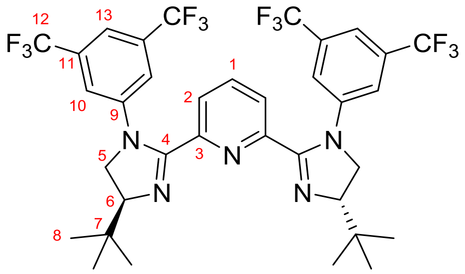

2,6-bis((*S*)-1-(3,5-bis(trifluoromethyl)phenyl)-4-(*tert*-butyl)-4,5-dihydro-1*H*-imidazol-2-yl)pyridine (L7): Following the general procedure for PyBIM synthesis, using *N*^{2},*N*^{6}-bis((*S*)-1-hydroxy-3,3-dimethylbutan-2-yl)pyridine-2,6-dicarboxamide and 3,5-bis(trifluoromethyl)-aniline (0.10 mL, 0.66 mmol, 2.2 equiv.) at 60 °C for 16 h, purified by silica gel column chromatography (eluent: 3% MeOH/CH_2Cl_2) provided the title compound as a light brown foam (163.0 mg, 0.212 mmol, 43%).

^{1}H NMR (600 MHz, $CDCl_3$): δ 7.99 (d, *J* = 7.8 Hz, 2 H, H2), 7.85 (t, *J* = 7.8 Hz, 1 H, H1), 7.37 (s, 2 H, H13), 6.88 (q, *J* = 1.1 Hz, 4 H, H10), 3.98 (dd, *J* = 10.6, 7.4 Hz, 2 H, H6), 3.92 (dd, *J* = 10.6, 8.9 Hz, 2 H, H5a), 3.65 (dd, *J* = 8.9, 7.4 Hz, 2 H, H5b), 0.90 (s, 18 H, H8).
^{13}C NMR (150 MHz, $CDCl_3$): δ 157.3 (C4), 148.3 – 148.2 (br, C3), 143.9 (C9), 137.8 (C1), 131.5 (q, *J* = 33.3 Hz, C11), 125.6 (C2), 123.1 (q, *J* = 272.8 Hz, C12), 121.1 – 120.09 (br, C10), 115.8 – 115.5 (br, C13), 74.2 (C6), 54.5 (C5), 34.4 (C7), 25.8 (C8).
^{19}F NMR (376 MHz, $CDCl_3$): δ -63.2 (s, 12 F, F12).
FTIR (ν_{max}, cm^{-1}): 2960 (w), 2871 (w), 1685 (w), 1610 (w), 1570 (w), 1520 (w), 1475 (m), 1396 (m), 1365 (m), 1274 (s), 1171 (m), 1126 (s), 1108 (m), 1057 (m), 1022 (w), 996 (w), 879 (w), 846 (w), 827 (w), 756 (m).
HRMS (ESI): calculated for $C_{35}H_{34}F_{12}N_5$ $[M+H]^+$ 752.2617, found 752.2640.
R_f = 0.29 (4% MeOH/CH_2Cl_2).
$[\alpha]_D^{28.4}$ = -100.1 ($CHCl_3$, c = 1.0).

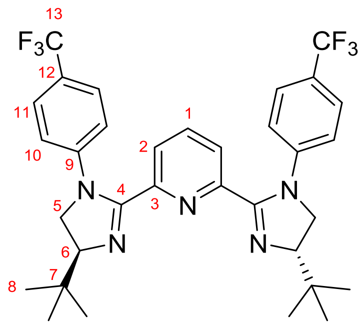

2,6-bis((*S*)-4-(*tert*-butyl)-1-(4-(trifluoromethyl)phenyl)-4,5-dihydro-1*H*-imidazol-2-yl)pyridine (L8): Following the general procedure for PyBIM synthesis, using N^2,N^6-bis((*S*)-1-hydroxy-3,3-dimethylbutan-2-yl)pyridine-2,6-dicarboxamide and 4-trifluoromethylaniline (0.14 mL, 1.1 mmol, 2.2 equiv.) at r.t. for 16 h, purified by silica gel column chromatography (eluent: 5% MeOH/CH_2Cl_2) provided the title compound as a yellow gum (215.1 mg, 0.349 mmol, 70%).

^{1}H NMR (600 MHz, $CDCl_3$): δ 7.88 (d, *J* = 7.8 Hz, 2 H, H2), 7.79 (t, *J* = 7.8 Hz, 1 H, H1), 7.27 (d, *J* = 8.5 Hz, 4 H, H11), 6.51 (d, *J* = 8.5 Hz, 4 H, H10), 3.96 – 3.88 (m, 4 H, H5a and H6), 3.66 – 3.59 (m, 2 H, H5b), 0.90 (s, 18 H, H8).
^{13}C NMR (150 MHz, $CDCl_3$): δ 158.1 (C4), 149.1 (C3), 145.4 (C9), 137.5 (C1), 125.3 (q, *J* = 3.7 Hz, C11), 125.1 (C2), 124.4 (q, *J* = 271.3 Hz, C13), 124.1 (q, *J* = 32.6 Hz, C12), 121.2 (C10), 73.9 (C6), 54.3 (C5), 34.3 (C7), 25.8 (C8).
^{19}F NMR (376 MHz, $CDCl_3$): δ -61.8 (s, 6 F, F13).
FTIR (ν_{max}, cm^{-1}): 2953 (w), 1604 (w), 1569 (w), 1523 (w), 1481 (w), 1412 (w), 1364 (w), 1323 (s), 1161 (m), 1114 (m), 1070 (m), 1047 (w), 1014 (w), 833 (w), 757 (w).
HRMS (ESI): calculated for $C_{33}H_{36}F_6N_5$ $[M+H]^+$ 616.2869, found 616.2861.
$\boldsymbol{R_f}$ = 0.07 (5% MeOH/CH_2Cl_2).
$[\alpha]_D^{28.4}$ = -44.5 ($CHCl_3$, c = 1.0).

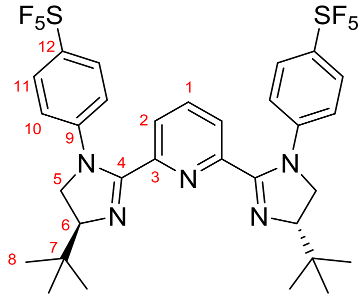

2,6-bis((*S*)-4-(*tert*-butyl)-1-(4-(pentafluorosulfanyl)phenyl)-4,5-dihydro-1*H*-imidazol-2-yl)pyridine (L9): Following the general procedure for PyBIM synthesis, using N^2,N^6-bis((*S*)-1-hydroxy-3,3-dimethylbutan-2-yl)pyridine-2,6-dicarboxamide and 4-(pentafluorothio)-aniline (0.241 g, 1.1 mmol, 2.2 equiv.) at r.t. for 72 h, purified by silica gel column chromatography (eluent: 3% → 5% MeOH/CH_2Cl_2) provided the title compound as an off-white foam (208.6 mg, 0.285 mmol, 57%). For scale-up to 5.0 mmol with respect to the imidoyl chloride, the general procedure was slightly modified, using 4-(pentafluorothio)aniline (3.29 g, 15.0 mmol, 3 equiv.), which provided the title compound as an off-white foam (1.54 g, 2.1 mmol, 42%).

^{1}H NMR (600 MHz, $CDCl_3$): δ 8.02 (d, *J* = 7.8 Hz, 2 H, H2), 7.83 (t, *J* = 7.8 Hz, 1 H, H1), 7.36 (d, *J* = 9.0 Hz, 4 H, H11), 6.41 (d, *J* = 9.0 Hz, 4 H, H10), 3.95 – 3.87 (m, 4 H, H5a and H6), 3.66 – 3.58 (m, 2 H, H5b), 0.91 (s, 18 H, H8).

^{13}C NMR (150 MHz, CDCl$_3$): δ 157.4 (C4), 148.5 (C3), 147.5 (qn, *J* = 17.3 Hz, C12), 144.7 (C9), 137.6 (C1), 125.7 (qn, *J* = 4.1 Hz, C11), 125.4 (C2), 120.4 (C10), 73.7 (C6), 54.2 (C5), 34.3 (C7), 25.8 (C8).
^{19}F NMR (376 MHz, CDCl$_3$): δ 86.3 (qn, *J* = 150.1 Hz, 2 F, SF_{ax}), 63.9 (d, *J* = 150.1 Hz, 8 F, SF_{eq}).
FTIR (ν_{max}, cm^{-1}): 2958 (w), 1596 (m), 1570 (w), 1507 (m), 1481 (m), 1429 (w), 1393 (m), 1365 (w), 1334 (w), 1208 (w), 1160 (w), 1103 (m), 1012 (w), 828 (s), 757 (w).
HRMS (ESI): calculated for $C_{31}H_{36}F_{10}N_5S_2$ $[M+H]^+$ 732.2247, found 732.2236.
R_f = 0.09 (5% MeOH/CH_2Cl_2).
$[\alpha]_D^{25.1}$ = -63.4 ($CHCl_3$, c = 1.0).

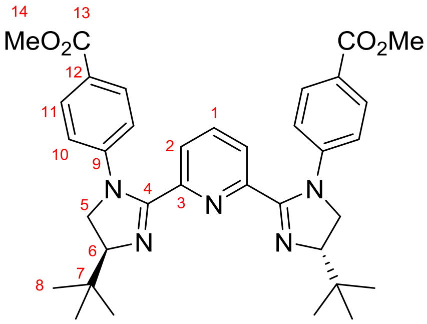

Dimethyl 4,4'-((4*S*,4'*S*)-pyridine-2,6-diylbis(4-(*tert*-butyl)-4,5-dihydro-1*H*-imidazole-2,1-diyl))dibenzoate: Following the general procedure for PyBIM synthesis, using *N*^{2},*N*^{6}-bis((*S*)-1-hydroxy-3,3-dimethylbutan-2-yl)pyridine-2,6-dicarboxamide and methyl 4-aminobenzoate (0.166 g, 1.1 mmol, 2.2 equiv.) at r.t. for 72 h, purified by silica gel column chromatography (eluent: 4% → 10% MeOH/CH_2Cl_2) provided the title compound as an orange foam (132.0 mg, 0.222 mmol, 44%).

^{1}H NMR (600 MHz, CDCl$_3$): δ 7.80 – 7.75 (m, 3 H, H1 and H2), 7.73 (d, *J* = 8.8 Hz, 4 H, H11), 6.50 (d, *J* = 8.8 Hz, 4 H, H10), 3.98 – 3.91 (m, 4 H, H5a and H6), 3.85 (s, 6 H, H14), 3.71 – 3.63 (m, 2 H, H5b), 0.89 (s, 18 H, H8).
^{13}C NMR (150 MHz, CDCl$_3$): δ 166.8 (C13), 158.4 (C4), 149.7 (br, C3), 146.5 (C9), 137.5 (C1), 130.1 (C11), 125.0 (C2), 123.7 (C12), 120.5 (C10), 74.1 (C6), 54.2 (C5), 52.0 (C14), 34.3 (C7), 25.9 (C8).
FTIR (ν_{max}, cm^{-1}): 2953 (m), 2869 (w), 1715 (s), 1603 (s), 1568 (m), 1516 (m), 1479 (m), 1434 (m), 1382 (m), 1363 (m), 1332 (w), 1276 (s), 1181 (m), 1112 (m), 1048 (w), 1000 (w), 932 (w), 847 (w), 827 (w), 769 (m), 753 (m).
HRMS (ESI): calculated for $C_{35}H_{42}N_5O_4$ $[M+H]^+$ 596.3231, found 596.3228.
R_f = 0.26 (5% MeOH/CH_2Cl_2).
$[\alpha]_D^{25.1}$ = -47.1 ($CHCl_3$, c = 1.0).

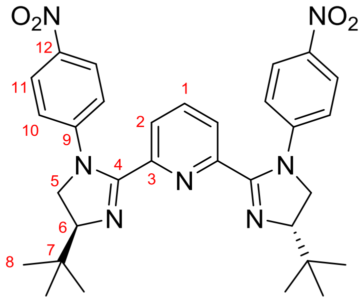

2,6-bis((*S*)-4-(*tert*-butyl)-1-(4-nitrophenyl)-4,5-dihydro-1*H*-imidazol-2-yl)pyridine (L10): Following the general procedure for PyBIM synthesis, using *N*^{2},*N*^{6}-bis((*S*)-1-hydroxy-3,3-

dimethylbutan-2-yl)pyridine-2,6-dicarboxamide and 4-nitroaniline (0.152 g, 1.1 mmol, 2.2 equiv.) at r.t. for 72 h, purified by silica gel column chromatography (eluent: 5% MeOH/CH_2Cl_2) provided the title compound as a yellow foam (119.7 mg, 0.210 mmol, 42%).

^{1}H NMR (600 MHz, CDCl$_3$): δ 7.93 (d, *J* = 7.7 Hz, 2 H, H2), 7.91 – 7.85 (m, 5 H, H1 and H10), 6.46 (d, *J* = 9.0 Hz, 4 H, H11), 4.00 – 3.94 (m, 2 H, H6), 3.90 (appears t, *J* = 9.8 Hz, 2 H, H5a), 3.69 (appears t, *J* = 8.4 Hz, 2 H, H5b), 0.89 (s, 18 H, H8).
^{13}C NMR (150 MHz, CDCl$_3$): δ 157.2 (C4), 149.1 (C3), 147.5 (C12), 141.7 (C9), 138.1 (C1), 125.4 (C2), 124.2 (C11), 119.8 (C10), 74.1 (C6), 53.9, (C5) 34.2 (C7), 25.8 (C8).
FTIR (ν_{max}, cm^{-1}): 2956 (w), 1590 (m), 1504 (m), 1478 (w), 1431 (w), 1389 (w), 1364 (w), 1325 (s), 1249 (w), 1187 (w), 1154 (w), 1113 (m), 1048 (w), 1000 (w), 854 (w), 843 (w), 751 (m).
HRMS (ESI): calculated for $C_{31}H_{36}N_7O_4$ $[M+H]^+$ 570.2823, found 570.2819.
$\boldsymbol{R_f}$ = 0.08 (5% MeOH/CH_2Cl_2).
$[\alpha]_D^{25.1}$ = -95.1 (CHCl$_3$, c = 1.0).

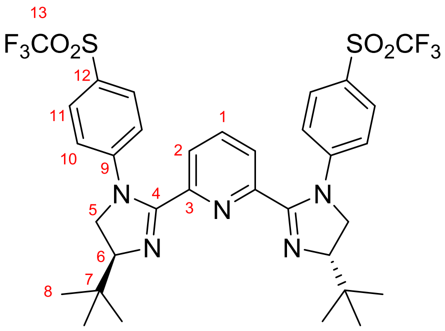

2,6-bis((*S*)-4-(*tert*-butyl)-1-(4-((trifluoromethyl)sulfonyl)phenyl)-4,5-dihydro-1*H*-imidazol-2-yl)pyridine (L11): Following the general procedure for PyBIM synthesis, using N^2,N^6-bis((*S*)-1-hydroxy-3,3-dimethylbutan-2-yl)pyridine-2,6-dicarboxamide and 4-((tri-fluoromethyl)sulfonyl)aniline (0.248 g, 1.1 mmol, 2.2 equiv.) at r.t. for 72 h, purified by silica gel column chromatography (eluent: 3% → 5% MeOH/CH_2Cl_2) provided the title compound as a yellow gum (69.6 mg, 0.094 mmol, 19%).

^{1}H NMR (600 MHz, CDCl$_3$): δ 8.10 (d, *J* = 7.8 Hz, 2 H, H2), 7.94 (t, *J* = 7.8 Hz, 1 H, H1), 7.61 (d, *J* = 8.8 Hz, 4 H, H11), 6.53 (d, *J* = 8.8 Hz, 4 H, H10), 3.99 (dd, *J* = 9.8, 7.7 Hz, 2 H, H6), 3.91 (t, *J* = 9.8 Hz, 2 H, H5a), 3.71 (dd, *J* = 9.8, 7.7 Hz, 2 H, H5b), 0.94 (s, 18 H, H8).
^{13}C NMR (150 MHz, CDCl$_3$): δ 156.5 (C4), 148.8 (C9), 148.3 (C3), 138.2 (C1), 131.0 (C11), 125.8 (C2), 121.8 (C12), 120.3 (C10), 120.1 (q, *J* = 325.7 Hz, C13), 74.0 (C6), 53.9 (C5), 34.3 (C7), 25.9 (C8).
^{19}F NMR (376 MHz, CDCl$_3$): δ -78.6 (s, 6 F, F13).
FTIR (ν_{max}, cm^{-1}): 2958 (m), 1587 (s), 1502 (m), 1479 (m), 1412 (w), 1216 (s), 1193 (s), 1141 (s), 1076 (s), 1003 (w), 830 (m), 768 (m).
HRMS (ESI): calculated for $C_{33}H_{36}F_6N_5O_4S_2$ $[M+H]^+$ 744.2107, found 744.2117.
$\boldsymbol{R_f}$ = 0.31 (5% MeOH/CH_2Cl_2).
$[\alpha]_D^{25.4}$ = -85.0 (CHCl$_3$, c = 1.0).

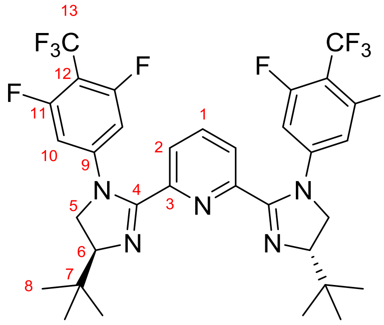

2,6-bis((*S*)-4-(*tert*-butyl)-1-(3,5-difluoro-4-(trifluoromethyl)phenyl)-4,5-dihydro-1*H*-imidazol-2-yl)pyridine: Following the general procedure for PyBIM synthesis, using N^2,N^6-bis((*S*)-1-hydroxy-3,3-dimethylbutan-2-yl)pyridine-2,6-dicarboxamide and 3,5-difluoro-4-(trifluoromethyl)aniline (0.217 g, 1.1 mmol, 2.2 equiv.) at r.t. for 72 h, purified by silica gel column chromatography (eluent: 3% MeOH/CH_2Cl_2) provided the title compound as a white foam (31.0 mg, 0.045 mmol, 9%).

^{1}H NMR (600 MHz, $CDCl_3$): δ 8.02 (d, *J* = 7.8 Hz, 2 H, H2), 7.91 (t, *J* = 7.8 Hz, 1 H, H1), 6.05 (d, *J* = 11.3 Hz, 4 H, H10), 3.99 (dd, *J* = 10.0, 8.2 Hz, 2 H, H6), 3.86 (t, *J* = 10.0 Hz, 2 H, H5a), 3.69 (dd, *J* = 10.0, 8.2 Hz, 2 H, H5b), 0.93 (s, 18 H, H8).
^{13}C NMR (150 MHz, $CDCl_3$): δ 159.7 (approx. dd, *J* = 256.0, 7.5 Hz, C11), 156.6 (C4), 148.2 (C3), 146.9 (t, *J* = 14.1 Hz, C9), 138.1 (C1), 125.8 (C2), 122.0 (q, *J* = 272.3 Hz, C13), 104.3 (d, *J* = 28.5 Hz, C10), 101.0-100.0 (br, C12, clearer on HMBC), 74.0 (C6), 54.0 (C5), 34.3 (C7), 25.8 (C8).
^{19}F NMR (376 MHz, $CDCl_3$): δ -55.5 (t, *J* = 21.3 Hz, 6 F, F13), -111.5 (q, *J* = 21.3 Hz, 4 F, F11).
FTIR (ν_{max}, cm^{-1}): 2958 (w), 2873 (w), 1644 (m), 1606 (m), 1576 (m), 1514 (w), 1480 (w), 1415 (w), 1400 (w), 1365 (w), 1301 (s), 1226 (m), 1186 (w), 1130 (m), 1083 (w), 1046 (m), 1033 (w), 1006 (w), 834 (w), 756 (w).
HRMS (ESI): calculated for $C_{33}H_{32}F_{10}N_5$ $[M+H]^+$ 688.2493, found 688.2493.
R_f = 0.29 (3% MeOH/CH_2Cl_2).
$[\alpha]_D^{25.0}$ = -53.6 ($CHCl_3$, c = 0.5).

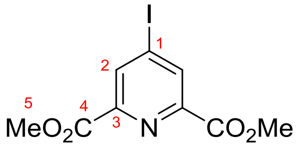

Dimethyl 4-iodopyridine-2,6-dicarboxylate:[20] To a solution of dimethyl 4-chloro-2,6,dicarboxylate (4.66 g, 20.3 mmol, 1 equiv.) in anhydrous MeCN (150 mL) was added acetyl chloride (4.33 mL, 60.9 mmol, 3 equiv.) and then sodium iodide (60.9 g, 406.0 mmol, 20 equiv.). The mixture was sonicated for 5 h, keeping the bath temperature below 30 °C. The mixture was then cooled to 0 °C and saturated aqueous Na_2CO_3 solution (75 mL) and CH_2Cl_2 (150 mL) were added. The organic layer was separated and then washed with saturated aqueous $Na_2S_2O_3$ solution (100 mL), water (2 × 100 mL), dried ($MgSO_4$) and evaporated under reduced pressure. The residue was recrystallised from MeOH to provide the title product as white crystalline needles (4.35 g, 13.6 mmol, 67%), m.p. 175-176 °C (lit. m.p.[20] 174-175 °C). Data are consistent with a reported example.[20]

^{1}H NMR (600 MHz, $CDCl_3$): δ 8.66 (s, 2 H, H2), 4.02 (s, 6 H, H5).
^{13}C NMR (150 MHz, $CDCl_3$): δ 164.0 (C4), 148.4 (C3), 137.3 (C2), 107.1 (C1), 53.6 (C5).

FTIR (ν_{max}, cm^{-1}): 3068 (w), 2949 (w), 1708 (s), 1566 (m), 1442 (s), 1324 (s), 1262 (s), 1241 (m), 1194 (m), 1142 (s), 981 (m), 960 (m), 888 (m), 825 (m), 778 (s).
HRMS (ESI): calculated for $C_9H_9NO_4I$ $[M+H]^+$ 321.9571, found 321.9578.

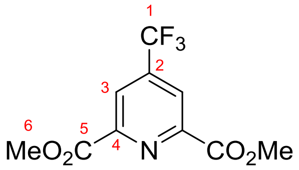

Dimethyl 4-(trifluoromethyl)pyridine-2,6-dicarboxylate:[21] To a mixture of dimethyl 4-iodopyridine-2,6-dicarboxylate (1.41 g, 4.4 mmol, 1 equiv.), copper(I) iodide (4.99 g, 26.2 mmol, 6 equiv.) and (dppf)$PdCl_2$•CH_2Cl_2 (0.18 g, 0.22 mmol, 0.05 equiv.) in anhydrous DMF (70 mL) was added a solution of methyl 2,2-difluoro-2-(fluorosulfonyl)acetate (5.03 g, 26.2 mmol, 6 equiv.) in anhydrous DMF (20 mL). The mixture was stirred at 100 °C for 16 h. The mixture was cooled to r.t. then diluted with CH_2Cl_2 (150 mL). The mixture was filtered through a pad of Celite, eluting with CH_2Cl_2, then the filtrate was washed with water (2 × 250 mL), brine/water (1:1, 250 mL), brine (250 mL), dried ($MgSO_4$) and evaporated under reduced pressure. The residue was purified by silica gel column chromatography (eluent: 40% EtOAc/hexane) to provide the title product as a yellow amorphous solid (1.03 g, 3.93 mmol, 89%), m.p. 122-123 °C (lit. m.p.[21] 122-124 °C). Data are consistent with a reported example.[21]

^{1}H NMR (600 MHz, CDCl$_3$): δ 8.52 (s, 2 H, H3), 4.07 (s, 6 H, H6).
^{13}C NMR (150 MHz, CDCl$_3$): δ 164.1 (C5), 149.8 (C4), 141.3 (q, J = 35.6 Hz, C2), 123.9 (q, J = 3.5 Hz, C3), 122.1 (q, J = 273.8 Hz, C1), 53.8 (C6).
^{19}F NMR (376 MHz, CDCl$_3$): δ -64.7 (s, 3 F, F1).
FTIR (ν_{max}, cm^{-1}): 3083 (w), 2964 (w), 1718 (s), 1630 (w), 1448 (m), 1435 (w), 1383 (w), 1274 (s), 1252 (s), 1198 (m), 1168 (s), 1129 (s), 985 (m), 970 (m), 936 (m), 891 (m), 851 (m), 784 (m).
HRMS (ESI): calculated for $C_{10}H_9F_3NO_4$ $[M+H]^+$ 264.0478, found 264.0482.
$\boldsymbol{R_f}$ = 0.51 (40% EtOAc/hexane).

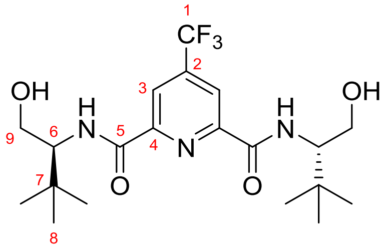

***N*^{2},*N*^{6}-bis((*S*)-1-hydroxy-3,3-dimethylbutan-2-yl)-4-(trifluoromethyl)pyridine-2,6-dicarboxamide:**[21] To a 5 mL microwave vial was added dimethyl 4-(trifluoromethyl)pyridine-2,6-dicarboxylate (0.526 g, 2.0 mmol, 1 equiv.) and L-*tert*-leucinol (0.516 g, 4.4 mmol, 2.2 equiv.). The neat mixture was stirred at 120 °C for 2.5 h, whereupon a precipitate formed. The residue was purified by silica gel column chromatography (60% EtOAc/hexane → EtOAc) to provide the title product as a white amorphous solid (0.837 g, 1.93 mmol, 96%), m.p. 186-188 °C (lit. m.p.[21] 178-180 °C). Compound has been prepared previously,[21] but NMR spectra were recorded in d$_6$-DMSO.

^{1}H NMR (600 MHz, CDCl$_3$): δ 8.60 (s, 2 H, H3), 8.04 (br d, J = 9.1 Hz, 2 H, NH), 4.05 – 3.99 (m, 2 H, H6), 3.99 – 3.94 (m, 2 H, H9a), 3.82 – 3.75 (m, 2 H, H9b), 2.23 (br s, 2 H, OH), 1.06 (s, 18 H, H8).
^{13}C NMR (150 MHz, CDCl$_3$): δ 163.0 (C5), 150.4 (C4), 142.2 (q, J = 35.3 Hz, C2), 122.3 (q, J = 274.0 Hz, C1), 121.4 (q, J = 3.4 Hz, C3), 63.2 (C9), 59.9 (C6), 34.2 (C7), 27.2 (C8).
^{19}F NMR (376 MHz, CDCl$_3$): δ -64.8 (s, 3 F, F1).
FTIR (ν_{max}, cm^{-1}): 3390 (br w), 3324 (w), 2967 (w), 1741 (w), 1664 (s), 1609 (w), 1539 (s), 1478 (w), 1435 (w), 1412 (w), 1366 (w), 1334 (w), 1283 (m), 1239 (w), 1220 (w), 1178 (m), 1151 (m), 1140 (s), 1096 (m), 1058 (w), 1046 (m), 1023 (w), 999 (w), 933 (w), 903 (w), 797 (w), 778 (w).
HRMS (ESI): calculated for $C_{20}H_{29}F_3N_3O_4$ [M-H]$^-$ 432.2116, found 432.2114.
$\boldsymbol{R_f}$ = 0.20 (50% EtOAc/hexane).
$[\alpha]_D^{28.4}$ = -5.2 (CHCl$_3$, c = 0.25); lit.[21] $[\alpha]_D^{28}$ = +6.32 (MeOH, c = 0.27).

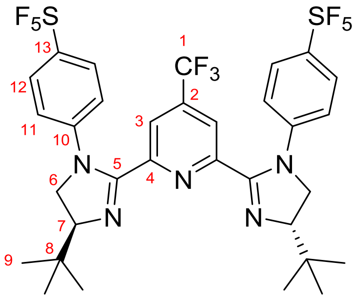

2,6-bis((*S*)-4-(*tert*-butyl)-1-(4-(pentafluorosulfanyl)phenyl)-4,5-dihydro-1*H*-imidazol-2-yl)-4-(trifluoromethyl)pyridine: Following the general procedure for PyBIM synthesis, using N^2,N^6-bis((*S*)-1-hydroxy-3,3-dimethylbutan-2-yl)-4-(trifluoromethyl)pyridine-2,6-dicarboxamide and 4-(pentafluorothio)aniline (0.241 g, 1.1 mmol, 2.2 equiv.) at 60 °C for 5 d, purified by silica gel column chromatography (eluent: 1% → 1.5% MeOH/CH$_2$Cl$_2$) provided the title compound as an off-white amorphous solid (196.0 mg, 0.245 mmol, 49%), m.p. 201-202 °C.

^{1}H NMR (600 MHz, CDCl$_3$): δ 8.33 (s, 2 H, H3), 7.38 (d, J = 8.9 Hz, 4 H, H12), 6.36 (d, J = 8.9 Hz, 4 H, H11), 3.95 (dd, J = 10.7, 7.7 Hz, 2 H, H7), 3.88 (dd, J = 10.7, 9.2 Hz, 2 H, H6a), 3.64 (dd, J = 9.2, 7.7 Hz, 2 H, H6b), 0.94 (s, 18 H, H9).
^{13}C NMR (150 MHz, CDCl$_3$): δ 156.4 (C5), 149.5 (C4), 147.9 (qn, J = 17.1 Hz, C13), 144.5 (C10), 140.1 (q, J = 34.8 Hz, C2), 125.8 (qn, J = 4.2 Hz, C12), 122.4 (q, J = 273.9 Hz, C1), 121.3 (q, J = 3.5 Hz, C3), 120.8 (C11), 73.9 (C7), 54.5 (C6), 34.4 (C8), 25.9 (C9).
^{19}F NMR (376 MHz, CDCl$_3$): δ 86.1 (qn, J = 150.1 Hz, 2 F, SF$_{ax}$), 63.9 (d, J = 150.1 Hz, 8 F, SF$_{eq}$), -64.5 (s, 3 F, F1).
FTIR (ν_{max}, cm^{-1}): 2959 (w), 2871 (w), 1594 (m), 1571 (m), 1506 (m), 1481 (m), 1448 (w), 1412 (w), 1395 (w), 1364 (m), 1327 (m), 1288 (m), 1269 (m), 1179 (m), 1147 (m), 1103 (m), 1024 (w), 907 (w), 826 (s), 790 (m), 755 (m).
HRMS (ESI): calculated for $C_{32}H_{35}N_5F_{13}S_2$ [M+H]$^+$ 800.2121, found 800.2103.
$\boldsymbol{R_f}$ = 0.20 (1% MeOH/CH$_2$Cl$_2$).
$[\alpha]_D^{28.4}$ = -84.2 (CHCl$_3$, c = 1.0).

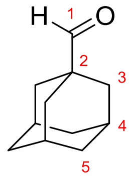

1-adamantanecarboxaldehyde:[22] To a solution of anhydrous DMSO (3.55 mL, 50.0 mmol, 2.5 equiv.) in anhydrous CH_2Cl_2 (50 mL) at -78 °C was added oxalyl chloride (2.20 mL, 26.0 mmol, 1.3 equiv.) slowly dropwise, keeping the internal temperature below -60 °C. The mixture was stirred further for 15 min. A solution of 1-adamantanemethanol (3.35 g, 20.0 mmol, 1 equiv.) in anhydrous CH_2Cl_2 (25 mL) was added slowly dropwise and the mixture then stirred for 1 h. Triethylamine (13.9 mL, 100.0 mmol, 5 equiv.) was then added and stirred for 30 min. The mixture was warmed to r.t., quenched with 10% aqueous KH_2PO_4 (50 mL) and diluted with Et_2O (50 mL). The organic layer was separated, washed with 10% aqueous KH_2PO_4 (3 × 50 mL), dried ($MgSO_4$) and evaporated under reduced pressure. The residue was purified by silica gel column chromatography (eluent: CH_2Cl_2) to provide the title product as a white amorphous solid (3.07 g, 18.7 mmol, 94%), m.p. 147-148 °C (lit. m.p.[23] 146-148 °C). Data are consistent with a reported example.[23]

^{1}H NMR (600 MHz, CDCl$_3$): δ 9.28 (s, 1 H, H1), 2.04 (s, 3 H, H4), 1.74 (d, *J* = 12.3 Hz, 3 H, H5a), 1.69 (s, 6 H, H3), 1.66 (d, *J* = 12.3 Hz, 3 H, H5b).
^{13}C NMR (150 MHz, CDCl$_3$): δ 206.1 (C1), 44.9 (C2), 36.6 (C5), 35.8 (C3), 27.4 (C4).
FTIR (ν_{max}, cm^{-1}): 2904 (s), 2850 (m), 2696 (w), 1802 (w), 1721 (s), 1451 (m), 1344 (w), 1264 (w), 1193 (w), 1143 (w), 1105 (w), 1052 (w), 988 (w), 905 (m).
HRMS (ESI): calculated for $C_{11}H_{17}O$ $[M+H]^+$ 165.1274, found 165.1279.
R_f = 0.76 (CH_2Cl_2).

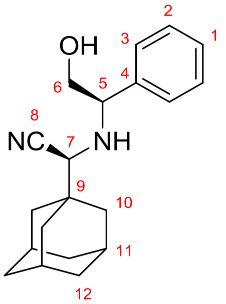

(*S*)-2-(adamantan-1-yl)-2-(((*R*)-2-hydroxy-1-phenylethyl)amino)acetonitrile:[22] To a mixture of 1-adamantanecarboxaldehyde (5.72 g, 34.8 mmol, 1 equiv.) in water (90 mL) was added sodium bisulfite (3.62 g, 34.8 mmol, 1 equiv.), then potassium cyanide (2.27 g, 34.8 mmol, 1 equiv.) at 0 °C. A solution of (*R*)-phenylglycinol (4.78 g, 34.8 mmol, 1 equiv.) in MeOH (9 mL) was then added dropwise. The mixture was stirred at r.t. for 2 h then under reflux for 16 h. The mixture was cooled to r.t. and extracted with EtOAc (2 × 100 mL). The combined organic extracts were washed with brine (50 mL), dried ($MgSO_4$) and evaporated under reduced pressure. The residue was purified by silica gel column chromatography (eluent: 20% EtOAc/hexane) to provide the title product as a white amorphous solid (2.41 g, 7.8 mmol, 22%), m.p. 122-124 °C (lit. m.p.[24] 118-120 °C for opposite enantiomer). Data are consistent with a reported example.[24]

^{1}H NMR (600 MHz, CDCl$_3$): δ 7.37 – 7.33 (m, 4 H, H2 and H3), 7.33 – 7.29 (m, 1 H, H1), 4.07 (dd, *J* = 9.2, 3.8 Hz, 1 H, H5), 3.80 (dt, *J* = 10.8, 3.8 Hz, 1 H, H6a), 3.61 – 3.54 (m, 1 H, H6b), 2.87 (s, 1 H, H7), 2.19 (br s, 1 H, OH), 2.04 (s, 3 H, H11), 1.80 – 1.70 (m, 6 H, H10a and H12a), 1.70 – 1.62 (m, 4 H, H12b and NH), 1.61 – 1.54 (m, 3 H, H10b).

^{13}C NMR (150 MHz, $CDCl_3$): δ 138.7 (C4), 128.9 (C2/C3), 128.4 (C1), 128.0 (C2/C3), 119.3 (C8), 67.8 (C6), 63.3 (C5), 59.3 (C7), 38.9 (C10), 36.9 (C12), 35.9 (C9), 28.3 (C11).
FTIR (ν_{max}, cm^{-1}): 3454 (br w), 2906 (s), 2850 (m), 2359 (w), 1454 (m), 1057 (m), 758 (m).
HRMS (ESI): calculated for $C_{20}H_{27}N_2O$ $[M+H]^+$ 311.2118, found 311.2111.
R_f = 0.32 (20% EtOAc/hexane). (The minor diastereomeric product is at 0.27).
$[\alpha]_D^{27.7}$ = -140.4 ($CHCl_3$, c = 1.0); lit.[24] $[\alpha]_D^{25}$ = +136.0 (MeOH, c = 0.25, for opposite enantiomer).

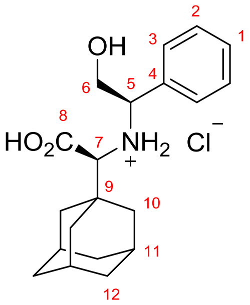

(*R*)-*N*-((*S*)-(adamantan-1-yl)(carboxy)methyl)-2-hydroxy-1-phenylethan-1-ammonium chloride:[22] A mixture of (*S*)-2-(adamantan-1-yl)-2-(((*R*)-2-hydroxy-1-phenylethyl)amino)-acetonitrile (2.41 g, 7.8 mmol) in 37% aqueous HCl (52 mL) and acetic acid (13 mL) was heated at 80 °C for 16 h. The mixture was then cooled to r.t. then at 0 °C, which resulted in the formation of a white precipitate after several hours. The precipitate was collected by vacuum filtration to provide the title product as a white crystalline solid (2.06 g, 5.6 mmol, 72%), m.p. >220 °C (dec.) (lit. m.p.[24] 228-230 °C for opposite enantiomer). Data are consistent with a reported example.[24]

^{1}H NMR (600 MHz, d_4-MeOD): δ 7.57 – 7.48 (m, 5 H, H1, H2 and H3), 4.27 (dd, *J* = 9.0, 4.7 Hz, 1 H, H5), 4.25 – 4.19 (m, 1 H, H6a), 3.98 (dd, *J* = 11.3, 4.7 Hz, 1 H, H6b), 3.20 (s, 1 H, H7), 2.01 (s, 3 H, H11), 1.77 – 1.71 (m, 6 H, H10a and H12a), 1.67 (d, *J* = 11.9 Hz, 3 H, H12b), 1.51 (d, *J* = 11.4 Hz, 3 H, H10b).
^{13}C NMR (150 MHz, d_4-MeOD): δ 169.5 (C8), 132.1 (C4), 131.7 (C1), 130.7 (C2/C3), 130.2 (C2/C3), 69.4 (C7), 67.0 (C5), 62.3 (C6), 39.1 (C10), 37.0 (C12), 36.3 (C9), 29.4 (C11).
FTIR (ν_{max}, cm^{-1}): 3600-2400 (br w), 3386 (br w), 2906 (s), 2851 (m), 1730 (m), 1556 (m), 1424 (m), 1207 (m), 1052 (m), 763 (m).
HRMS (ESI): calculated for $C_{20}H_{26}NO_3$ [of free base, $M-H]^-$ 328.1918, found 328.1918.
$[\alpha]_D^{27.7}$ = -5.7 (MeOH, c = 1.0); lit.[24] $[\alpha]_D^{25}$ = +100.9 (pyridine, c = 0.5, for opposite enantiomer).

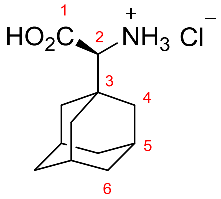

(*S*)-(adamantan-1-yl)(carboxy)methanammonium chloride:[22] A mixture of (*R*)-*N*-((*S*)-(adamantan-1-yl)(carboxy)methyl)-2-hydroxy-1-phenylethan-1-ammonium chloride (2.06 g, 5.6 mmol), 20% palladium(II) hydroxide on carbon (0.40 g) and acetic acid (4 mL) in methanol (20 mL) was stirred under H_2 (balloon) at r.t. for 4 d. The mixture was filtered through a pad of Celite, eluting with methanol, then the filtrate evaporated under reduced

pressure. The residue was triturated with Et_2O and filtered to provide the title product as a white crystalline solid (1.40 g, 5.6 mmol, 99%), m.p. >250 °C (dec.) (lit. m.p.[25] 247-292 °C (dec.)). Data are consistent with a reported example.[25]

^{1}H NMR (600 MHz, d$_4$-MeOD): δ 3.53 (s, 1 H, H2), 2.06 (s, 3 H, H5), 1.85 – 1.76 (m, 6 H, H4a and H6a), 1.71 (d, *J* = 11.8 Hz, 3 H, H6b), 1.65 (d, *J* = 12.1 Hz, 3 H, H4b).
^{13}C NMR (150 MHz, d$_4$-MeOD): δ 170.4 (C1), 63.5 (C2), 39.3 (C4), 37.3 (C6), 35.7 (C3), 29.5 (C5).
FTIR (ν_{max}, cm^{-1}): 3300-2400 (br w), 2890 (s), 2847 (s), 1741 (s), 1597 (m), 1582 (m), 1499 (s), 1454 (m), 1420 (m), 1374 (w), 1344 (w), 1316 (w), 1291 (w), 1256 (w), 1218 (s), 1201 (m), 1184 (w), 1140 (m), 1118 (m), 1107 (w), 1085 (m), 1035 (m), 976 (w), 938 (w), 911 (w), 858 (m), 832 (m), 815 (m).
HRMS (ESI): calculated for $C_{12}H_{18}NO_2$ [of free base, M-H]$^-$ 208.1343, found 208.1343.
$[\alpha]_D^{27.7}$ = +20.8 (MeOH, c = 1.0); lit.[25] $[\alpha]_D^{20}$ = +20.9 (MeOH, c = 0.93).

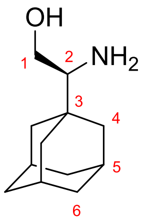

(*S*)-2-(adamantan-1-yl)-2-aminoethan-1-ol:[22] To a solution of $LiAlH_4$ (12.3 mL, 1.0 M in THF, 12.3 mmol, 2.2 equiv.) at 0 °C was added slowly portionwise (*S*)-(adamantan-1-yl)(carboxy)methanammonium chloride (1.40 g, 5.6 mmol, 1 equiv.). The mixture was then stirred under reflux for 16 h. The mixture was cooled to r.t., quenched slowly with Na_2SO_4•$10H_2O$ (6 g) and stirred vigorously for 1 h. The mixture was filtered through a pad of Celite, eluting copiously with EtOAc (150 mL). The filtrate was evaporated under reduced pressure to provide the title product as a white amorphous solid (0.98 g, 5.0 mmol, 90%), m.p. 98-100 °C. Data are consistent with a reported example.[22]

^{1}H NMR (600 MHz, CDCl$_3$): δ 3.69 (dd, *J* = 10.2, 3.8 Hz, 1 H, H1a), 3.24 (t, *J* = 10.2 Hz, 1 H, H1b), 2.32 (dd, *J* = 10.2, 3.8 Hz, 1 H, H2), 2.09 (br s, 3 H, OH and NH_2), 1.96 (s, 3 H, H5), 1.69 (d, *J* = 11.8 Hz, 3 H, H4a), 1.61 (d, *J* = 11.8 Hz, 3 H, H6a), 1.50 (s, 6 H, H4b and H6b).
^{13}C NMR (150 MHz, CDCl$_3$): δ 62.2 (C1), 61.3 (C2), 38.8 (C4), 37.3 (C6), 35.2 (C3), 28.4 (C5).
FTIR (ν_{max}, cm^{-1}): 3310 (br w), 2904 (m), 2849 (w), 1583 (w), 1450 (w), 1345 (w), 1047 (w), 986 (w), 905 (s), 858 (w).
HRMS (ESI): calculated for $C_{12}H_{22}NO$ [M+H]$^+$ 196.1696, found 196.1702.
$[\alpha]_D^{27.7}$ = +17.6 ($CHCl_3$, c = 1.0).

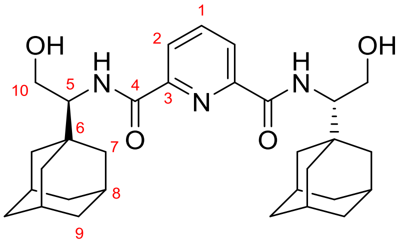

***N*^{2},*N*^{6}-bis((*S*)-1-(adamantan-1-yl)-2-hydroxyethyl)pyridine-2,6-dicarboxamide:** To a stirred solution of (*S*)-2-(adamantan-1-yl)-2-aminoethan-1-ol (0.488 g, 2.5 mmol, 2 equiv.)

and triethylamine (0.35 mL, 2.5 mmol, 2 equiv.) in anhydrous CH_2Cl_2 (13 mL) was added portionwise pyridine-2,6-dicarbonyl chloride (0.255 g, 1.25 mmol, 1 equiv.) at 0 °C. The mixture was warmed to r.t. and stirred further for 16 h. Water (10 mL) was added and the organic layer separated. The aqueous layer was extracted further with CH_2Cl_2 (3 × 10 mL) and the combined organic extracts dried ($MgSO_4$) and evaporated under reduced pressure. The residue was purified by silica gel column chromatography (eluent: 3% → 4% MeOH/CH_2Cl_2) to provide the title compound as a white foam (0.522 g, 1.0 mmol, 80%).

^{1}H NMR (600 MHz, $CDCl_3$): δ 8.21 (d, *J* = 7.8 Hz, 2 H, H2), 8.07 (br d, *J* = 9.5 Hz, 2 H, NH), 7.93 (t, *J* = 7.8 Hz, 1 H, H1), 3.92 (dd, *J* = 11.0, 3.4 Hz, 2 H, H10a), 3.84 – 3.77 (m, 2 H, H5), 3.73 (dd, *J* = 11.0, 7.1 Hz, 2 H, H10b), 3.16 (br s, 2 H, OH), 1.95 (s, 6 H, H8), 1.70 – 1.58 (m, 24 H, H7 and H9).
^{13}C NMR (150 MHz, $CDCl_3$): δ 164.4 (C4), 148.7 (C3), 139.2 (C1), 125.1 (C2), 61.7 (C10), 60.3 (C5), 39.3 (C7), 36.9 (C6), 36.0 (C9), 28.3 (C8).
FTIR (ν_{max}, cm^{-1}): 3401 (br w), 2902 (s), 2849 (m), 1727 (w), 1665 (s), 1528 (s), 1446 (m), 1343 (w), 1316 (w), 1238 (w), 1172 (w), 1105 (w), 1073 (w), 1052 (w), 1031 (w), 1001 (w), 970 (w), 843 (w), 753 (s).
HRMS (ESI): calculated for $C_{31}H_{44}N_3O_4$ $[M+H]^+$ 522.3326, found 522.3309.
R_f = 0.04 (3% MeOH/CH_2Cl_2).
$[\alpha]_D^{25.7}$ = -72.3 ($CHCl_3$, c = 1.0).

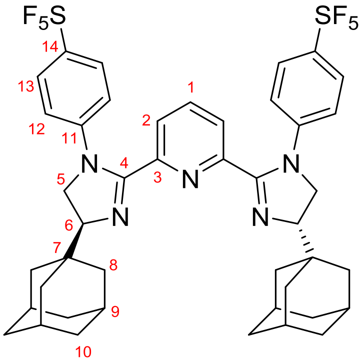

2,6-bis((*S*)-4-(adamantan-1-yl)-1-(4-(pentafluorosulfanyl)phenyl)-4,5-dihydro-1*H*-imidazol-2-yl)pyridine (L12): Following the general procedure for PyBIM synthesis, using N^2,N^6-bis((*S*)-1-(adamantan-1-yl)-2-hydroxyethyl)pyridine-2,6-dicarboxamide and 4-(pentafluorothio)aniline (0.241 g, 1.1 mmol, 2.2 equiv.) at 60 °C for 16 h, purified by silica gel column chromatography (eluent: 3% → 5% MeOH/CH_2Cl_2) provided the title compound as a pale yellow gum (319.0 mg, 0.359 mmol, 72%).

^{1}H NMR (600 MHz, $CDCl_3$): δ 8.04 (d, *J* = 7.8 Hz, 2 H, H2), 7.85 (t, *J* = 7.8 Hz, 1 H, H1), 7.36 (d, *J* = 9.0 Hz, 4 H, H13), 6.39 (d, *J* = 9.0 Hz, 4 H, H12), 3.86 (dd, *J* = 10.5, 9.2 Hz, 2 H, H5a), 3.75 (dd, *J* = 10.5, 6.6 Hz, 2 H, H6), 3.66 (dd, *J* = 9.2, 6.6 Hz, 2 H, H5b), 1.97 (s, 6 H, H9), 1.70 (d, *J* = 12.0 Hz, 6 H, H10a), 1.66 – 1.59 (m, 12 H, H8a and H10b), 1.45 (d, *J* = 11.8 Hz, 6 H, H8b).
^{13}C NMR (150 MHz, $CDCl_3$): δ 157.3 (C4), 148.4 (C3), 147.5 – 147.1 (m, C14), 144.6 (C11), 137.5 (C1), 125.7 (br, C13), 125.5 (C2), 120.3 (C12), 73.7 (C6), 52.7 (C5), 38.3 (C8), 37.2 (C10), 36.2 (C7), 28.3 (C9).
^{19}F NMR (376 MHz, $CDCl_3$): δ 86.4 (qn, *J* = 150.2 Hz, 2 F, SF_{ax}), 64.0 (d, *J* = 150.2 Hz, 8 F, SF_{eq}).

FTIR (ν_{max}, cm^{-1}): 2902 (m), 2849 (w), 1595 (m), 1568 (m), 1506 (m), 1479 (m), 1451 (w), 1430 (w), 1411 (w), 1392 (m), 1334 (w), 1316 (w), 1276 (w), 1245 (w), 1200 (w), 1163 (w), 1136 (w), 1103 (m), 1081 (w), 1008 (w), 907 (m), 822 (s), 778 (m).
HRMS (ESI): calculated for $C_{43}H_{48}F_{10}N_5S_2$ $[M+H]^+$ 888.3186, found 888.3177.
$\boldsymbol{R_f}$ = 0.28 (5% MeOH/CH_2Cl_2).
$[\alpha]_D^{28.5}$ = -126.4 ($CHCl_3$, c = 1.0).

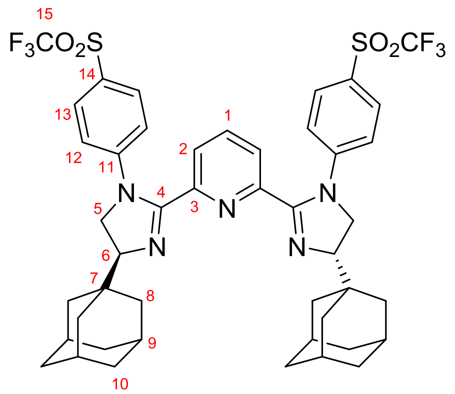

2,6-bis((*S*)-4-(adamantan-1-yl)-1-(4-((trifluoromethyl)sulfonyl)phenyl)-4,5-dihydro-1*H*-imidazol-2-yl)pyridine: Following the general procedure for PyBIM synthesis, using N^2,N^6-bis((*S*)-1-(adamantan-1-yl)-2-hydroxyethyl)pyridine-2,6-dicarboxamide and 4-((trifluoro-methyl)sulfonyl)aniline (0.248 g, 1.1 mmol, 2.2 equiv.) at 60 °C for 16 h, purified by silica gel column chromatography (eluent: 50% EtOAc/hexane) provided the title compound as a pale yellow foam (284.2 mg, 0.316 mmol, 63%).

^{1}H NMR (600 MHz, $CDCl_3$): δ 8.08 (d, *J* = 7.9 Hz, 2 H, H2), 7.92 (t, *J* = 7.9 Hz, 1 H, H1), 7.58 (d, *J* = 8.9 Hz, 4 H, H13), 6.50 (d, *J* = 8.9 Hz, 4 H, H12), 3.83 (dd, *J* = 9.8, 8.0 Hz, 2 H, H5a), 3.81 – 3.77 (m, 2 H, H6), 3.74 (dd, *J* = 8.0, 6.3 Hz, 2 H, H5b), 1.96 (s, 6 H, H9), 1.68 (d, *J* = 11.9 Hz, 6 H, H10a), 1.65 – 1.56 (m, 12 H, H8a and H10b), 1.44 (d, *J* = 12.0 Hz, 6 H, H8b).
^{13}C NMR (150 MHz, $CDCl_3$): δ 156.2 (C4), 148.6 (C11), 148.1 (C3), 138.0 (C1), 130.8 (C13), 125.7 (C2), 121.3 (C14), 120.2 (C12), 119.9 (q, *J* = 325.7 Hz, C15), 73.9 (C6), 52.2 (C5), 38.3 (C8), 37.0 (C10), 36.1 (C7), 28.1 (C9).
^{19}F NMR (376 MHz, $CDCl_3$): δ -78.7 (s, 6 F, F15).
FTIR (ν_{max}, cm^{-1}): 2904 (m), 2850 (w), 1586 (m), 1568 (w), 1501 (w), 1476 (w), 1411 (w), 1363 (m), 1315 (w), 1303 (w), 1276 (w), 1246 (w), 1215 (s), 1193 (m), 1140 (s), 1076 (m), 1039 (w), 1005 (w), 905 (s), 830 (m), 792 (w), 764 (w).
HRMS (ESI): calculated for $C_{45}H_{48}F_6N_5O_4S_2$ $[M+H]^+$ 900.3046, found 900.3045.
$\boldsymbol{R_f}$ = 0.37 (50% EtOAc/hexane).
$[\alpha]_D^{28.5}$ = -136.3 ($CHCl_3$, c = 1.0).

3. Synthetic procedures and characterisation for alkyne starting materials

General procedure for propargylamide synthesis via EDC coupling: To a solution of the appropriate carboxylic acid (5.0 mmol, 1.0 equiv.) in CH_2Cl_2 (20 mL) was added, if stated, DIPEA (1.05 mL, 6.0 mmol, 1.2 equiv.). EDC hydrochloride (1.15 g, 6.0 mmol, 1.2 equiv.) was added and stirred for 2 min, then HOBt (0.743 g, 5.5 mmol, 1.1 equiv.) was added and stirred for a further 2 min. Propargylamine (0.35 mL, 5.5 mmol, 1.1 equiv.) was then added and the mixture stirred at r.t. for 16 h. The mixture was diluted with CH_2Cl_2 (20 mL), washed with saturated aqueous Na_2CO_3 solution (25 mL) then 1 N aqueous HCl solution (20 mL). The organic phase was then dried ($MgSO_4$) and evaporated under reduced pressure. The residue was purified by silica gel column chromatography to provide the desired propargylamide.

General procedure for propargylamide synthesis via acyl chlorides: To a solution of the propargylamine (0.35 mL, 5.5 mmol, 1.1 equiv.) in CH_2Cl_2 (10 mL) was added Et_3N (0.77 mL, 5.5 mmol, 1.1 equiv.) and DMAP (61.1 mg, 0.5 mmol, 0.1 equiv.). The appropriate acyl chloride (5.0 mmol, 1.0 equiv.) was then added (dropwise if liquid, as a solution in 2 mL CH_2Cl_2 if solid) and the mixture stirred at r.t. for 16 h. The mixture was diluted with CH_2Cl_2 (10 mL) and treated with 1 N aqueous NaOH solution (20 mL). The organic layer was separated and the aqueous layer extracted further with CH_2Cl_2 (3 × 10 mL). The combined organic extracts were dried ($MgSO_4$) then evaporated under reduced pressure. The residue was purified by silica gel column chromatography to provide the desired propargylamide.

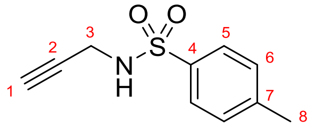

4-methyl-*N*-(prop-2-yn-1-yl)benzenesulfonamide (3a): To a solution of propargylamine (3.2 mL, 50.0 mmol, 1 equiv.) in CH_2Cl_2 (125 mL) at 0 °C was added Et_3N (17.4 mL, 125.0 mmol, 2.5 equiv.) then *p*-toluenesulfonyl chloride (9.53 g, 50.0 mmol, 1 equiv.) portionwise. The mixture was stirred at r.t. for 16 h. The reaction mixture was then diluted with Et_2O (500 mL) and the organic phase washed with 1 N aqueous HCl solution (300 mL), saturated aqueous NH_4Cl solution (300 mL), dried ($MgSO_4$) and evaporated under reduced pressure. The residual precipitate was triturated with hexane (3 × 25 mL) to provide the title product as an off-white amorphous solid (9.55 g, 45.6 mmol, 91%), m.p. 74-76 °C (lit. m.p.[26] 74-75 °C). Data are consistent with a reported example.[26]

^{1}H NMR (600 MHz, $CDCl_3$): δ 7.77 (d, *J* = 8.2 Hz, 2 H, H5), 7.31 (d, *J* = 8.2 Hz, 2 H, H6), 4.82 (br t, *J* = 5.8 Hz, 1 H, NH), 3.82 (dd, *J* = 5.8, 2.5 Hz, 2 H, H3), 2.43 (s, 3 H, H8), 2.10 (t, *J* = 2.5 Hz, 1 H, H1).
^{13}C NMR (150 MHz, $CDCl_3$): δ 144.0 (C7), 136.6 (C4), 129.8 (C6), 127.5 (C5), 78.1 (C2), 73.1 (C1), 33.0 (C3), 21.7 (C8).
FTIR (ν_{max}, cm^{-1}): 3276 (m), 1598 (w), 1495 (w), 1428 (w), 1324 (m), 1307 (m), 1292 (w), 1186 (w), 1156 (s), 1121 (w), 1093 (m), 1070 (m), 1019 (w), 993 (w), 925 (w), 813 (m).
HRMS (ESI): calculated for $C_{10}H_{12}NO_2S$ $[M+H]^+$ 210.0583, found 210.0586.

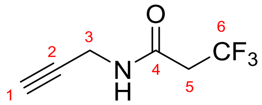

3,3,3-trifluoro-*N*-(prop-2-yn-1-yl)propanamide: Following the general procedure for propargylamide synthesis via acyl chlorides using 3,3,3-trifluoropropionyl chloride (1.54 mL, 15.0 mmol), purified by silica gel column chromatography (eluent: 35% EtOAc/hexane) provided the title compound as a white crystalline solid (1.66 g, 10.1 mmol, 67%), m.p. 76-77 °C.

^{1}H NMR (600 MHz, $CDCl_3$): δ 6.89 (br s, 1 H, NH), 4.06 (dd, *J* = 5.3, 2.6 Hz, 2 H, H3), 3.13 (q, *J* = 10.5 Hz, 2 H, H5), 2.25 (t, *J* = 2.5 Hz, 1 H, H1).
^{13}C NMR (150 MHz, $CDCl_3$): δ 163.2 (q, *J* = 3.5 Hz, C4), 124.0 (q, *J* = 276.7 Hz, C6), 78.7 (C2), 72.1 (C1), 41.2 (q, *J* = 29.6 Hz, C5), 29.6 (C3).
^{19}F NMR (376 MHz, $CDCl_3$): δ -63.1 (s, 3 F, F6).
FTIR (ν_{max}, cm^{-1}): 3317 (m), 3302 (m), 3098 (w), 1661 (s), 1567 (m), 1427 (m), 1373 (m), 1349 (w), 1311 (w), 1262 (s), 1242 (s), 1132 (m), 1106 (m), 1057 (m), 1035 (w), 928 (m), 854 (w), 778 (w).
HRMS (ESI): calculated for $C_6H_7F_3NO$ $[M+H]^+$ 166.0474, found 166.0468.
R$_f$ = 0.32 (35% EtOAc/hexane).

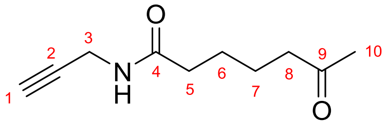

6-oxo-*N*-(prop-2-yn-1-yl)heptanamide: Following the general procedure for propargylamide synthesis via EDC coupling using 6-oxoheptanoic acid (0.800 g, 5.0 mmol, 90% purity), purified by silica gel column chromatography (eluent: 90% EtOAc/hexane) provided the title compound as a white crystalline solid (0.471 g, 2.60 mmol, 52%), m.p. 89-91 °C (lit. m.p.[27] 88-90 °C). Data are consistent with a reported example.[27]

^{1}H NMR (600 MHz, $CDCl_3$): δ 5.83 (br s, 1 H, NH), 4.05 (dd, *J* = 5.2, 2.5 Hz, 2 H, H3), 2.47 (t, *J* = 6.7 Hz, 2 H, H8), 2.23 – 2.19 (m, 3 H, H1 and H5), 2.14 (s, 3 H, H10), 1.67 – 1.56 (m, 4 H, H6 and H7).
^{13}C NMR (150 MHz, $CDCl_3$): δ 209.0 (C9), 172.3 (C4), 79.7 (C2), 71.7 (C1), 43.4 (C8), 36.2 (C5), 30.1 (C10), 29.3 (C3), 25.0 (C6/C7), 23.2 (C6/C7).
FTIR (ν_{max}, cm^{-1}): 3284 (s), 3078 (w), 2933 (w), 2862 (w), 1711 (m), 1703 (m), 1640 (s), 1556 (w), 1466 (w), 1424 (w), 1376 (w), 1358 (w), 1272 (w), 1241 (w), 1168 (w), 1106 (w), 1017 (w), 924 (w), 880 (w).
HRMS (ESI): calculated for $C_{10}H_{16}NO_2$ $[M+H]^+$ 188.1176, found 188.1183.
R$_f$ = 0.45 (90% EtOAc/hexane).

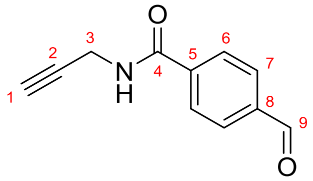

4-formyl-*N*-(prop-2-yn-1-yl)benzamide: Following the general procedure for propargylamide synthesis via EDC coupling using 4-formylbenzoic acid (0.751 g, 5.0 mmol), purified by silica gel column chromatography (eluent: 50% EtOAc/hexane) provided the title compound as a white amorphous solid (0.444 g, 2.37 mmol, 47%), m.p. 171-172 °C. Data are consistent with a reported example.[28]

^{1}H NMR (600 MHz, CDCl$_3$): δ 10.08 (s, 1 H, H9), 7.96 (d, J = 8.5 Hz, 2 H, H7), 7.94 (d, J = 8.5 Hz, 2 H, H6), 6.39 (br s, 1 H, NH), 4.28 (dd, J = 5.2, 2.6 Hz, 2 H, H3), 2.31 (t, J = 2.6 Hz, 1 H, H1).
^{13}C NMR (150 MHz, CDCl$_3$): δ 191.6 (C9), 166.1 (C4), 138.9 (C5), 138.6 (C8), 130.0 (C7), 127.9 (C6), 79.1 (C2), 72.5 (C1), 30.2 (C3).
FTIR (ν_{max}, cm^{-1}): 3316 (m), 3247 (w), 1712 (m), 1642 (s), 1540 (m), 1501 (w), 1416 (w), 1299 (w), 1259 (w), 1210 (w), 850 (w), 757 (w).
HRMS (ESI): calculated for $C_{11}H_{10}NO_2$ $[M+H]^+$ 188.0706, found 188.0704.
R$_f$ = 0.41 (50% EtOAc/hexane).

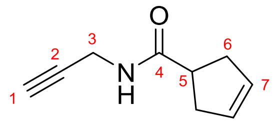

***N*-(prop-2-yn-1-yl)cyclopent-3-ene-1-carboxamide:** Following the general procedure for propargylamide synthesis via EDC coupling using 3-cyclopentene-1-carboxylic acid (0.561 g, 5.0 mmol), purified by silica gel column chromatography (eluent: 30% EtOAc/hexane) provided the title compound as a white amorphous solid (0.518 g, 3.47 mmol, 69%), m.p. 102-104 °C (lit. m.p.[29] 105-107 °C). Data are consistent with a reported example.[29]

^{1}H NMR (600 MHz, CDCl$_3$): δ 5.69 (s, 2 H, H7), 5.63 (br s, 1 H, NH), 4.07 (dd, J = 5.2, 2.5 Hz, 2 H, H3), 2.96 (qn, J = 8.0 Hz, 1 H, H5), 2.64 (d, J = 8.0 Hz, 4 H, H6), 2.23 (t, J = 2.5 Hz, 1 H, H1).
^{13}C NMR (150 MHz, CDCl$_3$): δ 175.6 (C4), 129.4 (C7), 79.8 (C2), 71.7 (C1), 43.4 (C5), 37.0 (C6), 29.5 (C3).
FTIR (ν_{max}, cm^{-1}): 3278 (m), 3062 (w), 2914 (w), 2848 (w), 1634 (s), 1618 (m), 1538 (m), 1440 (w), 1388 (w), 1343 (w), 1299 (w), 1234 (m), 1182 (w), 1039 (w), 948 (w), 847 (w).
HRMS (ESI): calculated for $C_9H_{12}NO$ $[M+H]^+$ 150.0913, found 150.0919.
R$_f$ = 0.39 (40% EtOAc/hexane).

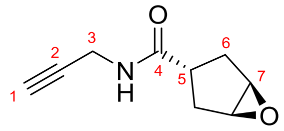

(1*R*,3*s*,5*S*)-*N*-(prop-2-yn-1-yl)-6-oxabicyclo[3.1.0]hexane-3-carboxamide: To a solution of *N*-(prop-2-yn-1-yl)cyclopent-3-ene-1-carboxamide (0.298 g, 2.0 mmol, 1 equiv.) in CH_2Cl_2 (4 mL) at 0 °C was added *m*-CPBA (0.583 g, 2.6 mmol, 1.3 equiv., 77% purity) portionwise. The mixture was then stirred at r.t. for 4 h. The mixture was filtered to remove precipitated *m*-chlorobenzoic acid, washed once on the filter with CH_2Cl_2 (1 mL), then the filtrate washed with saturated aqueous Na_2CO_3 solution. The organic phase was dried ($MgSO_4$), evaporated under reduced pressure and purified by silica gel column chromatography (eluent: 80% EtOAc/hexane) to provide the title compound *trans*-epoxide as a white amorphous solid (0.143 g, 0.87 mmol, 43%), m.p. 133-135 °C.

^{1}H NMR (600 MHz, CDCl$_3$): δ 5.71 (br s, 1 H, NH), 4.04 (dd, J = 5.2, 2.6 Hz, 2 H, H3), 3.53 (s, 2 H, H7), 2.40 (tt, J = 9.6, 7.9 Hz, 1 H, H5), 2.26 (dd, J = 14.0, 7.9 Hz, 2 H, H6a), 2.23 (t, J = 2.6 Hz, 1 H, H1), 1.96 (dd, J = 14.0, 9.6 Hz, 2 H, H6b).
^{13}C NMR (150 MHz, CDCl$_3$): δ 173.7 (C4), 79.5 (C2), 71.9 (C1), 56.6 (C7), 38.9 (C5), 31.9 (C6), 29.5 (C3).

FTIR (ν_{max}, cm^{-1}): 3286 (m), 3230 (m), 3050 (w), 2926 (w), 1635 (s), 1544 (m), 1446 (w), 1391 (w), 1294 (w), 1243 (w), 1224 (w), 1059 (w), 1035 (w), 958 (w), 832 (m).
HRMS (ESI): calculated for $C_9H_{12}NO_2$ $[M+H]^+$ 166.0863, found 166.0871.
R_f = 0.52 (80% EtOAc/hexane).

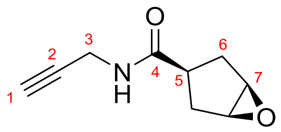

(1*R*,3*r*,5*S*)-*N*-(prop-2-yn-1-yl)-6-oxabicyclo[3.1.0]hexane-3-carboxamide: Following the epoxidation of *N*-(prop-2-yn-1-yl)cyclopent-3-ene-1-carboxamide, the *cis*-epoxide was also isolated as a white amorphous solid (0.149 g, 0.90 mmol, 45%), m.p. 114-116 °C.

^{1}H NMR (600 MHz, CDCl$_3$): δ 7.16 (br s, 1 H, NH), 3.95 (dd, *J* = 5.3, 2.5 Hz, 2 H, H3), 3.64 (s, 2 H, H7), 2.96 (tt, *J* = 10.3, 1.3 Hz, 1 H, H5), 2.23 (dd, *J* = 15.5, 1.3 Hz, 2 H, H6a), 2.19 (t, *J* = 2.5 Hz, 1 H, H1), 2.15 (dd, *J* = 15.5, 10.3 Hz, 2 H, H6b).
^{13}C NMR (150 MHz, CDCl$_3$): δ 176.2 (C4), 80.1 (C2), 71.2 (C1), 59.1 (C7), 42.5 (C5), 32.1 (C6), 29.3 (C3).
FTIR (ν_{max}, cm^{-1}): 3287 (m), 3038 (w), 2929 (w), 1652 (s), 1528 (s), 1431 (w), 1402 (w), 1352 (w), 1306 (w), 1243 (m), 1195 (w), 1100 (w), 1063 (w), 1010 (w), 948 (w), 921 (w), 889 (w), 841 (s).
HRMS (ESI): calculated for $C_9H_{12}NO_2$ $[M+H]^+$ 166.0863, found 166.0870.
R_f = 0.30 (80% EtOAc/hexane).

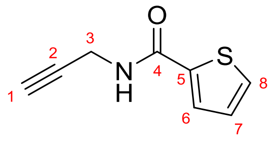

***N*-(prop-2-yn-1-yl)thiophene-2-carboxamide:** Following the general procedure for propargylamide synthesis via EDC coupling using 2-thiophenecarboxylic acid (0.641 g, 5.0 mmol), purified by silica gel column chromatography (eluent: 40% EtOAc/hexane) provided the title compound as an off-white amorphous solid (0.690 g, 4.17 mmol, 83%), m.p. 117-119 °C (lit. m.p.[29] 109-111 °C). Data are consistent with a reported example.[30]

^{1}H NMR (600 MHz, CDCl$_3$): δ 7.54 (dd, *J* = 3.8, 1.0 Hz, 1 H, H6), 7.49 (dd, *J* = 5.0, 1.0 Hz, 1 H, H8), 7.08 (dd, *J* = 5.0, 3.8 Hz, 1 H, H7), 6.21 (br s, 1 H, NH), 4.24 (dd, *J* = 5.3, 2.5 Hz, 2 H, H3), 2.28 (t, *J* = 2.5 Hz, 1 H, H1).
^{13}C NMR (150 MHz, CDCl$_3$): δ 161.6 (C4), 138.1 (C5), 130.5 (C8), 128.7 (C6), 127.8 (C7), 79.4 (C2), 72.1 (C1), 29.8 (C3).
FTIR (ν_{max}, cm^{-1}): 3289 (s), 3108 (w), 3085 (w), 3051 (w), 1626 (s), 1549 (s), 1516 (w), 1414 (m), 1358 (w), 1344 (w), 1310 (m), 1264 (w), 1248 (w), 1148 (w), 1062 (w), 1041 (w), 963 (w), 911 (w), 860 (w), 790 (w), 752 (w).
HRMS (ESI): calculated for C_8H_8NOS $[M+H]^+$ 166.0321, found 166.0324.
R_f = 0.40 (40% EtOAc/hexane).

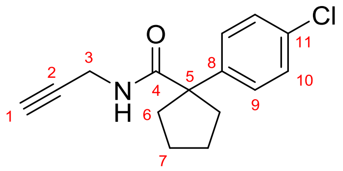

1-(4-chlorophenyl)-*N*-(prop-2-yn-1-yl)cyclopentane-1-carboxamide: Following the general procedure for propargylamide synthesis via acyl chlorides using 1-(4-chlorophenyl)-1-cyclopentanecarbonyl chloride (1.22 g, 5.0 mmol), purified by silica gel column chromatography (eluent: 30% EtOAc/hexane) provided the title compound as a white amorphous solid (0.909 g, 3.47 mmol, 69%), m.p. 123-124 °C.

^{1}H NMR (600 MHz, $CDCl_3$): δ 7.30 (d, *J* = 8.7 Hz, 2 H, H10), 7.27 (d, *J* = 8.7 Hz, 2 H, H9), 5.40 (br s, 1 H, NH), 3.92 (dd, *J* = 5.3, 2.5 Hz, 2 H, H3), 2.47 – 2.40 (m, 2 H, H6a), 2.14 (t, *J* = 2.5 Hz, 1 H, H1), 2.00 – 1.93 (m, 2 H, H6b), 1.83 – 1.77 (m, 2 H, H7a), 1.69 – 1.62 (m, 2 H, H7b).
^{13}C NMR (150 MHz, $CDCl_3$): δ 175.7 (C4), 142.3 (C11), 133.0 (C8), 129.0 (C10), 128.3 (C9), 79.5 (C2), 71.6 (C1), 58.8 (C5), 36.9 (C6), 29.7 (C3), 23.9 (C7).
FTIR (ν_{max}, cm^{-1}): 3306 (m), 3286 (m), 2957 (m), 2925 (m), 2874 (m), 1694 (m), 1637 (s), 1594 (w), 1526 (s), 1491 (m), 1462 (m), 1416 (m), 1401 (w), 1343 (w), 1275 (m), 1256 (m), 1219 (w), 1177 (w), 1093 (m), 1013 (m), 949 (w), 920 (w), 898 (w), 875 (w), 831 (m), 773 (w), 761 (w).
HRMS (ESI): calculated for $C_{15}H_{17}NOCl$ $[M+H]^+$ 262.0993, found 262.0990.
R_f = 0.38 (30% EtOAc/hexane).

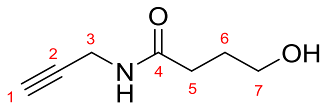

4-hydroxy-*N*-(prop-2-yn-1-yl)butanamide: A neat mixture of γ-butyrolactone (0.77 mL, 10.0 mmol, 1 equiv.) and propargylamine (0.96 mL, 15.0 mmol, 1.5 equiv.) in a 5 mL microwave vial were heated at 120 °C for 3 h. The mixture was then purified directly by silica gel column chromatography (eluent: EtOAc) to provide the title compound as a pale orange amorphous solid (0.994 g, 7.03 mmol, 70%), m.p. 37-40 °C.

^{1}H NMR (600 MHz, $CDCl_3$): δ 6.11 (br s, 1 H, NH), 4.05 (dd, *J* = 5.3, 2.6 Hz, 2 H, H3), 3.72 – 3.68 (m, 2 H, H7), 2.77 (br s, 1 H, OH), 2.37 (t, *J* = 6.9 Hz, 2 H, H5), 2.23 (t, *J* = 2.6 Hz, 1 H, H1), 1.93 – 1.86 (m, 2 H, H6).
^{13}C NMR (150 MHz, $CDCl_3$): δ 173.2 (C4), 79.6 (C2), 71.8 (C1), 62.3 (C7), 33.6 (C5), 29.4 (C3), 28.0 (C6).
FTIR (ν_{max}, cm^{-1}): 3600-3200 (br m), 3276 (m), 2941 (w), 1634 (s), 1544 (s), 1423 (m), 1339 (w), 1262 (m), 1168 (w), 1038 (m), 928 (w).
HRMS (ESI): calculated for $C_7H_{12}NO_2$ $[M+H]^+$ 142.0863, found 142.0868.
R_f = 0.19 (EtOAc).

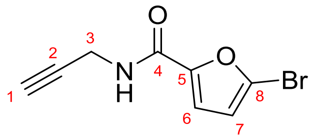

5-bromo-*N*-(prop-2-yn-1-yl)furan-2-carboxamide: Following the general procedure for propargylamide synthesis via EDC coupling using 5-bromo-2-furoic acid (0.995 g, 5.0 mmol), purified by silica gel column chromatography (eluent: 40% EtOAc/hexane) provided the title compound as a white crystalline solid (0.900 g, 3.95 mmol, 79%), m.p. 105-107 °C.

^{1}H NMR (600 MHz, $CDCl_3$): δ 7.09 (d, *J* = 3.5 Hz, 1 H, H6), 6.47 (br s, 1 H, NH), 6.45 (d, *J* = 3.5 Hz, 1 H, H7), 4.21 (dd, *J* = 5.5, 2.5 Hz, 2 H, H3), 2.28 (t, *J* = 2.5 Hz, 1 H, H1).

^{13}C NMR (150 MHz, $CDCl_3$): δ 156.8 (C4), 149.1 (C5), 124.9 (C8), 117.3 (C6), 114.4 (C7), 79.1 (C2), 72.2 (C1), 29.1 (C3).
FTIR (ν_{max}, cm^{-1}): 3299 (m), 3122 (w), 1650 (s), 1596 (m), 1574 (w), 1523 (s), 1469 (s), 1422 (w), 1352 (w), 1298 (m), 1204 (w), 1172 (w), 1126 (w), 1054 (w), 1014 (w), 938 (w), 925 (w), 799 (w), 753 (w).
HRMS (ESI): calculated for $C_8H_7NO_2Br$ $[M+H]^+$ 227.9655, found 227.9650.
R_f = 0.53 (40% EtOAc/hexane).

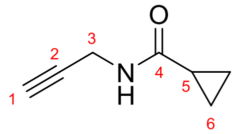

***N*-(prop-2-yn-1-yl)cyclopropanecarboxamide:** Following the general procedure for propargylamide synthesis via acyl chlorides using cyclopropanecarbonyl chloride (0.14 mL, 1.5 mmol), purified by silica gel column chromatography (eluent: 35% EtOAc/hexane) provided the title compound as a white crystalline solid (85.8 mg, 0.697 mmol, 46%), m.p. 70-71 °C (lit. m.p.[29] 64-66 °C). Data are consistent with a reported example.[29]

^{1}H NMR (600 MHz, $CDCl_3$): δ 5.83 (br s, 1 H, NH), 4.08 (dd, *J* = 5.3, 2.5 Hz, 2 H, H3), 2.23 (t, *J* = 2.5 Hz, 1 H, H1), 1.39 – 1.32 (m, 1 H, H5), 1.02 – 0.97 (m, 2 H, H6a), 0.80 – 0.73 (m, 2 H, H6b).
^{13}C NMR (150 MHz, $CDCl_3$): δ 173.8 and 173.7 (rotameric, C4), 79.9 (C2), 71.31 and 71.29 (rotameric, C1), 29.3 (C3), 14.48 and 14.46 (rotameric, C5), 7.4 (C6).
FTIR (ν_{max}, cm^{-1}): 3293 (m), 3064 (w), 3013 (w), 2924 (w), 1799 (w), 1727 (w), 1643 (s), 1537 (w), 1449 (m), 1421 (m), 1401 (m), 1349 (m), 1237 (s), 1197 (m), 1104 (m), 1061 (m), 1032 (m), 1012 (m), 931 (m), 896 (m), 825 (w).
HRMS (ESI): calculated for $C_7H_{10}NO$ $[M+H]^+$ 124.0757, found 124.0754.
R_f = 0.26 (35% EtOAc/hexane).

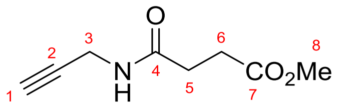

Methyl 4-oxo-4-(prop-2-yn-1-ylamino)butanoate: Following the general procedure for propargylamide synthesis via acyl chlorides using methyl 4-chloro-4-oxobutyrate (0.37 mL, 3.0 mmol), purified by silica gel column chromatography (eluent: 35% → 50% EtOAc/hexane) provided the title compound as a yellow crystalline solid (0.334 g, 1.97 mmol, 66%), m.p. 48-49 °C (lit. m.p.[31] 44-46 °C). Data are consistent with a reported example.[31]

^{1}H NMR (600 MHz, $CDCl_3$): δ 7.00 (br s, 1 H, NH), 3.89 (dd, *J* = 5.5, 2.6 Hz, 2 H, H3), 3.54 (s, 3 H, H8), 2.53 (t, *J* = 7.1 Hz, 2 H, H6), 2.42 (t, *J* = 7.1 Hz, 2 H, H5), 2.14 (t, *J* = 2.6 Hz, 1 H, H1).
^{13}C NMR (150 MHz, $CDCl_3$): δ 173.2 (C7), 171.4 (C4), 79.6 (C2), 71.1 (C1), 51.6 (C8), 30.3 (C6), 29.0 (C3/C5), 28.9 (C3/C5).
FTIR (ν_{max}, cm^{-1}): 3287 (m), 2955 (w), 2924 (w), 2851 (w), 1732 (s), 1651 (s), 1535 (s), 1438 (m), 1363 (m), 1201 (s), 1167 (s), 1089 (w), 1026 (m), 992 (w), 922 (w), 847 (w), 801 (w).
HRMS (ESI): calculated for $C_8H_{12}NO_3$ $[M+H]^+$ 170.0812, found 170.0816.
R_f = 0.10 (35% EtOAc/hexane).

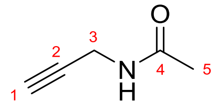

***N*-(prop-2-yn-1-yl)acetamide:** Following the general procedure for propargylamide synthesis via acyl chlorides using acetyl chloride (0.35 mL, 5.0 mmol), purified by silica gel column chromatography (eluent: 35% EtOAc/CH_2Cl_2) provided the title compound as a yellow crystalline solid (0.271 g, 2.79 mmol, 56%), m.p. 89-90 °C (lit. m.p.[32] 83-85 °C). Data are consistent with a reported example.[33]

^{1}H NMR (600 MHz, $CDCl_3$): δ 5.97 (br s, 1 H, NH), 4.03 (dd, *J* = 5.3, 2.5 Hz, 2 H, H3), 2.22 (t, *J* = 2.6 Hz, 1 H, H1), 2.00 (s, 3 H, H5).
^{13}C NMR (150 MHz, $CDCl_3$): δ 170.0 (C4), 79.7 (C2), 71.6 (C1), 29.4 (C3), 23.1 (C5).
FTIR (ν_{max}, cm^{-1}): 3293 (m), 3072 (w), 2922 (m), 2851 (w), 1644 (s), 1544 (s), 1423 (m), 1374 (m), 1287 (m), 1099 (w), 1029 (w), 927 (w).
HRMS (ESI): calculated for C_5H_7NONa $[M+Na]^+$ 120.0420, found 120.0425.
R$_f$ = 0.23 (35% EtOAc/CH_2Cl_2).

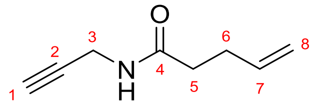

***N*-(prop-2-yn-1-yl)pent-4-enamide:** Following the general procedure for propargylamide synthesis via EDC coupling using 4-pentenoic acid (0.20 mL, 2.0 mmol), purified by silica gel column chromatography (eluent: 35% EtOAc/hexane) provided the title compound as a yellow oil (0.149 g, 1.09 mmol, 54%).

^{1}H NMR (600 MHz, $CDCl_3$): δ 6.82 (br s, 1 H, NH), 5.75 (ddt, *J* = 17.1, 10.2, 6.3 Hz, 1 H, H7), 4.99 (dd, *J* = 17.1, 1.5 Hz, 1 H, $H8_{trans}$), 4.92 (dd, *J* = 10.2, 1.5 Hz, 1 H, $H8_{cis}$), 3.96 (dd, *J* = 5.4, 2.6 Hz, 2 H, H3), 2.35 – 2.29 (m, 2 H, H6), 2.29 – 2.24 (m, 2 H, H5), 2.16 (t, *J* = 2.6 Hz, 1 H, H1).
^{13}C NMR (150 MHz, $CDCl_3$): δ 172.6 (C4), 136.8 (C7), 115.5 (C8), 79.7 (C2), 71.2 (C1), 35.3 (C5), 29.4 (C6), 29.0 (C3).
FTIR (ν_{max}, cm^{-1}): 3293 (m), 3078 (w), 2924 (w), 2855 (w), 1639 (s), 1536 (s), 1421 (m), 1343 (w), 1266 (m), 1194 (w), 1117 (w), 1031 (w), 996 (w), 915 (m).
HRMS (ESI): calculated for $C_8H_{12}NO$ $[M+H]^+$ 138.0913, found 138.0914.
R$_f$ = 0.22 (35% EtOAc/hexane).

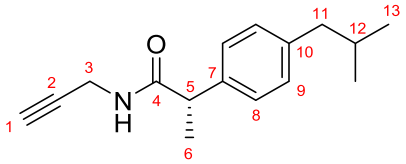

(*S*)-2-(4-isobutylphenyl)-*N*-(prop-2-yn-1-yl)propanamide: Following the general procedure for propargylamide synthesis via EDC coupling using (*S*)-ibuprofen (1.072 g, 5.2 mmol), purified by silica gel column chromatography (eluent: 25% EtOAc/hexane) provided the title compound as a white crystalline solid (1.071 g, 4.40 mmol, 85%), m.p. 75-76 °C.

^{1}H NMR (600 MHz, $CDCl_3$): δ 7.18 (d, *J* = 8.0 Hz, 2 H, H8), 7.12 (d, *J* = 8.0 Hz, 2 H, H9), 5.66 (br s, 1 H, NH), 4.03 (ddd, *J* = 17.6, 5.5, 2.5 Hz, 1 H, H3a), 3.92 (ddd, *J* = 17.6, 5.1, 2.5 Hz, 1 H, H3b), 3.55 (q, *J* = 7.2 Hz, 1 H, H5), 2.45 (d, *J* = 7.2 Hz, 2 H, H11), 2.16 (t, *J* =

2.5 Hz, 1 H, H1), 1.89 – 1.81 (m, 1 H, H12), 1.51 (d, J = 7.2 Hz, 3 H, H6), 0.90 (d, J = 6.7 Hz, 6 H, H13).
^{13}C NMR (150 MHz, $CDCl_3$): δ 174.2 (C4), 141.0 (C10), 138.2 (C7), 129.8 (C9), 127.5 (C8), 79.7 (C2), 71.5 (C1), 46.6 (C5), 45.1 (C11), 30.3 (C12), 29.5 (C3), 22.5 (C13), 18.6 (C6).
FTIR (ν_{max}, cm^{-1}): 3292 (m), 2955 (m), 2928 (m), 2869 (w), 1650 (s), 1537 (m), 1512 (m), 1465 (w), 1421 (w), 1366 (w), 1230 (w), 1073 (w), 1016 (w), 923 (w), 851 (w).
HRMS (ESI): calculated for $C_{16}H_{21}NONa$ $[M+Na]^+$ 266.1515, found 266.1502.
$\boldsymbol{R_f}$ = 0.29 (25% EtOAc/hexane).
$[\alpha]_D^{25.0}$ = +3.0 ($CHCl_3$, c = 1.0).

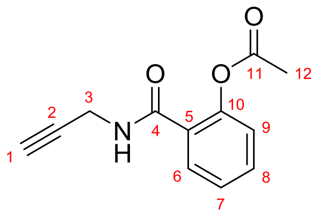

2-(prop-2-yn-1-ylcarbamoyl)phenyl acetate: Following the general procedure for propargylamide synthesis via EDC coupling using aspirin (0.901 g, 5.0 mmol), purified by silica gel column chromatography (eluent: 30% EtOAc/hexane) then trituration with Et_2O (1 mL) provided the title compound as a white crystalline solid (0.408 g, 1.88 mmol, 38%), m.p. 91-92 °C (lit. m.p.[31] 86-88 °C). Data are consistent with a reported example.[34]

^{1}H NMR (600 MHz, $CDCl_3$): δ 7.79 (dd, J = 8.0, 1.6 Hz, 1 H, H6), 7.47 (td, J = 8.0, 1.6 Hz, 1 H, H8), 7.30 (td, J = 8.0, 1.0 Hz, 1 H, H7), 7.11 (dd, J = 8.0, 1.0 Hz, 1 H, H9), 6.58 (br s, 1 H, NH), 4.19 (dd, J = 5.2, 2.6 Hz, 2 H, H3), 2.35 (s, 3 H, H12), 2.28 (t, J = 2.6 Hz, 1 H, H1).
^{13}C NMR (150 MHz, $CDCl_3$): δ 169.1 (C11), 165.1 (C4), 148.1 (C10), 132.2 (C8), 130.2 (C6), 127.5 (C5), 126.5 (C7), 123.4 (C9), 79.5 (C2), 72.1 (C1), 29.7 (C3), 21.1 (C12).
FTIR (ν_{max}, cm^{-1}): 3287 (w), 1766 (m), 1649 (m), 1608 (m), 1521 (m), 1481 (m), 1448 (w), 1424 (w), 1370 (m), 1302 (m), 1191 (s), 1101 (m), 1046 (w), 1012 (w), 988 (w), 955 (w), 913 (w), 874 (w), 833 (w), 788 (w), 751 (w).
HRMS (ESI): calculated for $C_{12}H_{11}NO_3Na$ $[M+Na]^+$ 240.0631, found 240.0621.
$\boldsymbol{R_f}$ = 0.14 (30% EtOAc/hexane).

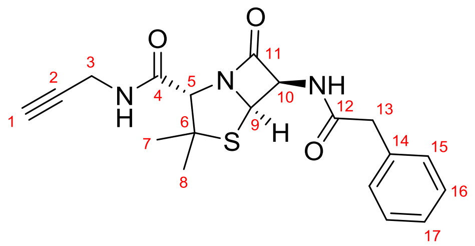

(2*S*,5*R*,6*R*)-3,3-dimethyl-7-oxo-6-(2-phenylacetamido)-*N*-(prop-2-yn-1-yl)-4-thia-1-azabicyclo[3.2.0]heptane-2-carboxamide: Penicillin G sodium salt (1.43 g, 4.0 mmol) was dissolved in 1 N HCl aqueous solution (30 mL) and then extracted with CH_2Cl_2 (3 × 25 mL). The combined organic extracts were dried ($MgSO_4$) and evaporated under reduced pressure to provide the carboxylic acid. Then, following the general procedure for propargylamide synthesis via EDC coupling using penicillin G (1.34 g, 4.0 mmol) and DMAP (48.9 mg, 0.4 mmol) as additive, purified by silica gel column chromatography (eluent: 40% EtOAc/hexane) provided the title compound as a pale yellow amorphous solid (0.570 g, 1.53 mmol, 38%), m.p. 140-142 °C.

^{1}H NMR (600 MHz, $CDCl_3$): δ 7.36 (t, *J* = 7.4 Hz, 2 H, H16), 7.30 (t, *J* = 7.4 Hz, 1 H, H17), 7.24 (d, *J* = 7.4 Hz, 2 H, H15), 6.79 (br t, *J* = 5.2 Hz, 1 H, C3-NH), 6.24 (br d, *J* = 9.2 Hz, 1 H, C10-NH), 5.73 (dd, *J* = 9.2, 4.5 Hz, 1 H, H10), 5.36 (d, *J* = 4.5 Hz, 1 H, H9), 4.11 (s, 1 H, H5), 4.07 (ddd, *J* = 17.6, 5.9, 2.5 Hz, 1 H, H3a), 3.92 (ddd, *J* = 17.6, 5.0, 2.5 Hz, 1 H, H3b), 3.64 – 3.56 (m, 2 H, H13), 2.23 (t, *J* = 2.5 Hz, 1 H, H1), 1.66 (s, 3 H, H7/H8), 1.48 (s, 3 H, H7/H8).
^{13}C NMR (150 MHz, $CDCl_3$): δ 176.4 (C11), 170.7 (C12), 167.2 (C4), 133.7 (C14), 129.5 (C15), 129.2 (C16), 127.8 (C17), 78.9 (C2), 72.4 (C5), 72.1 (C1), 66.4 (C9), 65.0 (C6), 57.4 (C10), 43.3 (C13), 29.1 (C3), 28.5 (C7/C8), 26.6 (C7/C8).
FTIR (ν_{max}, cm^{-1}): 3287 (m), 3061 (w), 2969 (w), 2932 (w), 1781 (m), 1654 (s), 1605 (w), 1525 (m), 1455 (w), 1422 (w), 1390 (w), 1371 (w), 1292 (m), 1238 (m), 1160 (w), 1130 (w), 1058 (w), 1031 (w), 939 (w), 893 (w).
HRMS (ESI): calculated for $C_{19}H_{22}N_3O_3S$ $[M+H]^+$ 372.1376, found 372.1387.
$\boldsymbol{R_f}$ = 0.24 (50% EtOAc/hexane).
$[\alpha]_D^{25.0}$ = +228.6 ($CHCl_3$, c = 1.0).

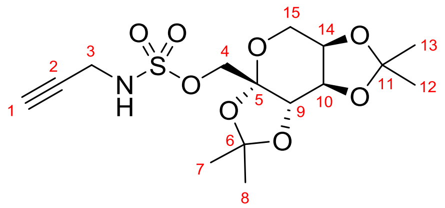

((3a*S*,5a*R*,8a*R*,8b*S*)-2,2,7,7-tetramethyltetrahydro-3a*H*-bis([1,3]dioxolo)[4,5-*b*:4',5'-*d*]pyran-3a-yl)methyl prop-2-yn-1-ylsulfamate: To a solution of topiramate (1.12 g, 3.3 mmol, 1 equiv.) in CH_2Cl_2 (6 mL) was added DMAP (4.0 mg, 0.033 mmol, 0.01 equiv.), Et_3N (0.51 mL, 3.63 mmol, 1.1 equiv.) and Boc anhydride (0.83 mL, 3.63 mmol, 1.1 equiv.). The mixture was stirred at r.t. for 16 h, then evaporated under reduced pressure to provide crude mono-Boc protected topiramate. The residue was redissolved in anhydrous THF (20 mL), then cooled to 0 °C. Triphenylphosphine (1.57 g, 6.0 mmol, 1.8 equiv.) and propargyl alcohol (0.35 mL, 6.0 mmol, 1.8 equiv.) were added, followed by dropwise addition of diisopropyl azodicarboxylate (1.18 mL, 6.0 mmol, 1.8 equiv.) over ca. 15 min. The mixture was warmed to r.t. and stirred further for 16 h. The mixture was then evaporated under reduced pressure then the residue triturated with Et_2O (25 mL). The triphenylphosphine oxide byproduct was filtered off, washed on the filter with Et_2O (25 mL) then the filtrate evaporated under reduced pressure. The residue was redissolved in CH_2Cl_2 (20 mL) then trifluoroacetic acid (4.8 mL, 50.0 mmol, 15.0 equiv.) was added and the mixture stirred at r.t. for 16 h. The reaction mixture was quenched with saturated aqueous $NaHCO_3$ solution (40 mL) and the organic layer separated. The aqueous layer was further extracted with CH_2Cl_2 (3 × 20 mL) and the combined organic extracts dried ($MgSO_4$) and evaporated under reduced pressure. The residue was purified by silica gel column chromatography (eluent: 10% Et_2O/CH_2Cl_2) to provide the title compound as a colourless gum (0.375 g, 0.99 mmol, 30% over three steps).

^{1}H NMR (600 MHz, $CDCl_3$): δ 4.84 (br t, *J* = 5.7 Hz, 1 H, NH), 4.61 (dd, *J* = 7.9, 2.6 Hz, 1 H, H10), 4.33 (d, *J* = 2.6 Hz, 1 H, H9), 4.26 (d, *J* = 10.6 Hz, 1 H, H4a), 4.24 (ddd, *J* = 7.9, 1.9, 0.7 Hz, 1 H, H14), 4.18 (d, *J* = 10.6 Hz, 1 H, H4b), 4.00 – 3.91 (m, 2 H, H3), 3.91 (dd, *J* = 13.0, 1.9 Hz, 1 H, H15a), 3.78 (dd, *J* = 13.0, 0.7 Hz, 1 H, H15b), 2.37 (t, *J* = 2.5 Hz, 1 H, H1), 1.55 (s, 3 H, H7/H8), 1.48 (s, 3 H, H12/H13), 1.42 (s, 3 H, H7/H8), 1.34 (s, 3 H, H12/H13).

^{13}C NMR (150 MHz, $CDCl_3$): δ 109.5 (C6), 109.4 (C11), 100.9 (C5), 78.0 (C2), 73.8 (C1), 70.9 (C14), 70.7 (C4), 70.5 (C9), 70.0 (C10), 61.5 (C15), 33.7 (C3), 26.6 (C7/C8), 26.0 (C12/C13), 25.3 (C7/C8), 24.2 (C12/C13).
FTIR (ν_{max}, cm^{-1}): 3276 (w), 2992 (w), 2941 (w), 1450 (w), 1371 (m), 1320 (w), 1252 (m), 1205 (m), 1176 (s), 1068 (s), 995 (s), 980 (s), 912 (m), 885 (s), 865 (s), 845 (m), 802 (m), 756 (m).
HRMS (ESI): calculated for $C_{15}H_{24}NO_8S$ $[M+H]^+$ 378.1217, found 378.1216.
R$_f$ = 0.36 (10% Et_2O/CH_2Cl_2).
$[\alpha]_D^{25.0}$ = -24.4 ($CHCl_3$, c = 1.0).

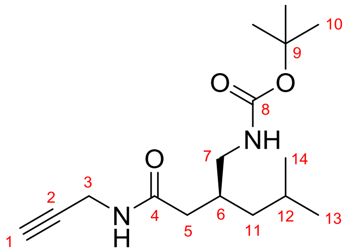

***tert*-butyl (*S*)-(4-methyl-2-(2-oxo-2-(prop-2-yn-1-ylamino)ethyl)pentyl)carbamate:** Following the general procedure for propargylamide synthesis via EDC coupling in the presence of DIPEA using Boc-pregabalin (1.037 g, 4.0 mmol), purified by silica gel column chromatography (eluent: 40% EtOAc/hexane) provided the title compound as a white amorphous solid (0.617 g, 2.08 mmol, 52%), m.p. 81-83 °C.

^{1}H NMR (600 MHz, $CDCl_3$): δ 6.92 (br s, 1 H, C3-NH), 4.82 (br s, 1 H, C7-NH), 4.09 – 3.99 (m, 2 H, H3), 3.23 (ddd, J = 14.1, 6.5, 3.9 Hz, 1 H, H7a), 3.01 (dt, J = 14.1, 6.5 Hz, 1 H, H7b), 2.21 (t, J = 2.5 Hz, 1 H, H1), 2.17 – 2.08 (m, 2 H, H5), 2.07 – 2.00 (m, 1 H, H6), 1.68 – 1.60 (m, 1 H, H12), 1.44 (s, 9 H, H10), 1.13 (t, J = 7.2 Hz, 2 H, H11), 0.93 – 0.85 (m, 6 H, H13 and H14).
^{13}C NMR (150 MHz, $CDCl_3$): δ 172.3 (br, C4), 157.1 (br, C8), 79.9 (br, C2), 79.7 (br, C9), 71.3 (br, C1), 43.5 (br, C7), 41.7 (br, C11), 39.2 (C5), 34.6 (br, C6), 29.3 (C3), 28.5 (C10), 25.3 (C12), 22.9 (C13/C14), 22.8 (C13/C14).
FTIR (ν_{max}, cm^{-1}): 3313 (m), 2958 (m), 2931 (m), 2870 (w), 1691 (s), 1647 (s), 1531 (s), 1452 (w), 1391 (w), 1366 (m), 1272 (m), 1251 (m), 1169 (s), 1023 (w), 856 (w).
HRMS (ESI): calculated for $C_{16}H_{28}N_2O_3Na$ $[M+Na]^+$ 319.1992, found 319.1980.
R$_f$ = 0.34 (40% EtOAc/hexane).
$[\alpha]_D^{25.0}$ = -10.2 ($CHCl_3$, c = 1.0).

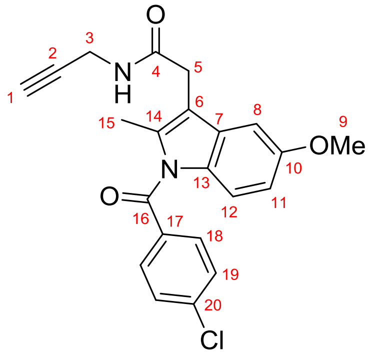

2-(1-(4-chlorobenzoyl)-5-methoxy-2-methyl-1*H*-indol-3-yl)-*N*-(prop-2-yn-1-yl)acetamide: Following the general procedure for propargylamide synthesis via EDC coupling using indomethacin (1.79 g, 5.0 mmol), purified by silica gel column chromatography (eluent: 50%

EtOAc/hexane) provided the title compound as a pale-yellow amorphous solid (0.636 g, 1.61 mmol, 32%), m.p. 171-173 °C. (N.B. Product is highly insoluble and the reaction mixture was diluted/extracted with copious CH_2Cl_2).

^{1}H NMR (600 MHz, $CDCl_3$): δ 7.67 (d, *J* = 8.7 Hz, 2 H, H18), 7.49 (d, *J* = 8.7 Hz, 2 H, H19), 6.89 – 6.86 (m, 2 H, H8 and H12), 6.71 (dd, *J* = 9.0, 2.5 Hz, 1 H, H11), 5.77 (br s, 1 H, NH), 4.01 (dd, *J* = 5.5, 2.5 Hz, 2 H, H3), 3.83 (s, 3 H, H9), 3.67 (s, 2 H, H5), 2.39 (s, 3 H, H15), 2.16 (t, *J* = 2.5 Hz, 1 H, H1).
^{13}C NMR (150 MHz, $CDCl_3$): δ 169.7 (C4), 168.5 (C16), 156.5 (C10), 139.8 (C20), 136.6 (C14), 133.7 (C17), 131.4 (C18), 131.0 (C13), 130.3 (C7), 129.4 (C19), 115.3 (C12), 112.6 (C11), 112.4 (C6), 100.9 (C8), 79.5 (C2), 71.7 (C1), 55.9 (C9), 32.2 (C5), 29.4 (C3), 13.4 (C15).
FTIR (ν_{max}, cm^{-1}): 3297 (m), 2930 (w), 1679 (s), 1641 (s), 1596 (m), 1532 (m), 1477 (s), 1401 (w), 1355 (s), 1322 (s), 1290 (w), 1265 (w), 1222 (s), 1179 (w), 1150 (m), 1089 (m), 1069 (m), 1038 (w), 1012 (w), 992 (w), 927 (w), 868 (w), 835 (w), 801 (w), 755 (m).
HRMS (ESI): calculated for $C_{22}H_{20}N_2O_3Cl$ $[M+H]^+$ 395.1157, found 395.1169.
$\boldsymbol{R_f}$ = 0.42 (5% EtOAc/hexane).

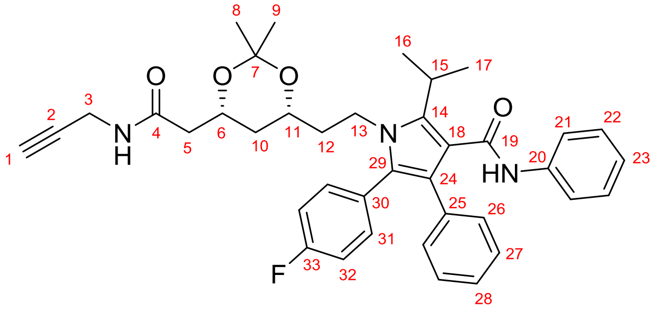

1-(2-((4*R*,6*R*)-2,2-dimethyl-6-(2-oxo-2-(prop-2-yn-1-ylamino)ethyl)-1,3-dioxan-4-yl)ethyl)-5-(4-fluorophenyl)-2-isopropyl-*N*,4-diphenyl-1*H*-pyrrole-3-carboxamide: Atorvastatin calcium salt (0.994 g, 1.66 mmol with respect to one atorvastatin equivalent, 1 equiv.) was dissolved in 1 N HCl aqueous solution (25 mL). The mixture was extracted with CH_2Cl_2 (3 × 25 mL) and the combined organic extracts were dried ($MgSO_4$) and evaporated under reduced pressure to provide the carboxylic acid. The residual foam was redissolved in acetone (8 mL) and 2,2-dimethoxypropane (2 mL), then (±)-camphorsulfonic acid (77.1 mg, 0.33 mmol, 0.2 equiv.) was added. The mixture was stirred at r.t. for 16 h. The reaction mixture was quenched with Et_3N (0.05 mL) and evaporated under reduced pressure to provide crude acetonide-protected atorvastatin. Then, following the general procedure for propargylamide synthesis via EDC coupling in the presence of DIPEA using crude acetonide-protected atorvastatin, purified by silica gel column chromatography (eluent: 50% EtOAc/hexane) provided the title compound as a white foam (0.470 g, 0.74 mmol, 45% over three steps).

^{1}H NMR (600 MHz, $CDCl_3$): δ 7.22 – 7.13 (m, 9 H, H22, H26, H27, H28 and H31), 7.06 (d, *J* = 7.9 Hz, 2 H, H21), 7.02 – 6.96 (m, 3 H, H23 and H32), 6.86 (br s, 1 H, C19-NH), 6.40 (br t, *J* = 4.9 Hz, 1 H, C3-NH), 4.17 – 4.11 (m, 1 H, H6), 4.11 – 4.04 (m, 1 H, H13a), 4.04 – 4.00 (m, 2 H, H3), 3.86 – 3.79 (m, 1 H, H13b), 3.72 – 3.65 (m, 1 H, H11), 3.62 – 3.53 (m, 1 H, H15), 2.34 (dd, *J* = 15.1, 7.4 Hz, 1 H, H5a), 2.29 (dd, *J* = 15.1, 4.1 Hz, 1 H, H5b), 2.21 (t, *J* = 2.5 Hz, 1 H, H1), 1.71 – 1.61 (m, 2 H, H12), 1.53 (superimposed d, *J* = 7.1 Hz, 6 H, H16 and H17), 1.38 (s, 3 H, H8/H9), 1.34 (s, 3 H, H8/H9), 1.31 (dt, *J* = 12.8, 2.3 Hz, 1 H, H10a), 1.09 (dt, *J* = 12.8, 11.7 Hz, 1 H, H10b).

^{13}C NMR (150 MHz, CDCl$_3$): δ 170.1 (C4), 164.9 (C19), 162.4 (d, *J* = 247.8 Hz, C33), 141.6 (C14), 138.5 (C20), 134.7 (C25), 133.3 (d, *J* = 8.1 Hz, C31), 130.6 (C26), 128.9 (C29), 128.8 (C27), 128.5 (C22), 128.4 (d, *J* = 3.3 Hz, C30), 126.7 (C28), 123.7 (C23), 122.0 (C24), 119.7 (C21), 115.52 (d, *J* = 21.5 Hz, C32), 115.50 (C18), 99.1 (C7), 79.6 (C2), 71.6 (C1), 66.5 (C11), 66.2 (C6), 42.8 (C5), 40.9 (C13), 38.1 (C12), 35.8 (C10), 30.1 (C8/C9), 29.2 (C3), 26.2 (C15), 21.9 (C16/C17), 21.7 (C16/C17), 19.9 (C8/C9).
^{19}F NMR (376 MHz, CDCl$_3$): δ -113.6 (s, 1 F, F33).
FTIR (ν_{max}, cm^{-1}): 3302 (w), 3051 (w), 2960 (w), 1652 (m), 1595 (m), 1526 (s), 1508 (s), 1436 (m), 1381 (m), 1313 (m), 1265 (m), 1222 (m), 1201 (m), 1169 (m), 1156 (s), 1114 (w), 1094 (w), 1032 (w), 994 (w), 964 (w), 942 (w), 918 (w), 886 (w), 841 (w), 809 (w).
HRMS (ESI): calculated for $C_{39}H_{43}FN_3O_4$ [M+H]$^+$ 636.3232, found 636.3236.
R_f = 0.35 (50% EtOAc/hexane).
$[\alpha]_D^{25.0}$ = -4.3 (CHCl$_3$, c = 1.0).

4. Synthetic procedures and characterisation for hydrazone starting materials

General procedure for aldehyde-derived hydrazone formation:[35] To a solution of aldehyde (20.0 mmol, 1.0 equiv.) in methanol (20 mL) was added hydrazine hydrate (1.2 mL, 24.0 mmol, 1.2 equiv.) and the mixture stirred at r.t. for 1 h. The mixture was then evaporated under reduced pressure to provide the desired hydrazone. The crude hydrazone was used for generation of the corresponding diazo compound without further purification.

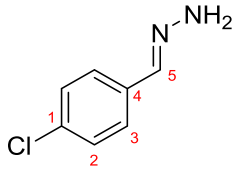

(4-chlorobenzylidene)hydrazine (1a): Following the general procedure for aldehyde-derived hydrazone formation using 4-chlorobenzaldehyde (2.81 g, 20.0 mmol), provided the title product as a white crystalline solid (3.08 g, 19.9 mmol, 99%), m.p. 59-61 °C (lit. m.p.[36] 60-61 °C). Data are consistent with a reported example.[37]

^{1}H NMR (600 MHz, CDCl$_3$): δ 7.69 (s, 1 H, H5), 7.47 (d, *J* = 8.5 Hz, 2 H, H3), 7.31 (d, *J* = 8.5 Hz, 2 H, H2), 5.54 (br s, 2 H, NH).
^{13}C NMR (150 MHz, CDCl$_3$): δ 141.8 (C5), 134.4 (C4), 133.7 (C1), 128.9 (C2), 127.4 (C3).
FTIR (ν_{max}, cm^{-1}): 3357 (w), 3186 (w), 1625 (w), 1594 (w), 1486 (w), 1393 (w), 1249 (w), 1218 (w), 1109 (w), 1090 (m), 1010 (w), 961 (w), 924 (w), 912 (w), 864 (w), 824 (s).
HRMS (ESI): calculated for $C_7H_7N_2ClNa$ $[M+Na]^+$ 177.0190, found 177.0188.

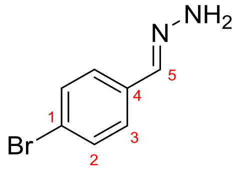

(4-bromobenzylidene)hydrazine: Following the general procedure for aldehyde-derived hydrazone formation using 4-bromobenzaldehyde (3.70 g, 20.0 mmol), provided the title product as an off-white crystalline solid (3.98 g, 19.9 mmol, 99%), m.p. 73-75 °C (lit. m.p.[36] 77-78 °C). Data are consistent with a reported example.[36]

^{1}H NMR (600 MHz, CDCl$_3$): δ 7.67 (s, 1 H, H5), 7.47 (d, *J* = 8.5 Hz, 2 H, H2), 7.41 (d, *J* = 8.5 Hz, 2 H, H3), 5.56 (br s, 2 H, NH).
^{13}C NMR (150 MHz, CDCl$_3$): δ 141.7 (C5), 134.3 (C4), 131.9 (C2), 127.7 (C3), 122.6 (C1).
FTIR (ν_{max}, cm^{-1}): 3353 (w), 3194 (w), 1627 (w), 1586 (m), 1483 (w), 1391 (m), 1069 (s), 1006 (m), 924 (m), 912 (m), 862 (m), 818 (s).
HRMS (ESI): calculated for $C_7H_7N_2BrNa$ $[M+Na]^+$ 220.9685, found 220.9690.

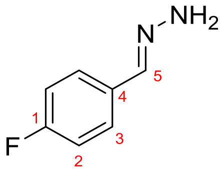

(4-fluorobenzylidene)hydrazine: Following the general procedure for aldehyde-derived hydrazone formation using 4-fluorobenzaldehyde (2.48 g, 20.0 mmol), provided the title product as a colourless oil (2.75 g, 19.9 mmol, 99%).

^{1}H NMR (600 MHz, $CDCl_3$): δ 7.72 (s, 1 H, H5), 7.52 (dd, *J* = 8.7, 5.5 Hz, 2 H, H3), 7.04 (t, *J* = 8.7 Hz, 2 H, H4), 5.47 (br s, 2 H, NH).
^{13}C NMR (150 MHz, $CDCl_3$): δ 163.1 (d, *J* = 248.2 Hz, C1), 142.0 (C5), 131.5 (d, *J* = 3.2 Hz, C4), 127.9 (d, *J* = 8.2 Hz, C3), 115.7 (d, *J* = 21.9 Hz, C2).
^{19}F NMR (376 MHz, $CDCl_3$): δ -112.6 (s, 1 F, F1).
FTIR (ν_{max}, cm^{-1}): 3379 (w), 3192 (w), 2905 (w), 1604 (m), 1508 (s), 1398 (w), 1300 (w), 1228 (s), 1154 (m), 1080 (w), 1012 (w), 947 (w), 919 (w), 875 (w), 832 (s), 797 (m).
HRMS (ESI): calculated for $C_7H_7FN_2Na$ $[M+Na]^+$ 161.0485, found 161.0484.

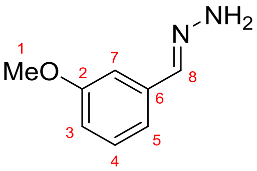

(3-methoxybenzylidene)hydrazine: Following the general procedure for aldehyde-derived hydrazone formation using *m*-anisaldehyde (2.72 g, 20.0 mmol), provided the title product as a yellow oil (2.99 g, 19.9 mmol, 99%). Data is consistent with a reported example.[35]

^{1}H NMR (600 MHz, $CDCl_3$): δ 7.72 (s, 1 H, H8), 7.26 (t, *J* = 7.9 Hz, 1 H, H4), 7.16 (dd, *J* = 2.5, 0.8 Hz, 1 H, H7), 7.07 (dt, *J* = 7.9, 0.8 Hz, 1 H, H5), 6.86 (ddd, *J* = 7.9, 2.5, 0.8 Hz, 1 H, H3), 5.51 (br s, 2 H, NH), 3.83 (s, 3 H, H1).
^{13}C NMR (150 MHz, $CDCl_3$): δ 160.0 (C2), 143.1 (C8), 136.7 (C6), 129.7 (C4), 119.5 (C5), 115.4 (C3), 110.2 (C7), 55.4 (C1).
FTIR (ν_{max}, cm^{-1}): 3387 (w), 3200 (w), 2911 (w), 2835 (w), 1597 (s), 1575 (s), 1489 (m), 1466 (m), 1455 (m), 1431 (m), 1397 (w), 1317 (w), 1288 (m), 1263 (s), 1195 (w), 1155 (s), 1072 (w), 1038 (s), 994 (w), 967 (w), 922 (m), 862 (w).
HRMS (ESI): calculated for $C_8H_{10}N_2ONa$ $[M+Na]^+$ 173.0685, found 173.0692.

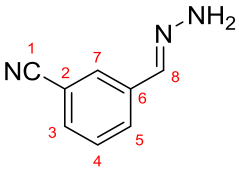

3-(hydrazonomethyl)benzonitrile: Following the general procedure for aldehyde-derived hydrazone formation using 3-formylbenzonitrile (2.62 g, 20.0 mmol), provided the title product as a pale yellow amorphous solid (2.90 g, 19.9 mmol, 99%), m.p. 74-77 °C (lit. m.p.[36] 83-84°C). Data are consistent with a reported example.[36]

^{1}H NMR (600 MHz, $CDCl_3$): δ 7.81 (t, *J* = 1.3 Hz, 1 H, H7), 7.74 (dt, *J* = 7.8 Hz, 1.3 Hz, 1 H, H5), 7.68 (s, 1 H, H8), 7.54 (dt, *J* = 7.8, 1.3 Hz, 1 H, H3), 7.43 (t, *J* = 7.8 Hz, 1 H, H4), 5.73 (br s, 2 H, NH).

^{13}C NMR (150 MHz, CDCl$_3$): δ 139.6 (C8), 136.7 (C6), 131.6 (C3), 130.1 (C5), 129.55 (C7), 129.47 (C4), 118.8 (C1), 112.9 (C2).
FTIR (ν_{max}, cm^{-1}): 3402 (m), 3292 (w), 3215 (w), 2917 (w), 2231 (s), 1590 (m), 1573 (m), 1481 (w), 1434 (w), 1388 (w), 1284 (w), 1245 (w), 1157 (w), 1088 (w), 918 (m), 798 (s).
HRMS (ESI): calculated for $C_8H_7N_3Na$ $[M+Na]^+$ 168.0532, found 168.0535.

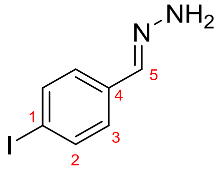

(4-iodobenzylidene)hydrazine: Following the general procedure for aldehyde-derived hydrazone formation using 4-iodobenzaldehyde (9.28 g, 40.0 mmol), provided the title product as a pale yellow amorphous solid (9.82 g, 39.9 mmol, 99%), m.p. 96-98 °C.

^{1}H NMR (600 MHz, CDCl$_3$): δ 7.67 (d, J = 8.4 Hz, 2 H, H2), 7.65 (s, 1 H, H5), 7.27 (d, J = 8.4 Hz, 2 H, H3), 5.57 (br s, 2 H, NH).
^{13}C NMR (150 MHz, CDCl$_3$): δ 141.8 (C5), 137.8 (C2), 134.8 (C4), 127.9 (C3), 94.3 (C1).
FTIR (ν_{max}, cm^{-1}): 3360 (m), 3196 (w), 2912 (w), 1594 (w), 1483 (w), 1389 (m), 1080 (w), 1059 (w), 1004 (w), 925 (w), 912 (w), 863 (w), 818 (s).
HRMS (ESI): calculated for $C_7H_7N_2INa$ $[M+Na]^+$ 268.9546, found 268.9555.

5. Optimisation and discussion

5.1. Ligand list

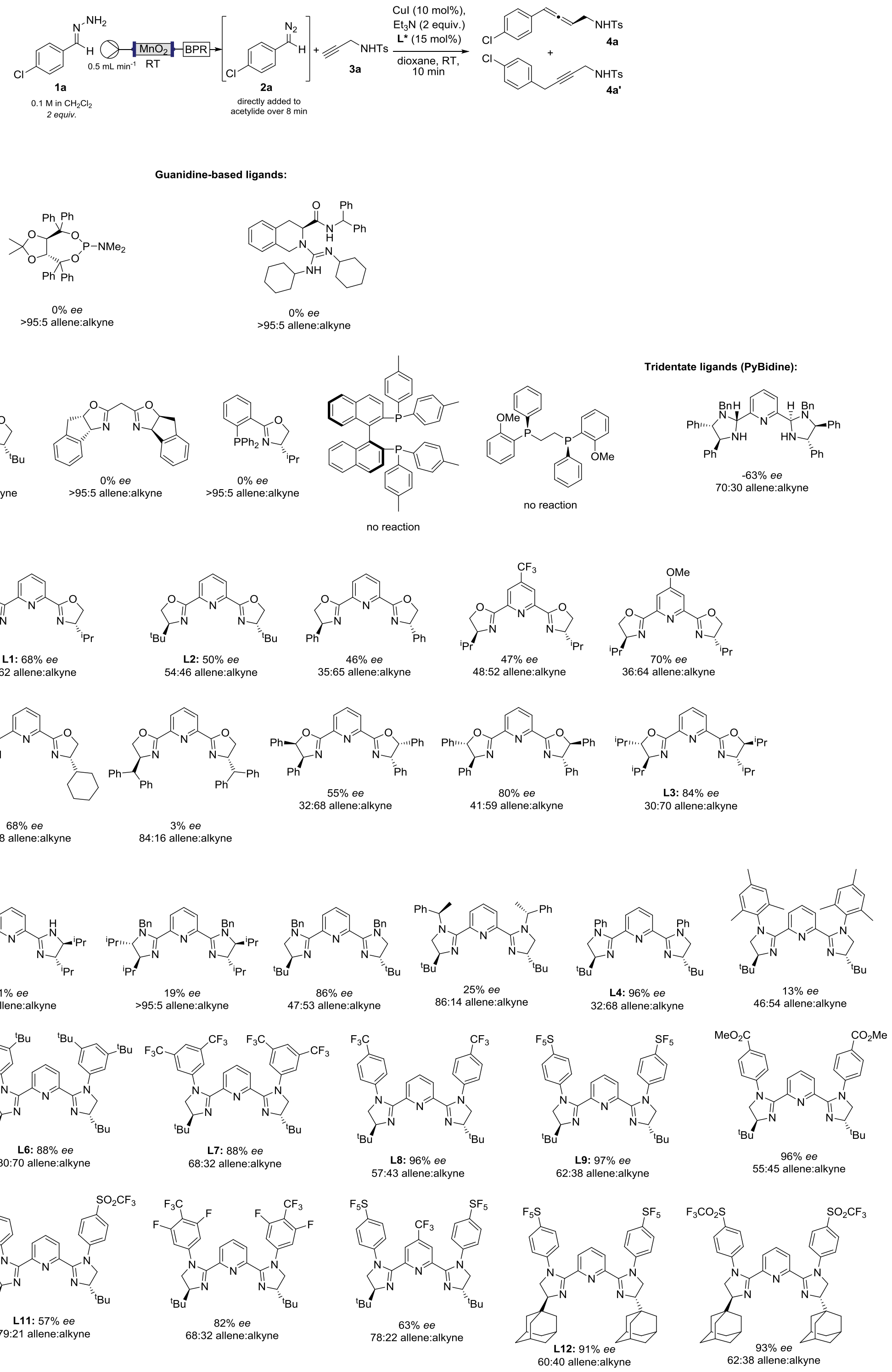

Scheme S1: List of ligands tested for the asymmetric allenylation of alkyne **3a**.

The absence of any enantioselectivity for the bidentate ligands, particularly the two BOX ligands tested, appears to imply that the use of *tridentate* ligands are essential for asymmetric *disubstituted* allene synthesis (Scheme S1). It should be noted that during our tests with these tridentate ligands for the allenylation of ketone-derived diazo compounds and terminal alkynes, no enantioselectivity was observed. In contrast, Wang *et al*. describe the use of *bidentate* BOX ligands for *trisubstituted* allene synthesis. Taken together, these results seem to suggest that these ligand systems are complementary to each other – with *tridentate* ligands allowing asymmetric induction for *disubstituted* allenes and *bidentate* ligands allowing asymmetric induction for *trisubstituted* allenes.

For the PyBOX ligands, enantioselectivities were best when the 4-position group on the oxazoline was a secondary alkyl group (68% *ee* with ^{i}Pr and cyclohexyl). A primary alkyl group does not provide enough steric bulk (45% *ee* with Me), whereas a tertiary alkyl group (50% *ee* with ^{t}Bu, **L2**) perhaps diverts the binding mode of the oxazoline units to species that are poorer at inducing enantioselectivity, by partially favouring coordination to oxygen rather than nitrogen (Scheme S2a).

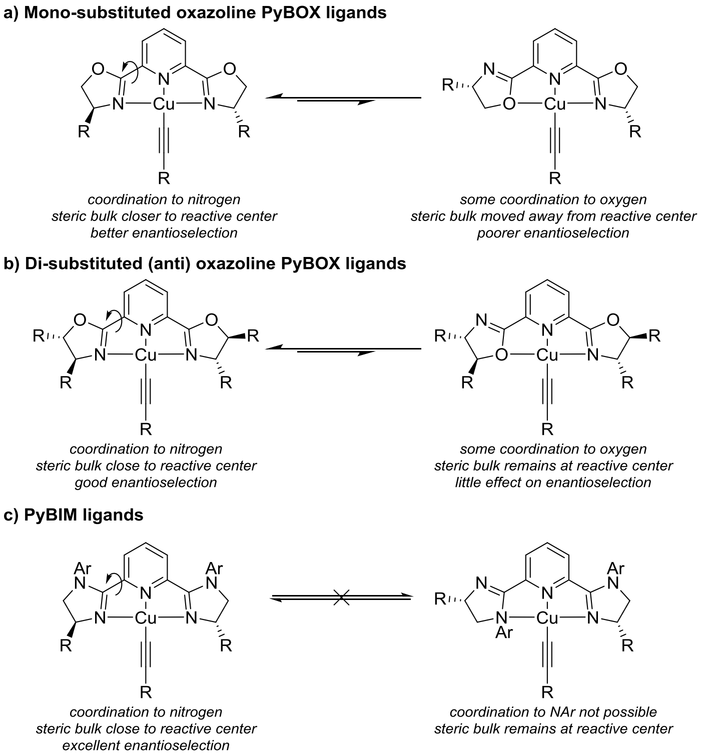

Scheme S2: Proposed rationale for trends in enantiocontrol for PyBOX and PyBIM ligands.

Comparison of the (*S*)-Ph-PyBOX result (46% *ee*) with the (*S*,*S*)-diPh-PyBOX result (80% *ee*) lends further evidence for this hypothesis, since coordination to the oxygen atom for the latter case maintains steric bulk close to the reactive center (Scheme S2b). A similar trend is observed when comparing (*S*)-^{i}Pr-PyBOX (**L1**, 68% *ee*) to (*S*,*S*)-di^{i}Pr-PyBOX (**L3**, 84% *ee*).

For the PyBIM ligands, a tertiary alkyl group (86% *ee* for (*S*)-^{t}Bu-NBn-PyBIM) was better than a secondary alkyl group (67% *ee* for (*S*)-^{i}Pr-NBn-PyBIM) for inducing enantioselection, contrasting with the trend seen with the PyBOX ligands. Attempting to install ^{i}Pr groups in a similar fashion to that of (*S*,*S*)-diPh-PyBOX only causes erosion in enantioselectivity (19% *ee*), suggesting that the imidazoline units do not act as ambidentate ligands as proposed for the oxazoline units of PyBOX ligands (Scheme S2c).

5.2. Optimisation table

Table S1: Optimisation table for asymmetric allenylation[a]

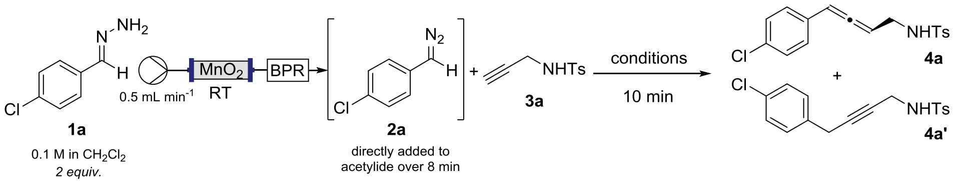

entry	L*	solvent	metal source	base	temp.	other conditions	allene/alkyne[b]	*ee* / % [c]
1	**L1**	dioxane	CuI	Et_3N	RT	-	38:62	68
2	**L1**	1,2-DME	CuI	Et_3N	RT	-	50:50	45
3	**L1**	DMSO	CuI	Et_3N	RT	-	>95:5	0
4	**L1**	CH_2Cl_2	CuI	Et_3N	RT	-	48:52	43
5	**L1**	THF	CuI	Et_3N	RT	-	42:58	48
6	**L1**	MeCN	CuI	Et_3N	RT	-	81:19	0
7	**L1**	EtOAc	CuI	Et_3N	RT	-	38:62	50
8	**L1**	DMF	CuI	Et_3N	RT	-	81:19	0
9	**L1**	dioxane	$Cu(MeCN)_4PF_6$	Et_3N	RT	-	(no conv.)	(no conv.)
10	**L1**	dioxane	AuI	Et_3N	RT	-	(no conv.)	(no conv.)
11	**L1**	dioxane	ZnI_2	Et_3N	RT	-	(no conv.)	(no conv.)
12	**L1**	dioxane	CuI	Et_3N	RT	initial acetylide solution diluted to 0.025 M	48:52	40
13	**L1**	dioxane	CuI	Et_3N	RT	2 equiv. $Et_3NH^+I^-$ added	88:12	7
14	**L1**	dioxane	CuI	Et_3N	RT	2 equiv. MeOH added	40:60	64
15	**L1**	dioxane	CuI	Et_3N	RT	4Å MS added	38:62	66
16	**L1**	dioxane	CuI	DIPEA	RT	-	38:62	62
17	**L1**	dioxane	CuI	TMEDA	RT	-	>95:5	0
18	**L1**	dioxane	CuI	Quinine	RT	-	>95:5	8
19	**L1**	dioxane	CuI	Cs_2CO_3	RT	-	20:80	(n.d.)
20	**L11**	dioxane	CuI	Et_3N	RT	-	79:21	57
21	**L11**	dioxane/CH_2Cl_2	CuI	Et_3N	0 °C	(4:1 dioxane/CH_2Cl_2 to prevent freezing)	68:32	71
22	**L9**	dioxane	CuI	Et_3N	RT	-	62:38	97
23	**L9**	dioxane	CuI	DIPEA	RT	-	52:48	94
24	**L9**	dioxane	CuI	Et_3N	RT	using 5 mol% CuI, 7.5 mol% ligand	68:32	90

[a] Standard conditions: Using 0.2 mmol of alkyne **3a**, 0.02 mmol of metal source, 0.03 mmol of ligand and 0.4 mmol of base at specified temperature in 2 mL solvent, with 0.4 mmol of hydrazone **1a** (with DIPEA as buffer) flowed through activated MnO_2. [b] Allene/alkyne ratio determined by ^1H NMR analysis of the crude reaction mixture. [c] *ee* determined by chiral HPLC.

6. Synthetic procedures and characterisation for asymmetric allenylation

Stock solution of L9-CuI: To a mixture of 2,6-bis((*S*)-4-(*tert*-butyl)-1-(4-(pentafluorosulfanyl)phenyl)-4,5-dihydro-1*H*-imidazol-2-yl)pyridine (**L9**) (0.549 g, 0.75 mmol) and copper(I) iodide (95.2 mg, 0.50 mmol) was added 1,4-dioxane (50 mL) and Et_3N (1.39 mL, 10.0 mmol). The mixture was stirred at r.t. for 2 h, resulting in a brown-red solution of the ligand/copper complex. The solution was stored at -20 °C under Ar as a precaution.

General procedure for racemic allenylation:[35]

Conditioning phase: A solution of hydrazone (0.1 M) and DIPEA (0.2 M) in CH_2Cl_2 was passed through a column reactor (Omnifit® column, 6.6 mm i.d. × 50 mm length), packed with activated MnO_2 (0.86 g), at a flow rate of 0.5 mL min^{-1} for 20 min and the reactor output was monitored using a FlowIR® device (SiComp head, 2100-2000 cm^{-1}).. The flow was switched to solvent (DIPEA, 0.2 M in CH_2Cl_2) for 10 min. The column was then ready for the generation of the diazo compound.

Generation phase: A 5 mL microwave vial was charged with the appropriate alkyne (0.2 mmol, 1.0 equiv.), copper (I) iodide (3.9 mg, 0.02 mmol, 0.1 equiv.), 1,4-dioxane (2 mL) and Et_3N (0.05 mL, 0.4 mmol, 2 equiv.) and pre-mixed for 10 min. A solution of hydrazone (0.1 M) and DIPEA (0.2 M) in CH_2Cl_2 was passed through the pre-conditioned column reactor (Omnifit® column, 6.6 mm i.d. × 50 mm length), packed with activated MnO_2 (0.86 g), at a flow rate of 0.5 mL min^{-1}. When the FlowIR® showed that the intensity of the diazo peak was stable, 3 mL of the output (1.5 equiv. with respect to the hydrazone) was directly added into the reaction vial (over 6 min) containing the copper acetylide and the reaction mixture further stirred at r.t. for 10 min. The mixture was then filtered through a pad of Celite, eluting with Et_2O, and the filtrate evaporated under reduced pressure. The residue was immediately purified by silica gel column chromatography to provide the desired di-substituted allene product.

Any excess diazo compound produced during the conditioning phase or the generation phase before steady-state was reached was gently quenched by directing the output of the flow reactor into a stirred suspension of copper (I) iodide (0.10 g) in MeOH (25 mL).

General procedure for asymmetric allenylation:

Conditioning phase: A solution of hydrazone (0.1 M) and DIPEA (0.2 M) in CH_2Cl_2 was passed through a column reactor (Omnifit® column, 6.6 mm i.d. × 50 mm length), packed with activated MnO_2 (0.86 g), at a flow rate of 0.5 mL min^{-1} for 20 min and the reactor output was monitored using a FlowIR® device (SiComp head, 2100-2000 cm^{-1}). The flow was switched to solvent (DIPEA, 0.2 M in CH_2Cl_2) for 10 min. The column was then ready for the generation of the diazo compound.

Generation phase: To a 5 mL microwave vial was added the appropriate alkyne (0.2 mmol, 1.0 equiv.), then an aliquot of the vigorously stirred **L9**-CuI stock solution (2.05 mL, containing 0.15 equiv. ligand, 0.1 equiv. CuI and 2 equiv. Et_3N) was added. The mixture was pre-mixed at r.t. for 10 min, forming a clear red-orange homogeneous solution of the copper acetylide-ligand complex.

A solution of hydrazone (0.1 M) and DIPEA (0.2 M) in CH_2Cl_2 was passed through the pre-conditioned column reactor (Omnifit® column, 6.6 mm i.d. × 50 mm length), packed with activated MnO_2 (0.86 g), at a flow rate of 0.5 mL min^{-1}. When the FlowIR® showed that the intensity of the diazo peak was stable, 4 mL of the output (2.0 equiv. with respect to the hydrazone) was directly added into the reaction vial (over 8 min) containing the copper acetylide-ligand complex and the reaction mixture further stirred at r.t. for 10 min. The solution was evaporated under reduced pressure and the residue taken up in Et_2O (5 mL). The mixture was then filtered through a pad of Celite, eluting with Et_2O, and the filtrate evaporated under reduced pressure. The residue was immediately purified by silica gel column chromatography to provide the desired allene (and alkyne) cross-products.

Any excess diazo compound produced during the conditioning phase or the generation phase before steady-state was reached was gently quenched by directing the output of the flow reactor into a stirred suspension of copper (I) iodide (0.10 g) in MeOH (25 mL).

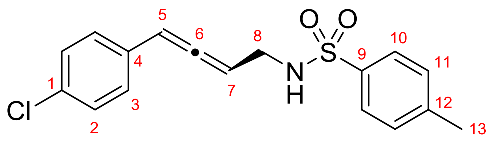

(*R*)-*N*-(4-(4-chlorophenyl)buta-2,3-dien-1-yl)-4-methylbenzenesulfonamide (4a): Following the general procedure for asymmetric allenylation using 4-methyl-*N*-(prop-2-yn-1-yl)benzenesulfonamide (41.8 mg, 0.2 mmol) and (4-chlorobenzylidene)hydrazine, purified by silica gel column chromatography (eluent: 1% Et_2O/CH_2Cl_2) provided the title compound as an off-white amorphous solid (33.7 mg, 0.101 mmol, 51%, 97% *ee*), m.p. 86-88 °C. Data are consistent with a reported racemic example.[35]

^{1}H NMR (600 MHz, $CDCl_3$): δ 7.74 (d, *J* = 8.2 Hz, 2 H, H10), 7.27 (d, *J* = 8.2 Hz, 2 H, H11), 7.24 (d, *J* = 8.4 Hz, 2 H, H2), 7.12 (d, *J* = 8.4 Hz, 2 H, H3), 6.17 (dt, *J* = 6.2, 3.0 Hz, 1 H, H5), 5.55 (q, *J* = 6.2 Hz, 1 H, H7), 4.68 (br s, 1 H, NH), 3.73 – 3.68 (m, 2 H, H8), 2.42 (s, 3 H, H13).
^{13}C NMR (150 MHz, $CDCl_3$): δ 204.8 (C6), 143.8 (C12), 137.0 (C9), 133.3 (C1), 131.9 (C4), 129.9 (C11), 129.0 (C2), 128.2 (C3), 127.3 (C10), 97.1 (C5), 92.6 (C7), 41.7 (C8), 21.7 (C13).
FTIR (ν_{max}, cm^{-1}): 3276 (w), 2924 (w), 1957 (w), 1723 (w), 1597 (w), 1491 (m), 1408 (w), 1323 (m), 1265 (w), 1155 (s), 1090 (s), 1013 (m), 874 (m), 834 (m), 812 (s).
HRMS (ESI): calculated for $C_{17}H_{15}NO_2SCl$ [M-H]$^-$ 332.0518, found 332.0517.
R$_f$ = 0.56 (1% Et_2O/CH_2Cl_2).
$[\alpha]_D^{25.0}$ = -205.7 ($CHCl_3$, c = 1.0, 97% *ee*).
HPLC: Chiralpak AS, 90:10 hexane/isopropanol, 1.0 mL/min flow rate, T = 25 °C, λ_{max} = 254 nm; t_R (min) = 58.4 (minor), 75.5 (major).

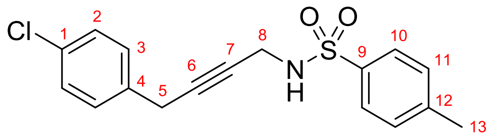

***N*-(4-(4-chlorophenyl)but-2-yn-1-yl)-4-methylbenzenesulfonamide (4a'):** Isolated as the alkyne cross-product from asymmetric allenylation of 4-methyl-*N*-(prop-2-yn-1-yl)benzenesulfonamide and (4-chlorobenzylidene)hydrazine, which provided the title compound as an off-white amorphous solid (18.8 mg, 0.056 mmol, 28%), m.p. 81-83 °C.

^{1}H NMR (600 MHz, $CDCl_3$): δ 7.76 (d, *J* = 8.3 Hz, 2 H, H10), 7.26 – 7.22 (m, 4 H, H2 and H11), 7.07 (d, *J* = 8.4 Hz, 2 H, H3), 4.63 (br s, 1 H, NH), 3.88 (dt, *J* = 5.9, 2.2 Hz, 2 H, H8), 3.35 (t, *J* = 2.2 Hz, 2 H, H5), 2.38 (s, 3 H, H13).
^{13}C NMR (150 MHz, $CDCl_3$): δ 143.8 (C12), 136.9 (C9), 134.6 (C4), 132.7 (C1), 129.7 (C11), 129.2 (C3), 128.7 (C2), 127.5 (C10), 82.5 (C6), 76.8 (C7), 33.5 (C8), 24.4 (C5), 21.7 (C13).
FTIR (ν_{max}, cm^{-1}): 3268 (w), 3033 (w), 2925 (w), 1723 (w), 1597 (w), 1491 (m), 1408 (m), 1325 (m), 1306 (m), 1291 (m), 1215 (w), 1120 (w), 1186 (m), 1156 (s), 1090 (s), 1058 (m), 1015 (m), 812 (s), 751 (s).
HRMS (ESI): calculated for $C_{17}H_{16}NO_2SClNa$ [M+Na]$^+$ 356.0482, found 356.0486.
R$_f$ = 0.48 (1% Et_2O/CH_2Cl_2).

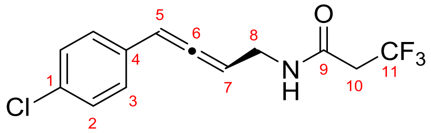

(*R*)-*N*-(4-(4-chlorophenyl)buta-2,3-dien-1-yl)-3,3,3-trifluoropropanamide (4b): Following the general procedure for asymmetric allenylation using 3,3,3-trifluoro-*N*-(prop-2-yn-1-yl)propanamide (33.0 mg, 0.2 mmol) and (4-chlorobenzylidene)hydrazine, purified by silica gel column chromatography (eluent: 25% EtOAc/hexane) provided the title compound as a yellow amorphous solid (29.9 mg, 0.103 mmol, 52%, 91% *ee*), m.p. 86-87 °C.

^{1}H NMR (600 MHz, $CDCl_3$): δ 7.27 (d, *J* = 8.4 Hz, 2 H, H2), 7.19 (d, *J* = 8.4 Hz, 2 H, H3), 6.28 (dt, *J* = 6.4, 3.2 Hz, 1 H, H5), 6.05 (br s, 1 H, NH), 5.67 (q, *J* = 6.4 Hz, 1 H, H7), 4.09 – 3.91 (m, 2 H, H8), 3.04 (q, *J* = 10.6 Hz, 2 H, H10).
^{13}C NMR (150 MHz, $CDCl_3$): δ 204.5 (C6), 162.6 (q, *J* = 3.5 Hz, C9), 133.2 (C1), 132.1 (C4), 129.0 (C2), 128.2 (C3), 124.0 (q, *J* = 276.7 Hz, C11), 97.3 (C5), 92.8 (C7), 41.7 (q, *J* = 29.6 Hz, C10), 38.2 (C8).
^{19}F NMR (376 MHz, $CDCl_3$): δ -63.0 (s, 3 F, F11).
FTIR (ν_{max}, cm^{-1}): 3309 (m), 2921 (w), 1953 (w), 1651 (s), 1556 (m), 1492 (m), 1390 (m), 1341 (w), 1264 (m), 1235 (m), 1141 (m), 1107 (m), 1093 (m), 1063 (w), 1014 (w), 920 (w), 880 (w), 851 (w), 836 (m), 752 (w).
HRMS (ESI): calculated for $C_{13}H_{10}F_3NOCl$ $[M-H]^-$ 288.0408, found 288.0407.
R_f = 0.23 (25% EtOAc/hexane).
$[\alpha]_D^{27.0}$ = -160.5 ($CHCl_3$, c = 1.0, 91% *ee*).
HPLC: ChiralART SC, 95:5 hexane/isopropanol, 1.0 mL/min flow rate, T = 25 °C, λ_{max} = 254 nm; t_R (min) = 15.2 (minor), 16.6 (major).

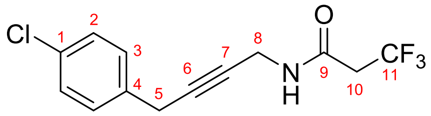

***N*-(4-(4-chlorophenyl)but-2-yn-1-yl)-3,3,3-trifluoropropanamide (4b'):** Isolated as the alkyne cross-product from asymmetric allenylation of 3,3,3-trifluoro-*N*-(prop-2-yn-1-yl)propanamide and (4-chlorobenzylidene)hydrazine, which provided the title compound as an off-white amorphous solid (14.8 mg, 0.051 mmol, 26%), m.p. 96-99 °C.

^{1}H NMR (600 MHz, $CDCl_3$): δ 7.28 (d, *J* = 8.4 Hz, 2 H, H2), 7.23 (d, *J* = 8.4 Hz, 2 H, H3), 5.98 (br s, 1 H, NH), 4.12 (dt, *J* = 4.8, 2.3 Hz, 2 H, H8), 3.56 (t, *J* = 2.3 Hz, 2 H, H5), 3.08 (q, *J* = 10.5 Hz, 2 H, H10).
^{13}C NMR (150 MHz, $CDCl_3$): δ 162.2 (q, *J* = 3.6 Hz, C9), 134.8 (C4), 132.7 (C1), 129.3 (C3), 128.8 (C2), 124.0 (q, *J* = 276.7 Hz, C11), 81.8 (C6), 77.3 (C7), 41.7 (q, *J* = 29.8 Hz, C10), 30.3 (C8), 24.6 (C5).
^{19}F NMR (376 MHz, $CDCl_3$): δ -62.9 (s, 3 F, F11).
FTIR (ν_{max}, cm^{-1}): 3304 (m), 2970 (w), 2903 (w), 1651 (s), 1554 (m), 1492 (w), 1452 (w), 1424 (w), 1393 (m), 1351 (m), 1304 (w), 1264 (m), 1236 (m), 1129 (m), 1101 (m), 1092 (m), 1057 (m), 1016 (w), 927 (w), 854 (w), 841 (w), 796 (w), 760 (w).
HRMS (ESI): calculated for $C_{13}H_{11}F_3NOClNa$ $[M+Na]^+$ 312.0373, found 312.0375.
R_f = 0.28 (25% EtOAc/hexane).

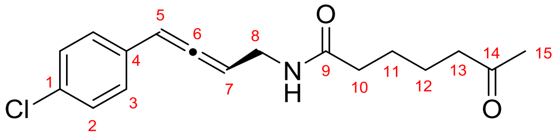

(*R*)-*N*-(4-(4-chlorophenyl)buta-2,3-dien-1-yl)-6-oxoheptanamide (4c): Following the general procedure for asymmetric allenylation using 6-oxo-*N*-(prop-2-yn-1-yl)heptanamide (36.2 mg, 0.2 mmol) and (4-chlorobenzylidene)hydrazine, purified by silica gel column chromatography (eluent: 30% Et_2O/CH_2Cl_2) provided the title compound as a white crystalline solid (30.0 mg, 0.098 mmol, 49%, 96% *ee*), m.p. 94-95 °C.

^{1}H NMR (600 MHz, $CDCl_3$): δ 7.26 (d, *J* = 8.5 Hz, 2 H, H2), 7.20 (d, *J* = 8.5 Hz, 2 H, H3), 6.25 (dt, *J* = 6.5, 3.3 Hz, 1 H, H5), 5.85 (br s, 1 H, NH), 5.67 (q, *J* = 6.5 Hz, 1 H, H7), 4.05 – 3.99 (m, 1 H, H8a), 3.96 – 3.90 (m, 1 H, H8b), 2.39 – 2.34 (m, 2 H, H13), 2.18 – 2.14 (m, 2 H, H10), 2.10 (s, 3 H, H15), 1.56 – 1.49 (m, 4 H, H11 and H12).
^{13}C NMR (150 MHz, $CDCl_3$): δ 208.9 (C14), 204.6 (C6), 172.6 (C9), 133.0 (C1), 132.5 (C4), 128.9 (C2), 128.2 (C3), 96.9 (C5), 93.8 (C7), 43.3 (C13), 37.7 (C8), 36.4 (C10), 30.1 (C15), 25.1 (C11/C12), 23.2 (C11/C12).
FTIR (ν_{max}, cm^{-1}): 3312 (m), 3062 (w), 2943 (w), 1950 (w), 1708 (s), 1640 (s), 1538 (s), 1492 (s), 1461 (w), 1422 (w), 1361 (w), 1263 (w), 1236 (w), 1161 (w), 1092 (m), 1014 (w), 876 (w), 835 (m), 812 (w).
HRMS (ESI): calculated for $C_{17}H_{20}NO_2ClNa$ $[M+Na]^+$ 328.1075, found 328.1080.
R_f = 0.28 (30% Et_2O/CH_2Cl_2).
$[\alpha]_D^{25.0}$ = -210.9 ($CHCl_3$, c = 1.0, 96% *ee*).
HPLC: ChiralART SC, 85:15 hexane/isopropanol, 1.0 mL/min flow rate, T = 25 °C, λ_{max} = 254 nm; t_R (min) = 33.8 (minor), 38.8 (major).

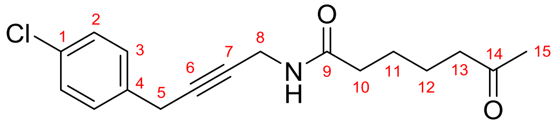

***N*-(4-(4-chlorophenyl)but-2-yn-1-yl)-6-oxoheptanamide (4c'):** Isolated as the alkyne cross-product from asymmetric allenylation of 6-oxo-*N*-(prop-2-yn-1-yl)heptanamide and (4-chlorobenzylidene)hydrazine, which provided the title compound as a white amorphous solid (23.3 mg, 0.076 mmol, 38%), m.p. 79-80 °C.

^{1}H NMR (600 MHz, $CDCl_3$): δ 7.27 (d, *J* = 8.6 Hz, 2 H, H2), 7.23 (d, *J* = 8.6 Hz, 2 H, H3), 5.88 (br s, 1 H, NH), 4.07 (dt, *J* = 4.9, 2.2 Hz, 2 H, H8), 3.54 (t, *J* = 2.2 Hz, 2 H, H5), 2.44 (t, *J* = 6.8 Hz, 2 H, H13), 2.19 (t, *J* = 7.1 Hz, 2 H, H10), 2.12 (s, 3 H, H15), 1.65 – 1.54 (m, 4 H, H11 and H12).
^{13}C NMR (150 MHz, $CDCl_3$): δ 209.0 (C14), 172.3 (C9), 135.1 (C4), 132.6 (C1), 129.4 (C3), 128.7 (C2), 80.8 (C6), 78.3 (C7), 43.4 (C13), 36.2 (C10), 30.1 (C15), 29.7 (C8), 25.0 (C11/C12), 24.6 (C5), 23.2 (C11/C12).
FTIR (ν_{max}, cm^{-1}): 3287 (m), 3056 (w), 2933 (w), 2866 (w), 1702 (s), 1635 (s), 1542 (m), 1492 (m), 1463 (w), 1419 (w), 1377 (w), 1360 (w), 1265 (w), 1237 (w), 1167 (w), 1097 (w), 1017 (w), 814 (w), 792 (w).
HRMS (ESI): calculated for $C_{17}H_{19}NO_2Cl$ $[M-H]^-$ 304.1110, found 304.1110.
R_f = 0.38 (30% Et_2O/CH_2Cl_2).

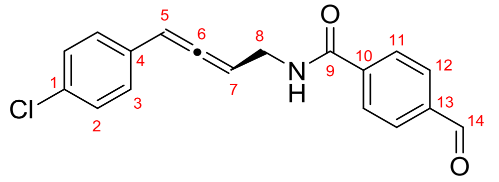

(R)-N-(4-(4-chlorophenyl)buta-2,3-dien-1-yl)-4-formylbenzamide (4d): Following the general procedure for asymmetric allenylation using 4-formyl-*N*-(prop-2-yn-1-yl)benzamide (37.4 mg, 0.2 mmol) and (4-chlorobenzylidene)hydrazine, purified by silica gel column chromatography (eluent: 5% Et_2O/CH_2Cl_2) provided the title compound as a pale yellow crystalline solid (29.2 mg, 0.094 mmol, 47%, 97% *ee*), m.p. 128-130 °C.

^{1}H NMR (600 MHz, $CDCl_3$): δ 10.05 (s, 1 H, H14), 7.90 (d, J = 8.2 Hz, 2 H, H12), 7.83 (d, J = 8.2 Hz, 2 H, H11), 7.27 (d, J = 8.5 Hz, 2 H, H2), 7.22 (d, J = 8.5 Hz, 2 H, H3), 6.40 (br s, 1 H, NH), 6.30 (dt, J = 6.4, 3.3 Hz, 1 H, H5), 5.79 (q, J = 6.4 Hz, 1 H, H7), 4.26 – 4.13 (m, 2 H, H8).
^{13}C NMR (150 MHz, $CDCl_3$): δ 204.8 – 204.7 (br, C6), 191.6 (C14), 166.5 (C9), 139.6 (C10), 138.4 (C13), 133.3 (C1), 132.2 (C4), 130.0 (C12), 129.1 (C2), 128.2 (C3), 127.7 (C11), 97.3 (C5), 93.3 (C7), 38.4 (C8).
FTIR (ν_{max}, cm^{-1}): 3325 (w), 3060 (w), 2838 (w), 1955 (w), 1705 (s), 1645 (s), 1610 (w), 1572 (w), 1537 (s), 1491 (s), 1430 (w), 1389 (w), 1346 (w), 1294 (m), 1207 (m), 1149 (w), 1090 (m), 1014 (w), 876 (w), 835 (m), 759 (w).
HRMS (ESI): calculated for $C_{18}H_{14}NO_2ClNa$ $[M+Na]^+$ 334.0605, found 334.0610.
R_f = 0.29 (5% Et_2O/CH_2Cl_2).
$[\alpha]_D^{25.0}$ = -473.6 ($CHCl_3$, c = 1.0, 97% *ee*).
HPLC: ChiralART SA, 93:7 hexane/isopropanol, 1.0 mL/min flow rate, T = 25 °C, λ_{max} = 254 nm; t_R (min) = 43.3 (minor), 45.0 (major).

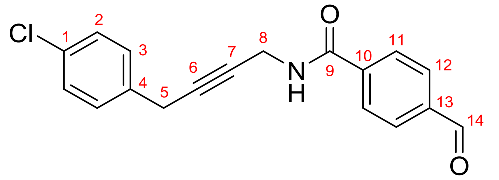

N-(4-(4-chlorophenyl)but-2-yn-1-yl)-4-formylbenzamide (4d'): Isolated as the alkyne cross-product from asymmetric allenylation of 4-formyl-*N*-(prop-2-yn-1-yl)benzamide and (4-chlorobenzylidene)hydrazine, after repurification by silica gel column chromatography (eluent: 40% EtOAc/hexane) of the alkyne-containing fractions, which provided the title compound as a white amorphous solid (20.8 mg, 0.067 mmol, 33%), m.p. 117-120 °C.

^{1}H NMR (600 MHz, $CDCl_3$): δ 10.07 (s, 1 H, H14), 7.96 – 7.92 (m, 4 H, H11 and H12), 7.28 (d, J = 8.6 Hz, 2 H, H2), 7.25 (d, J = 8.6 Hz, 2 H, H3), 6.40 (br s, 1 H, NH), 4.31 (dt, J = 4.9, 2.2 Hz, 2 H, H8), 3.58 (t, J = 2.2 Hz, 2 H, H5).
^{13}C NMR (150 MHz, $CDCl_3$): δ 191.6 (C14), 166.1 (C9), 139.1 (C10), 138.5 (C13), 134.9 (C4), 132.8 (C1), 130.0 (C12), 129.4 (C3), 128.8 (C2), 127.9 (C11), 81.7 (C6), 77.7 (C7), 30.6 (C8), 24.7 (C5).
FTIR (ν_{max}, cm^{-1}): 3312 (w), 3064 (w), 2835 (w), 1704 (s), 1647 (s), 1610 (w), 1572 (w), 1538 (s), 1491 (s), 1419 (w), 1408 (w), 1386 (w), 1355 (w), 1291 (m), 1207 (m), 1154 (w), 1090 (m), 1016 (m), 977 (w), 848 (m), 801 (m), 757 (m).
HRMS (ESI): calculated for $C_{18}H_{13}NO_2Cl$ $[M-H]^-$ 310.0640, found 310.0639.
R_f = 0.35 (5% Et_2O/CH_2Cl_2).

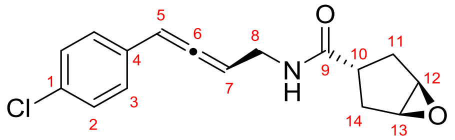

(1*R*,3*s*,5*S*)-*N*-((*R*)-4-(4-chlorophenyl)buta-2,3-dien-1-yl)-6-oxabicyclo[3.1.0]hexane-3-carboxamide (4e): Following the general procedure for asymmetric allenylation using (1*R*,3*s*,5*S*)-*N*-(prop-2-yn-1-yl)-6-oxabicyclo[3.1.0]hexane-3-carboxamide (33.0 mg, 0.2 mmol) and (4-chlorobenzylidene)hydrazine, purified by silica gel column chromatography (eluent: 30% Et_2O/CH_2Cl_2) provided the title compound as a white crystalline solid (32.0 mg, 0.110 mmol, 55%, 95% *ee*), m.p. 134-136 °C.

^{1}H NMR (600 MHz, $CDCl_3$): δ 7.27 (d, *J* = 8.5 Hz, 2 H, H2), 7.18 (d, *J* = 8.5 Hz, 2 H, H3), 6.24 (dt, *J* = 6.6, 3.4 Hz, 1 H, H5), 5.75 (br s, 1 H, NH), 5.65 (q, *J* = 6.6 Hz, 1 H, H7), 4.04 – 3.98 (m, 1 H, H8a), 3.92 – 3.85 (m, 1 H, H8b), 3.49 – 3.44 (m, 2 H, H12 and H13), 2.32 (tt, *J* = 9.6, 7.8 Hz, 1 H, H10), 2.18 (dd, *J* = 13.9, 7.8 Hz, 1 H, H11a/H14a), 2.08 (dd, *J* = 14.0, 7.8 Hz, 1 H, H11a/H14a), 1.92 (ddd, *J* = 13.9, 9.6, 1.3 Hz, 1 H, H11b/H14b), 1.82 (ddd, *J* = 14.0, 9.6, 1.3 Hz, 1 H, H11b/H14b).
^{13}C NMR (150 MHz, $CDCl_3$): δ 204.5 (C6), 173.9 (C9), 133.1 (C1), 132.3 (C4), 129.0 (C2), 128.1 (C3), 97.1 (C5), 93.7 (C7), 56.6 (C12 and C13), 39.0 (C10), 37.7 (C8), 31.93 (C11/C14), 31.86 (C11/C14).
FTIR (ν_{max}, cm^{-1}): 3298 (m), 3045 (w), 2925 (w), 1955 (w), 1649 (s), 1540 (m), 1491 (s), 1434 (w), 1391 (w), 1340 (w), 1294 (m), 1244 (w), 1220 (m), 1091 (m), 1060 (w), 1013 (w), 958 (w), 876 (w), 836 (s).
HRMS (ESI): calculated for $C_{16}H_{17}NO_2Cl$ $[M+H]^+$ 290.0942, found 290.0946.
R_f = 0.37 (30% Et_2O/CH_2Cl_2).
$[\alpha]_D^{25.0}$ = -230.6 ($CHCl_3$, c = 1.0, 95% *ee*).
HPLC: ChiralART SC, 90:10 hexane/isopropanol, 1.0 mL/min flow rate, T = 25 °C, λ_{max} = 254 nm; t_R (min) = 31.3 (minor), 36.1 (major).

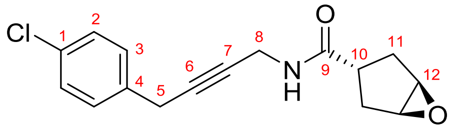

(1*R*,3*s*,5*S*)-*N*-(4-(4-chlorophenyl)but-2-yn-1-yl)-6-oxabicyclo[3.1.0]hexane-3-carboxamide (4e'): Isolated as the alkyne cross-product from asymmetric allenylation of (1*R*,3*s*,5*S*)-*N*-(prop-2-yn-1-yl)-6-oxabicyclo[3.1.0]hexane-3-carboxamide and (4-chloro-benzylidene)hydrazine, which provided the title compound as a white amorphous solid (21.7 mg, 0.075 mmol, 37%), m.p. 131-132 °C.

^{1}H NMR (600 MHz, $CDCl_3$): δ 7.28 (d, *J* = 8.5 Hz, 2 H, H2), 7.23 (d, *J* = 8.5 Hz, 2 H, H3), 5.76 (br s, 1 H, NH), 4.06 (dt, *J* = 5.0, 2.3 Hz, 2 H, H8), 3.54 (t, *J* = 2.3 Hz, 2 H, H5), 3.52 (s, 2 H, H12), 2.39 (tt, *J* = 9.6, 7.8 Hz, 1 H, H10), 2.24 (dd, *J* = 14.0, 7.8 Hz, 2 H, H11a), 1.95 (dd, *J* = 14.0, 9.6 Hz, 2 H, H11b).
^{13}C NMR (150 MHz, $CDCl_3$): δ 173.7 (C9), 135.0 (C4), 132.7 (C1), 129.3 (C3), 128.8 (C2), 81.1 (C6), 78.1 (C7), 56.6 (C12), 38.9 (C10), 31.9 (C11), 30.0 (C8), 24.6 (C5).
FTIR (ν_{max}, cm^{-1}): 3283 (m), 3031 (w), 2924 (w), 1649 (s), 1537 (s), 1491 (s), 1422 (w), 1408 (w), 1396 (w), 1348 (w), 1293 (m), 1243 (w), 1220 (m), 1090 (m), 1060 (w), 1016 (m), 958 (w), 838 (s), 807 (w).
HRMS (ESI): calculated for $C_{16}H_{16}NO_2ClNa$ $[M+Na]^+$ 312.0762, found 312.0770.
R_f = 0.46 (30% Et_2O/CH_2Cl_2).

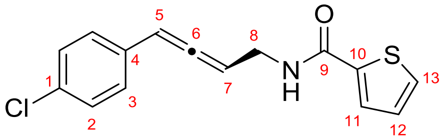

(*R*)-*N*-(4-(4-chlorophenyl)buta-2,3-dien-1-yl)thiophene-2-carboxamide (4f): Following the general procedure for asymmetric allenylation using *N*-(prop-2-yn-1-yl)thiophene-2-carboxamide (33.0 mg, 0.2 mmol) and (4-chlorobenzylidene)hydrazine, stirred for 20 min after addition of diazo compound, purified by silica gel column chromatography (eluent: CH_2Cl_2) provided the title compound as a yellow amorphous solid (30.7 mg, 0.106 mmol, 53%, 95% *ee*), m.p. 86-88 °C.

^{1}H NMR (600 MHz, $CDCl_3$): δ 7.46 – 7.43 (m, 2 H, H11 and H13), 7.26 (d, *J* = 8.5 Hz, 2 H, H2), 7.21 (d, *J* = 8.5 Hz, 2 H, H3), 7.03 (dd, *J* = 4.9, 3.8 Hz, 1 H, H12), 6.28 (dt superimposed on br s, *J* = 6.0, 3.2 Hz, 2 H, H5 and NH), 5.76 (q, *J* = 6.0 Hz, 1 H, H7), 4.15 (td, *J* = 6.0, 3.2 Hz, 2 H, H8).
^{13}C NMR (150 MHz, $CDCl_3$): δ 204.8 (C6), 161.9 (C9), 138.7 (C10), 133.1 (C1), 132.3 (C4), 130.1 (C11), 129.0 (C2), 128.3 (C13), 128.2 (C3), 127.8 (C12), 97.0 (C5), 93.4 (C7), 38.3 (C8).
FTIR (ν_{max}, cm^{-1}): 3310 (w), 3085 (w), 2921 (w), 1953 (w), 1626 (s), 1542 (s), 1510 (m), 1491 (s), 1420 (m), 1389 (w), 1358 (w), 1341 (w), 1297 (m), 1140 (w), 1090 (m), 1058 (w), 1013 (w), 875 (w), 859 (w), 833 (m).
HRMS (ESI): calculated for $C_{15}H_{12}NOSClNa$ $[M+Na]^+$ 312.0220, found 312.0226.
R_f = 0.34 (CH_2Cl_2).
$[\alpha]_D^{25.0}$ = -290.4 ($CHCl_3$, c = 1.0, 95% *ee*).
HPLC: ChiralART SC, 90:10 hexane/isopropanol, 1.0 mL/min flow rate, T = 25 °C, λ_{max} = 254 nm; t_R (min) = 30.6 (minor), 35.0 (major).

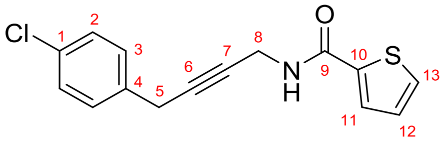

***N*-(4-(4-chlorophenyl)but-2-yn-1-yl)thiophene-2-carboxamide (4f'):** Isolated as the alkyne cross-product from asymmetric allenylation of *N*-(prop-2-yn-1-yl)thiophene-2-carboxamide and (4-chlorobenzylidene)hydrazine, which provided the title compound as a yellow gum (22.2 mg, 0.077 mmol, 38%).

^{1}H NMR (600 MHz, $CDCl_3$): δ 7.53 (dd, *J* = 3.7, 1.1 Hz, 1 H, H11), 7.48 (dd, *J* = 5.0, 1.1 Hz, 1 H, H13), 7.28 (d, *J* = 8.6 Hz, 2 H, H2), 7.25 (d, *J* = 8.6 Hz, 2 H, H3), 7.07 (dd, *J* = 5.0, 3.7 Hz, 1 H, H12), 6.17 (br s, 1 H, NH), 4.27 (dt, *J* = 5.3, 2.2 Hz, 2 H, H8), 3.57 (t, *J* = 2.2 Hz, 2 H, H5).
^{13}C NMR (150 MHz, $CDCl_3$): δ 161.6 (C9), 138.3 (C10), 135.0 (C4), 132.7 (C1), 130.4 (C13), 129.4 (C3), 128.8 (C2), 128.6 (C11), 127.8 (C12), 81.4 (C6), 78.0 (C7), 30.2 (C8), 24.7 (C5).
FTIR (ν_{max}, cm^{-1}): 3301 (w), 3090 (w), 2924 (w), 1626 (s), 1539 (s), 1509 (m), 1490 (s), 1419 (m), 1360 (w), 1347 (w), 1291 (m), 1246 (w), 1151 (w), 1122 (w), 1089 (m), 1057 (w), 1035 (w), 1016 (w), 949 (w), 913 (w), 858 (w), 799 (m).
HRMS (ESI): calculated for $C_{15}H_{12}NOSClNa$ $[M+Na]^+$ 312.0220, found 312.0229.
R_f = 0.40 (CH_2Cl_2).

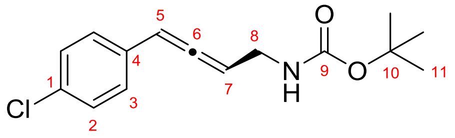

***tert*-butyl (*R*)-(4-(4-chlorophenyl)buta-2,3-dien-1-yl)carbamate (4g):** Following the general procedure for asymmetric allenylation using *N*-Boc-propargylamine (31.0 mg, 0.2 mmol) and (4-chlorobenzylidene)hydrazine, purified by silica gel column chromatography (eluent: CH_2Cl_2) provided the title compound as a pale yellow gum (27.6 mg, 0.099 mmol, 47%, 93% *ee*). Data are consistent with a reported racemic example.[35]

^{1}H NMR (600 MHz, $CDCl_3$): δ 7.26 (d, *J* = 8.5 Hz, 2 H, H2), 7.21 (d, *J* = 8.5 Hz, 2 H, H3), 6.24 (dt, *J* = 6.4, 3.2 Hz, 1 H, H5), 5.65 (q, *J* = 6.4 Hz, 1 H, H7), 4.70 (br s, 1 H, NH), 3.92 – 3.75 (m, 2 H, H8), 1.40 (s, 9 H, H11).
^{13}C NMR (150 MHz, $CDCl_3$): δ 204.7 (C6), 155.8 (C9), 132.9 (C1), 132.7 (C4), 128.9 (C2), 128.2 (C3), 96.7 (C5), 94.1 (C7), 79.7 (C10), 39.1 (C8), 28.5 (C11).
FTIR (ν_{max}, cm^{-1}): 3350 (w), 2979 (w), 2929 (w), 1952 (w), 1693 (s), 1592 (w), 1491 (s), 1455 (m), 1392 (m), 1367 (m), 1249 (s), 1161 (s), 1091 (s), 1049 (w), 1014 (m), 952 (w), 858 (w), 834 (m), 781 (w), 760 (m).
HRMS (ESI): calculated for $C_{15}H_{18}NO_2ClNa$ $[M+Na]^+$ 302.0918, found 302.0909.
$\boldsymbol{R_f}$ = 0.42 (CH_2Cl_2).
$[\alpha]_D^{26.7}$ = -101.0 ($CHCl_3$, c = 1.0, 93% *ee*).
HPLC: Chiralpak OD-H, 95:5 hexane/isopropanol, 1.0 mL/min flow rate, T = 25 °C, λ_{max} = 254 nm; t_R (min) = 7.7 (minor), 8.6 (major).

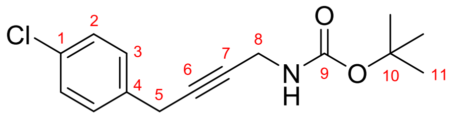

***tert*-butyl (*R*)-(4-(4-chlorophenyl)buta-2,3-dien-1-yl)carbamate (4g'):** Isolated as the alkyne cross-product from asymmetric allenylation of *N*-Boc-propargylamine and (4-chlorobenzylidene)hydrazine, which provided the title compound as a colourless gum (19.8 mg, 0.071 mmol, 35%).

^{1}H NMR (600 MHz, $CDCl_3$): δ 7.28 (d, *J* = 8.5 Hz, 2 H, H2), 7.25 (d, *J* = 8.5 Hz, 2 H, H3), 4.70 (br s, 1 H, NH), 3.95 (br s, 2 H, H8), 3.55 (t, *J* = 2.1 Hz, 2 H, H5), 1.45 (s, 9 H, H11).
^{13}C NMR (150 MHz, $CDCl_3$): δ 155.5 (C9), 135.2 (C4), 132.6 (C1), 129.4 (C3), 128.7 (C2), 80.6 (C6), 80.2 – 80.0 (br, C10), 78.9 (C7), 31.0 – 30.9 (br, C8), 28.5 (C11), 24.6 (C5).
FTIR (ν_{max}, cm^{-1}): 3338 (w), 2978 (w), 2934 (w), 1692 (s), 1491 (s), 1455 (w), 1421 (w), 1408 (w), 1392 (w), 1366 (m), 1330 (w), 1275 (m), 1249 (m), 1163 (s), 1090 (m), 1048 (w), 1016 (m), 931 (w), 916 (w), 862 (w), 806 (w).
HRMS (ESI): calculated for $C_{15}H_{17}NO_2Cl$ $[M-H]^-$ 278.0953, found 278.0960.
$\boldsymbol{R_f}$ = 0.46 (CH_2Cl_2).

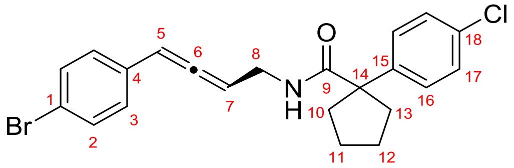

(*R*)-*N*-(4-(4-bromophenyl)buta-2,3-dien-1-yl)-1-(4-chlorophenyl)cyclopentane-1-carboxamide (4h): Following the general procedure for asymmetric allenylation using 1-(4-chlorophenyl)-*N*-(prop-2-yn-1-yl)cyclopentane-1-carboxamide (52.4 mg, 0.2 mmol) and (4-

bromobenzylidene)hydrazine, purified by silica gel column chromatography (eluent: 40% EtOAc/hexane) provided the title compound as a yellow gum (40.5 mg, 0.094 mmol, 47%, 95% *ee*).

^{1}H NMR (600 MHz, CDCl$_3$): δ 7.43 (d, *J* = 8.4 Hz, 2 H, H2), 7.15 (d, *J* = 8.7 Hz, 2 H, H17), 7.11 (d, *J* = 8.7 Hz, 2 H, H16), 7.05 (d, *J* = 8.4 Hz, 2 H, H3), 6.06 (dt, *J* = 6.3, 3.7 Hz, 1 H, H5), 5.58 (dt, *J* = 6.3, 4.8 Hz, 1 H, H7), 5.30 (br s, 1 H, NH), 3.95 – 3.85 (m, 1 H, H8a), 3.85 – 3.75 (m, 1 H, H8b), 2.44 – 2.33 (m, 2 H, H10/H13), 1.98 – 1.84 (m, 2 H, H10/H13), 1.84 – 1.71 (m, 2 H, H11/H12), 1.62 – 1.56 (m, 2 H, H11/H12).
^{13}C NMR (150 MHz, CDCl$_3$): δ 203.8 (C6), 175.9 (C9), 142.5 (C18), 132.9 (C4 and C15), 131.9 (C2), 128.8 (C16), 128.5 (C17), 128.4 (C3), 121.2 (C1), 97.5 (C5), 93.9 (C7), 58.9 (C14), 37.5 (C8), 37.1 (C10/C13), 37.0 (C10/C13), 24.01 (C11/C12), 24.00 (C11/C12).
FTIR (ν_{max}, cm^{-1}): 3339 (w), 2958 (w), 2873 (w), 1955 (w), 1646 (m), 1593 (w), 1488 (s), 1428 (w), 1401 (w), 1343 (w), 1262 (w), 1094 (m), 1070 (m), 1010 (s), 830 (s), 794 (m).
HRMS (ESI): calculated for $C_{22}H_{22}NOBrCl$ $[M+H]^+$ 430.0568, found 430.0550.
R_f = 0.33 (25% EtOAc/hexane).
$[\alpha]_D^{25.0}$ = -216.0 (CHCl$_3$, c = 1.0, 95% *ee*).
HPLC: ChiralART SC, 95:5 hexane/isopropanol, 1.0 mL/min flow rate, T = 25 °C, λ_{max} = 254 nm; t_R (min) = 19.1 (minor), 19.9 (major).

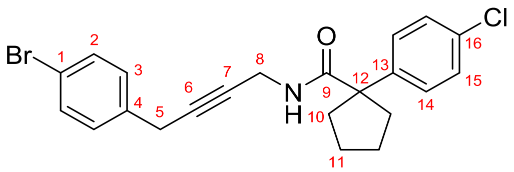

***N*-(4-(4-bromophenyl)but-2-yn-1-yl)-1-(4-chlorophenyl)cyclopentane-1-carboxamide (4h'):** Isolated as the alkyne cross-product from asymmetric allenylation of 1-(4-chlorophenyl)-*N*-(prop-2-yn-1-yl)cyclopentane-1-carboxamide and (4-bromobenzylidene)-hydrazine, which provided the title compound as a white crystalline solid (25.1 mg, 0.058 mmol, 29%), m.p. 116-119 °C.

^{1}H NMR (600 MHz, CDCl$_3$): δ 7.42 (d, *J* = 8.4 Hz, 2 H, H2), 7.29 (d, *J* = 8.9 Hz, 2 H, H15), 7.27 (d, *J* = 8.9 Hz, 2 H, H14), 7.12 (d, *J* = 8.4 Hz, 2 H, H3), 5.29 (br s, 1 H, NH), 3.96 (dt, *J* = 5.3, 2.3 Hz, 2 H, H8), 3.48 (t, *J* = 2.3 Hz, 2 H, H5), 2.48 – 2.41 (m, 2 H, H10a), 2.00 – 1.93 (m, 2 H, H10b), 1.85 – 1.77 (m, 2 H, H11a), 1.70 – 1.64 (m, 2 H, H11b).
^{13}C NMR (150 MHz, CDCl$_3$): δ 175.6 (C9), 142.5 (C13), 135.6 (C4), 133.0 (C16), 131.7 (C2), 129.7 (C3), 129.0 (C15), 128.4 (C14), 120.6 (C1), 80.7 (C6), 78.3 (C7), 58.9 (C12), 37.0 (C10), 30.2 (C8), 24.7 (C5), 24.0 (C11).
FTIR (ν_{max}, cm^{-1}): 3339 (w), 2954 (w), 2874 (w), 1646 (m), 1593 (w), 1487 (s), 1454 (w), 1419 (w), 1401 (w), 1349 (w), 1327 (w), 1268 (m), 1184 (w), 1135 (w), 1093 (m), 1071 (m), 1012 (s), 963 (w), 907 (m), 825 (m), 799 (m), 762 (w).
HRMS (ESI): calculated for $C_{22}H_{22}NOBrCl$ $[M+H]^+$ 430.0568, found 430.0565.
R_f = 0.42 (25% EtOAc/hexane).

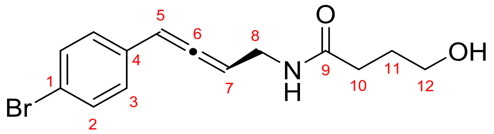

(*R*)-*N*-(4-(4-bromophenyl)buta-2,3-dien-1-yl)-4-hydroxybutanamide (4i): Following the general procedure for asymmetric allenylation using 4-hydroxy-*N*-(prop-2-yn-1-

yl)butanamide (28.2 mg, 0.2 mmol) and (4-bromobenzylidene)hydrazine, purified by silica gel column chromatography (eluent: 20% THF/Et_2O) provided the title compound as a white amorphous solid (30.3 mg, 0.098 mmol, 49%, 91% *ee*), m.p. 89-91 °C.

^{1}H NMR (600 MHz, $CDCl_3$): δ 7.42 (d, *J* = 8.3 Hz, 2 H, H2), 7.14 (d, *J* = 8.3 Hz, 2 H, H3), 6.24 (dt, *J* = 6.2, 3.0 Hz, 1 H, H5), 5.97 (br s, 1 H, NH), 5.65 (q, *J* = 6.2 Hz, 1 H, H7), 4.04 – 3.89 (m, 2 H, H8), 3.66 – 3.30 (m, 2 H, H12), 2.79 (br s, 1 H, OH), 2.34 – 2.29 (m, 2 H, H10), 1.84 – 1.79 (m, 2 H, H11).
^{13}C NMR (150 MHz, $CDCl_3$): δ 204.7 (C6), 173.5 (C9), 132.9 (C4), 131.9 (C2), 128.5 (C3), 121.2 (C1), 97.0 (C5), 93.6 (C7), 62.4 (C12), 37.9 (C8), 33.9 (C10), 28.1 (C11).
FTIR (ν_{max}, cm^{-1}): 3285 (br m), 2928 (w), 2876 (w), 1952 (w), 1637 (s), 1544 (m), 1488 (m), 1428 (w), 1380 (w), 1249 (w), 1217 (w), 1163 (w), 1070 (m), 1058 (m), 1010 (m), 918 (w), 881 (w), 832 (m), 810 (w).
HRMS (ESI): calculated for $C_{14}H_{16}NO_2BrNa$ $[M+Na]^+$ 332.0257, found 332.0262.
R_f = 0.20 (20% THF/Et_2O).
$[\alpha]_D^{25.0}$ = -237.5 ($CHCl_3$, c = 0.2, 91% *ee*).
HPLC: ChiralART SA, 93:7 hexane/isopropanol, 1.0 mL/min flow rate, T = 25 °C, λ_{max} = 254 nm; t_R (min) = 16.7 (minor), 18.2 (major).

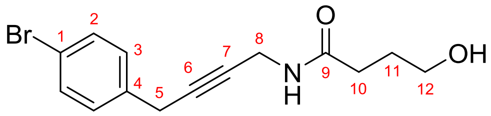

***N*-(4-(4-bromophenyl)but-2-yn-1-yl)-4-hydroxybutanamide (4i'):** Isolated as the alkyne cross-product from asymmetric allenylation of 4-hydroxy-*N*-(prop-2-yn-1-yl)butanamide and (4-bromobenzylidene)hydrazine, which provided the title compound as a white amorphous solid (23.0 mg, 0.074 mmol, 37%), m.p. 73-74 °C.

^{1}H NMR (600 MHz, $CDCl_3$): δ 7.43 (d, *J* = 8.4 Hz, 2 H, H2), 7.19 (d, *J* = 8.4 Hz, 2 H, H3), 5.89 (br s, 1 H, NH), 4.08 (dt, *J* = 4.9, 2.3 Hz, 2 H, H8), 3.69 (t, *J* = 5.7 Hz, 2 H, H12), 3.53 (t, *J* = 2.3 Hz, 2 H, H5), 2.62 (br s, 1 H, OH), 2.36 (t, *J* = 6.5 Hz, 2 H, H10), 1.88 (tt, *J* = 6.5, 5.7 Hz, 2 H, H11).
^{13}C NMR (150 MHz, $CDCl_3$): δ 173.1 (C9), 135.6 (C4), 131.8 (C2), 129.8 (C3), 120.7 (C1), 81.0 (C6), 78.2 (C7), 62.4 (C12), 33.7 (C10), 29.9 (C8), 28.0 (C11), 24.7 (C5).
FTIR (ν_{max}, cm^{-1}): 3272 (br m), 2937 (w), 1643 (s), 1542 (m), 1487 (s), 1420 (m), 1347 (w), 1259 (m), 1070 (m), 1012 (s), 916 (w), 801 (w).
HRMS (ESI): calculated for $C_{14}H_{16}NO_2BrNa$ $[M+Na]^+$ 332.0257, found 332.0268.
R_f = 0.26 (20% THF/Et_2O).

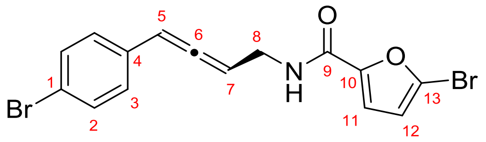

(*R*)-5-bromo-*N*-(4-(4-bromophenyl)buta-2,3-dien-1-yl)furan-2-carboxamide (4j): Following the general procedure for asymmetric allenylation using 5-bromo-*N*-(prop-2-yn-1-yl)furan-2-carboxamide (45.6 mg, 0.2 mmol) and (4-bromobenzylidene)hydrazine, purified by silica gel column chromatography (eluent: 2% Et_2O/CH_2Cl_2) provided the title compound as an orange amorphous solid (42.3 mg, 0.107 mmol, 53%, 96% *ee*), m.p. 108-110 °C.

^{1}H NMR (600 MHz, CDCl$_3$): δ 7.42 (d, *J* = 8.4 Hz, 2 H, H2), 7.15 (d, *J* = 8.4 Hz, 2 H, H3), 7.04 (d, *J* = 3.5 Hz, 1 H, H11), 6.50 (br s, 1 H, NH), 6.41 (d, *J* = 3.5 Hz, 1 H, H12), 6.26 (dt, *J* = 6.1, 3.0 Hz, 1 H, H5), 5.71 (q, *J* = 6.1 Hz, 1 H, H7), 4.18 – 4.08 (m, 2 H, H8).
^{13}C NMR (150 MHz, CDCl$_3$): δ 205.2 (C6), 157.2 (C9), 149.5 (C10), 132.8 (C4), 131.9 (C2), 128.6 (C3), 124.6 (C13), 121.2 (C1), 116.9 (C11), 114.3 (C12), 96.8 (C5), 93.1 (C7), 37.8 (C8).
FTIR (ν_{max}, cm^{-1}): 3295 (w), 1954 (w), 1647 (s), 1596 (m), 1575 (m), 1523 (s), 1488 (m), 1471 (s), 1430 (w), 1388 (w), 1342 (w), 1297 (m), 1221 (w), 1202 (w), 1171 (w), 1125 (w), 1070 (w), 1010 (m), 926 (w), 874 (w), 831 (m), 798 (m), 753 (w).
HRMS (ESI): calculated for $C_{15}H_{12}NO_2Br_2$ [M+H]$^+$ 395.9229, found 395.9234.
R_f = 0.37 (2% Et_2O/CH_2Cl_2).
$[\alpha]_D^{25.0}$ = -238.4 ($CHCl_3$, c = 1.0, 96% *ee*).
HPLC: ChiralART SC, 90:10 hexane/isopropanol, 1.0 mL/min flow rate, T = 25 °C, λ_{max} = 254 nm; t_R (min) = 19.8 (minor), 21.8 (major).

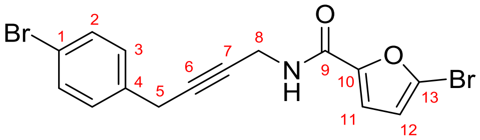

5-bromo-*N*-(4-(4-bromophenyl)but-2-yn-1-yl)furan-2-carboxamide (4j'): Isolated as the alkyne cross-product from asymmetric allenylation of 5-bromo-*N*-(prop-2-yn-1-yl)furan-2-carboxamide and (4-bromobenzylidene)hydrazine, which provided the title compound as an orange amorphous solid (31.0 mg, 0.078 mmol, 39%), m.p. 128-130 °C.

^{1}H NMR (600 MHz, CDCl$_3$): δ 7.44 (d, *J* = 8.4 Hz, 2 H, H2), 7.20 (d, *J* = 8.4 Hz, 2 H, H3), 7.09 (d, *J* = 3.5 Hz, 1 H, H11), 6.44 (d superimposed on br s, *J* = 3.5 Hz, 2 H, H12 and NH), 4.25 (dt, *J* = 5.2, 2.3 Hz, 2 H, H8), 3.56 (t, *J* = 2.3 Hz, 2 H, H5).
^{13}C NMR (150 MHz, CDCl$_3$): δ 156.8 (C9), 149.2 (C10), 135.5 (C4), 131.8 (C2), 129.8 (C3), 124.8 (C13), 120.7 (C1), 117.1 (C11), 114.4 (C12), 81.3 (C6), 77.8 (C7), 29.5 (C8), 24.7 (C5).
FTIR (ν_{max}, cm^{-1}): 3286 (w), 1650 (s), 1596 (m), 1574 (m), 1520 (s), 1487 (m), 1470 (s), 1419 (w), 1354 (w), 1295 (m), 1203 (w), 1172 (w), 1123 (w), 1071 (w), 1012 (m), 927 (w), 796 (m), 752 (w).
HRMS (ESI): calculated for $C_{15}H_{12}NO_2Br_2$ [M+H]$^+$ 395.9229, found 395.9230.
R_f = 0.44 (2% Et_2O/CH_2Cl_2).

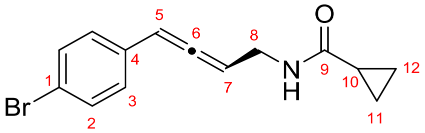

(*R*)-*N*-(4-(4-bromophenyl)buta-2,3-dien-1-yl)cyclopropanecarboxamide (4k): Following the general procedure for asymmetric allenylation using *N*-(prop-2-yn-1-yl)cyclopropanecarboxamide (24.6 mg, 0.2 mmol) and (4-bromobenzylidene)hydrazine, purified by silica gel column chromatography (eluent: 70% Et_2O/hexane) provided the title compound as an orange amorphous solid (30.0 mg, 0.103 mmol, 51%, 95% *ee*), m.p. 119-121 °C.

^{1}H NMR (600 MHz, CDCl$_3$): δ 7.42 (d, *J* = 8.4 Hz, 2 H, H2), 7.15 (d, *J* = 8.4 Hz, 2 H, H3), 6.24 (dt, *J* = 6.3, 3.1 Hz, 1 H, H5), 5.85 (br s, 1 H, NH), 5.67 (q, *J* = 6.3 Hz, 1 H, H7), 4.06 –

3.91 (m, 2 H, H8), 1.34 – 1.28 (m, 1 H, H10), 0.99 – 0.86 (m, 2 H, H11/H12), 0.74 – 0.64 (m, 2 H, H11/H12).
^{13}C NMR (150 MHz, CDCl$_3$): δ 204.7 – 204.6 (br, C6), 173.6 (C9), 133.0 (C4), 131.9 (C2), 128.5 (C3), 121.1 (C1), 96.8 (C5), 93.8 (C7), 38.0 (C8), 14.8 (C10), 7.37 (C11/C12), 7.36 (C11/C12).
FTIR (ν_{max}, cm^{-1}): 3277 (m), 3070 (w), 3011 (w), 2914 (w), 1954 (w), 1638 (s), 1545 (m), 1487 (m), 1451 (w), 1404 (w), 1336 (w), 1239 (m), 1196 (w), 1106 (w), 1070 (w), 1035 (w), 1010 (w), 991 (w), 912 (w), 879 (w), 830 (w).
HRMS (ESI): calculated for $C_{14}H_{14}NOBrNa$ [M+Na]$^+$ 314.0151, found 314.0136.
R_f = 0.29 (70% Et_2O/hexane).
$[\alpha]_D^{27.7}$ = -198.8 ($CHCl_3$, c = 0.5, 95% *ee*).
HPLC: ChiralART SA, 95:5 hexane/isopropanol, 1.0 mL/min flow rate, T = 25 °C, λ_{max} = 254 nm; t_R (min) = 20.1 (major), 28.3 (minor).

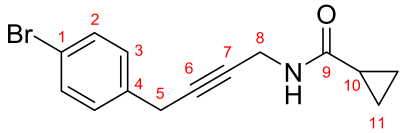

***N*-(4-(4-bromophenyl)but-2-yn-1-yl)cyclopropanecarboxamide (4k'):** Isolated as the alkyne cross-product from asymmetric allenylation of *N*-(prop-2-yn-1-yl)cyclopropanecarboxamide and (4-bromobenzylidene)hydrazine, which provided the title compound as an orange amorphous solid (20.4 mg, 0.070 mmol, 35%), m.p. 135-136 °C.

^{1}H NMR (600 MHz, CDCl$_3$): δ 7.43 (d, J = 8.4 Hz, 2 H, H2), 7.19 (d, J = 8.4 Hz, 2 H, H3), 5.85 (br s, 1 H, NH), 4.10 (dt, J = 4.9, 2.3 Hz, 2 H, H8), 3.54 (t, J = 2.3 Hz, 2 H, H5), 1.37 – 1.32 (m, 1 H, H10), 1.00 – 0.96 (m, 2 H, H11a), 0.77 – 0.73 (m, 2 H, H11b).
^{13}C NMR (150 MHz, CDCl$_3$): δ 173.3 (C9), 135.6 (C4), 131.7 (C2), 129.8 (C3), 120.7 (C1), 80.8 (C6), 78.6 (C7), 29.9 (C8), 24.7 (C5), 14.8 (C10), 7.5 (C11).
FTIR (ν_{max}, cm^{-1}): 3278 (m), 3098 (w), 3010 (w), 2942 (w), 2859 (w), 1637 (s), 1589 (w), 1551 (s), 1486 (m), 1447 (w), 1426 (w), 1403 (m), 1335 (m), 1243 (m), 1230 (m), 1109 (w), 1074 (w), 1055 (w), 1034 (w), 1011 (m), 1002 (w), 937 (w), 908 (m), 840 (m), 823 (w), 806 (w), 788 (m), 770 (w).
HRMS (ESI): calculated for $C_{14}H_{14}NOBrNa$ [M+Na]$^+$ 314.0151, found 314.0160.
R_f = 0.36 (70% Et_2O/hexane).

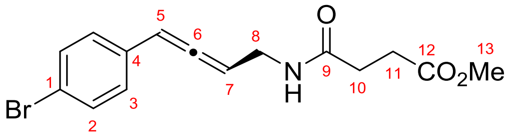

Methyl (*R*)-4-((4-(4-bromophenyl)buta-2,3-dien-1-yl)amino)-4-oxobutanoate (4l): Following the general procedure for asymmetric allenylation using methyl 4-oxo-4-(prop-2-yn-1-ylamino)butanoate (33.8 mg, 0.2 mmol) and (4-bromobenzylidene)hydrazine, purified by silica gel column chromatography (eluent: 5% Et_2O/CH_2Cl_2) provided the title compound as an orange amorphous solid (29.4 mg, 0.087 mmol, 43%, 91% *ee*), m.p. 90-92 °C.

^{1}H NMR (600 MHz, CDCl$_3$): δ 7.42 (d, J = 8.4 Hz, 2 H, H2), 7.14 (d, J = 8.4 Hz, 2 H, H3), 6.23 (dt, J = 6.4, 3.2 Hz, 1 H, H5), 5.90 (br s, 1 H, NH), 5.65 (q, J = 6.4 Hz, 1 H, H7), 4.04 – 3.89 (m, 2 H, H8), 3.64 (s, 3 H, H13), 2.65 – 2.58 (m, 2 H, H11), 2.48 – 2.42 (m, 2 H, H10).
^{13}C NMR (150 MHz, CDCl$_3$): δ 204.7 (C6), 173.6 (C12), 171.4 (C9), 132.9 (C4), 131.9 (C2), 128.5 (C3), 121.1 (C1), 96.9 (C5), 93.6 (C7), 52.0 (C13), 37.9 (C8), 31.1 (C10), 29.4 (C11).

FTIR (ν_{max}, cm^{-1}): 3305 (w), 2922 (w), 2851 (w), 1951 (w), 1734 (s), 1648 (s), 1538 (m), 1488 (s), 1436 (m), 1362 (m), 1199 (m), 1166 (s), 1069 (m), 1028 (w), 1009 (s), 913 (w), 876 (m), 830 (m).
HRMS (ESI): calculated for $C_{15}H_{16}NO_3BrNa$ $[M+Na]^+$ 360.0206, found 360.0195.
$\boldsymbol{R_f}$ = 0.15 (5% Et_2O/CH_2Cl_2).
$[\alpha]_D^{28.0}$ = -157.9 ($CHCl_3$, c = 1.0, 91% *ee*).
HPLC: ChiralART SA, 90:10 hexane/isopropanol, 1.0 mL/min flow rate, T = 25 °C, λ_{max} = 254 nm; t_R (min) = 23.1 (minor), 26.3 (major).

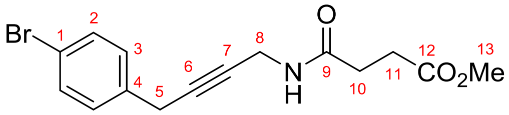

Methyl 4-((4-(4-bromophenyl)but-2-yn-1-yl)amino)-4-oxobutanoate (4l'): Isolated as the alkyne cross-product from asymmetric allenylation of methyl 4-oxo-4-(prop-2-yn-1-ylamino)butanoate and (4-bromobenzylidene)hydrazine, which provided the title compound as an off-white amorphous solid (25.2 mg, 0.075 mmol, 37%), m.p. 75-76 °C.

^{1}H NMR (600 MHz, CDCl$_3$): δ 7.43 (d, *J* = 8.4 Hz, 2 H, H2), 7.19 (d, *J* = 8.4 Hz, 2 H, H3), 5.87 (br s, 1 H, NH), 4.08 (dt, *J* = 4.9, 2.2 Hz, 2 H, H8), 3.68 (s, 3 H, H13), 3.53 (t, *J* = 2.2 Hz, 2 H, H5), 2.68 (t, *J* = 6.8 Hz, 2 H, H11), 2.49 (t, *J* = 6.8 Hz, 2 H, H10).
^{13}C NMR (150 MHz, CDCl$_3$): δ 173.5 (C12), 171.1 (C9), 135.6 (C4), 131.7 (C2), 129.8 (C3), 120.7 (C1), 80.9 (C6), 78.2 (C7), 52.0 (C13), 30.9 (C10), 29.9 (C8), 29.3 (C11), 24.7 (C5).
FTIR (ν_{max}, cm^{-1}): 3292 (w), 3061 (w), 2950 (w), 2925 (w), 1735 (s), 1649 (s), 1535 (m), 1487 (s), 1437 (m), 1420 (m), 1363 (m), 1200 (m), 1168 (s), 1071 (m), 1012 (s), 915 (w), 845 (m), 802 (m).
HRMS (ESI): calculated for $C_{15}H_{16}NO_3BrNa$ $[M+Na]^+$ 360.0206, found 360.0200.
$\boldsymbol{R_f}$ = 0.20 (5% Et_2O/CH_2Cl_2).

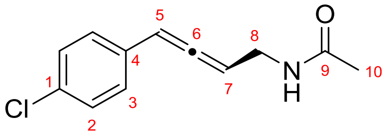

(*R*)-*N*-(4-(4-chlorophenyl)buta-2,3-dien-1-yl)acetamide (4m): Following the general procedure for asymmetric allenylation using *N*-(prop-2-yn-1-yl)acetamide (19.4 mg, 0.2 mmol) and (4-chlorobenzylidene)hydrazine, purified by silica gel column chromatography (eluent: 50% Et_2O/CH_2Cl_2) provided the title compound as an off-white amorphous solid (20.8 mg, 0.094 mmol, 47%, 94% *ee*), m.p. 81-82 °C.

^{1}H NMR (600 MHz, CDCl$_3$): δ 7.27 (d, *J* = 8.6 Hz, 2 H, H2), 7.20 (d, *J* = 8.6 Hz, 2 H, H3), 6.25 (dt, *J* = 6.4, 3.2 Hz, 1 H, H5), 5.74 (br s, 1 H, NH), 5.67 (q, *J* = 6.4 Hz, 1 H, H7), 4.02 – 3.89 (m, 2 H, H8), 1.95 (s, 3 H, H10).
^{13}C NMR (150 MHz, CDCl$_3$): δ 204.6 (C6), 170.1 (C9), 133.1 (C1), 132.4 (C4), 129.0 (C2), 128.1 (C3), 96.8 (C5), 93.5 (C7), 38.0 (C8), 23.3 (C10).
FTIR (ν_{max}, cm^{-1}): 3278 (m), 3072 (w), 2928 (w), 1954 (w), 1651 (s), 1549 (s), 1491 (s), 1431 (m), 1390 (w), 1372 (m), 1344 (w), 1292 (m), 1234 (w), 1091 (m), 1042 (w), 1013 (m), 877 (w), 833 (m).
HRMS (ESI): calculated for $C_{12}H_{13}NOCl$ $[M+H]^+$ 222.0680, found 222.0676.
$\boldsymbol{R_f}$ = 0.32 (50% Et_2O/CH_2Cl_2).
$[\alpha]_D^{25.2}$ = -234.5 ($CHCl_3$, c = 1.0, 94% *ee*).

HPLC: ChiralART SA, 97:3 hexane/isopropanol, 1.0 mL/min flow rate, T = 25 °C, λ_{max} = 254 nm; t_R (min) = 50.9 (minor), 54.1 (major).

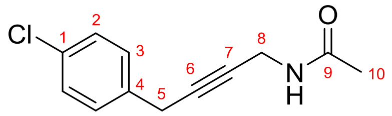

***N*-(4-(4-chlorophenyl)but-2-yn-1-yl)acetamide (4m'):** Isolated as the alkyne cross-product from asymmetric allenylation of *N*-(prop-2-yn-1-yl)acetamide and (4-chlorobenzylidene)-hydrazine, which provided the title compound as a white amorphous solid (14.1 mg, 0.064 mmol, 32%), m.p. 71-73 °C.

^{1}H NMR (600 MHz, $CDCl_3$): δ 7.28 (d, *J* = 8.5 Hz, 2 H, H2), 7.24 (d, *J* = 8.5 Hz, 2 H, H3), 5.71 (br s, 1 H, NH), 4.07 (dt, *J* = 4.9, 2.2 Hz, 2 H, H8), 3.55 (t, *J* = 2.2 Hz, 2 H, H5), 1.99 (s, 3 H, H10).
^{13}C NMR (150 MHz, $CDCl_3$): δ 169.7 (C9), 135.0 (C4), 132.7 (C1), 129.4 (C3), 128.8 (C2), 81.0 (C6), 78.2 (C7), 29.9 (C8), 24.6 (C5), 23.2 (C10).
FTIR (ν_{max}, cm^{-1}): 3277 (m), 3074 (w), 1651 (s), 1549 (m), 1492 (s), 1422 (m), 1373 (m), 1350 (m), 1289 (m), 1140 (w), 1090 (m), 1016 (m), 914 (w), 805 (w).
HRMS (ESI): calculated for $C_{12}H_{12}NOClNa$ $[M+Na]^+$ 244.0500, found 244.0496.
R_f = 0.34 (50% Et_2O/CH_2Cl_2).

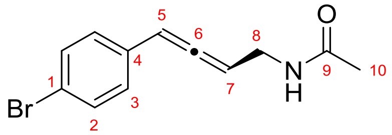

(*R*)-*N*-(4-(4-bromophenyl)buta-2,3-dien-1-yl)acetamide (4n): Following the general procedure for asymmetric allenylation using *N*-(prop-2-yn-1-yl)acetamide (19.4 mg, 0.2 mmol) and (4-bromobenzylidene)hydrazine, purified by silica gel column chromatography (eluent: 2% Et_2O/CH_2Cl_2) provided the title compound as an off-white amorphous solid (27.4 mg, 0.103 mmol, 51%, 96% *ee*), m.p. 92-94 °C.

^{1}H NMR (600 MHz, $CDCl_3$): δ 7.42 (d, *J* = 8.4 Hz, 2 H, H2), 7.14 (d, *J* = 8.4 Hz, 2 H, H3), 6.24 (dt, *J* = 6.4, 3.2 Hz, 1 H, H5), 5.70 (br s, 1 H, NH), 5.66 (q, *J* = 6.4 Hz, 1 H, H7), 4.03 – 3.89 (m, 2 H, H8), 1.95 (s, 3 H, H10).
^{13}C NMR (150 MHz, $CDCl_3$): δ 204.7 – 204.6 (br, C6), 170.1 (C9), 132.9 (C4), 131.9 (C2), 128.5 (C3), 121.1 (C1), 96.9 (C5), 93.6 (C7), 37.9 (C8), 23.3 (10).
FTIR (ν_{max}, cm^{-1}): 3275 (m), 3079 (w), 2930 (w), 1954 (w), 1651 (s), 1548 (s), 1488 (s), 1429 (m), 1372 (m), 1343 (w), 1291 (m), 1099 (w), 1070 (m), 1010 (m), 877 (w), 830 (m).
HRMS (ESI): calculated for $C_{12}H_{12}NOBrNa$ $[M+Na]^+$ 287.9994, found 287.9986.
R_f = 0.33 (50% Et_2O/CH_2Cl_2).
$[\alpha]_D^{25.0}$ = -233.0 ($CHCl_3$, c = 1.0, 96% *ee*).
HPLC: ChiralART SA, 95:5 hexane/isopropanol, 1.0 mL/min flow rate, T = 25 °C, λ_{max} = 254 nm; t_R (min) = 24.2 (minor), 25.5 (major).

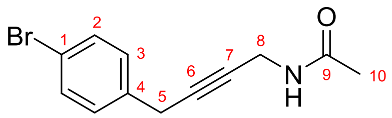

***N*-(4-(4-bromophenyl)but-2-yn-1-yl)acetamide (4n'):** Isolated as the alkyne cross-product from asymmetric allenylation of *N*-(prop-2-yn-1-yl)acetamide and (4-

bromobenzylidene)hydrazine, which provided the title compound as an off-white amorphous solid (20.0 mg, 0.075 mmol, 38%), m.p. 82-84 °C.

^{1}H NMR (600 MHz, CDCl$_3$): δ 7.43 (d, *J* = 8.4 Hz, 2 H, H2), 7.19 (d, *J* = 8.4 Hz, 2 H, H3), 5.67 (br s, 1 H, NH), 4.07 (dt, *J* = 4.9, 2.2 Hz, 2 H, H8), 3.53 (t, *J* = 2.2 Hz, 2 H, H5), 1.99 (s, 3 H, H10).
^{13}C NMR (150 MHz, CDCl$_3$): δ 169.7 (C9), 135.6 (C4), 131.7 (C2), 129.8 (C3), 120.7 (C1), 80.9 (C6), 78.3 (C7), 29.9 (C8), 24.7 (C5), 23.2 (C10).
FTIR (ν_{max}, cm^{-1}): 3273 (m), 3079 (w), 1652 (s), 1548 (m), 1487 (s), 1421 (m), 1373 (m), 1350 (m), 1288 (m), 1184 (w), 1141 (w), 1070 (m), 1027 (w), 1012 (m), 914 (w), 801 (w).
HRMS (ESI): calculated for $C_{12}H_{12}NOBrNa$ [M+Na]$^+$ 287.9994, found 287.9985.
R$_f$ = 0.39 (50% Et_2O/CH_2Cl_2).

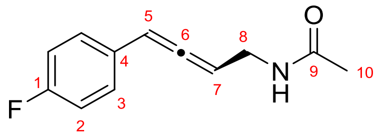

(*R*)-*N*-(4-(4-fluorophenyl)buta-2,3-dien-1-yl)acetamide (4o): Following the general procedure for asymmetric allenylation using *N*-(prop-2-yn-1-yl)acetamide (19.4 mg, 0.2 mmol) and (4-fluorobenzylidene)hydrazine, purified by silica gel column chromatography (eluent: 50% Et_2O/CH_2Cl_2) provided the title compound as an off-white amorphous solid (18.3 mg, 0.089 mmol, 45%, 91% *ee*), m.p. 75-78 °C.

^{1}H NMR (600 MHz, CDCl$_3$): δ 7.24 (dd, *J* = 8.7, 5.4 Hz, 2 H, H3), 7.00 (t, *J* = 8.7 Hz, 2 H, H2), 6.27 (dt, *J* = 6.4, 3.2 Hz, 1 H, H5), 5.66 (q, *J* = 6.4 Hz, 1 H, H7), 4.05 – 3.87 (m, 2 H, H8), 1.96 (s, 3 H, H10).
^{13}C NMR (150 MHz, CDCl$_3$): δ 204.3 (C6), 170.1 (C9), 162.2 (d, *J* = 246.7 Hz, C1), 129.8 (d, *J* = 3.3 Hz, C4), 128.4 (d, *J* = 8.0 Hz, C3), 115.8 (d, *J* = 21.8 Hz, C2), 96.8 (C5), 93.3 (C7), 38.0 (C8), 23.4 (C10).
^{19}F NMR (376 MHz, CDCl$_3$): δ -114.7 (s, 1 F, F1).
FTIR (ν_{max}, cm^{-1}): 3277 (w), 3074 (w), 2925 (w), 1953 (w), 1651 (m), 1603 (w), 1548 (m), 1507 (s), 1432 (w), 1394 (w), 1373 (m), 1345 (w), 1285 (m), 1223 (s), 1156 (m), 1096 (w), 1041 (w), 1014 (w), 877 (w), 837 (m), 765 (w).
HRMS (ESI): calculated for $C_{12}H_{12}FNONa$ [M+Na]$^+$ 228.0795, found 228.0802.
R$_f$ = 0.35 (50% Et_2O/CH_2Cl_2).
$[\alpha]_D^{25.0}$ = -201.5 ($CHCl_3$, c = 1.0, 91% *ee*).
HPLC: ChiralART SA, 95:5 hexane/isopropanol, 1.0 mL/min flow rate, T = 25 °C, λ_{max} = 254 nm; t_R (min) = 22.0 (minor), 22.9 (major).

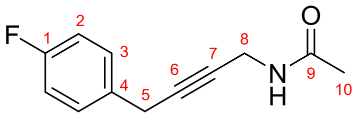

***N*-(4-(4-fluorophenyl)but-2-yn-1-yl)acetamide (4o'):** Isolated as the alkyne cross-product from asymmetric allenylation of *N*-(prop-2-yn-1-yl)acetamide and (4-fluorobenzylidene)hydrazine, which provided the title compound as a white amorphous solid (14.2 mg, 0.069 mmol, 35%), m.p. 56-57 °C.

^{1}H NMR (600 MHz, CDCl$_3$): δ 7.27 (dd, *J* = 8.7, 5.4 Hz, 2 H, H3), 7.00 (t, *J* = 8.7 Hz, 2 H, H2), 5.67 (br s, 1 H, NH), 4.08 (dt, *J* = 4.8, 2.2 Hz, 2 H, H8), 3.55 (t, *J* = 2.2 Hz, 2 H, H5), 1.99 (s, 3 H, H10).

^{13}C NMR (150 MHz, $CDCl_3$): δ 169.7 (C9), 161.9 (d, J = 244.7 Hz, C1), 132.2 (d, J = 3.2 Hz, C4), 129.5 (d, J = 8.0 Hz, C3), 115.5 (d, J = 21.5 Hz, C2), 81.4 (C6), 78.0 (C7), 29.9 (C8), 24.4 (C5), 23.2 (C10).
^{19}F NMR (376 MHz, $CDCl_3$): δ -116.4 (s, 1 F, F1).
FTIR (ν_{max}, cm^{-1}): 3278 (w), 3073 (w), 2925 (w), 1651 (m), 1604 (w), 1548 (m), 1508 (s), 1424 (w), 1374 (w), 1350 (w), 1287 (w), 1221 (m), 1158 (w), 1094 (w), 1017 (w), 912 (w), 820 (w), 758 (w).
HRMS (ESI): calculated for $C_{12}H_{13}FNO$ $[M+H]^+$ 206.0976, found 206.0976.
R_f = 0.42 (50% Et_2O/CH_2Cl_2).

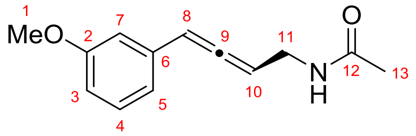

(*R*)-*N*-(4-(3-methoxyphenyl)buta-2,3-dien-1-yl)acetamide (4p): Following the general procedure for asymmetric allenylation using *N*-(prop-2-yn-1-yl)acetamide (19.4 mg, 0.2 mmol) and (3-methoxybenzylidene)hydrazine, purified by silica gel column chromatography (eluent: 40% Et_2O/CH_2Cl_2) provided the title compound as a colourless gum (19.4 mg, 0.089 mmol, 45%, 95% *ee*).

^{1}H NMR (600 MHz, $CDCl_3$): δ 7.22 (t, J = 7.9 Hz, 1 H, H4), 6.87 (d, J = 7.9 Hz, 1 H, H5), 6.84 – 6.82 (m, 1 H, H7), 6.77 (dd, J = 7.9, 2.5 Hz, 1 H, H3), 6.27 (dt, J = 6.5, 3.3 Hz, 1 H, H8), 5.70 (br s, 1 H, NH), 5.67 (q, J = 6.5 Hz, 1 H, H10), 4.02 – 3.91 (m, 2 H, H11), 3.81 (s, 3 H, H1), 1.96 (s, 3 H, H13).
^{13}C NMR (150 MHz, $CDCl_3$): δ 204.6 (C9), 170.1 (C12), 160.1 (C2), 135.3 (C6), 129.8 (C4), 119.6 (C5), 113.2 (C3), 112.2 (C7), 97.7 (C8), 93.1 (C10), 55.4 (C1), 38.0 (C11), 23.4 (C13).
FTIR (ν_{max}, cm^{-1}): 3289 (m), 3068 (w), 2938 (w), 2835 (w), 1951 (w), 1652 (s), 1598 (m), 1582 (m), 1549 (m), 1490 (m), 1467 (m), 1439 (m), 1408 (w), 1372 (w), 1344 (w), 1264 (m), 1155 (m), 1092 (w), 1043 (m), 995 (w), 883 (w), 787 (m).
HRMS (ESI): calculated for $C_{13}H_{15}NO_2Na$ $[M+Na]^+$ 240.0995, found 240.1000.
R_f = 0.31 (40% Et_2O/CH_2Cl_2).
$[\alpha]_D^{25.0}$ = -218.1 ($CHCl_3$, c = 1.0, 95% *ee*).
HPLC: ChiralART SC, 85:15 hexane/isopropanol, 1.0 mL/min flow rate, T = 25 °C, λ_{max} = 254 nm; t_R (min) = 16.3 (minor), 21.1 (major).

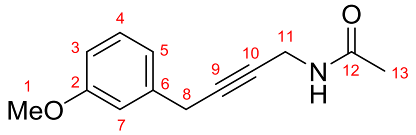

***N*-(4-(3-methoxyphenyl)but-2-yn-1-yl)acetamide (4p'):** Isolated as the alkyne cross-product from asymmetric allenylation of *N*-(prop-2-yn-1-yl)acetamide and (3-methoxybenzylidene)hydrazine, which provided the title compound as a colourless gum (15.9 mg, 0.073 mmol, 37%).

^{1}H NMR (600 MHz, $CDCl_3$): δ 7.23 (t, J = 8.0 Hz, 1 H, H4), 6.91 – 6.86 (m, 2 H, H5 and H7), 6.78 (dd, J = 8.0, 2.3 Hz, 1 H, H3), 5.69 (br s, 1 H, NH), 4.07 (dt, J = 4.8, 2.2 Hz, 2 H, H11), 3.81 (s, 3 H, H1), 3.56 (t, J = 2.2 Hz, 2 H, H8), 1.99 (s, 3 H, H13).
^{13}C NMR (150 MHz, $CDCl_3$): δ 169.7 (C12), 159.9 (C2), 138.1 (C6), 129.7 (C4), 120.4 (C5), 113.9 (C3), 112.1 (C7), 81.5 (C9), 77.8 (C10), 55.4 (C1), 30.0 (C11), 25.2 (C8), 23.2 (C13).

FTIR (ν_{max}, cm^{-1}): 3283 (m), 3070 (w), 2837 (w), 1651 (s), 1601 (m), 1586 (m), 1546 (m), 1489 (m), 1455 (m), 1436 (m), 1373 (w), 1351 (w), 1317 (w), 1283 (m), 1260 (s), 1155 (m), 1136 (w), 1090 (w), 1047 (m), 852 (w), 775 (m).
HRMS (ESI): calculated for $C_{13}H_{15}NO_2Na$ [M+Na]$^+$ 240.0995, found 240.1000.
$\boldsymbol{R_f}$ = 0.38 (40% Et_2O/CH_2Cl_2).

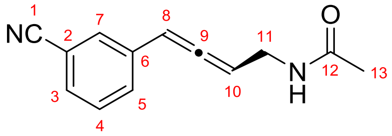

(*R*)-*N*-(4-(3-cyanophenyl)buta-2,3-dien-1-yl)acetamide (4q): Following the general procedure for asymmetric allenylation using *N*-(prop-2-yn-1-yl)acetamide (19.4 mg, 0.2 mmol) and 3-(hydrazonomethyl)benzonitrile, purified by silica gel column chromatography (eluent: 50% Et_2O/CH_2Cl_2) provided the title compound as a colourless gum (17.0 mg, 0.080 mmol, 40%, 89% *ee*).

^{1}H NMR (600 MHz, $CDCl_3$): δ 7.55 (s, 1 H, H7), 7.52 – 7.47 (m, 2 H, H3 and H5), 7.41 (t, *J* = 7.8 Hz, 1 H, H4), 6.27 (dt, *J* = 6.3, 3.1 Hz, 1 H, H8), 5.74 (q superimposed on br s, *J* = 6.3 Hz, 2 H, H10 and NH), 4.07 – 3.92 (m, 2 H, H11), 1.98 (s, 3 H, H13).
^{13}C NMR (150 MHz, $CDCl_3$): δ 205.3 (C9), 170.2 (C12), 135.6 (C6), 131.1 (C5), 130.7 (C3), 130.3 (C7), 129.6 (C4), 118.8 (C1), 113.0 (C2), 96.1 (C8), 94.2 (C10), 38.0 (C11), 23.3 (C13).
FTIR (ν_{max}, cm^{-1}): 3281 (m), 3072 (w), 2929 (w), 2231 (m), 1954 (w), 1651 (s), 1599 (w), 1543 (s), 1483 (m), 1432 (m), 1372 (m), 1344 (w), 1283 (m), 1228 (w), 1096 (w), 1043 (w), 999 (w), 898 (w), 801 (m).
HRMS (ESI): calculated for $C_{13}H_{13}N_2O$ [M+H]$^+$ 213.1022, found 213.1017.
$\boldsymbol{R_f}$ = 0.25 (50% Et_2O/CH_2Cl_2).
$[\boldsymbol{\alpha}]_D^{25.0}$ = -194.4 ($CHCl_3$, c = 1.0, 89% *ee*).
HPLC: ChiralART SA, 93:7 hexane/isopropanol, 1.0 mL/min flow rate, T = 25 °C, λ_{max} = 254 nm; t_R (min) = 30.5 (minor), 32.8 (major).

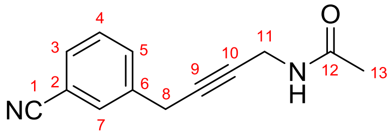

***N*-(4-(3-cyanophenyl)but-2-yn-1-yl)acetamide (4q'):** Isolated as the alkyne cross-product from asymmetric allenylation of *N*-(prop-2-yn-1-yl)acetamide and 3-(hydrazonomethyl)-benzonitrile, which provided the title compound as a yellow amorphous solid (23.4 mg, 0.110 mmol, 55%), m.p. 87-90 °C.

^{1}H NMR (600 MHz, $CDCl_3$): δ 7.62 (s, 1 H, H7), 7.56 – 7.51 (m, 2 H, H3 and H5), 7.41 (t, *J* = 7.8 Hz, 1 H, H4), 5.88 (br s, 1 H, NH), 4.08 (dt, *J* = 5.3, 2.2 Hz, 2 H, H11), 3.62 (t, *J* = 2.2 Hz, 2 H, H8), 2.00 (s, 3 H, H13).
^{13}C NMR (150 MHz, $CDCl_3$): δ 169.9 (C12), 138.1 (C6), 132.6 (C5), 131.5 (C7), 130.6 (C3), 129.4 (C4), 118.8 (C1), 112.7 (C2), 79.6 (C9), 79.2 (C10), 29.7 (C11), 24.8 (C8), 23.2 (C13).
FTIR (ν_{max}, cm^{-1}): 3290 (m), 3065 (w), 2923 (w), 2231 (m), 1652 (s), 1543 (s), 1483 (m), 1432 (m), 1374 (m), 1351 (w), 1285 (m), 1093 (w), 1027 (w), 788 (m).
HRMS (ESI): calculated for $C_{13}H_{13}N_2O$ [M+H]$^+$ 213.1022, found 213.1028.
$\boldsymbol{R_f}$ = 0.32 (50% Et_2O/CH_2Cl_2).

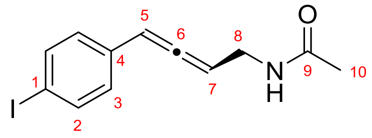

(*R*)-*N*-(4-(4-iodophenyl)buta-2,3-dien-1-yl)acetamide (4r): Following the general procedure for asymmetric allenylation using *N*-(prop-2-yn-1-yl)acetamide (19.4 mg, 0.2 mmol) and (4-iodobenzylidene)hydrazine, purified by silica gel column chromatography (eluent: 50% Et_2O/CH_2Cl_2) provided the title compound as an off-white amorphous solid (27.6 mg, 0.088 mmol, 44%, 95% *ee*), m.p. 118-119 °C.

^{1}H NMR (600 MHz, $CDCl_3$): δ 7.62 (d, *J* = 8.3 Hz, 2 H, H2), 7.01 (d, *J* = 8.3 Hz, 2 H, H3), 6.22 (dt, *J* = 6.3, 3.2 Hz, 1 H, H5), 5.70 (br s, 1 H, NH), 5.66 (q, *J* = 6.3 Hz, 1 H, H7), 4.03 – 3.87 (m, 2 H, H8), 1.95 (s, 3 H, H10).
^{13}C NMR (150 MHz, $CDCl_3$): δ 204.7 – 204.6 (br, C6), 170.1 (C9), 137.9 (C2), 133.5 (C4), 128.7 (C3), 97.0 (C5), 93.6 (C7), 92.5 (C1), 37.9 (C8), 23.3 (C10).
FTIR (ν_{max}, cm^{-1}): 3280 (m), 3068 (w), 2925 (w), 1951 (w), 1651 (s), 1551 (s), 1484 (s), 1429 (w), 1372 (m), 1343 (w), 1291 (m), 1108 (w), 1058 (w), 1006 (m), 876 (w), 826 (m).
HRMS (ESI): calculated for $C_{12}H_{12}NOINa$ $[M+Na]^+$ 335.9856, found 335.9862.
$\boldsymbol{R_f}$ = 0.38 (50% Et_2O/CH_2Cl_2).
$[\alpha]_D^{25.0}$ = -198.4 ($CHCl_3$, c = 1.0, 95% *ee*).
HPLC: ChiralART SA, 97:3 hexane/isopropanol, 1.0 mL/min flow rate, T = 25 °C, λ_{max} = 254 nm; t_R (min) = 58.0 (minor), 62.4 (major).

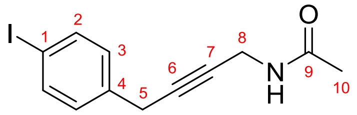

***N*-(4-(4-iodophenyl)but-2-yn-1-yl)acetamide (4r'):** Isolated as the alkyne cross-product from asymmetric allenylation of *N*-(prop-2-yn-1-yl)acetamide and (4-iodobenzylidene)hydrazine, which provided the title compound as a yellow amorphous solid (22.4 mg, 0.072 mmol, 36%), m.p. 103-104 °C.

^{1}H NMR (600 MHz, $CDCl_3$): δ 7.63 (d, *J* = 8.3 Hz, 2 H, H2), 7.06 (d, *J* = 8.3 Hz, 2 H, H3), 5.72 (br s, 1 H, NH), 4.07 (dt, *J* = 5.2, 2.1 Hz, 2 H, H8), 3.52 (t, *J* = 2.1 Hz, 2 H, H5), 1.99 (s, 3 H, H10).
^{13}C NMR (150 MHz, $CDCl_3$): δ 169.7 (C9), 137.7 (C2), 136.3 (C4), 130.1 (C3), 92.0 (C1), 80.8 (C6), 78.3 (C7), 29.9 (C8), 24.8 (C5), 23.2 (C10).
FTIR (ν_{max}, cm^{-1}): 3283 (m), 3069 (w), 2919 (w), 1648 (s), 1544 (s), 1483 (s), 1420 (m), 1400 (m), 1373 (m), 1350 (m), 1287 (m), 1187 (w), 1140 (w), 1110 (w), 1087 (w), 1060 (w), 1027 (w), 1008 (s), 918 (w), 778 (m).
HRMS (ESI): calculated for $C_{12}H_{13}NOI$ $[M+H]^+$ 314.0036, found 314.0049.
$\boldsymbol{R_f}$ = 0.45 (50% Et_2O/CH_2Cl_2).

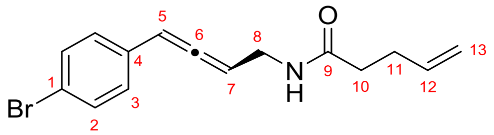

(*R*)-*N*-(4-(4-bromophenyl)buta-2,3-dien-1-yl)pent-4-enamide (4s): Following the general procedure for asymmetric allenylation using *N*-(prop-2-yn-1-yl)pent-4-enamide (27.4 mg, 0.2 mmol) and (4-bromobenzylidene)hydrazine, purified by silica gel column chromatography

(eluent: 40% EtOAc/hexane) provided the title compound as an off-white crystalline solid (25.1 mg, 0.082 mmol, 41%, 92% *ee*), m.p. 97-99 °C.

^{1}H NMR (600 MHz, CDCl$_3$): δ 7.42 (d, *J* = 8.4 Hz, 2 H, H2), 7.14 (d, *J* = 8.4 Hz, 2 H, H3), 6.23 (dt, *J* = 6.4, 3.2 Hz, 1 H, H5), 5.81 – 5.69 (m, 2 H, H12 and NH), 5.65 (q, *J* = 6.4 Hz, 1 H, H7), 5.01 (dq, *J* = 17.2, 1.2 Hz, 1 H, H13$_{trans}$), 4.94 (dq, *J* = 10.2, 1.2 Hz, 1 H, H13$_{cis}$), 4.04 – 3.89 (m, 2 H, H8), 2.36 – 2.30 (m, 2 H, H11), 2.27 – 2.22 (m, 2 H, H10).
^{13}C NMR (150 MHz, CDCl$_3$): δ 204.6 – 204.5 (br, C6), 172.3 (C9), 137.1 (C12), 132.9 (C4), 131.9 (C2), 128.5 (C3), 121.1 (C1), 115.7 (C13), 96.9 (C5), 93.7 (C7), 37.7 (C8), 35.9 (C10), 29.6 (C11).
FTIR (ν_{max}, cm^{-1}): 3294 (m), 3079 (w), 2920 (w), 2851 (w), 1952 (w), 1638 (s), 1538 (s), 1488 (s), 1431 (m), 1381 (w), 1339 (w), 1264 (m), 1230 (m), 1196 (w), 1111 (w), 1070 (m), 1010 (s), 912 (m), 877 (m), 832 (s), 811 (m), 750 (w).
HRMS (ESI): calculated for $C_{15}H_{16}NOBrNa$ [M+Na]$^+$ 328.0307, found 328.0309.
$\boldsymbol{R_f}$ = 0.28 (40% EtOAc/hexane).
$[\alpha]_D^{24.8}$ = -172.5 (CHCl$_3$, c = 1.0, 92% *ee*).
HPLC: ChiralART SC, 95:5 hexane/isopropanol, 1.0 mL/min flow rate, T = 25 °C, λ_{max} = 254 nm; t_R (min) = 32.1 (minor), 33.1 (major).

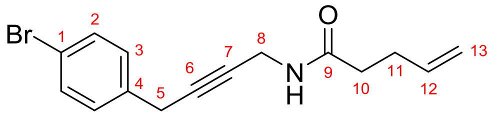

***N*-(4-(4-bromophenyl)but-2-yn-1-yl)pent-4-enamide (4s'):** Isolated as the alkyne cross-product from asymmetric allenylation of *N*-(prop-2-yn-1-yl)pent-4-enamide and (4-bromobenzylidene)hydrazine, which provided the title compound as an orange amorphous solid (19.1 mg, 0.062 mmol, 31%), m.p. 68-70 °C.

^{1}H NMR (600 MHz, CDCl$_3$): δ 7.43 (d, *J* = 8.4 Hz, 2 H, H2), 7.19 (d, *J* = 8.4 Hz, 2 H, H3), 5.82 (ddt, *J* = 17.0, 10.2, 6.5 Hz, 1 H, H12), 5.65 (br s, 1 H, NH), 5.07 (dq, *J* = 17.0, 1.4 Hz, 1 H, H13$_{trans}$), 5.01 (dq, *J* = 10.2, 1.4 Hz, 1 H, H13$_{cis}$), 4.08 (dt, *J* = 4.8, 2.2 Hz, 2 H, H8), 3.53 (t, *J* = 2.2 Hz, 2 H, H5), 2.43 – 2.36 (m, 2 H, H11), 2.28 (t, *J* = 7.5 Hz, 2 H, H10).
^{13}C NMR (150 MHz, CDCl$_3$): δ 171.9 (C9), 137.0 (C12), 135.6 (C4), 131.7 (C2), 129.8 (C3), 120.7 (C1), 115.9 (C13), 80.9 (C6), 78.3 (C7), 35.7 (C10), 29.8 (C8), 29.6 (C11), 24.7 (C5).
FTIR (ν_{max}, cm^{-1}): 3290 (w), 3078 (w), 2920 (m), 2851 (w), 1641 (s), 1539 (s), 1487 (s), 1419 (m), 1345 (w), 1266 (m), 1184 (w), 1139 (w), 1110 (w), 1071 (m), 1030 (w), 1012 (s), 914 (m), 840 (m), 801 (m).
HRMS (ESI): calculated for $C_{15}H_{17}NOBr$ [M+H]$^+$ 306.0488, found 306.0485.
$\boldsymbol{R_f}$ = 0.36 (40% EtOAc/hexane).

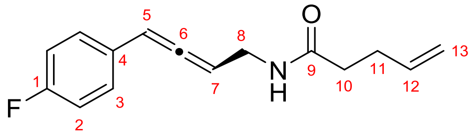

(*R*)-*N*-(4-(4-fluorophenyl)buta-2,3-dien-1-yl)pent-4-enamide (4t): Following the general procedure for asymmetric allenylation using *N*-(prop-2-yn-1-yl)pent-4-enamide (27.4 mg, 0.2 mmol) and (4-fluorobenzylidene)hydrazine, purified by silica gel column chromatography (eluent: 60% Et$_2$O/hexane) provided the title compound as a yellow amorphous solid (21.8 mg, 0.089 mmol, 44%, 95% *ee*), m.p. 66-68 °C.

^{1}H NMR (600 MHz, $CDCl_3$): δ 7.24 (dd, *J* = 8.7, 5.4 Hz, 2 H, H3), 7.00 (t, *J* = 8.7 Hz, 2 H, H2), 6.27 (dt, *J* = 6.5, 3.3 Hz, 1 H, H5), 5.77 (ddt, *J* = 17.1, 10.2, 6.5 Hz, 1 H, H12), 5.69 (br s, 1 H, NH), 5.66 (q, *J* = 6.5 Hz, 1 H, H7), 5.01 (dd, *J* = 17.1, 1.4 Hz, 1 H, $H13_{trans}$), 4.94 (dd, *J* = 10.2, 1.4 Hz, 1 H, $H13_{cis}$), 4.05 – 3.89 (m, 2 H, H8), 2.36 – 2.30 (m, 2 H, H11), 2.29 – 2.18 (m, 2 H, H10).
^{13}C NMR (150 MHz, $CDCl_3$): δ 204.3 – 204.2 (br, C6), 172.3 (C9), 162.3 (d, *J* = 246.7 Hz, C1), 137.1 (C12), 129.8 (d, *J* = 3.3 Hz, C4), 128.5 (d, *J* = 8.1 Hz, C3), 115.8 (d, *J* = 22.0 Hz, C2), 115.7 (C13), 96.8 (C5), 93.4 (C7), 37.8 (C8), 35.9 (C10), 29.6 (C11).
^{19}F NMR (376 MHz, $CDCl_3$): δ -114.7 (s, 1 F, F1).
FTIR (ν_{max}, cm^{-1}): 3284 (w), 3072 (w), 2921 (w), 1951 (w), 1643 (m), 1604 (w), 1543 (m), 1508 (s), 1436 (w), 1394 (w), 1341 (w), 1263 (w), 1225 (m), 1156 (w), 1094 (w), 1014 (w), 996 (w), 916 (w), 876 (w), 838 (m), 765 (w).
HRMS (ESI): calculated for $C_{15}H_{16}FNONa$ $[M+Na]^+$ 268.1108, found 268.1115.
R_f = 0.21 (60% Et_2O/hexane).
$[\alpha]_D^{25.0}$ = -212.4 ($CHCl_3$, c = 1.0, 95% *ee*).
HPLC: ChiralART SA, 97:3 hexane/isopropanol, 1.0 mL/min flow rate, T = 25 °C, λ_{max} = 254 nm; t_R (min) = 35.4 (major), 38.0 (minor).

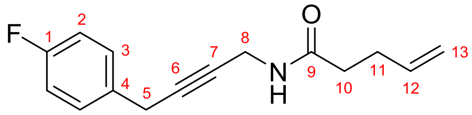

***N*-(4-(4-fluorophenyl)but-2-yn-1-yl)pent-4-enamide (4t'):** Isolated as the alkyne cross-product from asymmetric allenylation of *N*-(prop-2-yn-1-yl)pent-4-enamide and (4-fluorobenzylidene)hydrazine, which provided the title compound as a yellow amorphous solid (21.3 mg, 0.087 mmol, 43%), m.p. 49-50 °C.

^{1}H NMR (600 MHz, $CDCl_3$): δ 7.26 (dd, *J* = 8.7, 5.4 Hz, 2 H, H3), 7.00 (t, *J* = 8.7 Hz, 2 H, H2), 5.82 (ddt, *J* = 17.0, 10.2, 6.5 Hz, 1 H, H12), 5.67 (br s, 1 H, NH), 5.07 (dd, *J* = 17.0, 1.4 Hz, 1 H, $H13_{trans}$), 5.00 (dd, *J* = 10.2, 1.4 Hz, 1 H, $H13_{cis}$), 4.08 (dt, *J* = 4.8, 2.2 Hz, 2 H, H8), 3.55 (t, *J* = 2.2 Hz, 2 H, H5), 2.43 – 2.36 (m, 2 H, H11), 2.28 (t, *J* = 7.5 Hz, 2 H, H10).
^{13}C NMR (150 MHz, $CDCl_3$): δ 171.9 (C9), 161.9 (d, *J* = 244.8 Hz, C1), 137.0 (C12), 132.2 (d, *J* = 3.2 Hz, C4), 129.5 (d, *J* = 8.0 Hz, C3), 115.8 (C13), 115.5 (d, *J* = 21.5 Hz, C2), 81.4 (C6), 78.1 (C7), 35.7 (C10), 29.8 (C8), 29.6 (C11), 24.5 (C5).
^{19}F NMR (376 MHz, $CDCl_3$): δ -116.4 (s, 1 F, F1).
FTIR (ν_{max}, cm^{-1}): 3281 (w), 3069 (w), 2918 (w), 1642 (m), 1604 (w), 1539 (m), 1508 (s), 1423 (w), 1345 (w), 1268 (w), 1222 (m), 1158 (w), 1094 (w), 1016 (w), 996 (w), 914 (w), 838 (w), 756 (w).
HRMS (ESI): calculated for $C_{15}H_{16}FNONa$ $[M+Na]^+$ 268.1108, found 268.1114.
R_f = 0.29 (60% Et_2O/hexane).

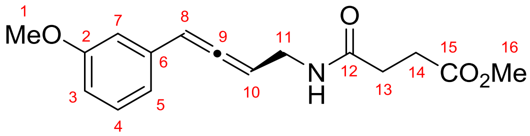

Methyl (*R*)-4-((4-(3-methoxyphenyl)buta-2,3-dien-1-yl)amino)-4-oxobutanoate (4u): Following the general procedure for asymmetric allenylation using methyl 4-oxo-4-(prop-2-yn-1-ylamino)butanoate (33.8 mg, 0.2 mmol) and (3-methoxybenzylidene)hydrazine, purified by silica gel column chromatography (eluent: 15% Et_2O/CH_2Cl_2) provided the title compound as a colourless gum (26.2 mg, 0.091 mmol, 45%, 95% *ee*).

^{1}H NMR (600 MHz, CDCl$_3$): δ 7.22 (t, J = 7.9 Hz, 1 H, H4), 6.87 (d, J = 7.9 Hz, 1 H, H5), 6.84 – 6.82 (m, 1 H, H7), 6.77 (dd, J = 7.9, 2.4 Hz, 1 H, H3), 6.26 (dt, J = 6.5, 3.3 Hz, 1 H, H8), 5.89 (br s, 1 H, NH), 5.65 (q, J = 6.5 Hz, 1 H, H10), 4.03 – 3.90 (m, 2 H, H11), 3.81 (s, 3 H, H1), 3.64 (s, 3 H, H16), 2.66 – 2.58 (m, 2 H, H14), 2.48 – 2.41 (m, 2 H, H13).
^{13}C NMR (150 MHz, CDCl$_3$): δ 204.6 (C9), 173.6 (C15), 171.4 (C12), 160.0 (C2), 135.3 (C6), 129.8 (C4), 119.6 (C5), 113.2 (C3), 112.2 (C7), 97.8 (C8), 93.1 (C10), 55.4 (C1), 51.9 (C16), 38.0 (C11), 31.1 (C13), 29.4 (C14).
FTIR (ν_{max}, cm^{-1}): 3313 (w), 2953 (w), 2838 (w), 1954 (w), 1735 (m), 1649 (m), 1597 (m), 1581 (m), 1536 (m), 1490 (m), 1454 (m), 1437 (m), 1408 (w), 1363 (w), 1319 (w), 1260 (s), 1154 (s), 1083 (w), 1039 (m), 994 (w), 946 (w), 875 (m), 846 (m), 785 (m), 754 (m).
HRMS (ESI): calculated for $C_{16}H_{19}NO_4Na$ $[M+Na]^+$ 312.1206, found 312.1213.
R_f = 0.30 (15% Et_2O/CH_2Cl_2).
$[\alpha]_D^{25.0}$ = -190.1 ($CHCl_3$, c = 1.0, 95% *ee*).
HPLC: ChiralART SC, 80:20 hexane/isopropanol, 1.0 mL/min flow rate, T = 25 °C, λ_{max} = 254 nm; t_R (min) = 36.1 (minor), 40.2 (major).

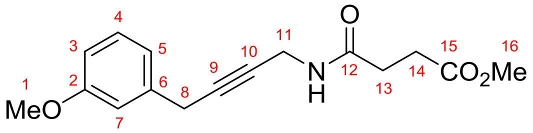

Methyl 4-((4-(3-methoxyphenyl)but-2-yn-1-yl)amino)-4-oxobutanoate (4u'): Isolated as the alkyne cross-product from asymmetric allenylation of methyl 4-oxo-4-(prop-2-yn-1-ylamino)butanoate and (3-methoxybenzylidene)hydrazine, which provided the title compound as a colourless gum (22.4 mg, 0.077 mmol, 39%).

^{1}H NMR (600 MHz, CDCl$_3$): δ 7.23 (t, J = 8.0 Hz, 1 H, H4), 6.91 – 6.85 (m, 2 H, H5 and H7), 6.78 (dd, J = 8.0, 2.4 Hz, 1 H, H3), 5.88 (br s, 1 H, NH), 4.08 (dt, J = 4.8, 2.3 Hz, 2 H, H11), 3.81 (s, 3 H, H1), 3.68 (s, 3 H, H16), 3.56 (t, J = 2.3 Hz, 2 H, H8), 2.67 (t, J = 6.8 Hz, 2 H, H14), 2.48 (t, J = 6.8 Hz, 2 H, H13).
^{13}C NMR (150 MHz, CDCl$_3$): δ 173.5 (C15), 171.0 (C12), 159.9 (C2), 138.1 (C6), 129.7 (C4), 120.4 (C5), 113.9 (C3), 112.1 (C7), 81.5 (C9), 77.8 (C10), 55.4 (C1), 52.0 (C16), 30.9 (C13), 30.0 (C11), 29.3 (C14), 25.2 (C8).
FTIR (ν_{max}, cm^{-1}): 3304 (w), 2951 (w), 2837 (w), 1735 (s), 1649 (s), 1601 (m), 1586 (m), 1533 (m), 1489 (s), 1453 (m), 1436 (s), 1317 (m), 1257 (s), 1201 (m), 1161 (s), 1080 (w), 1040 (s), 995 (w), 933 (w), 879 (w), 846 (m), 775 (m).
HRMS (ESI): calculated for $C_{16}H_{19}NO_4Na$ $[M+Na]^+$ 312.1206, found 312.1216.
R_f = 0.38 (15% Et_2O/CH_2Cl_2).

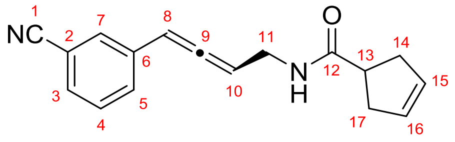

(*R*)-*N*-(4-(3-cyanophenyl)buta-2,3-dien-1-yl)cyclopent-3-ene-1-carboxamide (4v): Following the general procedure for asymmetric allenylation using *N*-(prop-2-yn-1-yl)cyclopent-3-ene-1-carboxamide (29.8 mg, 0.2 mmol) and 3-(hydrazonomethyl)benzonitrile, purified by silica gel column chromatography (eluent: 80% Et_2O/hexane) provided the title compound as a yellow amorphous solid (21.2 mg, 0.080 mmol, 40%, 92% *ee*), m.p. 95-96 °C.

^{1}H NMR (600 MHz, $CDCl_3$): δ 7.53 (s, 1 H, H7), 7.51 – 7.46 (m, 2 H, H3 and H5), 7.40 (t, *J* = 7.7 Hz, 1 H, H4), 6.26 (dt, *J* = 6.4, 3.2 Hz, 1 H, H8), 5.79 (br s, 1 H, NH), 5.74 (q, *J* = 6.4 Hz, 1 H, H10), 5.64 – 5.58 (m, 2 H, H15 and H16), 4.07 – 4.00 (m, 1 H, H11a), 3.99 – 3.92 (m, 1 H, H11b), 2.92 (qn, *J* = 7.9 Hz, 1 H, H13), 2.59 – 2.52 (m, 4 H, H14 and H17).
^{13}C NMR (150 MHz, $CDCl_3$): δ 205.2 (C9), 176.1 (C12), 135.6 (C6), 131.1 (C5), 130.7 (C3), 130.2 (C7), 129.6 (C4), 129.35 (C15/C16), 129.33 (C15/C16), 118.8 (C1), 112.9 (C2), 96.2 (C8), 94.6 (C10), 43.6 (C13), 37.6 (C11), 37.11 (C14/C17), 37.05 (C14/C17).
FTIR (ν_{max}, cm^{-1}): 3286 (m), 3058 (w), 2926 (m), 2852 (w), 2231 (m), 1956 (w), 1649 (s), 1536 (s), 1483 (m), 1438 (m), 1340 (w), 1296 (w), 1228 (m), 1181 (w), 899 (w), 871 (w), 842 (w), 801 (m).
HRMS (ESI): calculated for $C_{17}H_{17}N_2O$ $[M+H]^+$ 265.1335, found 265.1340.
R_f = 0.29 (80% Et_2O/hexane).
$[\alpha]_D^{25.0}$ = -213.1 ($CHCl_3$, c = 1.0, 92% *ee*).
HPLC: ChiralART SC, 80:20 hexane/isopropanol, 1.0 mL/min flow rate, T = 25 °C, λ_{max} = 254 nm; t_R (min) = 20.4 (minor), 22.4 (major).

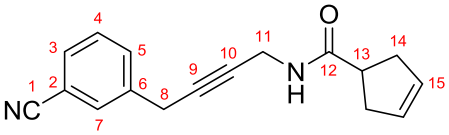

***N*-(4-(3-cyanophenyl)but-2-yn-1-yl)cyclopent-3-ene-1-carboxamide (4v'):** Isolated as the alkyne cross-product from asymmetric allenylation of *N*-(prop-2-yn-1-yl)cyclopent-3-ene-1-carboxamide and 3-(hydrazonomethyl)benzonitrile, which provided the title compound as an off-white amorphous solid (31.7 mg, 0.120 mmol, 60%), m.p. 91-93 °C.

^{1}H NMR (600 MHz, $CDCl_3$): δ 7.62 (s, 1 H, H7), 7.56 – 7.51 (m, 2 H, H3 and H5), 7.42 (t, *J* = 7.8 Hz, 1 H, H4), 5.82 (br s, 1 H, NH), 5.67 (s, 2 H, H15), 4.10 (dt, *J* = 5.3, 2.1 Hz, 2 H, H11), 3.62 (t, *J* = 2.1 Hz, 2 H, H8), 2.97 (qn, *J* = 8.1 Hz, 1 H, H13), 2.63 (d, *J* = 8.1 Hz, 4 H, H14).
^{13}C NMR (150 MHz, $CDCl_3$): δ 175.7 (C12), 138.1 (C6), 132.6 (C5), 131.5 (C7), 130.6 (C3), 129.4 (C4), 129.3 (C15), 118.8 (C1), 112.7 (C2), 79.6 (C9), 79.4 (C10), 43.4 (C13), 37.0 (C14), 29.7 (C11), 24.9 (C8).
FTIR (ν_{max}, cm^{-1}): 3287 (m), 3058 (w), 2922 (w), 2851 (w), 2231 (m), 1648 (s), 1584 (w), 1532 (s), 1483 (m), 1435 (m), 1342 (w), 1297 (w), 1228 (m), 1180 (w), 1094 (w), 1038 (w), 946 (w), 900 (w), 844 (m), 788 (m).
HRMS (ESI): calculated for $C_{17}H_{17}N_2O$ $[M+H]^+$ 265.1335, found 265.1344.
R_f = 0.36 (80% Et_2O/hexane).

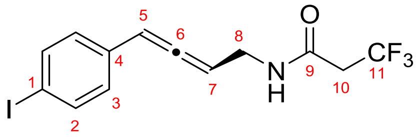

(*R*)-3,3,3-trifluoro-*N*-(4-(4-iodophenyl)buta-2,3-dien-1-yl)propanamide (4w): Following the general procedure for asymmetric allenylation using 3,3,3-trifluoro-*N*-(prop-2-yn-1-yl)propanamide (37.1 mg, 0.2 mmol) and (4-iodobenzylidene)hydrazine, purified by silica gel column chromatography (eluent: 70% Et_2O/hexane) provided the title compound as an off-white amorphous solid (37.1 mg, 0.097 mmol, 49%, 95% *ee*), m.p. 131-132 °C.

Scale up to 5 mmol was possible using a slightly modified procedure as follows: To a stirred **L9**-CuI solution in a 250 mL RBF (prepared on the same scale as stated for the stock solution

– 51.4 mL, containing 0.15 equiv. ligand, 0.1 equiv. CuI and 2 equiv. Et_3N) was added was added 3,3,3-trifluoro-*N*-(prop-2-yn-1-yl)propanamide (0.825 g, 5.0 mmol, 1.0 equiv.). The mixture was pre-mixed at r.t. for 10 min, forming a clear red-orange homogeneous solution of the copper acetylide-ligand complex. Solutions of hydrazone (0.1 M) and DIPEA (0.2 M) in CH_2Cl_2 were passed through eight pre-conditioned column reactors concurrently (Omnifit® column, 6.6 mm i.d. × 50 mm length), packed with activated MnO_2 (0.86 g), at a flow rate of 0.5 mL min^{-1} each. When the FlowIR® showed that the intensity of the diazo peak was stable, 12.5 mL of each output stream from the eight columns (2.0 equiv. total with respect to the hydrazone) was directly added into the reaction vial (over 25 min) containing the copper acetylide-ligand complex and the reaction mixture further stirred at r.t. for 10 min. The solution was evaporated under reduced pressure and the residue taken up in CH_2Cl_2 (50 mL). The mixture was washed with concentrated ammonium hydroxide/saturated aqueous NH_4Cl solution (1:9, 50 mL), then the organic layer dried ($MgSO_4$) and evaporated under reduced pressure. The residue was immediately purified by silica gel column chromatography (eluent: 70% Et_2O/hexane) to provide the title compound as an off-white amorphous solid (0.889 g, 2.33 mmol, 47%, 95% *ee*).

^{1}H NMR (600 MHz, $CDCl_3$): δ 7.62 (d, *J* = 8.3 Hz, 2 H, H2), 7.00 (d, *J* = 8.3 Hz, 2 H, H3), 6.25 (dt, *J* = 6.4, 3.2 Hz, 1 H, H5), 6.04 (br s, 1 H, NH), 5.66 (q, *J* = 6.4 Hz, 1 H, H7), 4.07 – 3.92 (m, 2 H, H8), 3.04 (q, *J* = 10.6 Hz, 2 H, H10).
^{13}C NMR (150 MHz, $CDCl_3$): δ 204.7 (C6), 162.6 (q, *J* = 3.4 Hz, C9), 137.9 (C2), 133.2 (C4), 128.8 (C3), 124.0 (d, *J* = 276.8 Hz, C11), 97.5 (C5), 92.9 (C7), 92.7 (C1), 41.7 (q, *J* = 29.6 Hz, C10), 38.1 (C8).
^{19}F NMR (376 MHz, $CDCl_3$): δ -63.0 (s, 3 F, F11).
FTIR (ν_{max}, cm^{-1}): 3308 (m), 1958 (m), 1652 (s), 1556 (m), 1486 (w), 1385 (w), 1337 (w), 1268 (w), 1238 (m), 1140 (m), 1107 (m), 1061 (w), 1006 (w), 923 (w), 879 (w), 851 (w), 833 (w).
HRMS (ESI): calculated for $C_{13}H_{12}F_3NOI$ $[M+H]^+$ 381.9910, found 381.9927.
R_f = 0.30 (70% Et_2O/hexane).
$[\alpha]_D^{25.0}$ = -176.5 ($CHCl_3$, c = 1.0, 95% *ee*).
HPLC: ChiralART SC, 95:5 hexane/isopropanol, 1.0 mL/min flow rate, T = 25 °C, λ_{max} = 254 nm; t_R (min) = 16.9 (minor), 18.3 (major).

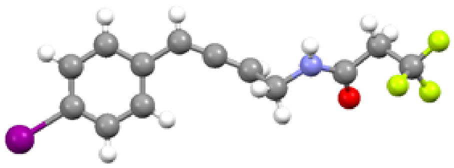

Slow diffusion of hexane into a saturated solution of **4w** in CH_2Cl_2 provided white needle-like crystals suitable for X-ray diffraction analysis. The absolute stereochemistry of the structure was unambiguously confirmed by X-ray crystallography and deposited at the Cambridge Crystallographic Data Centre, deposition number CCDC 1514945.

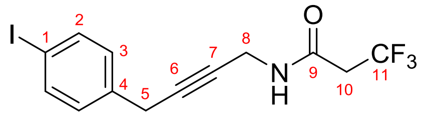

3,3,3-trifluoro-*N*-(4-(4-iodophenyl)but-2-yn-1-yl)propanamide (4w'): Isolated as the alkyne cross-product from asymmetric allenylation of 3,3,3-trifluoro-*N*-(prop-2-yn-1-yl)propanamide and (4-iodobenzylidene)hydrazine, which provided the title compound as an

orange amorphous solid (34.4 mg, 0.090 mmol, 45%), m.p. 136-137 °C. During the scale up of **4w**, **4w'** was also isolated as an off-white amorphous solid (0.821 g, 2.15 mmol, 43%).

^{1}H NMR (600 MHz, CDCl$_3$): δ 7.63 (d, *J* = 8.3 Hz, 2 H, H2), 7.05 (d, *J* = 8.3 Hz, 2 H, H3), 6.02 (br s, 1 H, NH), 4.12 (dt, *J* = 4.8, 2.2 Hz, 2 H, H8), 3.53 (t, *J* = 2.2 Hz, 2 H, H5), 3.08 (q, *J* = 10.5 Hz, 2 H, H10).
^{13}C NMR (150 MHz, CDCl$_3$): δ 162.3 (q, *J* = 3.4 Hz, C9), 137.8 (C2), 136.1 (C4), 130.0 (C3), 124.0 (q, *J* = 276.8 Hz, C11), 92.1 (C1), 81.6 (C6), 77.3 (C7), 41.6 (q, *J* = 29.7 Hz, C10), 30.3 (C8), 24.8 (C5).
^{19}F NMR (376 MHz, CDCl$_3$): δ -62.9 (s, 3 F, F11).
FTIR (ν_{max}, cm^{-1}): 3305 (m), 1654 (s), 1555 (m), 1483 (w), 1451 (w), 1399 (w), 1352 (w), 1278 (w), 1234 (m), 1128 (m), 1111 (m), 1060 (w), 1007 (w), 920 (w), 851 (w), 785 (w).
HRMS (ESI): calculated for $C_{13}H_{12}F_3NOI$ [M+H]$^+$ 381.9910, found 381.9904.
R$_f$ = 0.38 (70% Et$_2$O/hexane).

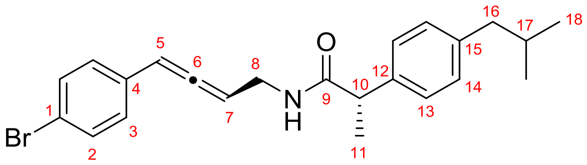

(*S*)-*N*-((*R*)-4-(4-bromophenyl)buta-2,3-dien-1-yl)-2-(4-isobutylphenyl)propanamide (4x): Following the general procedure for asymmetric allenylation using (*S*)-2-(4-isobutylphenyl)-*N*-(prop-2-yn-1-yl)propanamide (48.7 mg, 0.2 mmol) and (4-bromobenzylidene)hydrazine, purified by silica gel column chromatography (eluent: 50% Et$_2$O/hexane) provided the title compound as a pale yellow gum (42.2 mg, 0.102 mmol, 51%, 96% *de*).

^{1}H NMR (600 MHz, CDCl$_3$): δ 7.42 (d, *J* = 8.4 Hz, 2 H, H2), 7.09 – 7.05 (m, 4 H, H3 and H13), 6.96 (d, *J* = 8.0 Hz, 2 H, H14), 6.11 (dt, *J* = 6.5, 3.7 Hz, 1 H, H5), 5.59 (dt, *J* = 6.5, 5.2 Hz, 1 H, H7), 5.50 (br s, 1 H, NH), 3.90 (td, *J* = 5.2, 3.7 Hz, 2 H, H8), 3.48 (q, *J* = 7.3 Hz, 1 H, H10), 2.39 (d, *J* = 7.2 Hz, 2 H, H16), 1.85 – 1.76 (m, 1 H, H17), 1.48 (d, *J* = 7.3 Hz, 3 H, H11), 0.89 (d, *J* = 6.6 Hz, 6 H, H18).
^{13}C NMR (150 MHz, CDCl$_3$): δ 204.2 (C6), 174.5 (C9), 140.9 (C15), 138.1 (C12), 133.0 (C4), 131.9 (C2), 129.7 (C14), 128.4 (C3), 127.6 (C13), 121.1 (C1), 97.3 (C5), 93.9 (C7), 46.9 (C10), 45.1 (C16), 37.4 (C8), 30.3 (C17), 22.5 (C18), 18.5 (C11).
FTIR (ν_{max}, cm^{-1}): 3290 (w), 2954 (m), 2926 (w), 2868 (w), 1953 (w), 1647 (s), 1541 (m), 1512 (m), 1488 (m), 1464 (w), 1422 (w), 1384 (w), 1366 (w), 1339 (w), 1231 (w), 1192 (w), 1070 (m), 1010 (m), 876 (w), 830 (m).
HRMS (ESI): calculated for $C_{23}H_{26}NOBrNa$ [M+Na]$^+$ 434.1090, found 434.1099.
R$_f$ = 0.23 (50% Et$_2$O/hexane).
$[\alpha]_D^{25.0}$ = -229.6 (CHCl$_3$, c = 0.5, 96% *de*).
HPLC: ChiralART SC, 95:5 hexane/isopropanol, 1.0 mL/min flow rate, T = 25 °C, λ_{max} = 254 nm; t_R (min) = 26.8 (minor), 28.6 (major).

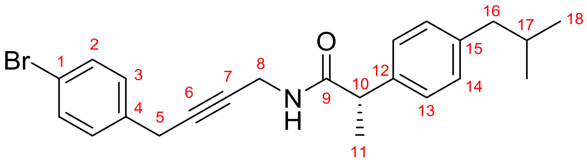

(*S*)-*N*-(4-(4-bromophenyl)but-2-yn-1-yl)-2-(4-isobutylphenyl)propanamide (4x'): Isolated as the alkyne cross-product from asymmetric allenylation of (*S*)-2-(4-isobutylphenyl)-*N*-(prop-2-yn-1-yl) and (4-bromobenzylidene)hydrazine, after repurification

by silica gel column chromatography (eluent: 10% Et_2O/CH_2Cl_2) of the alkyne-containing fractions, which provided the title compound as a pale yellow gum (23.7 mg, 0.057 mmol, 29%).

^{1}H NMR (600 MHz, $CDCl_3$): δ 7.41 (d, J = 8.4 Hz, 2 H, H2), 7.18 (d, J = 8.0 Hz, 2 H, H13), 7.14 (d, J = 8.4 Hz, 2 H, H3), 7.11 (d, J = 8.0 Hz, 2 H, H14), 5.51 (br s, 1 H, NH), 4.09 – 4.02 (m, 1 H, H8a), 4.01 – 3.94 (m, 1 H, H8b), 3.54 (q, J = 7.2 Hz, 1 H, H10), 3.48 (t, J = 2.2 Hz, 2 H, H5), 2.46 (d, J = 7.2 Hz, 2 H, H16), 1.90 – 1.80 (m, 1 H, H17), 1.51 (d, J = 7.2 Hz, 3 H, H11), 0.90 (d, J = 6.6 Hz, 6 H, H18).
^{13}C NMR (150 MHz, $CDCl_3$): δ 174.2 (C9), 141.0 (C15), 138.3 (C12), 135.6 (C4), 131.7 (C2), 129.8 (C3/C14), 129.7 (C3/C14), 127.5 (C13), 120.6 (C1), 80.7 (C6), 78.4 (C7), 46.7 (C10), 45.1 (C16), 30.3 (C17), 29.9 (C8), 24.7 (C5), 22.5 (C18), 18.6 (C11).
FTIR (ν_{max}, cm^{-1}): 3279 (w), 2955 (m), 2926 (w), 2868 (w), 1647 (s), 1534 (m), 1511 (m), 1487 (s), 1465 (m), 1420 (m), 1383 (w), 1366 (m), 1263 (w), 1230 (m), 1189 (w), 1071 (m), 1012 (s), 911 (w), 848 (w), 800 (m).
HRMS (ESI): calculated for $C_{23}H_{26}NOBrNa$ $[M+Na]^+$ 434.1090, found 434.1096.
$\boldsymbol{R_f}$ = 0.34 (50% Et_2O/hexane).
$[\alpha]_D^{25.0}$ = -5.5 ($CHCl_3$, c = 1.0).

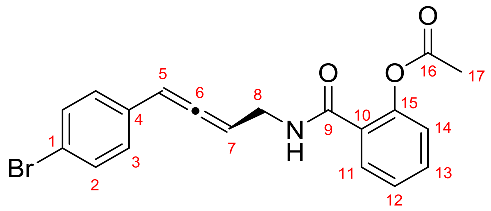

(*R*)-2-((4-(4-bromophenyl)buta-2,3-dien-1-yl)carbamoyl)phenyl acetate (4y): Following the general procedure for asymmetric allenylation using 2-(prop-2-yn-1-ylcarbamoyl)phenyl acetate (43.4 mg, 0.2 mmol) and (4-bromobenzylidene)hydrazine, purified by silica gel column chromatography (eluent: 65% Et_2O/hexane) provided the title compound as an off-white amorphous solid (39.6 mg, 0.103 mmol, 51%, 96% *ee*), m.p. 111-113 °C.

^{1}H NMR (600 MHz, $CDCl_3$): δ 7.65 (dd, J = 7.7, 1.6 Hz, 1 H, H11), 7.47 – 7.43 (m, 1 H, H13), 7.42 (d, J = 8.4 Hz, 2 H, H2), 7.27 – 7.23 (m, 1 H, H12), 7.16 (d, J = 8.4 Hz, 2 H, H3), 7.08 (dd, J = 8.2, 0.8 Hz, 1 H, H14), 6.44 (br s, 1 H, NH), 6.26 (dt, J = 6.2, 3.1 Hz, 1 H, H5), 5.73 (q, J = 6.2 Hz, 1 H, H7), 4.18 – 4.07 (m, 2 H, H8), 2.26 (s, 3 H, H17).
^{13}C NMR (150 MHz, $CDCl_3$): δ 205.0 – 204.9 (br, C6), 169.3 (C16), 165.8 (C9), 148.1 (C15), 132.8 (C4), 132.0 (C13), 131.9 (C2), 129.7 (C11), 128.6 (C3), 128.3 (C10), 126.4 (C12), 123.3 (C14), 121.2 (C1), 96.9 (C5), 93.2 (C7), 38.3 (C8), 21.2 (C17).
FTIR (ν_{max}, cm^{-1}): 3309 (w), 3073 (w), 2929 (w), 1953 (w), 1767 (m), 1649 (m), 1608 (m), 1522 (m), 1487 (m), 1448 (w), 1430 (w), 1368 (m), 1295 (m), 1194 (s), 1099 (w), 1070 (w), 1010 (m), 951 (w), 913 (w), 875 (w), 833 (m), 787 (w), 752 (w).
HRMS (ESI): calculated for $C_{19}H_{16}NO_3BrNa$ $[M+Na]^+$ 408.0206, found 408.0213.
$\boldsymbol{R_f}$ = 0.22 (65% Et_2O/hexane).
$[\alpha]_D^{25.0}$ = -194.2 ($CHCl_3$, c = 0.5, 96% *ee*).
HPLC: ChiralART SA, 93:7 hexane/isopropanol, 1.0 mL/min flow rate, T = 25 °C, λ_{max} = 254 nm; t_R (min) = 26.2 (minor), 28.6 (major).

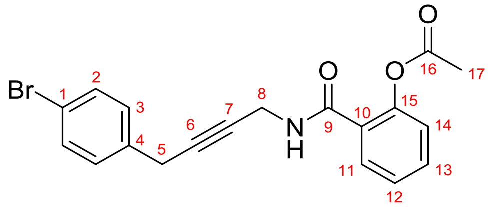

2-((4-(4-bromophenyl)but-2-yn-1-yl)carbamoyl)phenyl acetate (4y'): Isolated as the alkyne cross-product from asymmetric allenylation of 2-(prop-2-yn-1-ylcarbamoyl)phenyl acetate and (4-bromobenzylidene)hydrazine, which provided the title compound as an orange amorphous solid (29.4 mg, 0.076 mmol, 38%), m.p. 105-107 °C.

^{1}H NMR (600 MHz, CDCl$_3$): δ 7.80 (dd, *J* = 7.9, 1.6 Hz, 1 H, H11), 7.48 (td, *J* = 7.9, 1.6 Hz, 1 H, H13), 7.44 (d, *J* = 8.4 Hz, 2 H, H2), 7.31 (td, *J* = 7.9, 1.0 Hz, 1 H, H12), 7.20 (d, *J* = 8.4 Hz, 2 H, H3), 7.11 (dd, *J* = 7.9, 1.0 Hz, 1 H, H14), 6.49 (br s, 1 H, NH), 4.25 (dt, *J* = 4.8, 2.3 Hz, 2 H, H8), 3.56 (t, *J* = 2.3 Hz, 2 H, H5), 2.25 (s, 3 H, H17).
^{13}C NMR (150 MHz, CDCl$_3$): δ 169.1 (C16), 165.1 (C9), 148.1 (C15), 135.5 (C4), 132.2 (C13), 131.8 (C2), 130.2 (C11), 129.8 (C3), 127.7 (C10), 126.5 (C12), 123.4 (C14), 120.7 (C1), 81.3 (C6), 78.1 (C7), 30.3 (C8), 24.7 (C5), 21.0 (C17).
FTIR (ν_{max}, cm^{-1}): 3303 (w), 3064 (w), 2929 (w), 1768 (m), 1650 (m), 1608 (m), 1520 (m), 1486 (m), 1448 (w), 1421 (w), 1368 (m), 1292 (m), 1194 (s), 1099 (m), 1071 (w), 1045 (w), 1012 (m), 978 (w), 954 (w), 913 (w), 836 (m), 799 (m), 751 (m).
HRMS (ESI): calculated for $C_{19}H_{16}NO_3BrNa$ [M+Na]$^+$ 408.0206, found 408.0210.
R$_f$ = 0.27 (65% Et_2O/hexane).

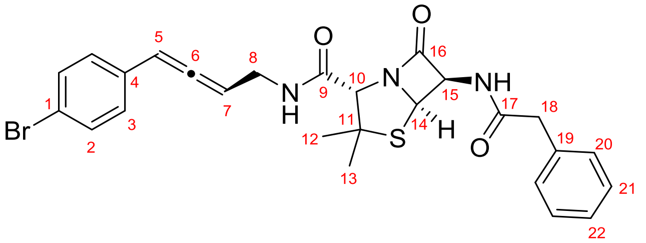

(2*S*,5*R*,6*R*)-*N*-((*R*)-4-(4-bromophenyl)buta-2,3-dien-1-yl)-3,3-dimethyl-7-oxo-6-(2-phenylacetamido)-4-thia-1-azabicyclo[3.2.0]heptane-2-carboxamide (4z): Following the general procedure for asymmetric allenylation using (2*S*,5*R*,6*R*)-3,3-dimethyl-7-oxo-6-(2-phenylacetamido)-*N*-(prop-2-yn-1-yl)-4-thia-1-azabicyclo[3.2.0]heptane-2-carboxamide (74.3 mg, 0.2 mmol) and (4-bromobenzylidene)hydrazine, stirred for 20 min after addition of diazo compound, purified by silica gel column chromatography (eluent: 90% Et_2O/hexane) provided the title compound as an off-white foam (32.0 mg, 0.059 mmol, 30%, 98% *de*). (N.B. The copper acetylide solution is yellow rather than red-orange and there is a delayed onset for the reaction during addition of diazo compound).

^{1}H NMR (600 MHz, CDCl$_3$): δ 7.43 (d, *J* = 8.4 Hz, 2 H, H2), 7.36 (t, *J* = 7.3 Hz, 2 H, H21), 7.31 (t, *J* = 7.3 Hz, 1 H, H22), 7.23 (d, *J* = 7.3 Hz, 2 H, H20), 7.13 (d, *J* = 8.4 Hz, 2 H, H3), 6.59 (br t, *J* = 5.7 Hz, 1 H, C8-NH), 6.23 (dt, *J* = 6.3, 3.2 Hz, 1 H, H5), 6.03 (br d, *J* = 9.2 Hz, 1 H, C15-NH), 5.66 – 5.59 (m, 2 H, H7 and H15), 5.19 (d, *J* = 4.5 Hz, 1 H, H14), 4.02 (s, 1 H, H10), 4.02 – 3.97 (m, 1 H, H8a), 3.94 – 3.87 (m, 1 H, H8b), 3.63 – 3.56 (m, 2 H, H18), 1.66 (s, 3 H, H12/H13), 1.46 (s, 3 H, H12/H13).
^{13}C NMR (150 MHz, CDCl$_3$): δ 204.9 (C6), 176.4 (C16), 170.5 (C17), 167.2 (C9), 133.7 (C19), 132.6 (C4), 132.0 (C2), 129.5 (C20), 129.3 (C21), 128.5 (C3), 127.9 (C22), 121.4

(C1), 97.3 (C5), 93.1 (C7), 72.8 (C10), 66.2 (C14), 64.7 (C11), 57.2 (C15), 43.4 (C18), 37.6 (C8), 28.4 (C12/C13), 26.7 (C12/C13).
FTIR (ν_{max}, cm^{-1}): 3294 (w), 3031 (w), 2964 (w), 1956 (w), 1782 (m), 1655 (s), 1598 (w), 1508 (m), 1489 (m), 1455 (w), 1431 (w), 1390 (w), 1368 (w), 1288 (m), 1234 (w), 1159 (w), 1129 (w), 1102 (w), 1070 (m), 1031 (w), 1010 (m), 909 (m), 833 (s).
HRMS (ESI): calculated for $C_{26}H_{26}N_3O_3SBrNa$ $[M+Na]^+$ 562.0770, found 562.0782.
R_f = 0.24 (90% Et_2O/hexane).
$[\alpha]_D^{25.0}$ = +2.2 ($CHCl_3$, c = 0.2, 98% *de*).
HPLC: ChiralART SC, 85:15 hexane/isopropanol, 1.0 mL/min flow rate, T = 25 °C, λ_{max} = 254 nm; t_R (min) = 30.3 (major), 33.3 (minor).

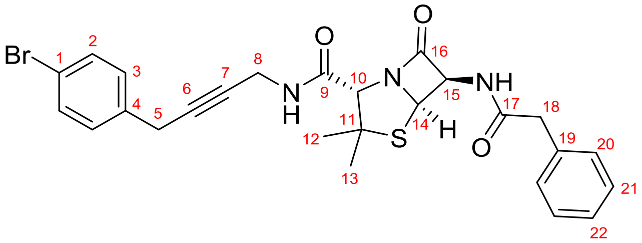

(2*S*,5*R*,6*R*)-*N*-(4-(4-bromophenyl)but-2-yn-1-yl)-3,3-dimethyl-7-oxo-6-(2-phenylacetamido)-4-thia-1-azabicyclo[3.2.0]heptane-2-carboxamide (4z’): Isolated as the alkyne cross-product from asymmetric allenylation of (2*S*,5*R*,6*R*)-3,3-dimethyl-7-oxo-6-(2-phenylacetamido)-*N*-(prop-2-yn-1-yl)-4-thia-1-azabicyclo[3.2.0]heptane-2-carboxamide and (4-bromobenzylidene)hydrazine, which provided the title compound as an orange foam (30.3 mg, 0.056 mmol, 28%).

^{1}H NMR (600 MHz, $CDCl_3$): δ 7.43 (d, *J* = 8.4 Hz, 2 H, H2), 7.38 (t, *J* = 7.4 Hz, 2 H, H21), 7.32 (t, *J* = 7.4 Hz, 1 H, H22), 7.25 (d, *J* = 7.4 Hz, 2 H, H20), 7.18 (d, *J* = 8.4 Hz, 2 H, H3), 6.59 (br t, *J* = 5.2 Hz, 1 H, C8-NH), 6.11 (br d, *J* = 9.3 Hz, 1 H, C15-NH), 5.77 (dd, *J* = 9.3, 4.5 Hz, 1 H, H15), 5.35 (d, *J* = 4.5 Hz, 1 H, H14), 4.14 – 4.08 (m, 2 H, H8a and H10), 4.00 (ddt, *J* = 17.5, 4.8, 2.2 Hz, 1 H, H8b), 3.66 – 3.58 (m, 2 H, H18), 3.53 (t, *J* = 2.2 Hz, 2 H, H5), 1.69 (s, 3 H, H12/H13), 1.47 (s, 3 H, H12/H13).
^{13}C NMR (150 MHz, $CDCl_3$): δ 176.6 (C16), 170.6 (C17), 167.1 (C9), 135.4 (C4), 133.7 (C19), 131.8 (C2), 129.7 (C3), 129.5 (C20), 129.3 (C21), 127.9 (C22), 120.7 (C1), 81.3 (C6), 77.6 (C7), 72.6 (C10), 66.3 (C14), 64.9 (C11), 57.3 (C15), 43.4 (C18), 29.6 (C8), 28.3 (C12/C13), 26.6 (C12/C13), 24.7 (C5).
FTIR (ν_{max}, cm^{-1}): 3295 (w), 3061 (w), 2967 (w), 1785 (m), 1657 (s), 1520 (m), 1488 (m), 1455 (w), 1420 (w), 1293 (w), 1238 (w), 1129 (w), 1071 (w), 1030 (w), 1012 (w), 910 (w), 841 (w).
HRMS (ESI): calculated for $C_{26}H_{26}N_3O_3SBrNa$ $[M+Na]^+$ 562.0770, found 562.0774.
R_f = 0.30 (90% Et_2O/hexane).
$[\alpha]_D^{25.0}$ = +145.0 ($CHCl_3$, c = 0.2).

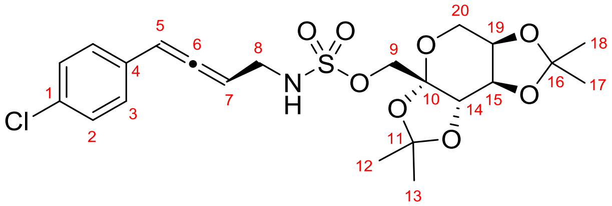

((3a*S*,5a*R*,8a*R*,8b*S*)-2,2,7,7-tetramethyltetrahydro-3a*H*-bis([1,3]dioxolo)[4,5-*b*:4',5'-*d*]pyran-3a-yl)methyl ((*R*)-4-(4-chlorophenyl)buta-2,3-dien-1-yl)sulfamate (4aa):

Following the general procedure for asymmetric allenylation using ((3a*S*,5a*R*,8a*R*,8b*S*)-2,2,7,7-tetramethyltetrahydro-3a*H*-bis([1,3]dioxolo)[4,5-*b*:4',5'-*d*]pyran-3a-yl)methyl prop-2-yn-1-ylsulfamate (75.5 mg, 0.2 mmol) and (4-chlorobenzylidene)hydrazine, purified by silica gel column chromatography (eluent: 60% Et_2O/hexane) provided a nearly inseparable mixture of the title product and the alkyne cross-product as a colourless gum (96.5 mg containing 56.8 mg of the allene, 0.113 mmol, 57%, 96% *de*). An analytical sample of the allene could be obtained by repeated purification by silica gel column chromatography (eluent: 50% Et_2O/hexane).

^{1}H NMR (600 MHz, $CDCl_3$): δ 7.28 (d, J = 8.5 Hz, 2 H, H2), 7.21 (d, J = 8.5 Hz, 2 H, H3), 6.31 (dt, J = 6.1, 3.0 Hz, 1 H, H5), 5.74 (q, J = 6.1 Hz, 1 H, H7), 4.70 (br t, J = 5.9 Hz, 1 H, NH), 4.61 (dd, J = 7.9, 2.6 Hz, 1 H, H15), 4.32 (d, J = 2.6 Hz, 1 H, H14), 4.25 – 4.23 (m, 1 H, H19), 4.22 (d, J = 10.5 Hz, 1 H, H9a), 4.15 (d, J = 10.5 Hz, 1 H, H9b), 3.91 – 3.86 (m, 3 H, H8 and H20a), 3.76 (d, J = 13.0 Hz, 1 H, H20b), 1.53 (s, 3 H, H12/H13), 1.47 (s, 3 H, H17/H18), 1.41 (s, 3 H, H12/H13), 1.34 (s, 3 H, H17/H18).
^{13}C NMR (150 MHz, $CDCl_3$): δ 205.1 (C6), 133.4 (C1), 131.8 (C4), 129.1 (C2), 128.3 (C3), 109.40 (C11), 109.36 (C16), 101.0 (C10), 97.3 (C5), 92.3 (C7), 70.7 (C14), 70.6 (C9), 70.5 (C19), 70.0 (C15), 61.5 (C20), 42.3 (C8), 26.6 (C12/C13), 26.0 (C17/C18), 25.3 (C12/C13), 24.1 (C17/C18).
FTIR (ν_{max}, cm^{-1}): 3286 (br w), 2991 (w), 2937 (w), 1954 (w), 1492 (w), 1455 (w), 1371 (m), 1319 (w), 1252 (m), 1205 (m), 1172 (s), 1070 (s), 1012 (s), 980 (m), 912 (m), 885 (s), 865 (s), 833 (s), 803 (s), 755 (s).
HRMS (ESI): calculated for $C_{22}H_{28}NO_8SClNa$ $[M+Na]^+$ 524.1116, found 524.1123.
R_f = 0.38 (60% Et_2O/hexane).
$[\alpha]_D^{25.0}$ = -124.6 ($CHCl_3$, c = 1.0, 96% *de*).
HPLC: ChiralART SC, 95:5 hexane/isopropanol, 1.0 mL/min flow rate, T = 25 °C, λ_{max} = 254 nm; t_R (min) = 32.5 (minor), 34.4 (major).

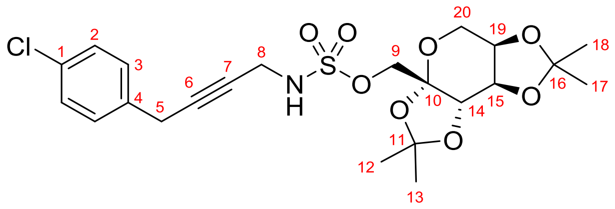

((3a*S*,5a*R*,8a*R*,8b*S*)-2,2,7,7-tetramethyltetrahydro-3a*H*-bis([1,3]dioxolo)[4,5-*b*:4',5'-*d*]pyran-3a-yl)methyl (4-(4-chlorophenyl)but-2-yn-1-yl)sulfamate (4aa'): Isolated as the alkyne cross-product from asymmetric allenylation of ((3a*S*,5a*R*,8a*R*,8b*S*)-2,2,7,7-tetramethyltetrahydro-3a*H*-bis([1,3]dioxolo)[4,5-*b*:4',5'-*d*]pyran-3a-yl)methyl prop-2-yn-1-ylsulfamate and (4-chlorobenzylidene)hydrazine as part of the colourless gum (96.5 mg containing 39.7 mg of the alkyne, 0.079 mmol, 40%). An analytical sample of the alkyne could be obtained by purification by $AgNO_3$-impregnated silica gel (10% w/w $AgNO_3/SiO_2$, eluent: 50% Et_2O/hexane). (N.B. The allene decomposes during purification with $AgNO_3$-impregnated silica gel).

^{1}H NMR (600 MHz, $CDCl_3$): δ 7.29 (d, J = 8.4 Hz, 2 H, H2), 7.24 (d, J = 8.4 Hz, 2 H, H3), 4.72 (br t, J = 5.7 Hz, 1 H, NH), 4.59 (dd, J = 7.9, 2.6 Hz, 1 H, H15), 4.32 (d, J = 2.6 Hz, 1 H, H14), 4.25 (d, J = 10.5 Hz, 1 H, H9a), 4.24 – 4.22 (m, 1 H, H19), 4.17 (d, J = 10.5 Hz, 1 H, H9b), 4.01 – 3.98 (m, 2 H, H8), 3.89 (dd, J = 13.0, 1.8 Hz, 1 H, H20a), 3.76 (d, J = 13.0 Hz, 1 H, H20b), 3.58 (t, J = 2.0 Hz, 2 H, H5), 1.53 (s, 3 H, H12/H13), 1.47 (s, 3 H, H17/H18), 1.41 (s, 3 H, H12/H13), 1.34 (s, 3 H, H17/H18).

^{13}C NMR (150 MHz, CDCl$_3$): δ 134.6 (C4), 132.8 (C1), 129.4 (C3), 128.9 (C2), 109.40 (C11), 109.36 (C16), 101.0 (C10), 83.1 (C6), 76.7 (C7), 70.8 (C9), 70.7 (C19), 70.5 (C14), 70.0 (C15), 61.5 (C20), 34.2 (C8), 26.6 (C12/C13), 26.0 (C17/C18), 25.3 (C12/C13), 24.6 (C5), 24.2 (C17/C18).
FTIR (ν_{max}, cm^{-1}): 3288 (w), 2989 (w), 2937 (w), 1492 (w), 1456 (w), 1372 (m), 1319 (w), 1252 (m), 1206 (m), 1176 (s), 1101 (m), 1070 (s), 1014 (s), 948 (w), 913 (m), 885 (m), 865 (m), 831 (m), 804 (m), 754 (s).
HRMS (ESI): calculated for $C_{22}H_{28}NO_8SClNa$ [M+Na]$^+$ 524.1116, found 524.1122.
$\boldsymbol{R_f}$ = 0.39 (60% Et_2O/hexane).
$[\alpha]_D^{29.5}$ = -34.8 ($CHCl_3$, c = 1.0).

(**Procedure for generation of $AgNO_3$-impregnated silica gel:** To a solution of $AgNO_3$ (2.0 g) in 75% aqueous EtOH (80 mL) in an amber glass 100 mL RBF was added silica gel (20 g, 230-400 mesh, pore size 60 Å). The mixture was sonicated for 10 min with manual stirring. The solvent was then removed under reduced pressure on a rotary evaporator (60 °C water bath temperature, 40 mmHg), then dried at 140 °C for 2 h.)

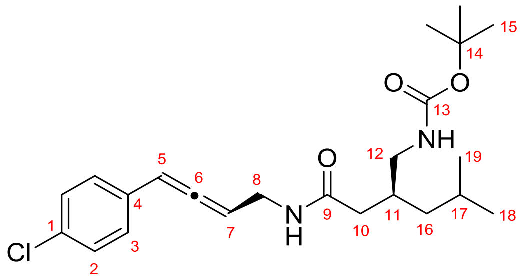

***tert*-butyl ((*S*)-2-(2-(((*R*)-4-(4-chlorophenyl)buta-2,3-dien-1-yl)amino)-2-oxoethyl)-4-methylpentyl)carbamate (4ab):** Following the general procedure for asymmetric allenylation using *tert*-butyl (*S*)-(4-methyl-2-(2-oxo-2-(prop-2-yn-1-ylamino)ethyl)pentyl)carbamate (59.3 mg, 0.2 mmol) and (4-chlorobenzylidene)hydrazine, purified by silica gel column chromatography (eluent: 10% Et_2O/CH_2Cl_2) provided the title compound as a colourless gum (38.7 mg, 0.092 mmol, 46%, 96% *de*).

^{1}H NMR (600 MHz, CDCl$_3$): δ 7.25 (d, *J* = 8.6 Hz, 2 H, H2), 7.20 (d, *J* = 8.6 Hz, 2 H, H3), 6.87 (br s, 1 H, C8-NH), 6.23 (dt, *J* = 6.4, 3.2 Hz, 1 H, H5), 5.68 (q, *J* = 6.4 Hz, 1 H, H7), 4.79 (br s, 1 H, C12-NH), 4.07 – 4.00 (m, 1 H, H8a), 3.97 – 3.89 (m, 1 H, H8b), 3.19 (ddd, *J* = 14.2, 6.6, 3.9 Hz, 1 H, H12a), 2.96 (dt, *J* = 14.2, 6.6 Hz, 1 H, H12b), 2.12 – 2.03 (m, 2 H, H10), 2.01 – 1.93 (m, 1 H, H11), 1.62 – 1.55 (m, 1 H, H17), 1.42 (s, 9 H, H15), 1.10 – 1.05 (m, 2 H, H16), 0.87 – 0.81 (m, 6 H, H18 and H19).
^{13}C NMR (150 MHz, CDCl$_3$): δ 204.9 (C6), 172.4 (C9), 157.1 (C13), 132.9 (C1), 132.7 (C4), 128.9 (C2), 128.2 (C3), 96.6 (C5), 93.7 (C7), 79.6 (C14), 43.5 (C12), 41.7 (C16), 39.4 (C10), 37.9 (C8), 34.6 (C11), 28.5 (C15), 25.3 (C17), 22.8 (C18/C19), 22.7 (C18/C19).
FTIR (ν_{max}, cm^{-1}): 3300 (m), 2957 (m), 2930 (m), 2871 (m), 1953 (w), 1691 (s), 1648 (s), 1535 (s), 1492 (s), 1453 (m), 1391 (m), 1366 (m), 1251 (m), 1169 (s), 1091 (m), 1014 (w), 874 (w), 834 (m).
HRMS (ESI): calculated for $C_{23}H_{34}N_2O_3Cl$ [M+H]$^+$ 421.2252, found 421.2255.
$\boldsymbol{R_f}$ = 0.30 (10% Et_2O/CH_2Cl_2).
$[\alpha]_D^{25.0}$ = -175.2 ($CHCl_3$, c = 1.0, 96% *de*).
HPLC: ChiralART SA, 95:5 hexane/isopropanol, 1.0 mL/min flow rate, T = 25 °C, λ_{max} = 254 nm; t_R (min) = 15.9 (minor), 16.8 (major).

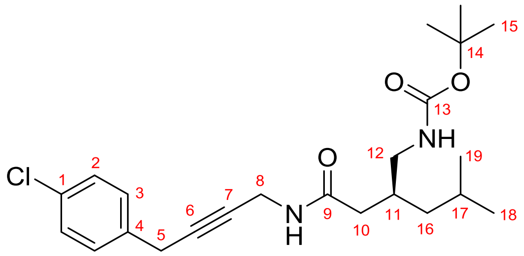

***tert*-butyl (*S*)-(2-(2-((4-(4-chlorophenyl)but-2-yn-1-yl)amino)-2-oxoethyl)-4-methylpentyl)carbamate (4ab'):** Isolated as the alkyne cross-product from asymmetric allenylation of *tert*-butyl (*S*)-(4-methyl-2-(2-oxo-2-(prop-2-yn-1-ylamino)ethyl)pentyl)carbamate and (4-chlorobenzylidene)hydrazine, which provided the title compound as a colourless gum (34.6 mg, 0.082 mmol, 41%).

^{1}H NMR (600 MHz, CDCl$_3$): δ 7.28 – 7.24 (m, 4 H, H2 and H3), 6.90 (br s, 1 H, C8-NH), 4.84 (br s, 1 H, C12-NH), 4.13 – 4.03 (m, 2 H, H8), 3.55 (t, *J* = 2.0 Hz, 2 H, H5), 3.23 (ddd, *J* = 14.2, 6.5, 3.9 Hz, 1 H, H12a), 3.00 (dt, *J* = 14.2, 6.5 Hz, 1 H, H12b), 2.17 – 2.09 (m, 2 H, H10), 2.07 – 1.99 (m, 1 H, H11), 1.68 – 1.60 (m, 1 H, H17), 1.43 (s, 9 H, H15), 1.13 (t, *J* = 7.1 Hz, 2 H, H16), 0.90 – 0.87 (m, 6 H, H18 and H19).
^{13}C NMR (150 MHz, CDCl$_3$): δ 172.3 (C9), 157.1 (C13), 135.2 (C4), 132.5 (C1), 129.4 (C3), 128.7 (C2), 80.5 (C6), 79.7 (C14), 78.6 (C7), 43.5 (C12), 41.7 (C16), 39.2 (C10), 34.6 (C11), 29.7 (C8), 28.5 (C15), 25.3 (C17), 24.6 (C5), 22.9 (C18/C19), 22.8 (C18/C19).
FTIR (ν_{max}, cm^{-1}): 3323 (m), 2958 (m), 2930 (m), 1691 (s), 1649 (s), 1531 (s), 1492 (s), 1453 (m), 1391 (m), 1366 (m), 1251 (m), 1169 (s), 1091 (m), 1016 (m), 806 (w).
HRMS (ESI): calculated for $C_{23}H_{33}N_2O_3ClNa$ [M+Na]$^+$ 443.2072, found 443.2069.
$\boldsymbol{R_f}$ = 0.39 (10% Et_2O/CH_2Cl_2).
$[\alpha]_D^{25.0}$ = -12.2 ($CHCl_3$, c = 1.0).

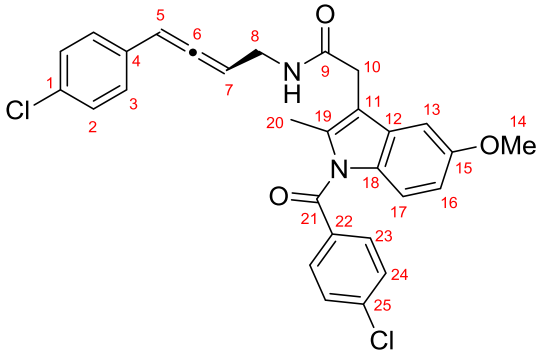

(*R*)-2-(1-(4-chlorobenzoyl)-5-methoxy-2-methyl-1*H*-indol-3-yl)-*N*-(4-(4-chlorophenyl)-buta-2,3-dien-1-yl)acetamide (4ac): Following the general procedure for asymmetric allenylation using 2-(1-(4-chlorobenzoyl)-5-methoxy-2-methyl-1*H*-indol-3-yl)-*N*-(prop-2-yn-1-yl)acetamide (79.0 mg, 0.2 mmol) and (4-chlorobenzylidene)hydrazine, purified by silica gel column chromatography (eluent: 5% Et_2O/CH_2Cl_2) provided the title compound as a yellow foam (41.8 mg, 0.080 mmol, 40%, 95% *ee*). (N.B. The starting material is sparingly soluble in reaction mixture at the start and thus the reaction mixture appears as a yellow-orange suspension, but solubilises over time as the diazo compound is added).

^{1}H NMR (600 MHz, CDCl$_3$): δ 7.57 (d, *J* = 8.5 Hz, 2 H, H23), 7.46 (d, *J* = 8.5 Hz, 2 H, H24), 7.16 (d, *J* = 8.4 Hz, 2 H, H2), 6.99 (d, *J* = 8.4 Hz, 2 H, H3), 6.82 (d, *J* = 2.4 Hz, 1 H, H13), 6.73 (d, *J* = 9.0 Hz, 1 H, H17), 6.65 (dd, *J* = 9.0, 2.4 Hz, 1 H, H16), 6.02 (dt, *J* = 6.3,

3.6 Hz, 1 H, H5), 5.80 (br t, J = 5.1 Hz, 1 H, NH), 5.61 (q, J = 6.3 Hz, 1 H, H7), 3.98 – 3.85 (m, 2 H, H8), 3.78 (s, 3 H, H14), 3.60 (AB q, J = 17.4 Hz, 2 H, H10), 2.25 (s, 3 H, H20).
^{13}C NMR (150 MHz, $CDCl_3$): δ 203.8 (C6), 169.8 (C21), 168.2 (C9), 156.3 (C15), 139.7 (C25), 136.5 (C19), 133.5 (C22), 133.0 (C1), 132.0 (C4), 131.3 (C23), 130.9 (C18), 130.3 (C12), 129.3 (C24), 128.9 (C2), 128.0 (C3), 115.2 (C17), 112.6 (C11), 112.2 (C16), 100.9 (C13), 97.4 (C5), 93.4 (C7), 55.9 (C14), 37.5 (C8), 32.2 (C10), 13.2 (C20).
FTIR (ν_{max}, cm^{-1}): 3304 (w), 3062 (w), 2930 (w), 2834 (w), 1953 (w), 1674 (s), 1651 (s), 1591 (m), 1521 (m), 1490 (m), 1477 (s), 1456 (m), 1436 (m), 1401 (w), 1357 (s), 1317 (s), 1289 (m), 1260 (m), 1223 (s), 1179 (m), 1149 (m), 1089 (s), 1070 (m), 1037 (m), 1014 (m), 993 (w), 925 (m), 909 (m), 877 (w), 832 (s), 754 (m).
HRMS (ESI): calculated for $C_{29}H_{24}N_2O_3Cl_2Na$ $[M+Na]^+$ 541.1056, found 541.1065.
R_f = 0.27 (5% Et_2O/CH_2Cl_2).
$[\alpha]_D^{25.0}$ = -171.8 ($CHCl_3$, c = 1.0, 95% *ee*).
HPLC: ChiralART SA, 85:15 hexane/isopropanol, 1.0 mL/min flow rate, T = 25 °C, λ_{max} = 254 nm; t_R (min) = 21.2 (major), 30.1 (minor).

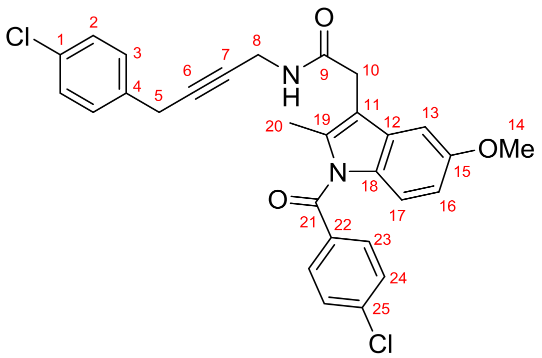

2-(1-(4-chlorobenzoyl)-5-methoxy-2-methyl-1*H*-indol-3-yl)-*N*-(4-(4-chlorophenyl)but-2-yn-1-yl)acetamide (4ac'): Isolated as the alkyne cross-product from asymmetric allenylation of 2-(1-(4-chlorobenzoyl)-5-methoxy-2-methyl-1*H*-indol-3-yl)-*N*-(prop-2-yn-1-yl)acetamide and (4-chlorobenzylidene)hydrazine, which provided the title compound as a pale yellow amorphous solid (39.6 mg, 0.076 mmol, 38%), m.p. 195-197 °C.

^{1}H NMR (600 MHz, $CDCl_3$): δ 7.66 (d, J = 8.6 Hz, 2 H, H23), 7.48 (d, J = 8.6 Hz, 2 H, H24), 7.24 (d, J = 8.5 Hz, 2 H, H2), 7.16 (d, J = 8.5 Hz, 2 H, H3), 6.88 (d, J = 2.5 Hz, 1 H, H13), 6.86 (d, J = 9.1 Hz, 1 H, H17), 6.71 (dd, J = 9.1, 2.5 Hz, 1 H, H16), 5.74 (br t, J = 5.3 Hz, 1 H, NH), 4.05 (dt, J = 5.3, 2.2 Hz, 2 H, H8), 3.79 (s, 3 H, H14), 3.66 (s, 2 H, H10), 3.49 (t, J = 2.2 Hz, 2 H, H5), 2.38 (s, 3 H, H20).
^{13}C NMR (150 MHz, $CDCl_3$): δ 169.6 (C21), 168.5 (C9), 156.4 (C15), 139.8 (C25), 136.6 (C19), 134.9 (C4), 133.6 (C22), 132.6 (C1), 131.3 (C23), 131.0 (C18), 130.3 (C12), 129.4 (C24), 129.3 (C3), 128.8 (C2), 115.2 (C17), 112.54 (C11), 112.46 (C16), 101.0 (C13), 80.9 (C6), 78.1 (C7), 55.9 (C14), 32.2 (C10), 29.8 (C8), 24.6 (C5), 13.4 (C20).
FTIR (ν_{max}, cm^{-1}): 3296 (m), 2930 (w), 1670 (m), 1628 (s), 1538 (m), 1490 (m), 1477 (m), 1438 (m), 1400 (m), 1361 (s), 1326 (s), 1288 (m), 1259 (m), 1221 (s), 1177 (w), 1151 (m), 1089 (s), 1073 (m), 1035 (m), 1013 (m), 992 (w), 914 (w), 833 (m), 792 (m), 754 (m).
HRMS (ESI): calculated for $C_{29}H_{24}N_2O_3Cl_2Na$ $[M+Na]^+$ 541.1056, found 541.1067.
R_f = 0.41 (5% Et_2O/CH_2Cl_2).

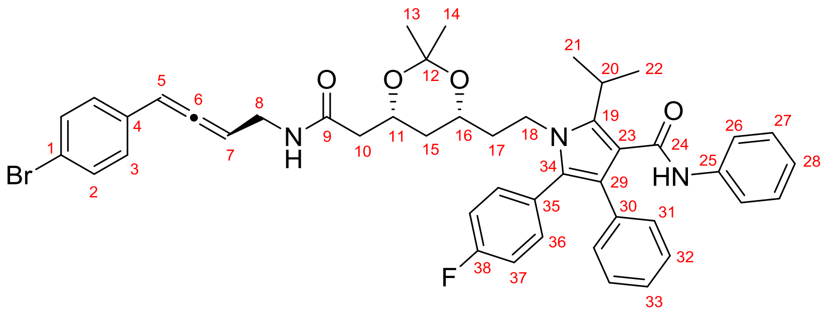

1-(2-((4*R*,6*R*)-6-(2-(((*R*)-4-(4-bromophenyl)buta-2,3-dien-1-yl)amino)-2-oxoethyl)-2,2-dimethyl-1,3-dioxan-4-yl)ethyl)-5-(4-fluorophenyl)-2-isopropyl-*N*,4-diphenyl-1*H*-pyrrole-3-carboxamide (4ad): Following the general procedure for asymmetric allenylation using 1-(2-((4*R*,6*R*)-2,2-dimethyl-6-(2-oxo-2-(prop-2-yn-1-ylamino)ethyl)-1,3-dioxan-4-yl)ethyl)-5-(4-fluorophenyl)-2-isopropyl-*N*,4-diphenyl-1*H*-pyrrole-3-carboxamide (127.2 mg, 0.2 mmol) and (4-bromobenzylidene)hydrazine, purified by silica gel column chromatography (eluent: 85% Et_2O/hexane) provided the title compound as an off-white foam (64.8 mg, 0.081 mmol, 40%, 94% *de*).

^{1}H NMR (600 MHz, $CDCl_3$): δ 7.41 (d, *J* = 8.4 Hz, 2 H, H2), 7.22 – 7.14 (m, 9 H, H27, H31, H32, H33 and H36), 7.13 (d, *J* = 8.4 Hz, 2 H, H3), 7.07 (d, *J* = 7.9 Hz, 2 H, H26), 7.01 – 6.96 (m, 3 H, H28 and H37), 6.87 (br s, 1 H, C24-NH), 6.29 (br t, *J* = 5.7 Hz, 1 H, C8-NH), 6.21 (dt, *J* = 6.4, 3.2 Hz, 1 H, H5), 5.65 (q, *J* = 6.4 Hz, 1 H, H7), 4.09 – 3.99 (m, 3 H, H8a, H11 and H18a), 3.92 – 3.86 (m, 1 H, H8b), 3.84 – 3.77 (m, 1 H, H18b), 3.65 – 3.59 (m, 1 H, H16), 3.59 – 3.53 (m, 1 H, H20), 2.32 (dd, *J* = 15.1, 7.6 Hz, 1 H, H10a), 2.23 (dd, *J* = 15.1, 4.1 Hz, 1 H, H10b), 1.69 – 1.59 (m, 2 H, H17), 1.52 (superimposed d, *J* = 7.1 Hz, 6 H, H21 and H22), 1.30 – 1.27 (s superimposed on m, 4 H, H13/H14 and H15a), 1.26 (s, 3 H, H13/H14), 1.04 (dt, *J* = 12.8, 11.7 Hz, 1 H, H15b).
^{13}C NMR (150 MHz, $CDCl_3$): δ 204.7 (C6), 170.4 (C9), 164.9 (C24), 162.4 (d, *J* = 247.9 Hz, C38), 141.6 (C19), 138.5 (C25), 134.7 (C30), 133.3 (d, *J* = 8.1 Hz, C36), 132.9 (C4), 131.9 (C2), 130.6 (C31), 128.9 (C34), 128.8 (C32), 128.49 (C3/C27), 128.48 (C3/C27), 128.4 (d, *J* = 3.2 Hz, C35), 126.7 (C33), 123.6 (C28), 121.9 (C29), 121.1 (C1), 119.7 (C26), 115.50 (d, *J* = 21.4 Hz, C37), 115.49 (C23), 99.0 (C12), 96.8 (C5), 93.6 (C7), 66.4 (C16), 66.2 (C11), 43.3 (C10), 40.9 (C18), 38.1 (C17), 37.7 (C8), 36.0 (C15), 30.0 (C13/C14), 26.2 (C20), 21.9 (C21/C22), 21.7 (C21/C22), 19.8 (C13/C14).
^{19}F NMR (376 MHz, $CDCl_3$): δ -113.6 (s, 1 F, F38).
FTIR (ν_{max}, cm^{-1}): 3301 (w), 2961 (w), 1957 (w), 1652 (s), 1595 (m), 1526 (s), 1509 (s), 1488 (s), 1436 (s), 1381 (m), 1313 (s), 1224 (m), 1201 (m), 1170 (m), 1157 (s), 1115 (w), 1070 (w), 1032 (w), 1010 (w), 942 (w), 909 (m), 838 (m), 753 (m).
HRMS (ESI): calculated for $C_{46}H_{48}FN_3O_4Br$ $[M+H]^+$ 804.2807, found 804.2822.
$\boldsymbol{R_f}$ = 0.31 (85% Et_2O/hexane).
$[\alpha]_D^{25.0}$ = -78.7 ($CHCl_3$, c = 1.0, 94% *de*).
HPLC: ChiralART SC, 85:15 hexane/isopropanol, 1.0 mL/min flow rate, T = 25 °C, λ_{max} = 254 nm; t_R (min) = 32.8 (minor), 38.0 (major).

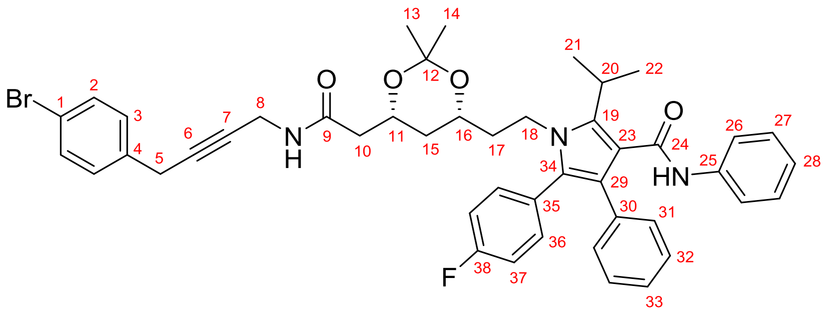

1-(2-((4*R*,6*R*)-6-(2-((4-(4-bromophenyl)but-2-yn-1-yl)amino)-2-oxoethyl)-2,2-dimethyl-1,3-dioxan-4-yl)ethyl)-5-(4-fluorophenyl)-2-isopropyl-*N*,4-diphenyl-1*H*-pyrrole-3-carboxamide (4ad'): Isolated as the alkyne cross-product from asymmetric allenylation of 1-(2-((4*R*,6*R*)-2,2-dimethyl-6-(2-oxo-2-(prop-2-yn-1-ylamino)ethyl)-1,3-dioxan-4-yl)ethyl)-5-(4-fluorophenyl)-2-isopropyl-*N*,4-diphenyl-1*H*-pyrrole-3-carboxamide and (4-bromobenzylidene)hydrazine, which provided the title compound as an orange foam (65.1 mg, 0.081 mmol, 40%).

^{1}H NMR (600 MHz, $CDCl_3$): δ 7.42 (d, *J* = 8.4 Hz, 2 H, H2), 7.21 – 7.13 (m, 11 H, H3, H27, H31, H32, H33 and H36), 7.07 (d, *J* = 7.8 Hz, 2 H, H26), 7.01 – 6.96 (m, 3 H, H28 and H37), 6.87 (br s, 1 H, C24-NH), 6.37 (br t, *J* = 5.0 Hz, 1 H, C8-NH), 4.16 – 4.10 (m, 1 H, H11), 4.10 – 4.00 (m, 3 H, H8 and H18a), 3.86 – 3.78 (m, 1 H, H18b), 3.70 – 3.64 (m, 1 H, H16), 3.60 – 3.54 (m, 1 H, H20), 3.52 (t, *J* = 2.1 Hz, 2 H, H5), 2.33 (dd, *J* = 15.1, 7.6 Hz, 1 H, H10a), 2.26 (dd, *J* = 15.1, 4.0 Hz, 1 H, H10b), 1.70 – 1.60 (m, 2 H, H17), 1.53 (superimposed d, *J* = 7.1 Hz, 6 H, H21 and H22), 1.33 (s, 3 H, H13/H14), 1.32 – 1.27 (s superimposed on m, 4 H, H13/H14 and H15a), 1.07 (dt, *J* = 12.8, 11.7 Hz, 1 H, H15b).
^{13}C NMR (150 MHz, $CDCl_3$): δ 170.0 (C9), 164.9 (C24), 162.4 (d, *J* = 247.9 Hz, C38), 141.6 (C19), 138.5 (C25), 135.6 (C4), 134.7 (C30), 133.3 (d, *J* = 8.1 Hz, C36), 131.7 (C2), 130.6 (C31), 129.7 (C3), 128.9 (C34), 128.8 (C32), 128.5 (C27), 128.4 (d, *J* = 3.3 Hz, C35), 126.7 (C33), 123.6 (C28), 121.9 (C29), 120.6 (C1), 119.7 (C26), 115.49 (d, *J* = 21.4 Hz, C37), 115.48 (C23), 99.0 (C12), 80.7 (C6), 78.3 (C7), 66.5 (C16), 66.2 (C11), 42.9 (C10), 40.9 (C18), 38.0 (C17), 35.9 (C15), 30.0 (C13/C14), 29.6 (C8), 26.2 (C20), 24.7 (C5), 21.9 (C21/C22), 21.7 (C21/C22), 19.9 (C13/C14).
^{19}F NMR (376 MHz, $CDCl_3$): δ -113.6 (s, 1 F, F38).
FTIR (ν_{max}, cm^{-1}): 3301 (w), 2960 (w), 1654 (s), 1596 (m), 1527 (s), 1509 (s), 1488 (s), 1437 (m), 1381 (m), 1314 (m), 1224 (m), 1201 (m), 1157 (m), 1073 (w), 1032 (w), 1013 (m), 942 (w), 910 (m), 886 (w), 841 (m), 805 (w), 754 (m).
HRMS (ESI): calculated for $C_{46}H_{48}FN_3O_4Br$ $[M+H]^+$ 804.2807, found 804.2817.
R_f = 0.40 (85% Et_2O/hexane).
$[\alpha]_D^{25.0}$ = -7.8 ($CHCl_3$, c = 1.0).

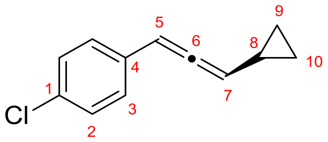

(*R*)-1-chloro-4-(3-cyclopropylpropa-1,2-dien-1-yl)benzene: Following the general procedure for asymmetric allenylation using cyclopropylacetylene (13.2 mg, 0.2 mmol) and (4-chlorobenzylidene)hydrazine, purified by silica gel column chromatography (eluent: hexane) provided the title compound as a colourless oil (11.3 mg, 0.059 mmol, 30%, 87% *ee*). Data are consistent with a reported racemic example.[35]

^{1}H NMR (600 MHz, CDCl$_3$): δ 7.26 (d, J = 8.5 Hz, 2 H, H2), 7.21 (d, J = 8.5 Hz, 2 H, H3), 6.16 (d, J = 6.4 Hz, 1 H, H5), 5.45 (t, 1 H, J = 6.4 Hz, H7), 1.39 – 1.31 (m, 1 H, H8), 0.81 – 0.73 (m, 2 H, H9a and H10a), 0.50 – 0.39 (m, 2 H, H9b and H10b).
^{13}C NMR (150 MHz, CDCl$_3$): δ 205.1 (C6), 133.6 (C4), 132.5 (C1), 128.8 (C2), 128.0 (C3), 100.1 (C7), 95.5 (C5), 9.5 (C8), 7.2 (C9/C10), 7.1 (C9/C10).
FTIR (ν_{max}, cm^{-1}): 3083 (w), 3005 (w), 2923 (w), 1948 (w), 1593 (w), 1573 (w), 1490 (s), 1457 (w), 1427 (w), 1397 (w), 1354 (w), 1296 (w), 1273 (w), 1251 (w), 1198 (w), 1174 (w), 1090 (s), 1047 (w), 1013 (m), 963 (w), 928 (w), 889 (w), 874 (m), 831 (s), 811 (s), 751 (m).
HRMS (ESI): calculated for $C_{12}H_{12}Cl$ $[M+H]^+$ 191.0622, found 191.0620.
R_f = 0.52 (hexane).
$[\alpha]_D^{25.0}$ = -183.9 (CHCl$_3$, c = 1.0, 87% *ee*).
HPLC: Chiralpak OD-H, hexane, 1.0 mL/min flow rate, T = 25 °C, λ_{max} = 254 nm; t_R (min) = 6.7 (minor), 9.9 (major).

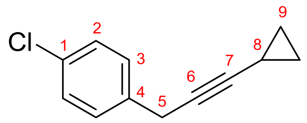

1-chloro-4-(3-cyclopropylprop-2-yn-1-yl)benzene: Isolated as the alkyne cross-product from asymmetric allenylation of cyclopropylacetylene and (4-chlorobenzylidene)hydrazine, which provided the title compound as a colourless oil (26.7 mg, 0.140 mmol, 70%).

^{1}H NMR (600 MHz, CDCl$_3$): δ 7.29 – 7.22 (m, 4 H, H2 and H3), 3.51 (d, J = 2.0 Hz, 2 H, H5), 1.33 – 1.16 (m, 1 H, H8), 0.78 – 0.72 (m, 2 H, H9a), 0.68 – 0.64 (m, 2 H, H9b).
^{13}C NMR (150 MHz, CDCl$_3$): δ 136.1 (C4), 132.3 (C1), 129.3 (C3), 128.6 (C2), 86.2 (C7), 72.5 (C6), 24.7 (C5), 8.2 (C8), -0.3 (C9).
FTIR (ν_{max}, cm^{-1}): 3010 (w), 2921 (w), 2853 (w), 1703 (w), 1596 (w), 1578 (w), 1490 (s), 1421 (m), 1406 (m), 1360 (w), 1291 (w), 1202 (w), 1177 (w), 1088 (m), 1052 (w), 1035 (w), 1016 (s), 914 (w), 886 (m), 798 (s).
HRMS (ESI): calculated for $C_{12}H_{12}Cl$ $[M+H]^+$ 191.0622, found 191.0628.
R_f = 0.24 (hexane).

7. Synthetic procedure and characterisation for silver-mediated cyclisation

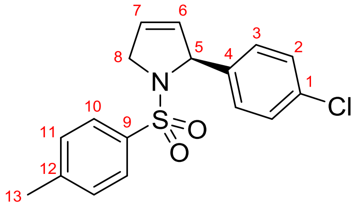

(*S*)-2-(4-chlorophenyl)-1-tosyl-2,5-dihydro-1*H*-pyrrole (5): In the dark, to a solution of (*R*)-*N*-(4-(4-chlorophenyl)buta-2,3-dien-1-yl)-4-methylbenzenesulfonamide (12.3 mg, 0.037 mmol, 1 equiv., 97% *ee*) in CH_2Cl_2 (0.3 mL) was added a solution of $AgPF_6$ (1.0 mg, 0.004 mmol, 0.1 equiv.) in CH_2Cl_2 (0.05 mL). The mixture was stirred at r.t. for 16 h. The reaction mixture was then evaporated under reduced pressure and purified by silica gel column chromatography (eluent: 20% EtOAc/hexane) to provide the title product as a white amorphous solid (12.2 mg, 0.037 mmol, 99%, 95% *ee*), m.p. 131-133 °C. Data is consistent with a reported example.[38]

^{1}H NMR (600 MHz, CDCl$_3$): δ 7.52 (d, *J* = 8.1 Hz, 2 H, H10), 7.24 (d, *J* = 8.4 Hz, 2 H, H2), 7.21 (d, *J* = 8.1 Hz, 2 H, H11), 7.18 (d, *J* = 8.4 Hz, 2 H, H3), 5.81 (dq, *J* = 6.3, 2.0 Hz, 1 H, H6), 5.61 (dq, *J* = 6.3, 2.3 Hz, 1 H, H7), 5.50 – 5.46 (m, 1 H, H5), 4.34 (ddd, *J* = 14.6, 4.7, 2.3 Hz, 1 H, H8a), 4.26 – 4.23 (m, 1 H, H8b), 2.40 (s, 3 H, H13).
^{13}C NMR (150 MHz, CDCl$_3$): δ 143.5 (C12), 139.2 (C4), 135.5 (C9), 133.8 (C1), 130.3 (C7), 129.7 (C11), 128.8 (C3), 128.7 (C2), 127.4 (C10), 125.1 (C6), 69.7 (C5), 55.6 (C8), 21.6 (C13).
FTIR (ν_{max}, cm^{-1}): 2921 (w), 1597 (w), 1491 (w), 1411 (w), 1346 (m), 1305 (w), 1162 (s), 1089 (m), 1059 (w), 1015 (w), 815 (m).
HRMS (ESI): calculated for $C_{17}H_{17}NO_2SCl$ $[M+H]^+$ 334.0663, found 334.0674.
R_f = 0.30 (20% EtOAc/hexane).
$[\alpha]_D^{25.0}$ = -268.2 ($CHCl_3$, c = 0.5, 95% *ee*); lit.[38] $[\alpha]_D^{20}$ = +215.2 ($CHCl_3$, c = 0.955, for opposite enantiomer; *ee* not stated, but at most 91% *ee*).
HPLC: ChiralART SC, 90:10 hexane/isopropanol, 1.0 mL/min flow rate, T = 25 °C, λ_{max} = 210 nm; t_R (min) = 33.7 (minor), 36.7 (major).

8. NMR spectra

8.1. NMR spectra for PyBOX ligands

2,6-bis((*S*)-4-methyl-4,5-dihydrooxazol-2-yl)pyridine:

^{1}H NMR, 600 MHz, $CDCl_3$:

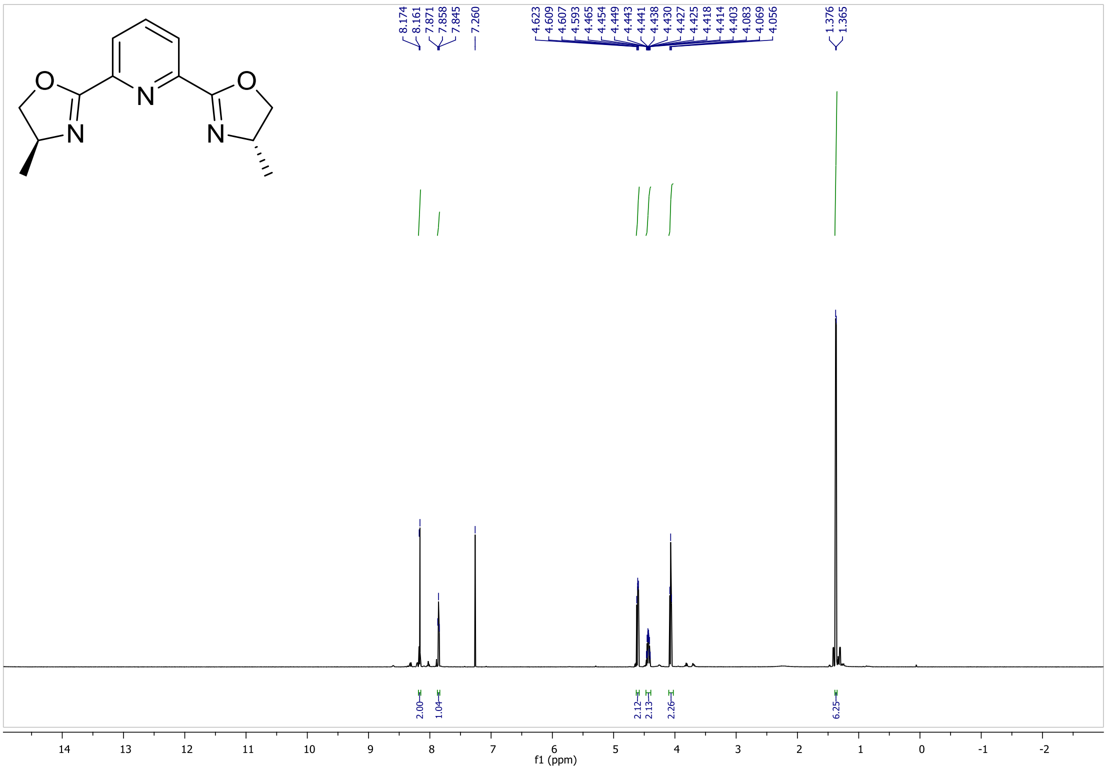

^{13}C NMR, 150 MHz, $CDCl_3$:

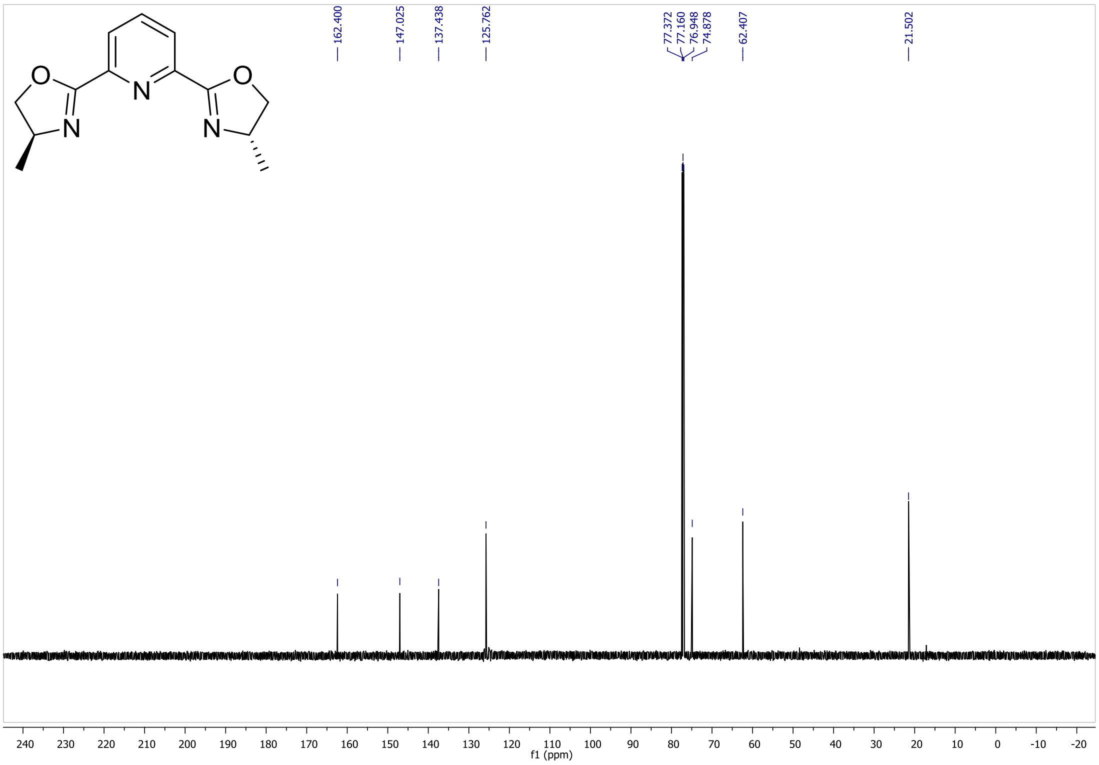

2,6-bis((S)-4-(*tert*-butyl)-4,5-dihydrooxazol-2-yl)pyridine (L2):

^{1}H NMR, 600 MHz, CDCl$_3$:

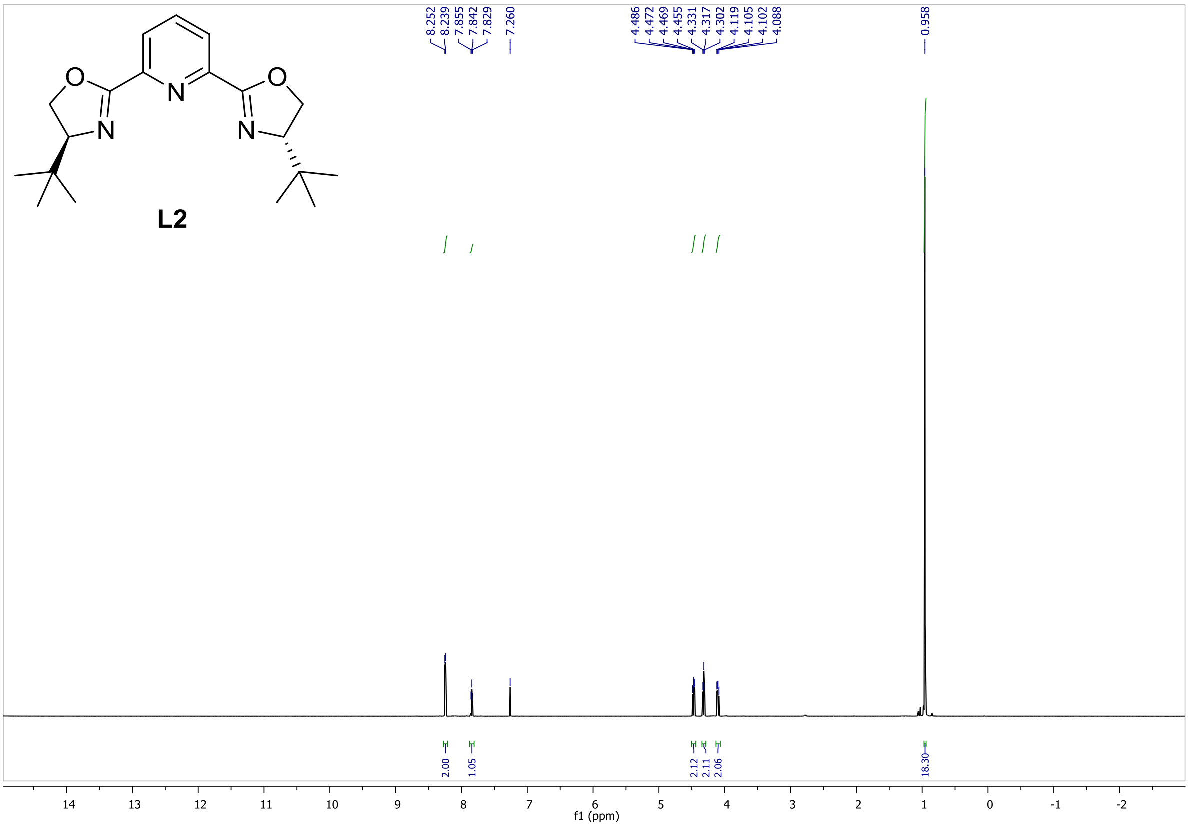

^{13}C NMR, 150 MHz, CDCl$_3$:

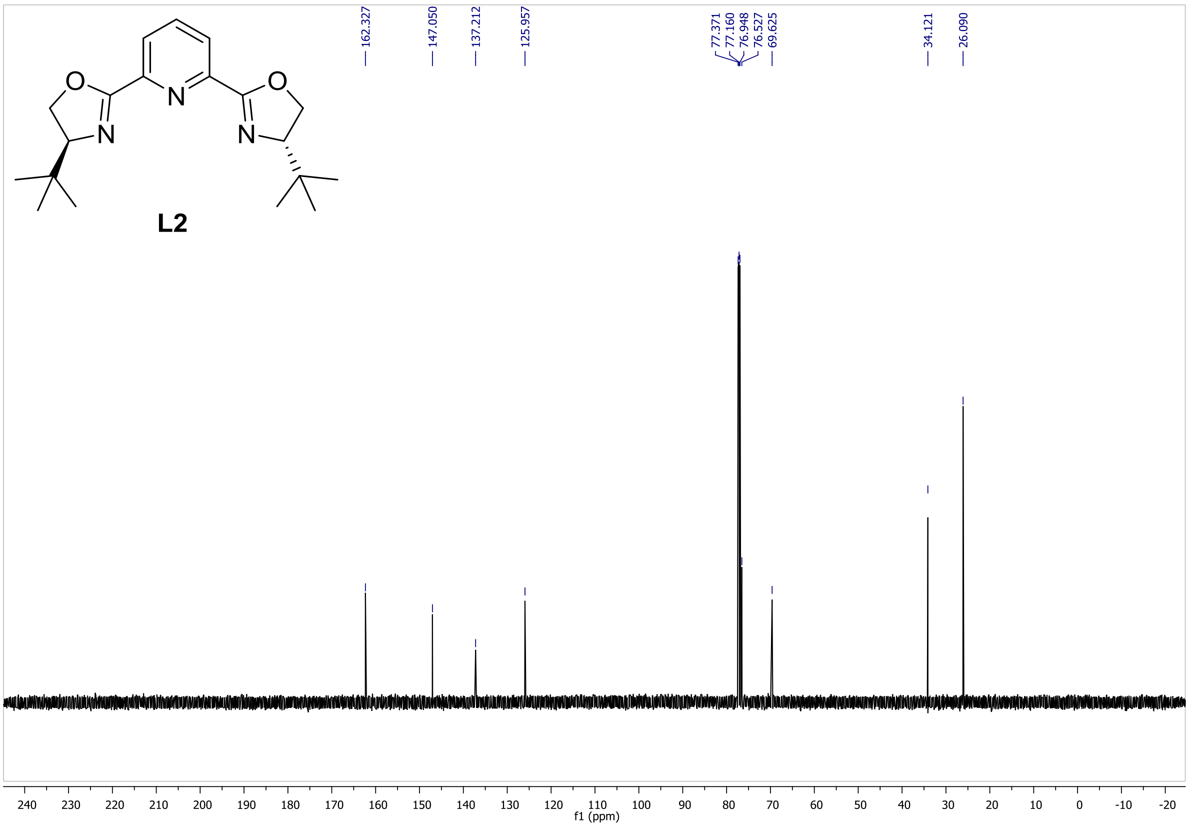

4-(trifluoromethyl)pyridine-2,6-dicarbonitrile:

^{1}H NMR, 600 MHz, $CDCl_3$:

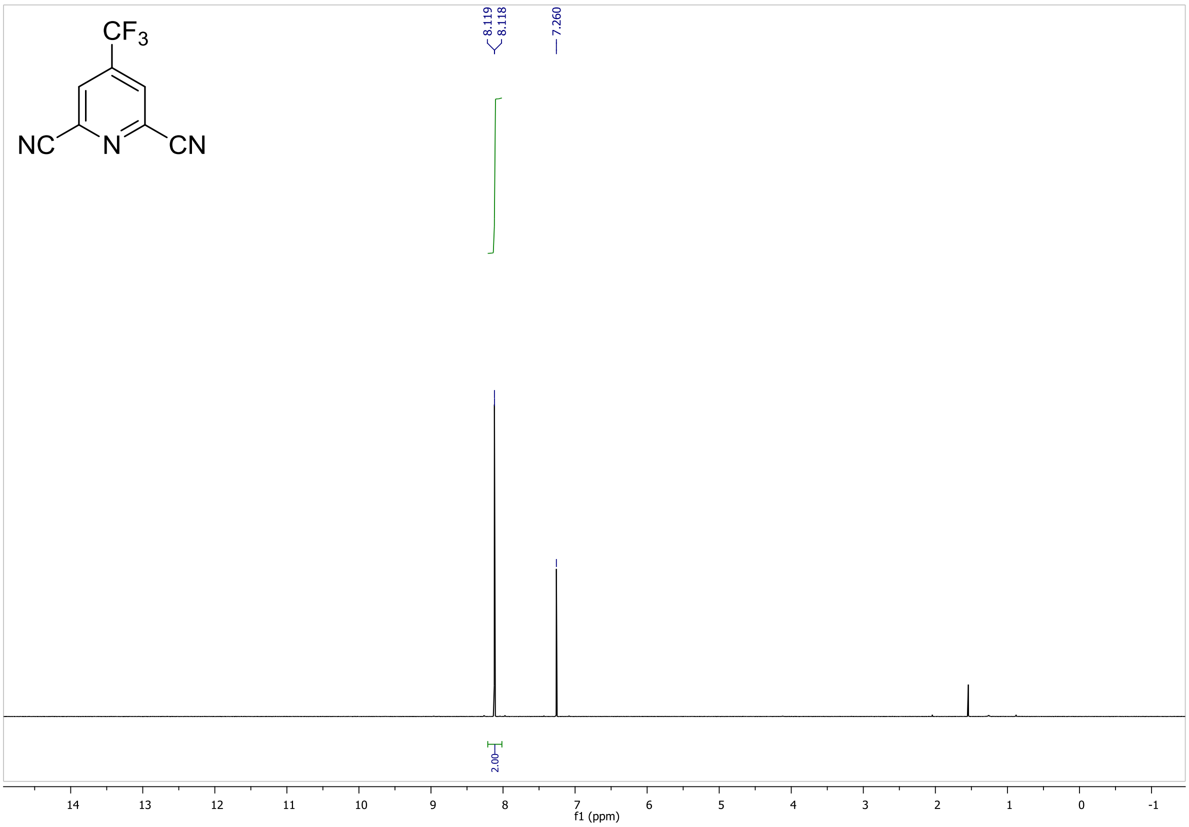

^{13}C NMR, 150 MHz, $CDCl_3$:

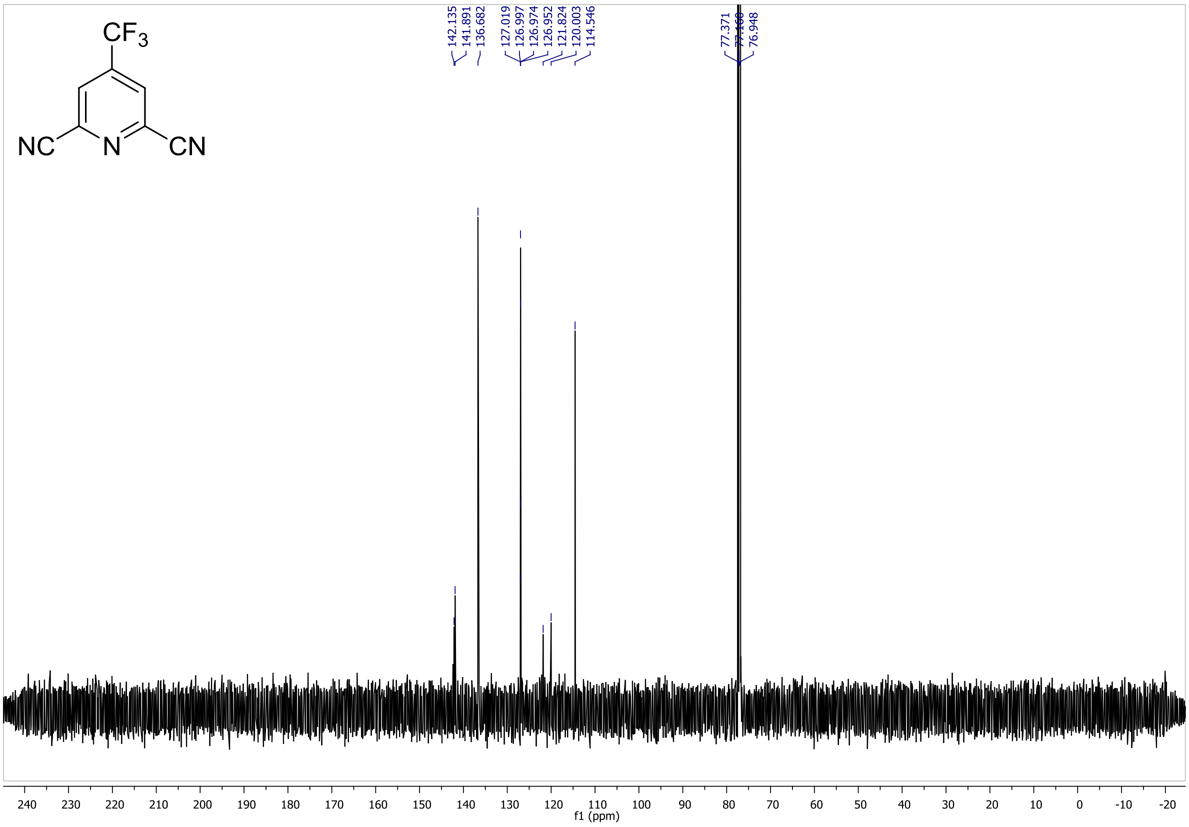

^{19}F NMR, 376 MHz, $CDCl_3$:

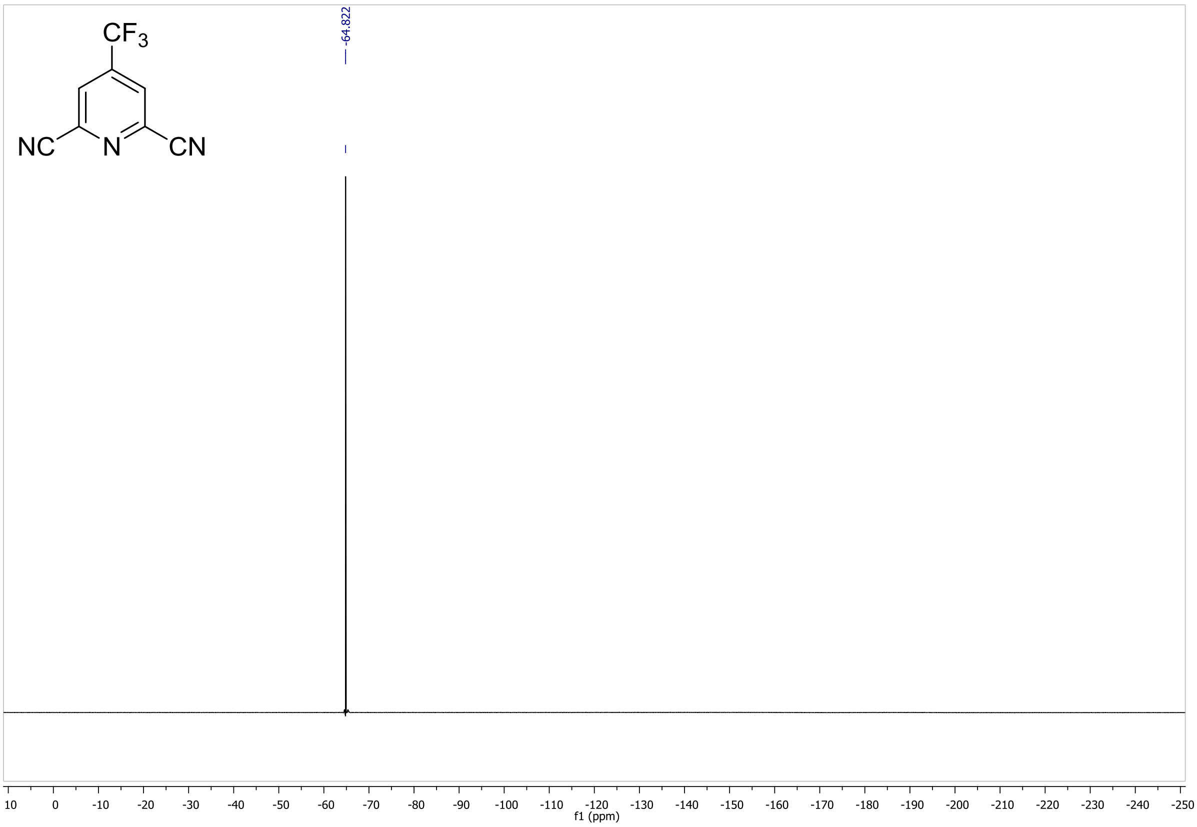

(4*S*,4'*S*)-2,2'-(4-(trifluoromethyl)pyridine-2,6-diyl)bis(4-isopropyl-4,5-dihydrooxazole):

^{1}H NMR, 600 MHz, $CDCl_3$:

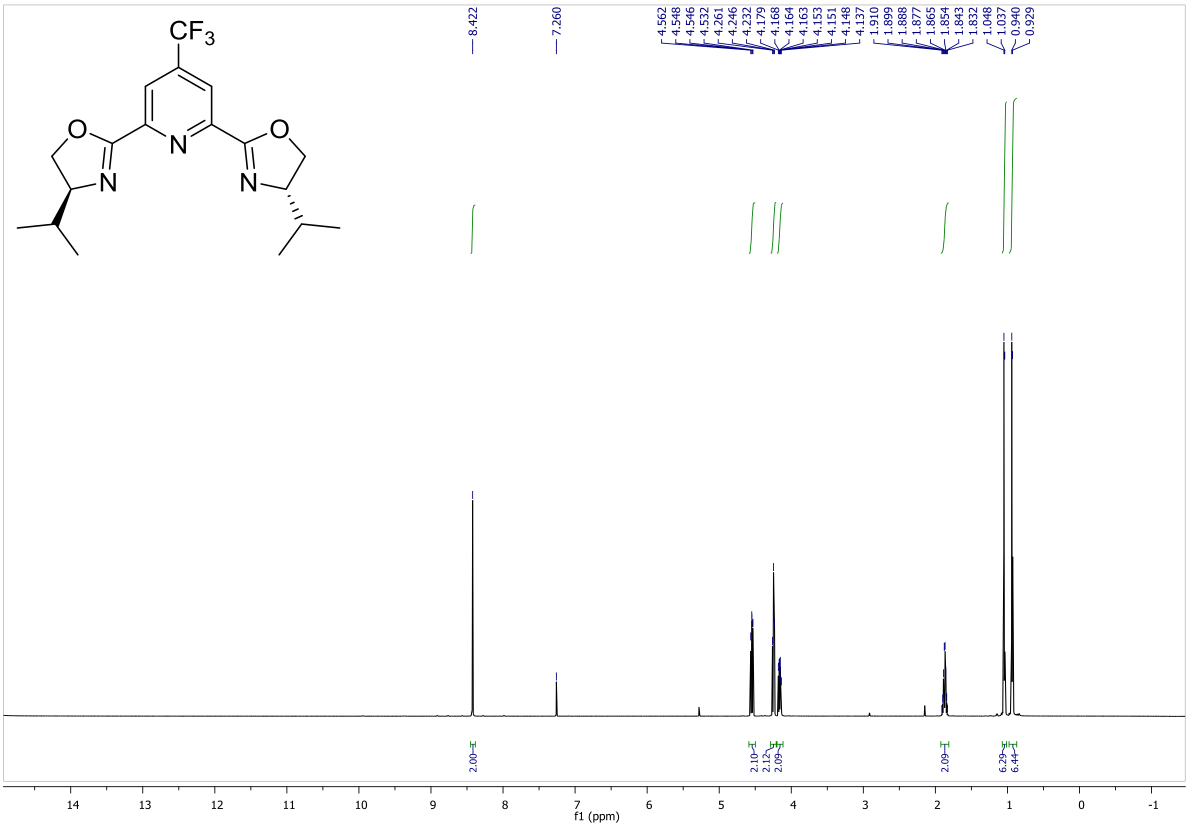

^{13}C NMR, 150 MHz, $CDCl_3$:

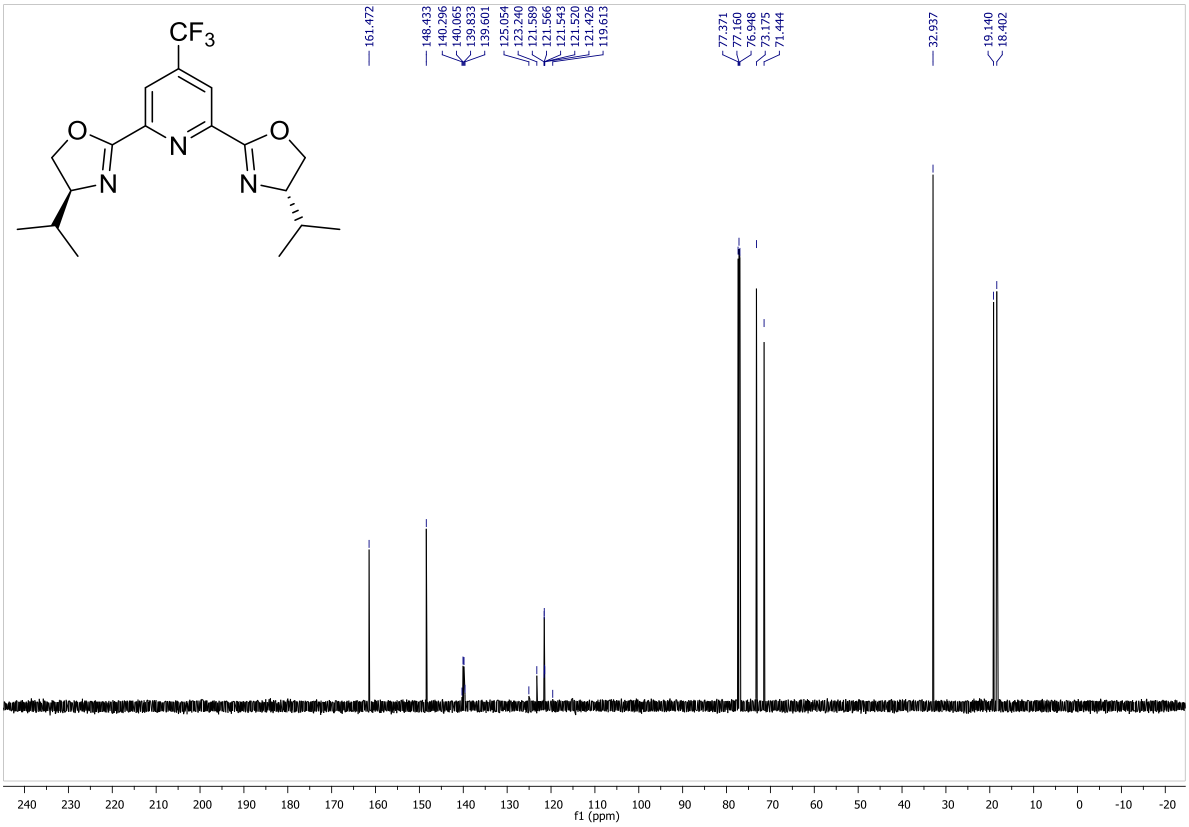

^{19}F NMR, 376 MHz, CDCl$_3$:

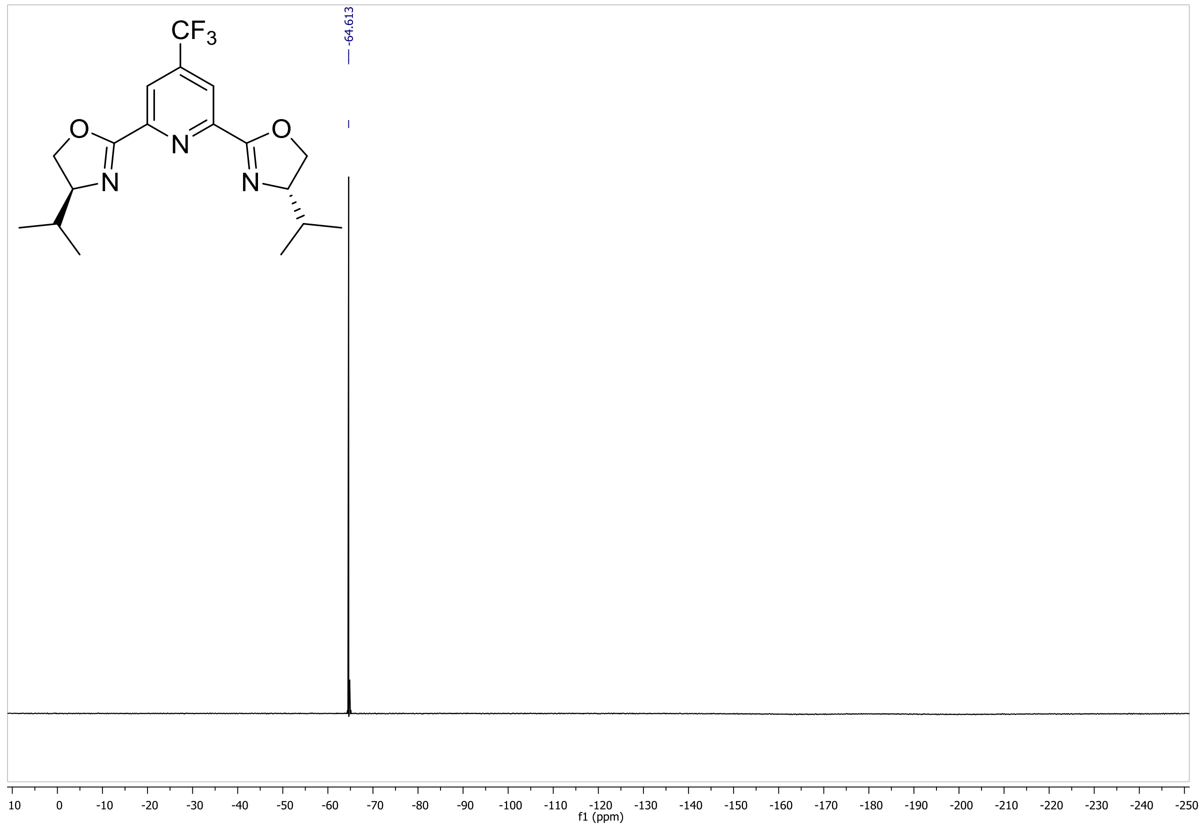

4-methoxypyridine-2,6-dicarbonitrile:

^{1}H NMR, 600 MHz, $CDCl_3$:

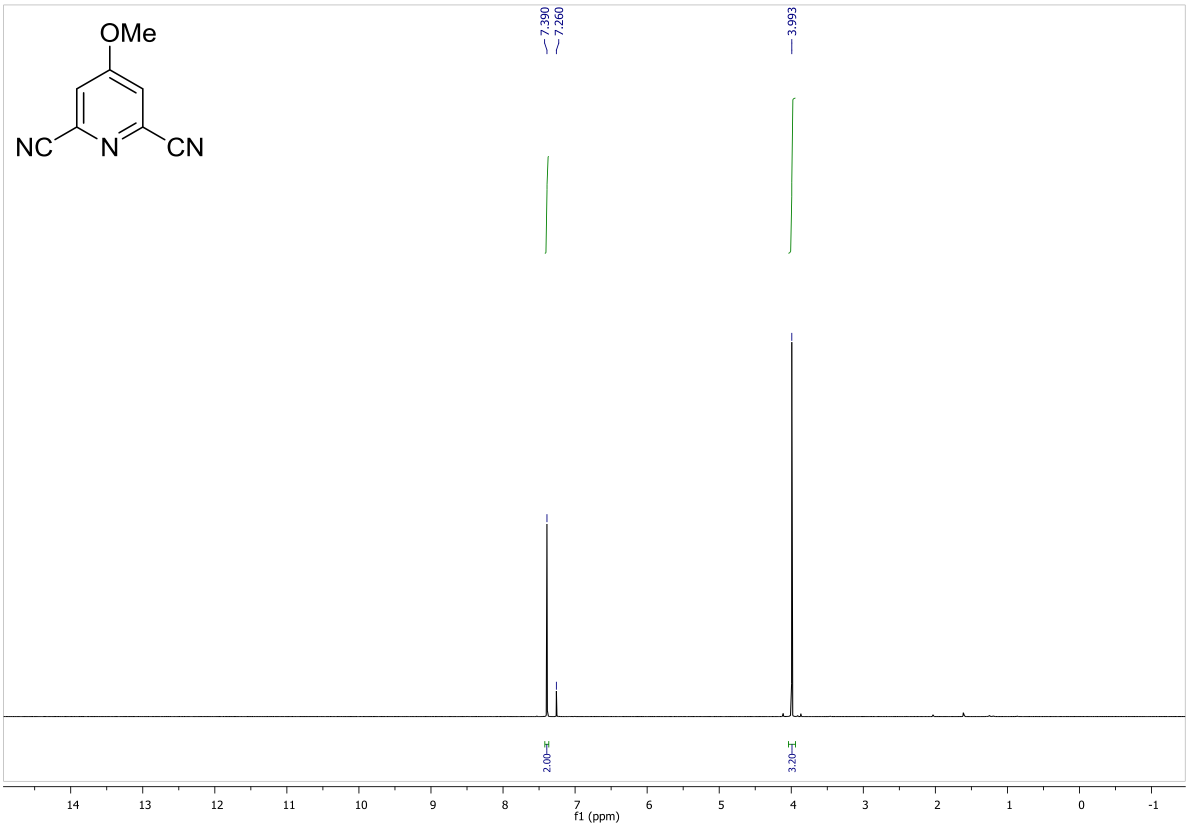

^{13}C NMR, 150 MHz, $CDCl_3$:

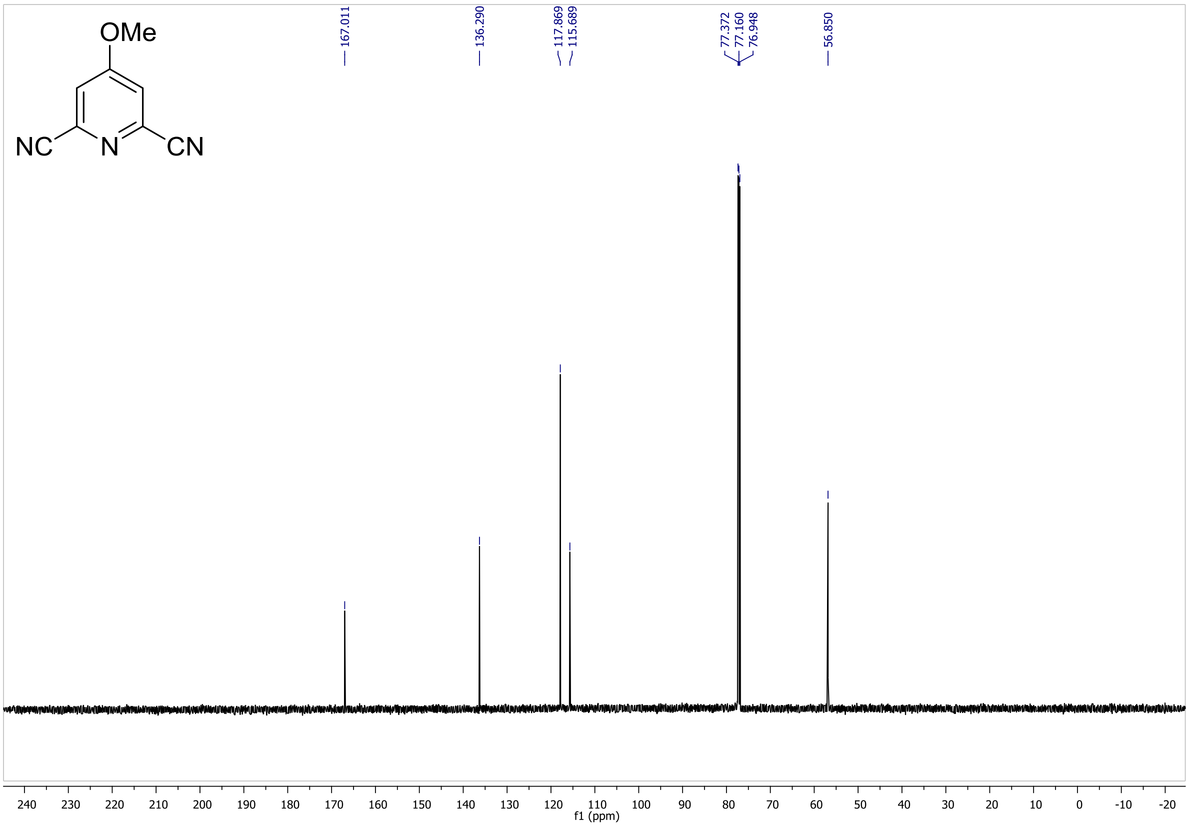

(4*S*,4'*S*)-2,2'-(4-methoxypyridine-2,6-diyl)bis(4-isopropyl-4,5-dihydrooxazole):

^{1}H NMR, 600 MHz, $CDCl_3$:

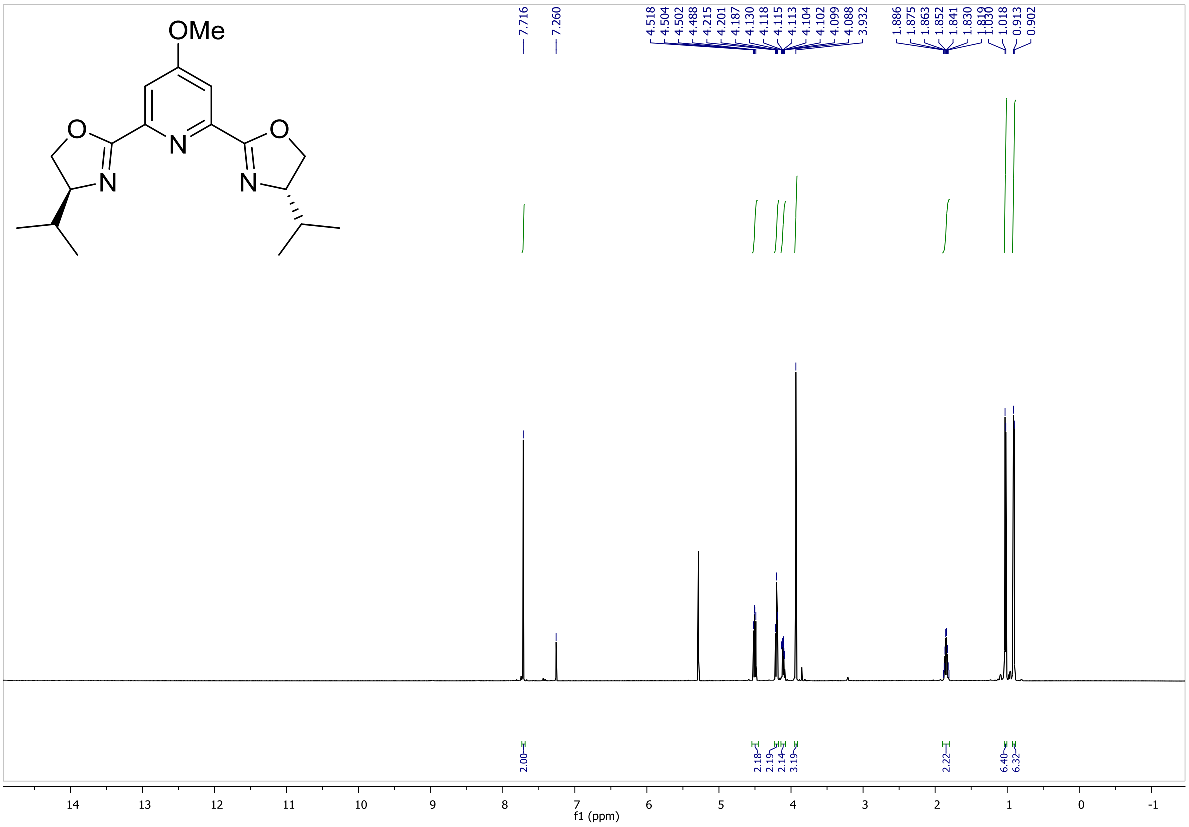

^{13}C NMR, 150 MHz, $CDCl_3$:

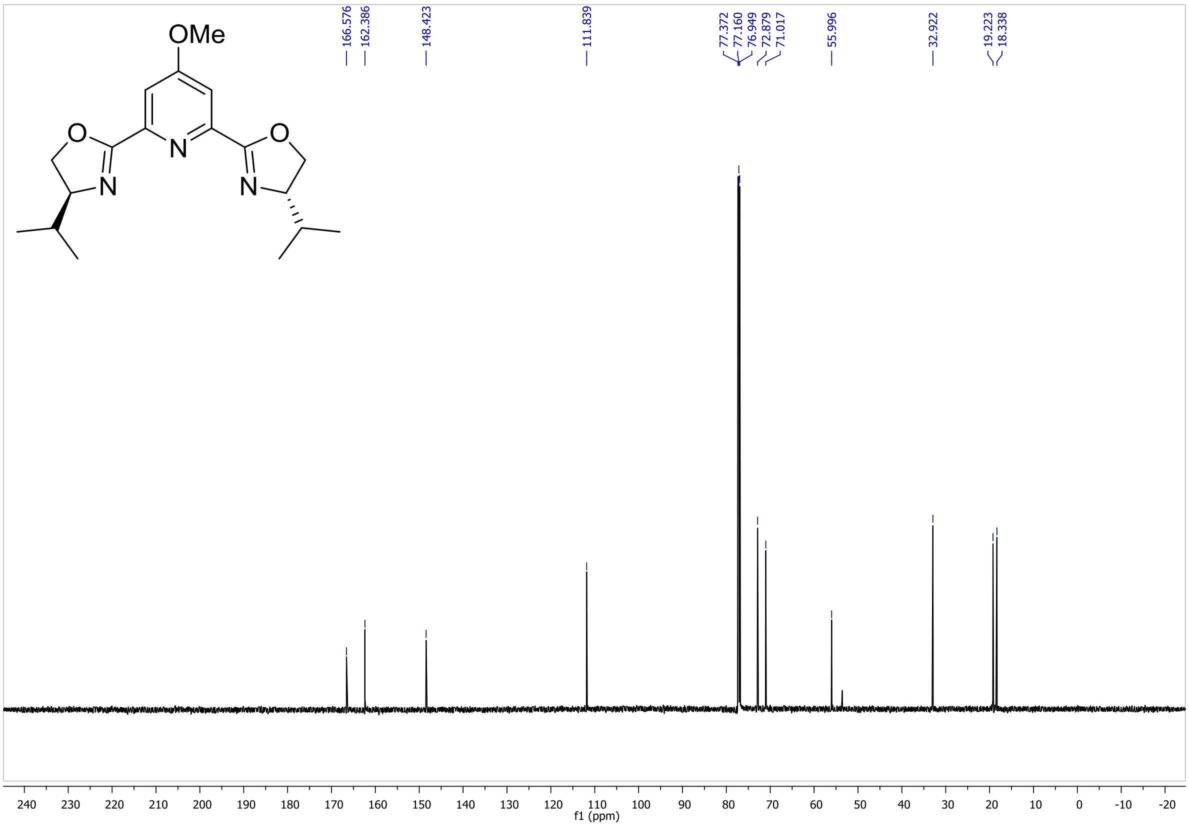

2,6-bis((*S*)-4-cyclopropyl-4,5-dihydrooxazol-2-yl)pyridine:

^{1}H NMR, 600 MHz, CDCl$_3$:

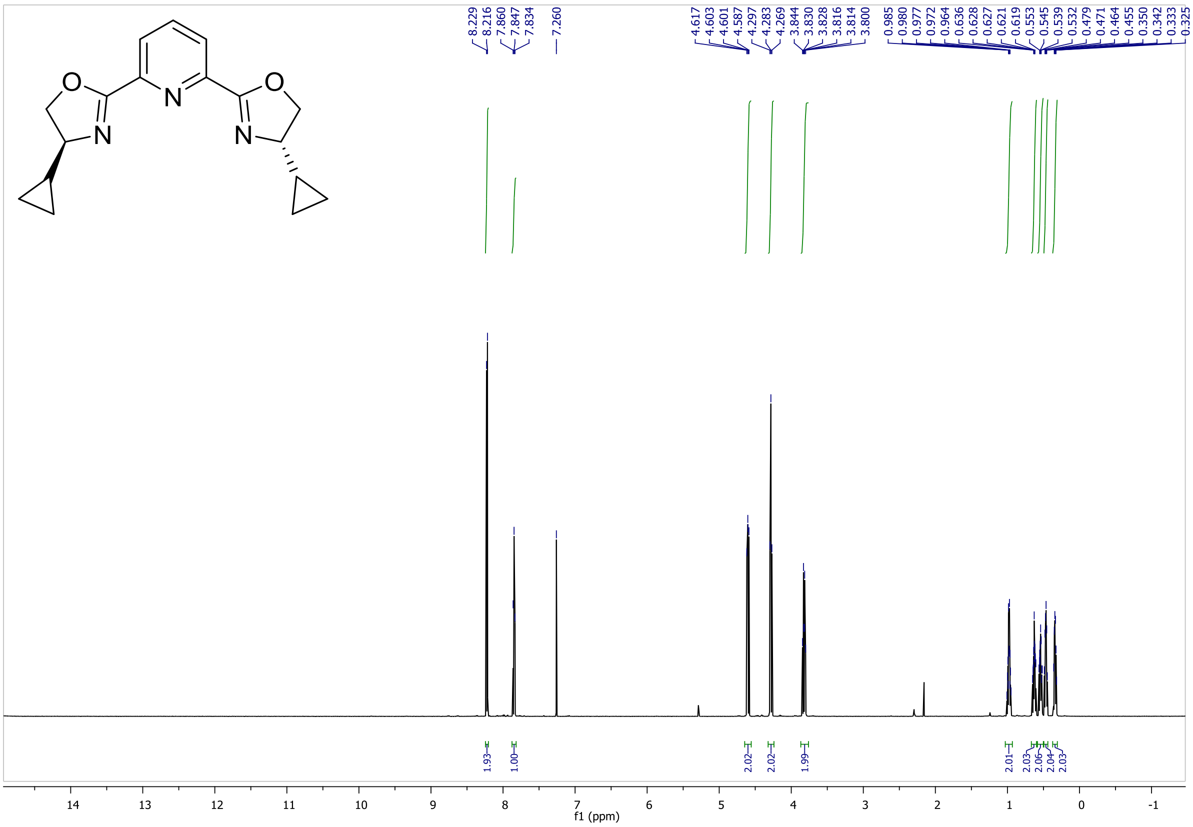

^{13}C NMR, 150 MHz, CDCl$_3$:

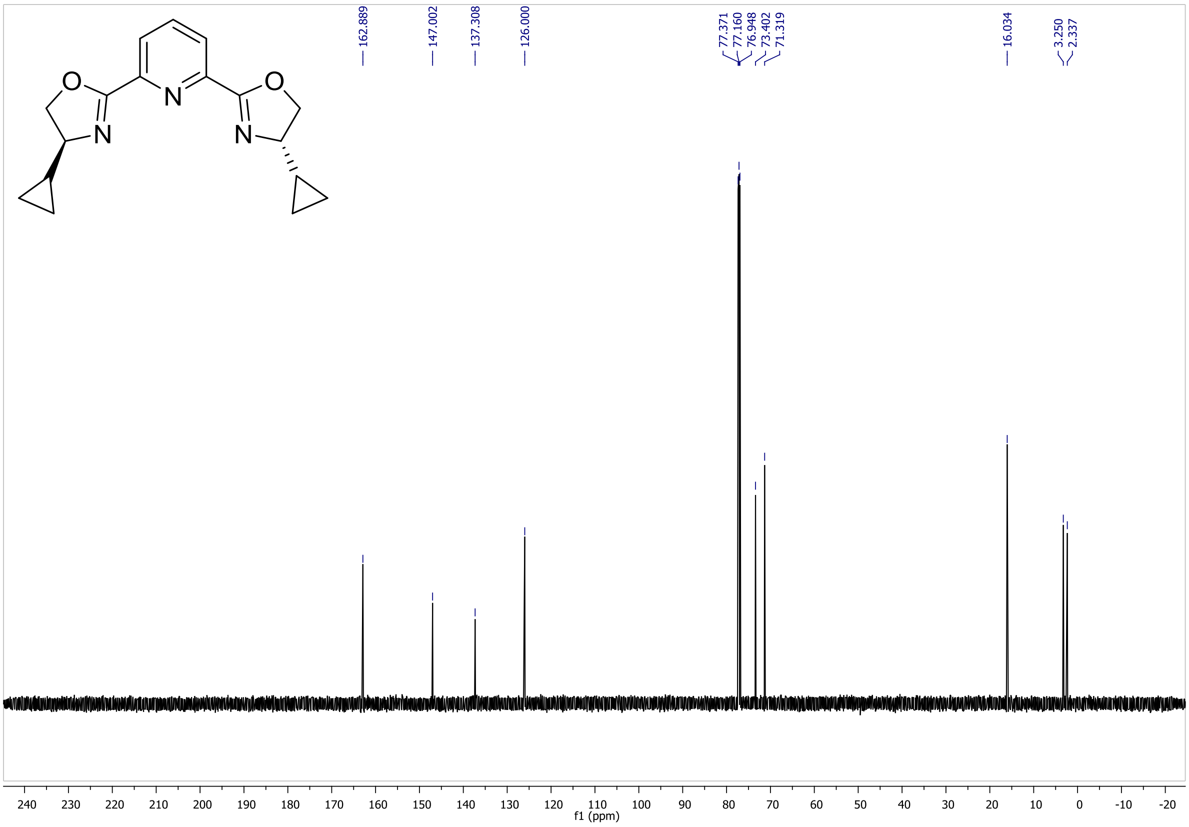

2,6-bis((*S*)-4-cyclohexyl-4,5-dihydrooxazol-2-yl)pyridine:

^{1}H NMR, 600 MHz, $CDCl_3$:

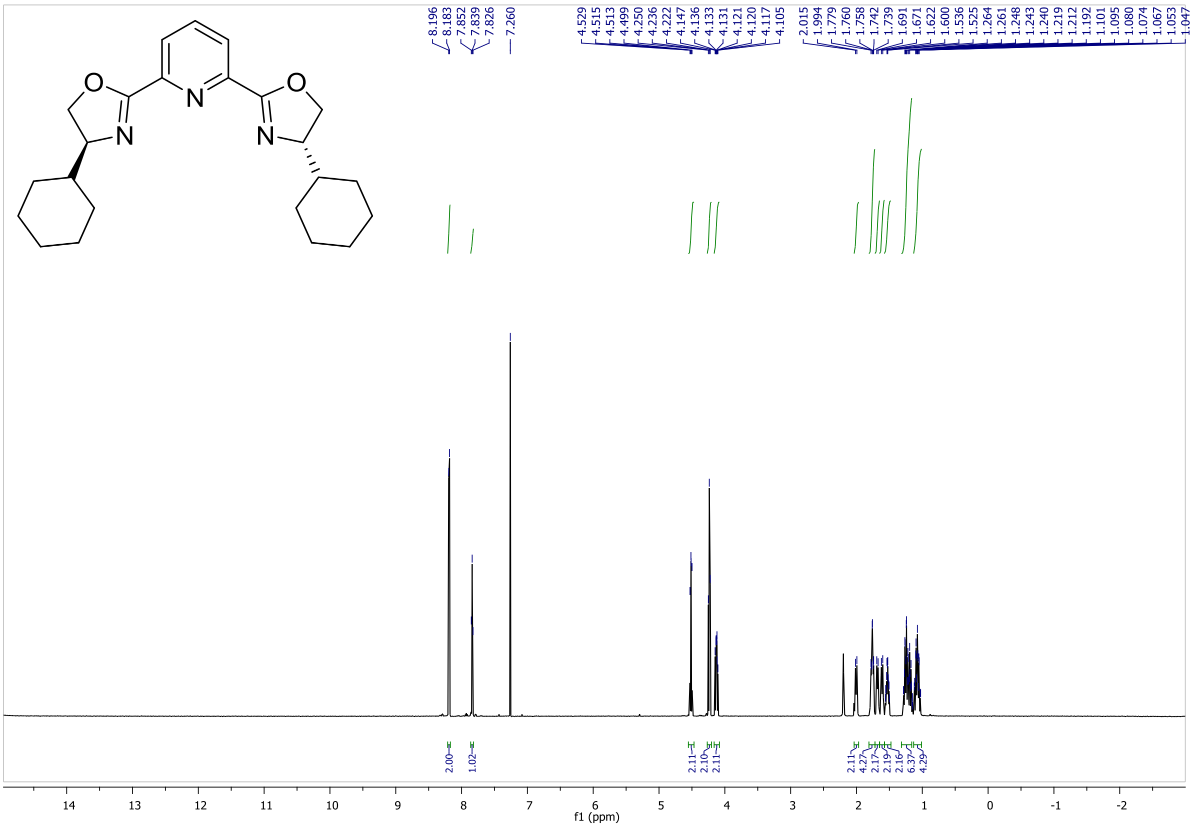

^{13}C NMR, 150 MHz, $CDCl_3$:

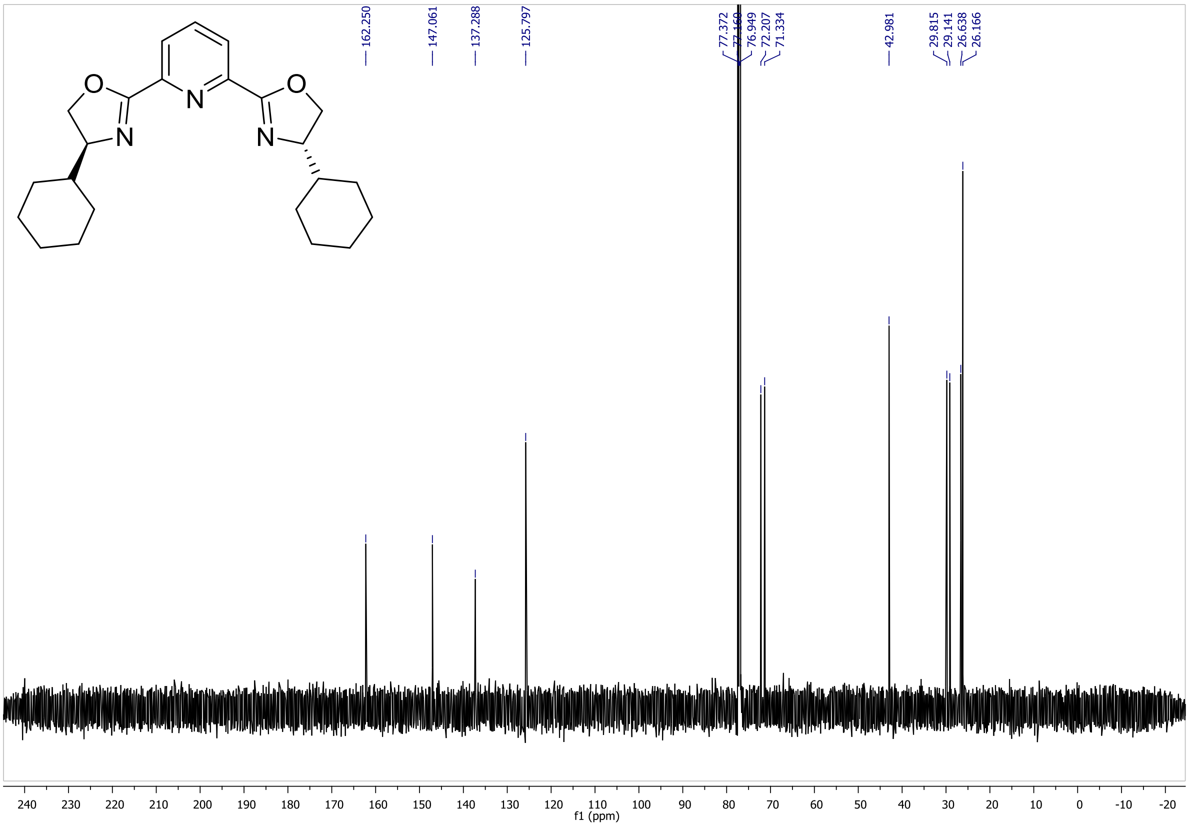

2,6-bis((*S*)-4-benzhydryl-4,5-dihydrooxazol-2-yl)pyridine:

^{1}H NMR, 600 MHz, CDCl$_3$:

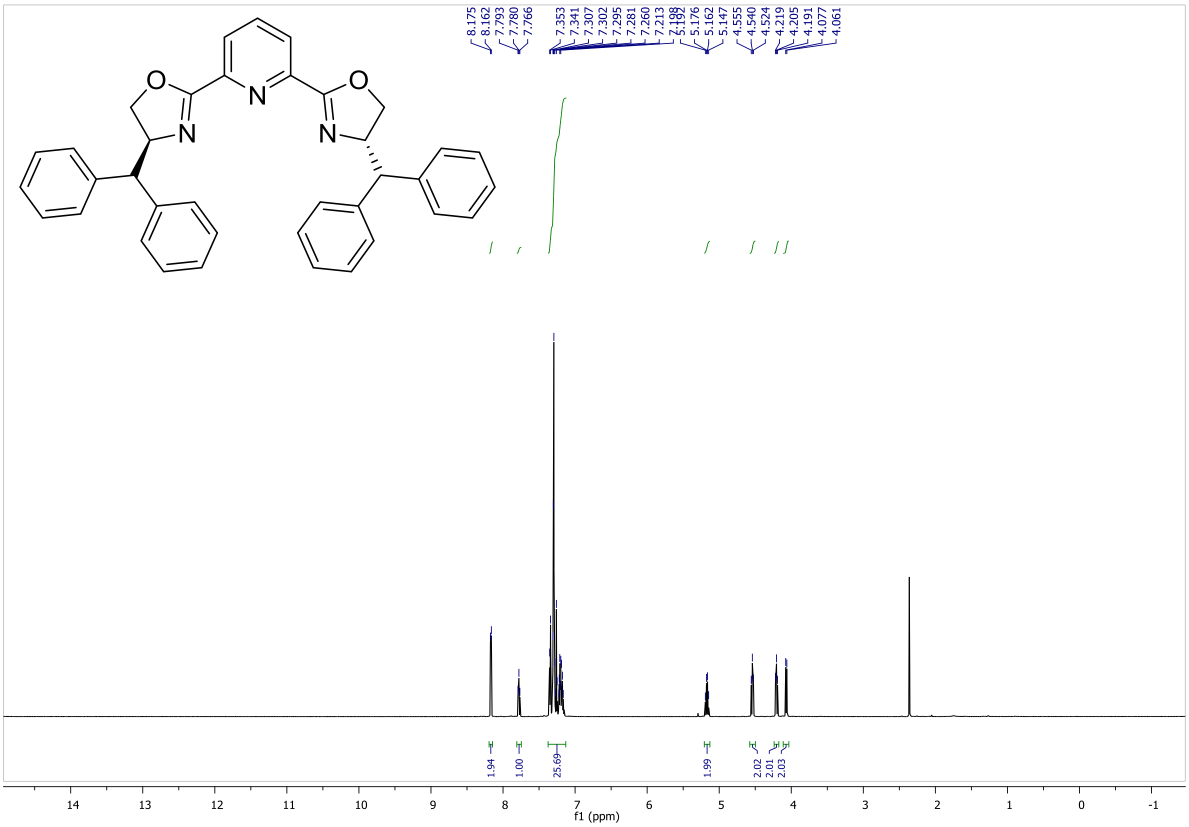

^{13}C NMR, 150 MHz, CDCl$_3$:

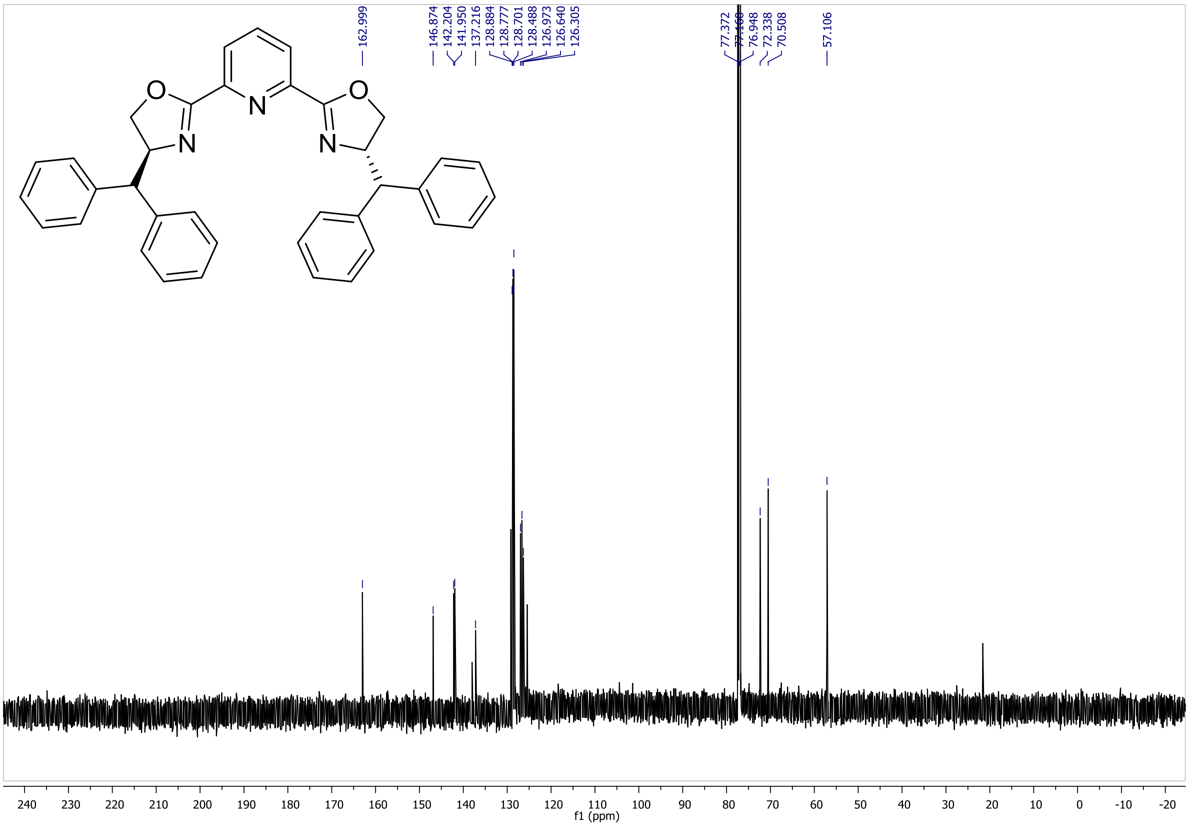

2,6-bis((4*S*,5*R*)-4,5-diphenyl-4,5-dihydrooxazol-2-yl)pyridine:

^{1}H NMR, 600 MHz, $CDCl_3$:

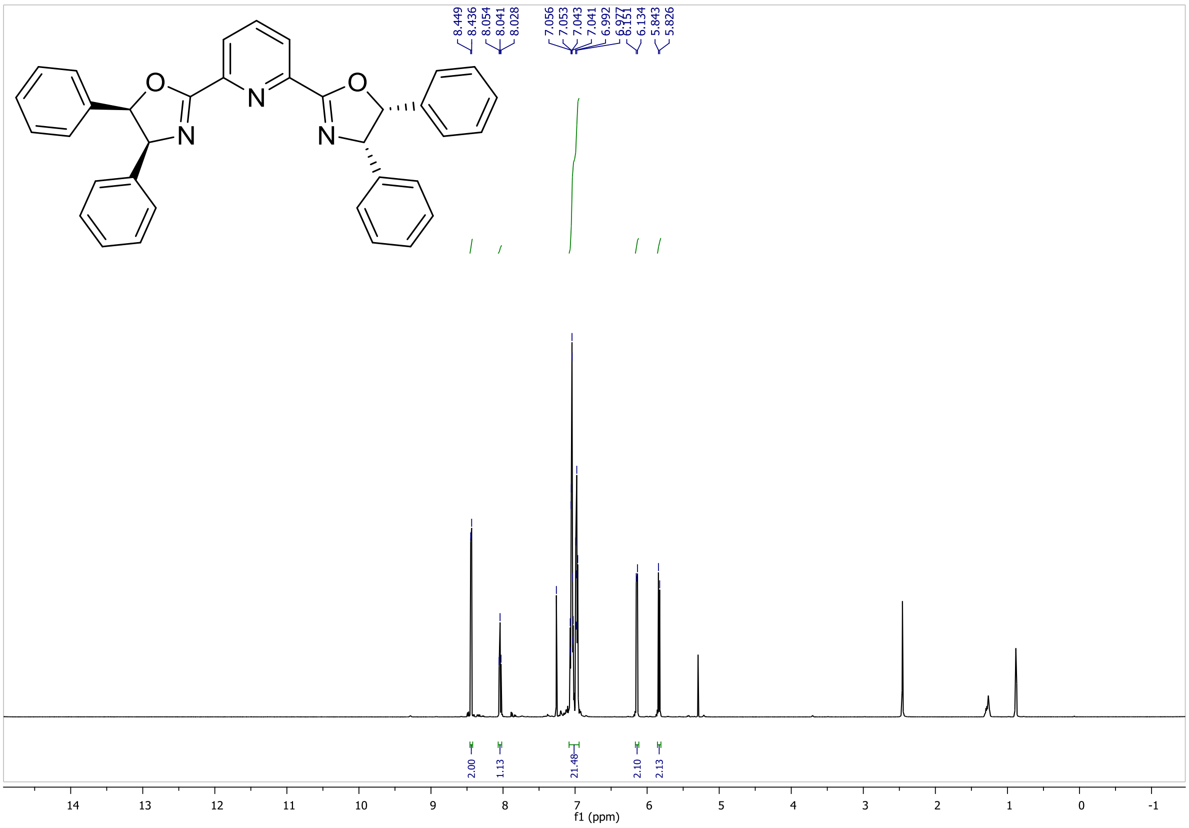

^{13}C NMR, 150 MHz, $CDCl_3$:

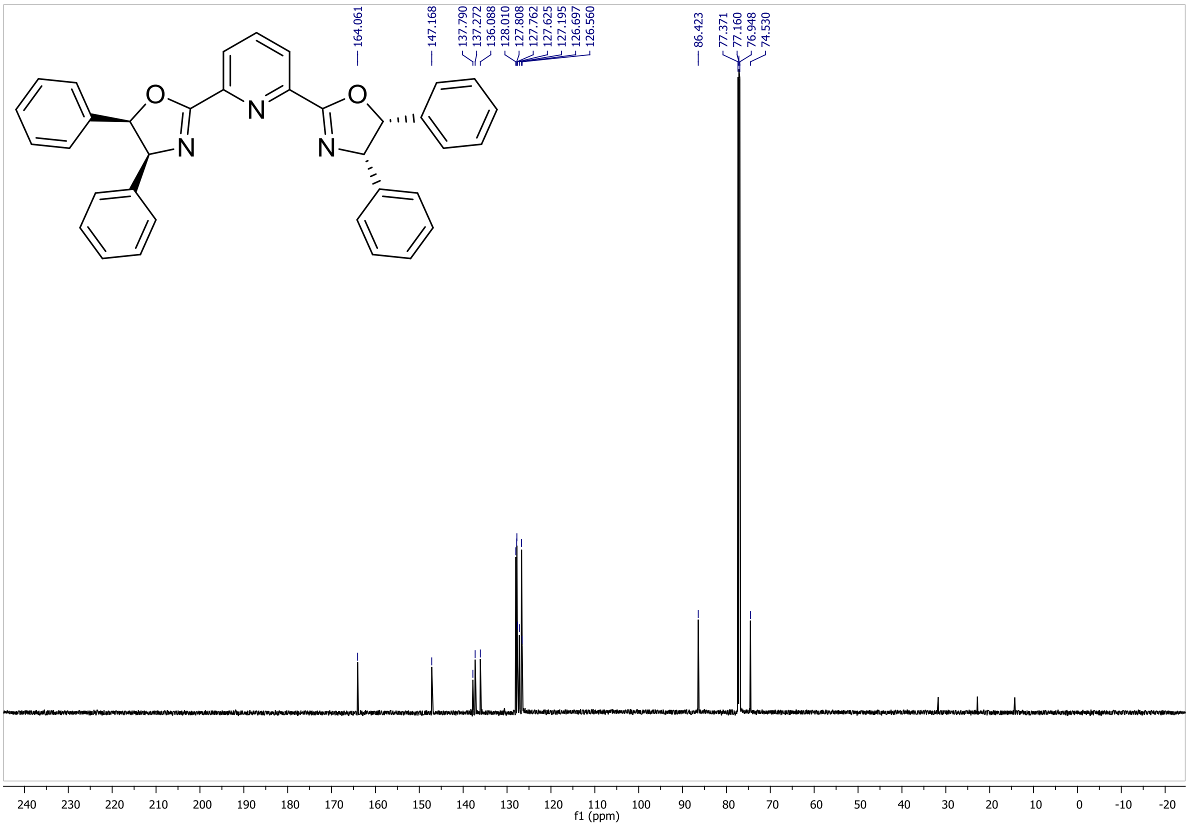

2,6-bis((4*S*,5*S*)-4,5-diphenyl-4,5-dihydrooxazol-2-yl)pyridine:

^{1}H NMR, 600 MHz, $CDCl_3$:

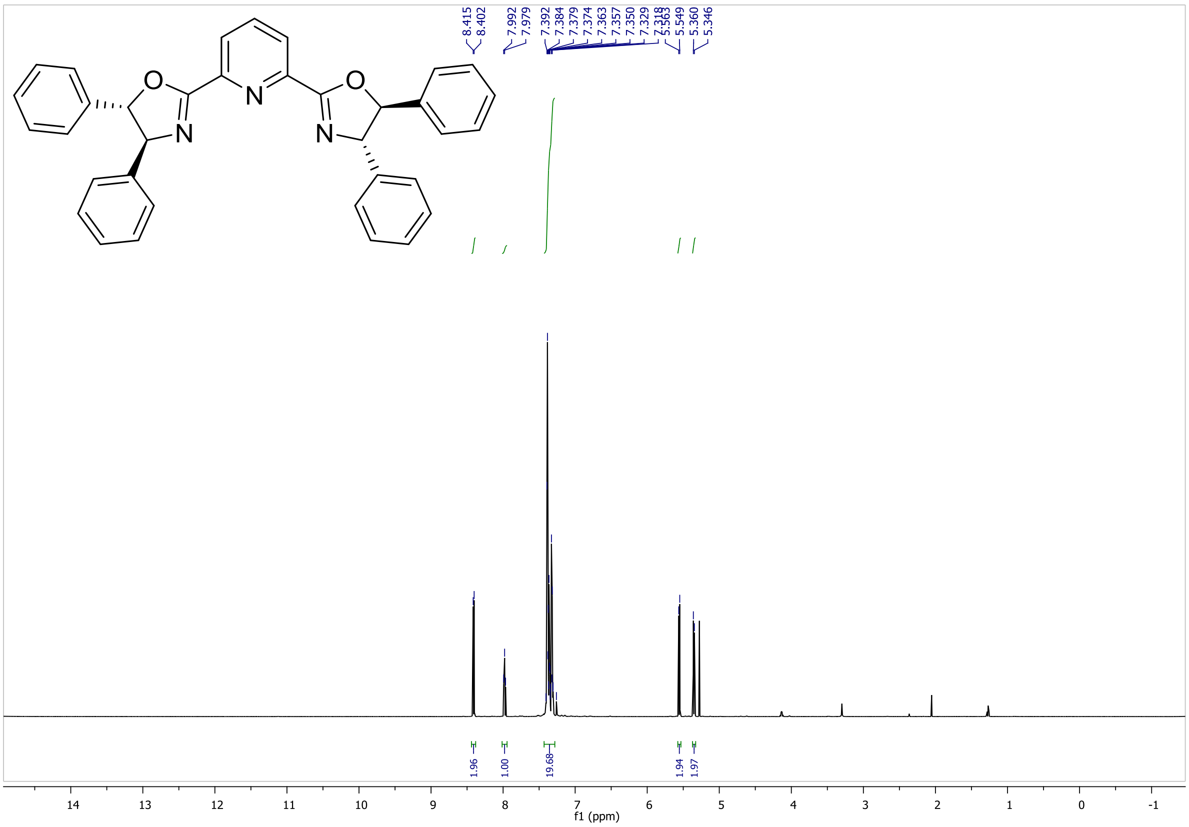

^{13}C NMR, 150 MHz, $CDCl_3$:

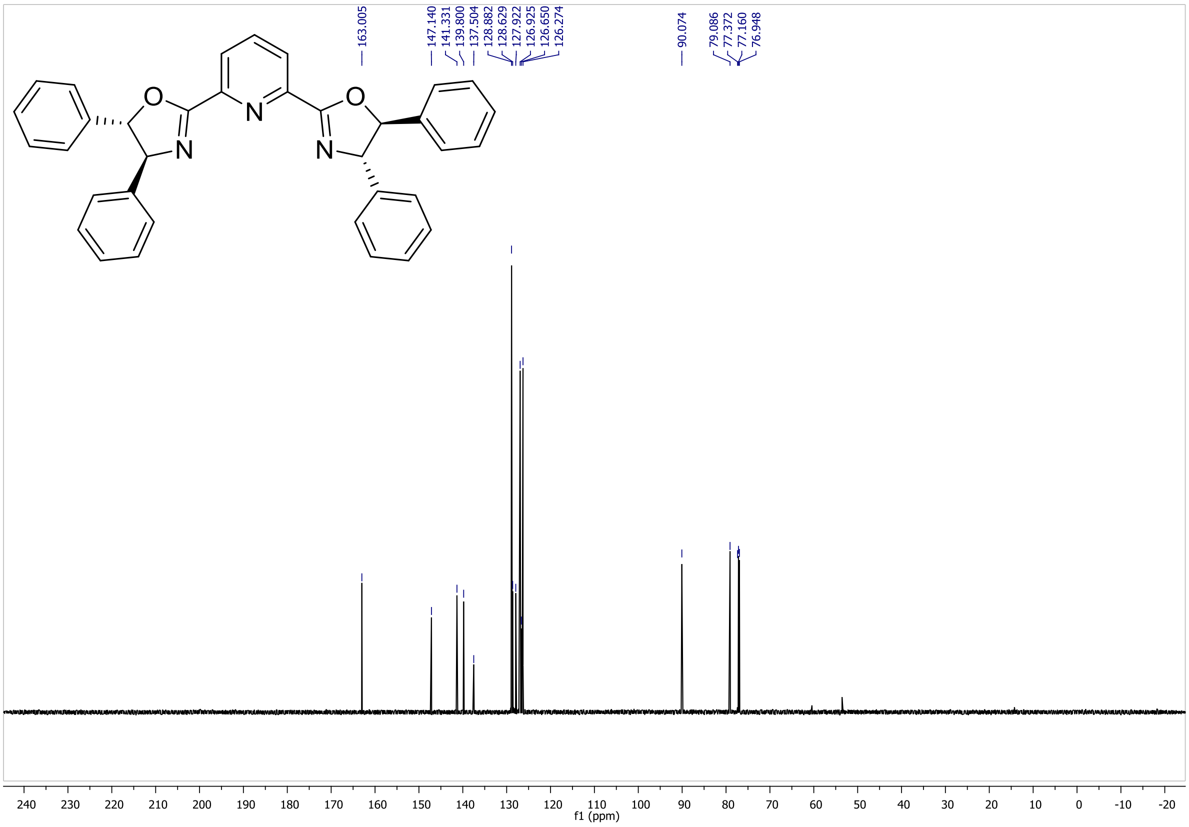

(*S*)-2-(dibenzylamino)-3-methylbutan-1-ol:

^{1}H NMR, 600 MHz, $CDCl_3$:

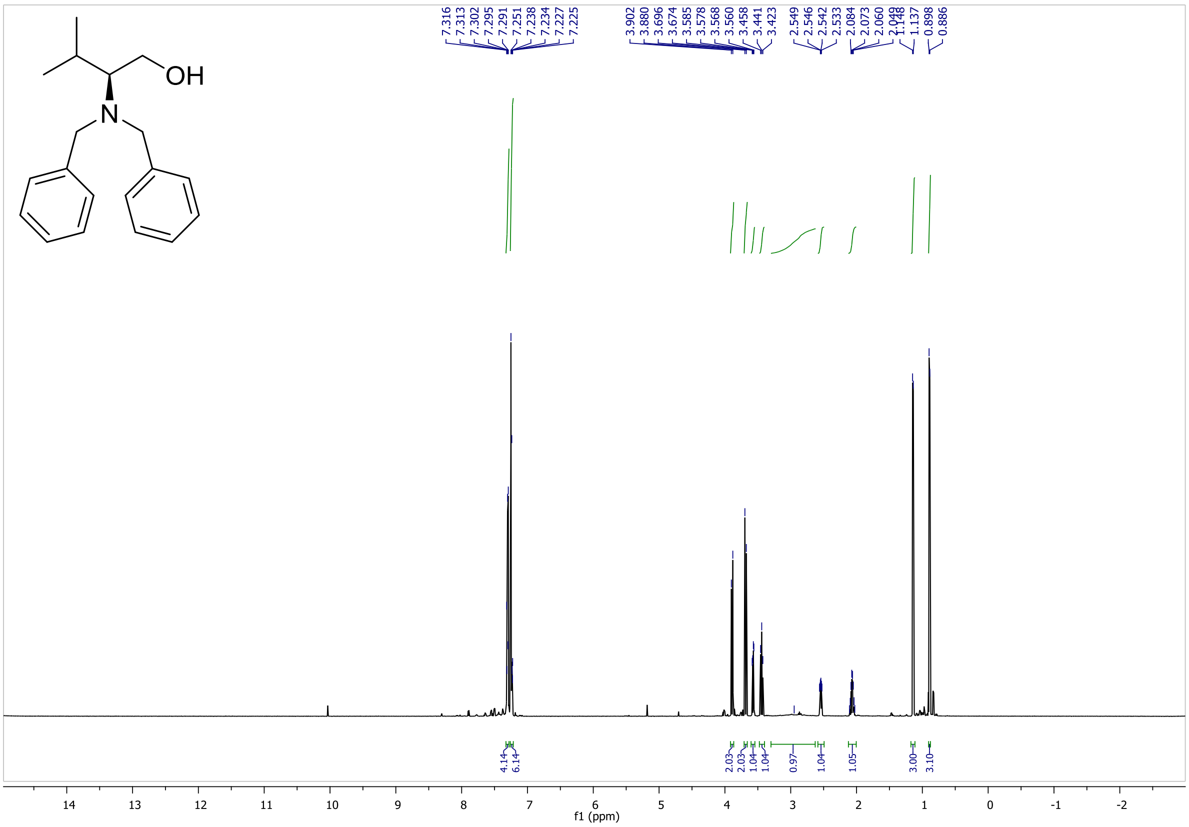

^{13}C NMR, 150 MHz, $CDCl_3$:

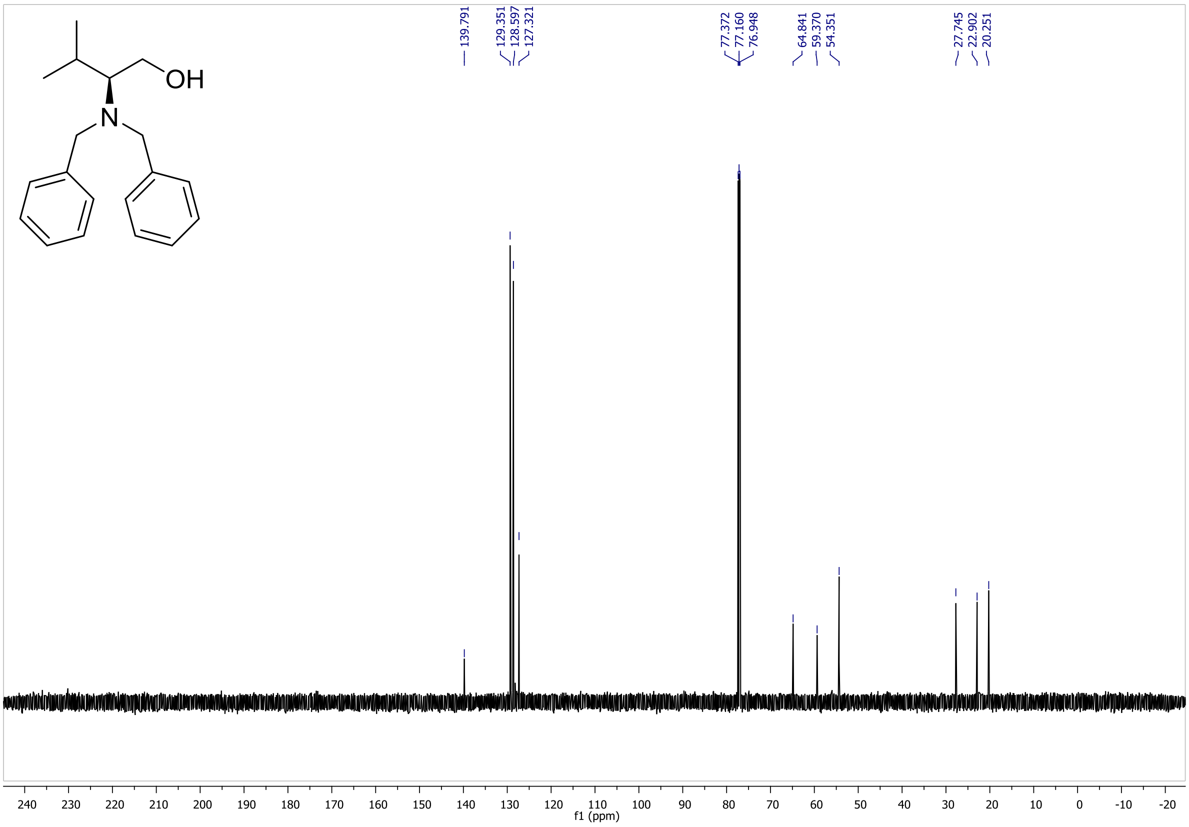

(*S*)-2-(dibenzylamino)-3-methylbutanal:

^{1}H NMR, 600 MHz, $CDCl_3$:

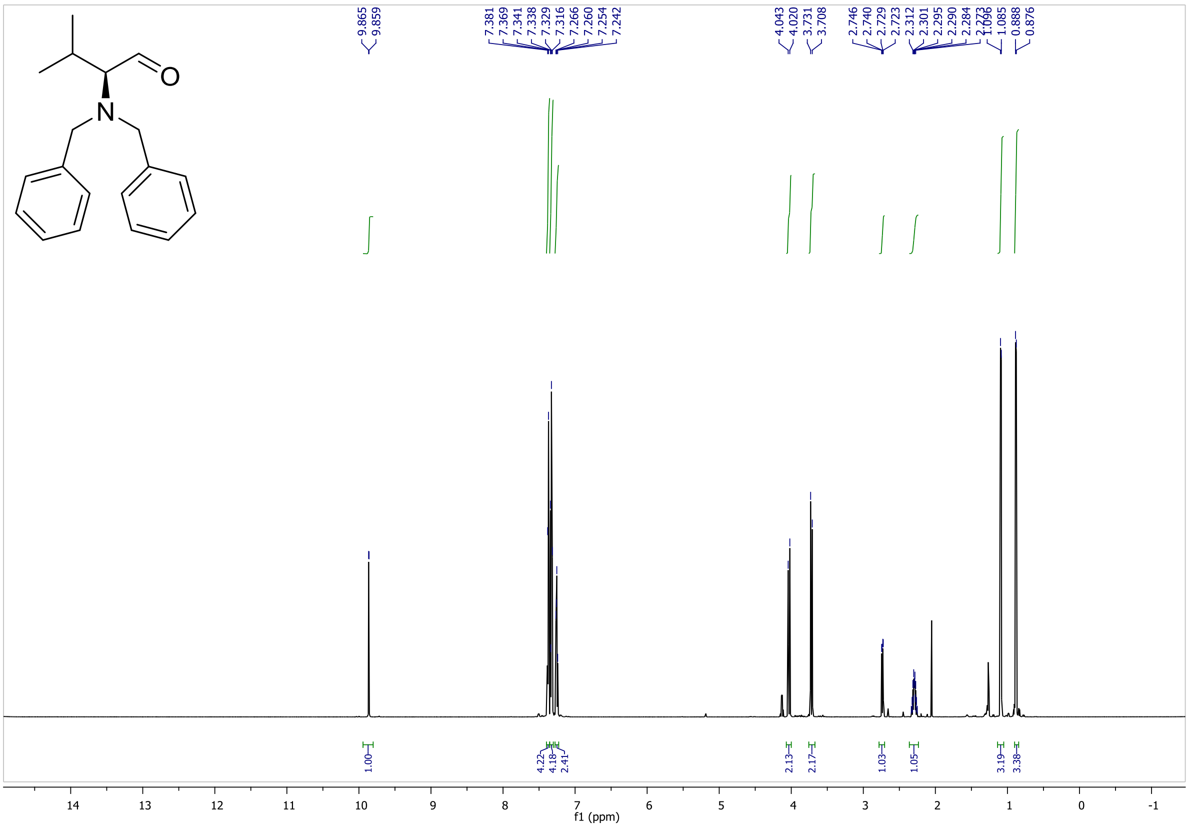

^{13}C NMR, 150 MHz, $CDCl_3$:

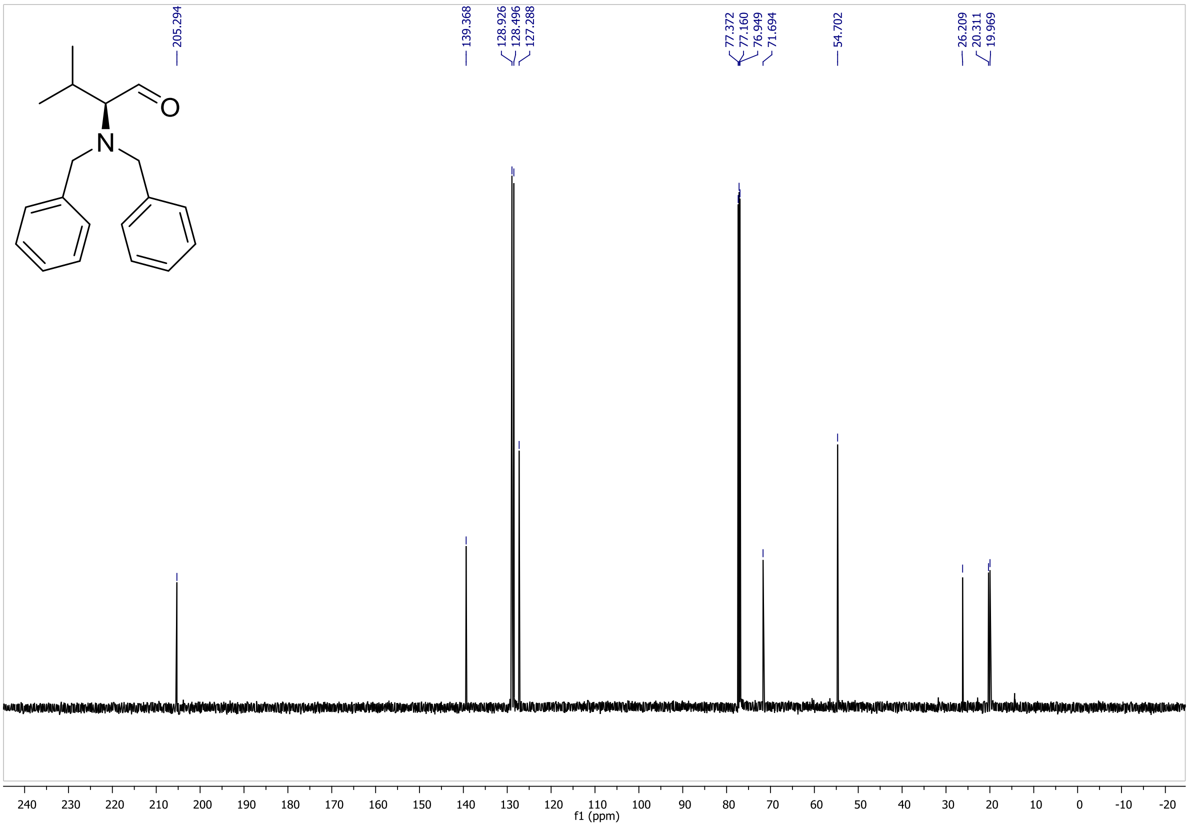

(3*S*,4*S*)-4-(dibenzylamino)-2,5-dimethylhexan-3-ol:

^{1}H NMR, 600 MHz, $CDCl_3$:

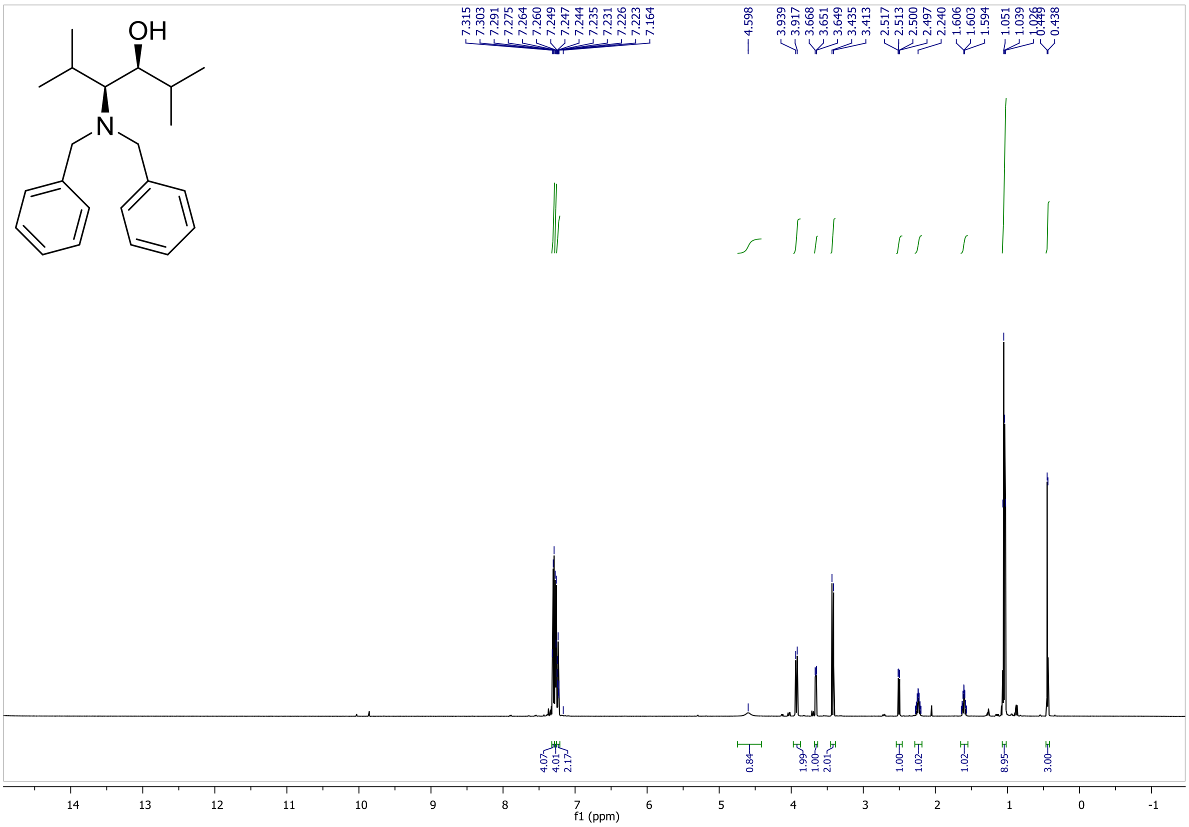

^{13}C NMR, 150 MHz, $CDCl_3$:

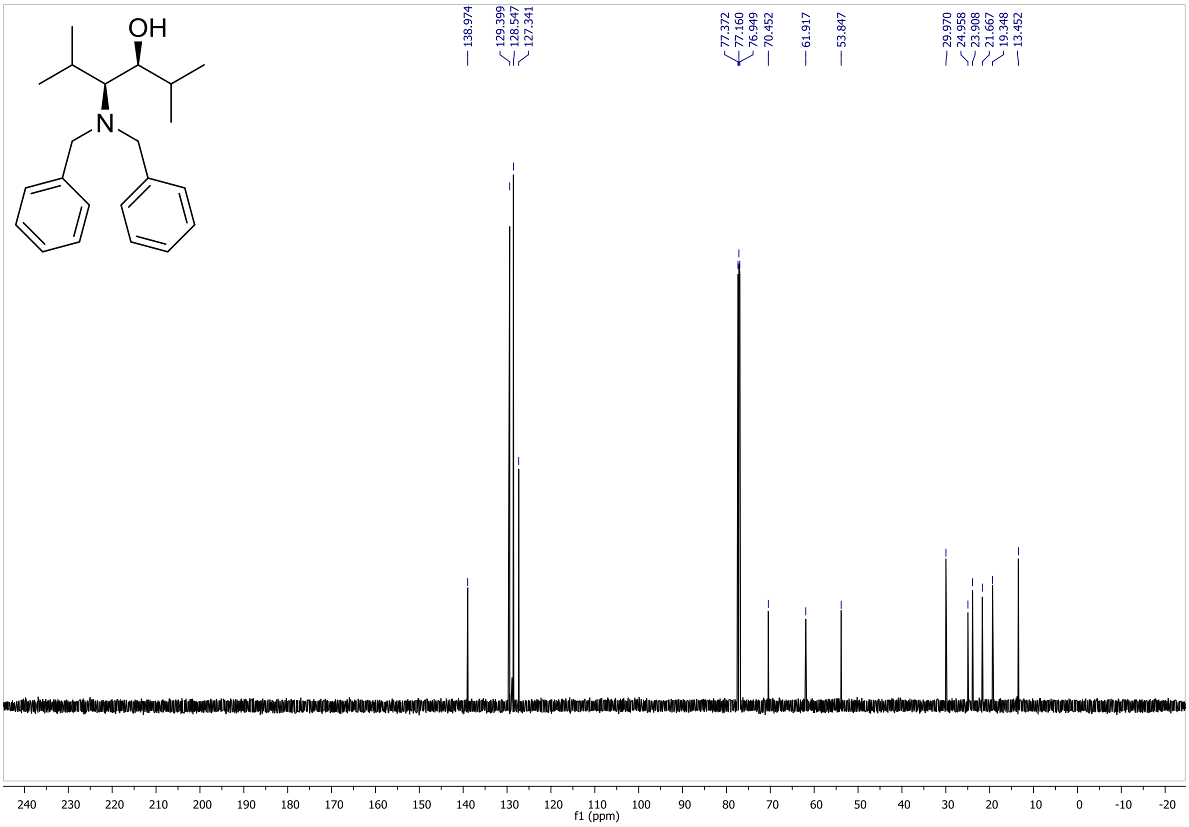

(3*S*,4*S*)-4-amino-2,5-dimethylhexan-3-ol:

^{1}H NMR, 600 MHz, $CDCl_3$:

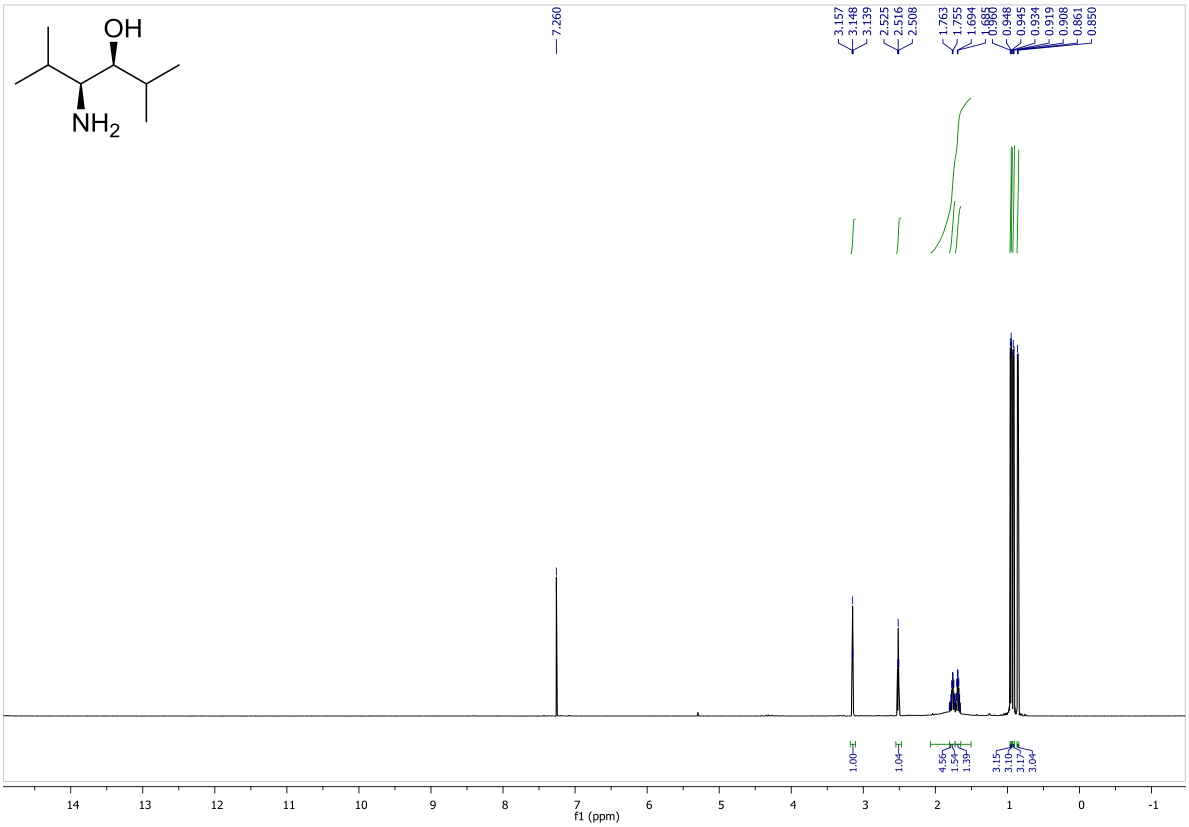

^{13}C NMR, 150 MHz, $CDCl_3$:

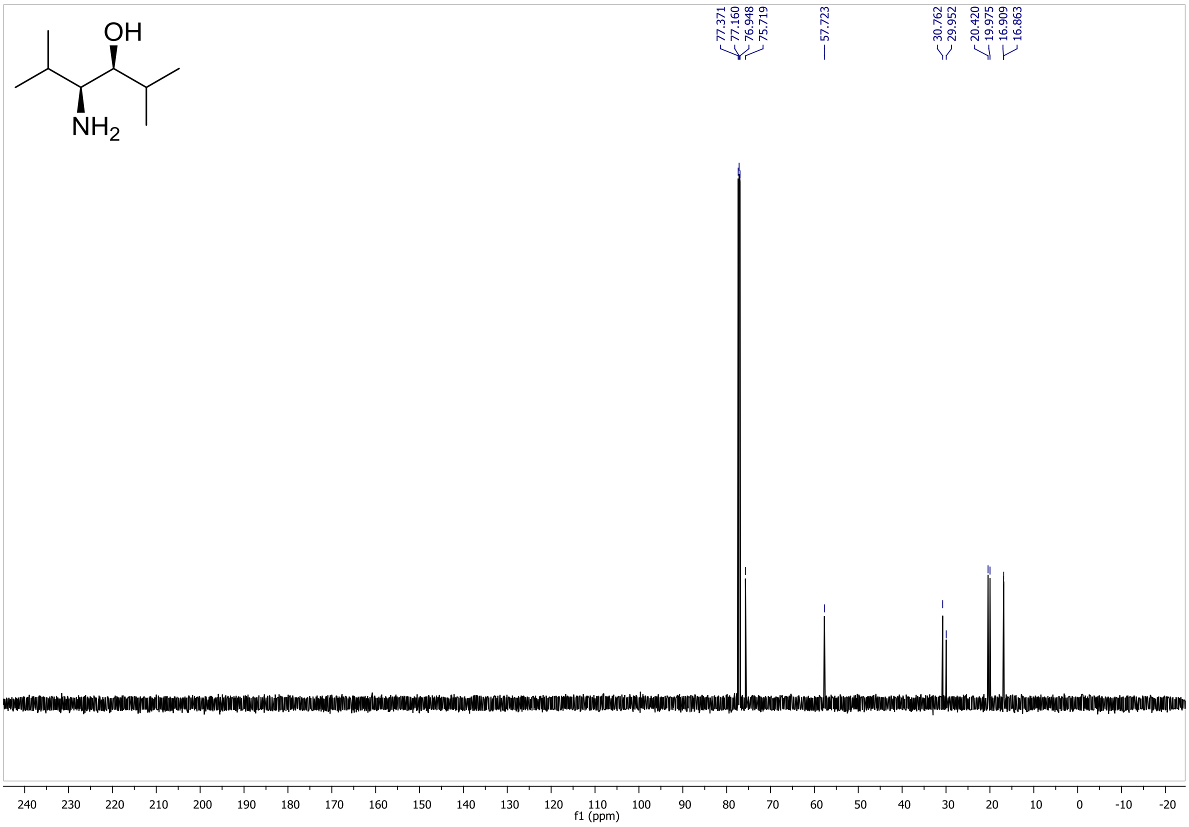

2,6-bis((4*S*,5*S*)-4,5-diisopropyl-4,5-dihydrooxazol-2-yl)pyridine (L3):

^{1}H NMR, 600 MHz, CDCl$_3$:

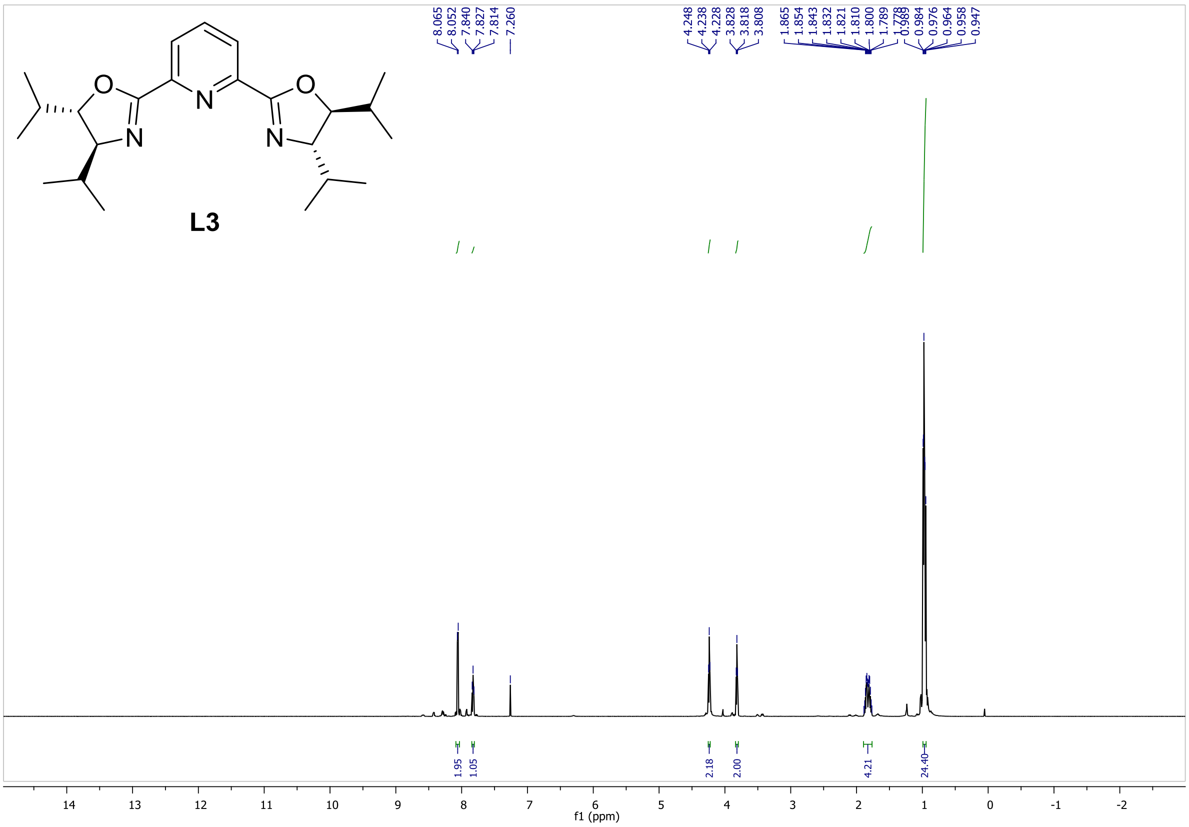

^{13}C NMR, 150 MHz, CDCl$_3$:

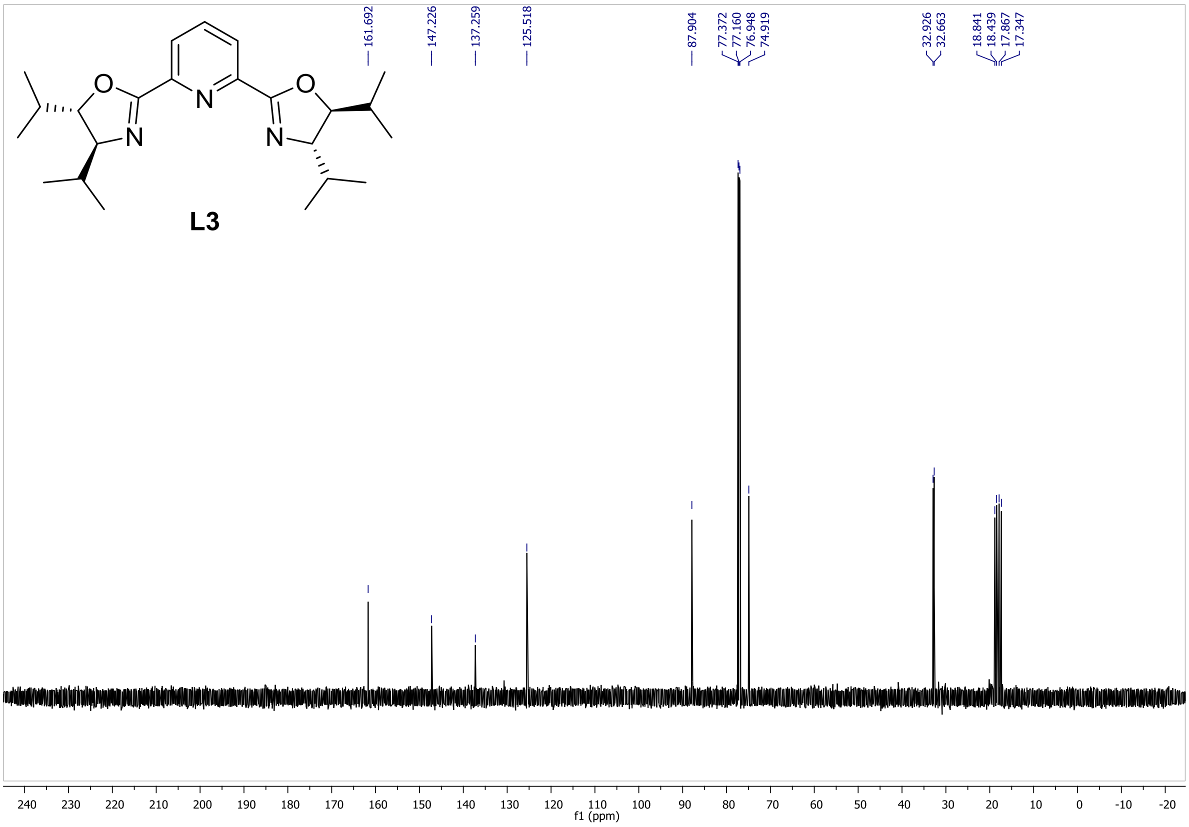

8.2. NMR spectra for PyBIM ligands

N^2,N^6-bis((*S*)-1-hydroxy-3-methylbutan-2-yl)pyridine-2,6-dicarboxamide:

^{1}H NMR, 600 MHz, $CDCl_3$:

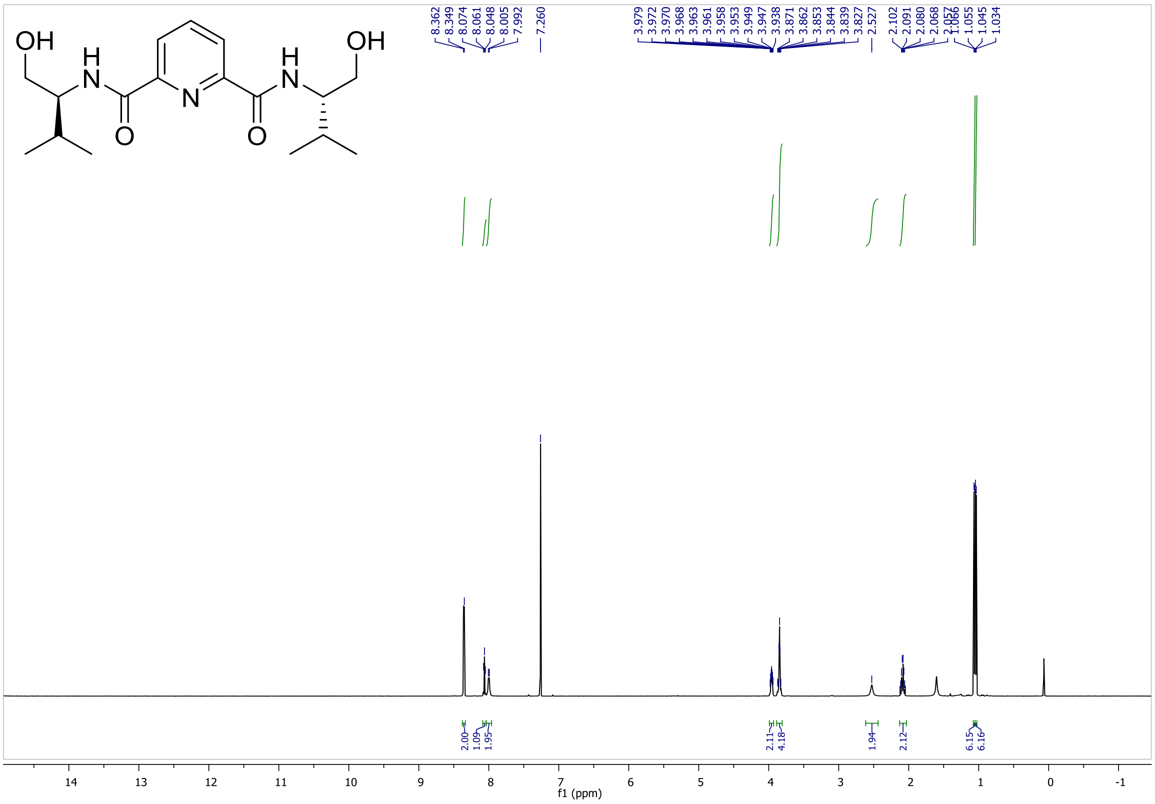

^{13}C NMR, 150 MHz, $CDCl_3$:

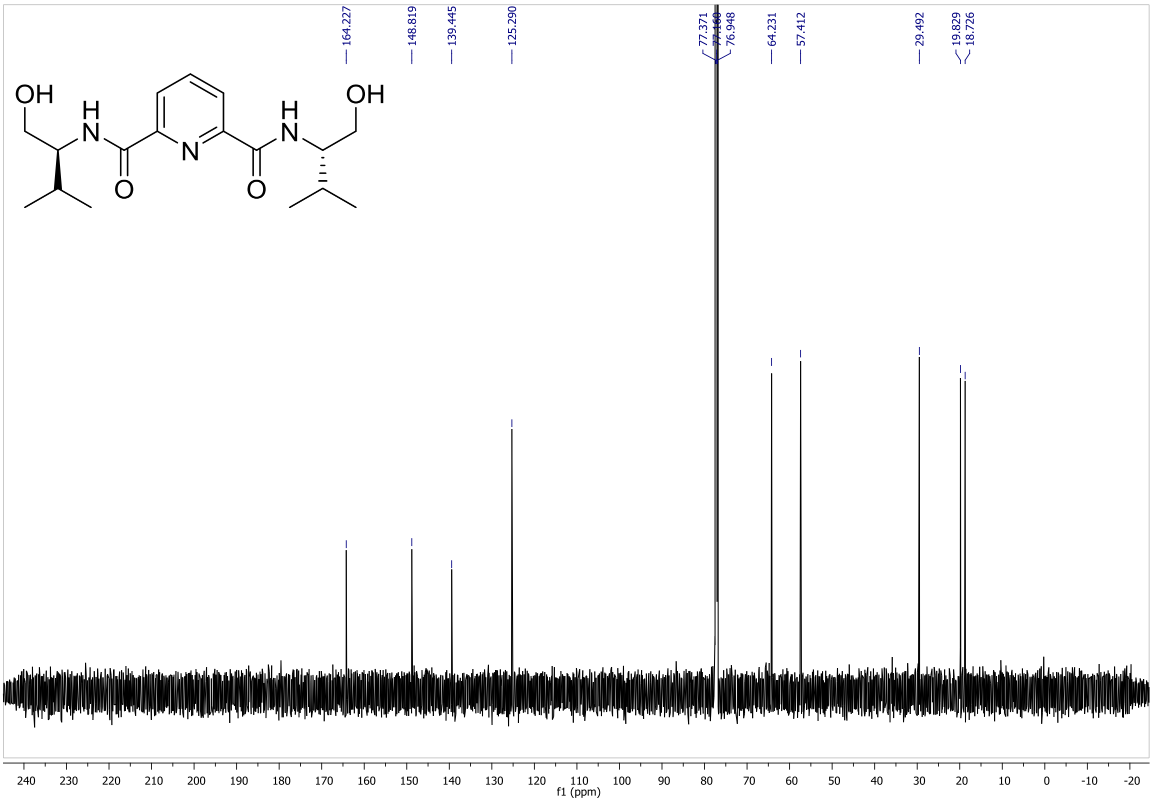

2,6-bis((*S*)-1-benzyl-4-isopropyl-4,5-dihydro-1*H*-imidazol-2-yl)pyridine:

^{1}H NMR, 600 MHz, CDCl$_3$:

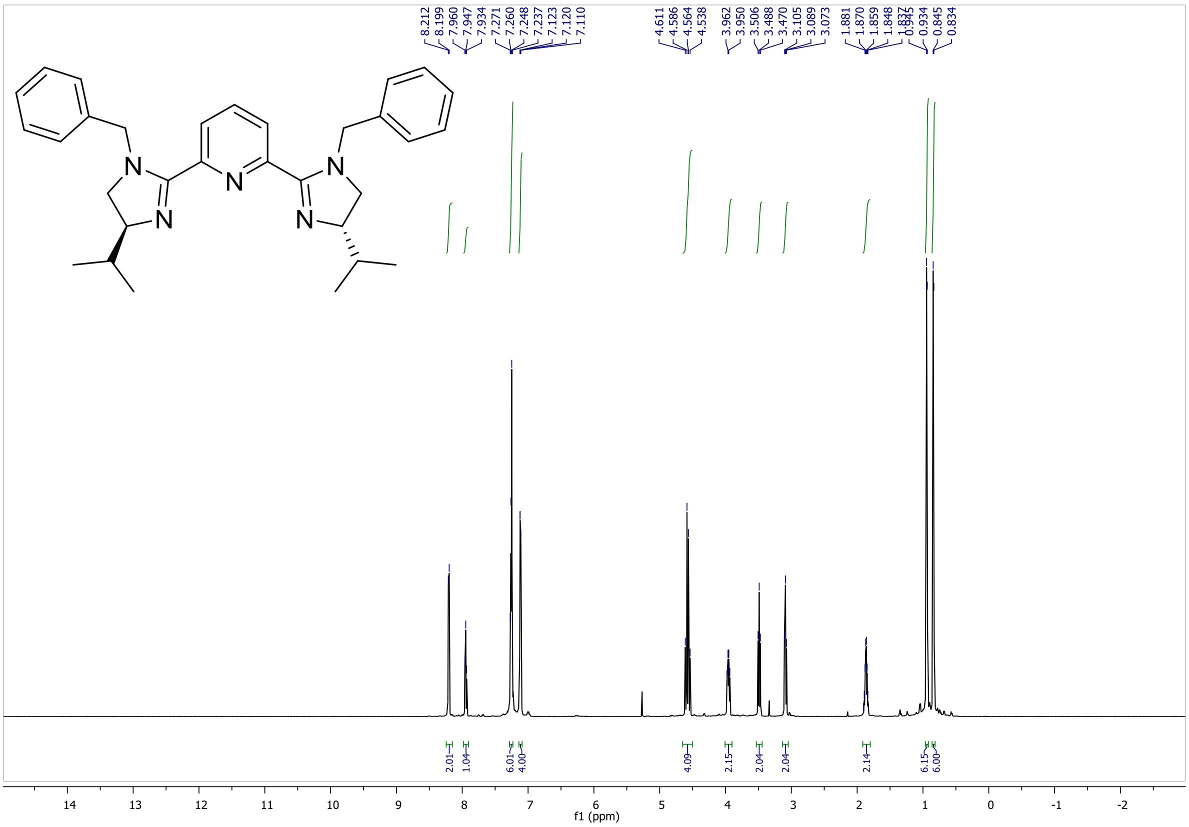

^{13}C NMR, 150 MHz, CDCl$_3$:

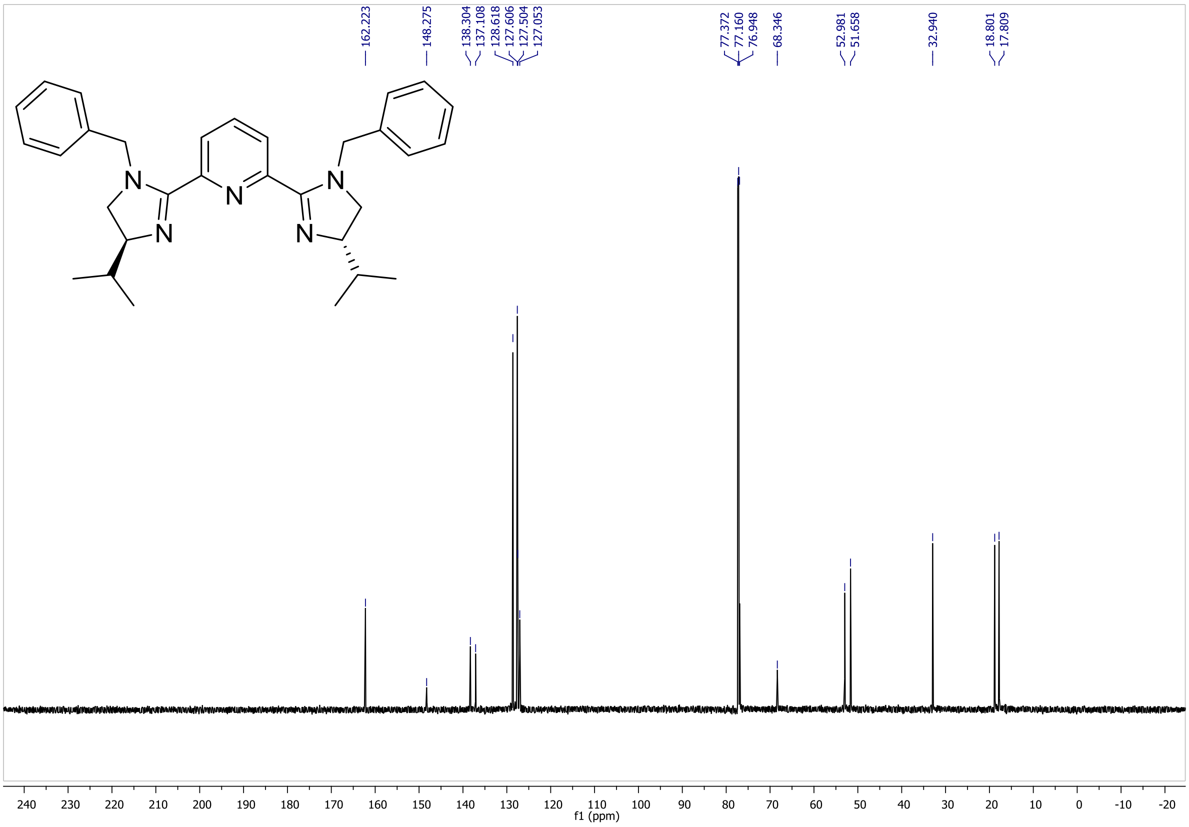

2,2'-((1*E*,1'*E*)-(((3*S*,4*S*)-2,5-dimethylhexane-3,4-diyl)bis(azanylylidene))bis(methanylylidene))diphenol:

^{1}H NMR, 600 MHz, $CDCl_3$:

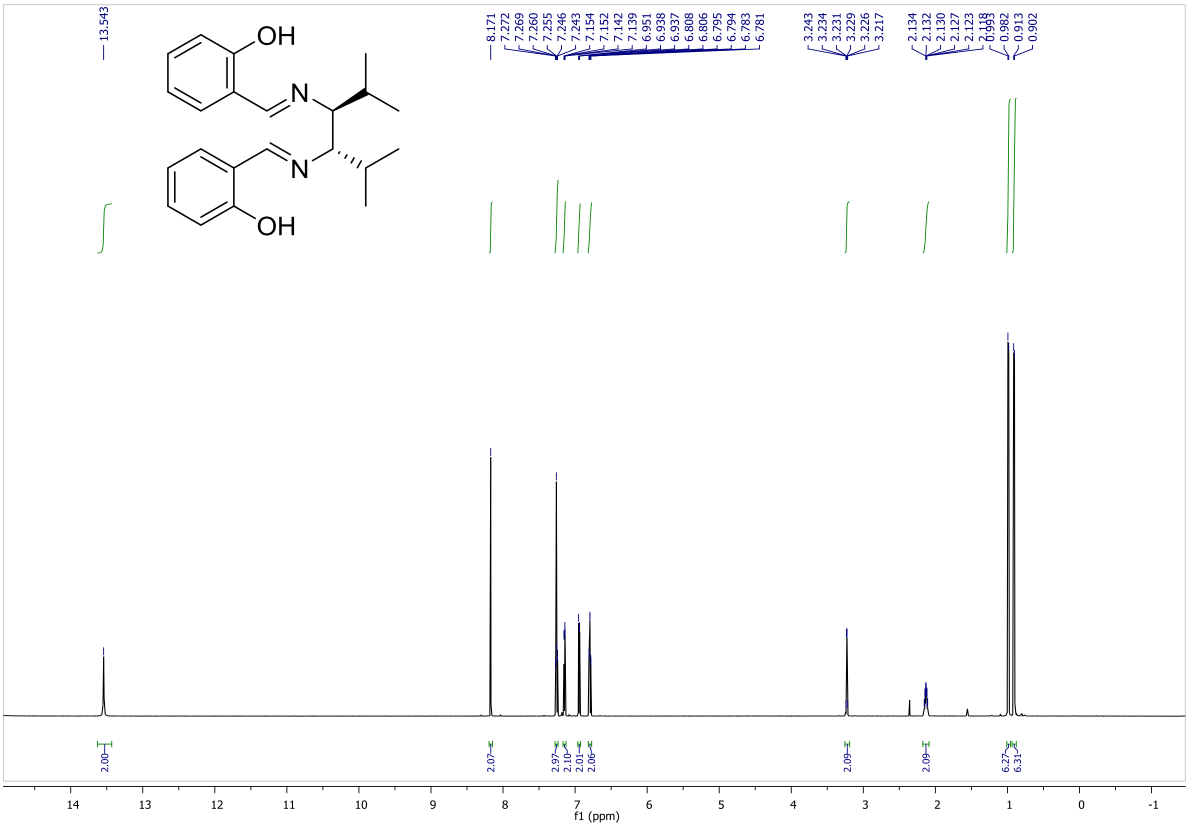

^{13}C NMR, 150 MHz, $CDCl_3$:

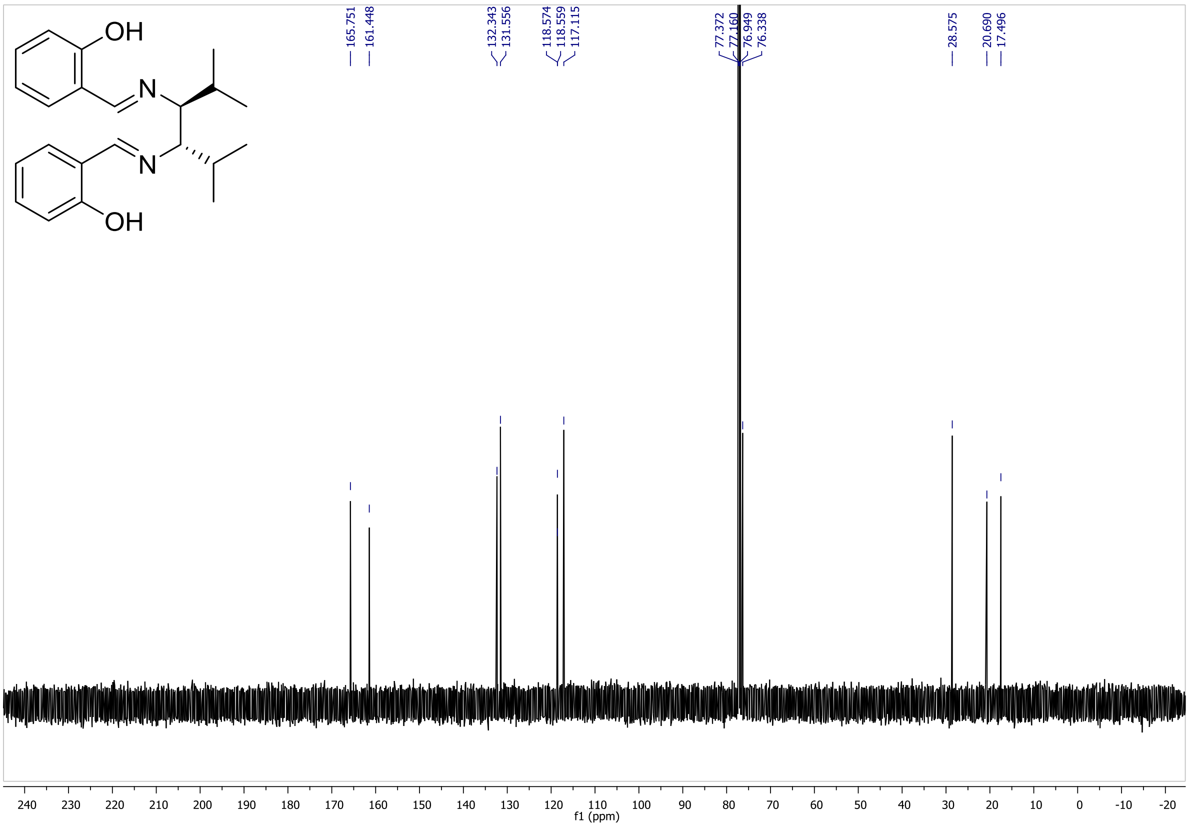

(3*S*,4*S*)-2,5-dimethylhexane-3,4-diammonium dichloride:

^{1}H NMR, 600 MHz, d_6-DMSO:

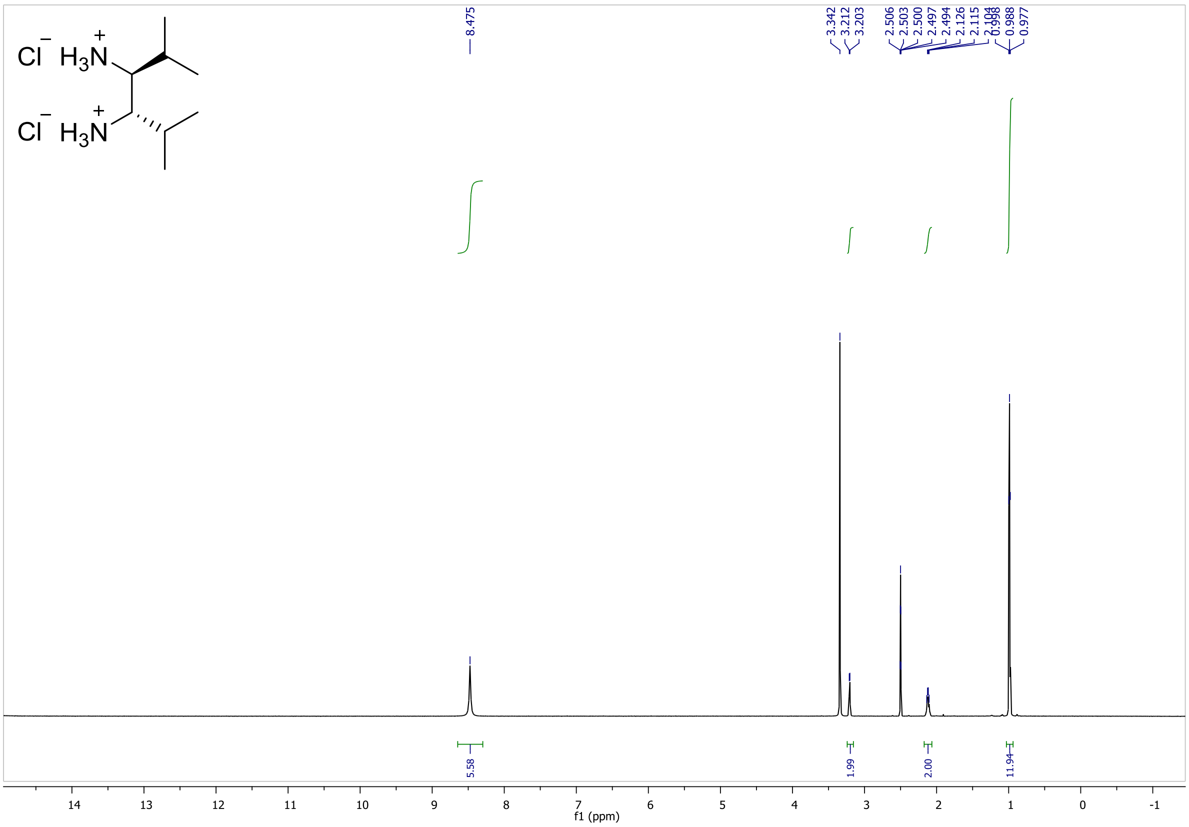

^{13}C NMR, 150 MHz, d_6-DMSO:

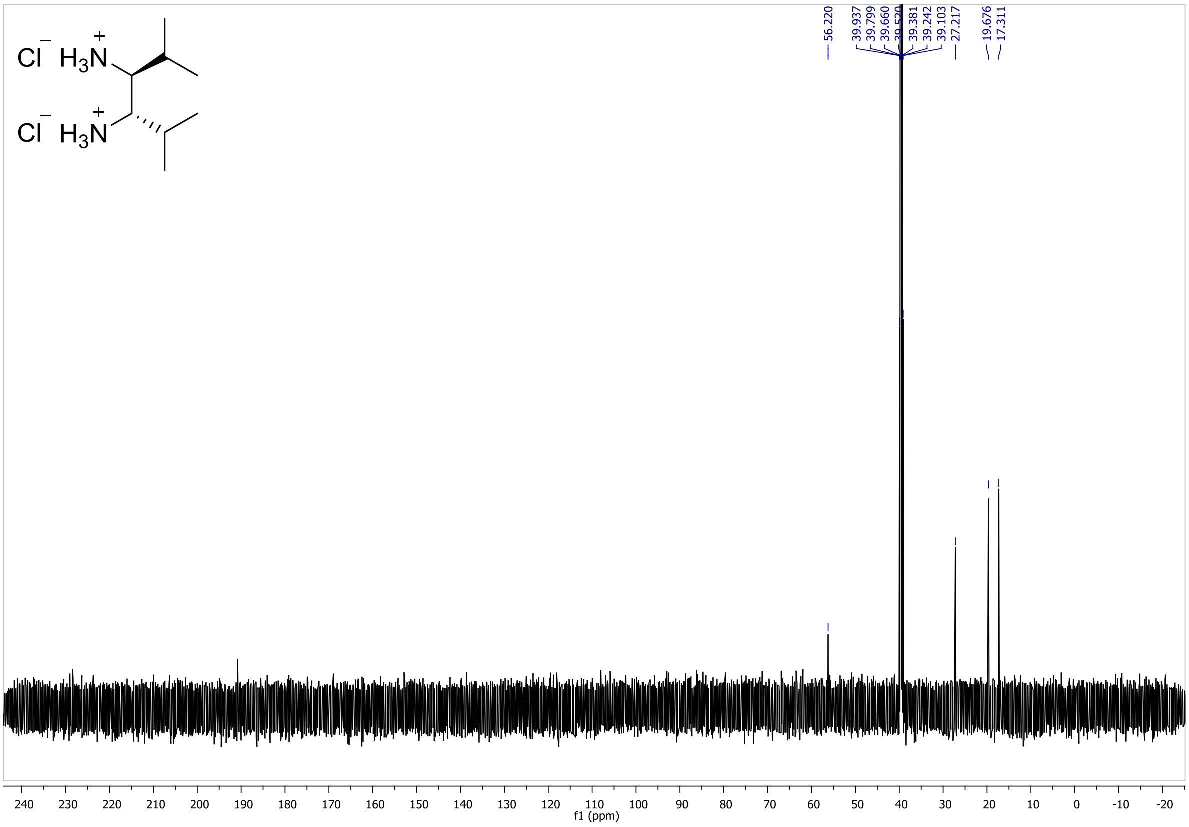

Dimethyl pyridine-2,6-bis(carbimidate):

^{1}H NMR, 600 MHz, $CDCl_3$:

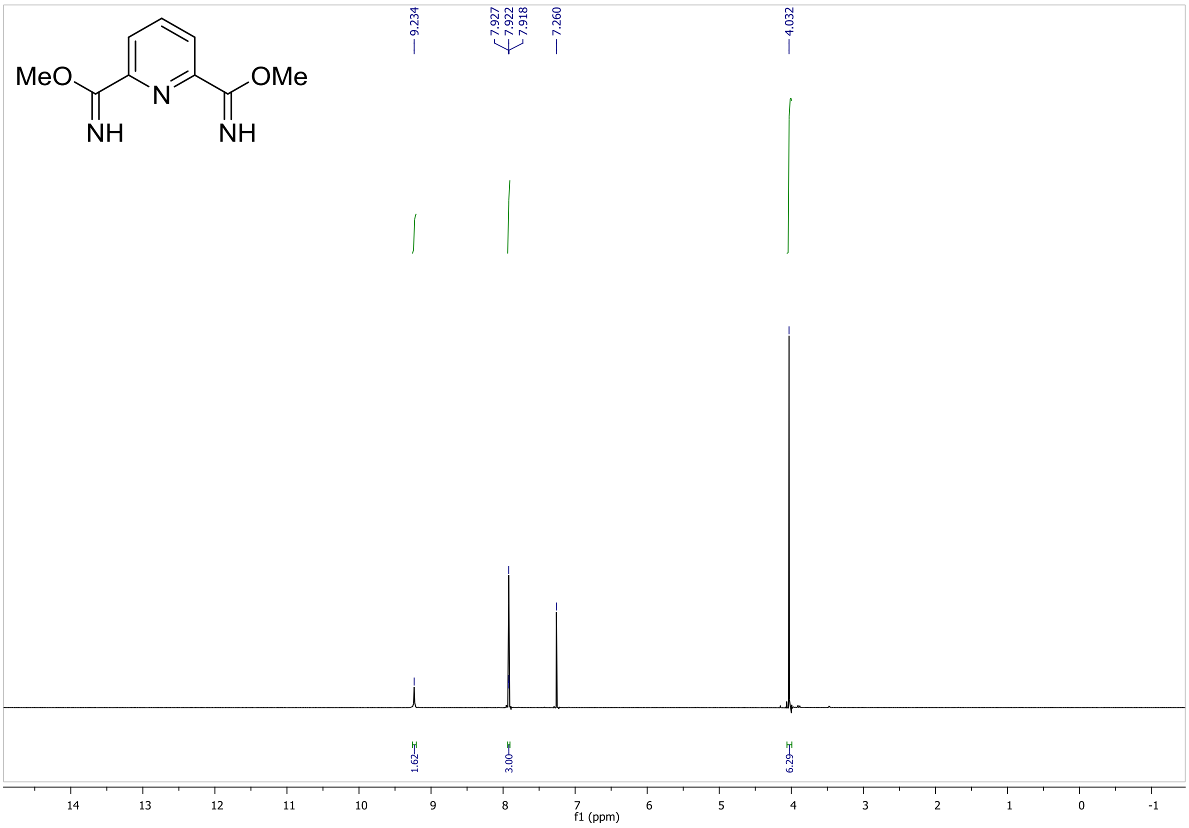

^{13}C NMR, 150 MHz, $CDCl_3$:

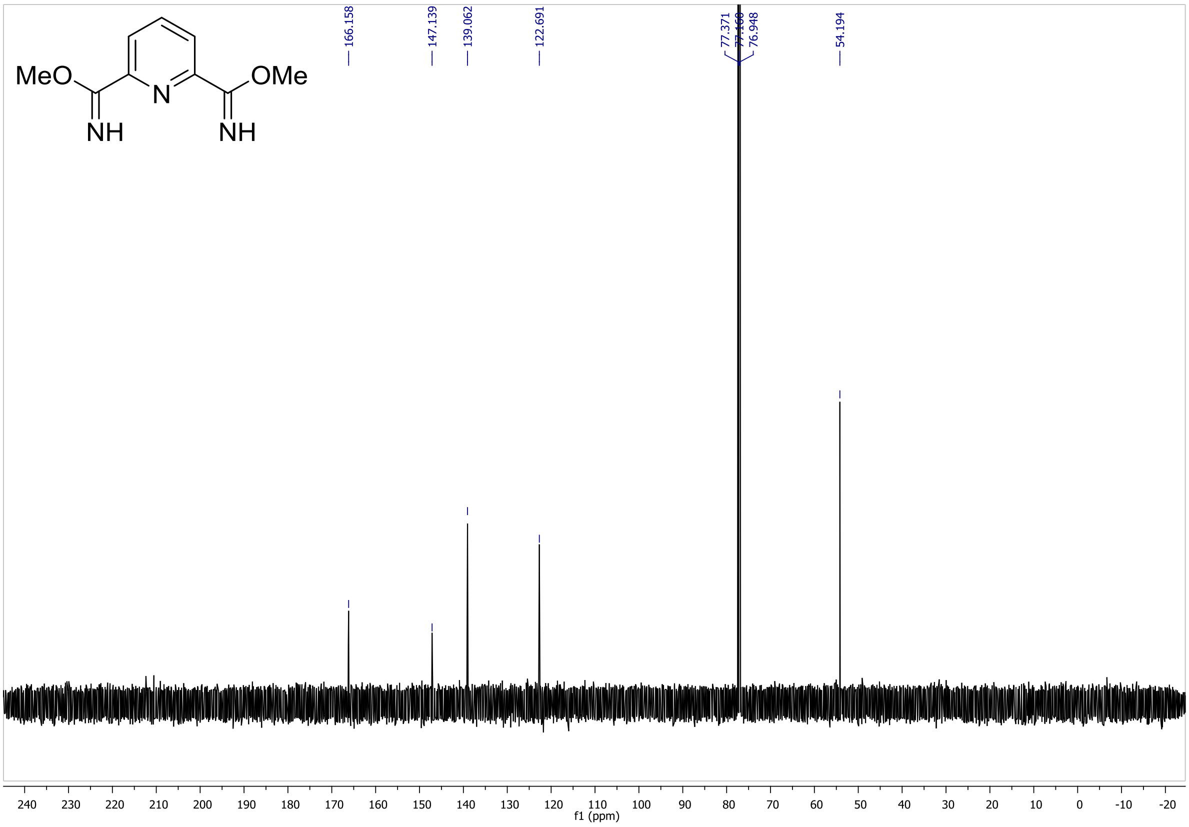

2,6-bis((4*S*,5*S*)-4,5-diisopropyl-4,5-dihydro-1*H*-imidazol-2-yl)pyridine:

^{1}H NMR, 600 MHz, $CDCl_3$:

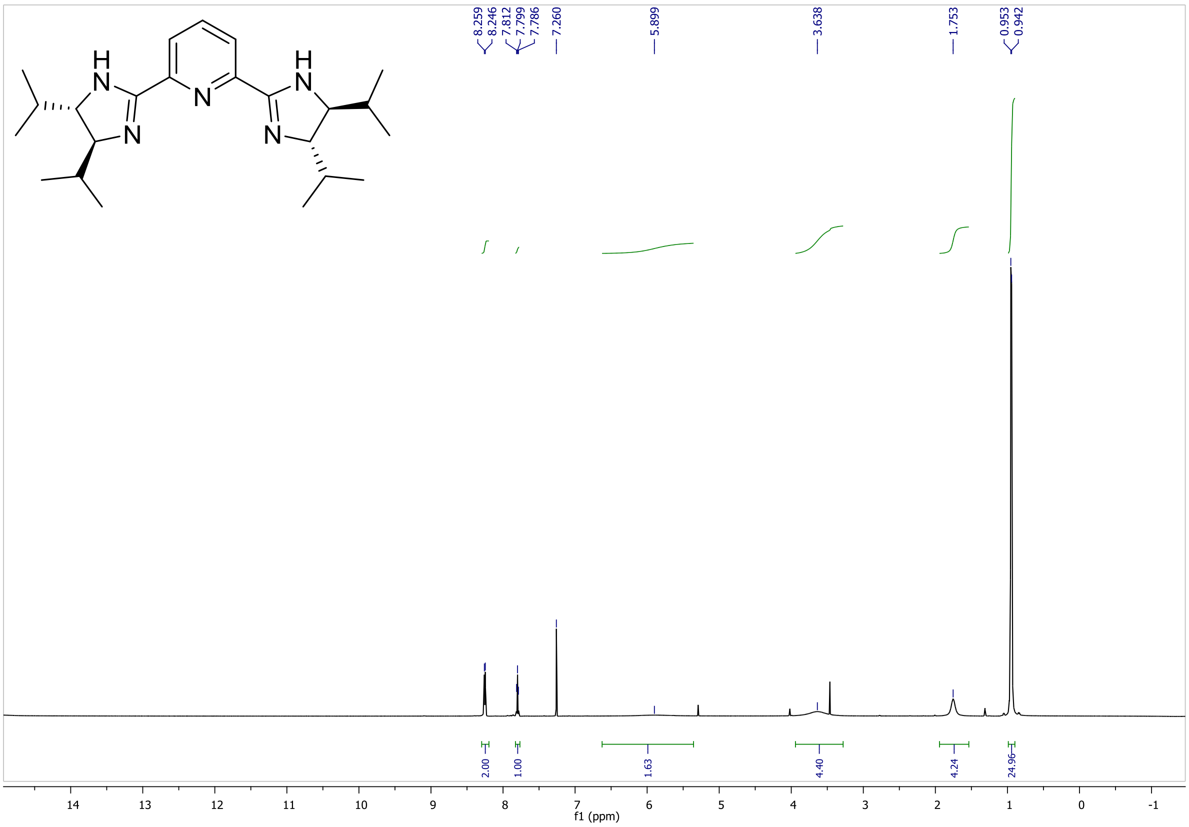

^{13}C NMR, 150 MHz, $CDCl_3$:

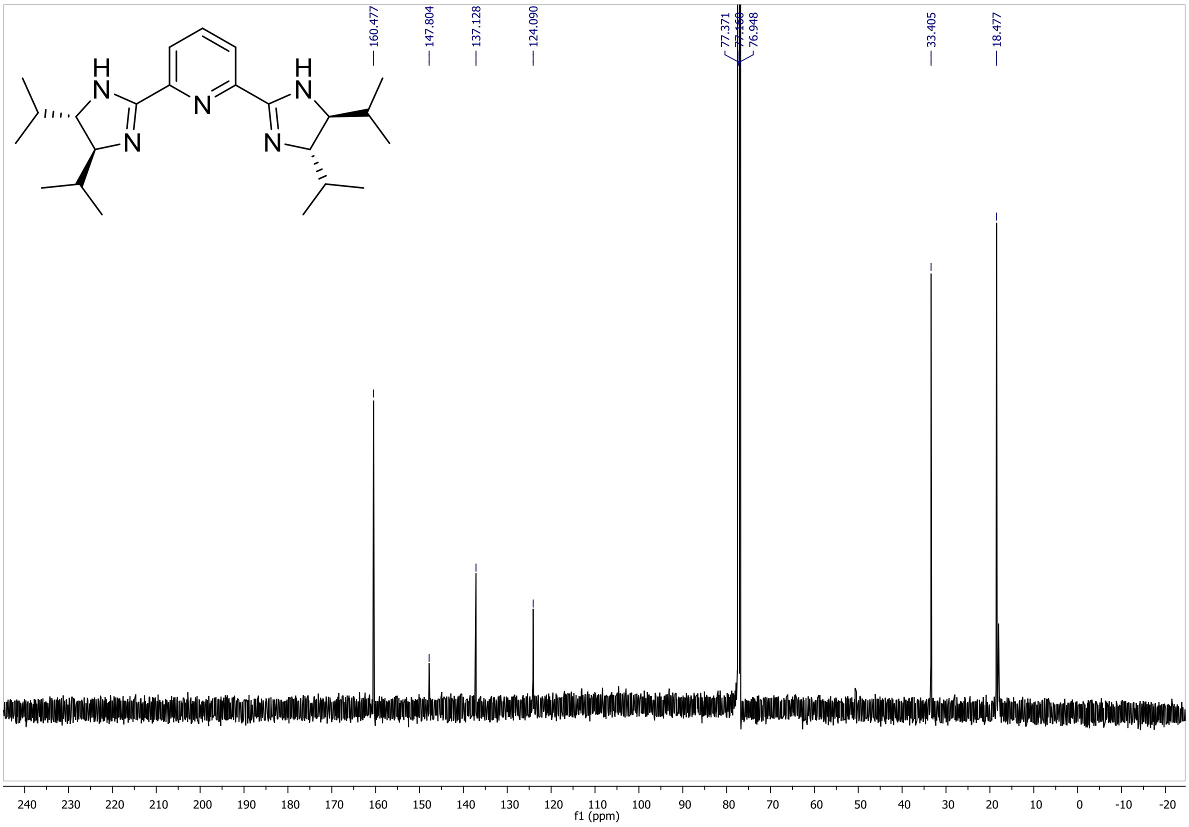

2,6-bis((4*S*,5*S*)-1-benzyl-4,5-diisopropyl-4,5-dihydro-1*H*-imidazol-2-yl)pyridine:

^{1}H NMR, 600 MHz, $CDCl_3$:

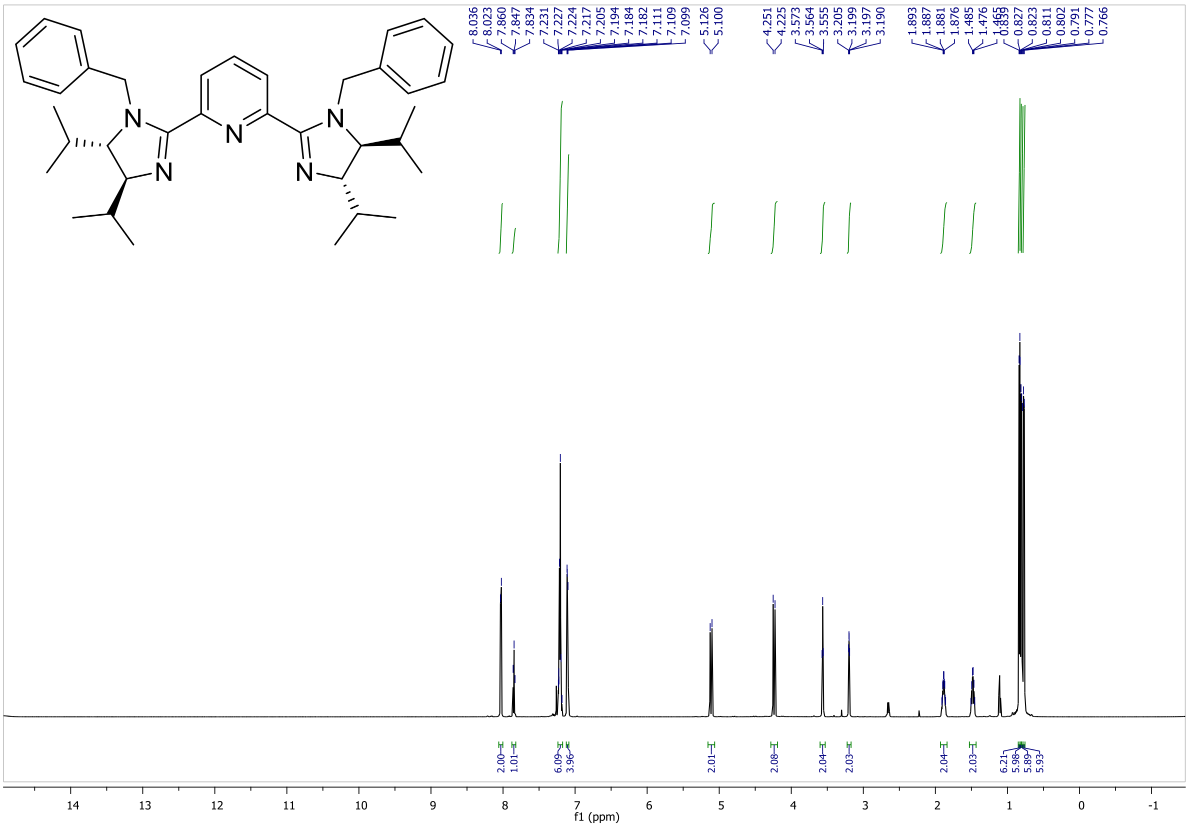

^{13}C NMR, 150 MHz, $CDCl_3$:

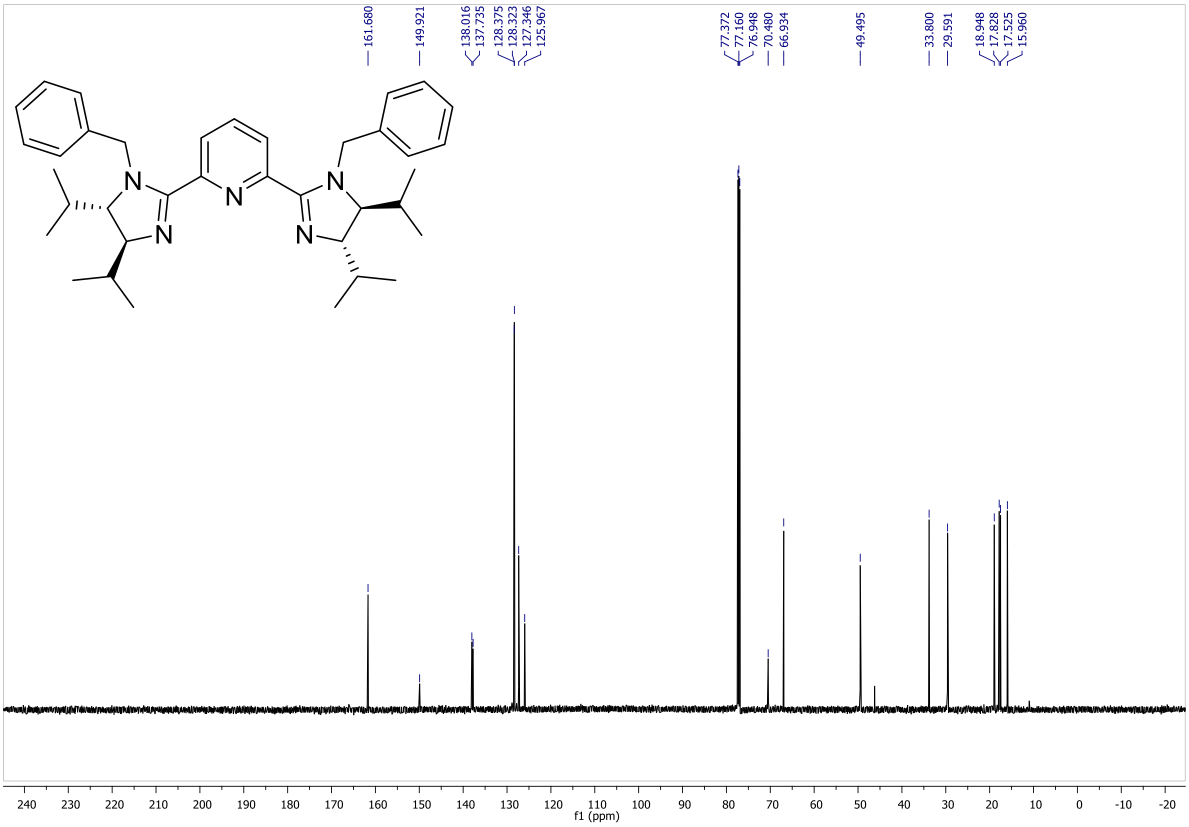

N^2,N^6-bis((*S*)-1-hydroxy-3,3-dimethylbutan-2-yl)pyridine-2,6-dicarboxamide:

^{1}H NMR, 600 MHz, $CDCl_3$:

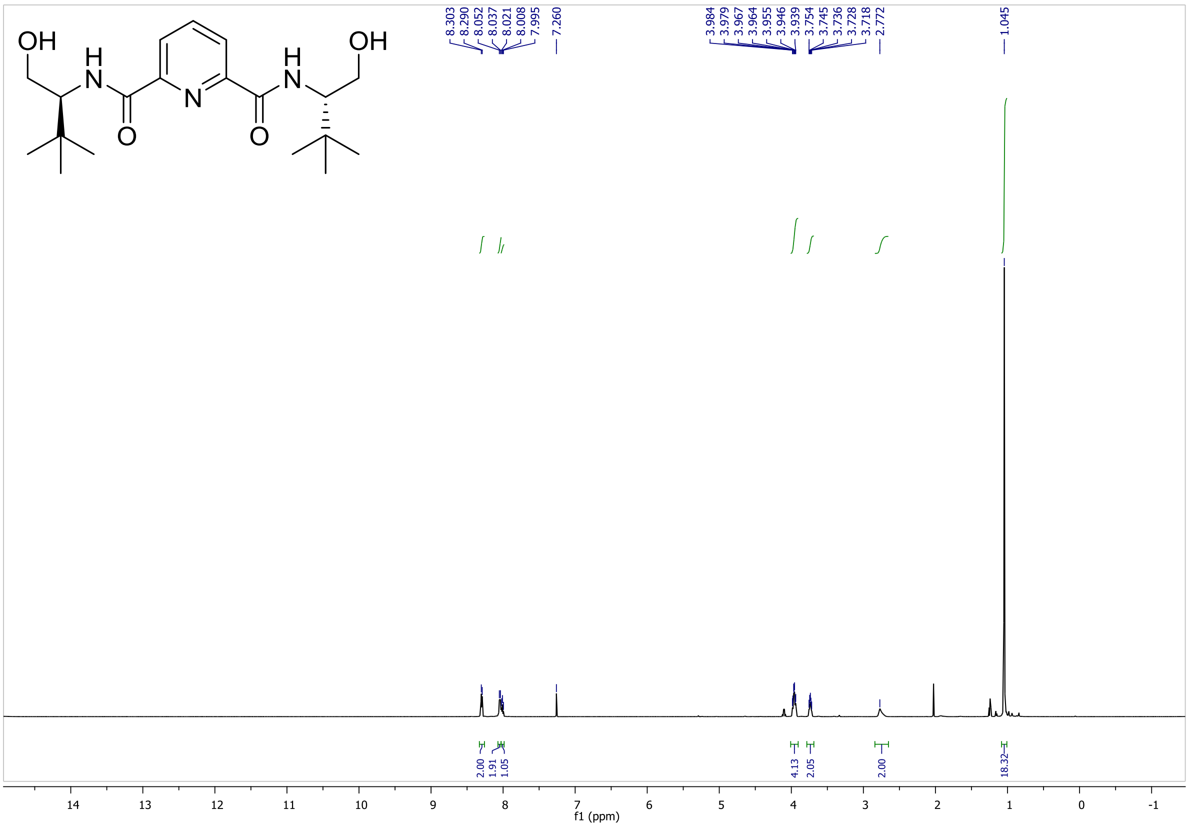

^{13}C NMR, 150 MHz, $CDCl_3$:

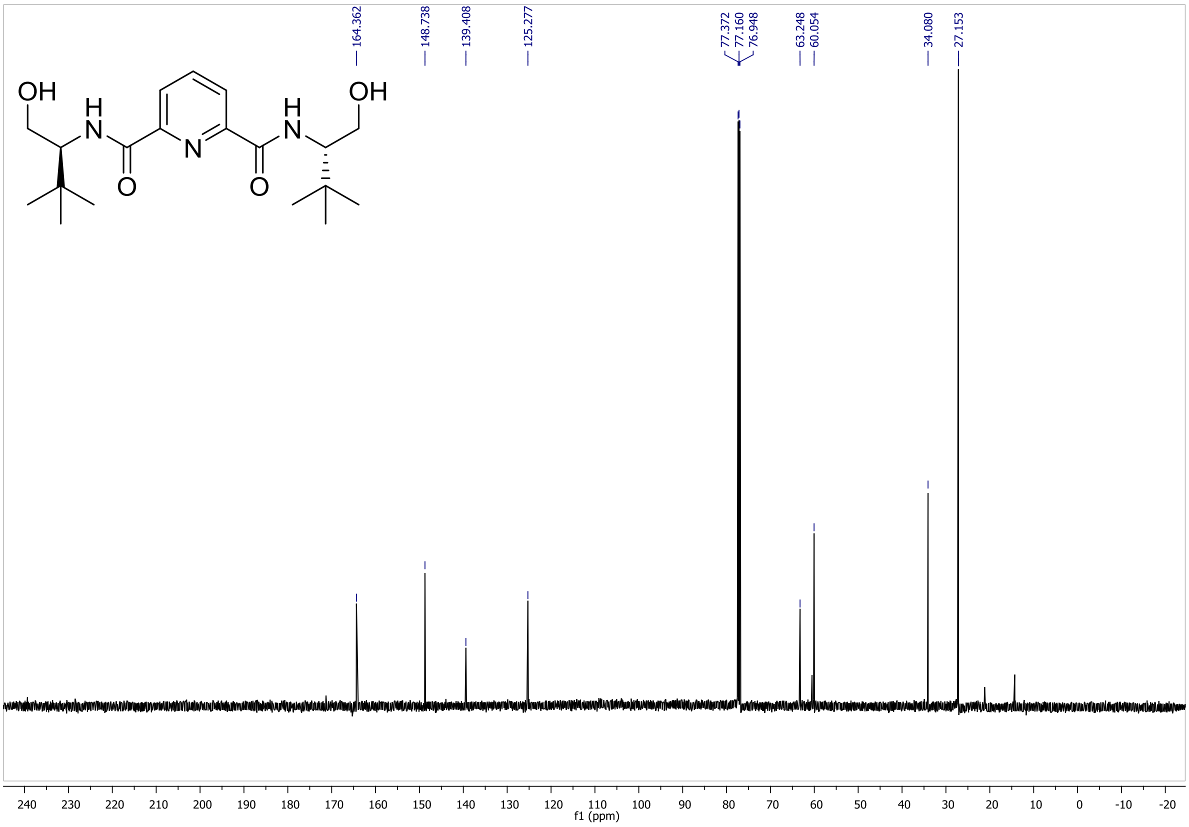

2,6-bis((*S*)-1-benzyl-4-(*tert*-butyl)-4,5-dihydro-1*H*-imidazol-2-yl)pyridine:

^{1}H NMR, 600 MHz, CDCl$_3$:

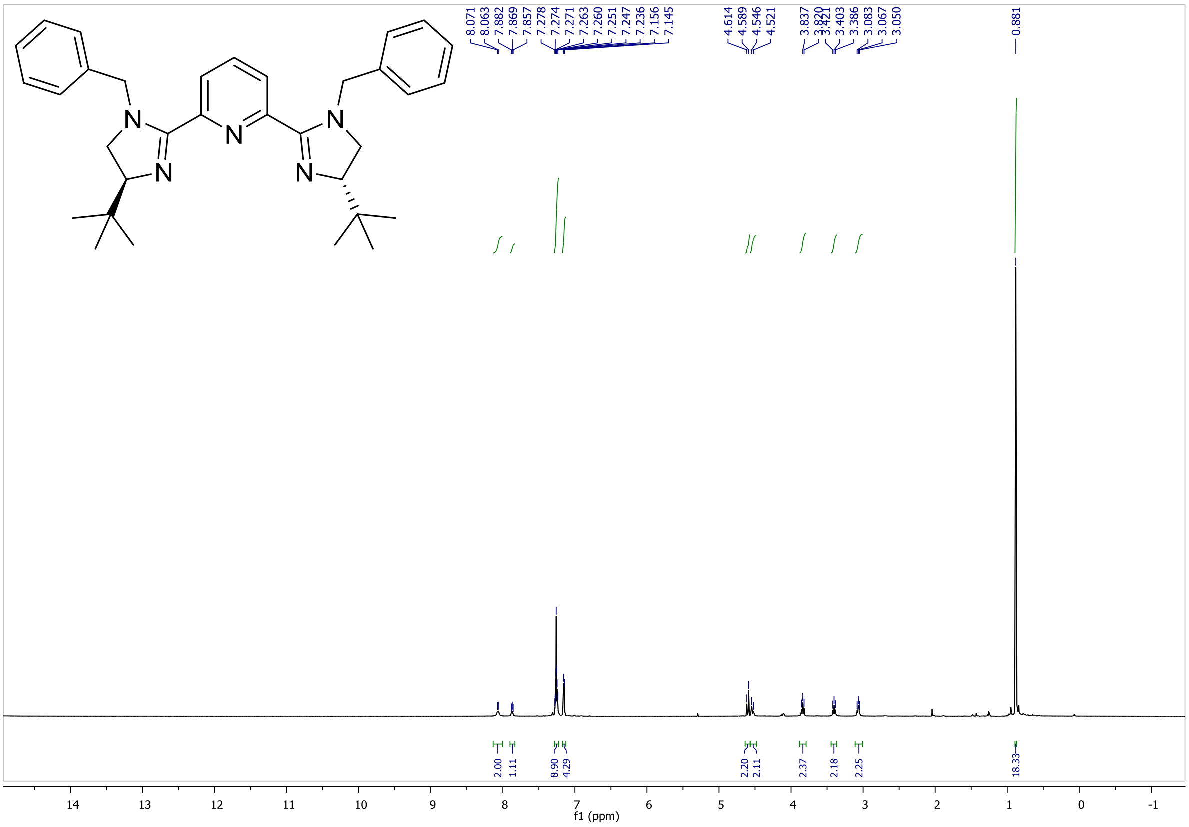

^{13}C NMR, 150 MHz, CDCl$_3$:

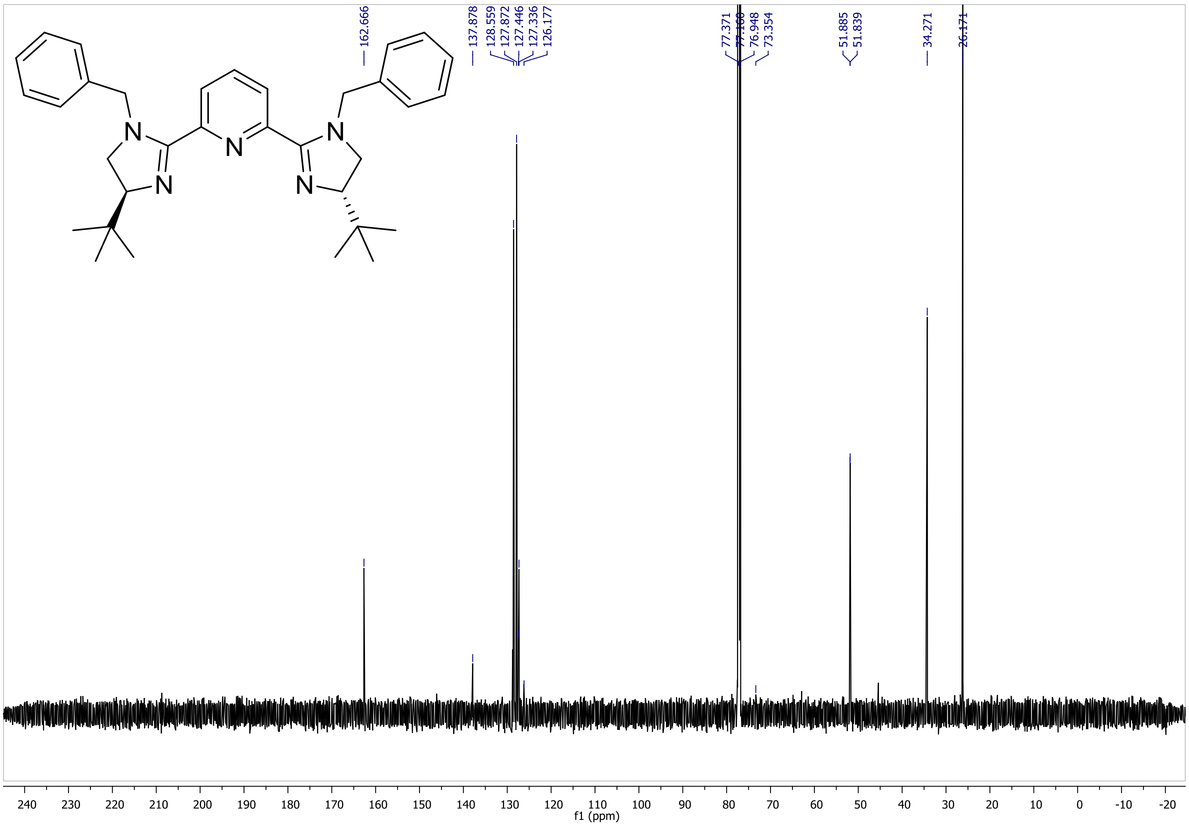

2,6-bis((*S*)-4-(*tert*-butyl)-1-((*R*)-1-phenylethyl)-4,5-dihydro-1*H*-imidazol-2-yl)pyridine:

^{1}H NMR, 600 MHz, $CDCl_3$:

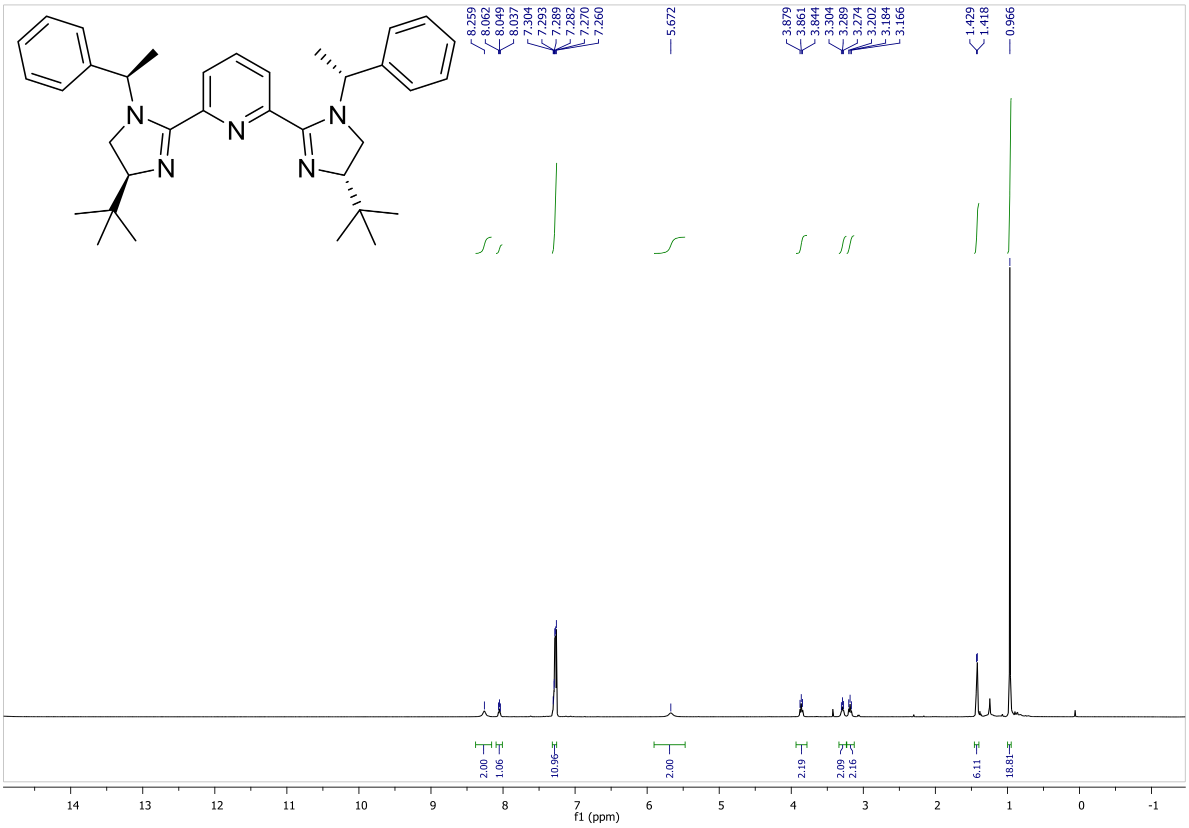

^{13}C NMR, 150 MHz, $CDCl_3$:

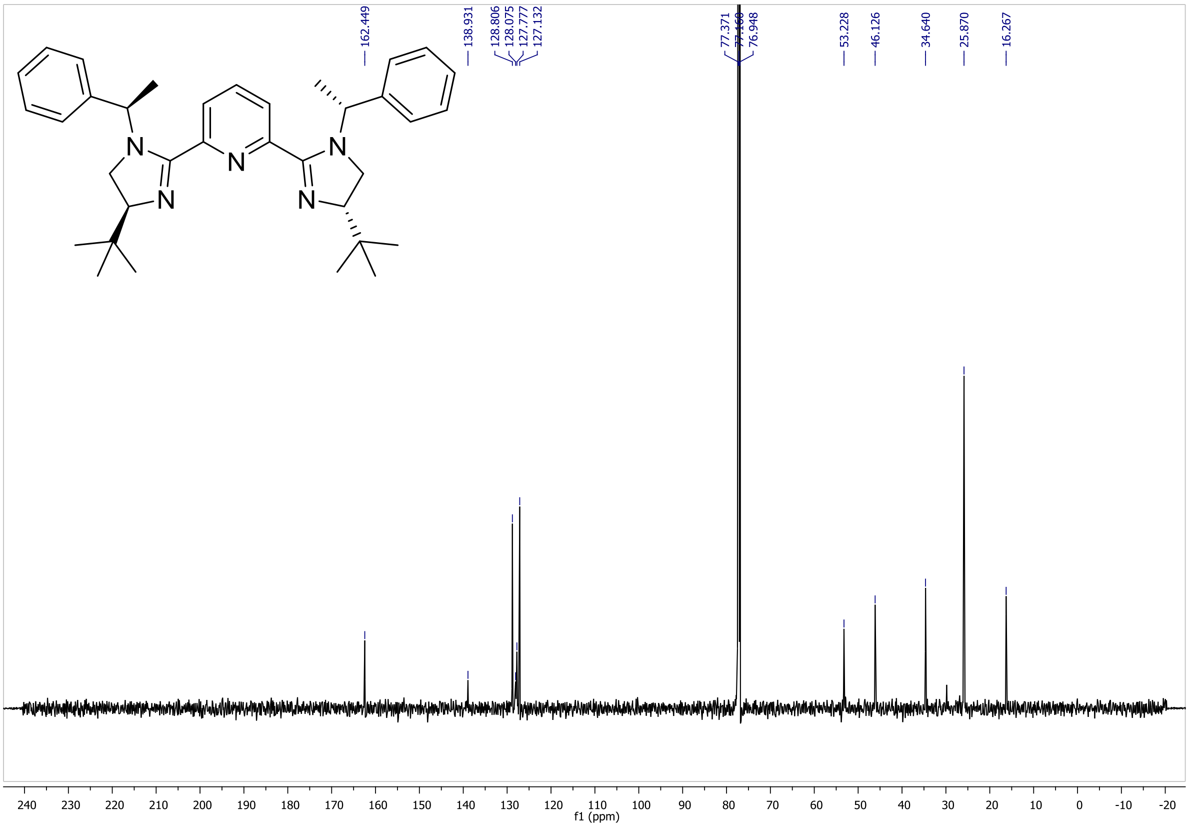

2,6-bis((*S*)-4-(*tert*-butyl)-1-phenyl-4,5-dihydro-1*H*-imidazol-2-yl)pyridine (L4):

^{1}H NMR, 600 MHz, $CDCl_3$:

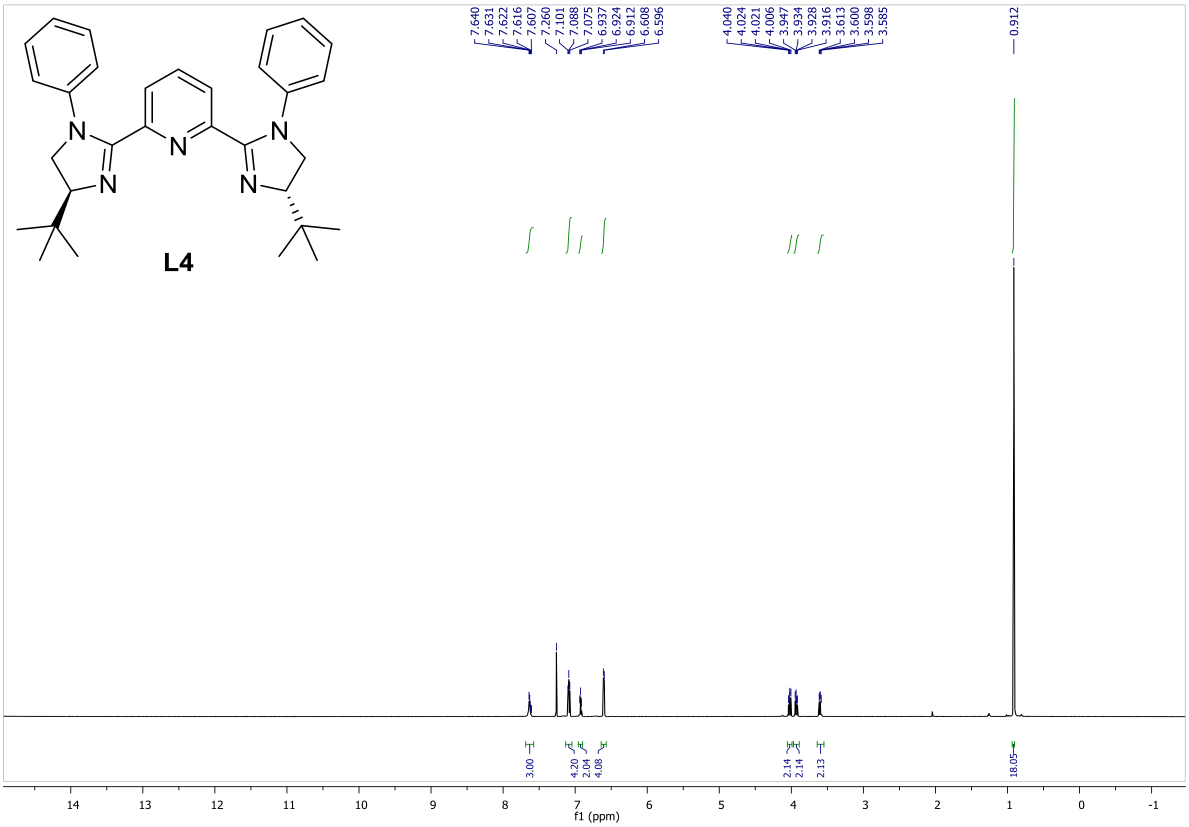

^{13}C NMR, 150 MHz, $CDCl_3$:

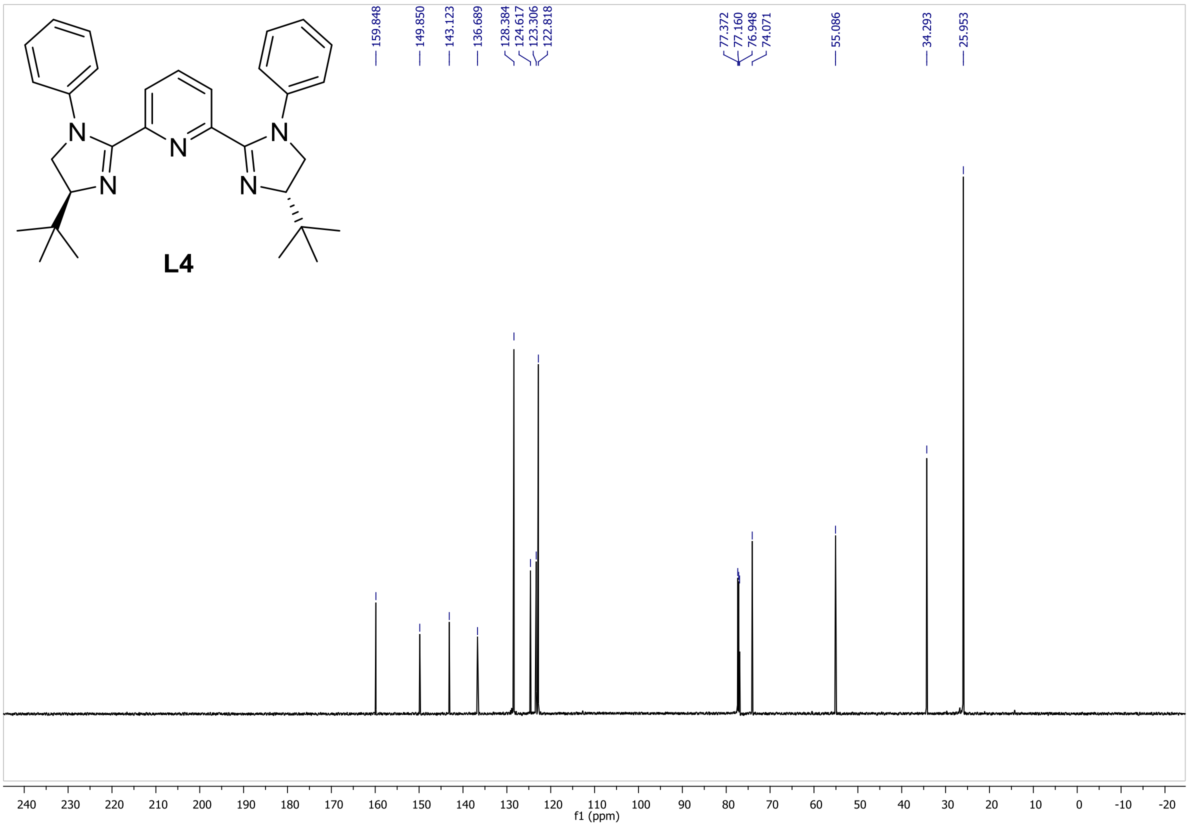

2,6-bis((*S*)-4-(*tert*-butyl)-1-mesityl-4,5-dihydro-1*H*-imidazol-2-yl)pyridine:

^{1}H NMR, 600 MHz, $CDCl_3$:

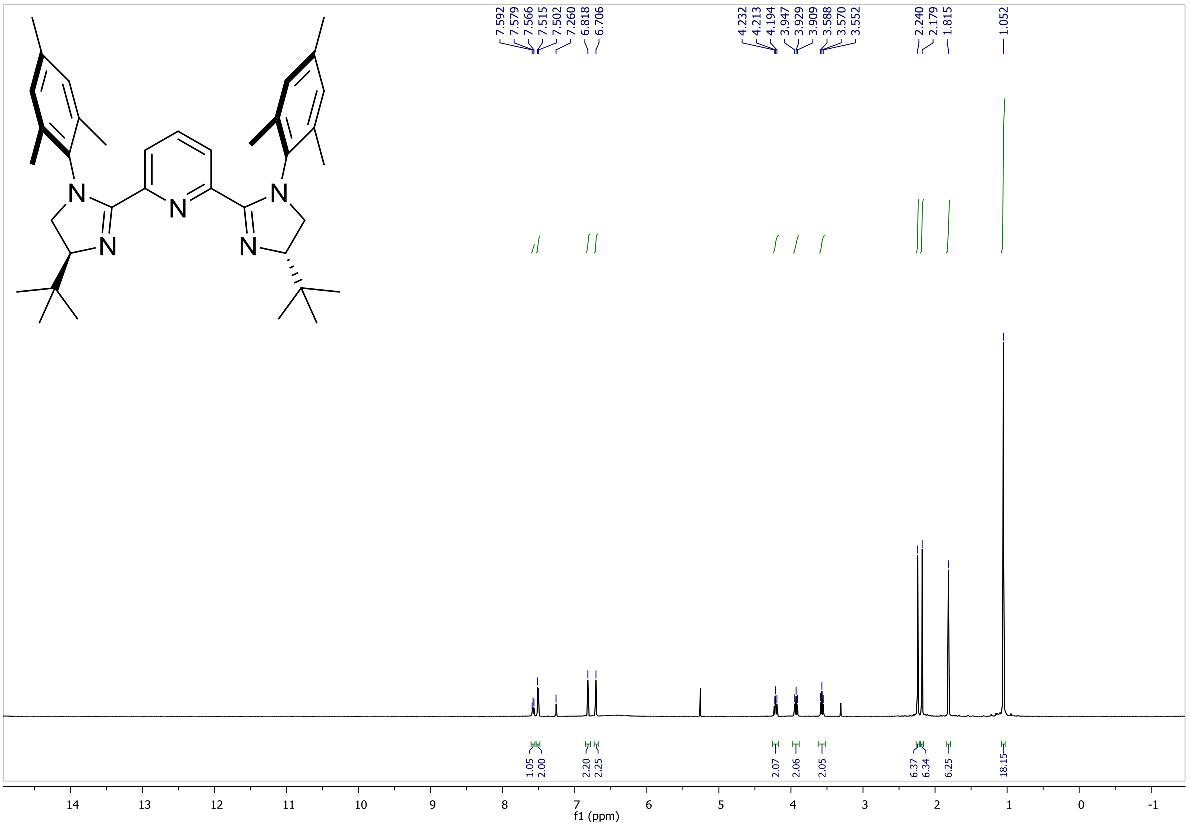

^{13}C NMR, 150 MHz, $CDCl_3$:

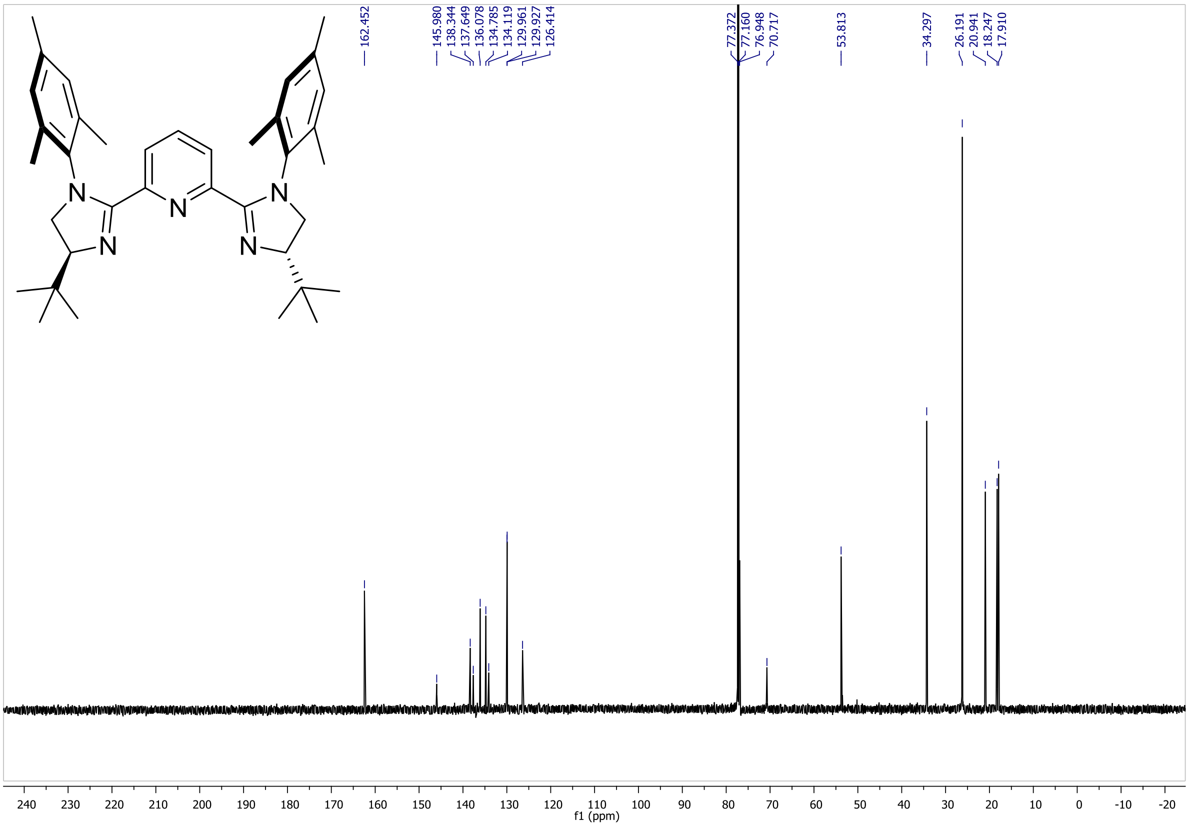

2,6-bis((*S*)-4-(*tert*-butyl)-1-(3,5-dimethylphenyl)-4,5-dihydro-1*H*-imidazol-2-yl)pyridine (L5):

^{1}H NMR, 600 MHz, $CDCl_3$:

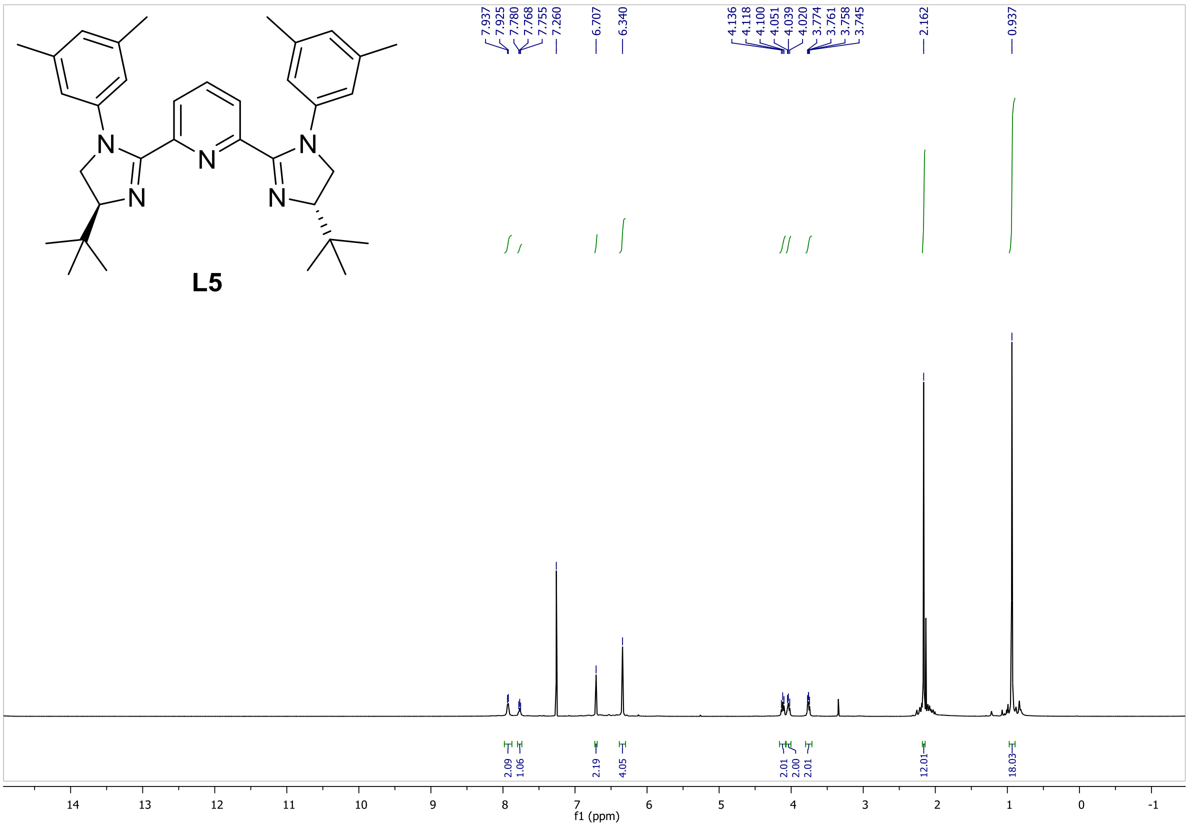

^{13}C NMR, 150 MHz, $CDCl_3$:

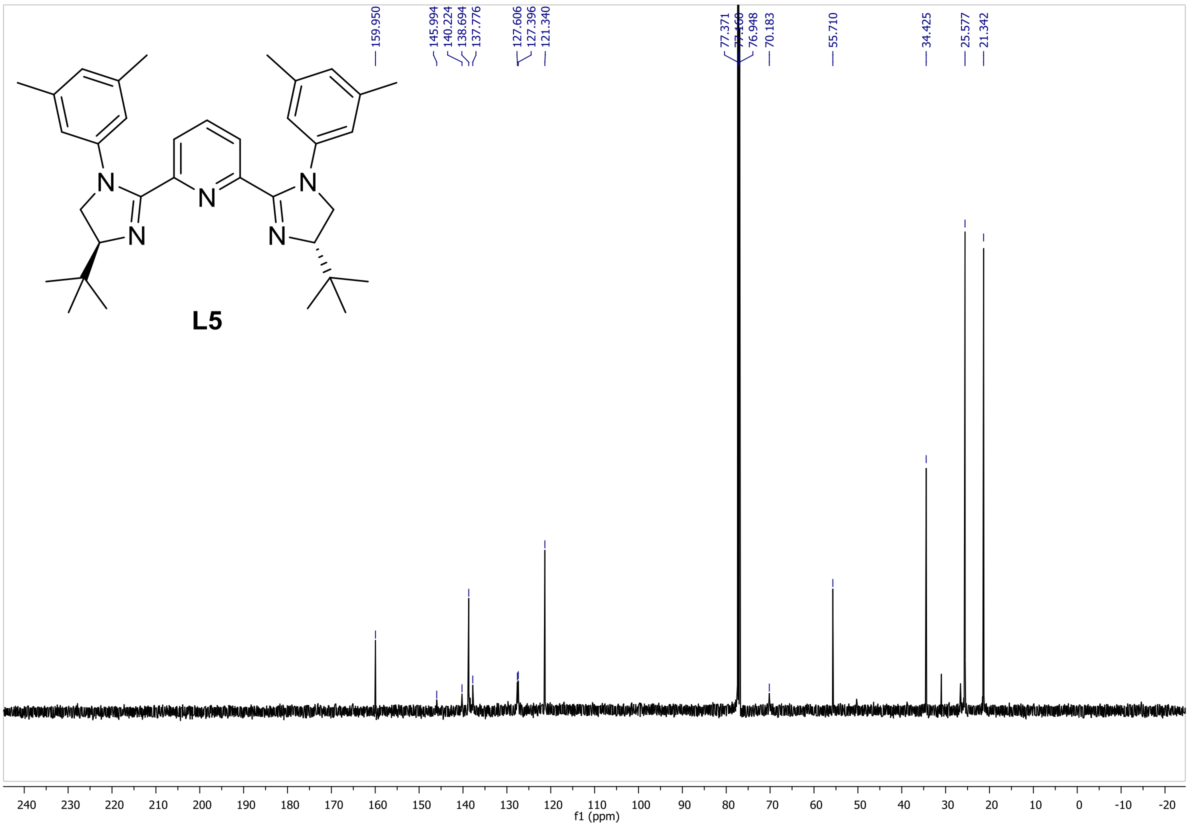

2,6-bis((*S*)-4-(*tert*-butyl)-1-(3,5-di-*tert*-butylphenyl)-4,5-dihydro-1*H*-imidazol-2-yl)pyridine (L6):

^{1}H NMR, 600 MHz, d$_4$-MeOD:

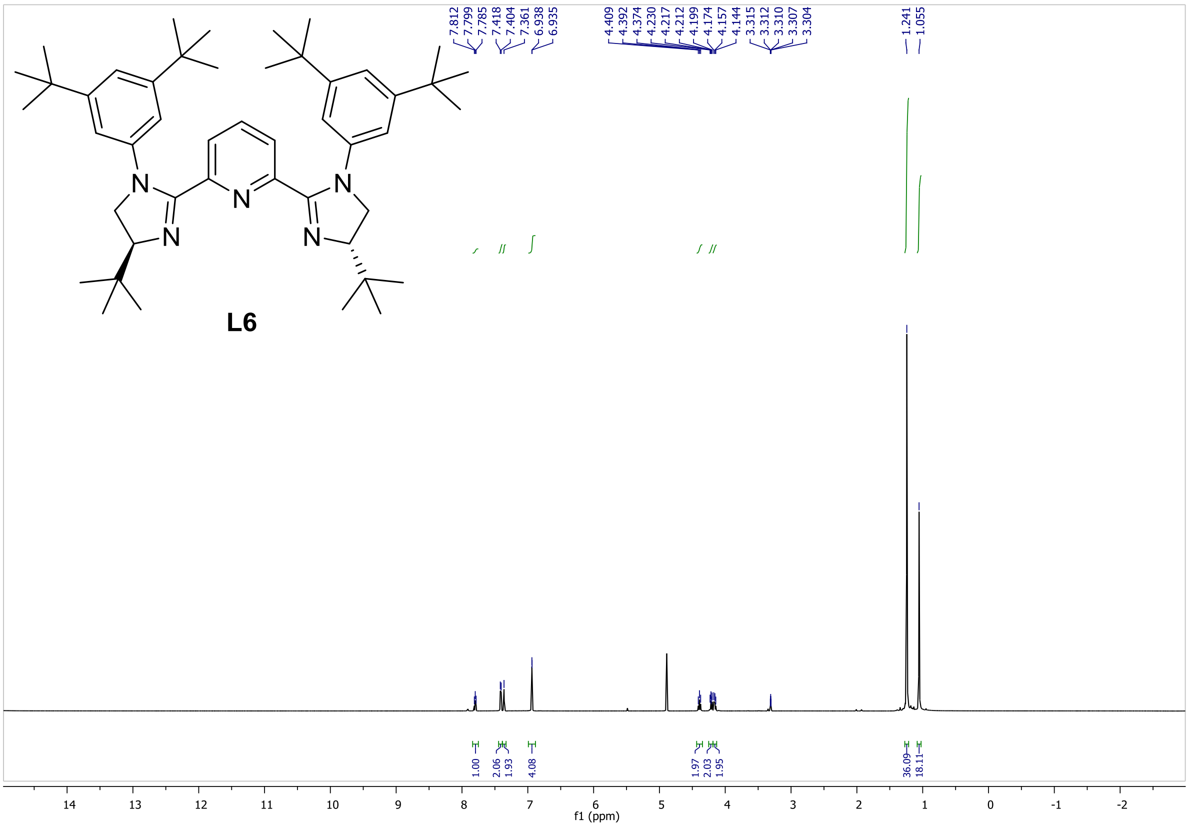

^{13}C NMR, 150 MHz, d$_4$-MeOD:

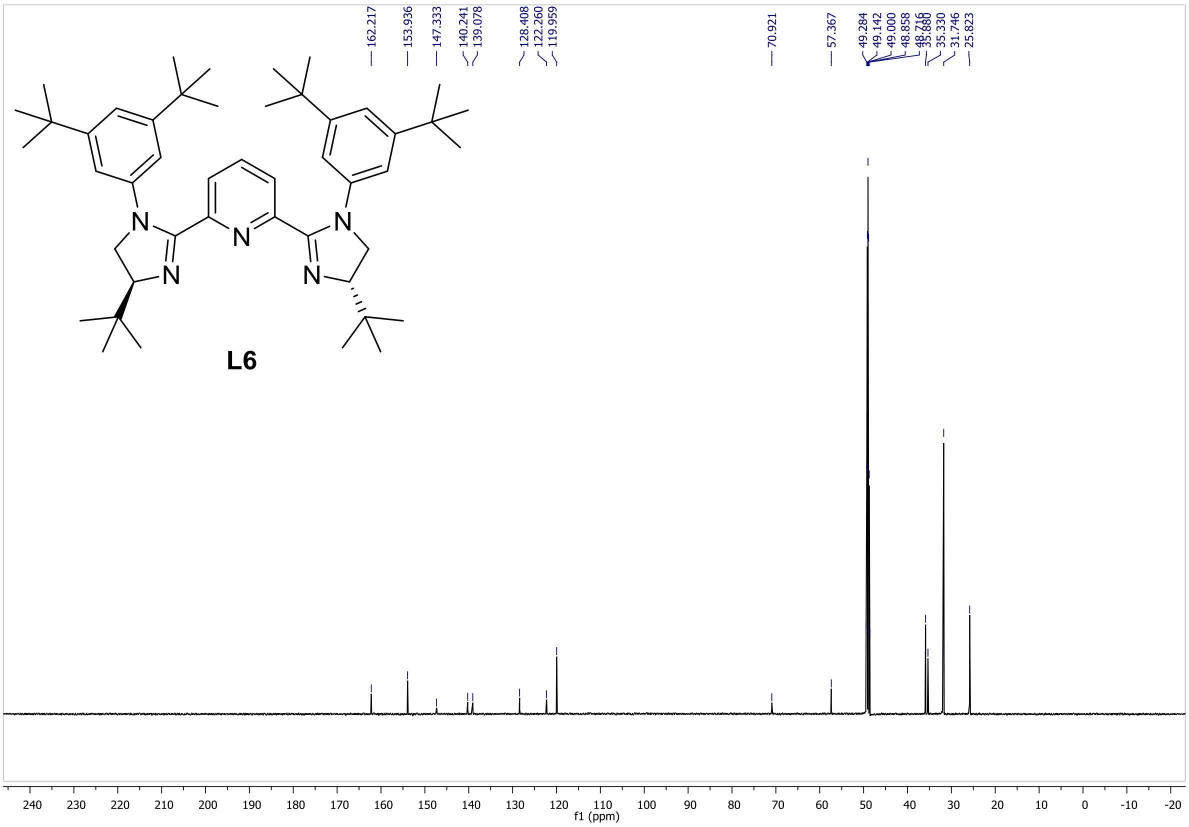

2,6-bis((*S*)-1-(3,5-bis(trifluoromethyl)phenyl)-4-(*tert*-butyl)-4,5-dihydro-1*H*-imidazol-2-yl)pyridine (L7):

^{1}H NMR, 600 MHz, $CDCl_3$:

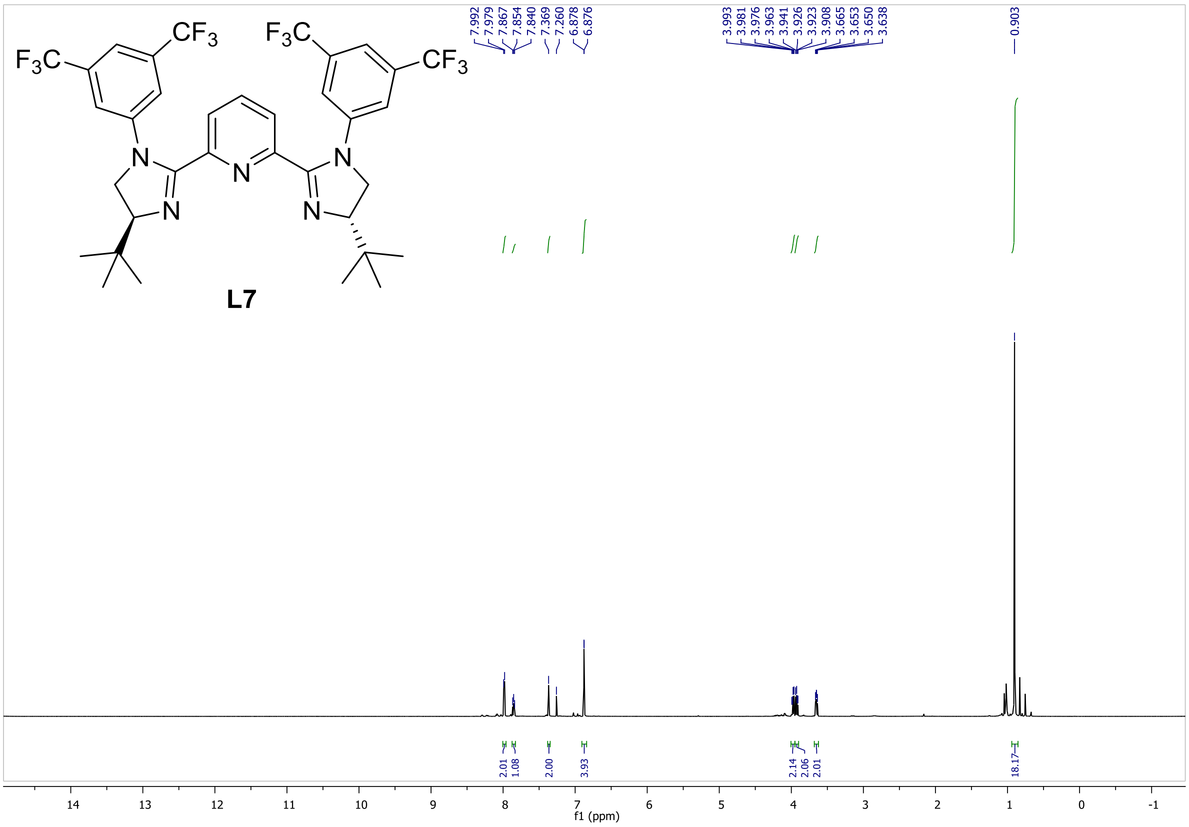

^{13}C NMR, 150 MHz, $CDCl_3$:

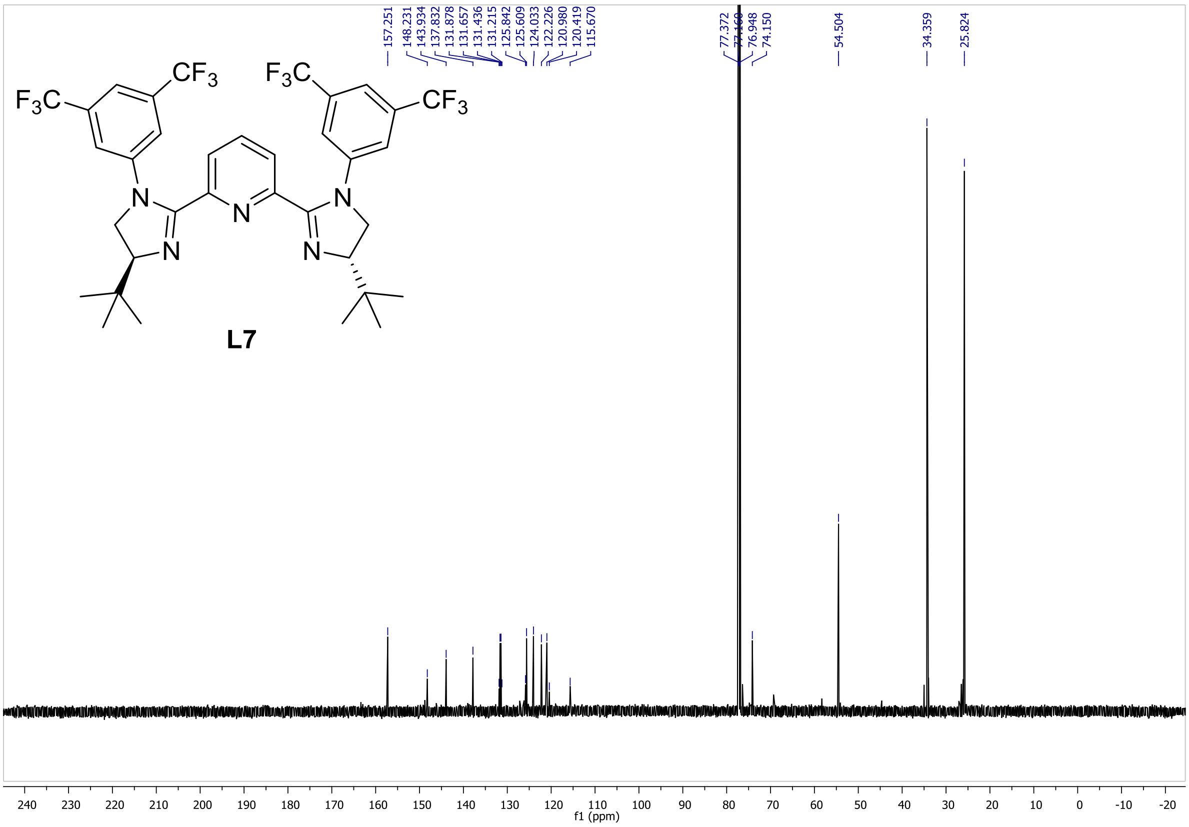

^{19}F NMR, 376 MHz, $CDCl_3$:

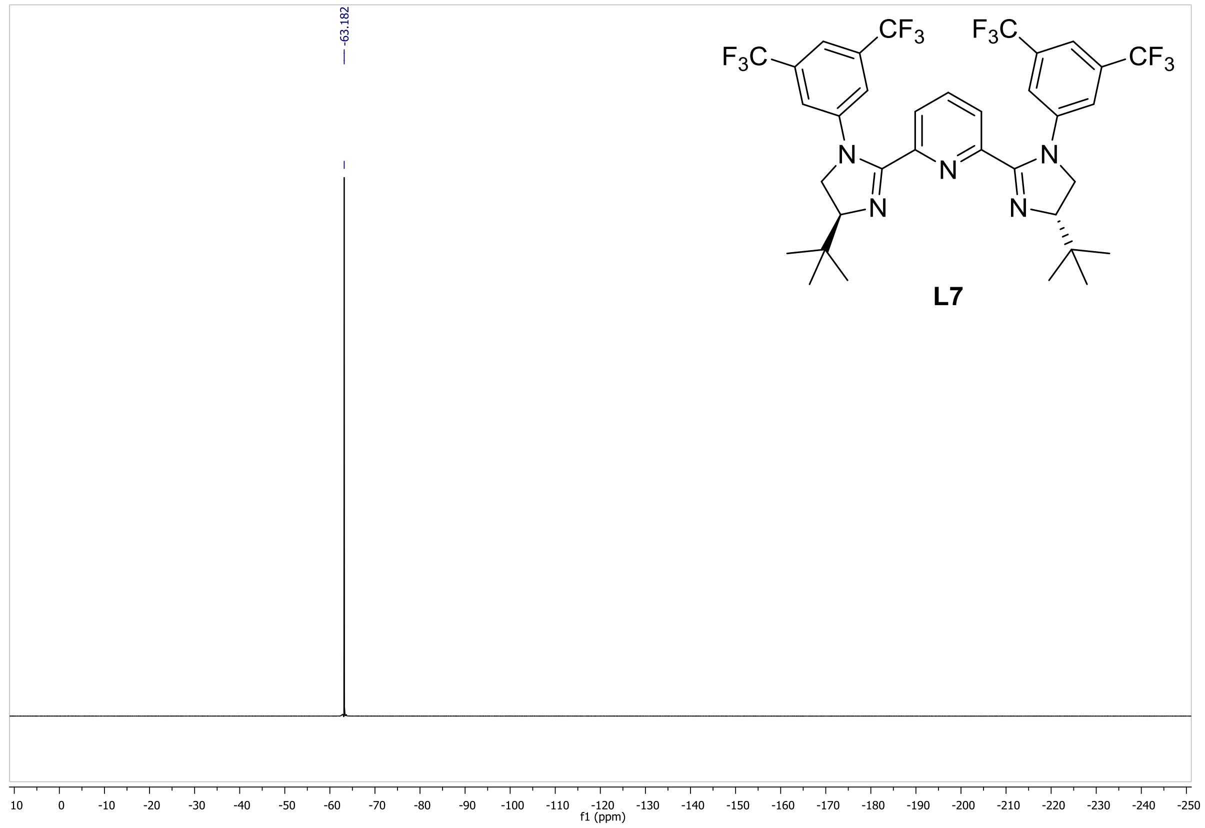

2,6-bis((*S*)-4-(*tert*-butyl)-1-(4-(trifluoromethyl)phenyl)-4,5-dihydro-1*H*-imidazol-2-yl)pyridine (L8):

^{1}H NMR, 600 MHz, $CDCl_3$:

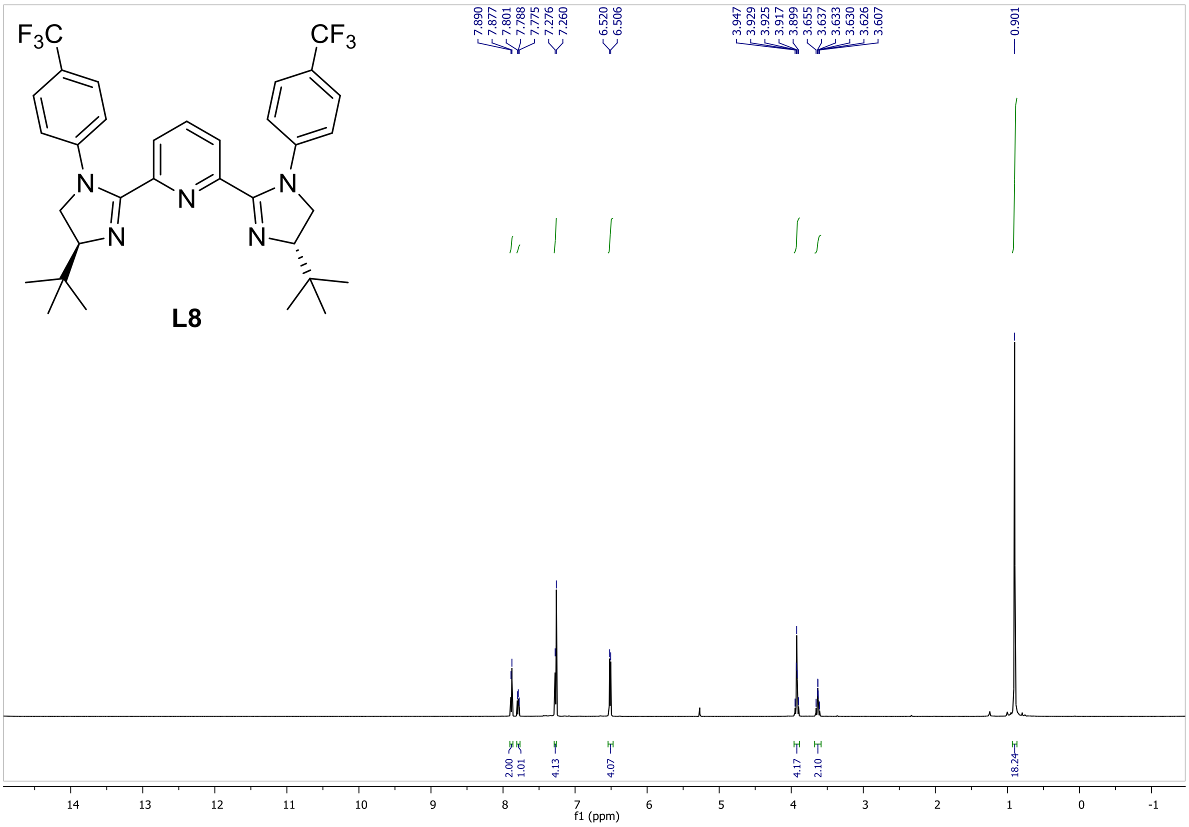

^{13}C NMR, 150 MHz, $CDCl_3$:

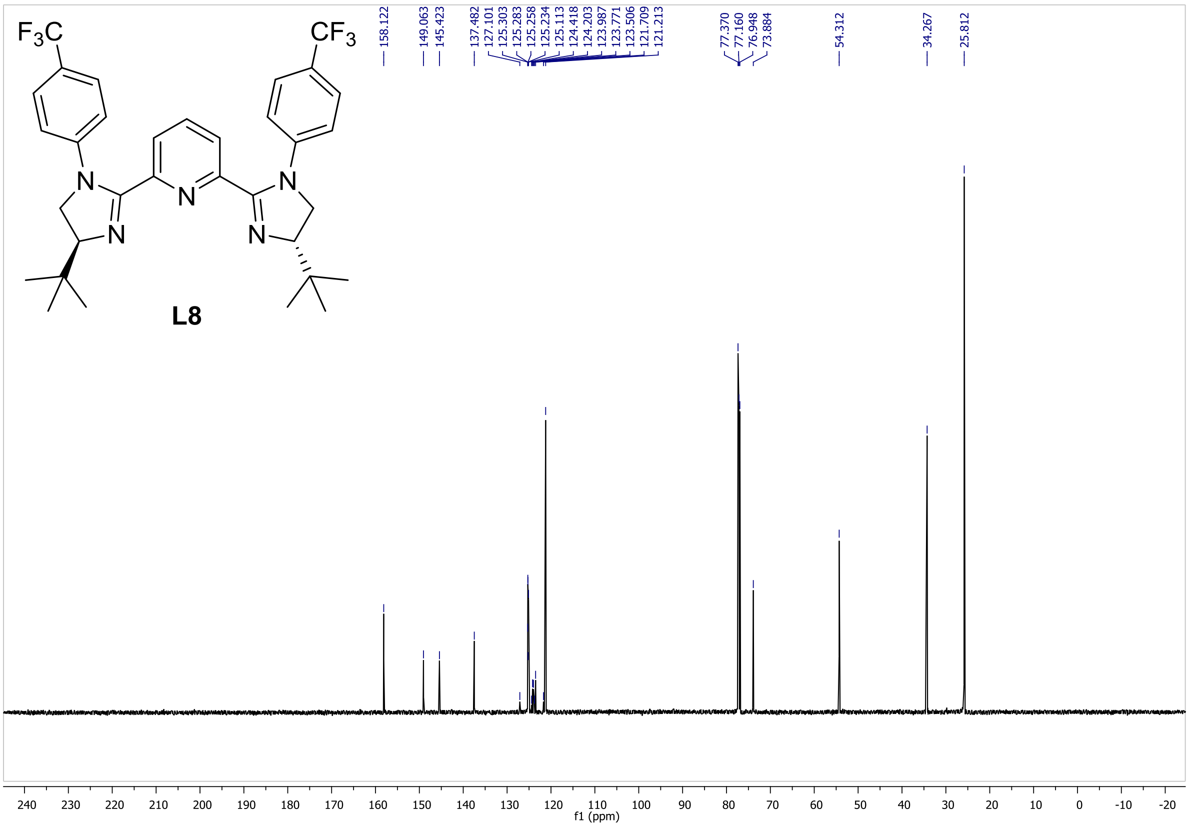

^{19}F NMR, 376 MHz, $CDCl_3$:

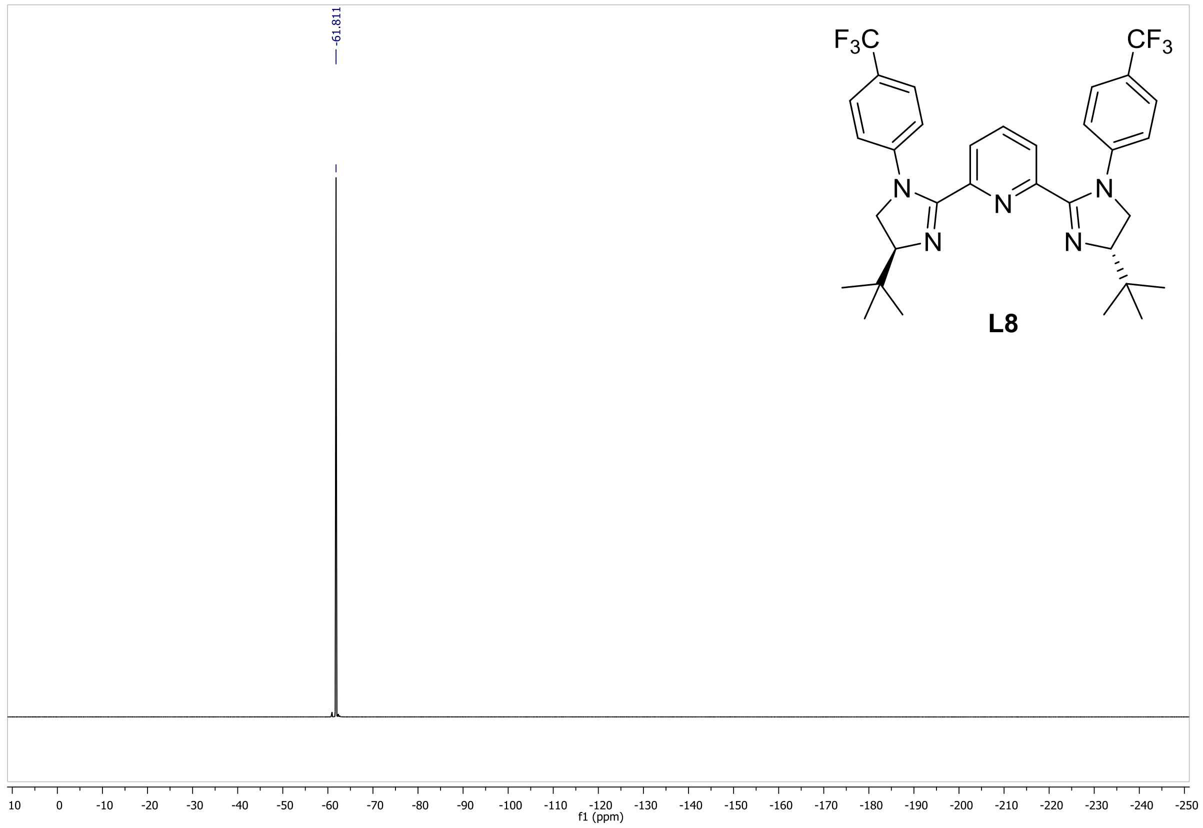

2,6-bis((*S*)-4-(*tert*-butyl)-1-(4-(pentafluorosulfanyl)phenyl)-4,5-dihydro-1*H*-imidazol-2-yl)pyridine (L9):

^{1}H NMR, 600 MHz, $CDCl_3$:

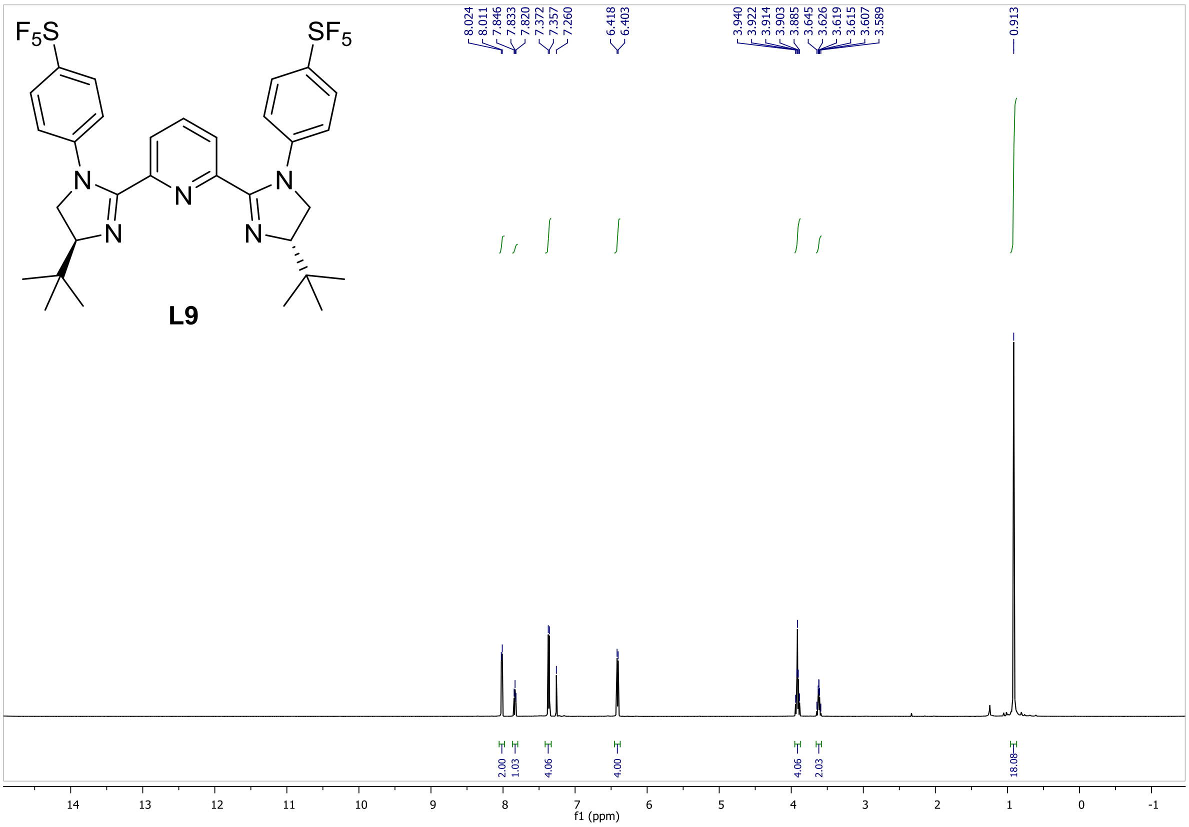

^{13}C NMR, 150 MHz, $CDCl_3$:

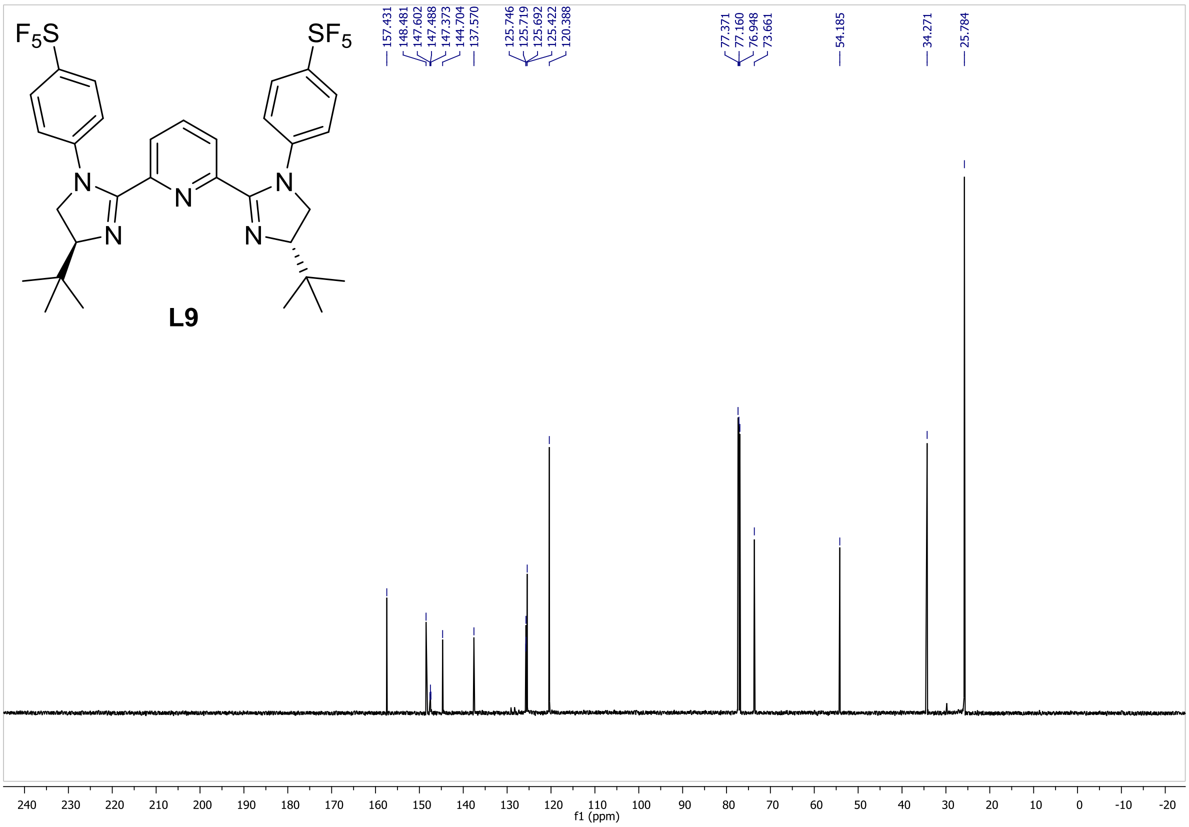

^{19}F NMR, 376 MHz, $CDCl_3$:

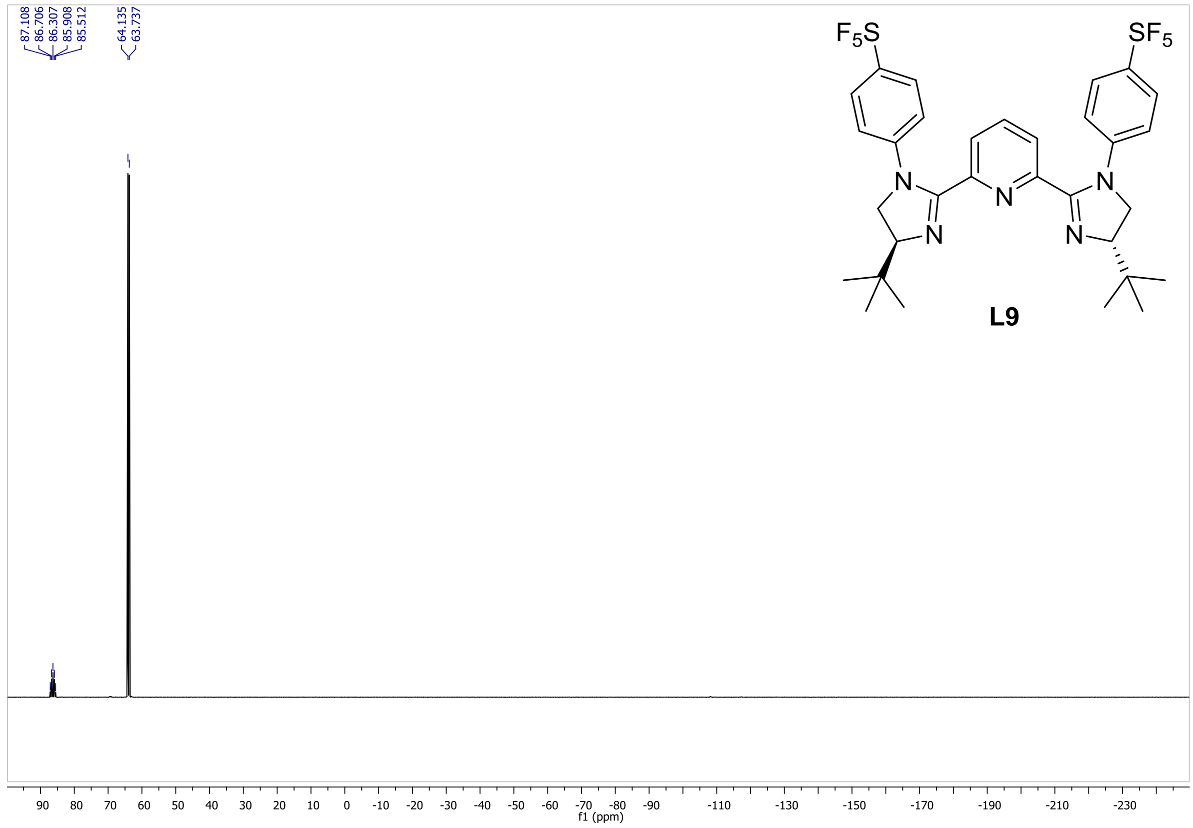

Dimethyl 4,4'-((4*S*,4'*S*)-pyridine-2,6-diylbis(4-(*tert*-butyl)-4,5-dihydro-1*H*-imidazole-2,1-diyl))dibenzoate:

^{1}H NMR, 600 MHz, CDCl$_3$:

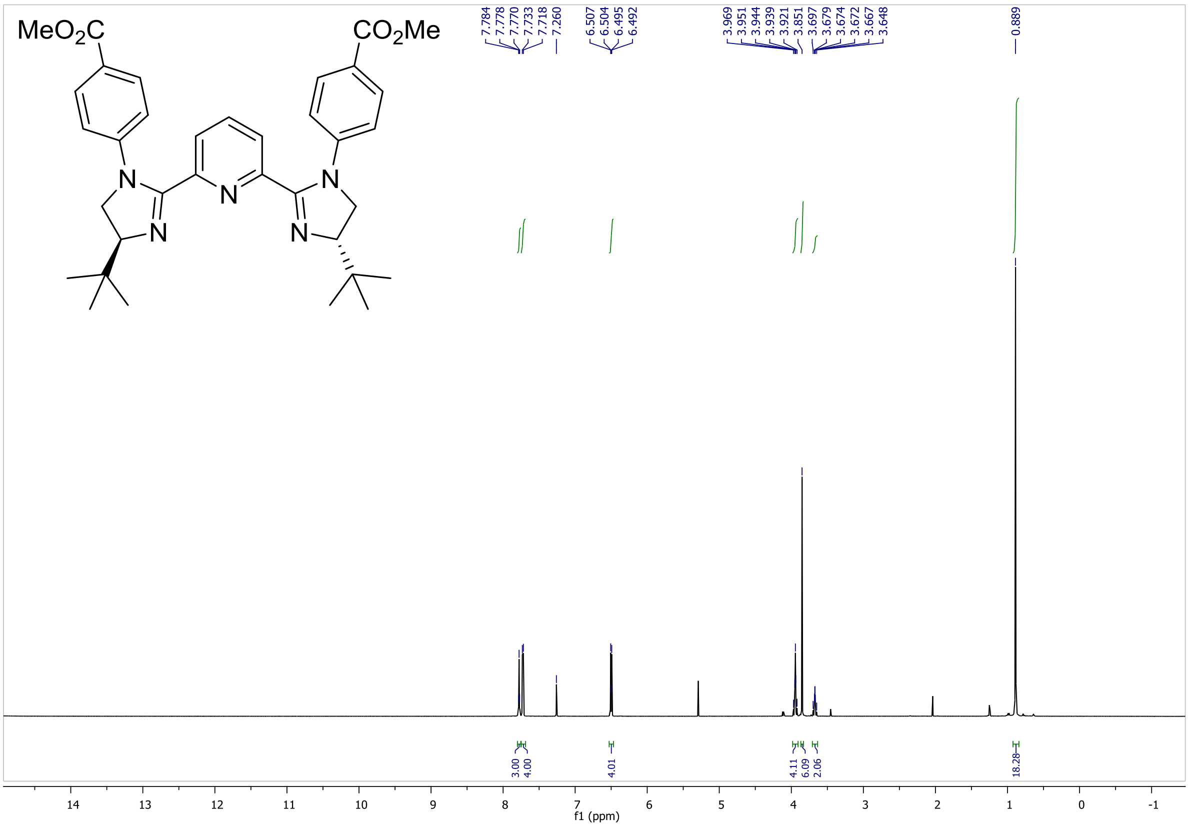

^{13}C NMR, 150 MHz, CDCl$_3$:

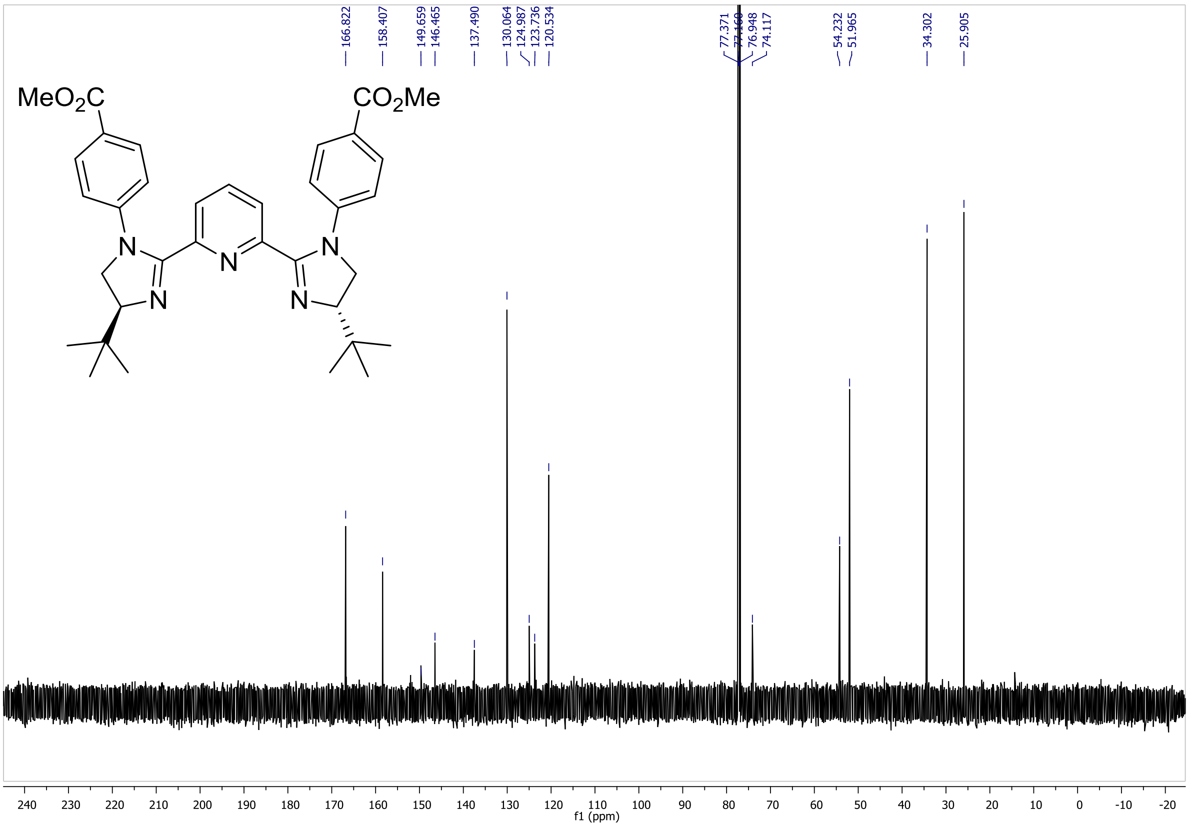

2,6-bis((*S*)-4-(*tert*-butyl)-1-(4-nitrophenyl)-4,5-dihydro-1*H*-imidazol-2-yl)pyridine (L10):

^{1}H NMR, 600 MHz, $CDCl_3$:

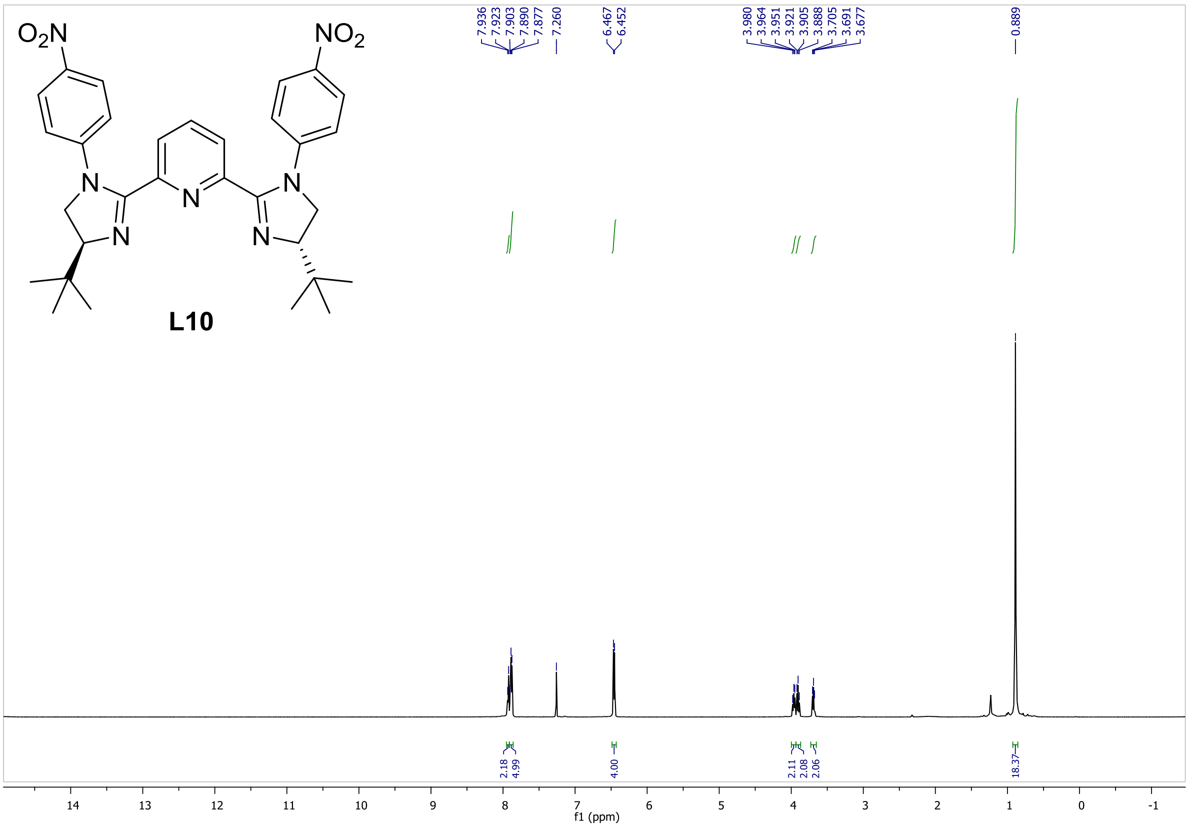

^{13}C NMR, 150 MHz, $CDCl_3$:

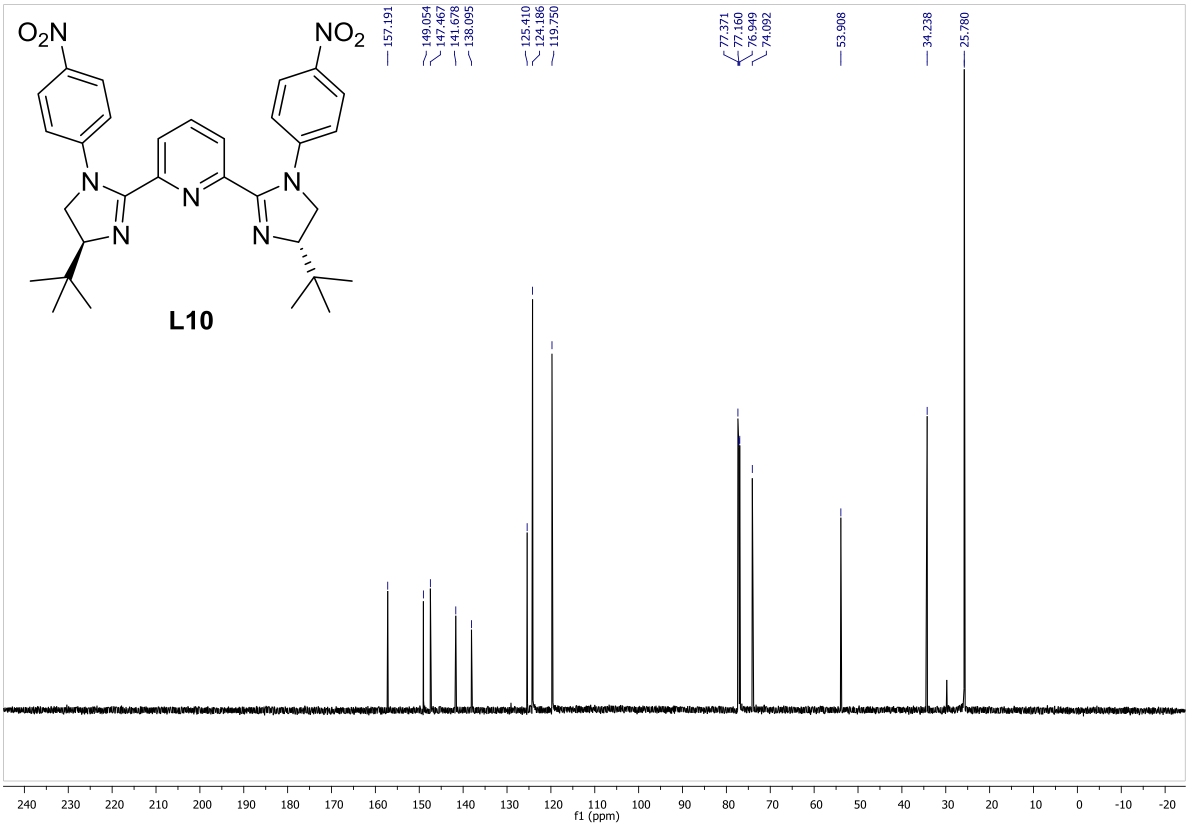

2,6-bis((*S*)-4-(*tert*-butyl)-1-(4-((trifluoromethyl)sulfonyl)phenyl)-4,5-dihydro-1*H*-imidazol-2-yl)pyridine (L11):

^{1}H NMR, 600 MHz, $CDCl_3$:

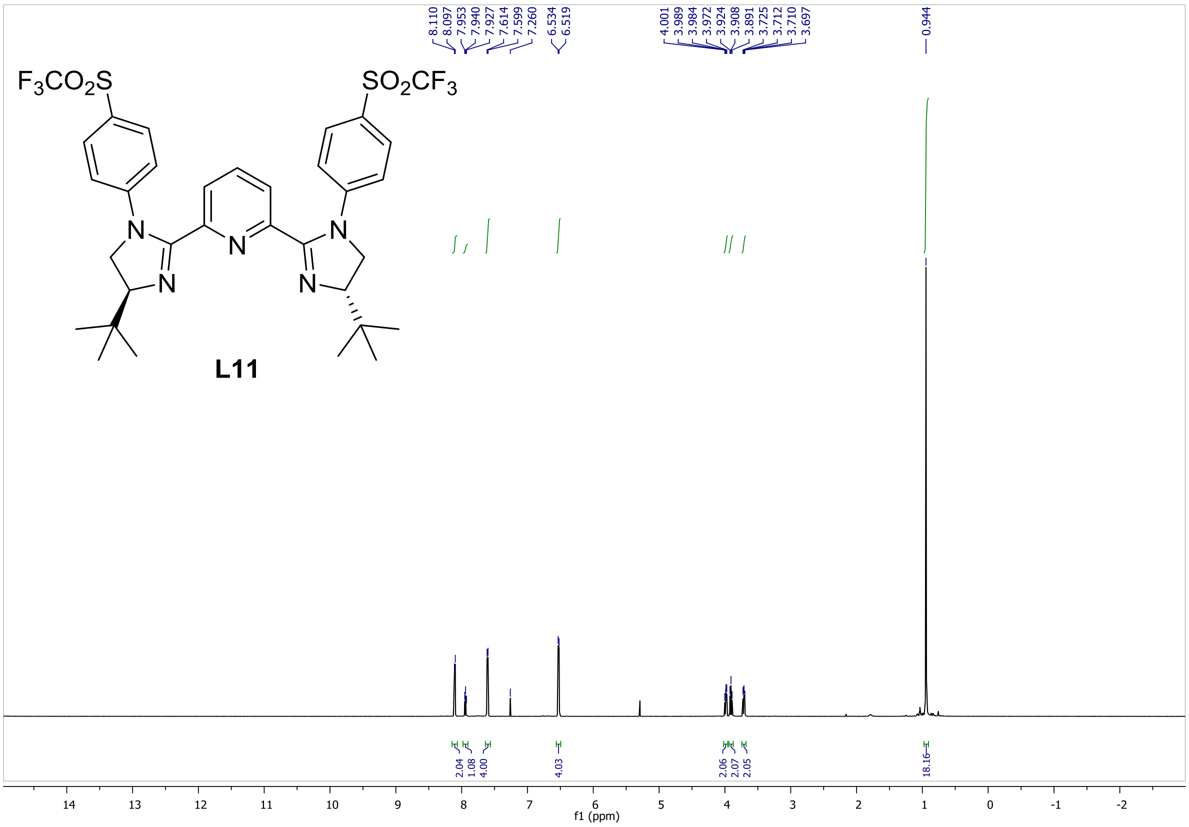

^{13}C NMR, 150 MHz, $CDCl_3$:

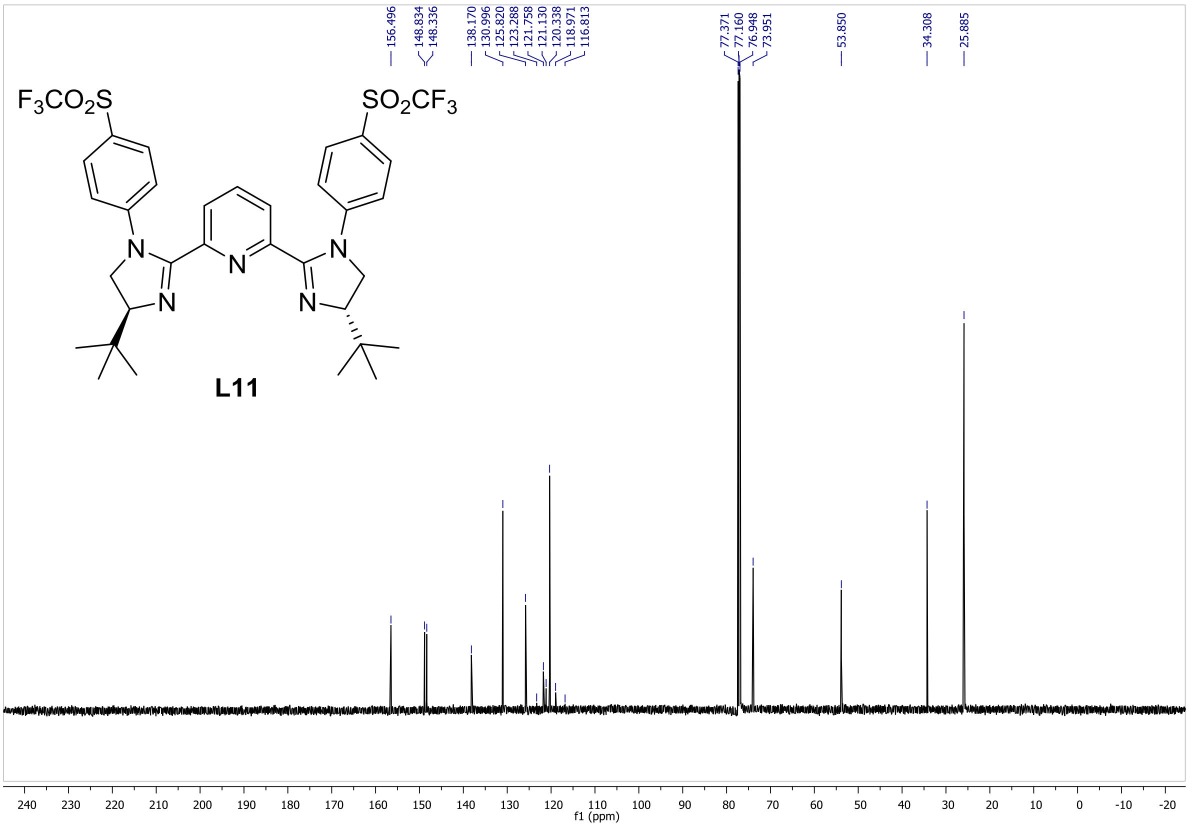

^{19}F NMR, 376 MHz, $CDCl_3$:

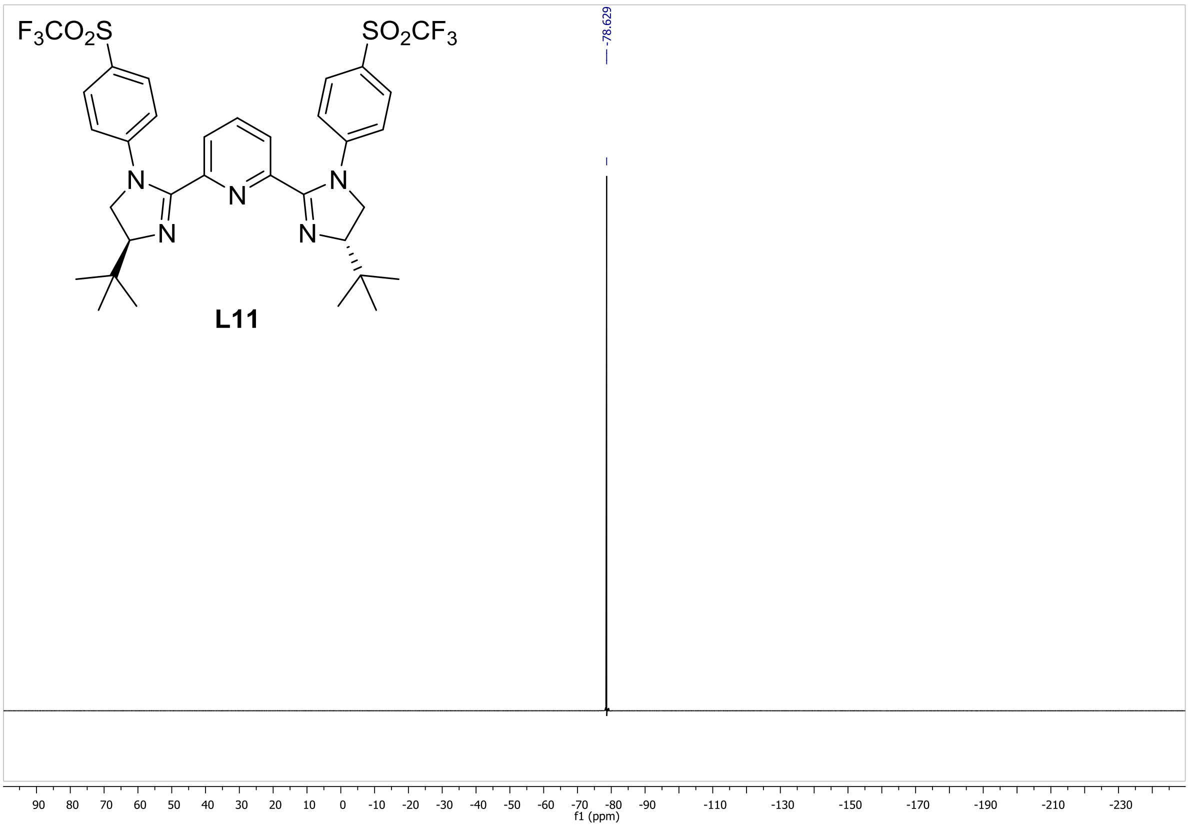

2,6-bis((*S*)-4-(*tert*-butyl)-1-(3,5-difluoro-4-(trifluoromethyl)phenyl)-4,5-dihydro-1*H*-imidazol-2-yl)pyridine:

^{1}H NMR, 600 MHz, CDCl$_3$:

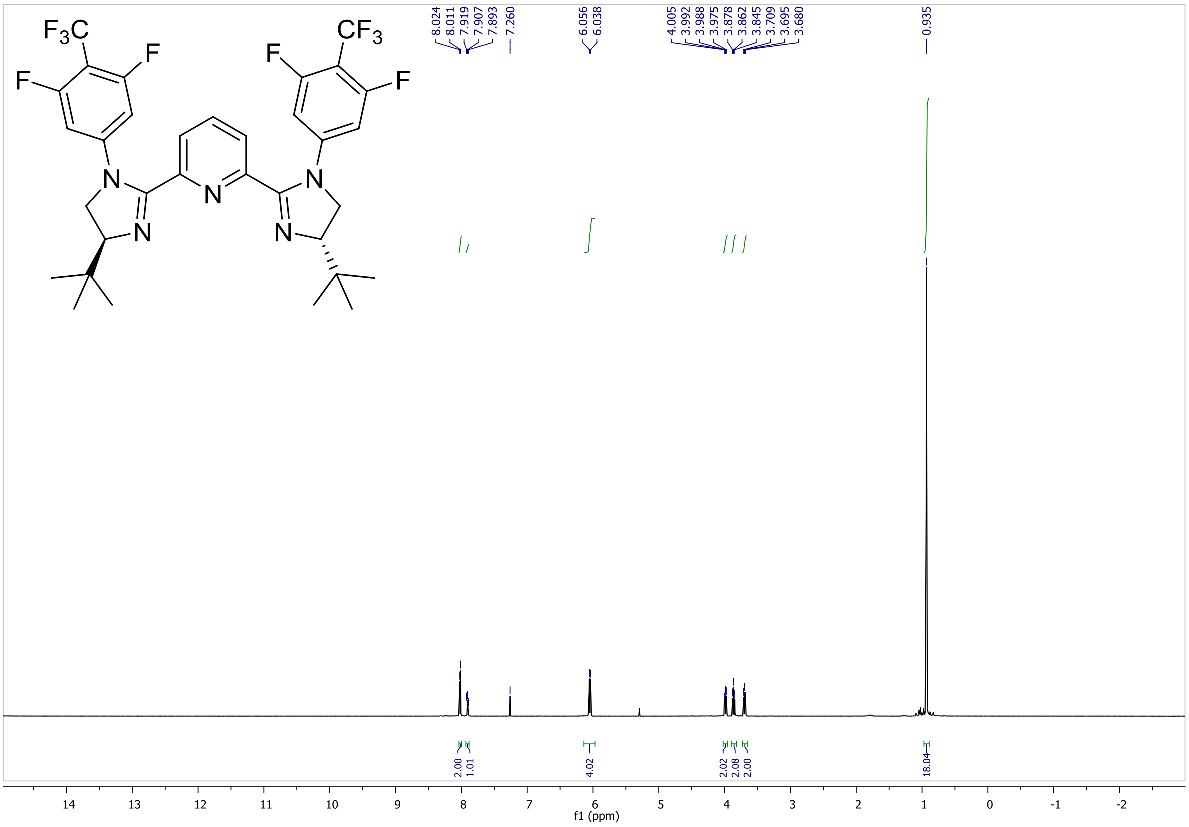

^{13}C NMR, 150 MHz, CDCl$_3$:

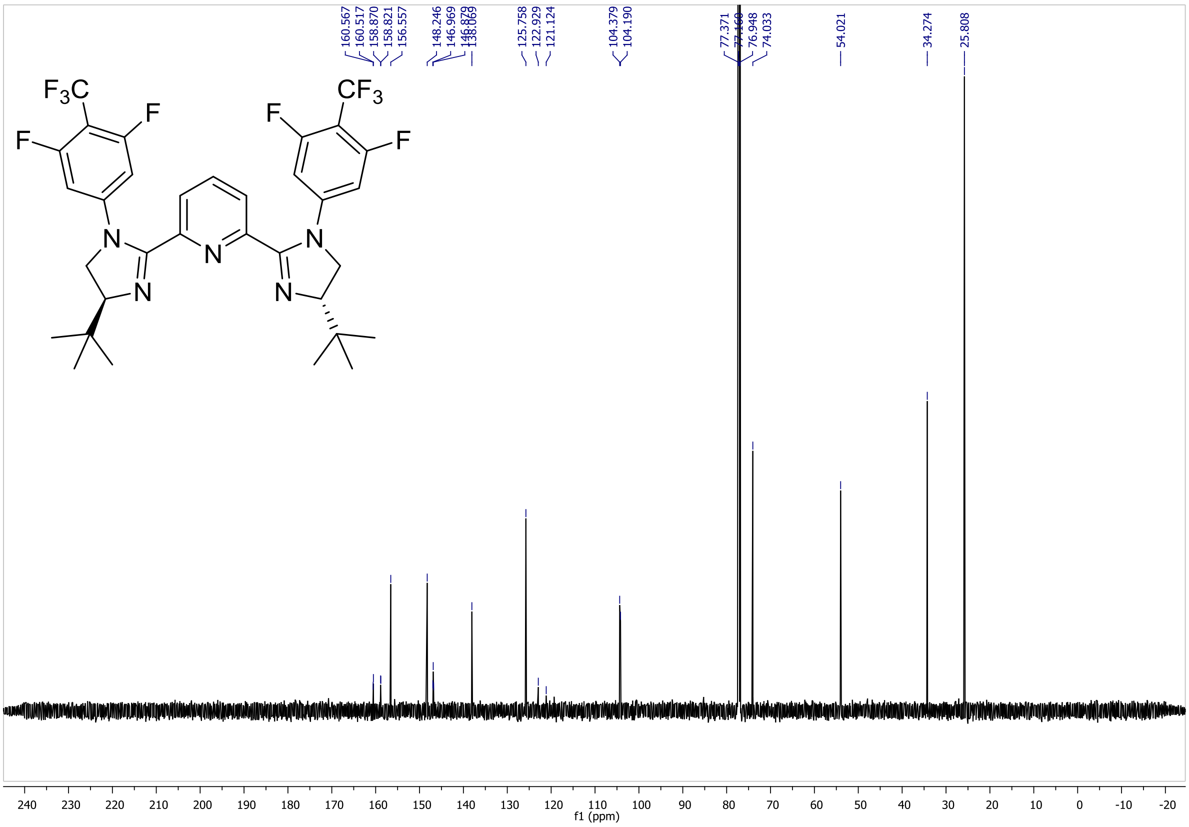

^{19}F NMR, 376 MHz, $CDCl_3$:

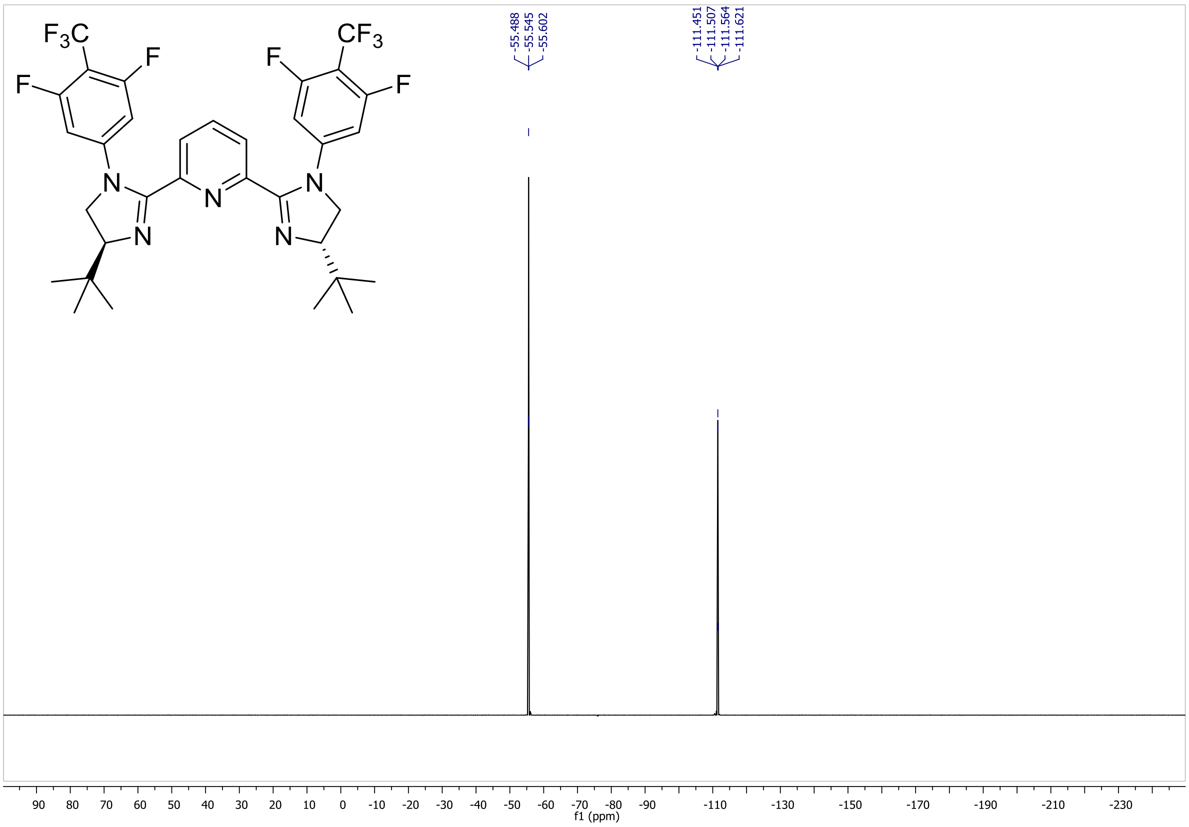

Dimethyl 4-iodopyridine-2,6-dicarboxylate:

^{1}H NMR, 600 MHz, $CDCl_3$:

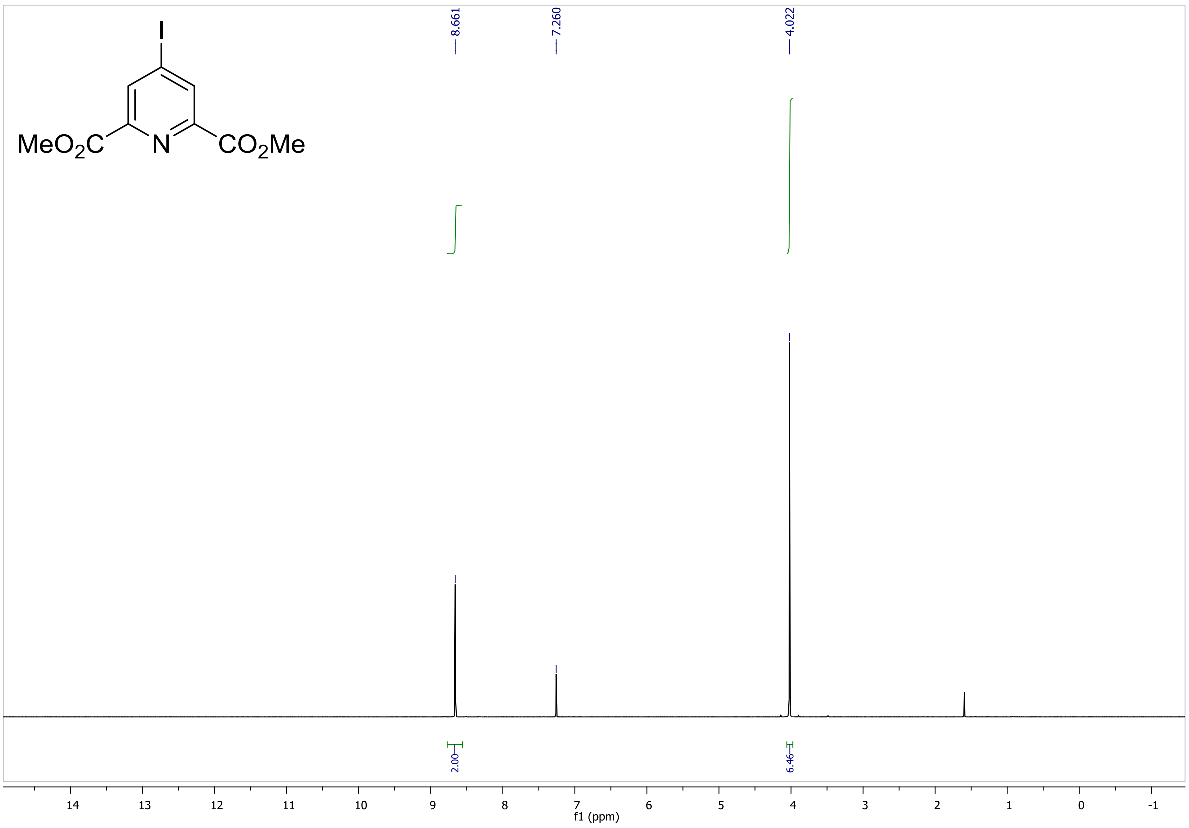

^{13}C NMR, 150 MHz, $CDCl_3$:

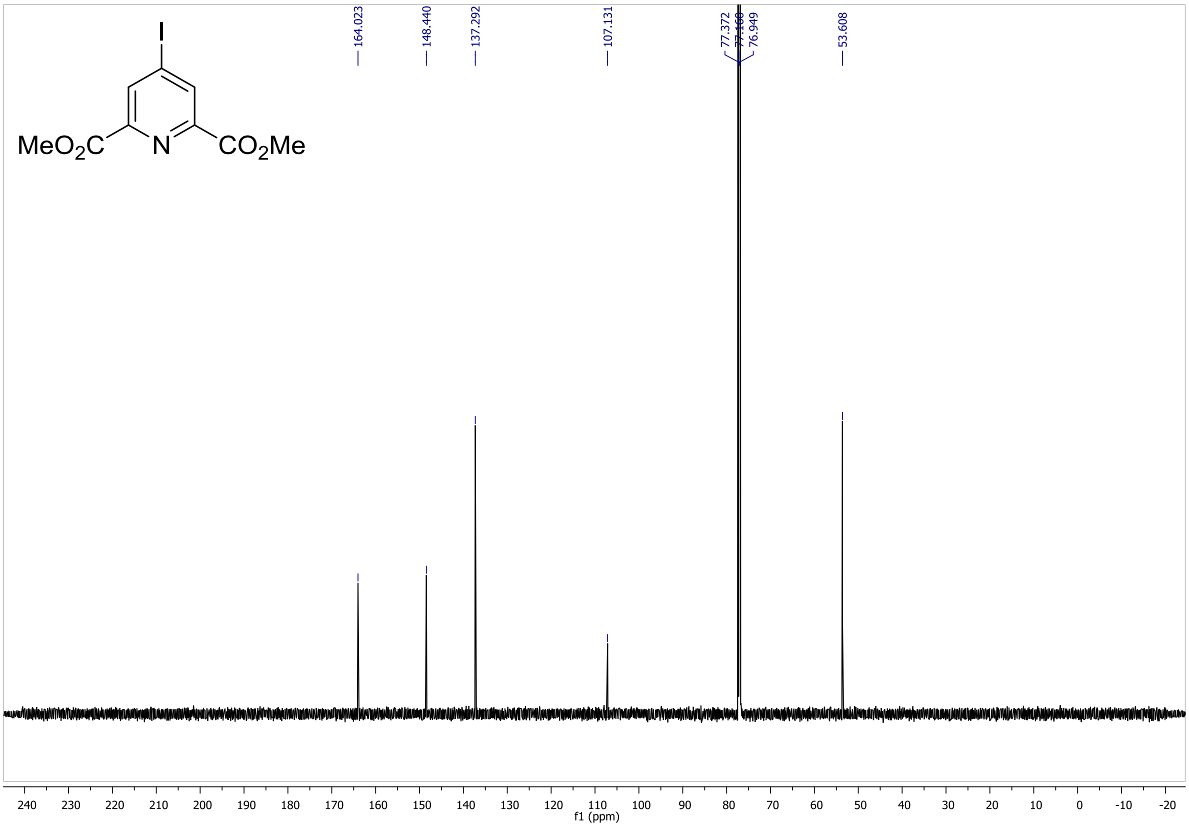

Dimethyl 4-(trifluoromethyl)pyridine-2,6-dicarboxylate:

^{1}H NMR, 600 MHz, $CDCl_3$:

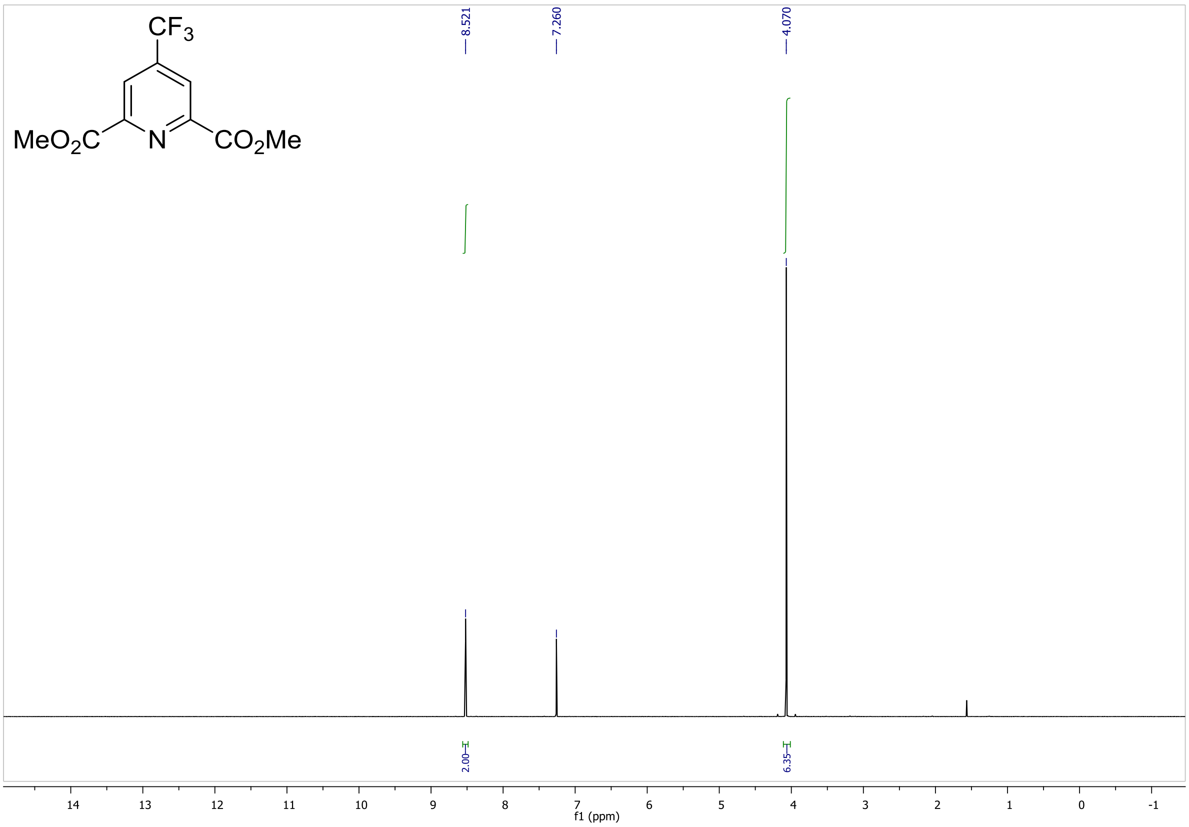

^{13}C NMR, 150 MHz, $CDCl_3$:

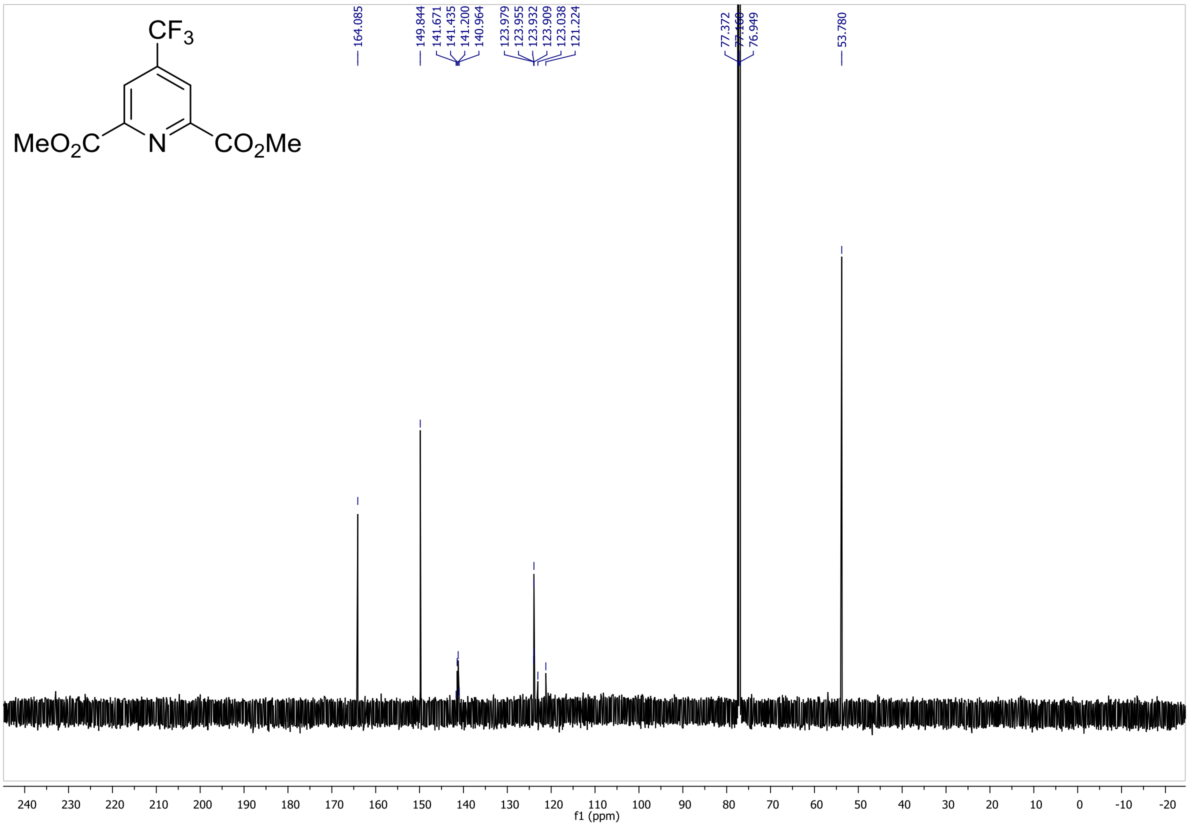

^{19}F NMR, 376 MHz, $CDCl_3$:

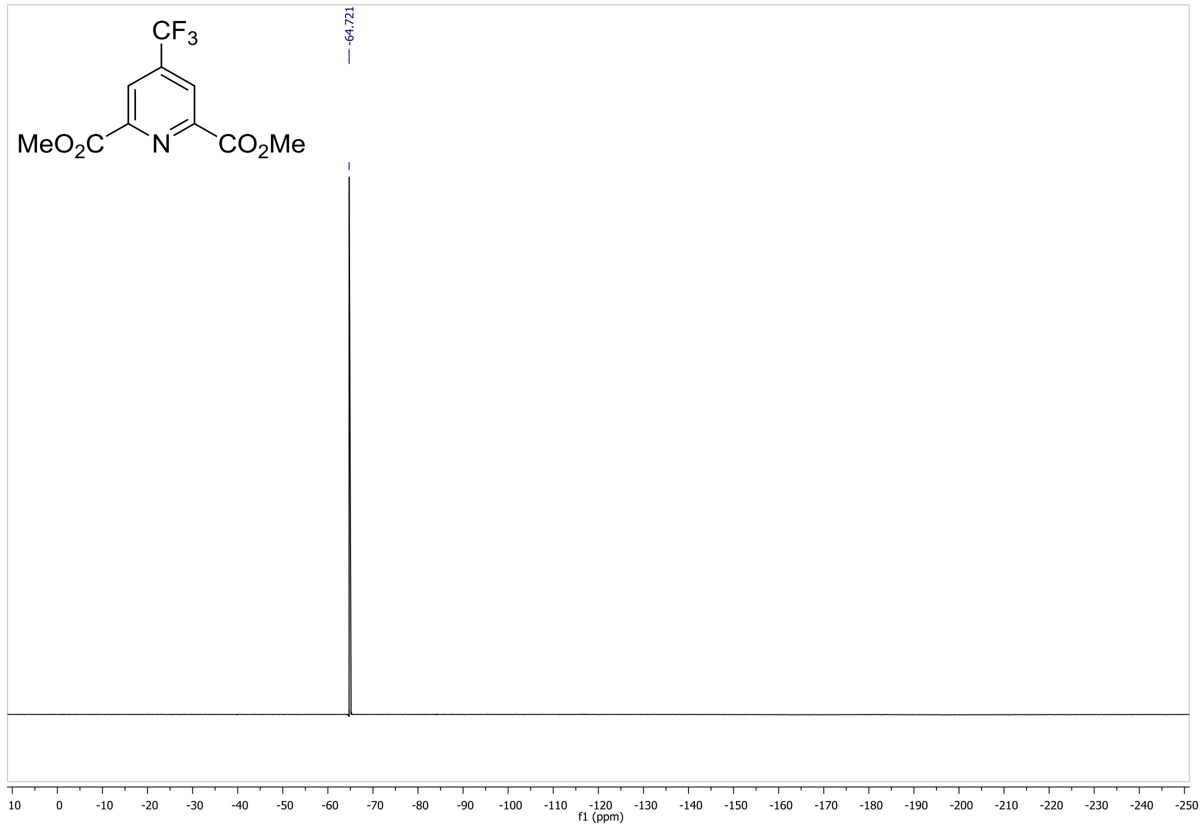

N^2,N^6-bis((*S*)-1-hydroxy-3,3-dimethylbutan-2-yl)-4-(trifluoromethyl)pyridine-2,6-dicarboxamide:

^{1}H NMR, 600 MHz, $CDCl_3$:

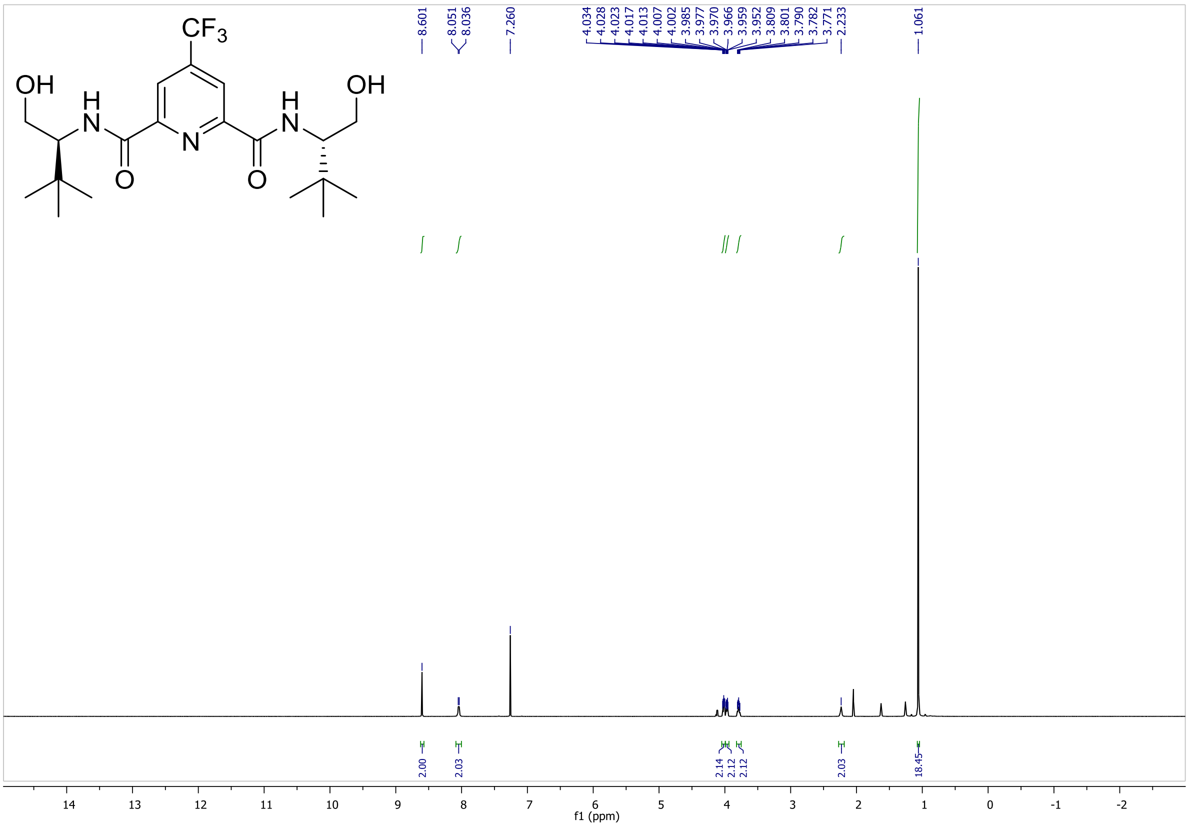

^{13}C NMR, 150 MHz, $CDCl_3$:

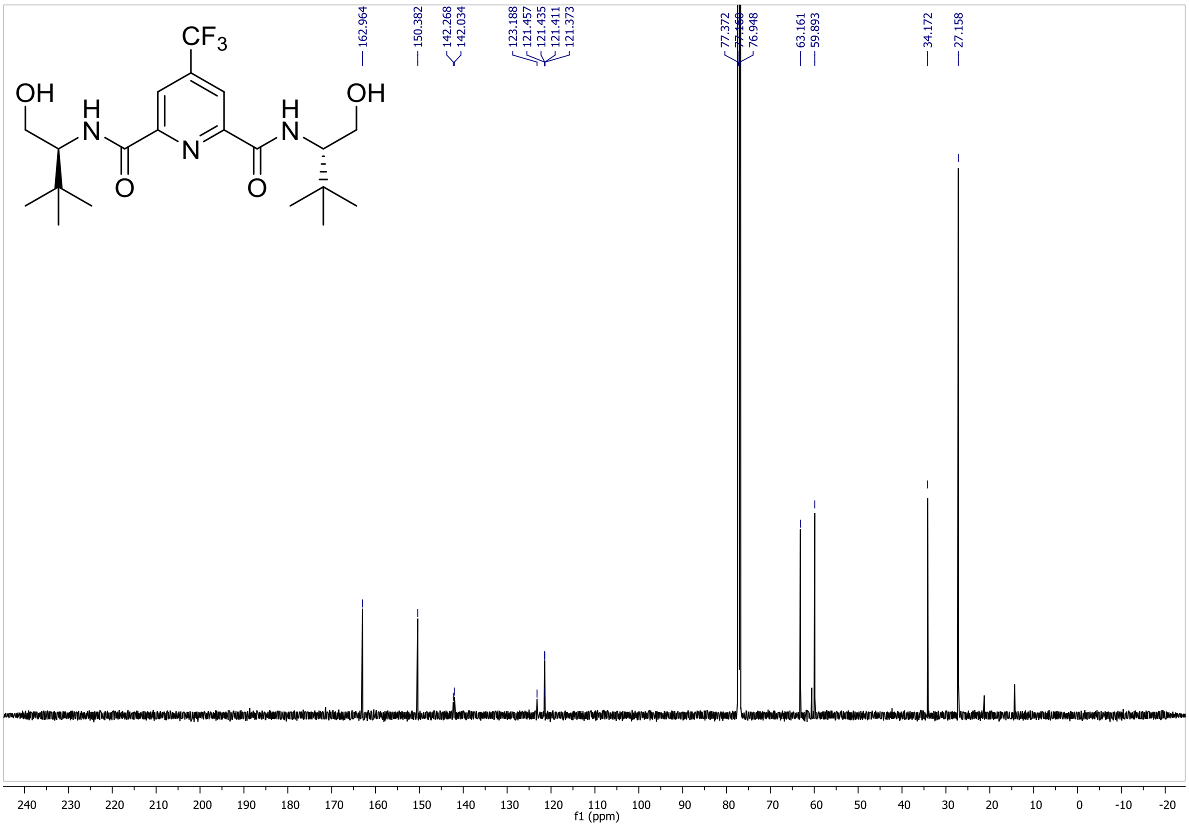

^{19}F NMR, 376 MHz, $CDCl_3$:

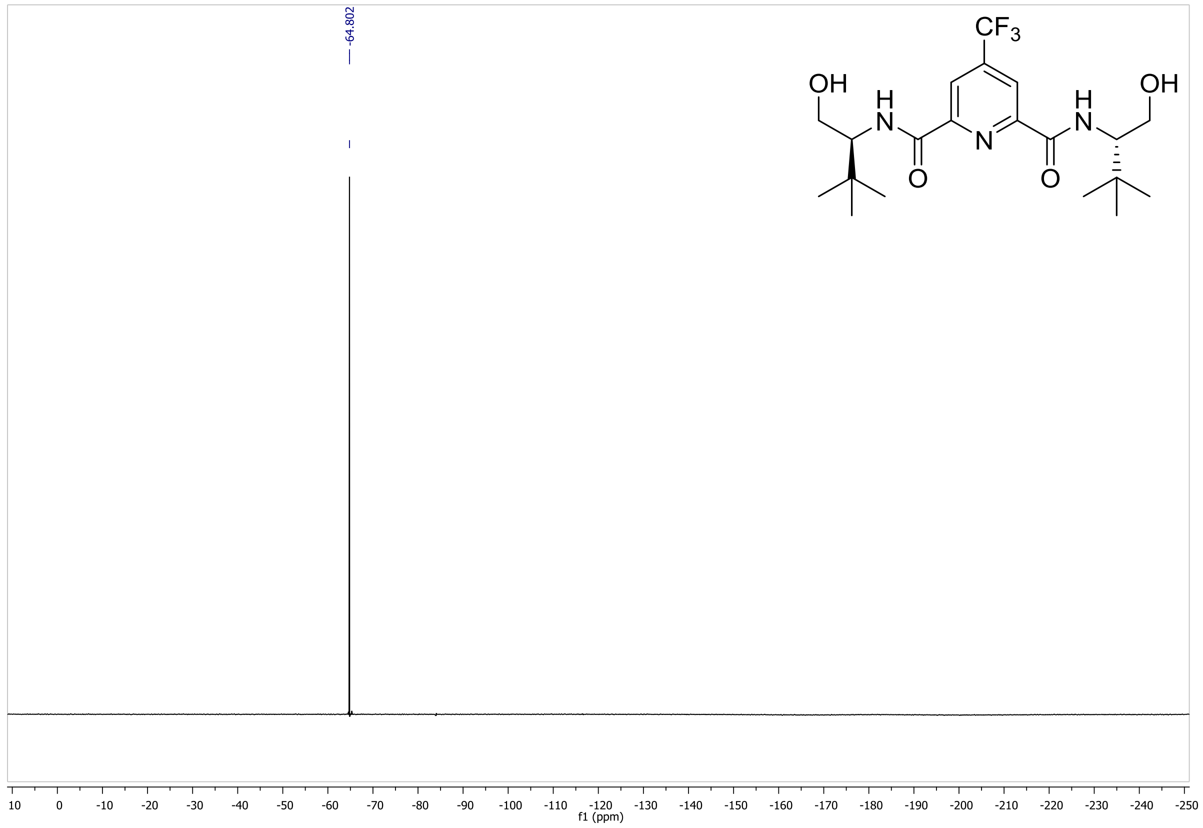

2,6-bis((*S*)-4-(*tert*-butyl)-1-(4-(pentafluorosulfanyl)phenyl)-4,5-dihydro-1*H*-imidazol-2-yl)-4-(trifluoromethyl)pyridine:

^{1}H NMR, 600 MHz, $CDCl_3$:

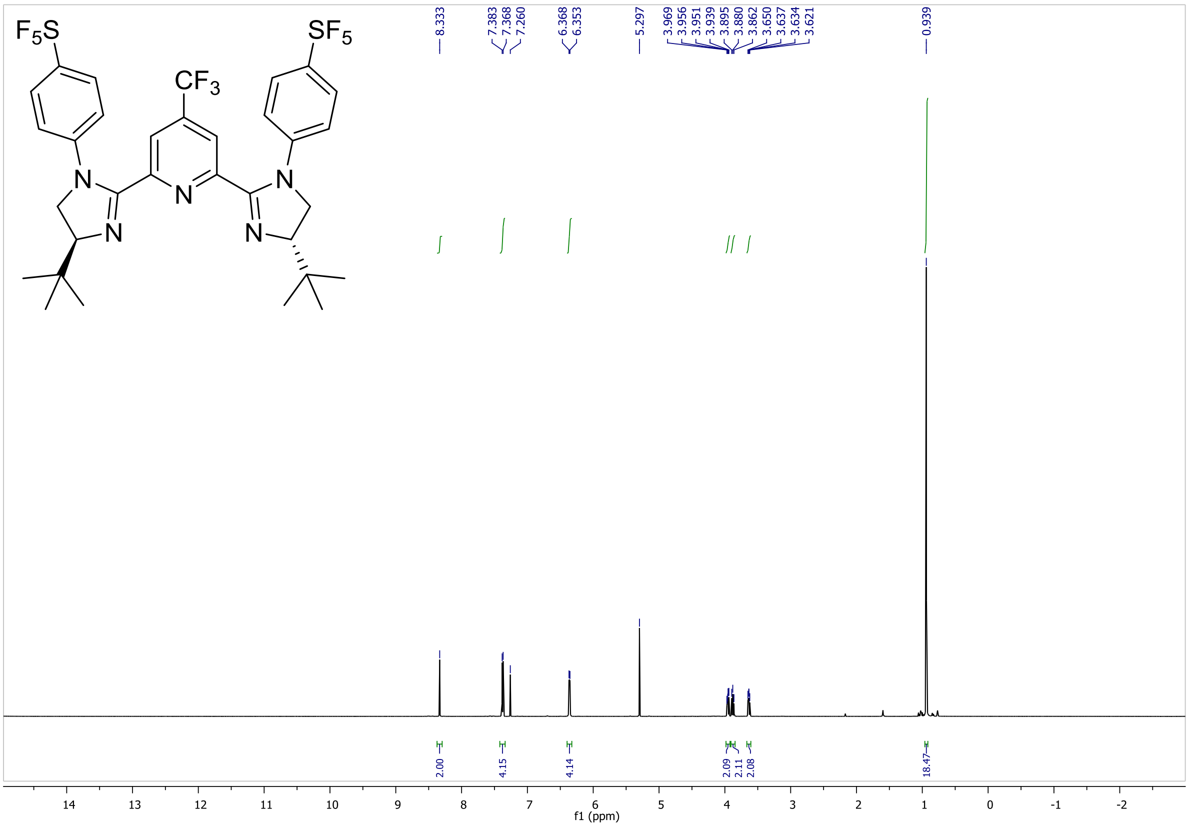

^{13}C NMR, 150 MHz, $CDCl_3$:

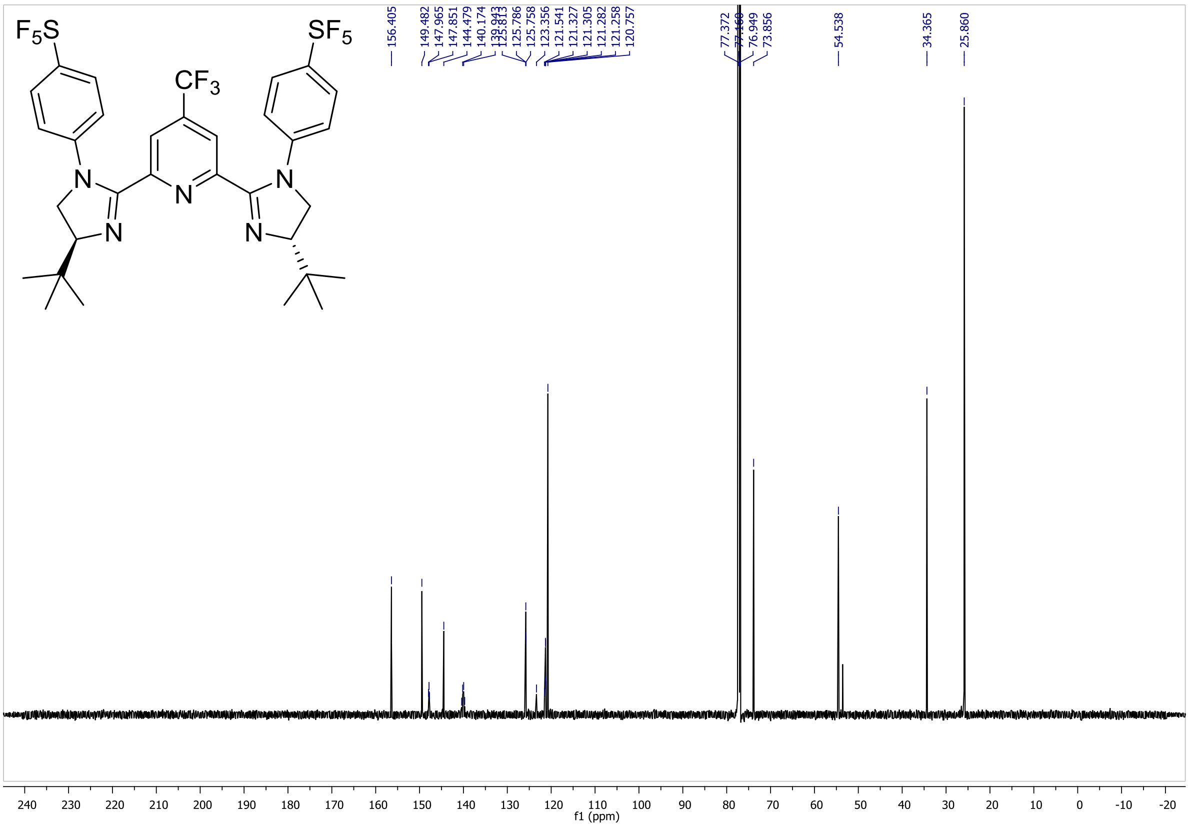

^{19}F NMR, 376 MHz, CDCl$_3$:

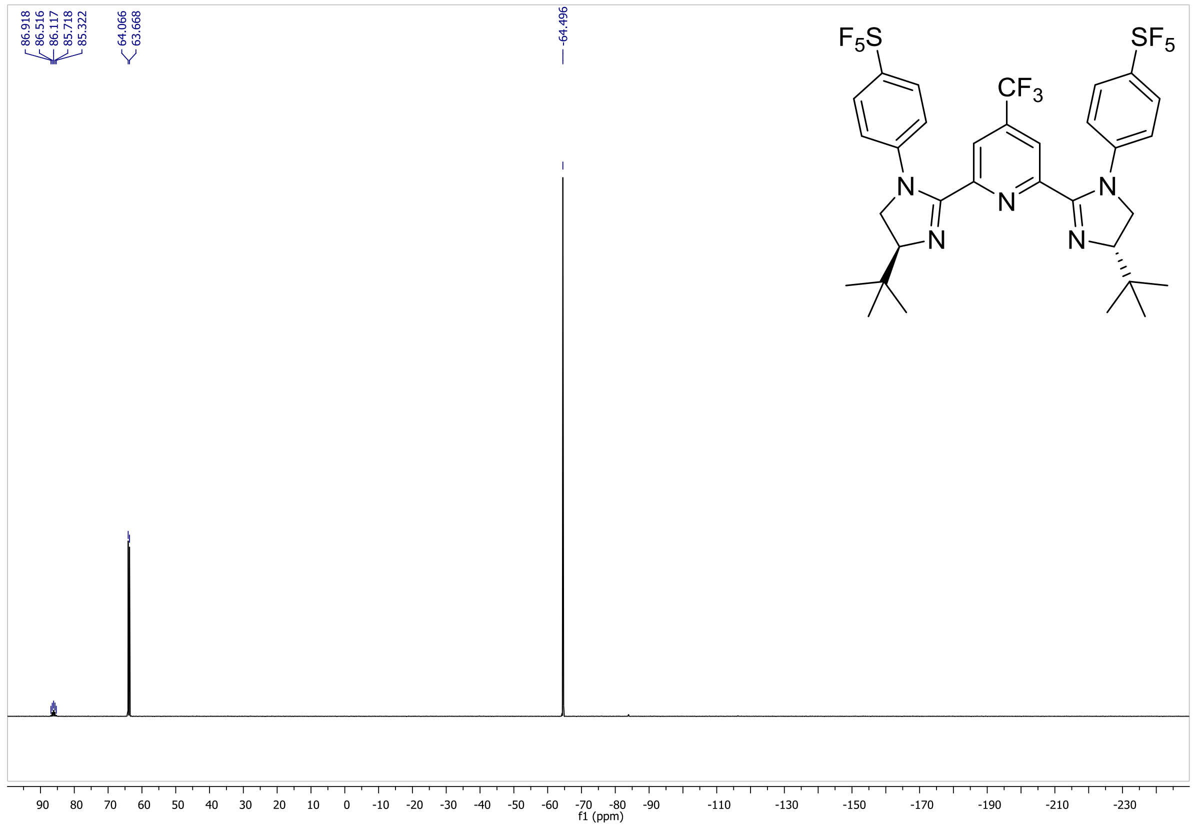

1-adamantanecarboxaldehyde:

^{1}H NMR, 600 MHz, $CDCl_3$:

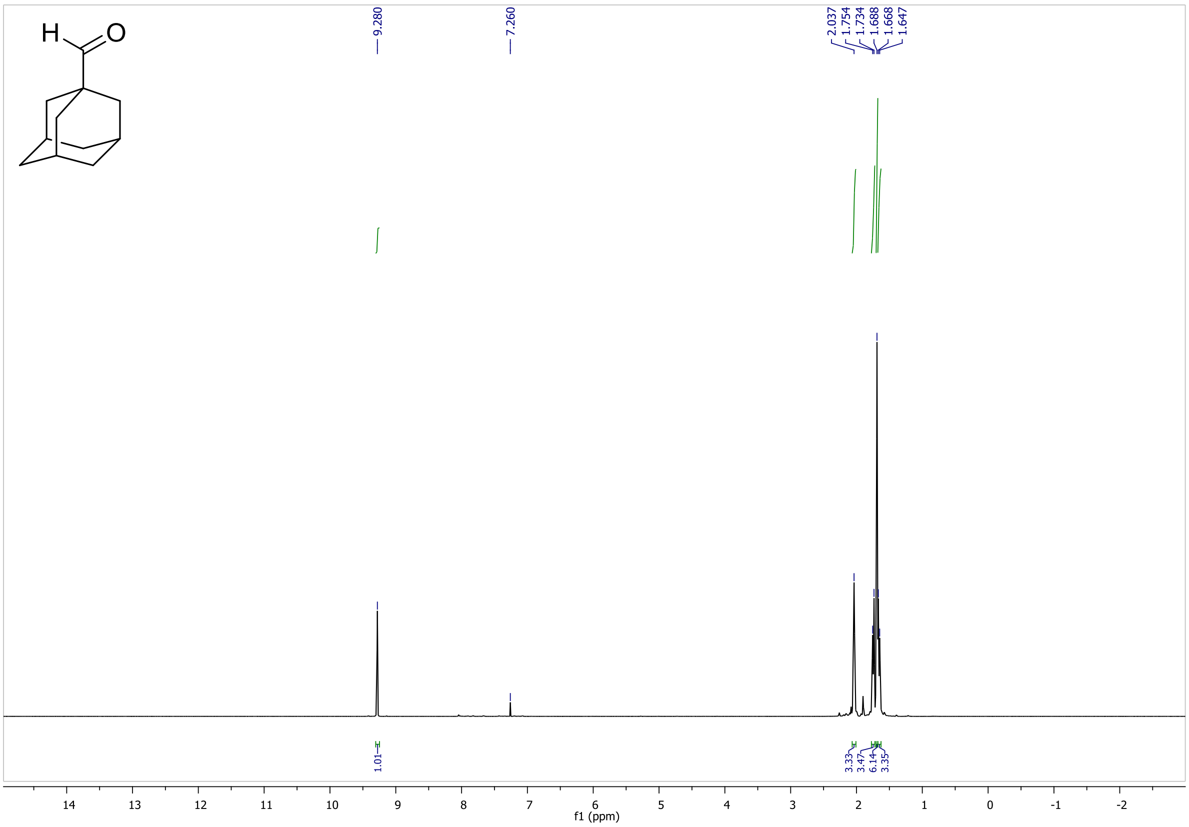

^{13}C NMR, 150 MHz, $CDCl_3$:

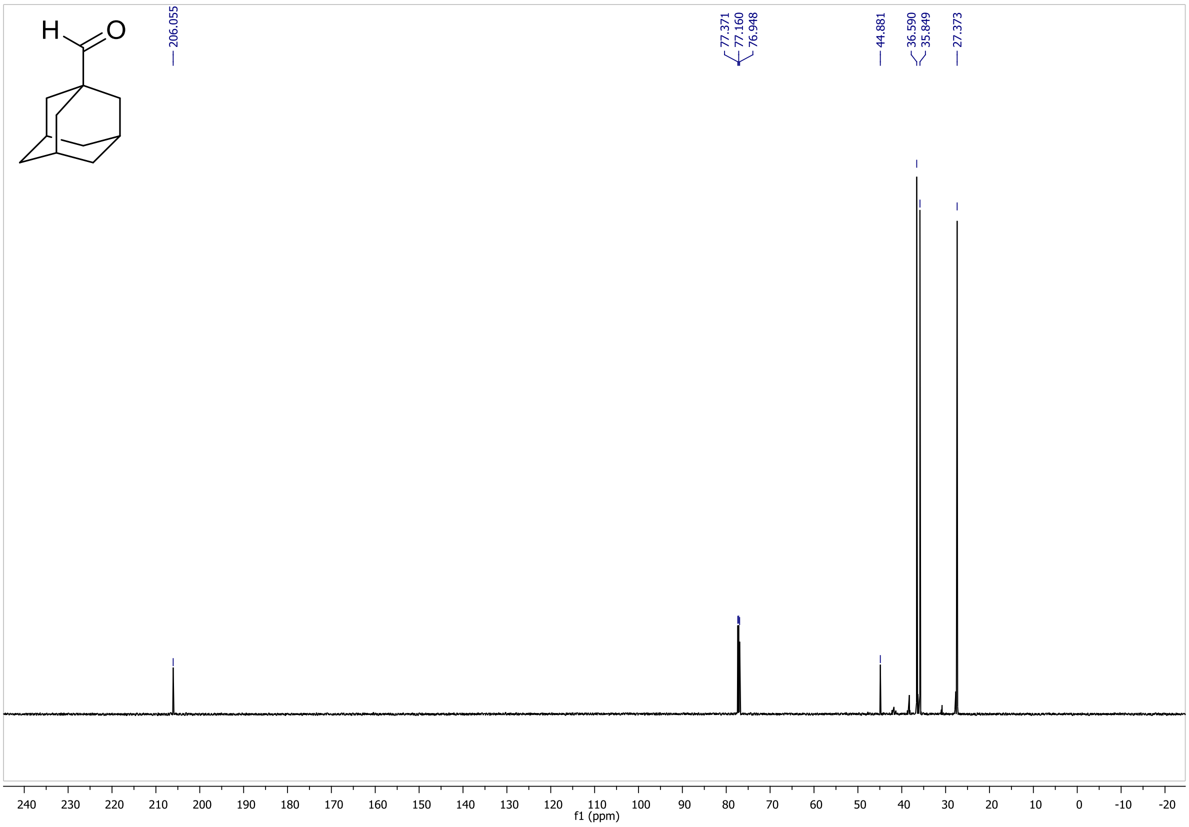

(*S*)-2-(adamantan-1-yl)-2-(((*R*)-2-hydroxy-1-phenylethyl)amino)acetonitrile:

^{1}H NMR, 600 MHz, $CDCl_3$:

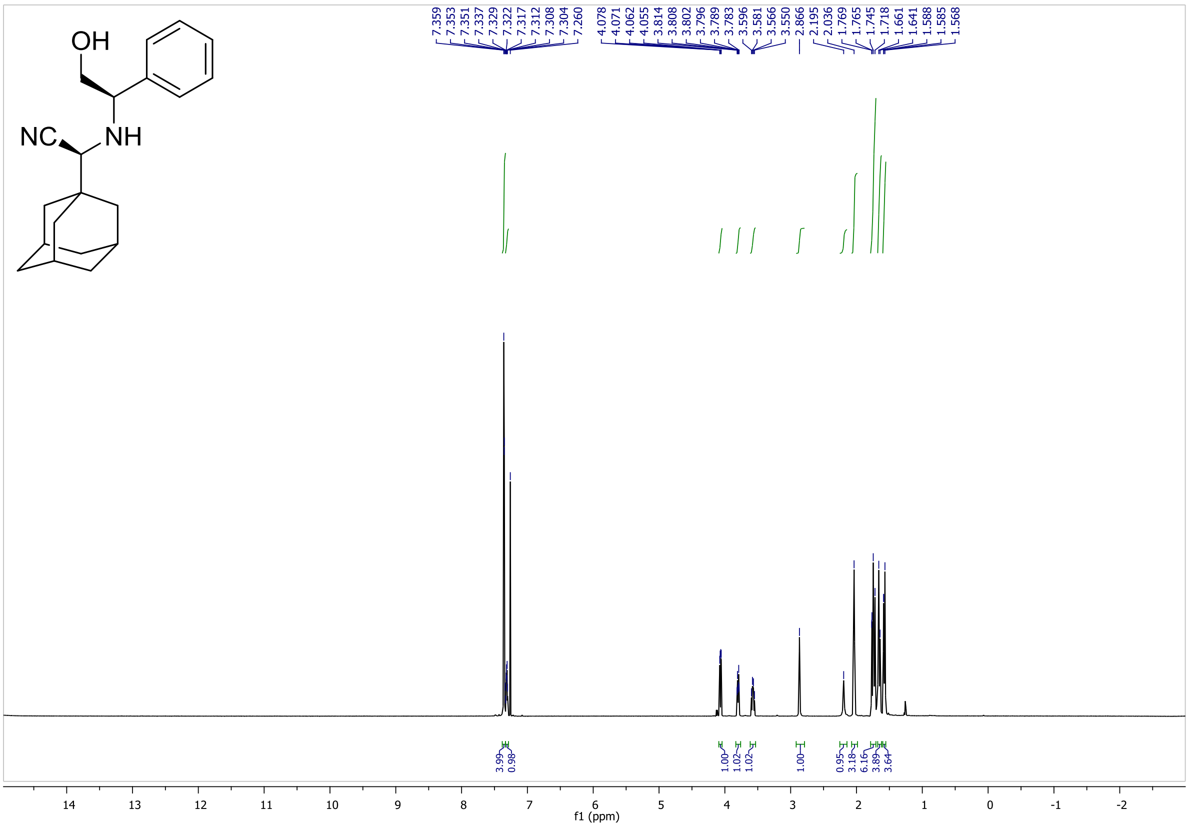

^{13}C NMR, 150 MHz, $CDCl_3$:

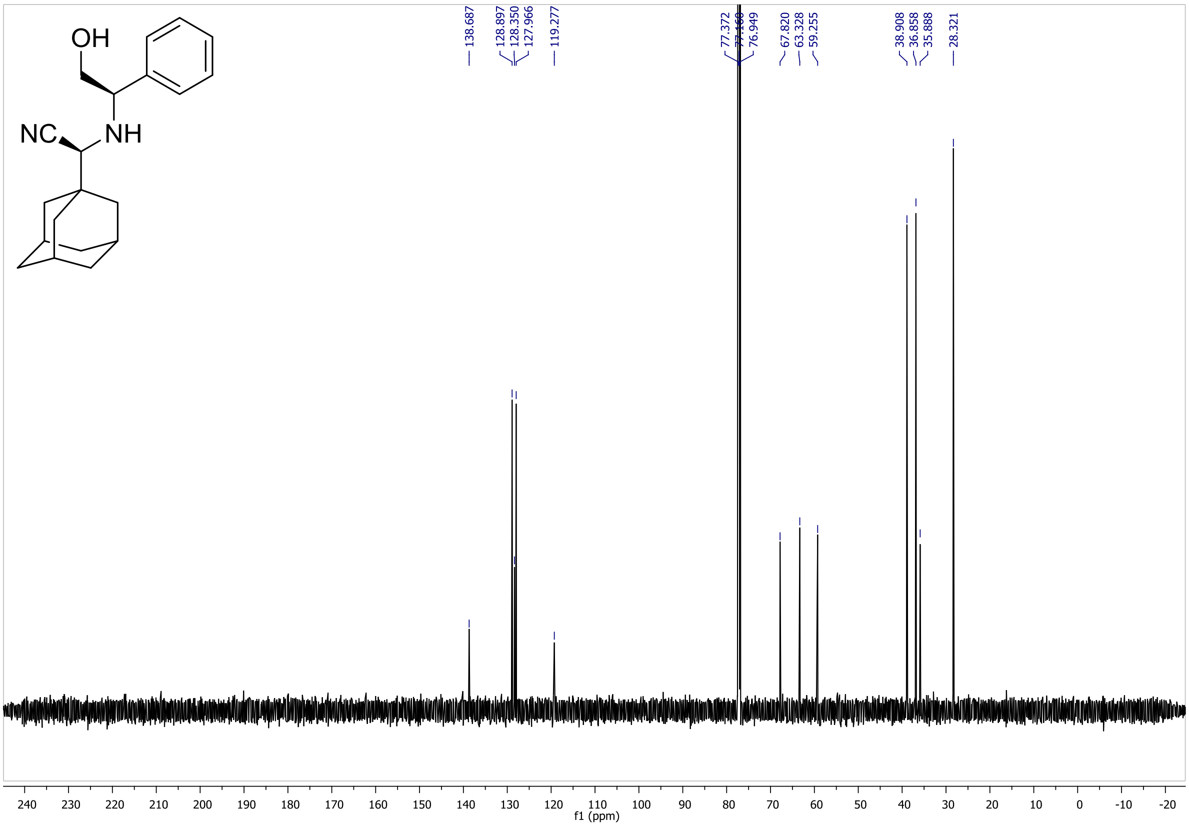

(*R*)-*N*-((*S*)-(adamantan-1-yl)(carboxy)methyl)-2-hydroxy-1-phenylethan-1-ammonium chloride:

^{1}H NMR, 600 MHz, d_4-MeOD:

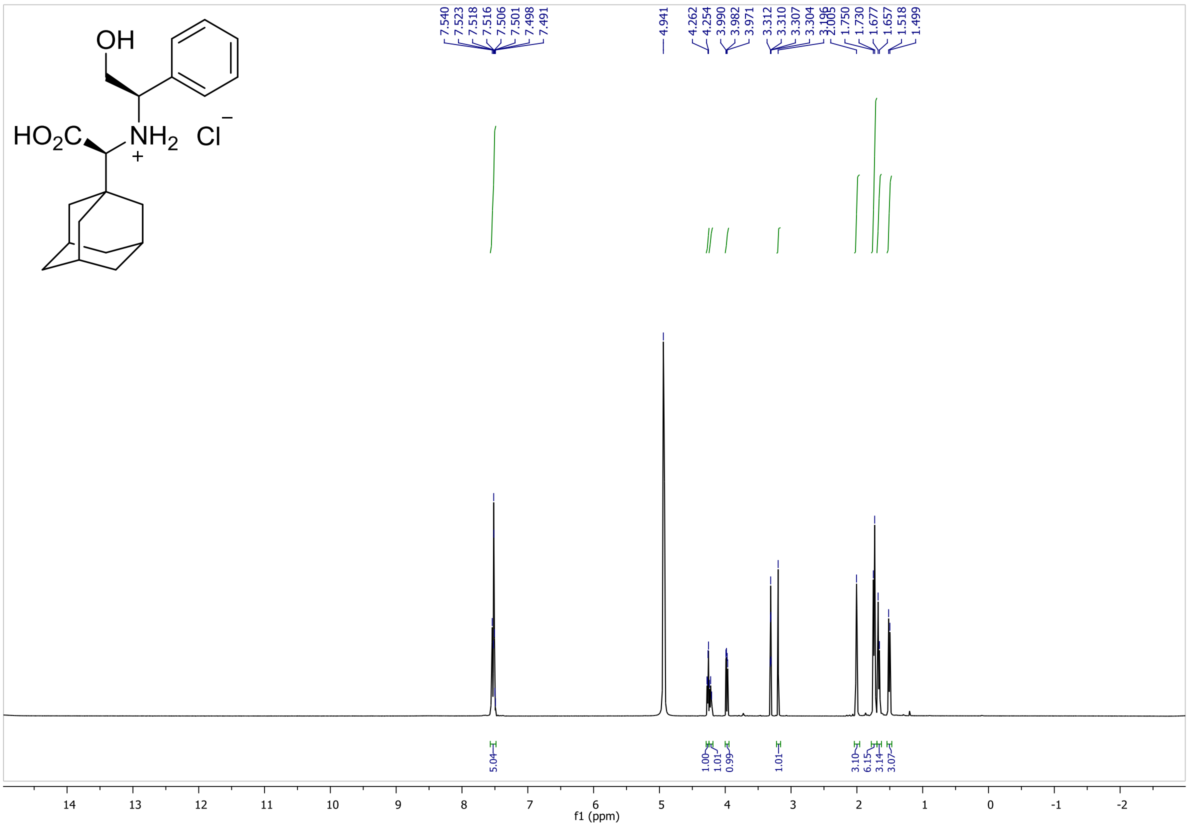

^{13}C NMR, 150 MHz, d_4-MeOD:

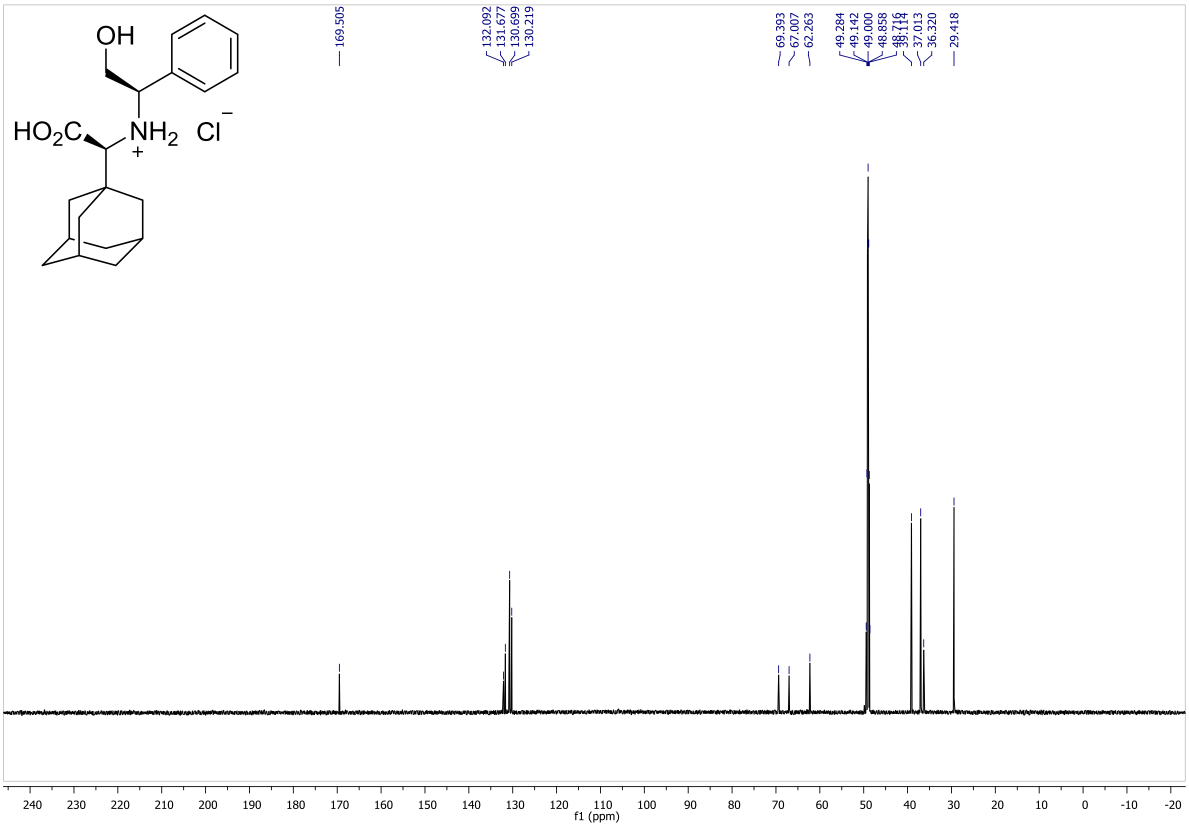

(*S*)-(adamantan-1-yl)(carboxy)methanammonium chloride:

^{1}H NMR, 600 MHz, d$_4$-MeOD:

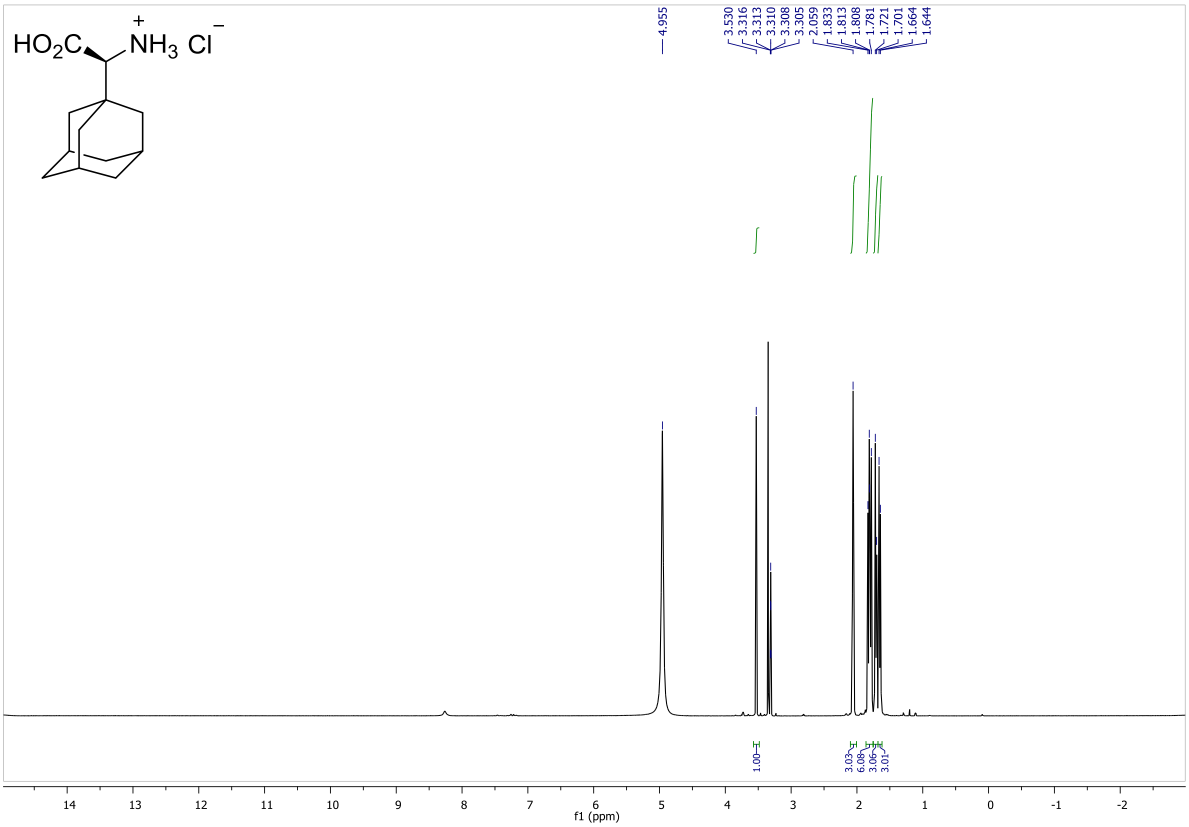

^{13}C NMR, 150 MHz, d$_4$-MeOD:

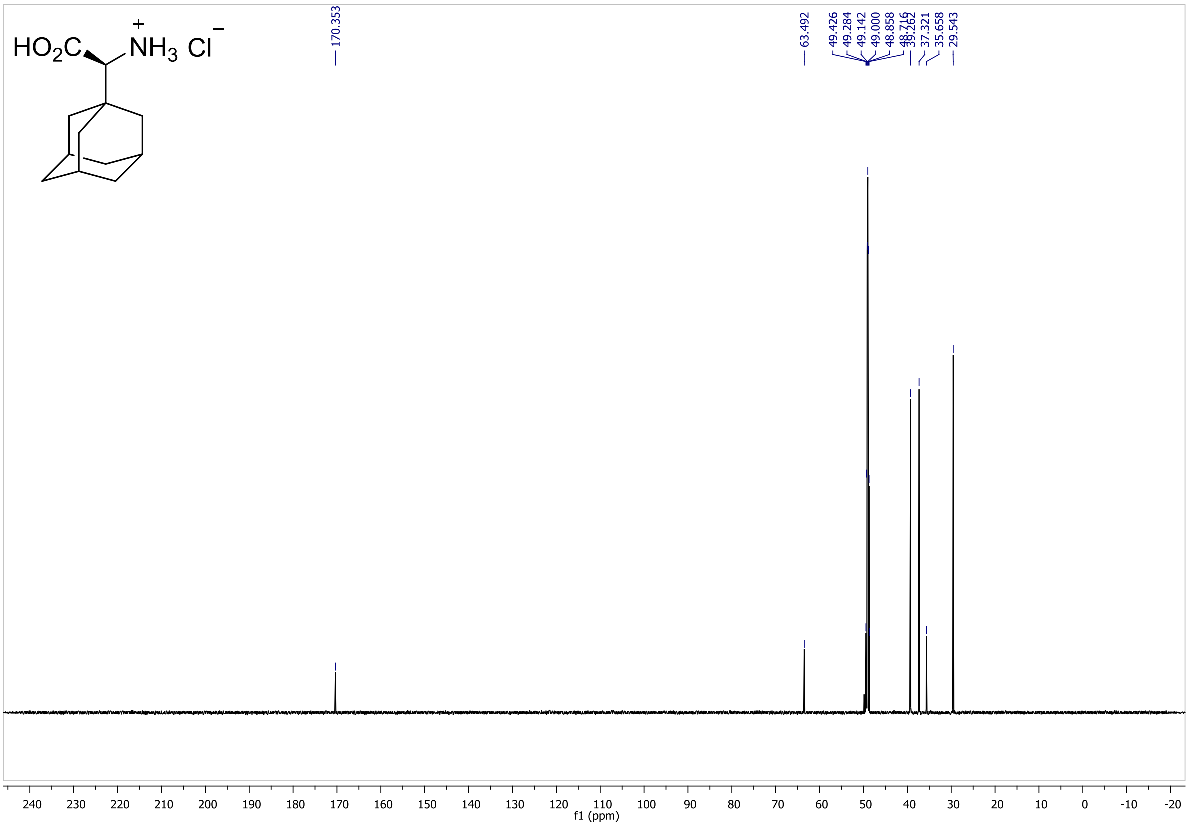

(*S*)-2-(adamantan-1-yl)-2-aminoethan-1-ol:

^{1}H NMR, 600 MHz, $CDCl_3$:

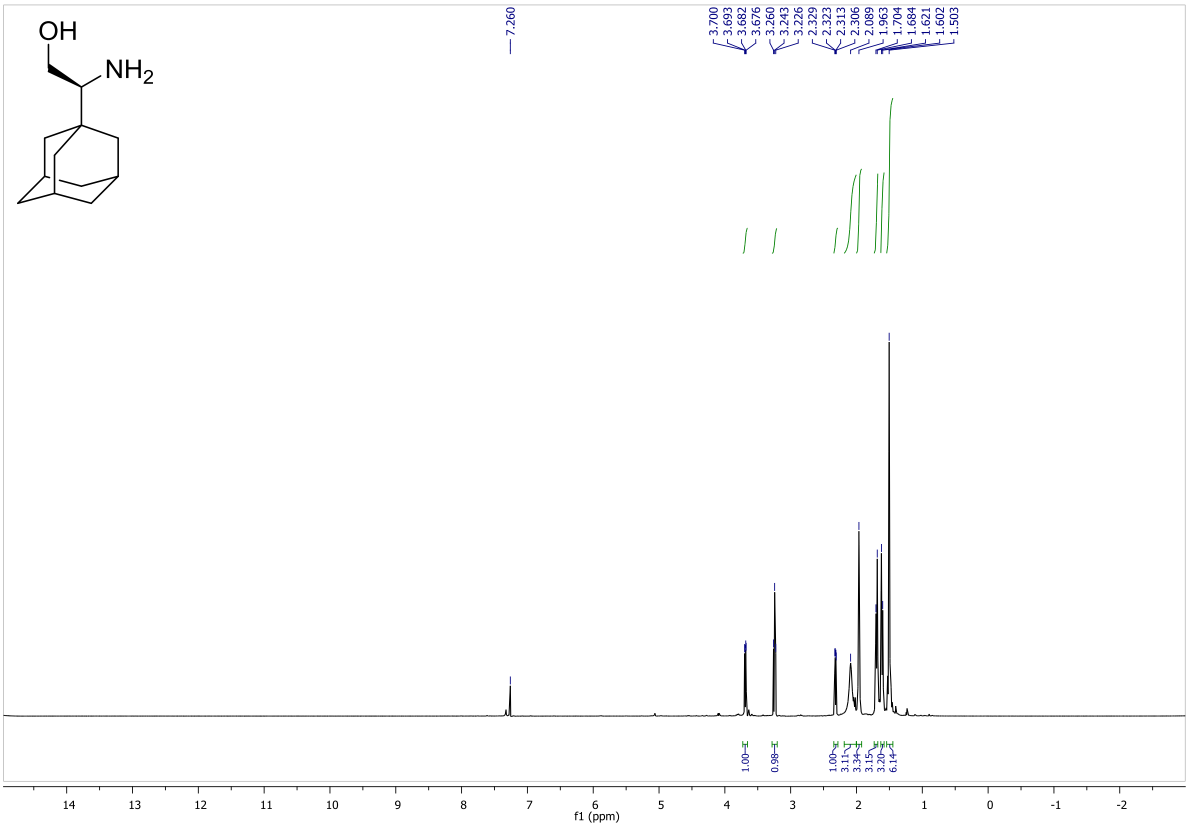

^{13}C NMR, 150 MHz, $CDCl_3$:

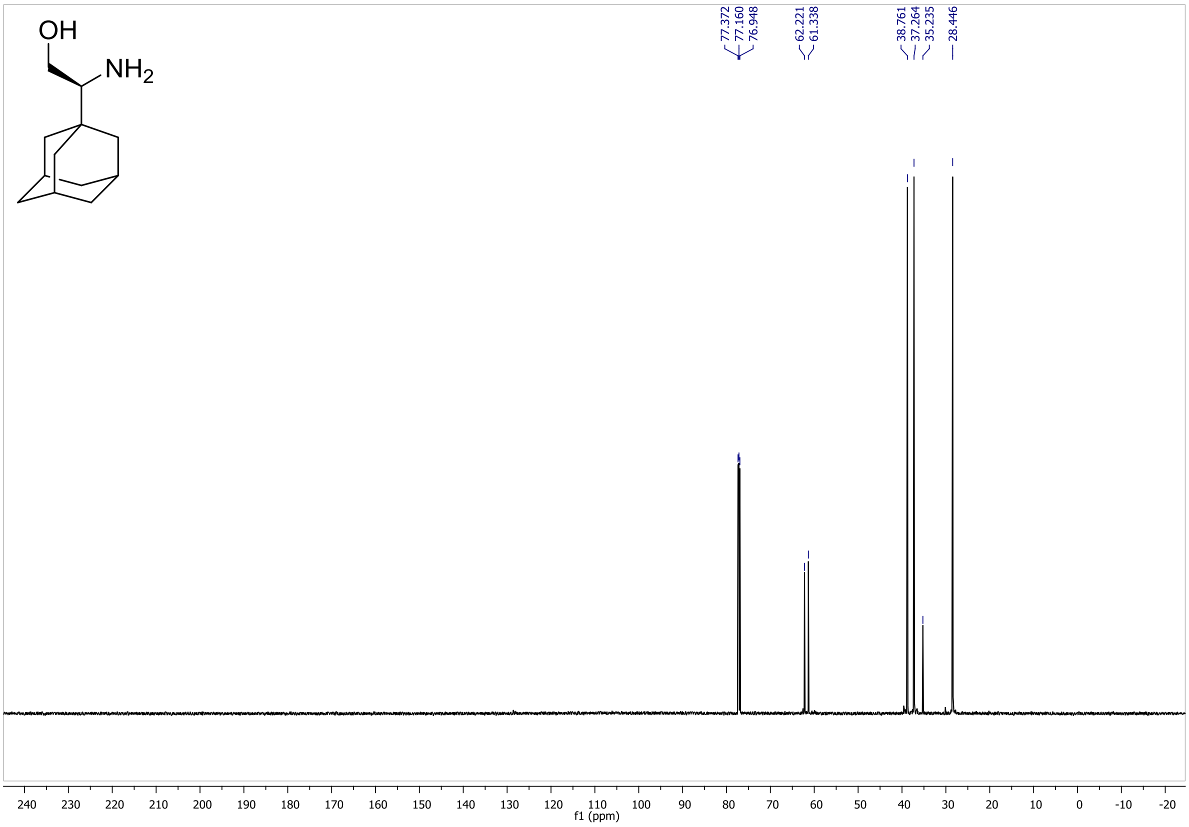

N^2,N^6-bis((*S*)-1-(adamantan-1-yl)-2-hydroxyethyl)pyridine-2,6-dicarboxamide:

^{1}H NMR, 600 MHz, $CDCl_3$:

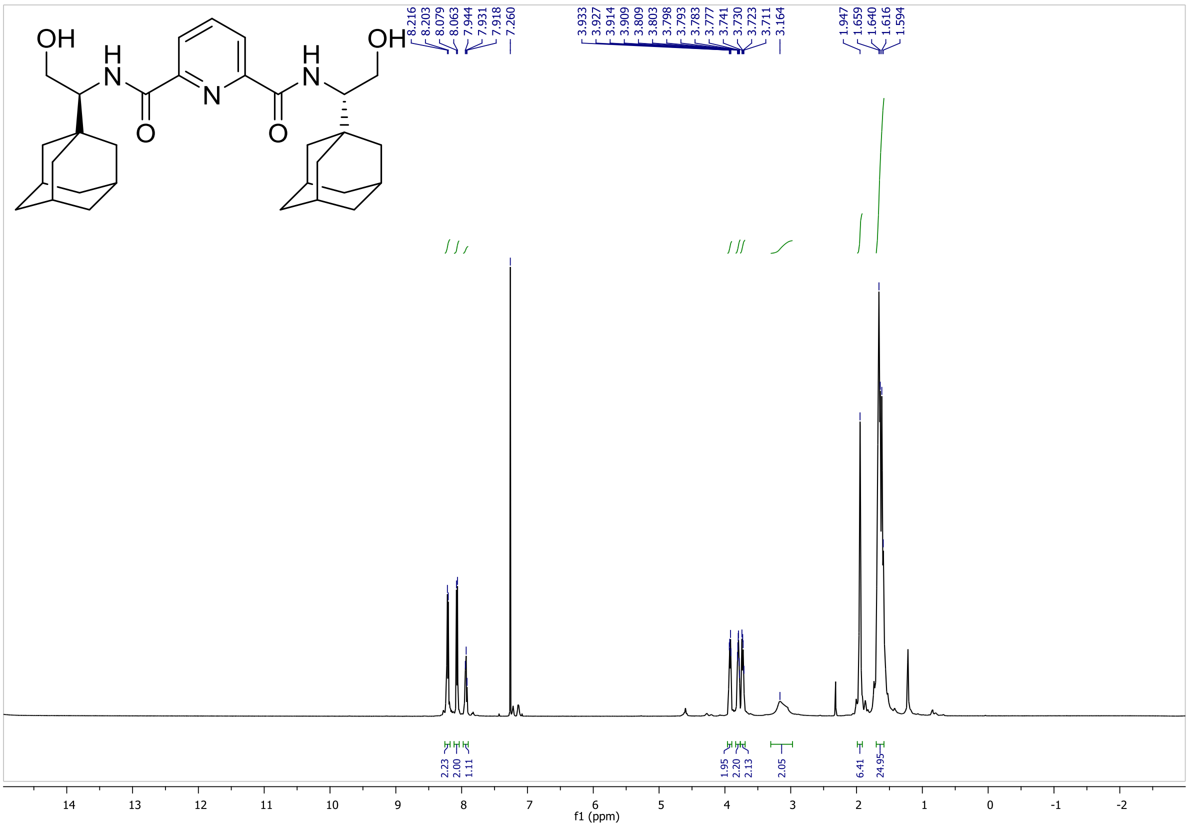

^{13}C NMR, 150 MHz, $CDCl_3$:

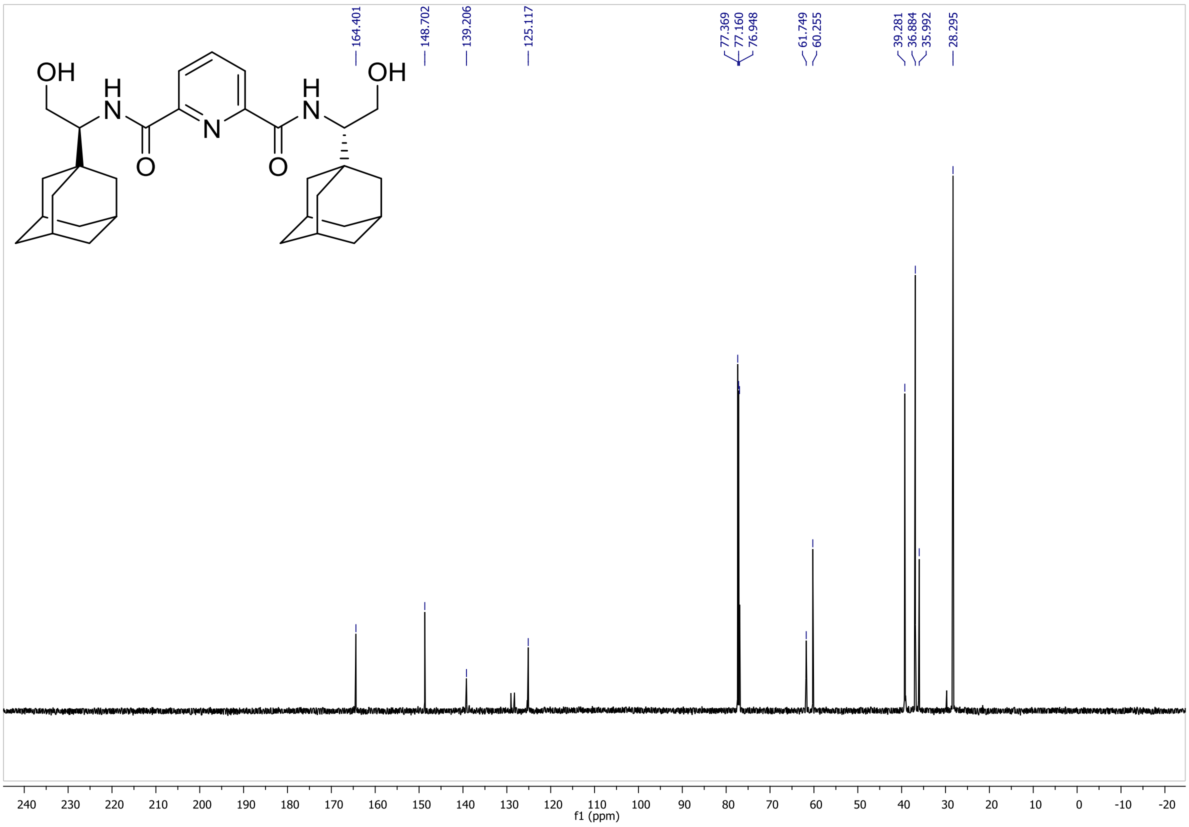

2,6-bis((*S*)-4-(adamantan-1-yl)-1-(4-(pentafluorosulfanyl)phenyl)-4,5-dihydro-1*H*-imidazol-2-yl)pyridine (L12):

^{1}H NMR, 600 MHz, CDCl$_3$:

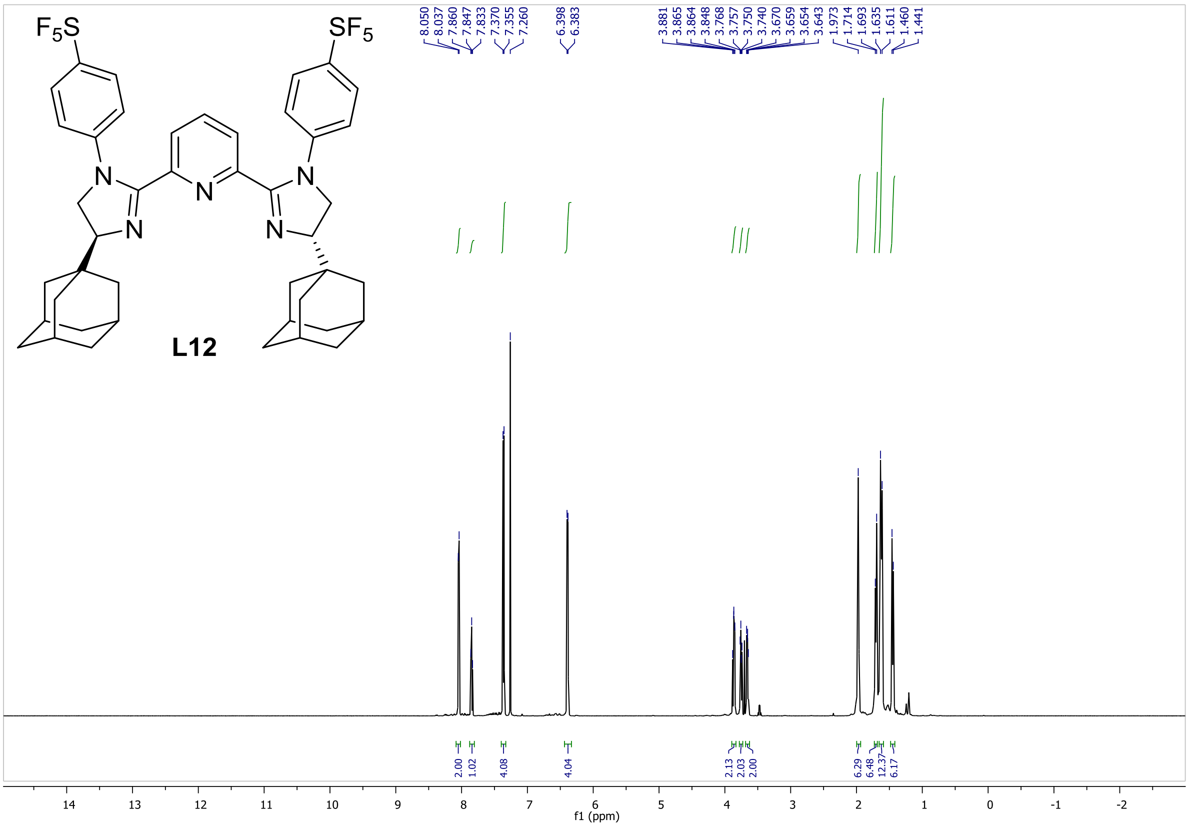

^{13}C NMR, 150 MHz, CDCl$_3$:

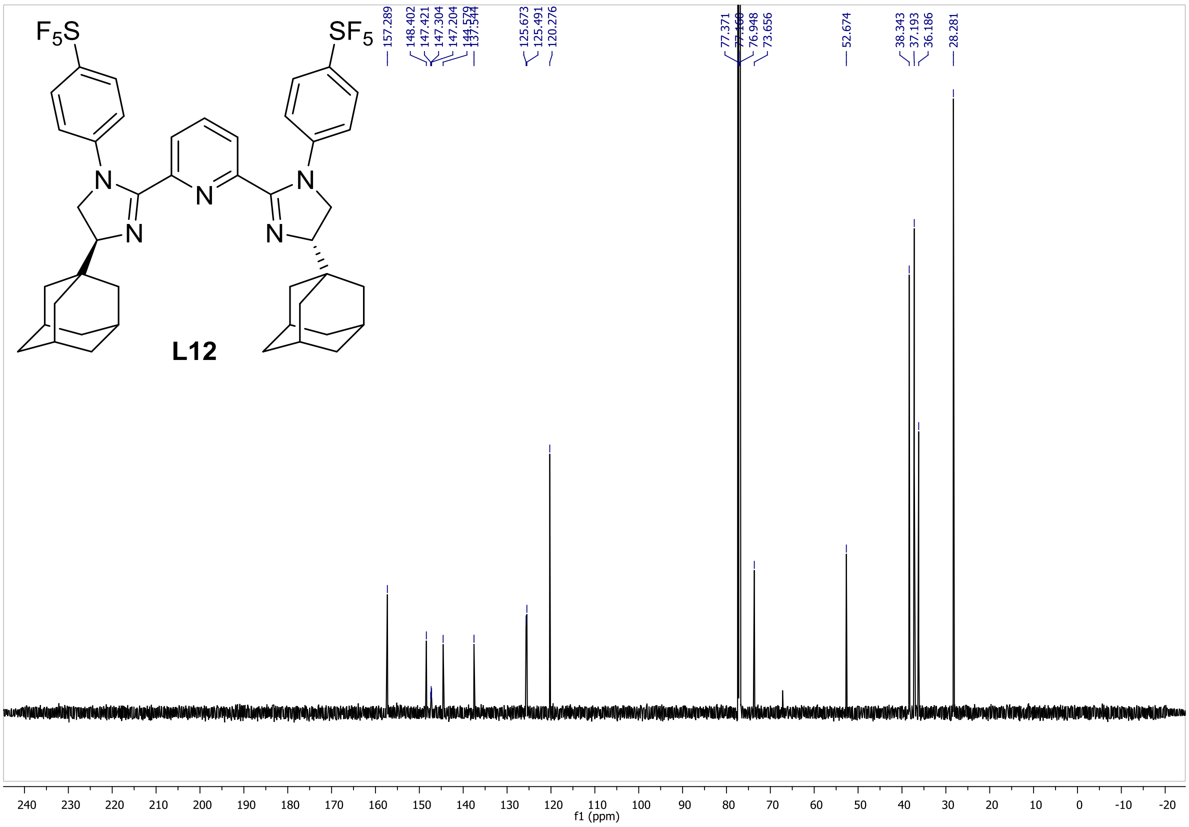

^{19}F NMR, 376 MHz, CDCl$_3$:

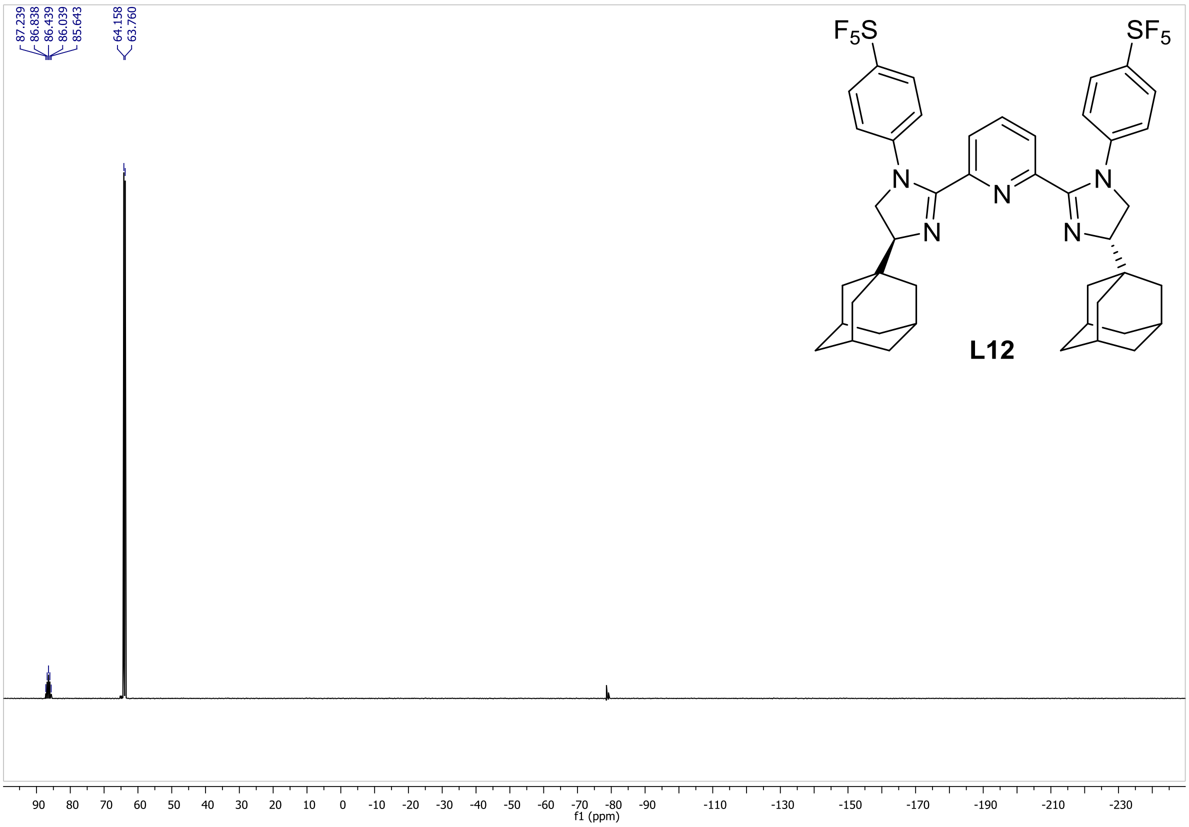

2,6-bis((*S*)-4-(adamantan-1-yl)-1-(4-((trifluoromethyl)sulfonyl)phenyl)-4,5-dihydro-1*H*-imidazol-2-yl)pyridine:

^{1}H NMR, 600 MHz, $CDCl_3$:

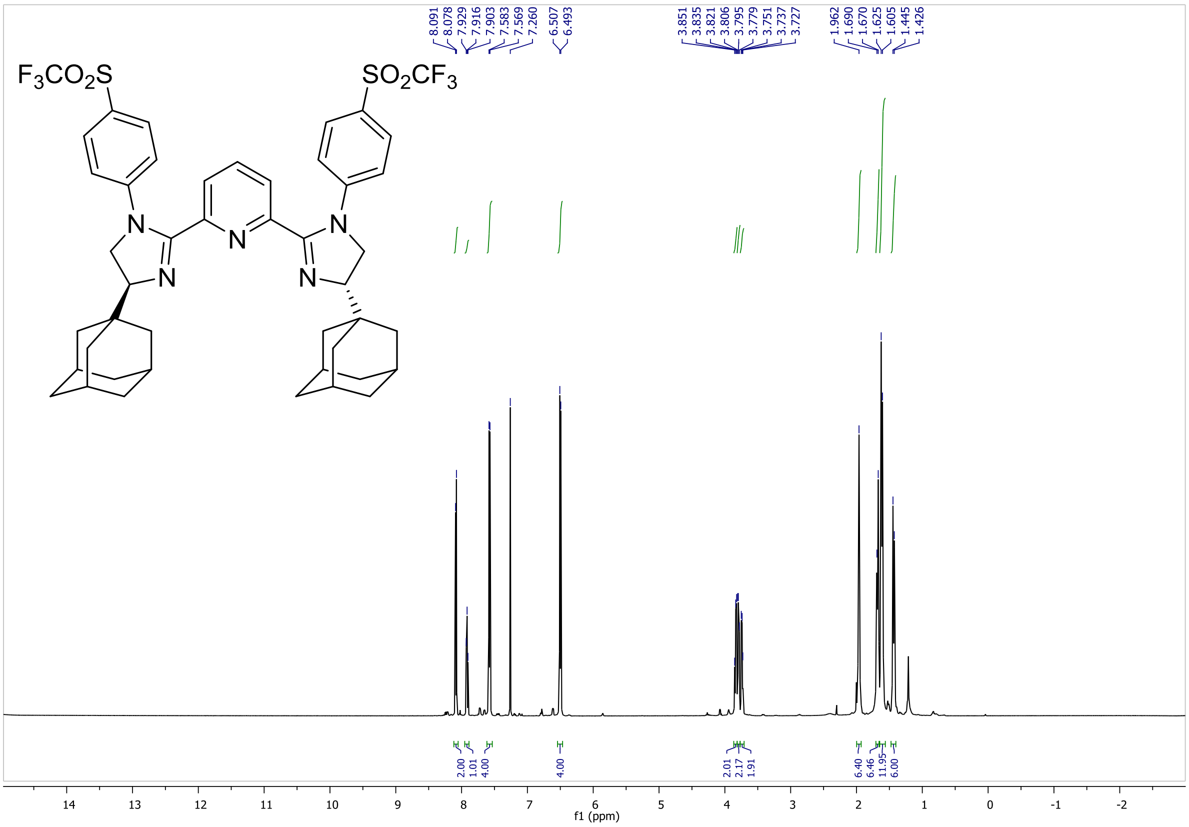

^{13}C NMR, 150 MHz, $CDCl_3$:

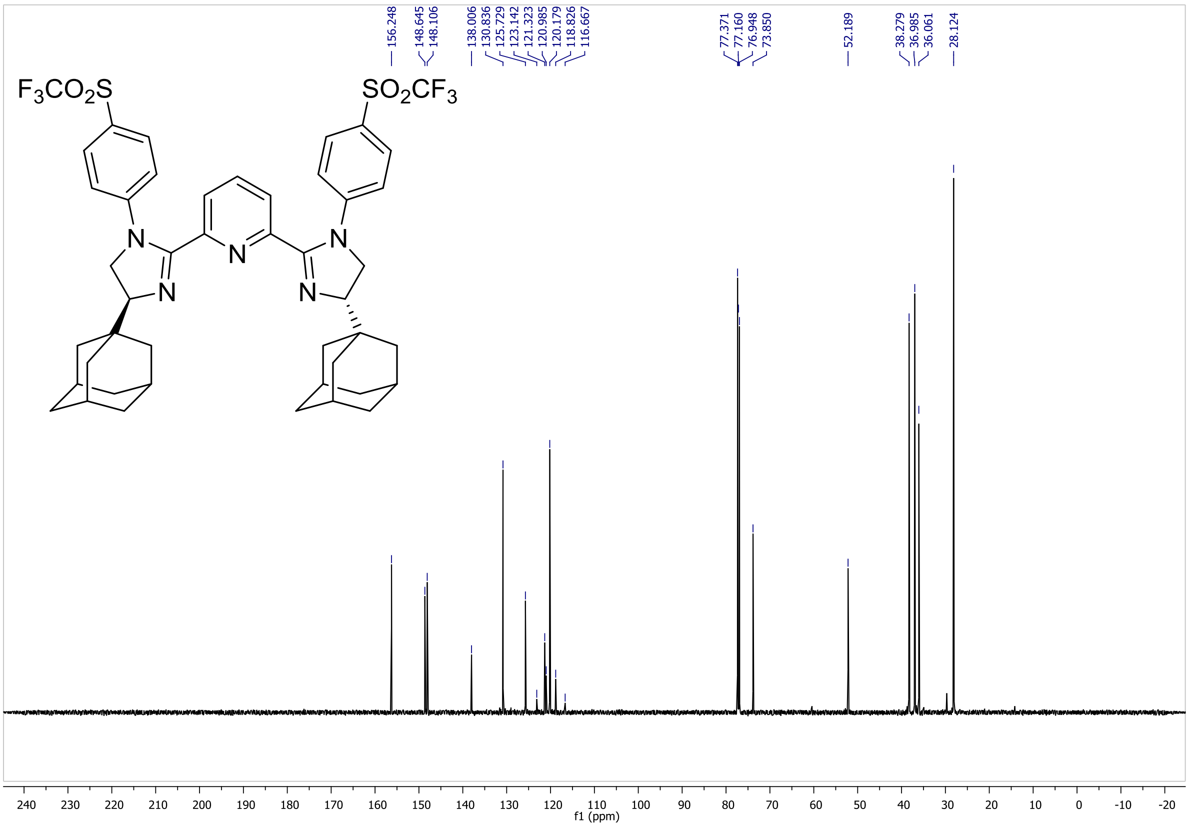

^{19}F NMR, 376 MHz, $CDCl_3$:

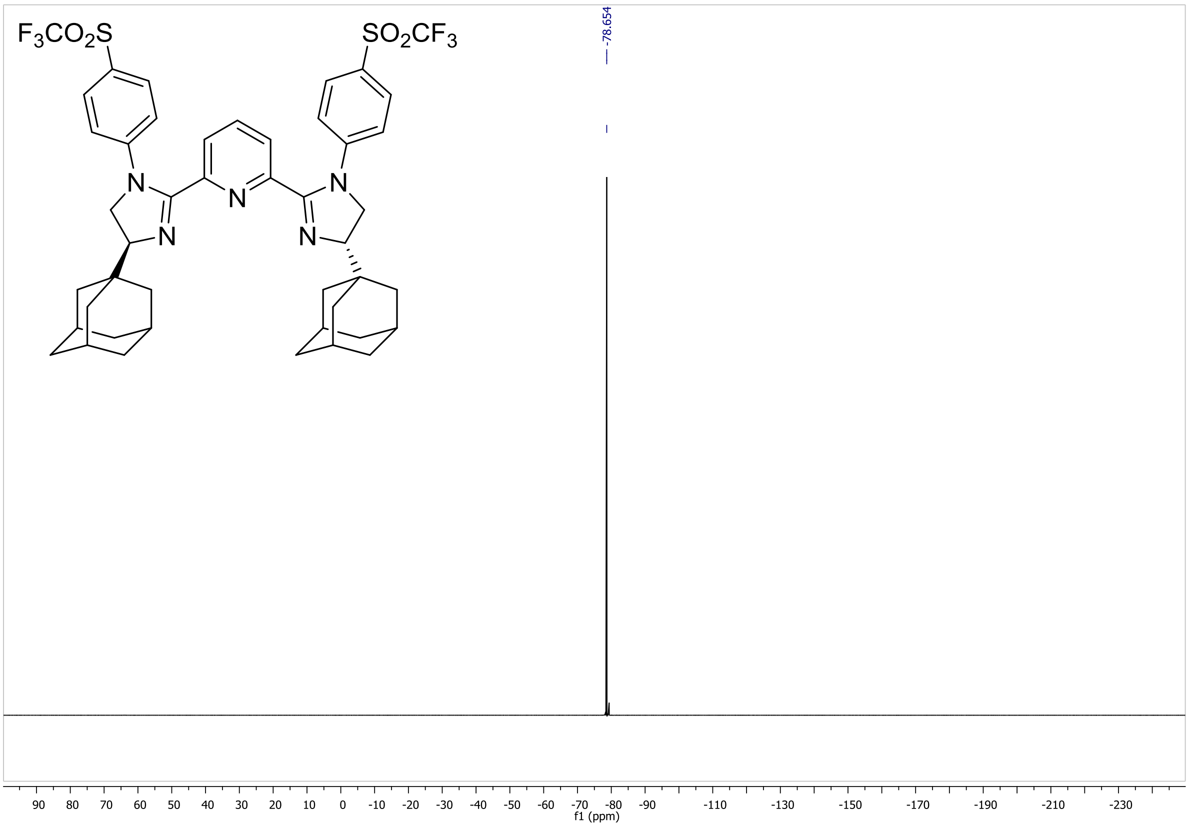

8.3. NMR spectra for alkyne starting materials

4-methyl-*N*-(prop-2-yn-1-yl)benzenesulfonamide (3a):

^{1}H NMR, 600 MHz, $CDCl_3$:

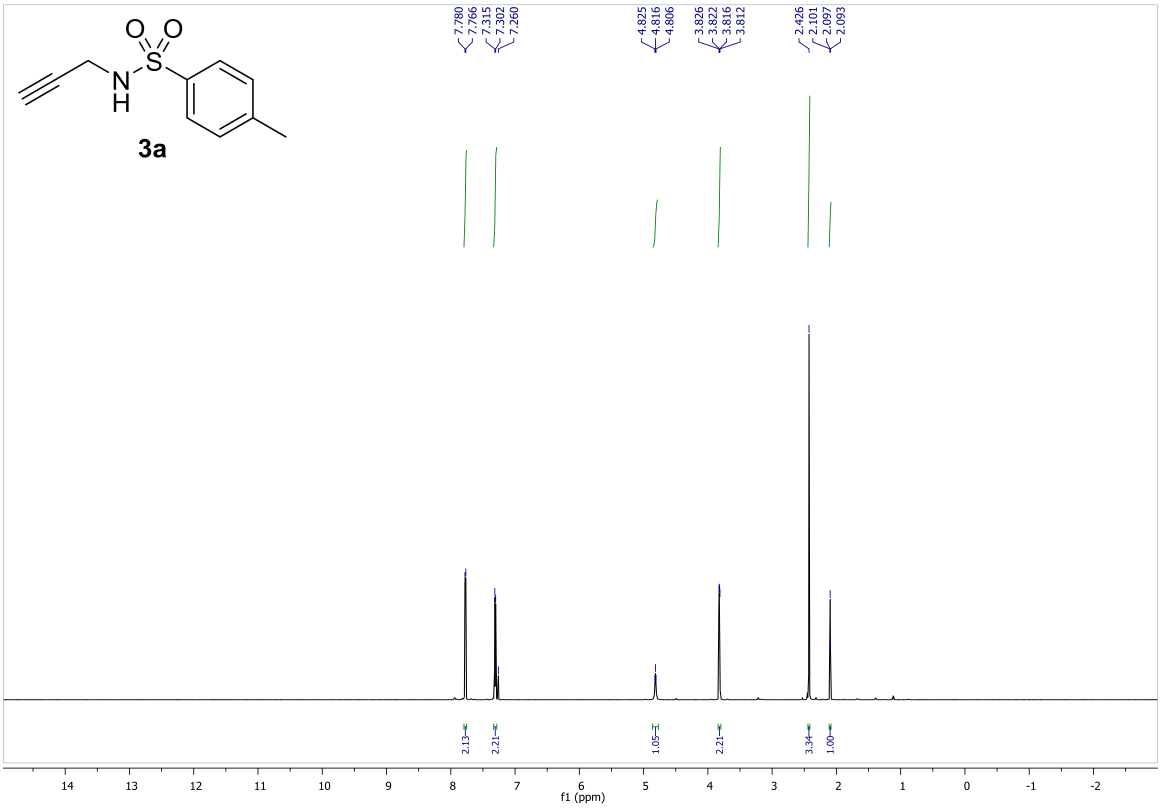

^{13}C NMR, 150 MHz, $CDCl_3$:

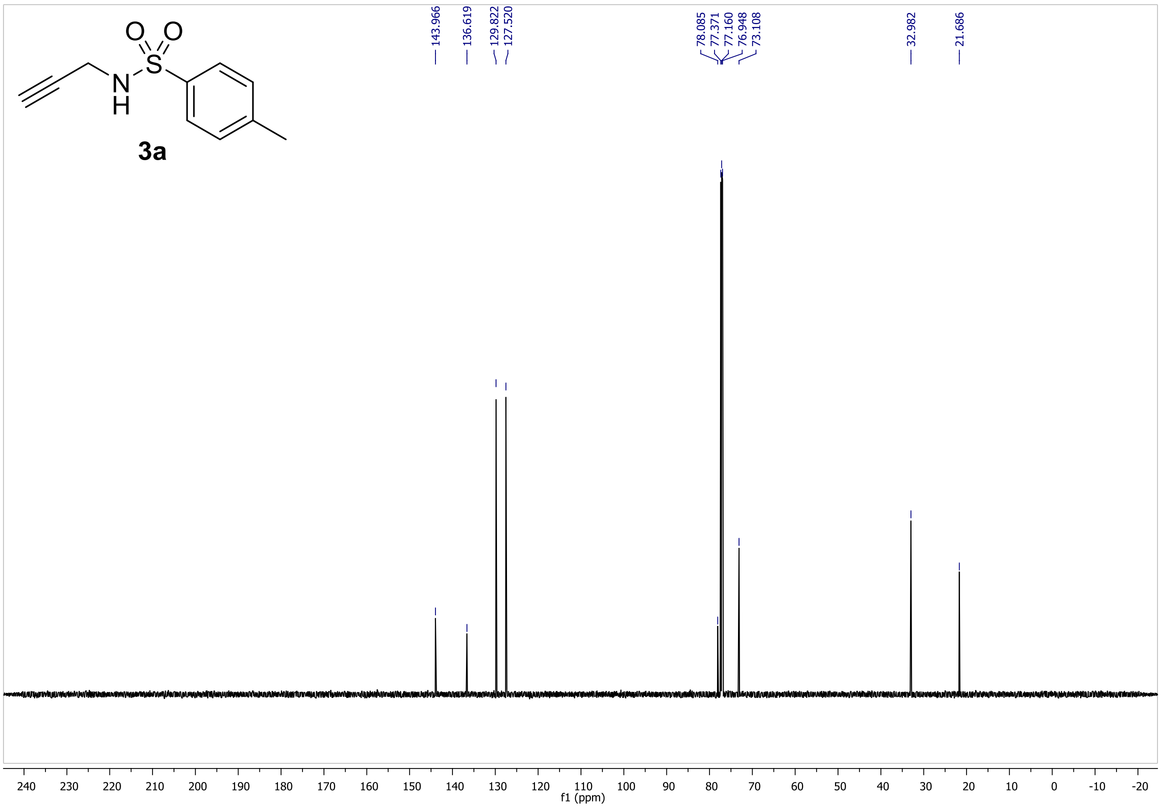

3,3,3-trifluoro-*N*-(prop-2-yn-1-yl)propanamide:

^{1}H NMR, 600 MHz, CDCl$_3$:

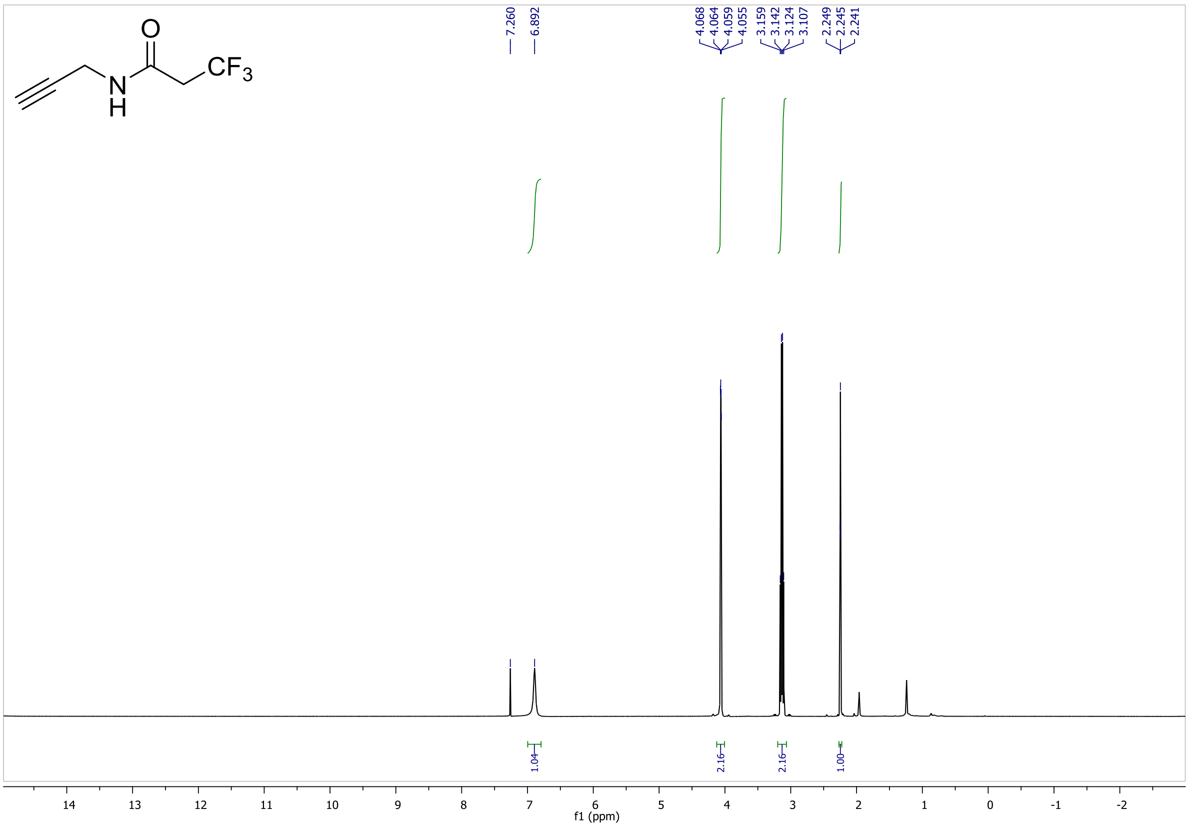

^{13}C NMR, 150 MHz, CDCl$_3$:

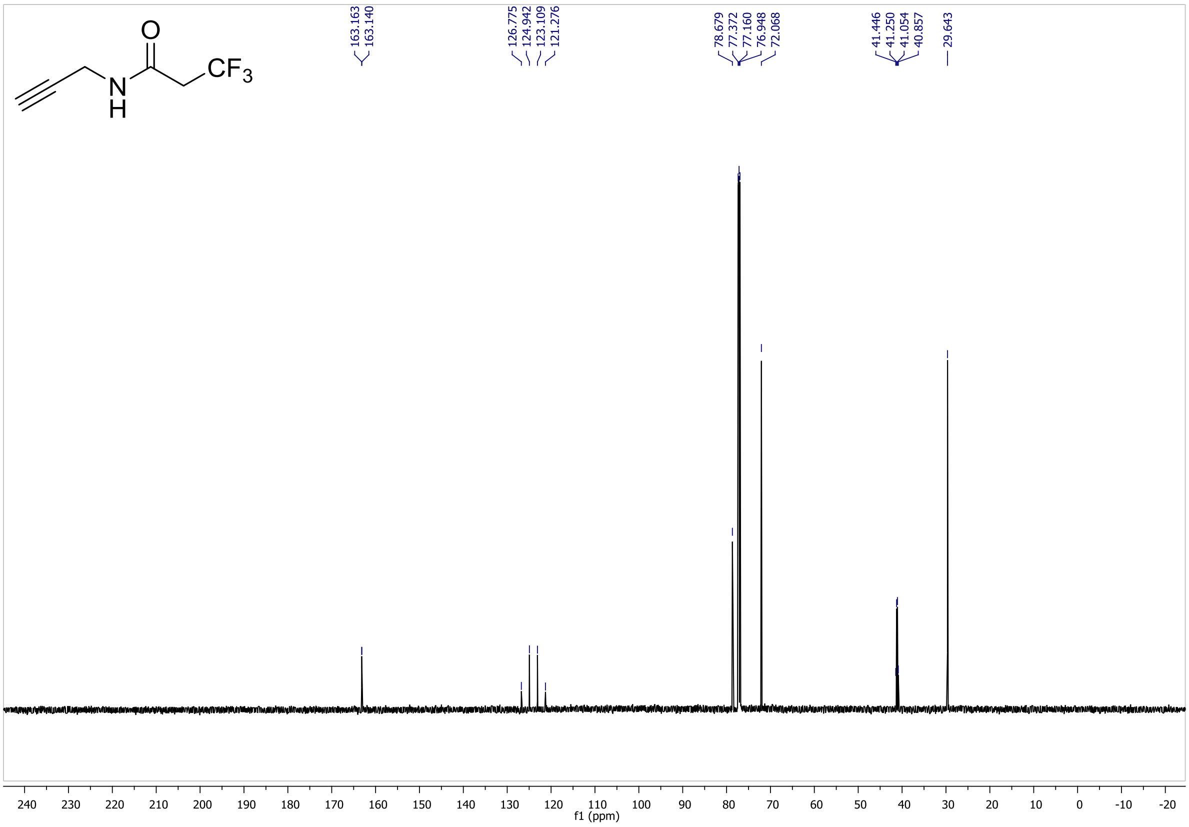

^{19}F NMR, 376 MHz, CDCl$_3$:

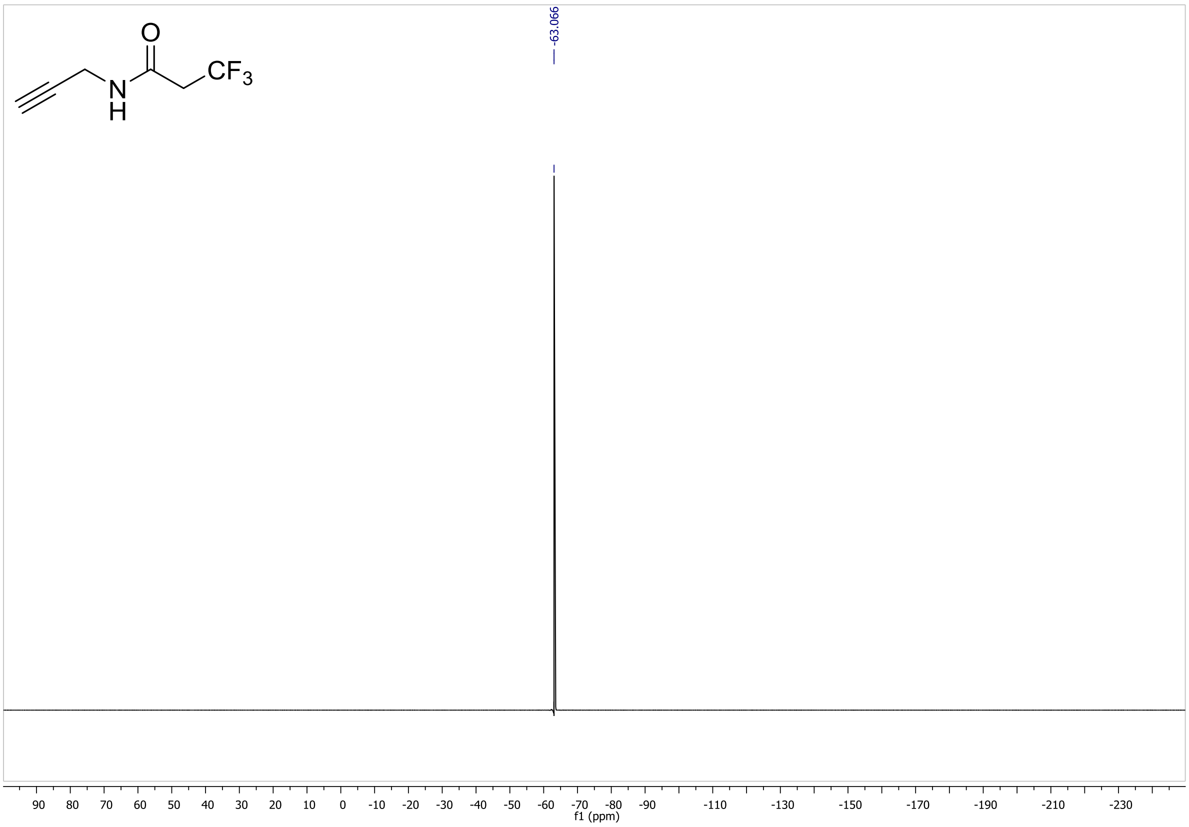

6-oxo-*N*-(prop-2-yn-1-yl)heptanamide:

^{1}H NMR, 600 MHz, $CDCl_3$:

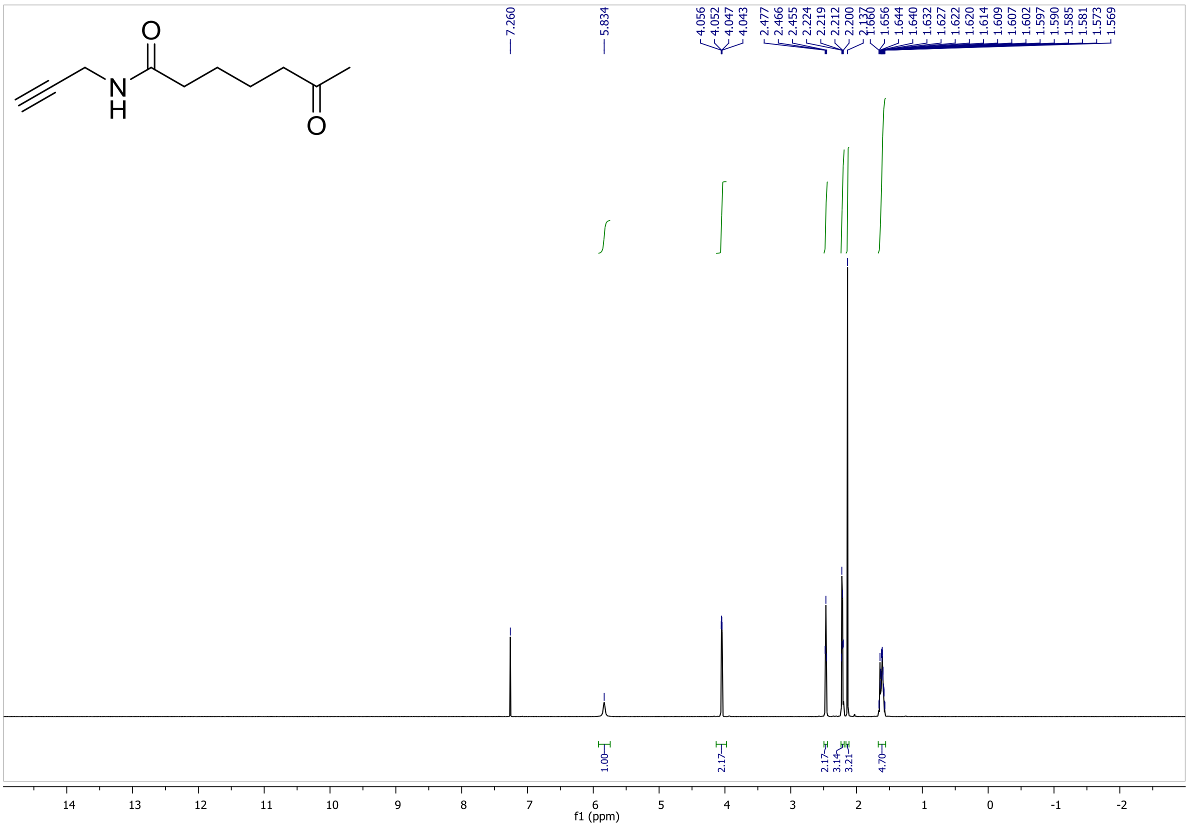

^{13}C NMR, 150 MHz, $CDCl_3$:

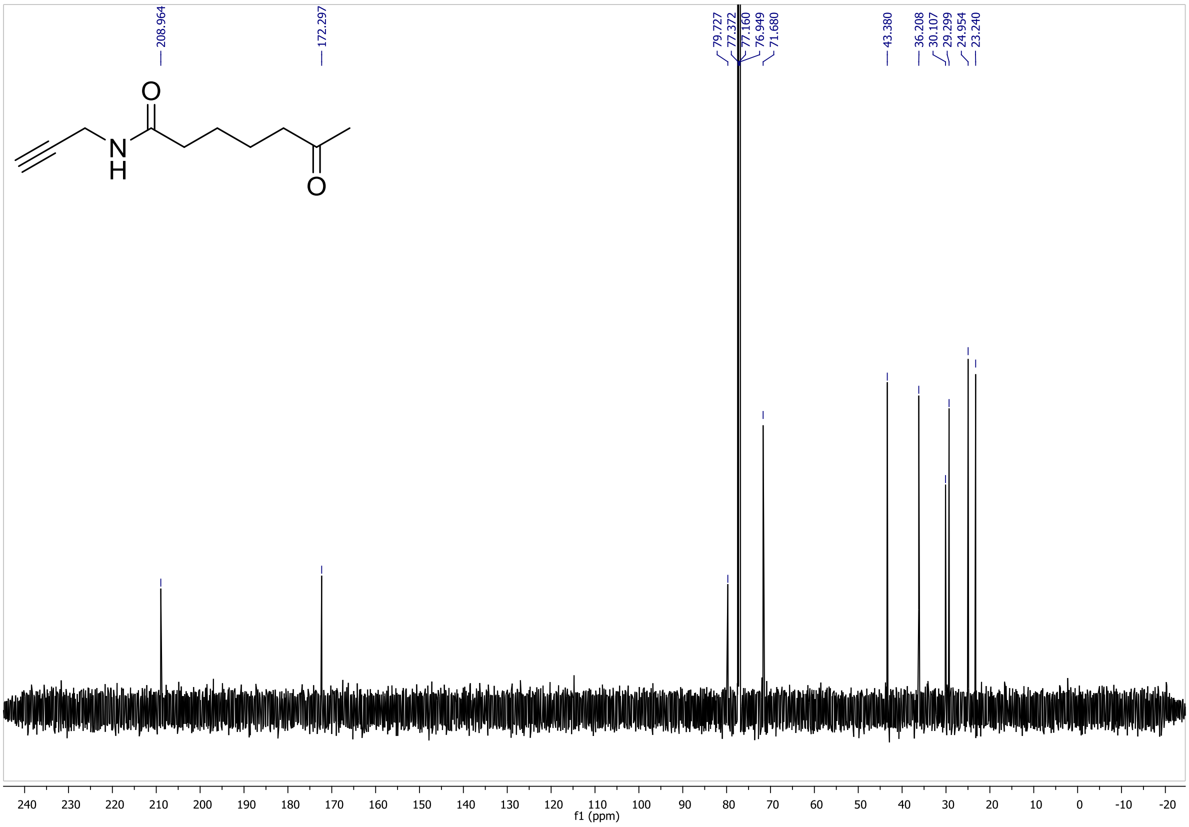

4-formyl-*N*-(prop-2-yn-1-yl)benzamide:

^{1}H NMR, 600 MHz, $CDCl_3$:

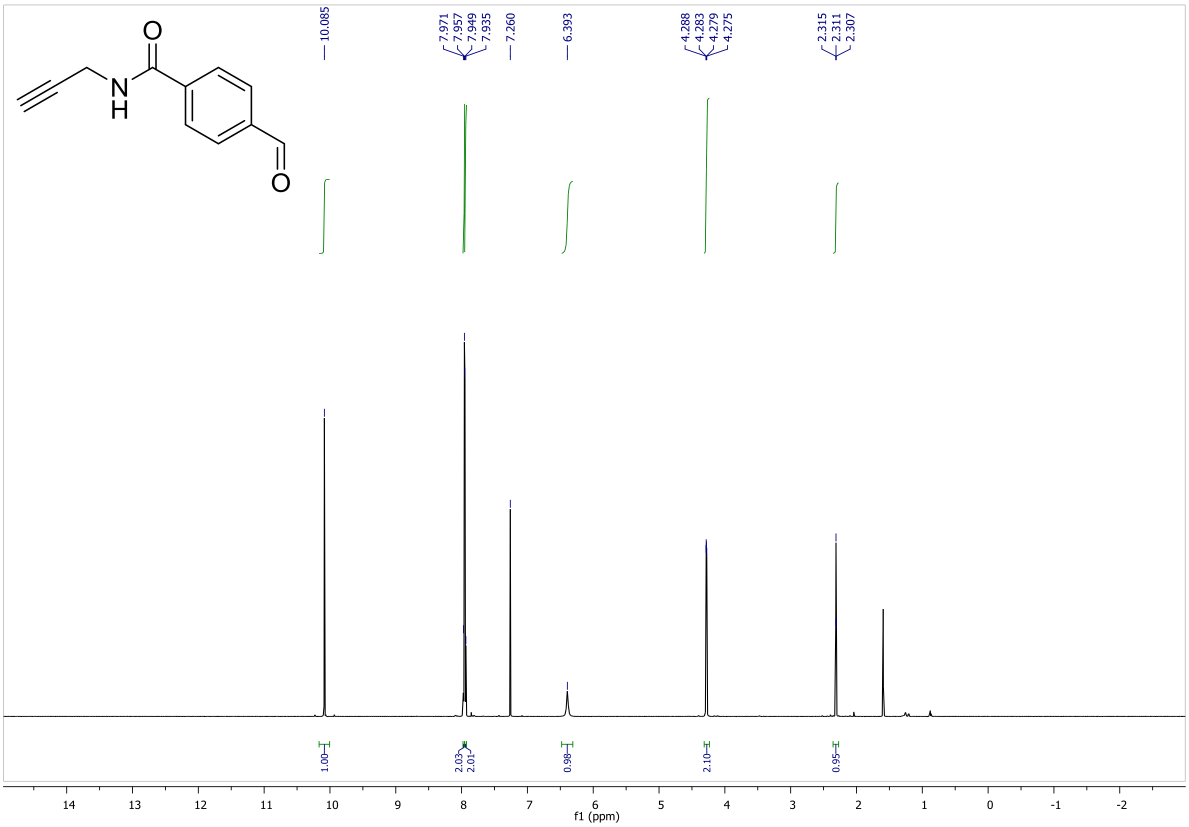

^{13}C NMR, 150 MHz, $CDCl_3$:

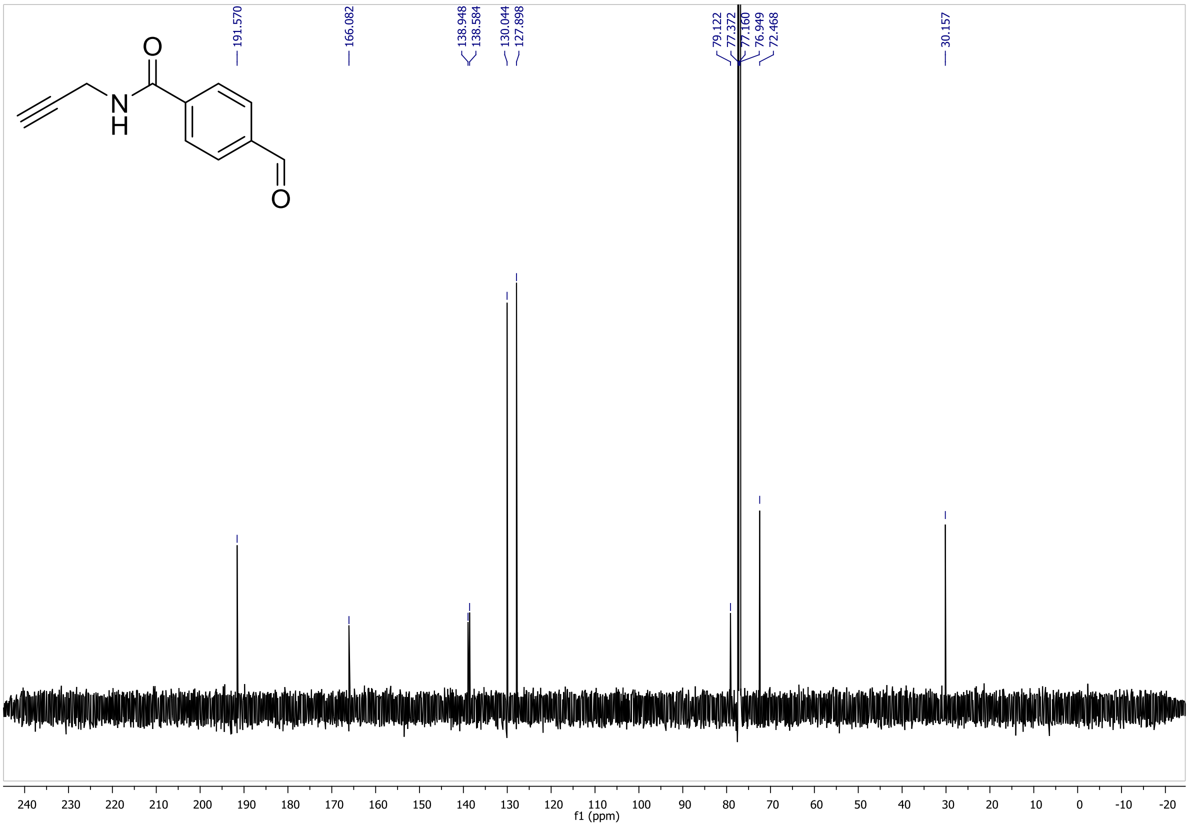

N-(prop-2-yn-1-yl)cyclopent-3-ene-1-carboxamide:

^{1}H NMR, 600 MHz, $CDCl_3$:

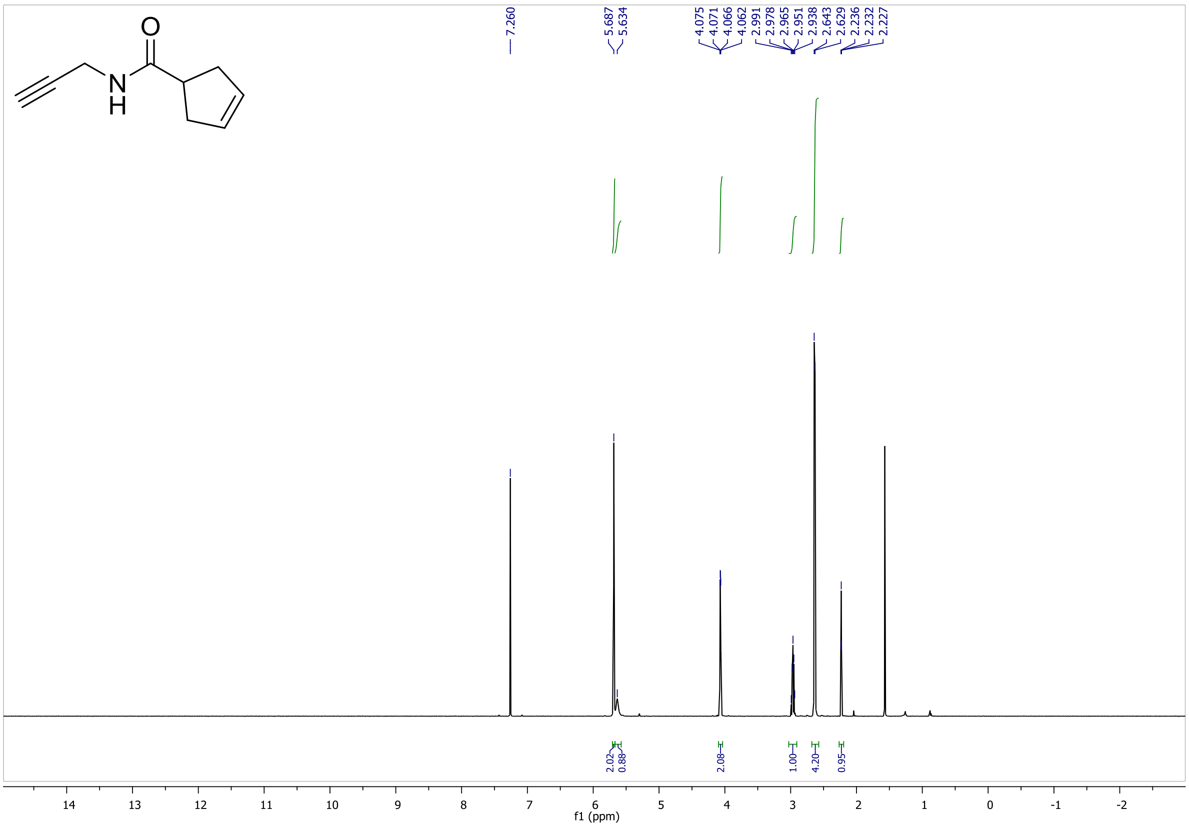

^{13}C NMR, 150 MHz, $CDCl_3$:

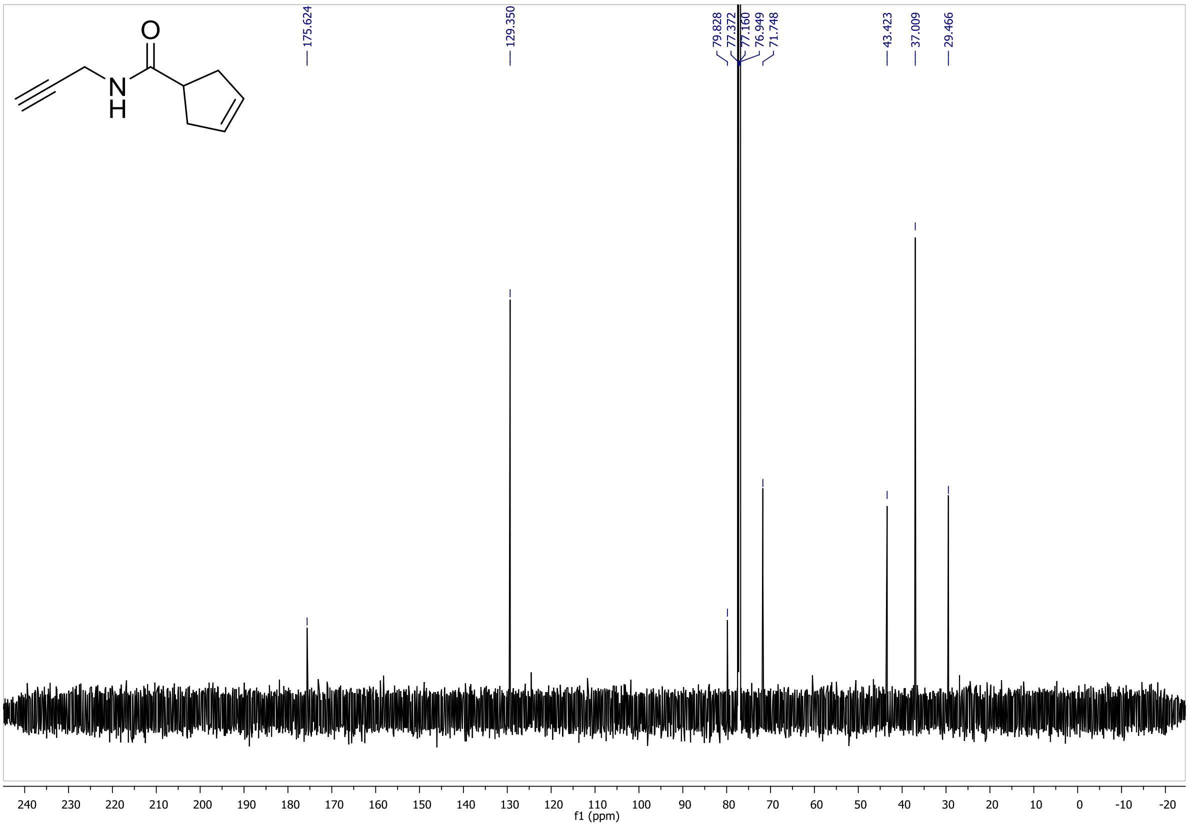

(1*R*,3*s*,5*S*)-*N*-(prop-2-yn-1-yl)-6-oxabicyclo[3.1.0]hexane-3-carboxamide:

^{1}H NMR, 600 MHz, $CDCl_3$:

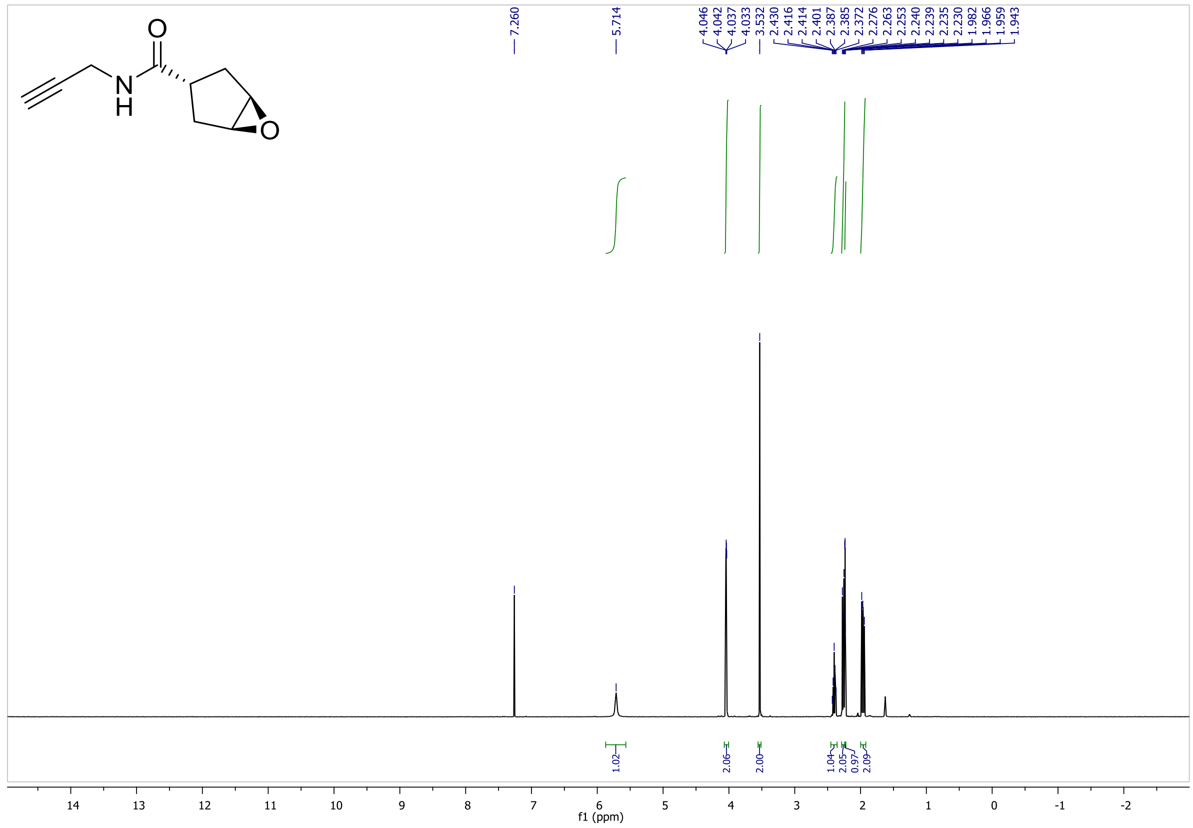

^{13}C NMR, 150 MHz, $CDCl_3$:

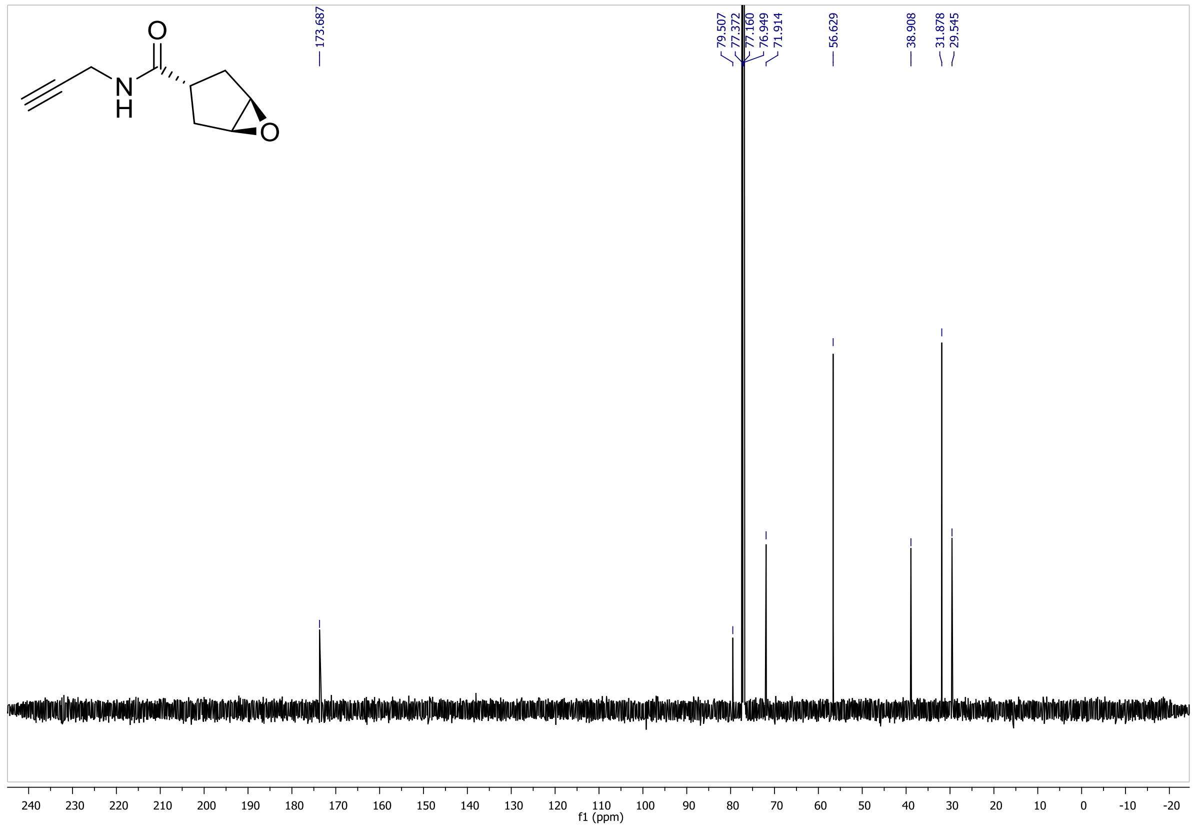

(1*R*,3*r*,5*S*)-*N*-(prop-2-yn-1-yl)-6-oxabicyclo[3.1.0]hexane-3-carboxamide:

^{1}H NMR, 600 MHz, $CDCl_3$:

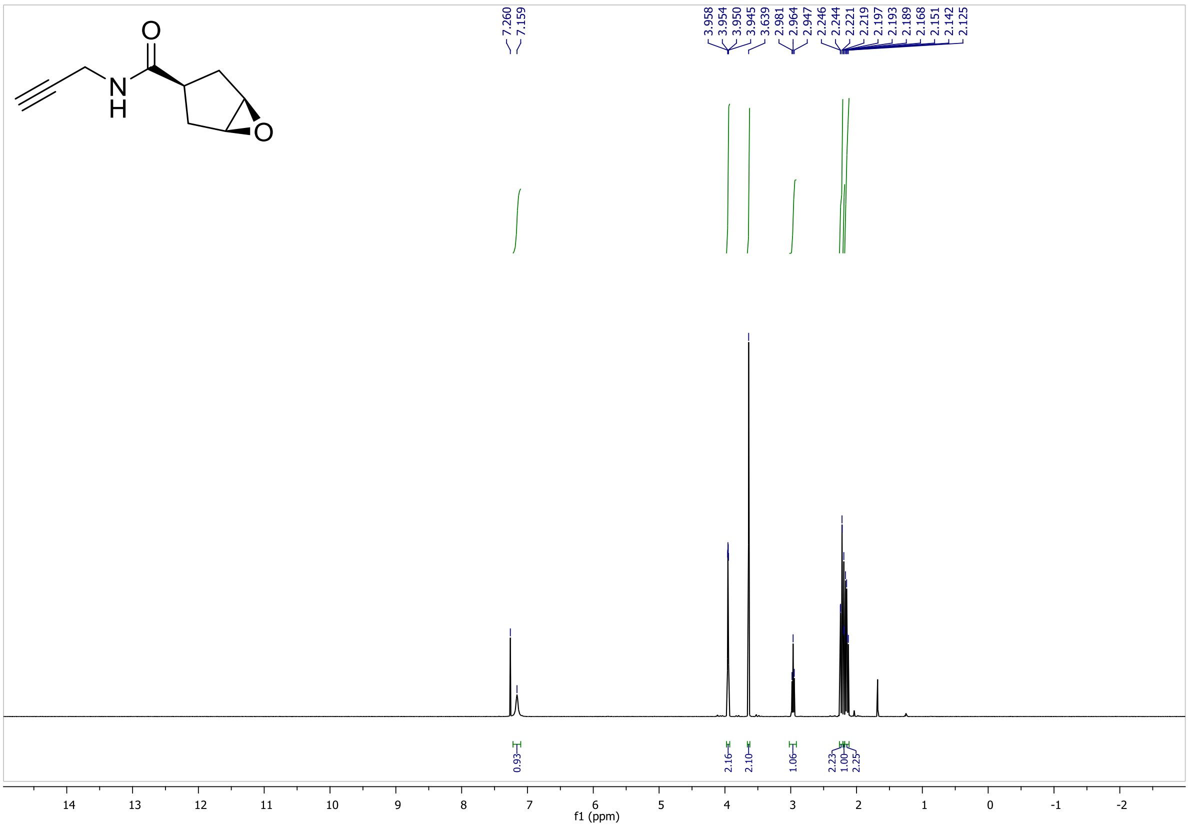

^{13}C NMR, 150 MHz, $CDCl_3$:

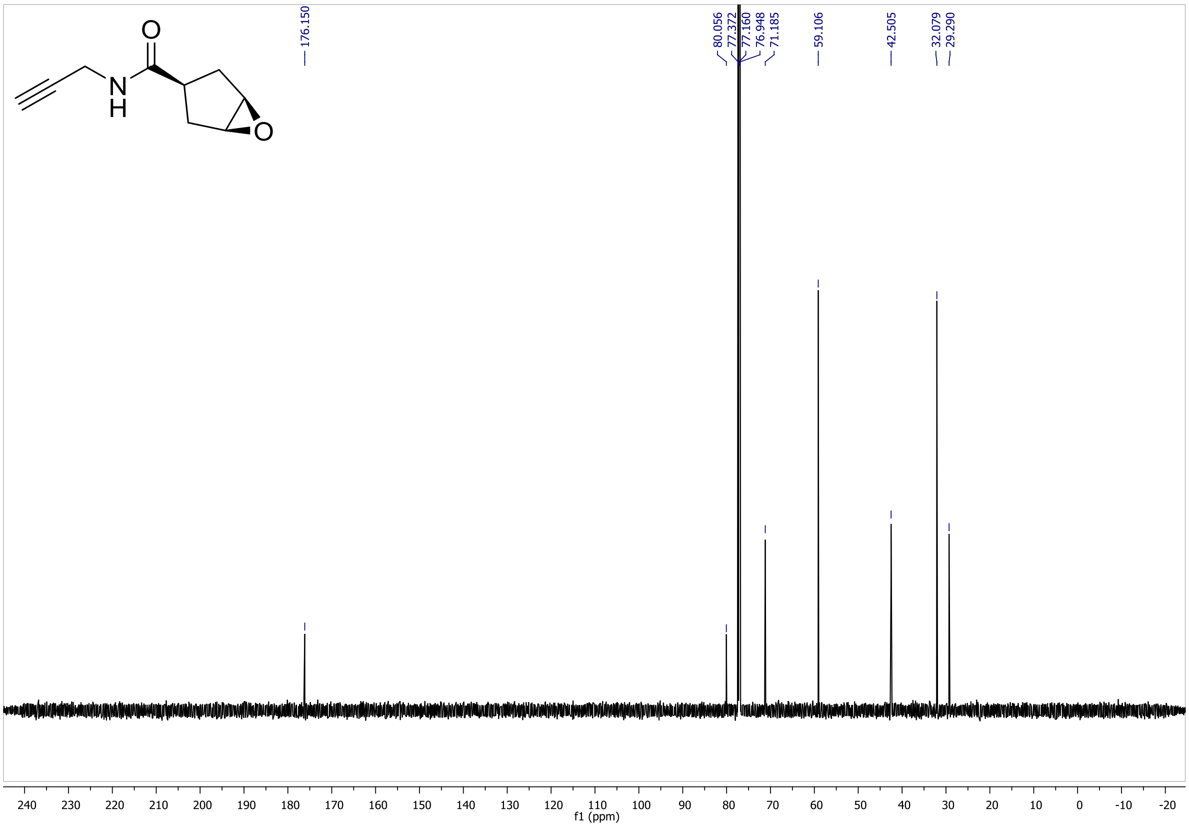

***N*-(prop-2-yn-1-yl)thiophene-2-carboxamide:**

^{1}H NMR, 600 MHz, $CDCl_3$:

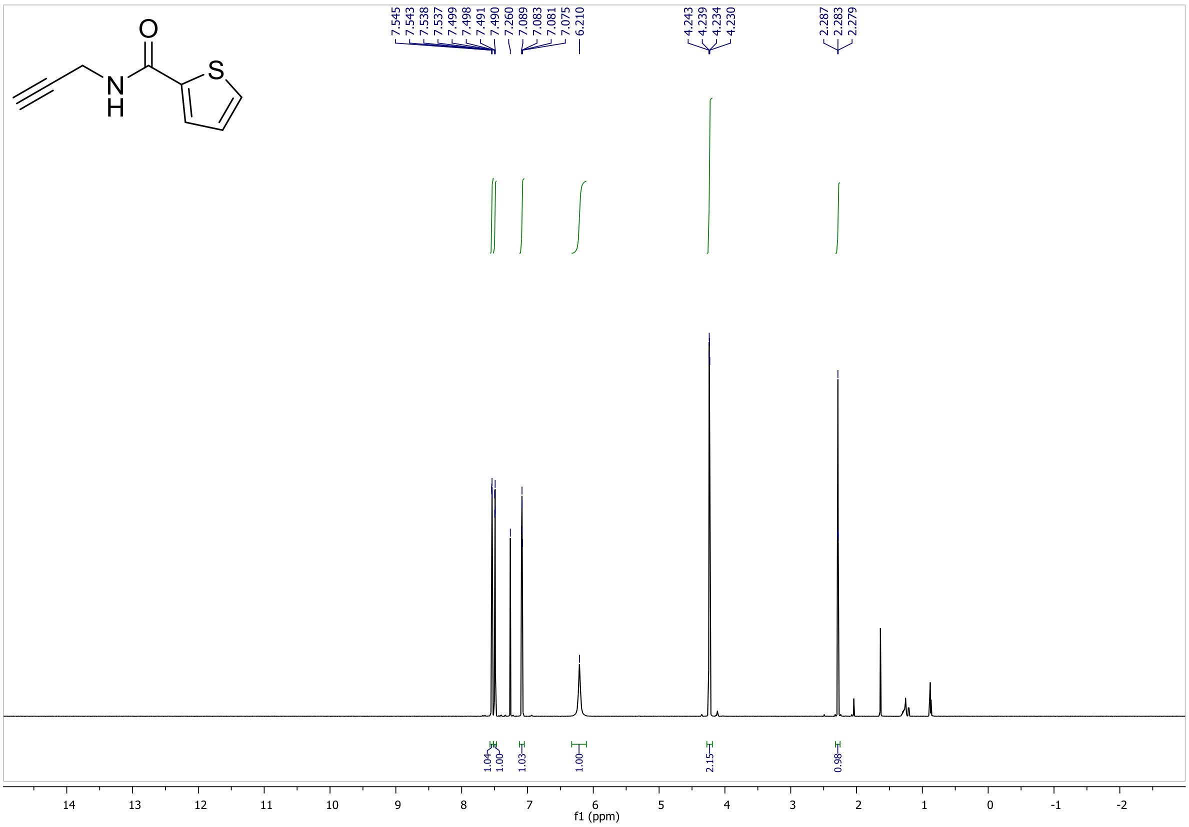

^{13}C NMR, 150 MHz, $CDCl_3$:

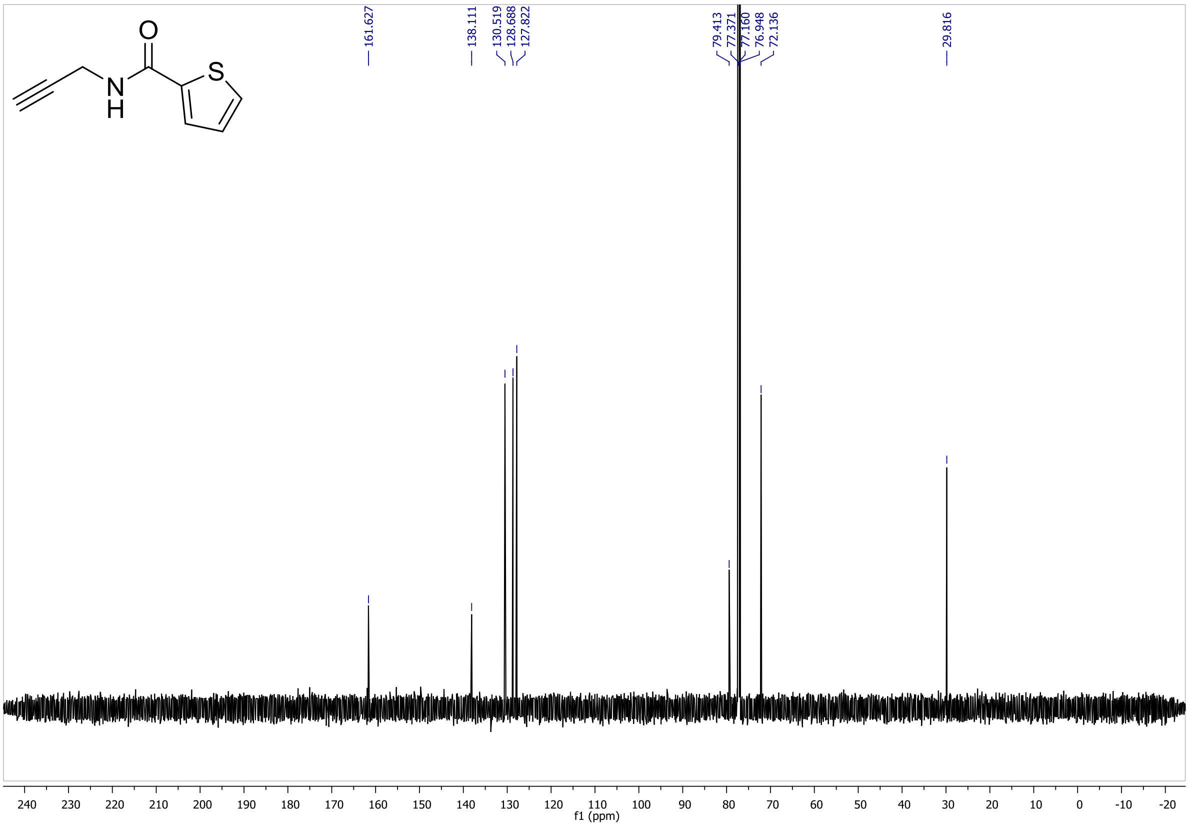

1-(4-chlorophenyl)-*N*-(prop-2-yn-1-yl)cyclopentane-1-carboxamide:

^{1}H NMR, 600 MHz, CDCl$_3$:

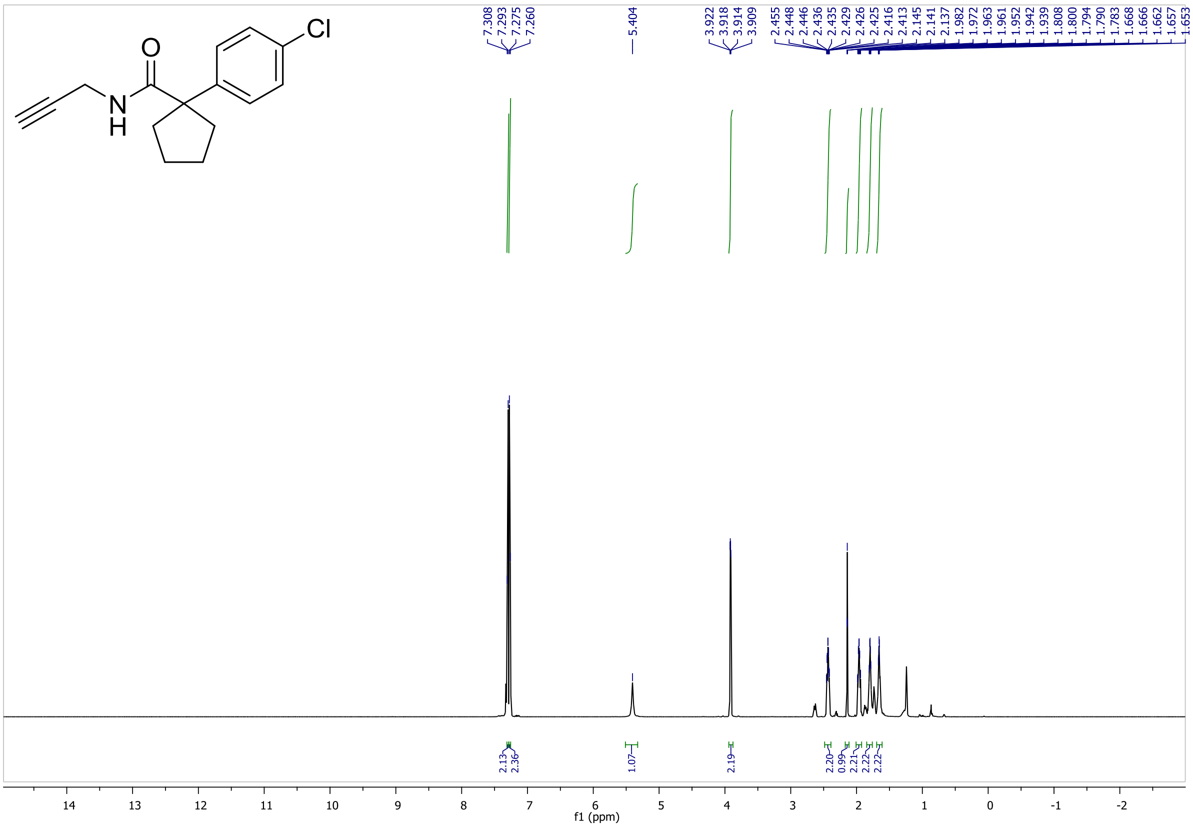

^{13}C NMR, 150 MHz, CDCl$_3$:

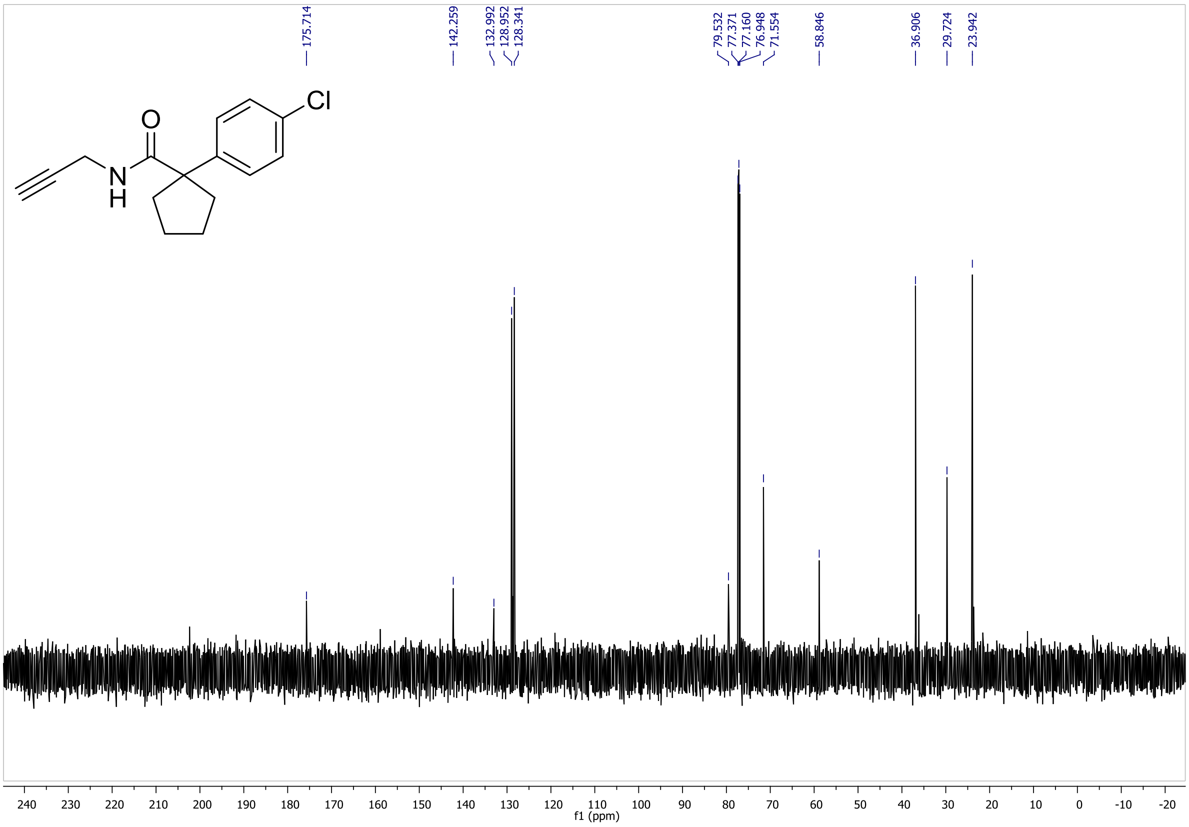

4-hydroxy-*N*-(prop-2-yn-1-yl)butanamide:

^{1}H NMR, 600 MHz, $CDCl_3$:

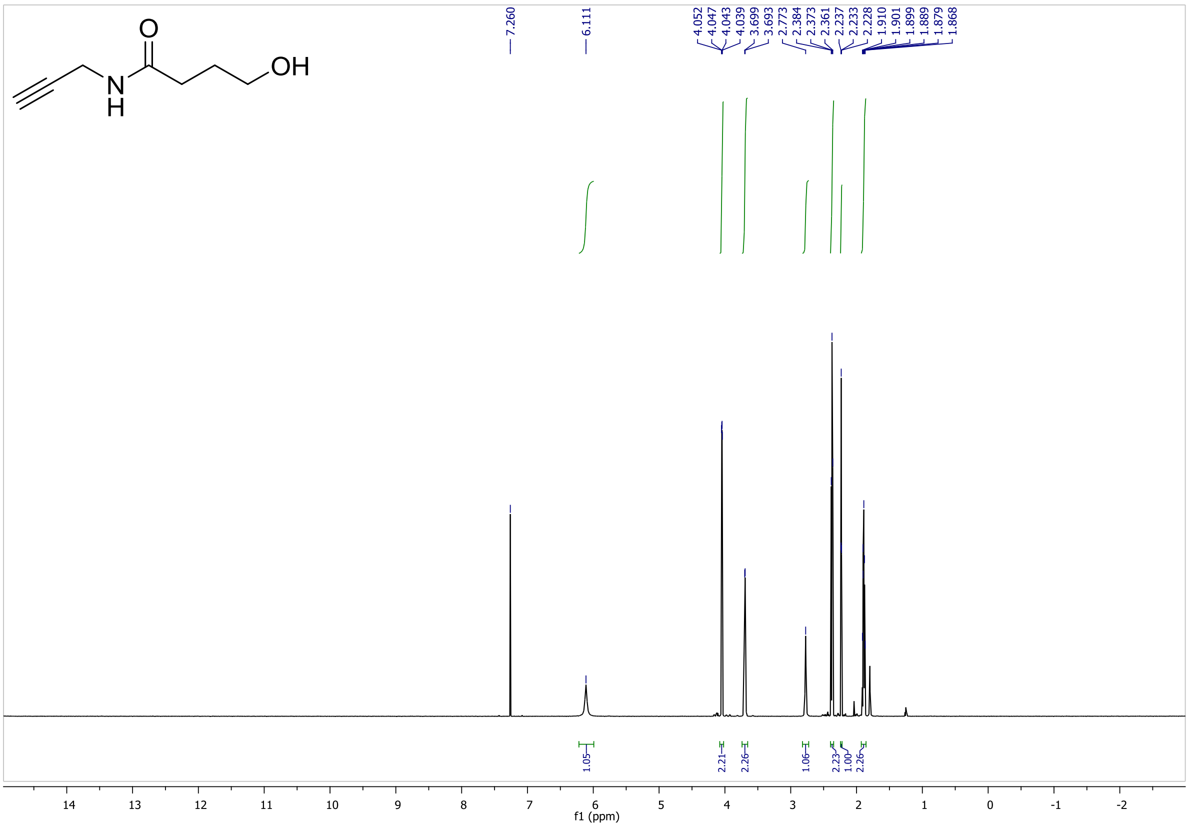

^{13}C NMR, 150 MHz, $CDCl_3$:

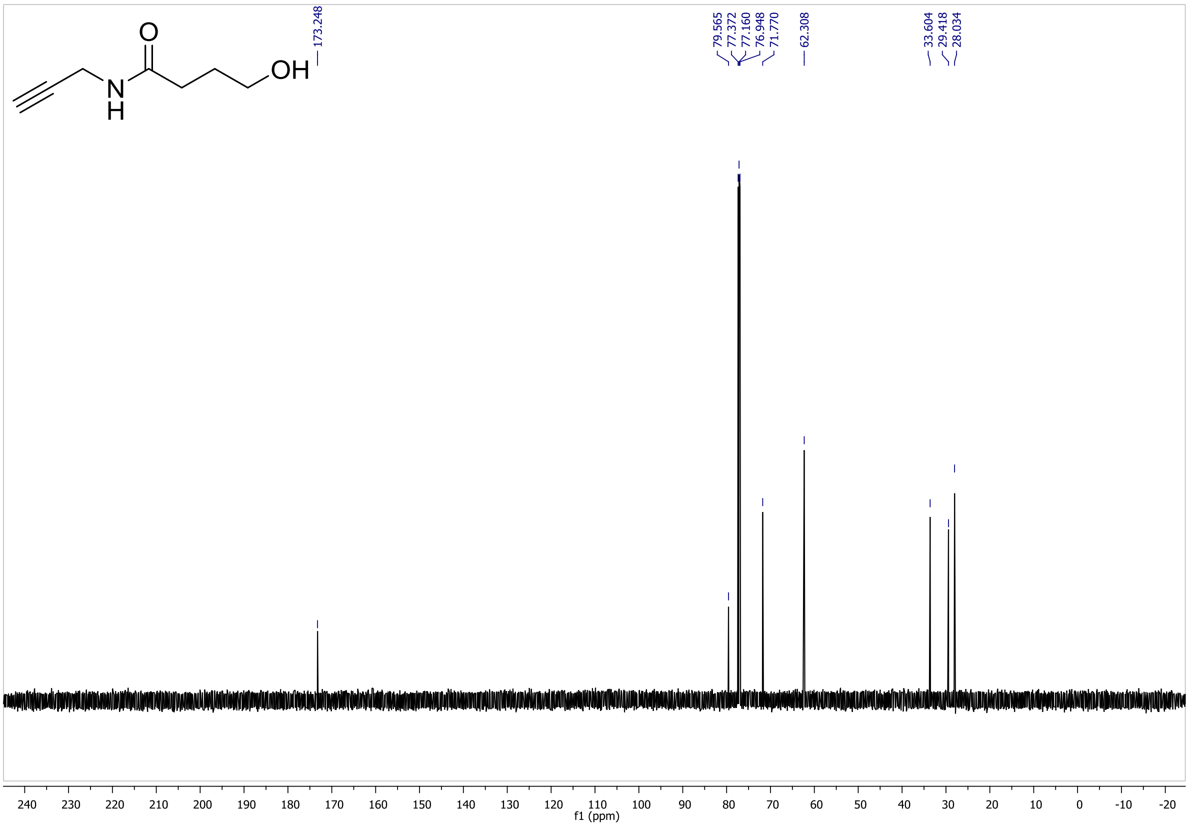

5-bromo-*N*-(prop-2-yn-1-yl)furan-2-carboxamide:

^{1}H NMR, 600 MHz, $CDCl_3$:

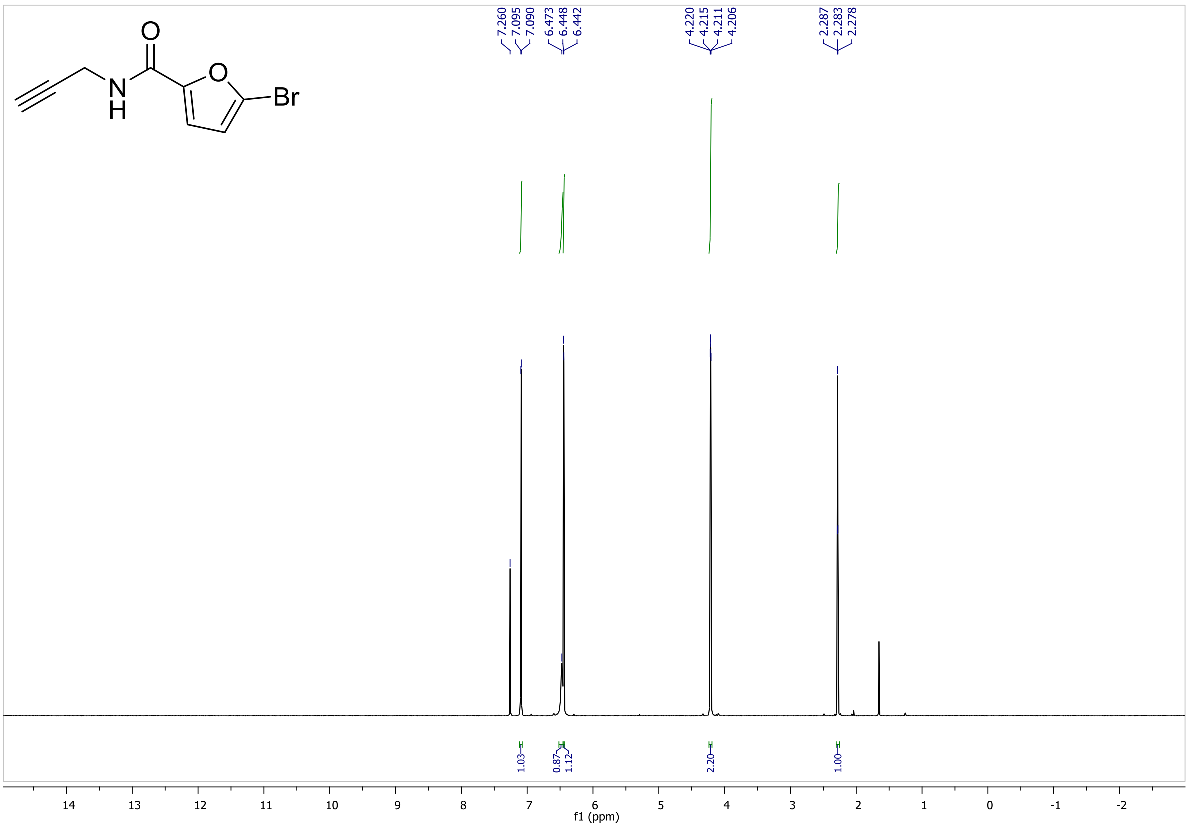

^{13}C NMR, 150 MHz, $CDCl_3$:

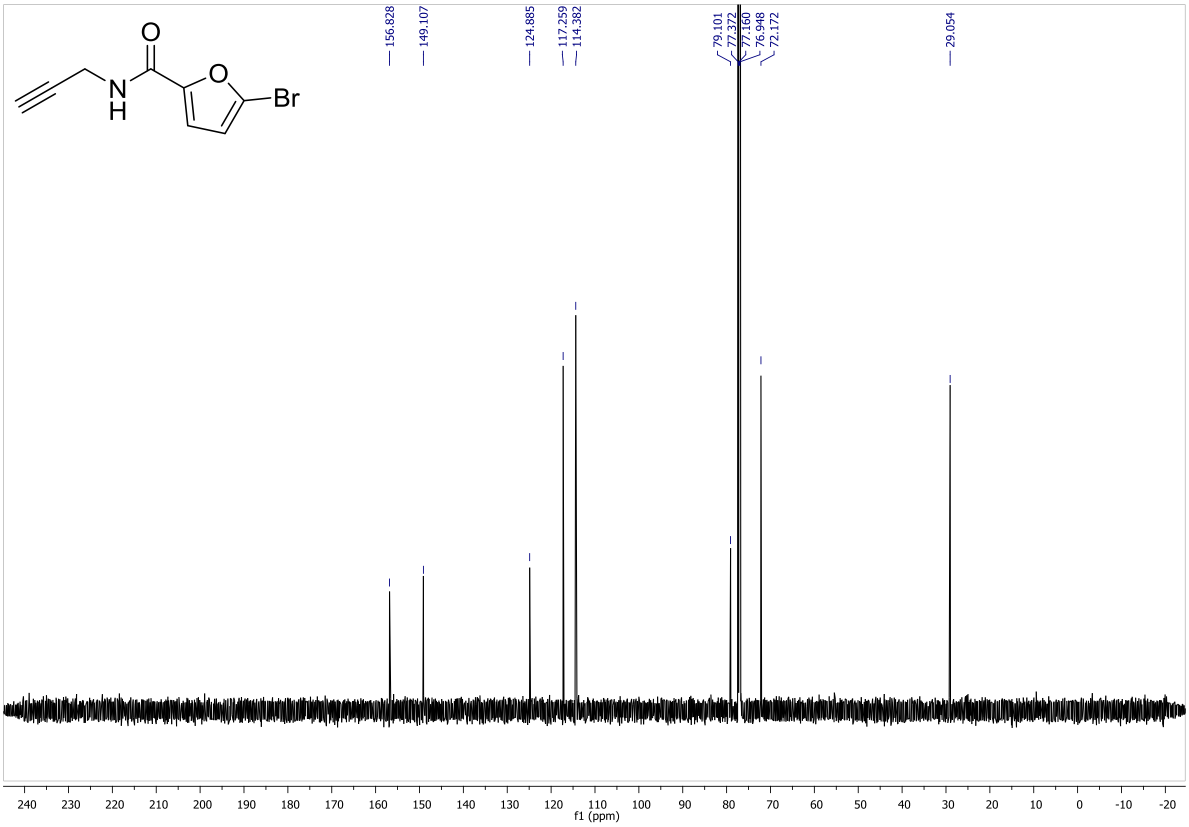

N-(prop-2-yn-1-yl)cyclopropanecarboxamide:

^{1}H NMR, 600 MHz, $CDCl_3$:

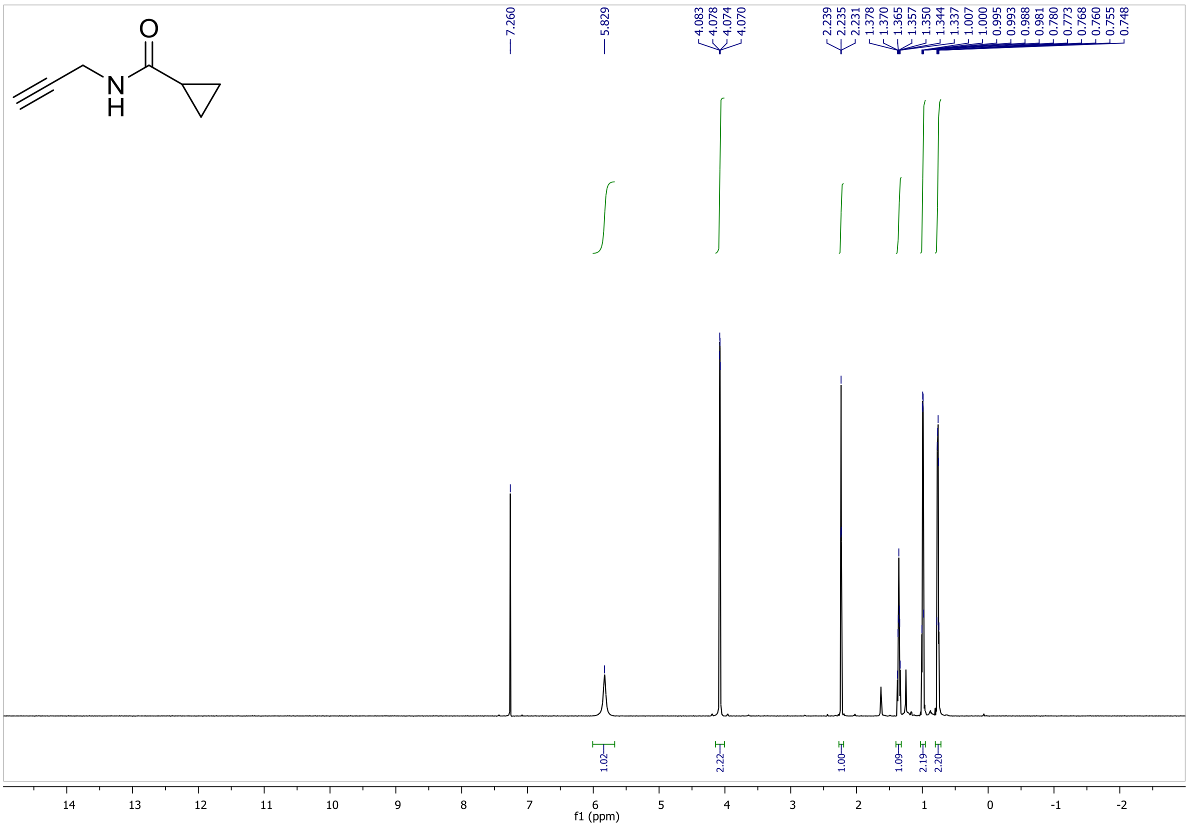

^{13}C NMR, 150 MHz, $CDCl_3$:

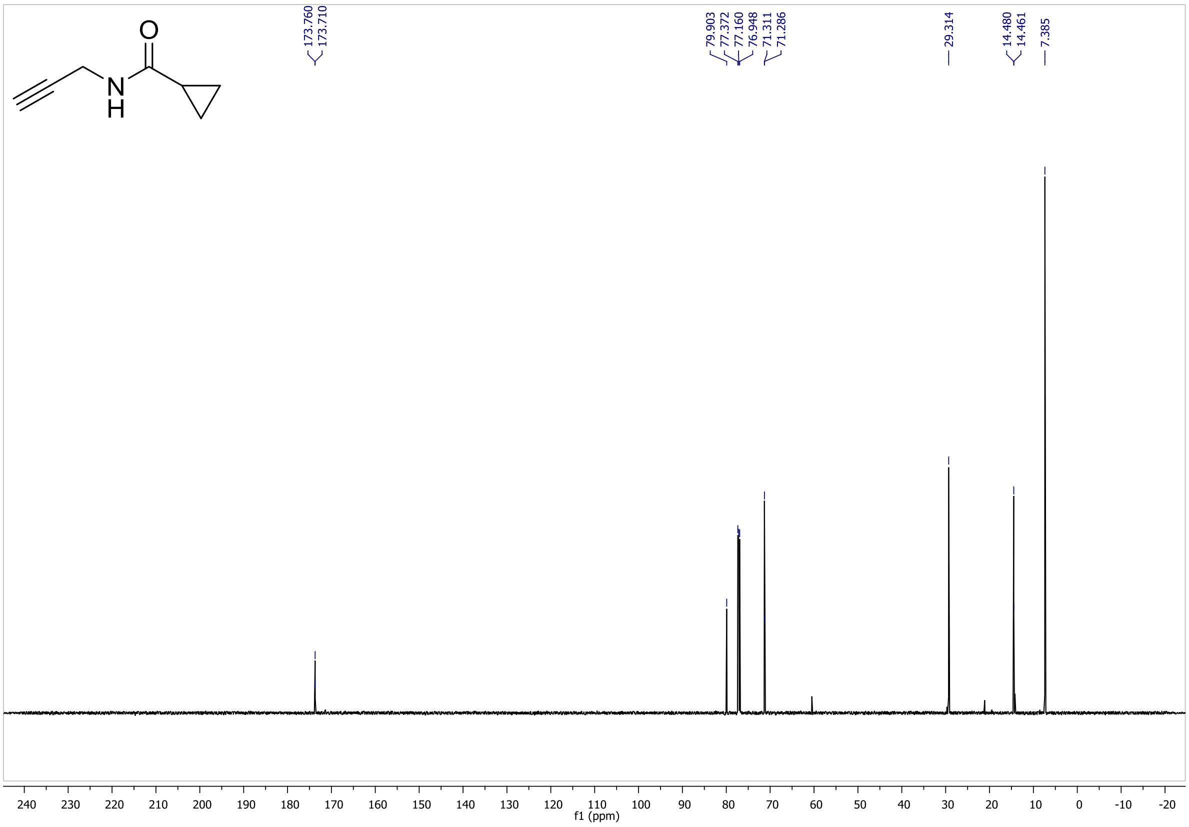

Methyl 4-oxo-4-(prop-2-yn-1-ylamino)butanoate:

^{1}H NMR, 600 MHz, $CDCl_3$:

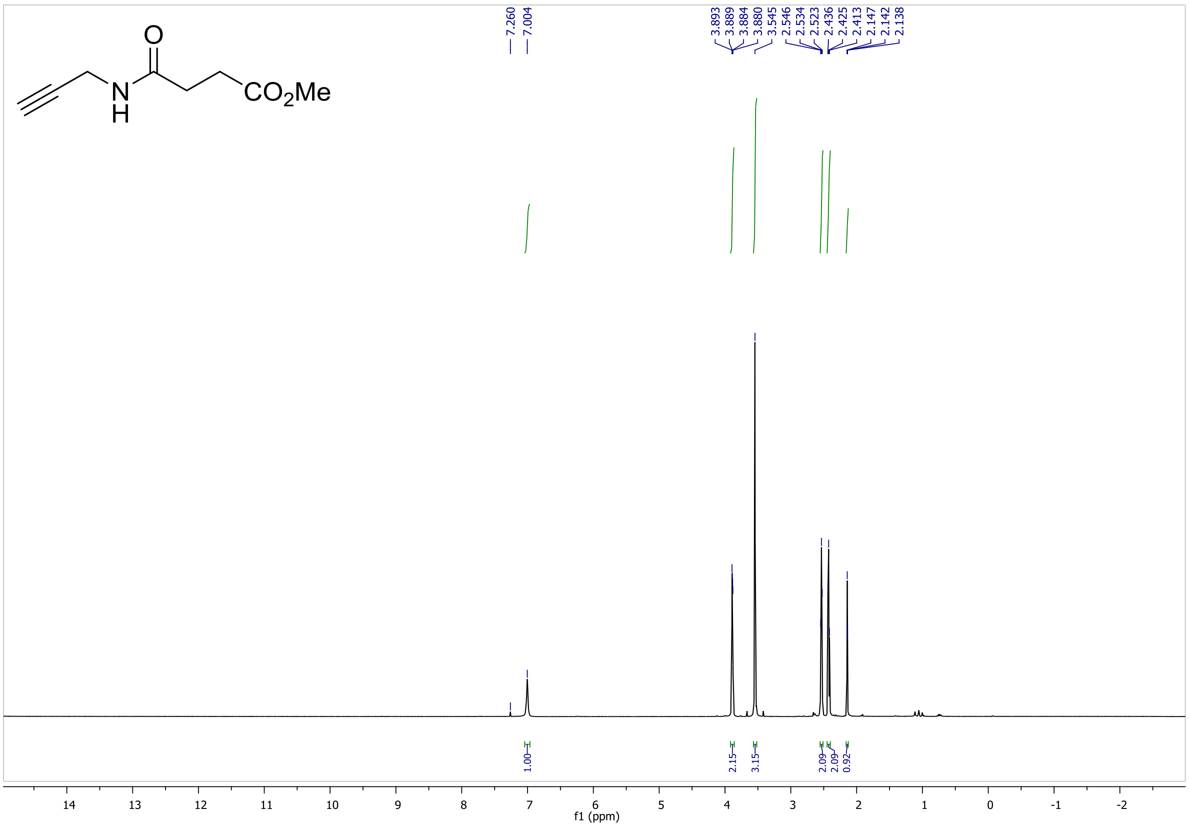

^{13}C NMR, 150 MHz, $CDCl_3$:

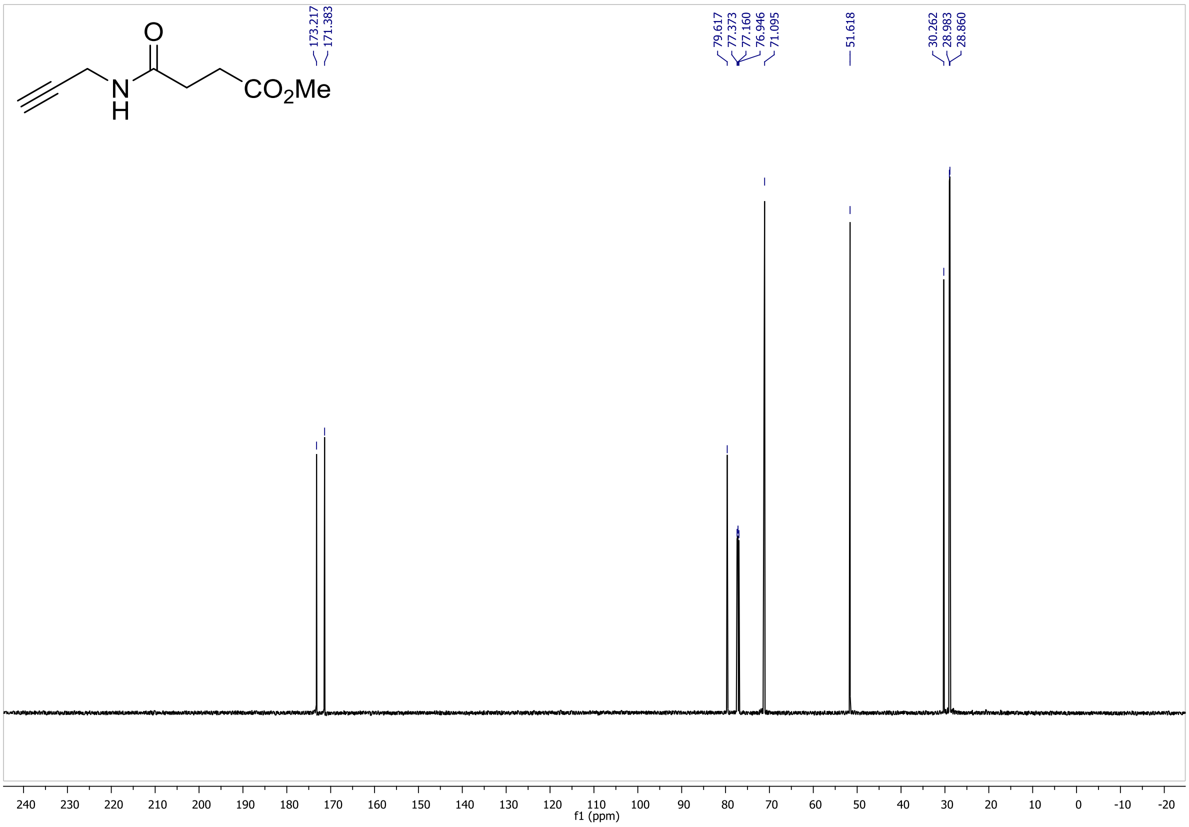

***N*-(prop-2-yn-1-yl)acetamide:**

^{1}H NMR, 600 MHz, $CDCl_3$:

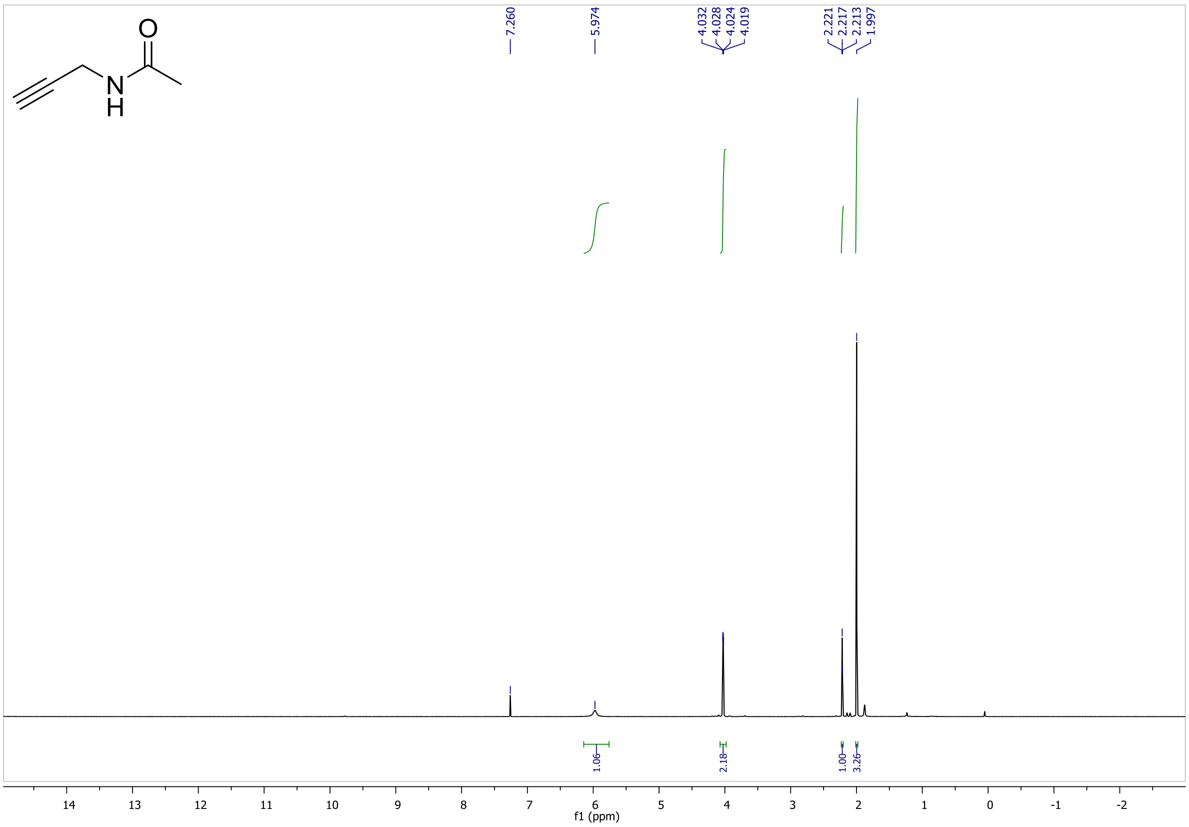

^{13}C NMR, 150 MHz, $CDCl_3$:

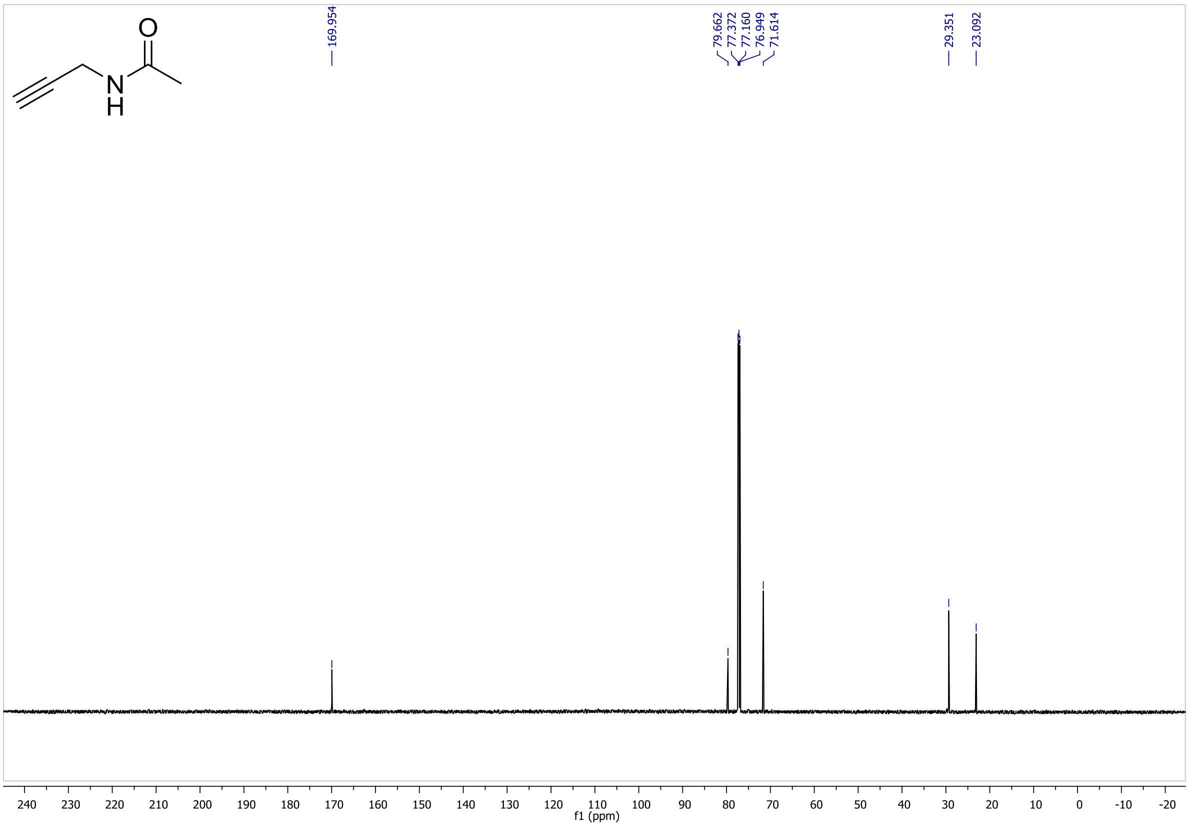

N-(prop-2-yn-1-yl)pent-4-enamide:

^{1}H NMR, 600 MHz, $CDCl_3$:

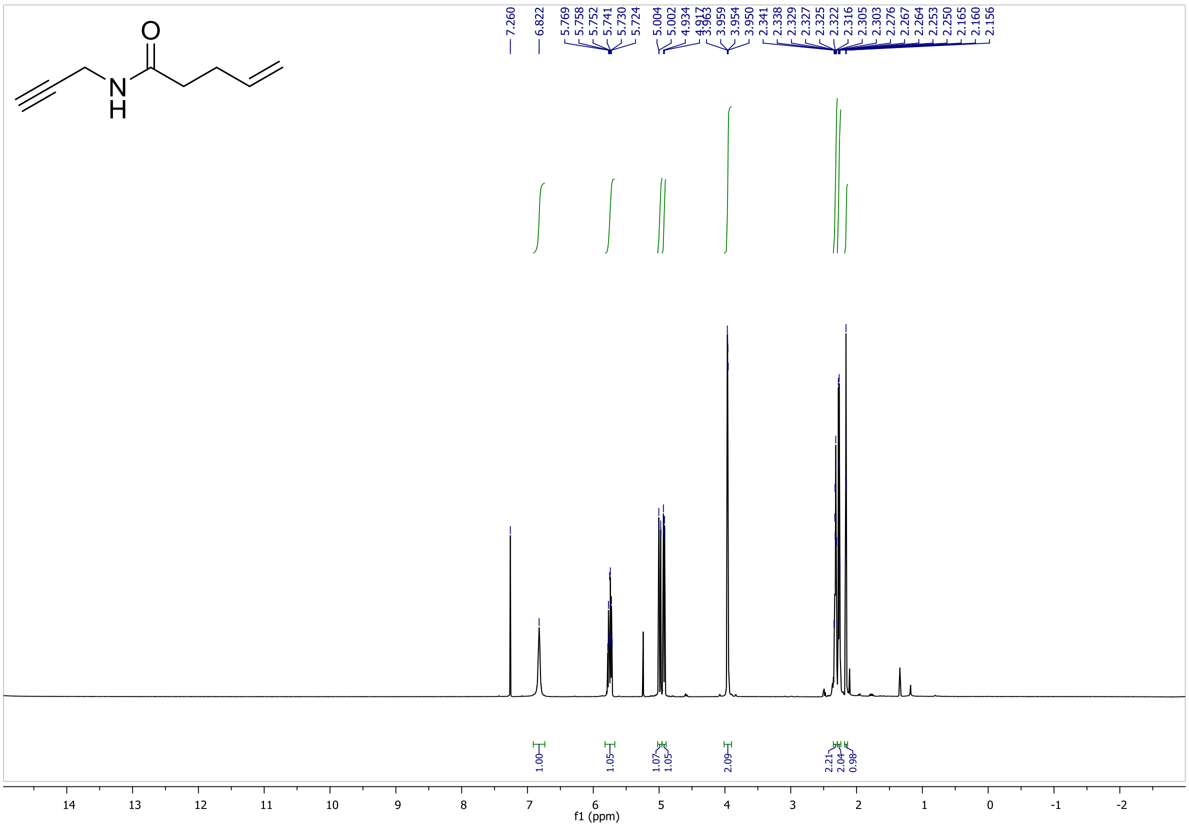

^{13}C NMR, 150 MHz, $CDCl_3$:

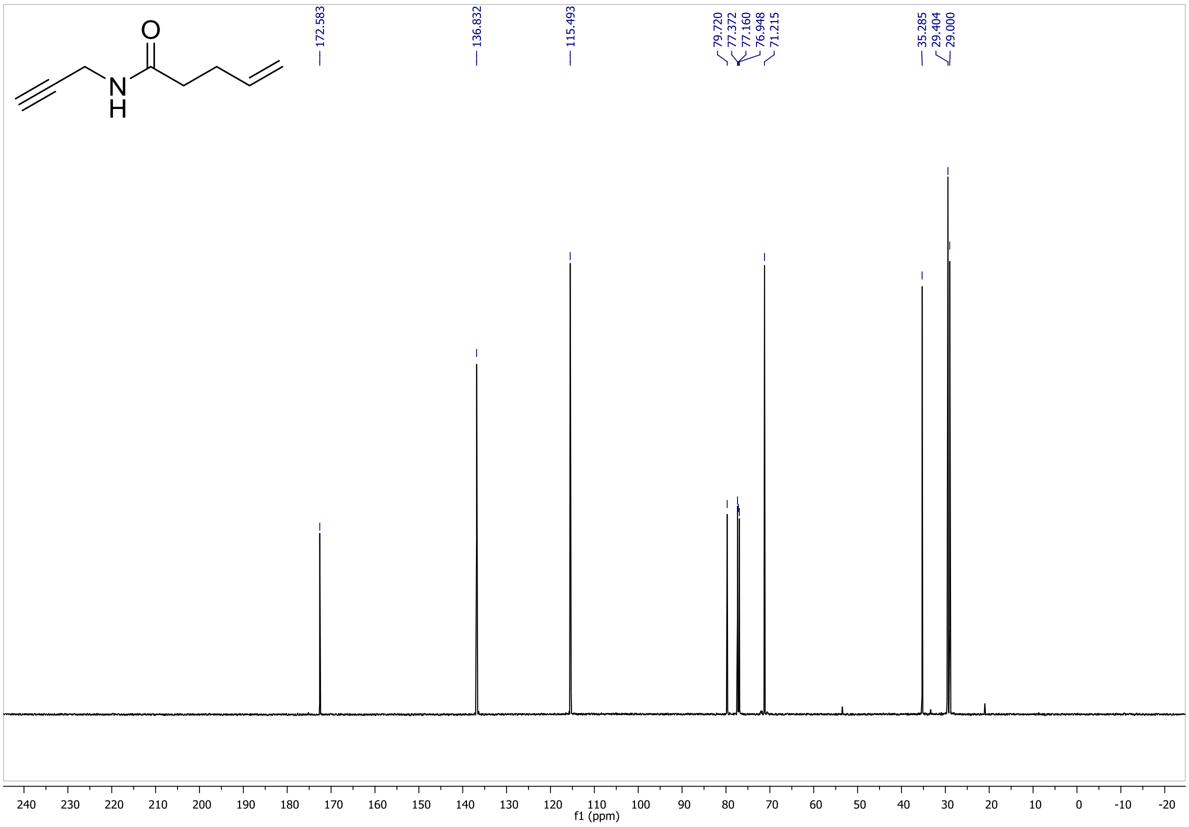

(*S*)-2-(4-isobutylphenyl)-*N*-(prop-2-yn-1-yl)propanamide:

^{1}H NMR, 600 MHz, CDCl$_3$:

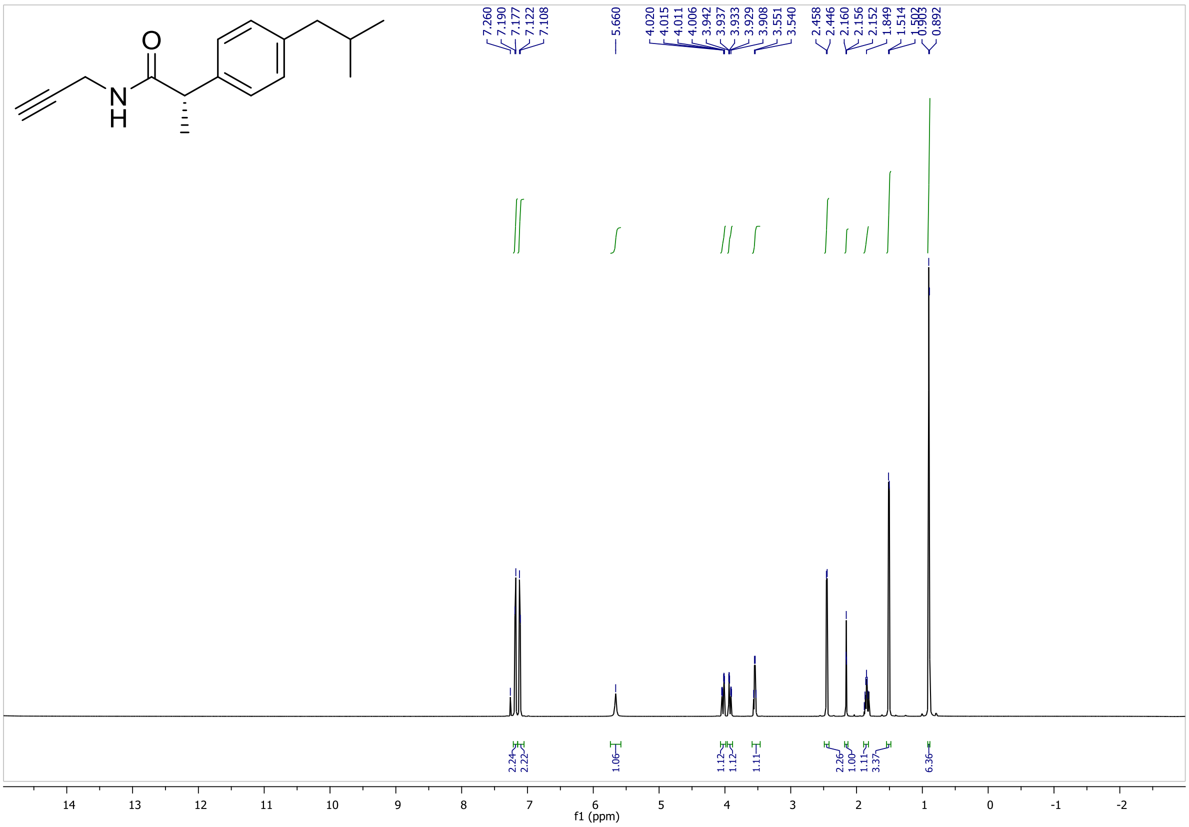

^{13}C NMR, 150 MHz, CDCl$_3$:

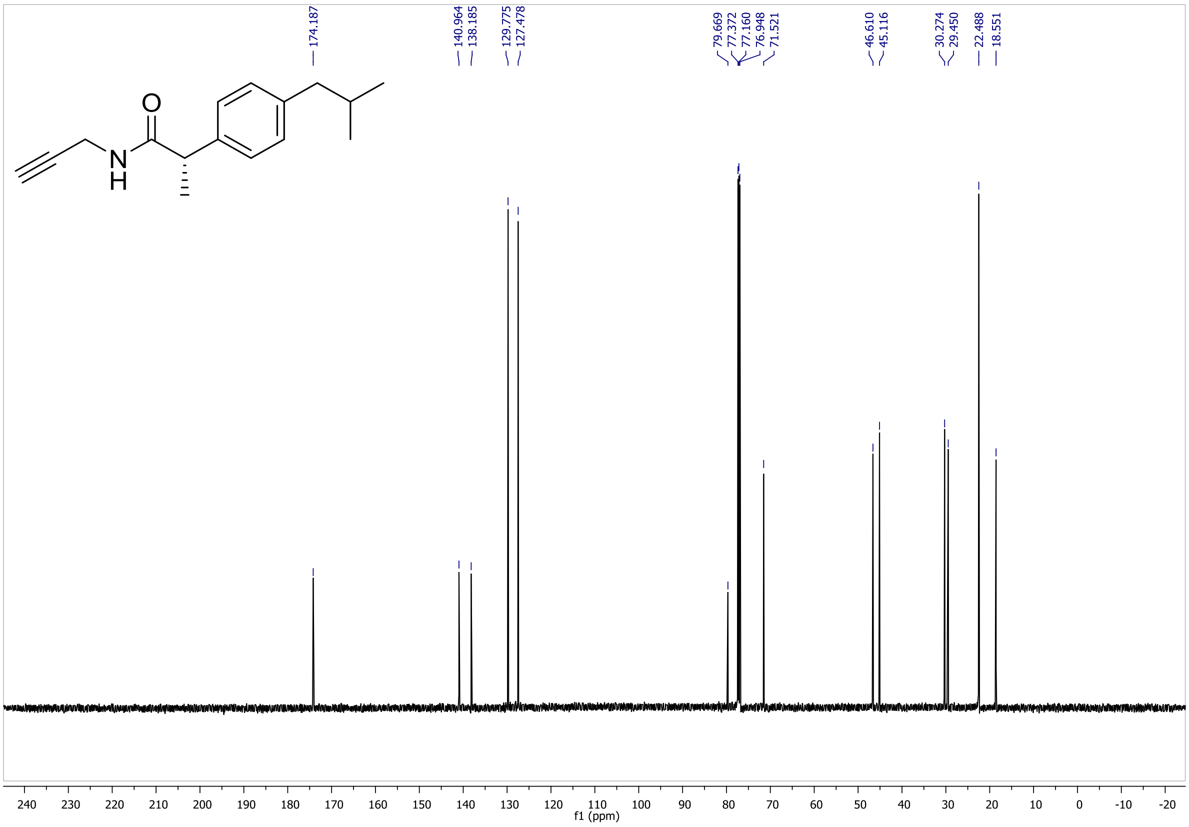

2-(prop-2-yn-1-ylcarbamoyl)phenyl acetate:

^{1}H NMR, 600 MHz, CDCl$_3$:

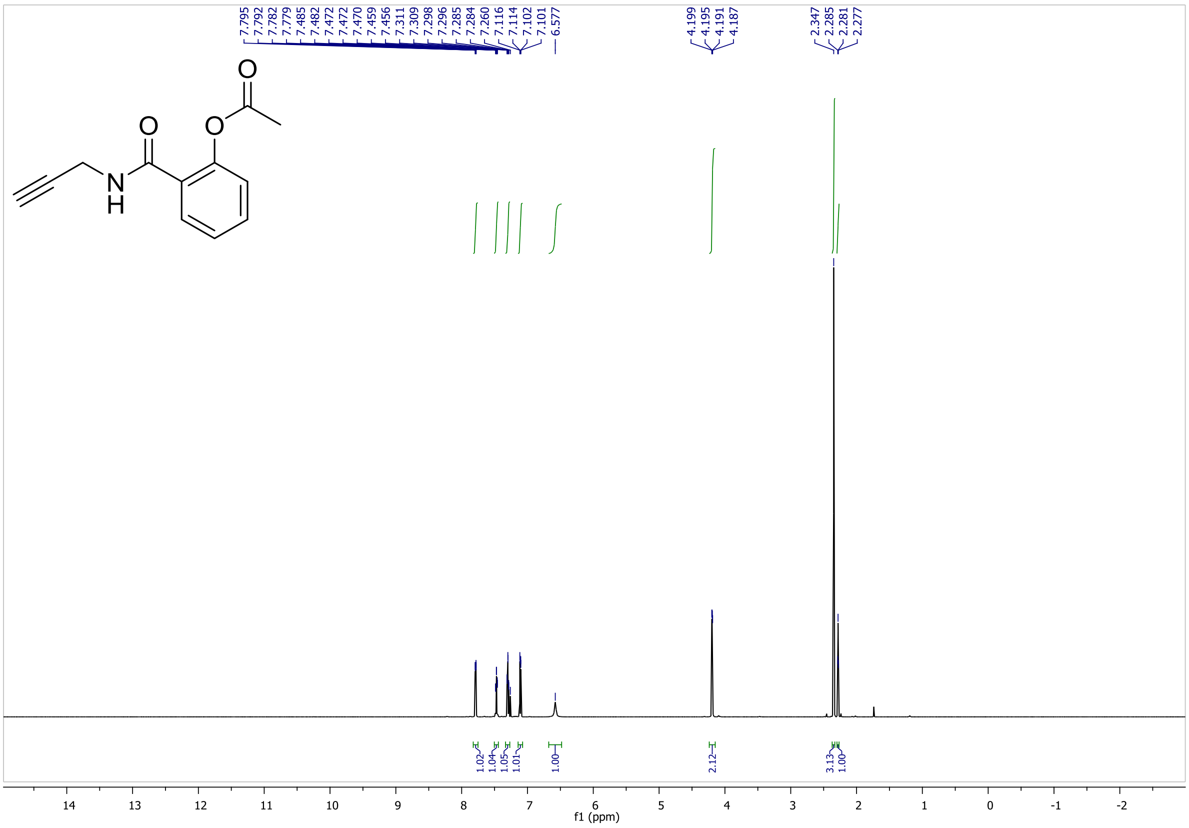

^{13}C NMR, 150 MHz, CDCl$_3$:

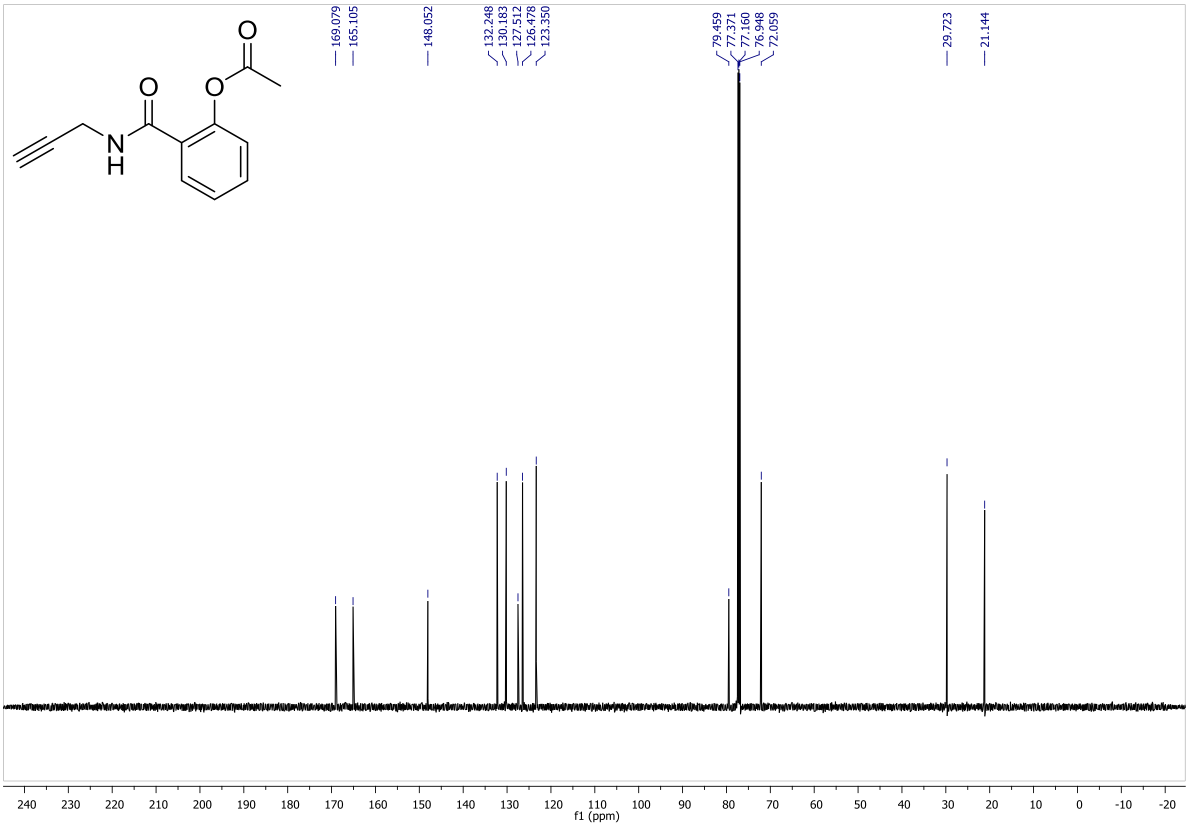

(2*S*,5*R*,6*R*)-3,3-dimethyl-7-oxo-6-(2-phenylacetamido)-*N*-(prop-2-yn-1-yl)-4-thia-1-azabicyclo[3.2.0]heptane-2-carboxamide:

^{1}H NMR, 600 MHz, CDCl$_3$:

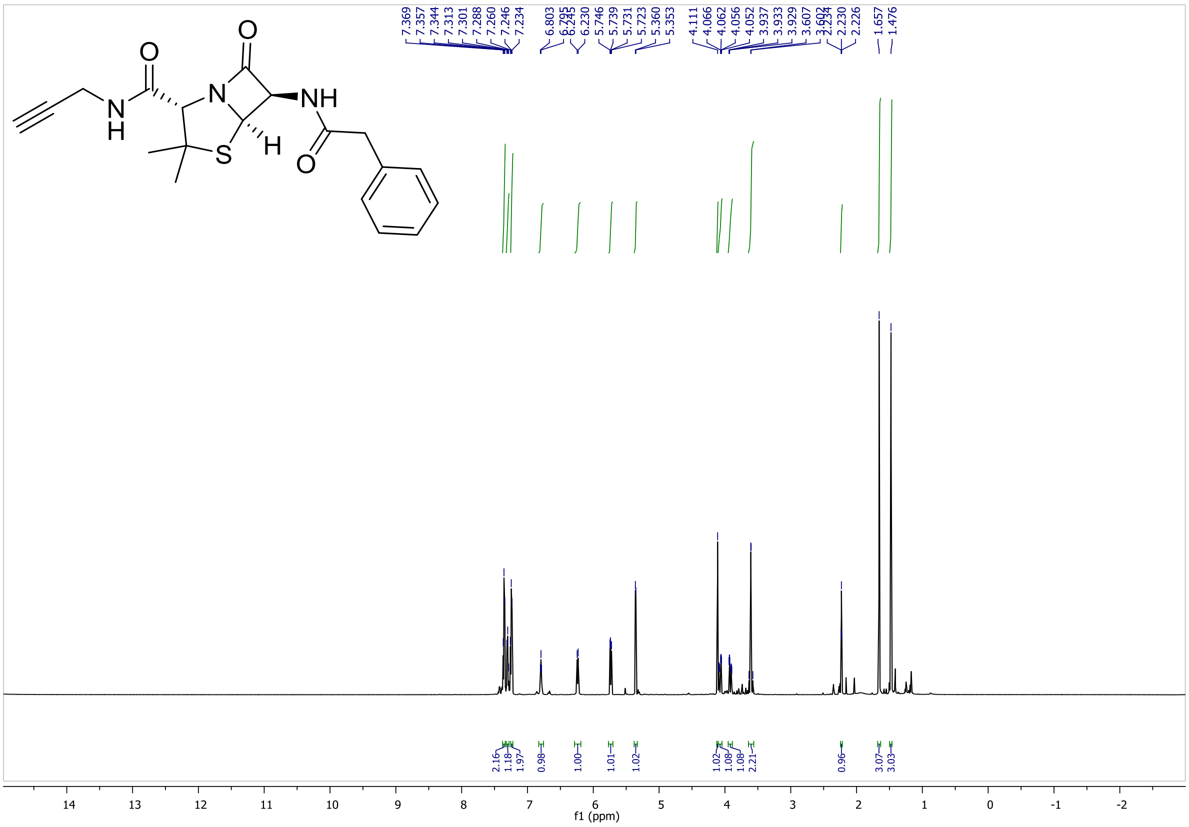

^{13}C NMR, 150 MHz, CDCl$_3$:

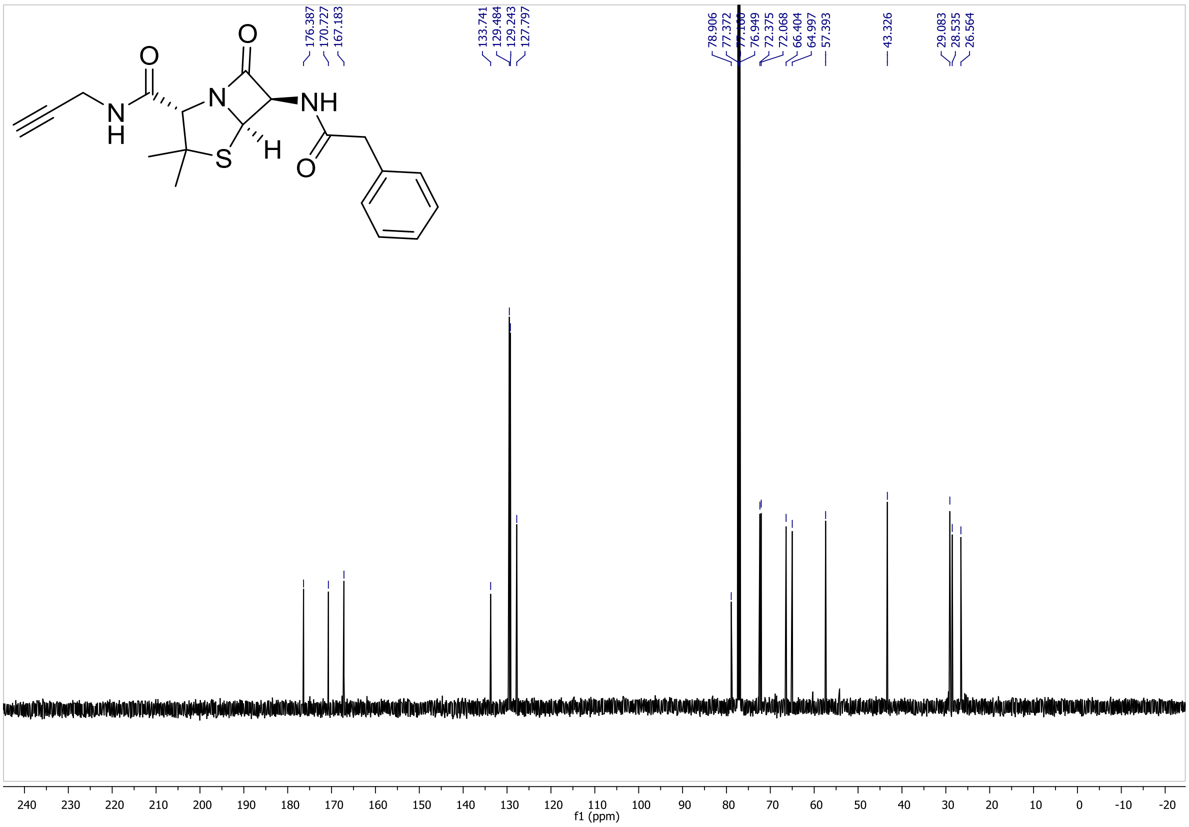

((3a*S*,5a*R*,8a*R*,8b*S*)-2,2,7,7-tetramethyltetrahydro-3a*H*-bis([1,3]dioxolo)[4,5-*b*:4',5'-*d*]pyran-3a-yl)methyl prop-2-yn-1-ylsulfamate:

^{1}H NMR, 600 MHz, CDCl$_3$:

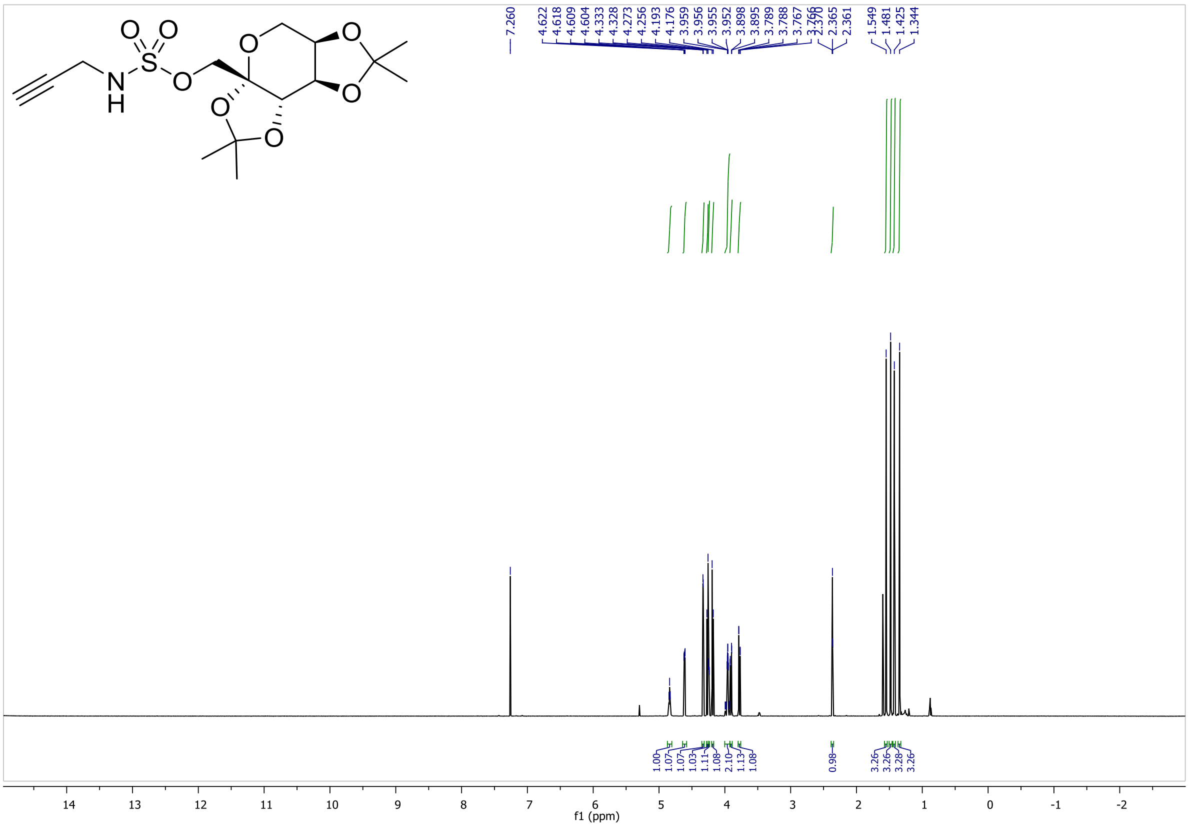

^{13}C NMR, 150 MHz, CDCl$_3$:

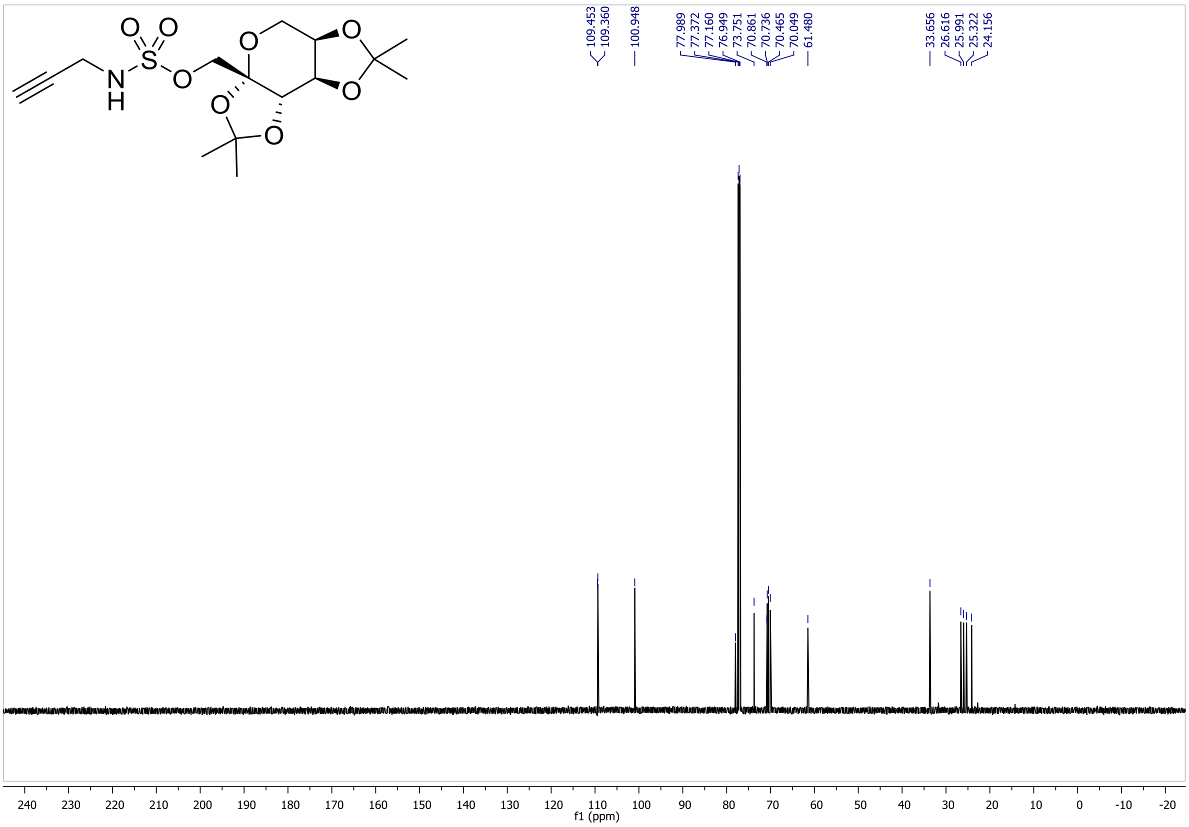

***tert*-butyl (*S*)-(4-methyl-2-(2-oxo-2-(prop-2-yn-1-ylamino)ethyl)pentyl)carbamate:**

^{1}H NMR, 600 MHz, $CDCl_3$:

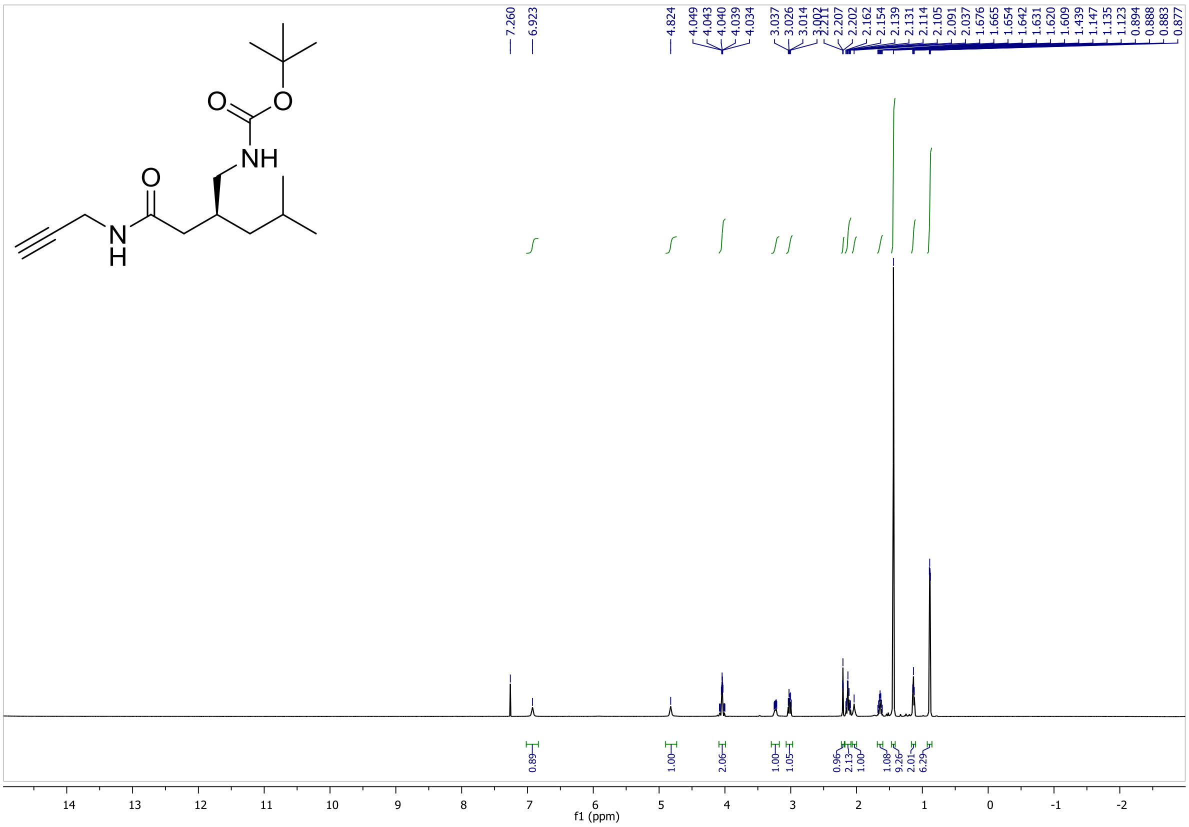

^{13}C NMR, 150 MHz, $CDCl_3$:

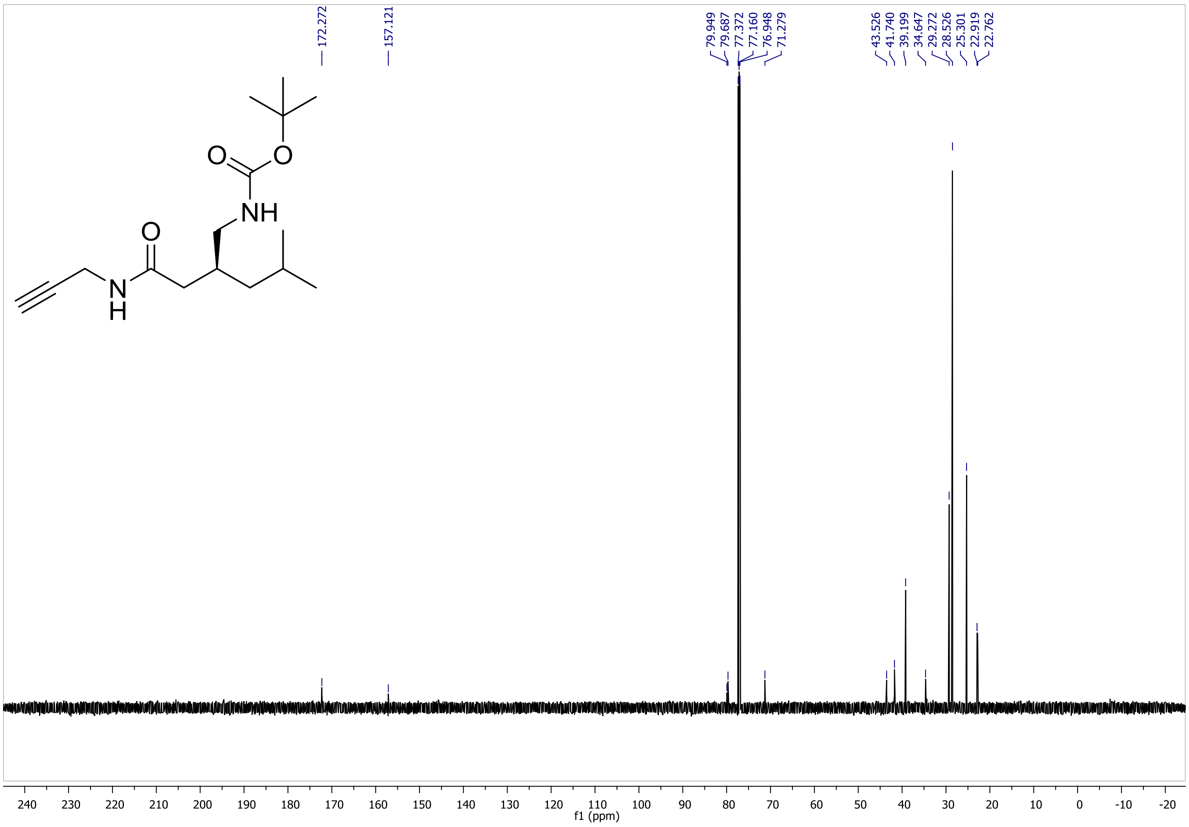

2-(1-(4-chlorobenzoyl)-5-methoxy-2-methyl-1*H*-indol-3-yl)-*N*-(prop-2-yn-1-yl)acetamide:

^{1}H NMR, 600 MHz, $CDCl_3$:

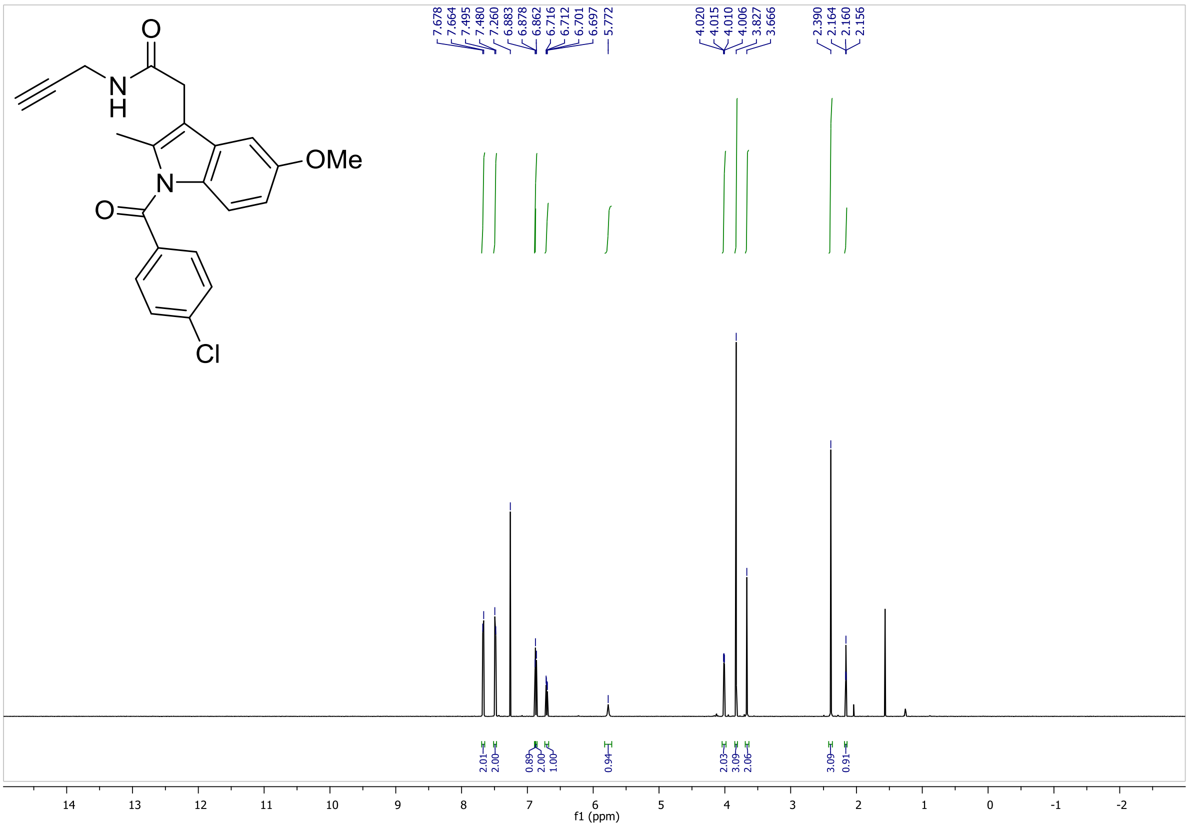

^{13}C NMR, 150 MHz, $CDCl_3$:

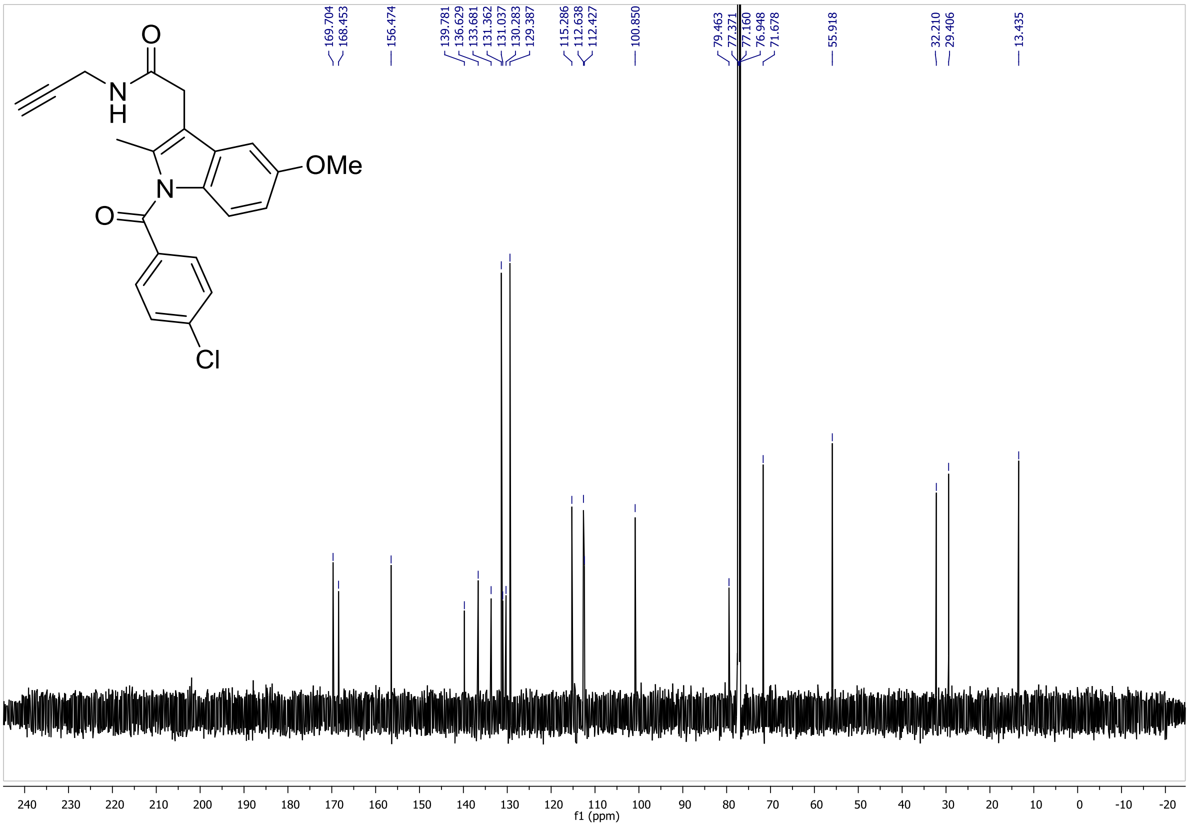

1-(2-((4*R*,6*R*)-2,2-dimethyl-6-(2-oxo-2-(prop-2-yn-1-ylamino)ethyl)-1,3-dioxan-4-yl)ethyl)-5-(4-fluorophenyl)-2-isopropyl-*N*,4-diphenyl-1*H*-pyrrole-3-carboxamide:

^{1}H NMR, 600 MHz, $CDCl_3$:

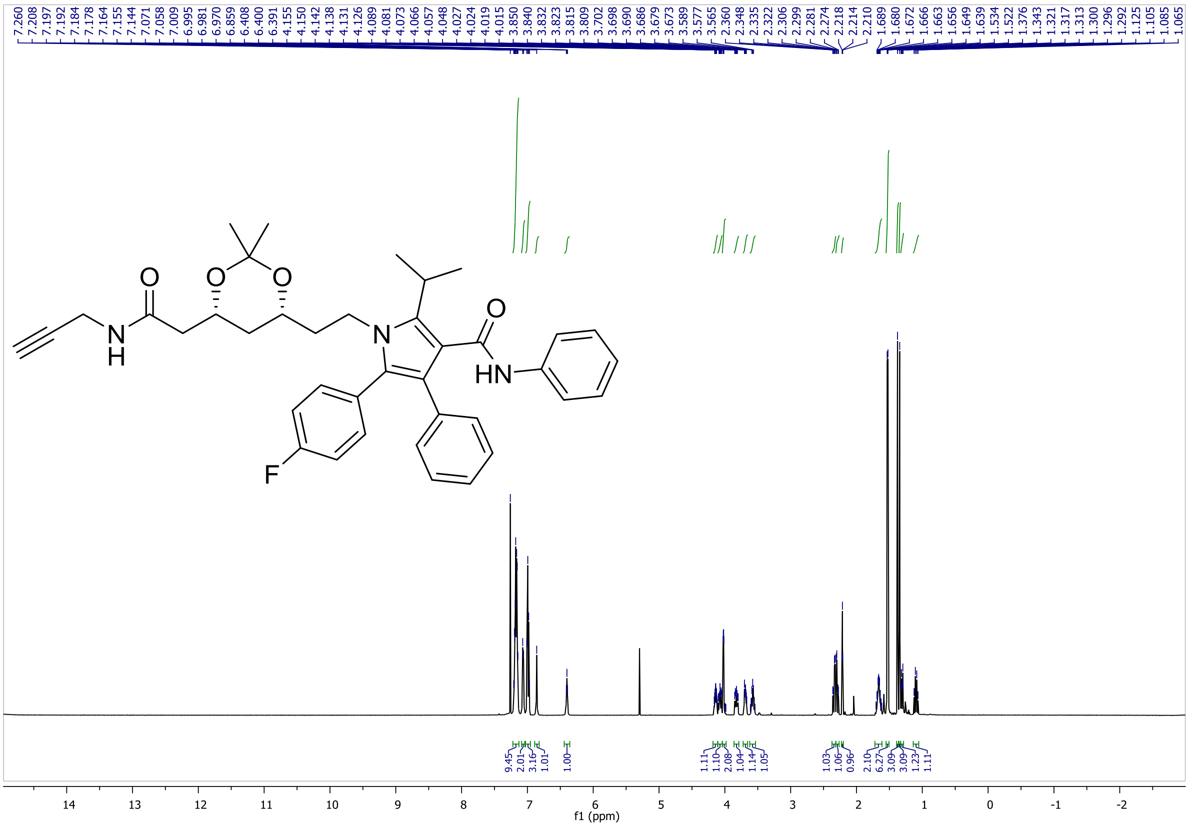

^{13}C NMR, 150 MHz, $CDCl_3$:

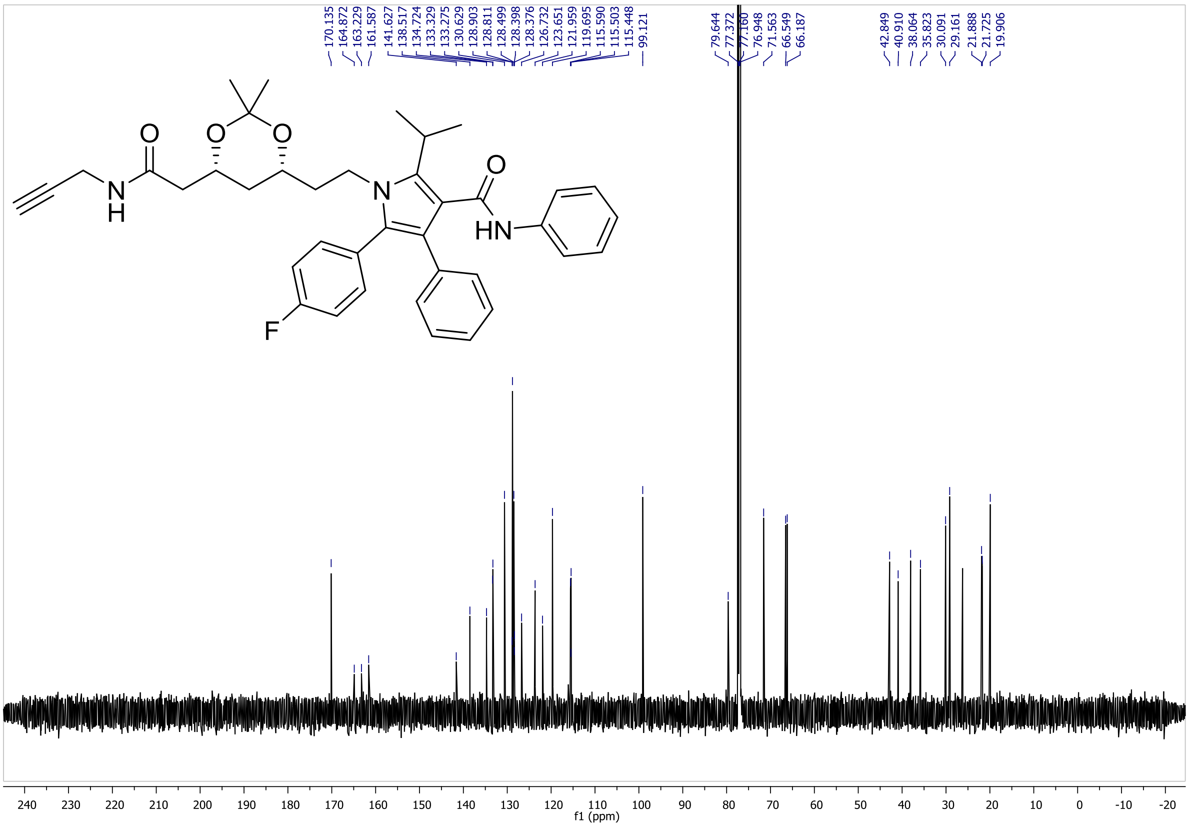

^{19}F NMR, 376 MHz, $CDCl_3$:

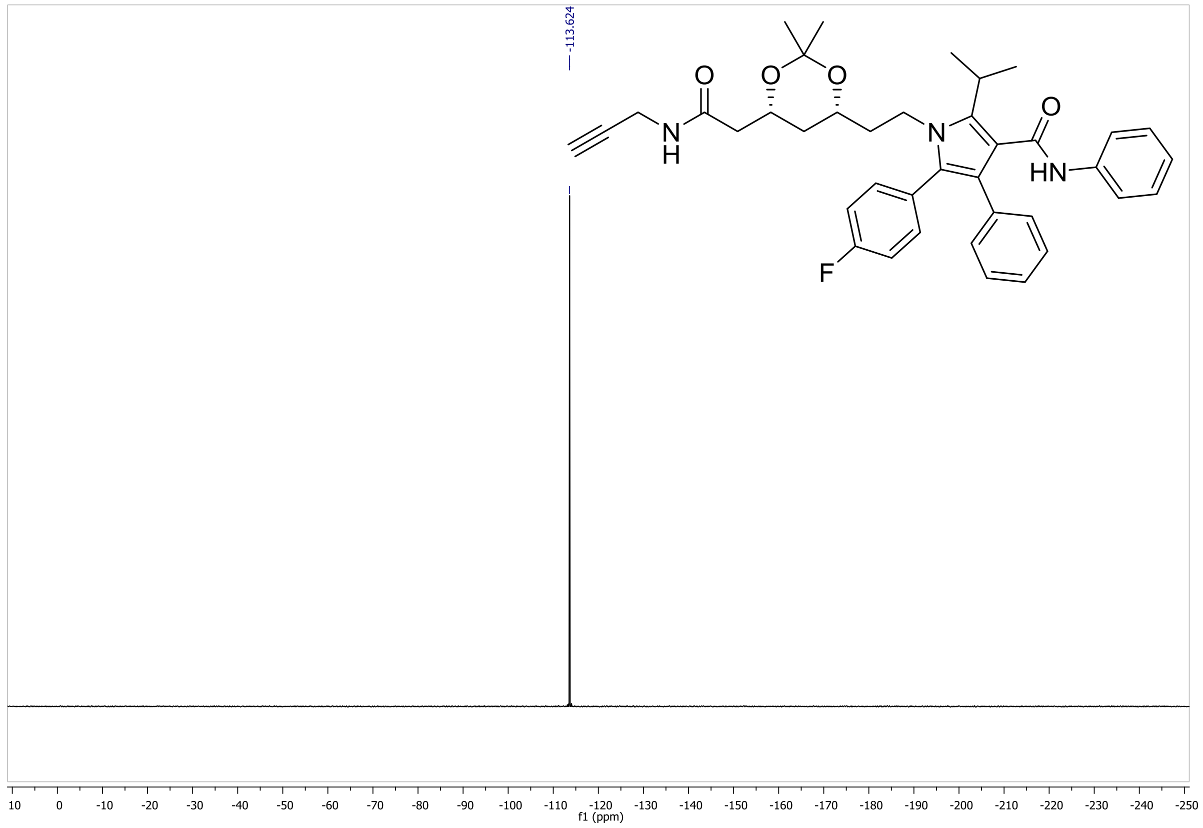

8.4. NMR spectra for hydrazone starting materials

(4-chlorobenzylidene)hydrazine (1a):

^{1}H NMR, 600 MHz, $CDCl_3$:

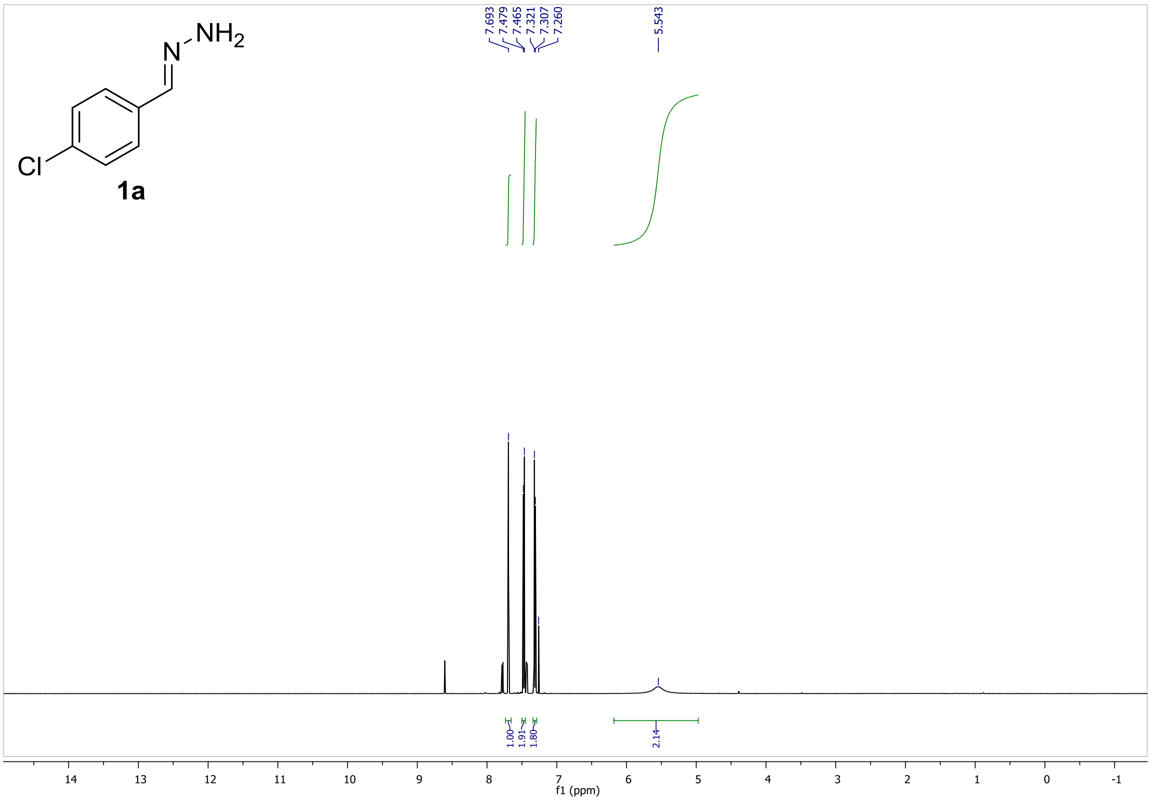

^{13}C NMR, 150 MHz, $CDCl_3$:

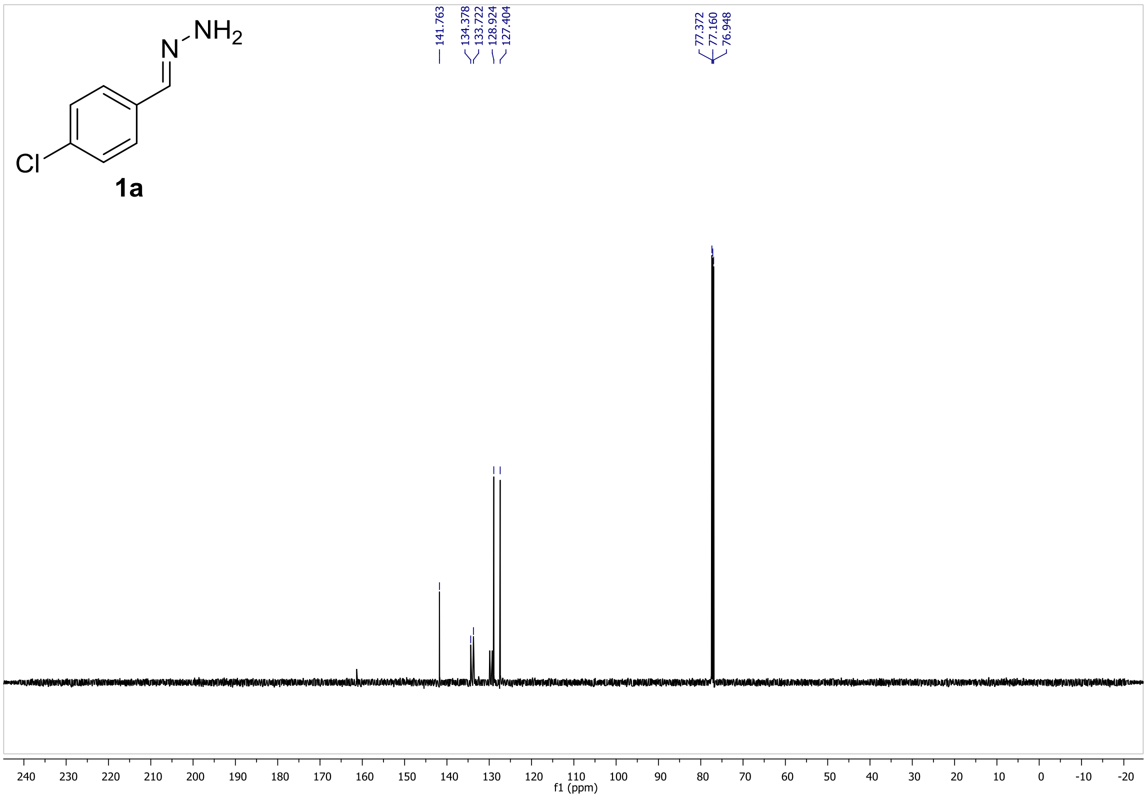

(4-bromobenzylidene)hydrazine:

^{1}H NMR, 600 MHz, CDCl$_3$:

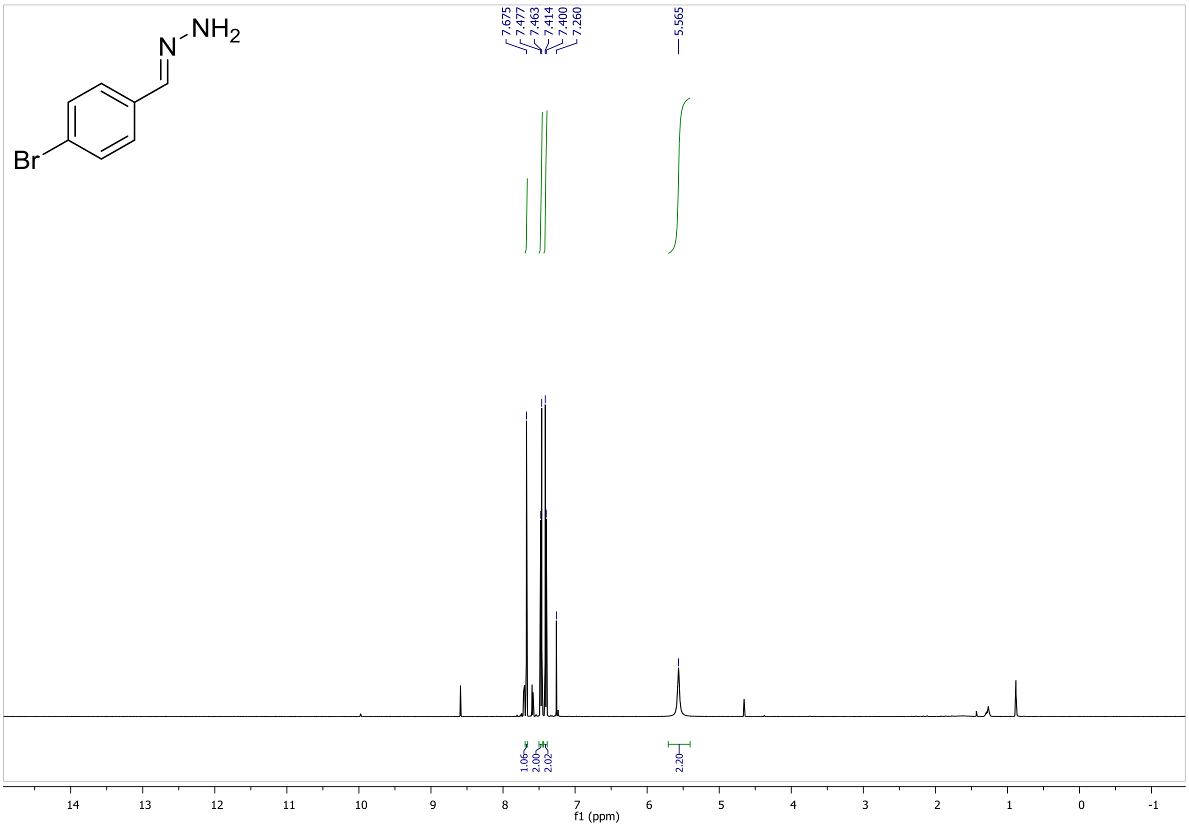

^{13}C NMR, 150 MHz, CDCl$_3$:

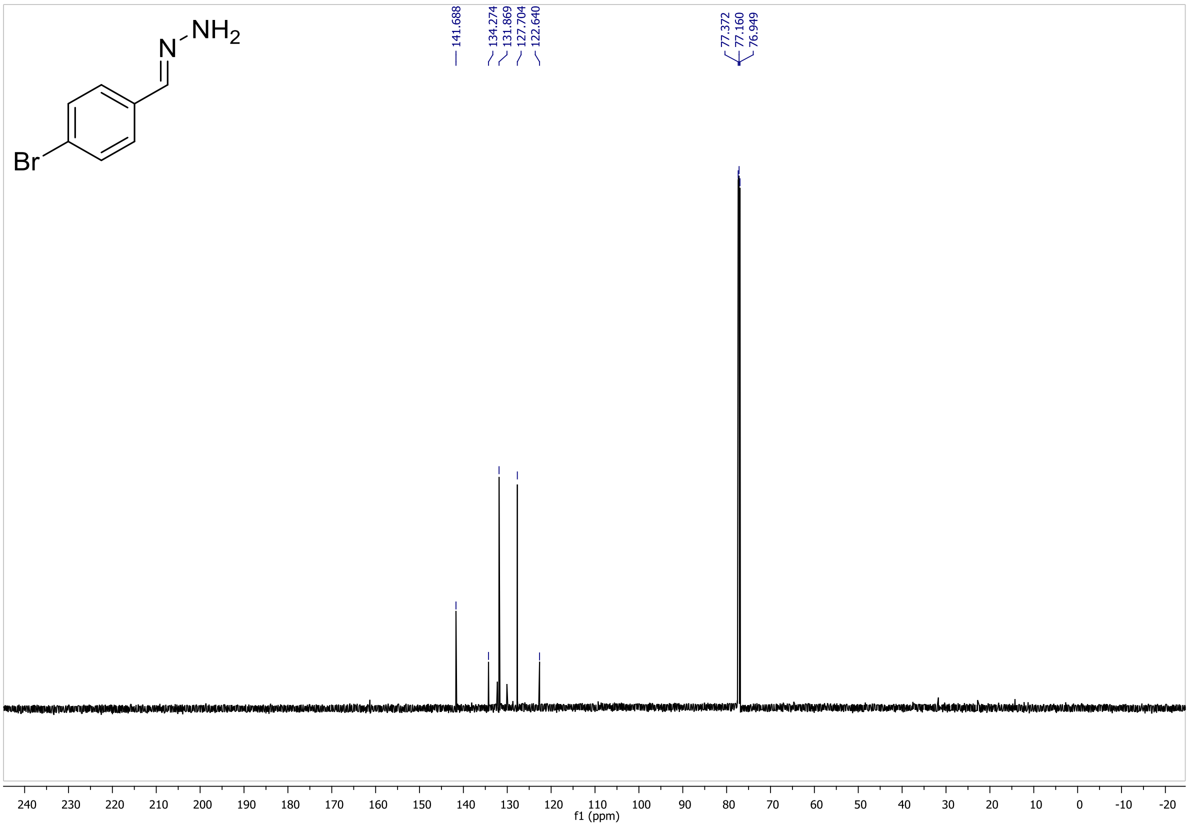

(4-fluorobenzylidene)hydrazine:

^{1}H NMR, 600 MHz, CDCl$_3$:

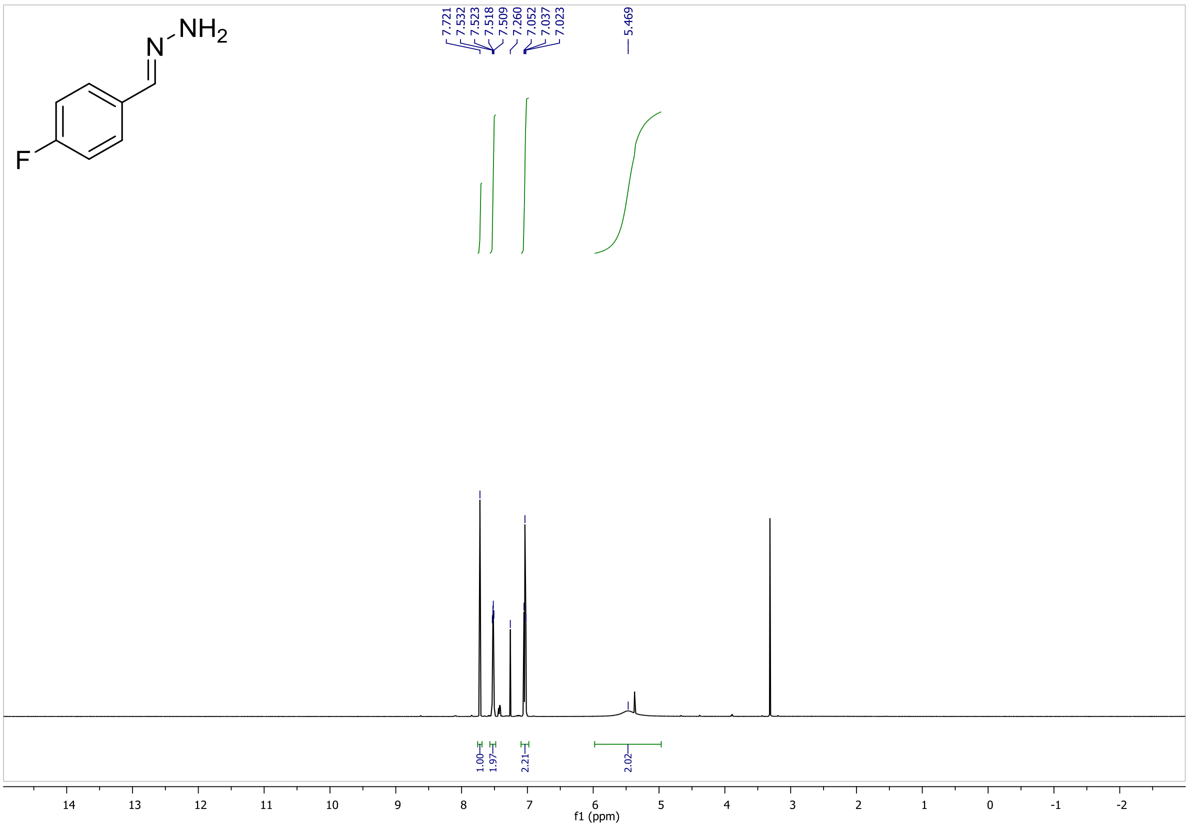

^{13}C NMR, 150 MHz, CDCl$_3$:

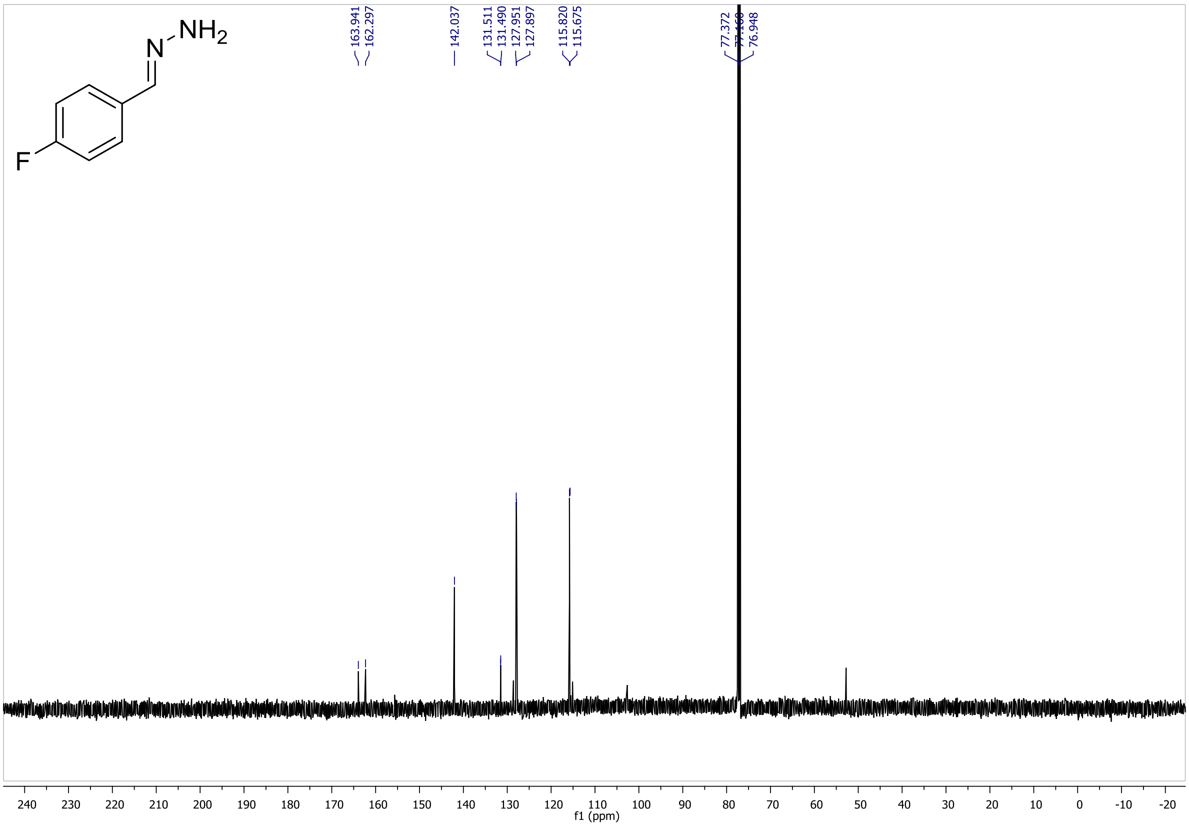

^{19}F NMR, 376 MHz, $CDCl_3$:

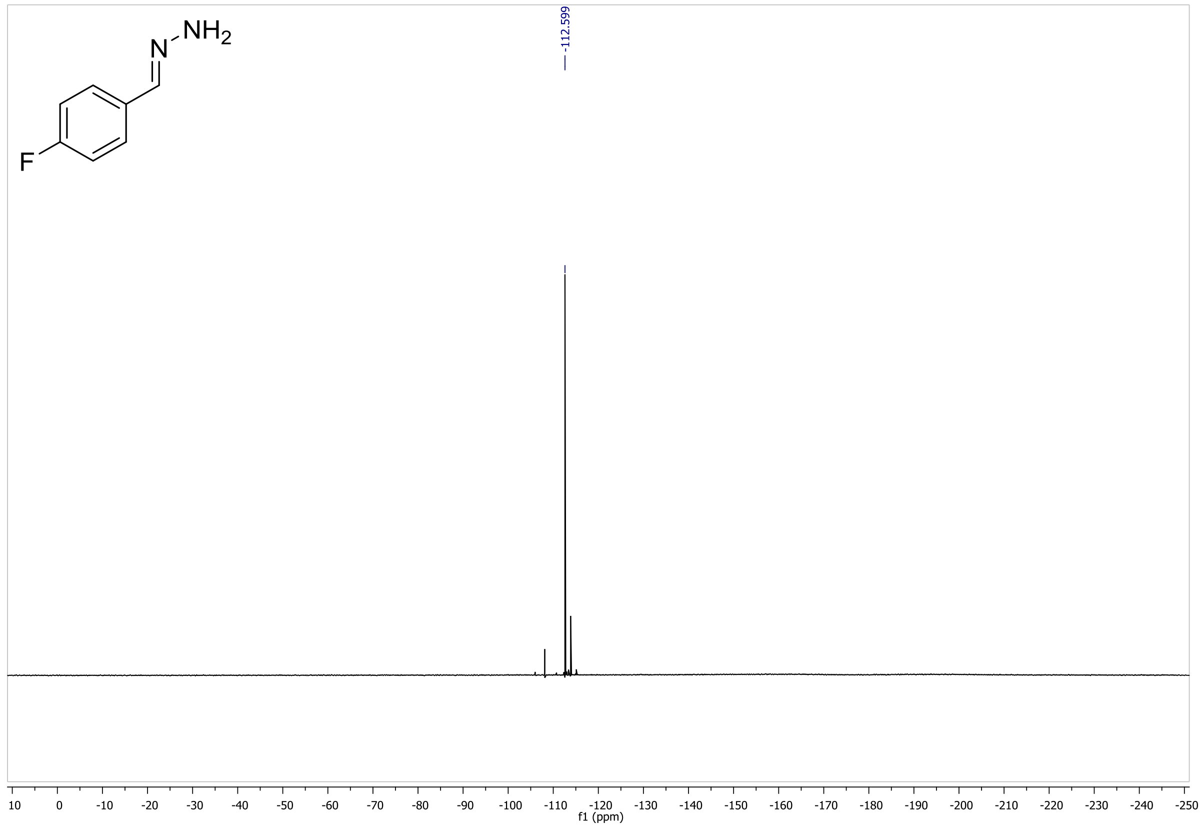

(3-methoxybenzylidene)hydrazine:

^{1}H NMR, 600 MHz, $CDCl_3$:

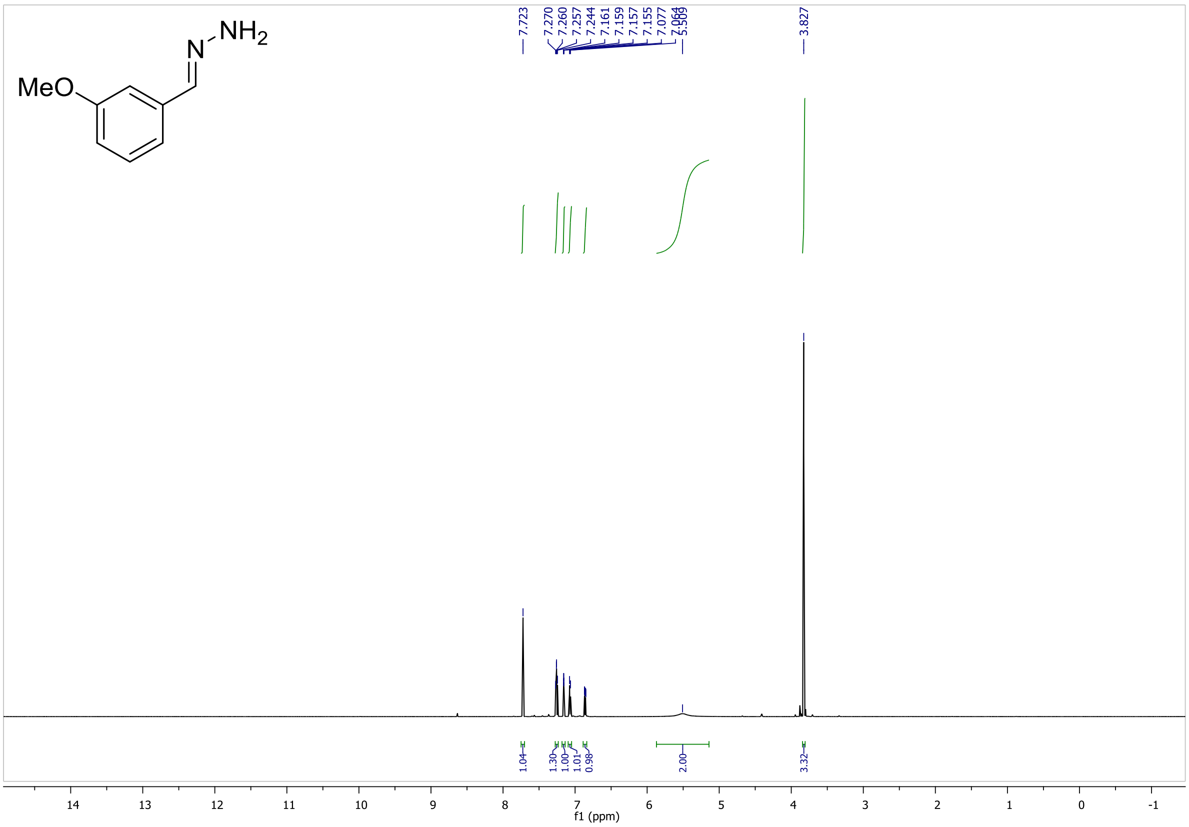

^{13}C NMR, 150 MHz, $CDCl_3$:

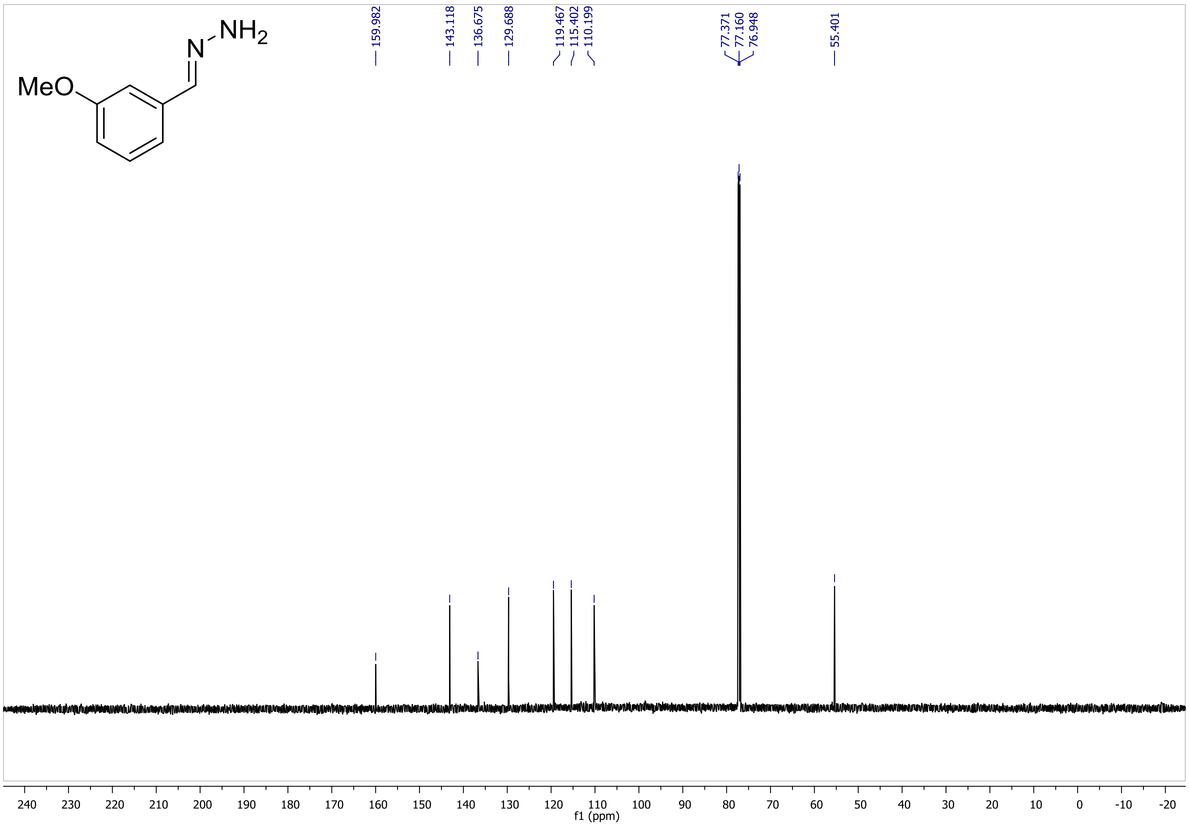

3-(hydrazonomethyl)benzonitrile:

^{1}H NMR, 600 MHz, $CDCl_3$:

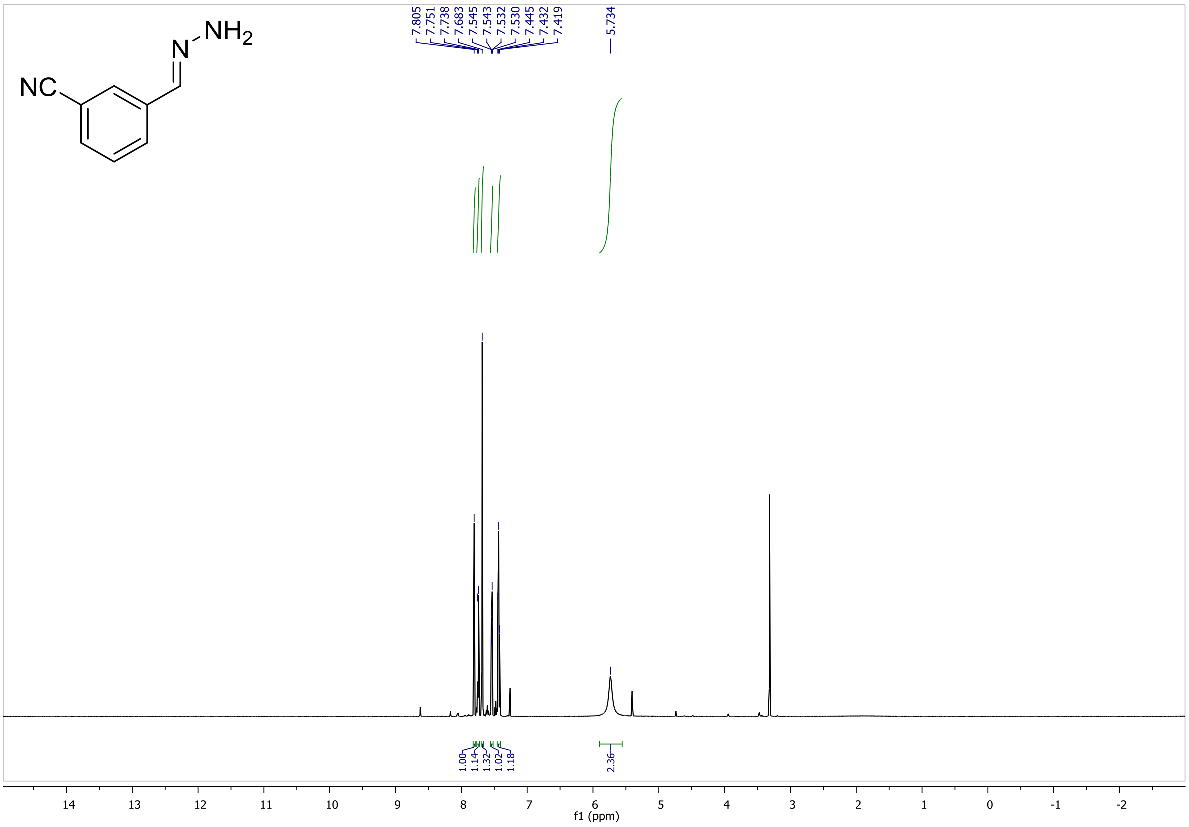

^{13}C NMR, 150 MHz, $CDCl_3$:

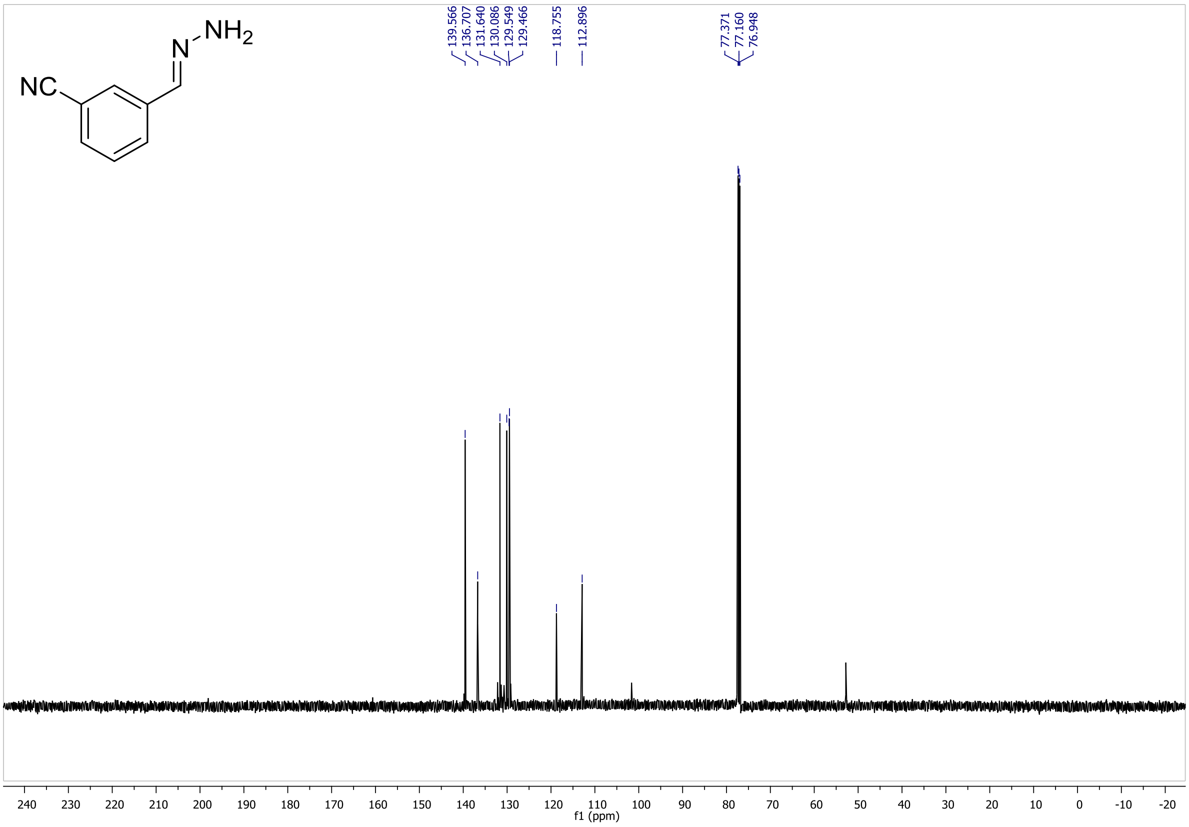

(4-iodobenzylidene)hydrazine:

^{1}H NMR, 600 MHz, $CDCl_3$:

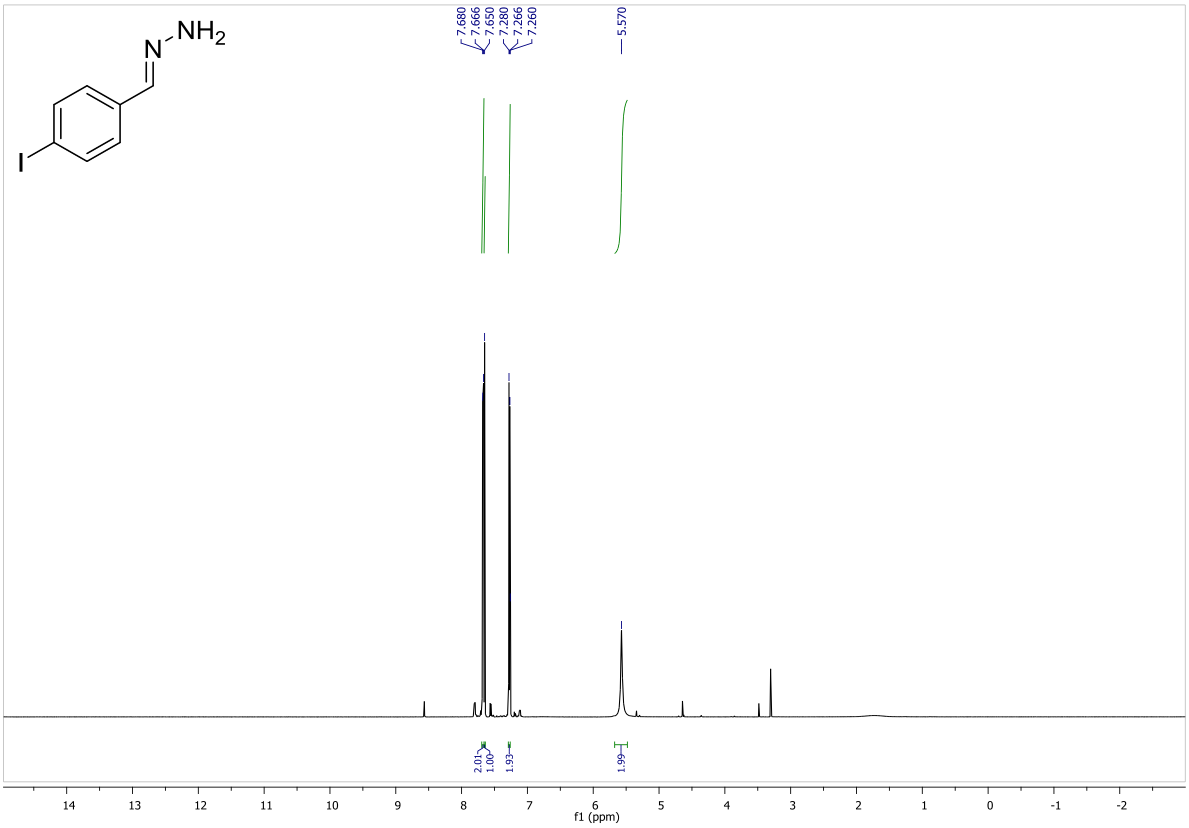

^{13}C NMR, 150 MHz, $CDCl_3$:

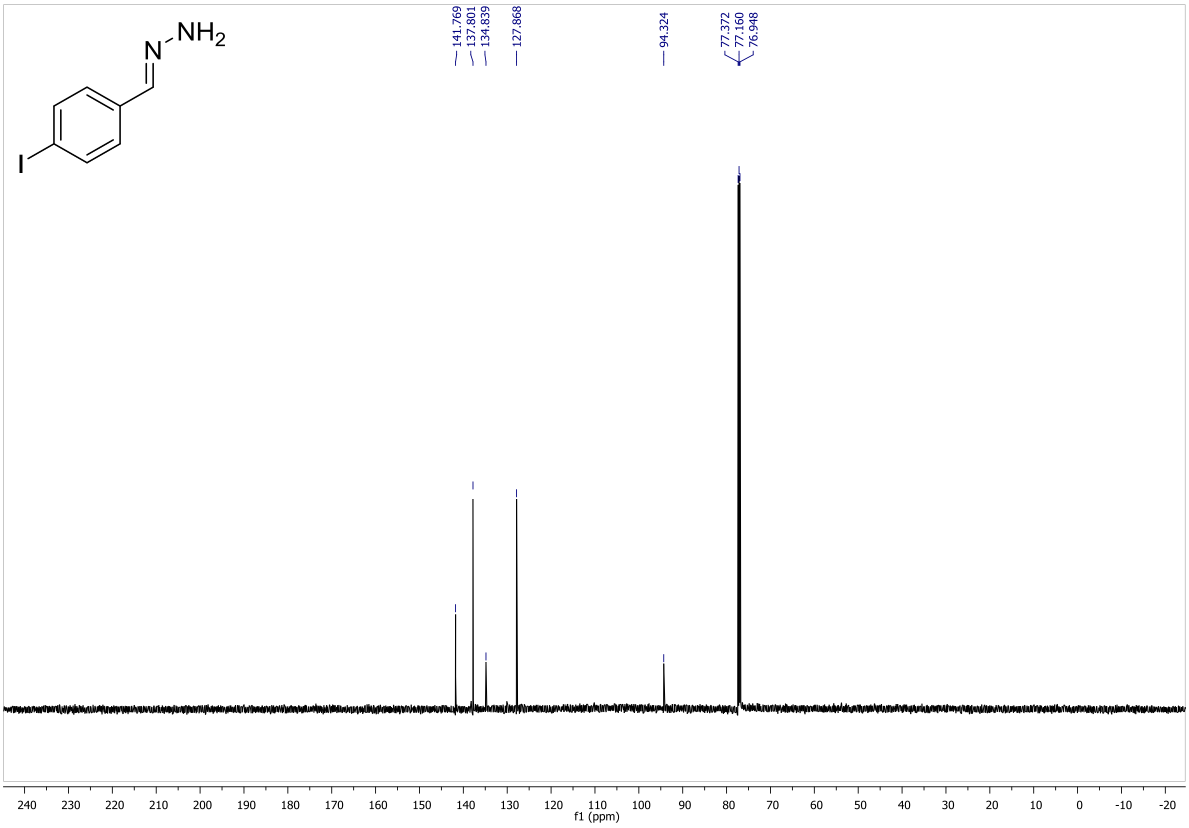

8.5. NMR spectra for chiral allenes

(*R*)-*N*-(4-(4-chlorophenyl)buta-2,3-dien-1-yl)-4-methylbenzenesulfonamide (4a):

^{1}H NMR, 600 MHz, $CDCl_3$:

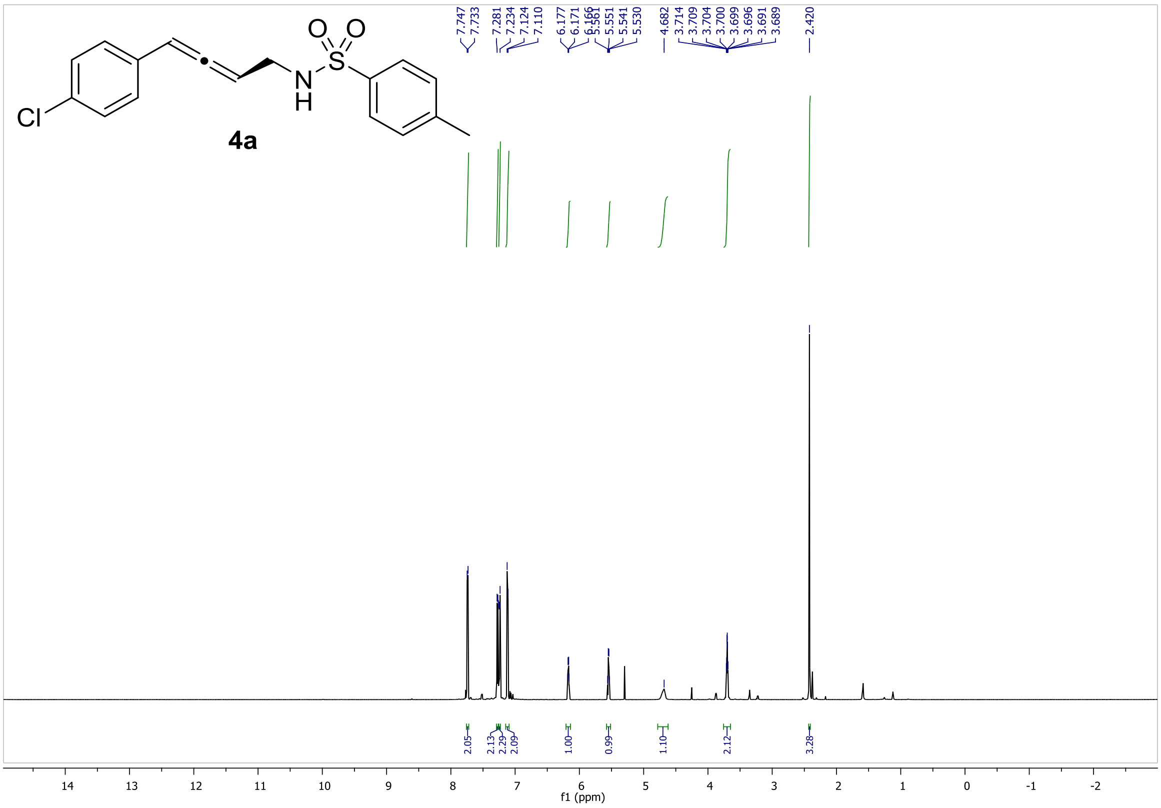

^{13}C NMR, 150 MHz, $CDCl_3$:

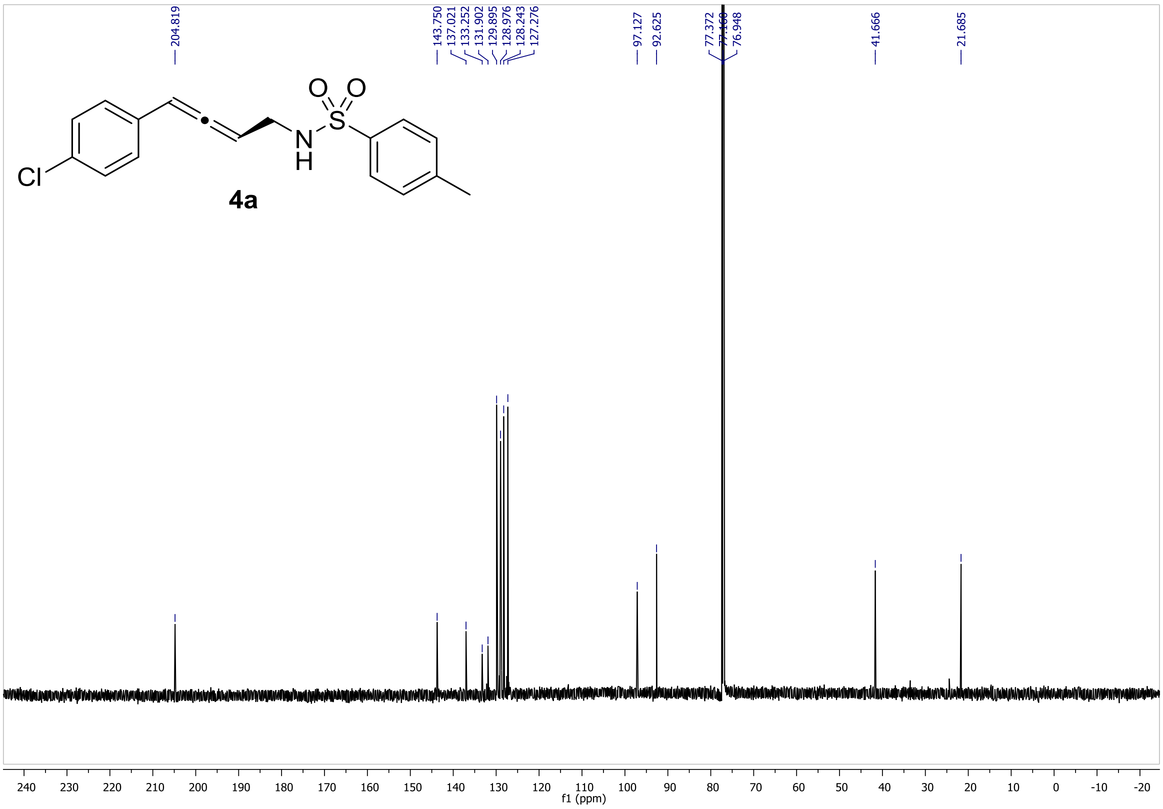

(*R*)-*N*-(4-(4-chlorophenyl)buta-2,3-dien-1-yl)-3,3,3-trifluoropropanamide (4b):

^{1}H NMR, 600 MHz, $CDCl_3$:

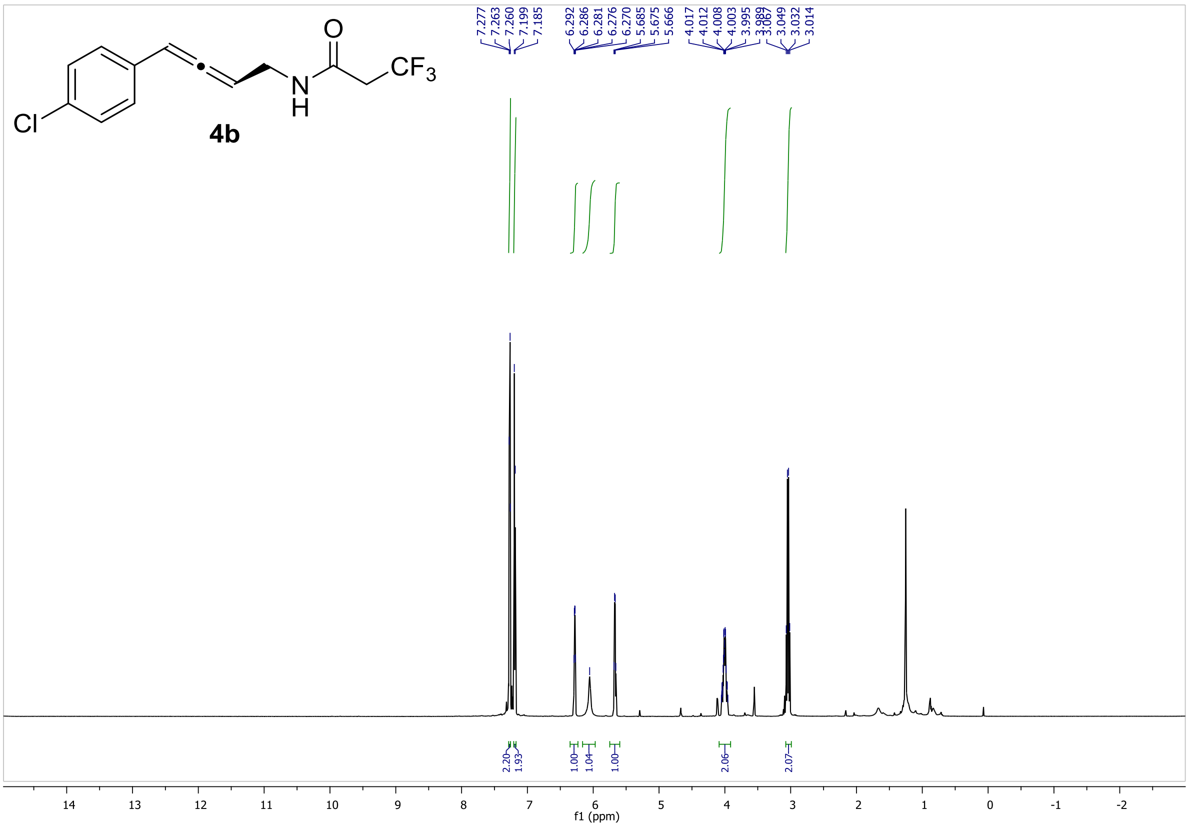

^{13}C NMR, 150 MHz, $CDCl_3$:

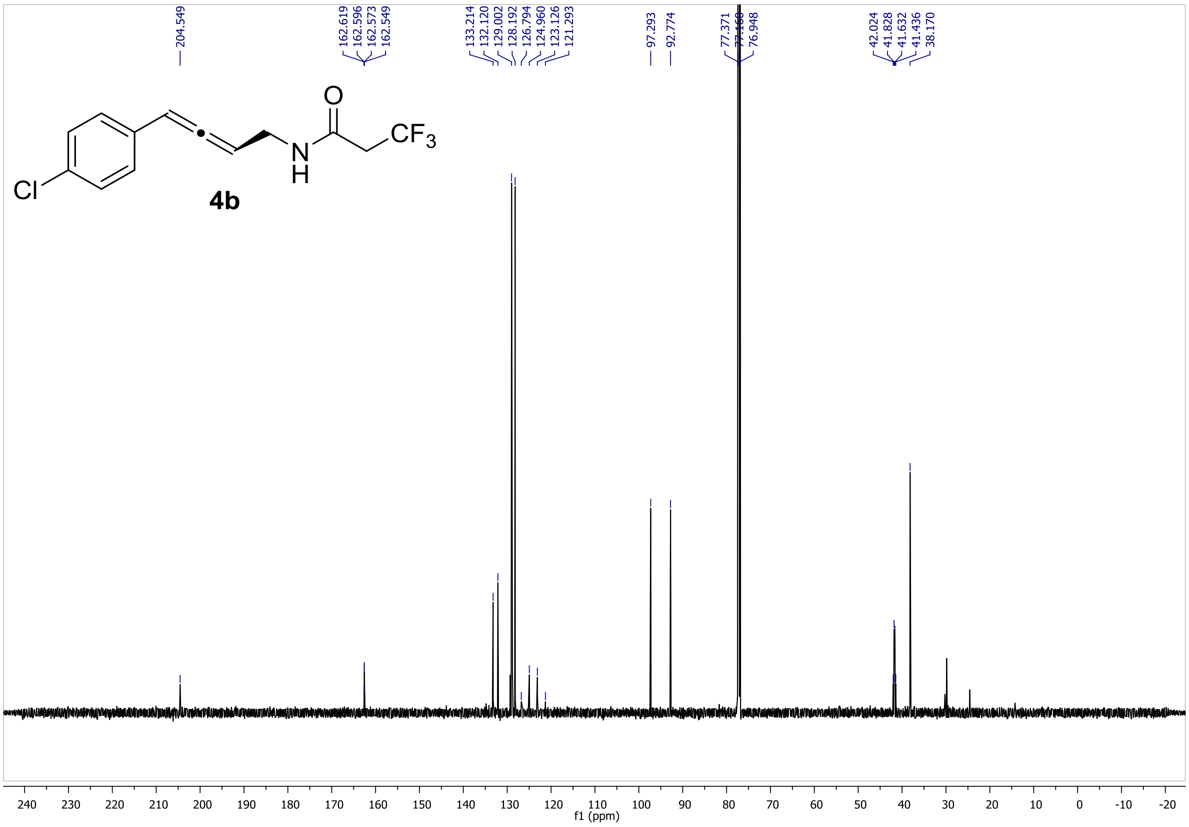

^{19}F NMR, 376 MHz, $CDCl_3$:

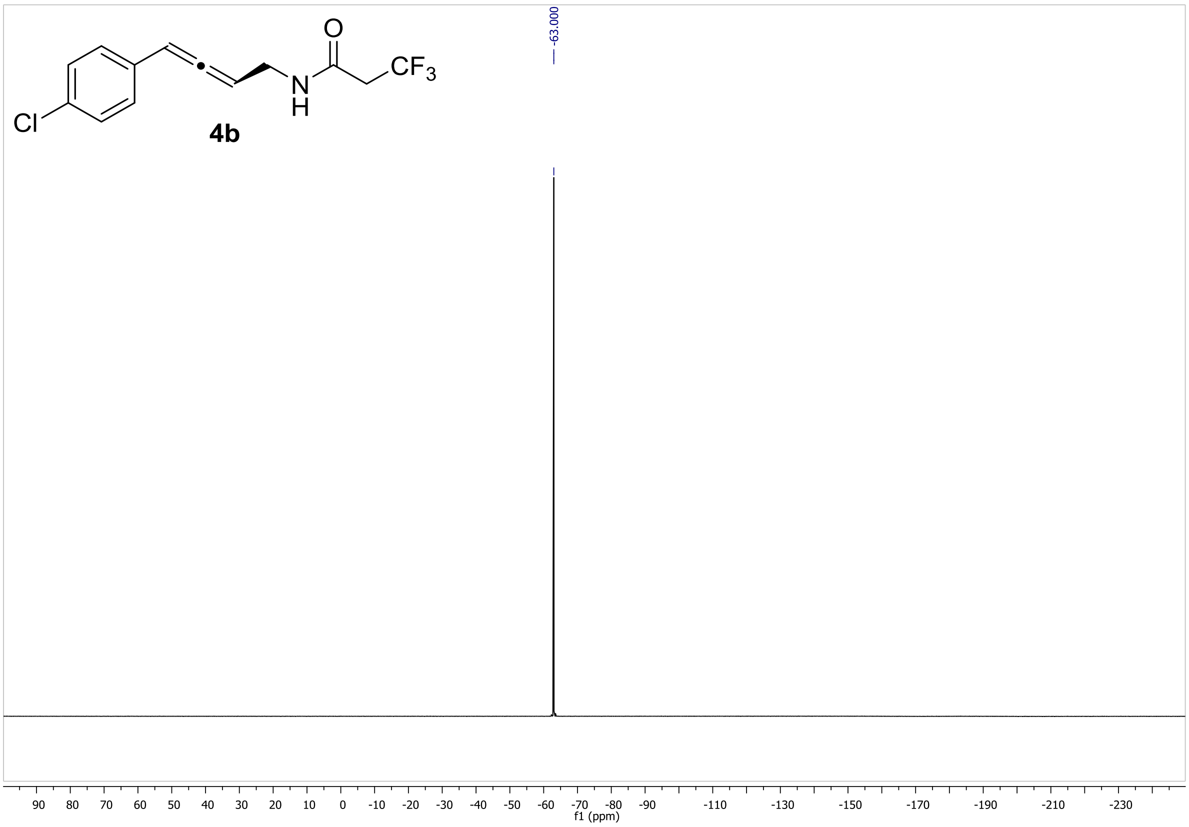

(*R*)-*N*-(4-(4-chlorophenyl)buta-2,3-dien-1-yl)-6-oxoheptanamide (4c):

^{1}H NMR, 600 MHz, CDCl$_3$:

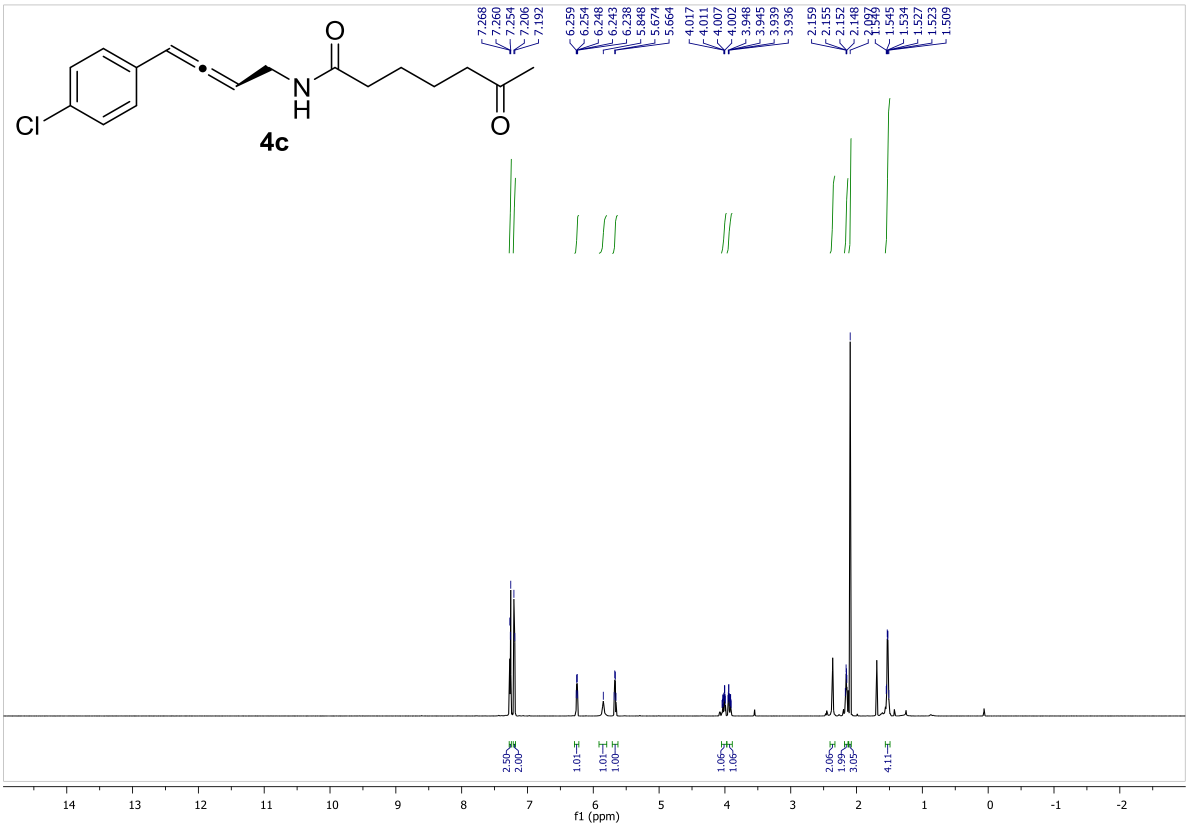

^{13}C NMR, 150 MHz, CDCl$_3$:

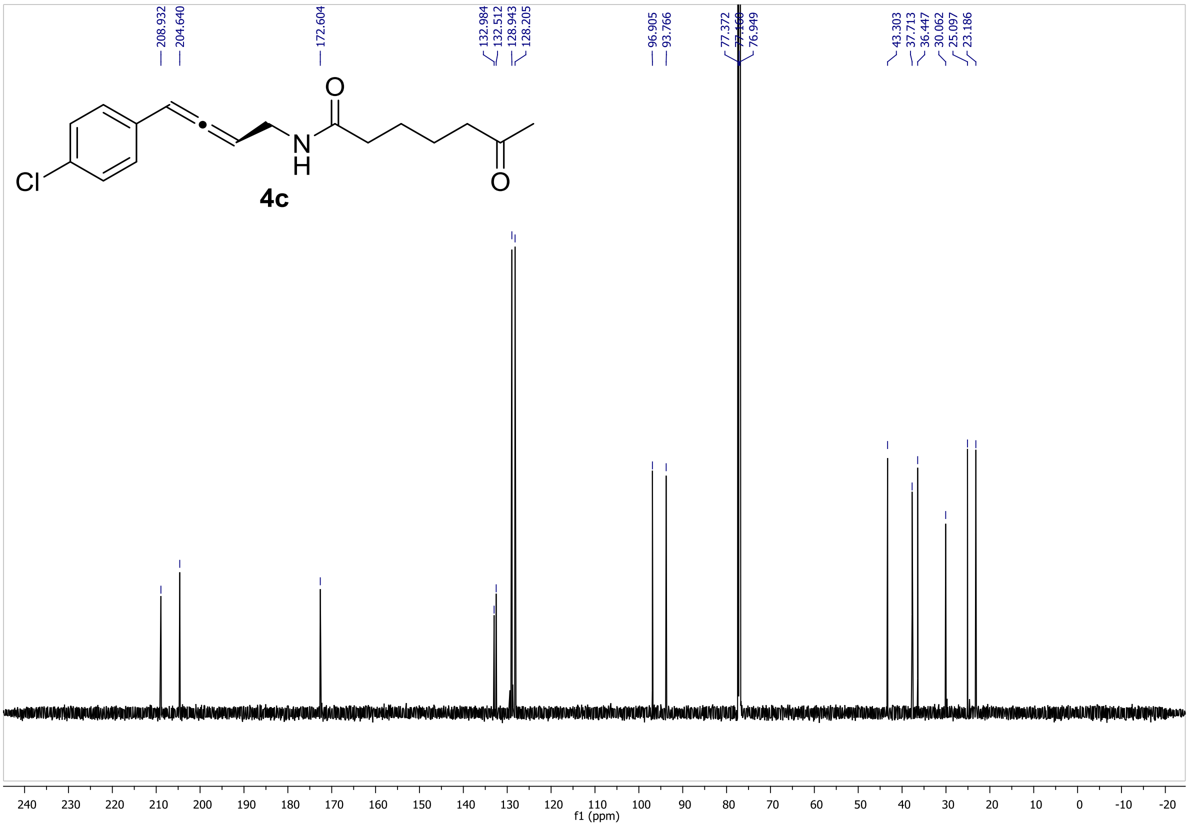

(*R*)-*N*-(4-(4-chlorophenyl)buta-2,3-dien-1-yl)-4-formylbenzamide (4d):

^{1}H NMR, 600 MHz, CDCl$_3$:

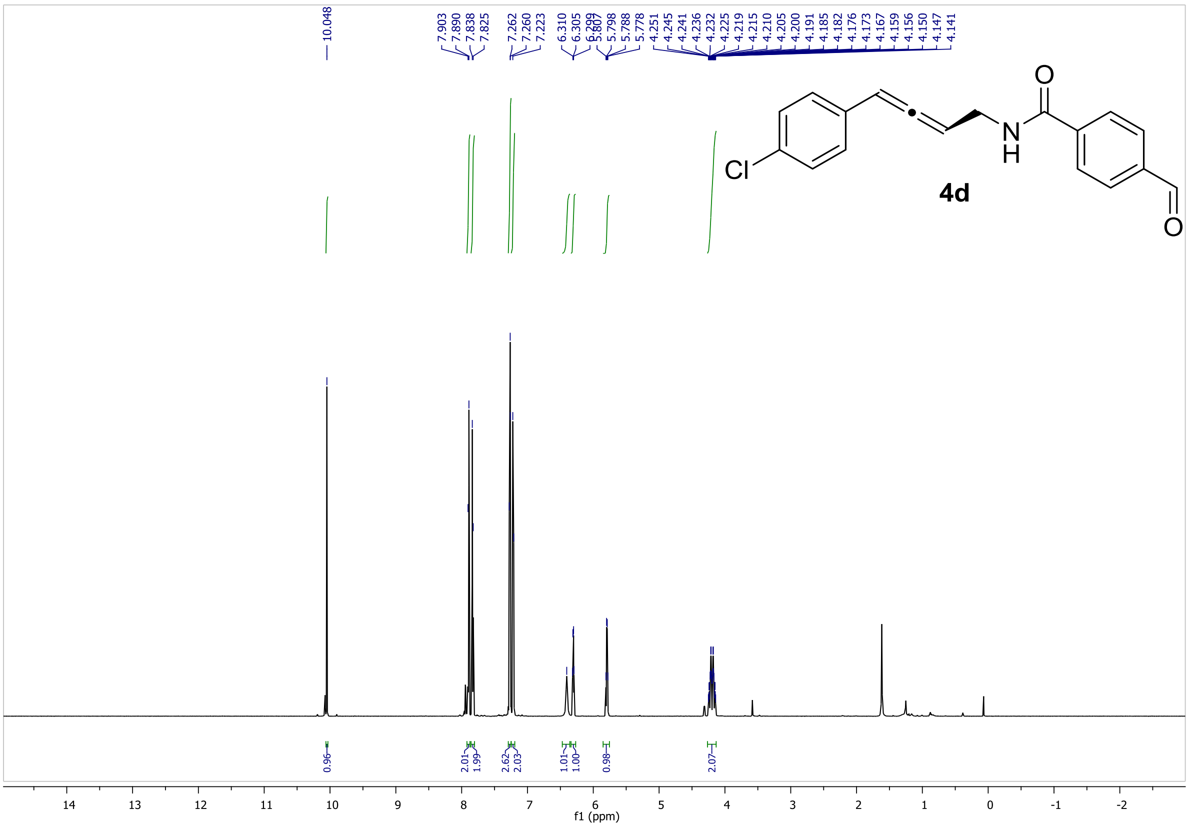

^{13}C NMR, 150 MHz, CDCl$_3$:

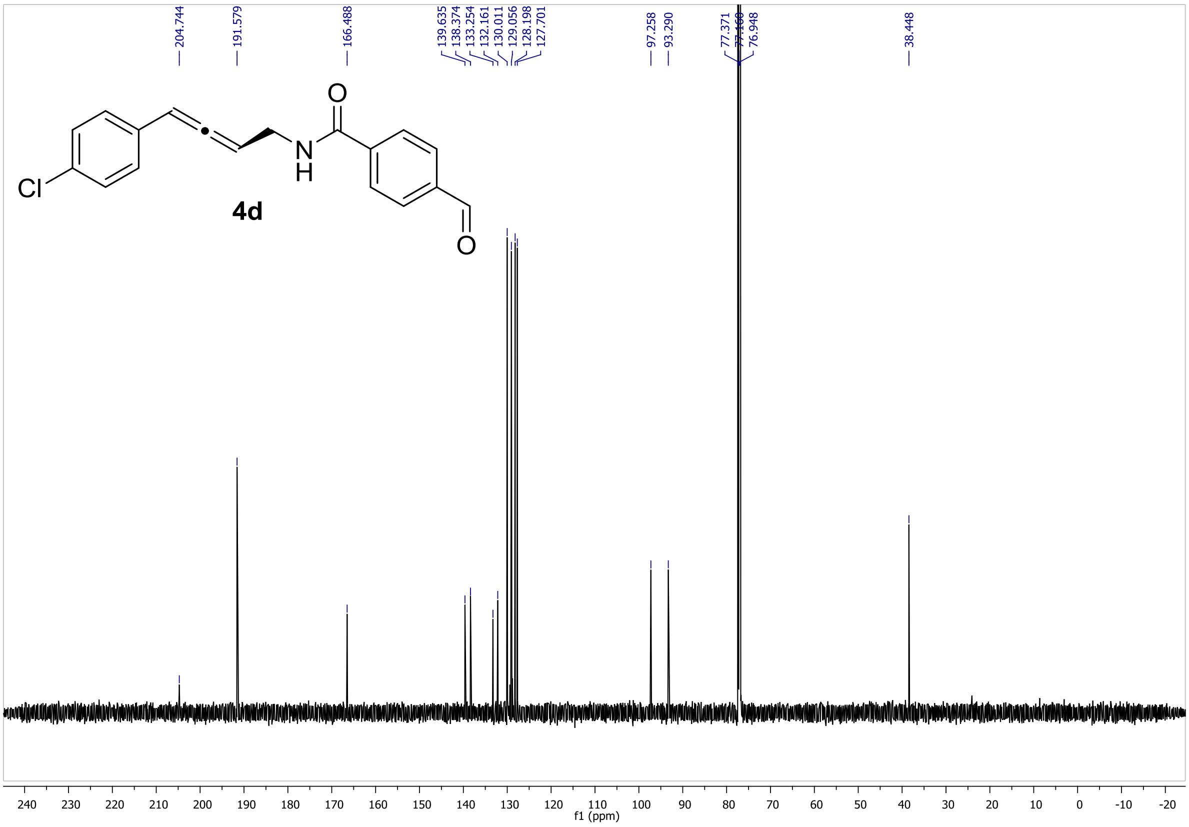

(1*R*,3*s*,5*S*)-*N*-((*R*)-4-(4-chlorophenyl)buta-2,3-dien-1-yl)-6-oxabicyclo[3.1.0]hexane-3-carboxamide (4e):

^{1}H NMR, 600 MHz, CDCl$_3$:

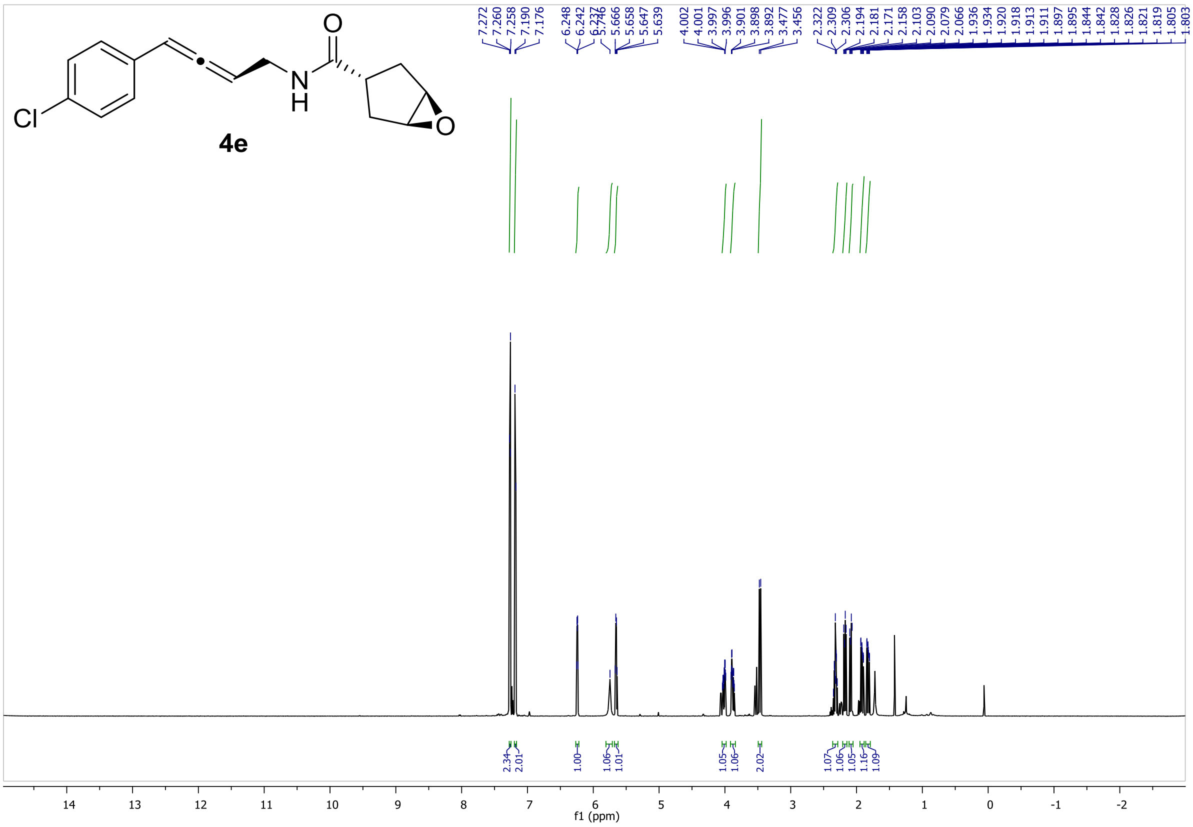

^{13}C NMR, 150 MHz, CDCl$_3$:

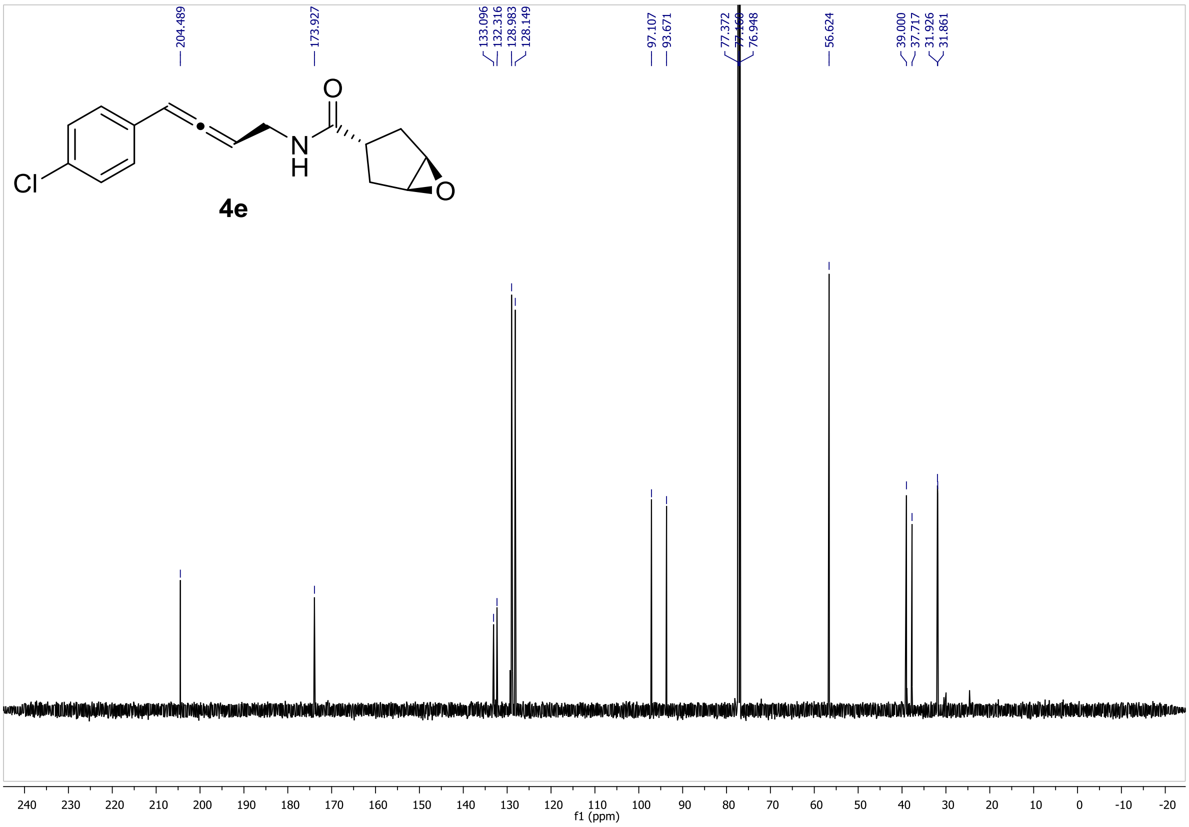

(*R*)-*N*-(4-(4-chlorophenyl)buta-2,3-dien-1-yl)thiophene-2-carboxamide (4f):

^{1}H NMR, 600 MHz, $CDCl_3$:

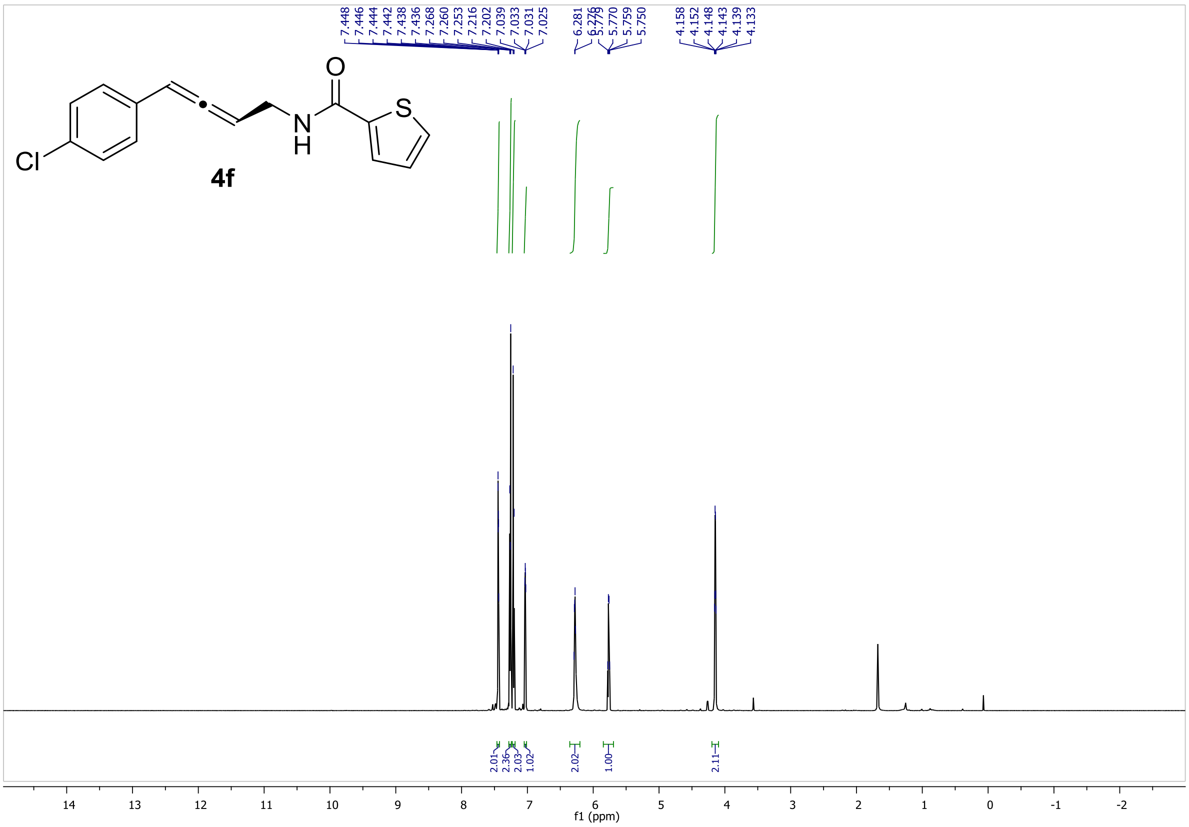

^{13}C NMR, 150 MHz, $CDCl_3$:

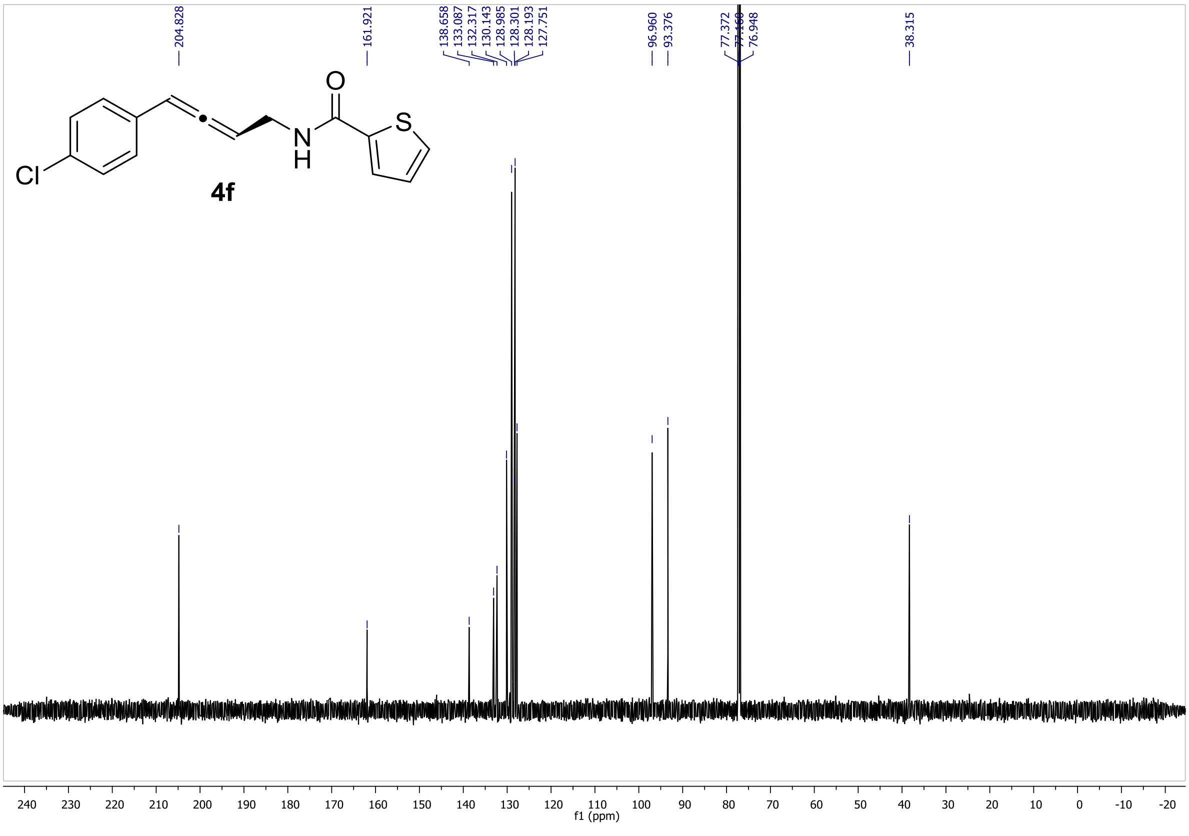

***tert*-butyl (*R*)-(4-(4-chlorophenyl)buta-2,3-dien-1-yl)carbamate (4g):**

^{1}H NMR, 600 MHz, $CDCl_3$:

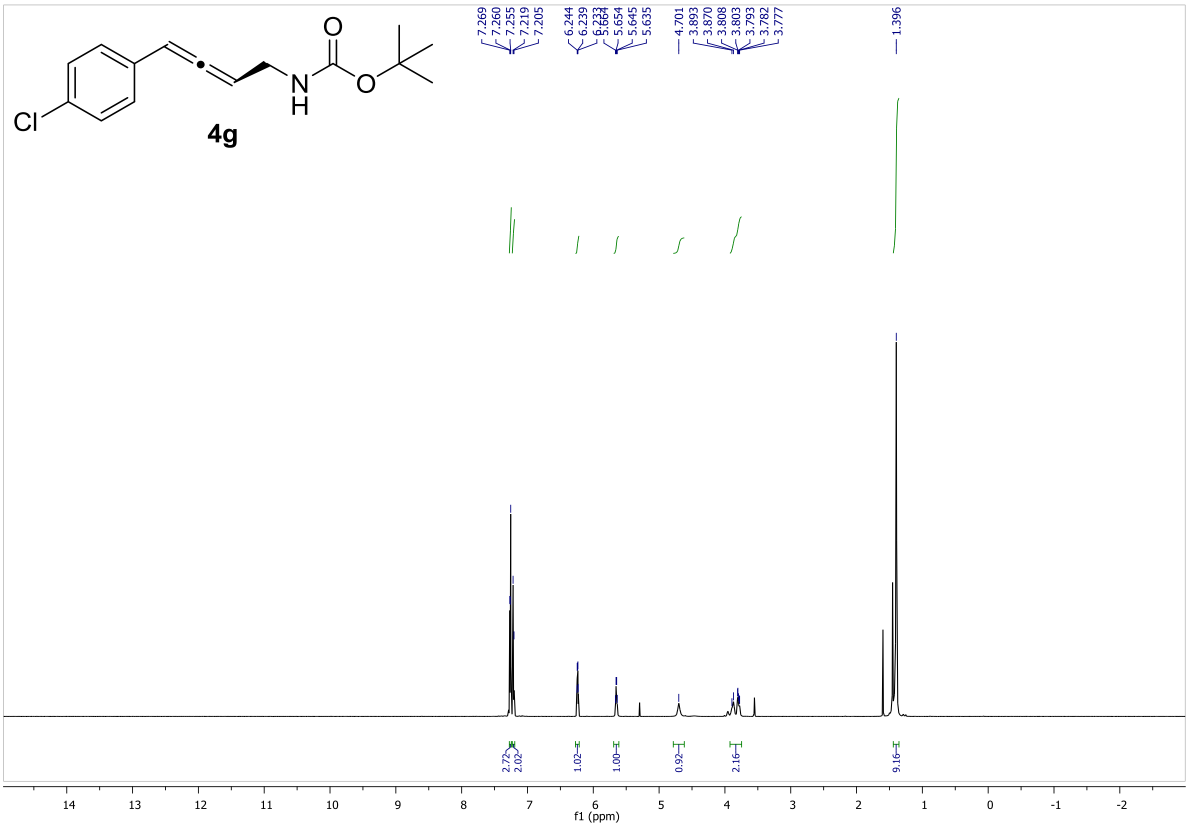

^{13}C NMR, 150 MHz, $CDCl_3$:

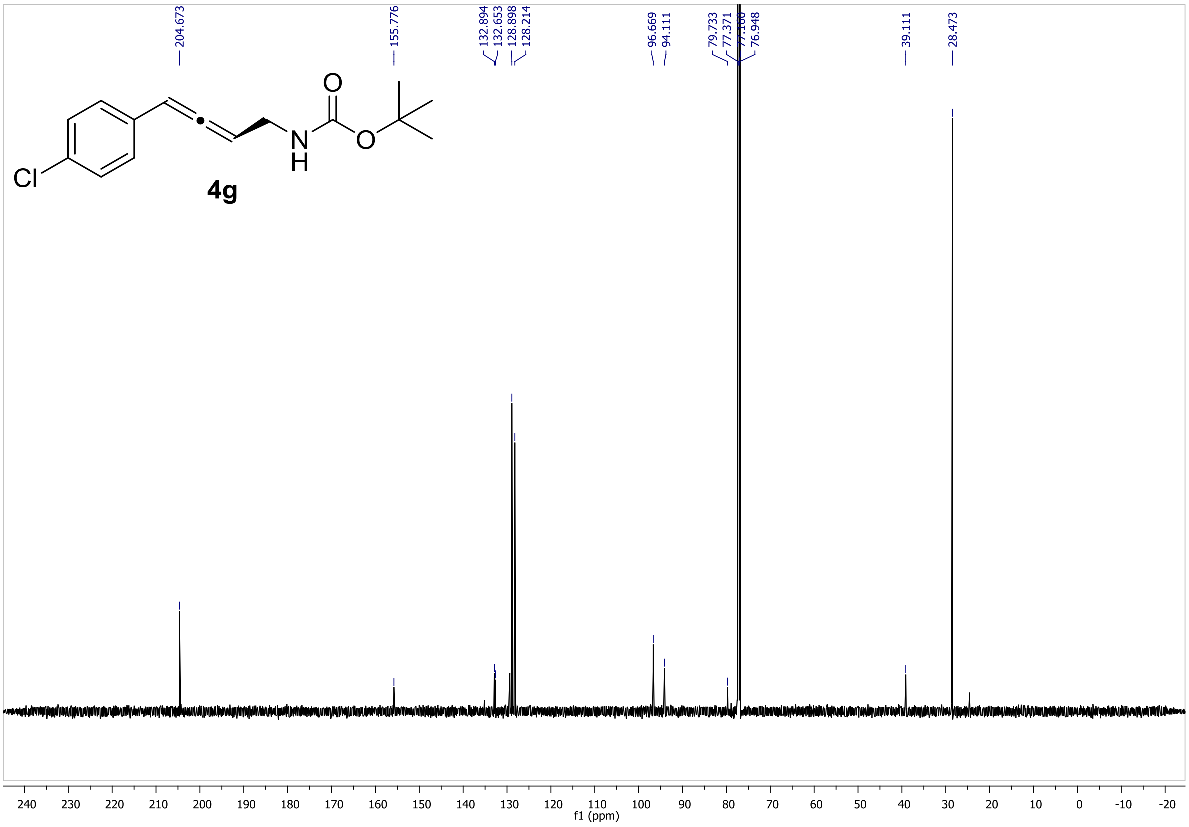

(*R*)-*N*-(4-(4-bromophenyl)buta-2,3-dien-1-yl)-1-(4-chlorophenyl)cyclopentane-1-carboxamide (4h):

^{1}H NMR, 600 MHz, $CDCl_3$:

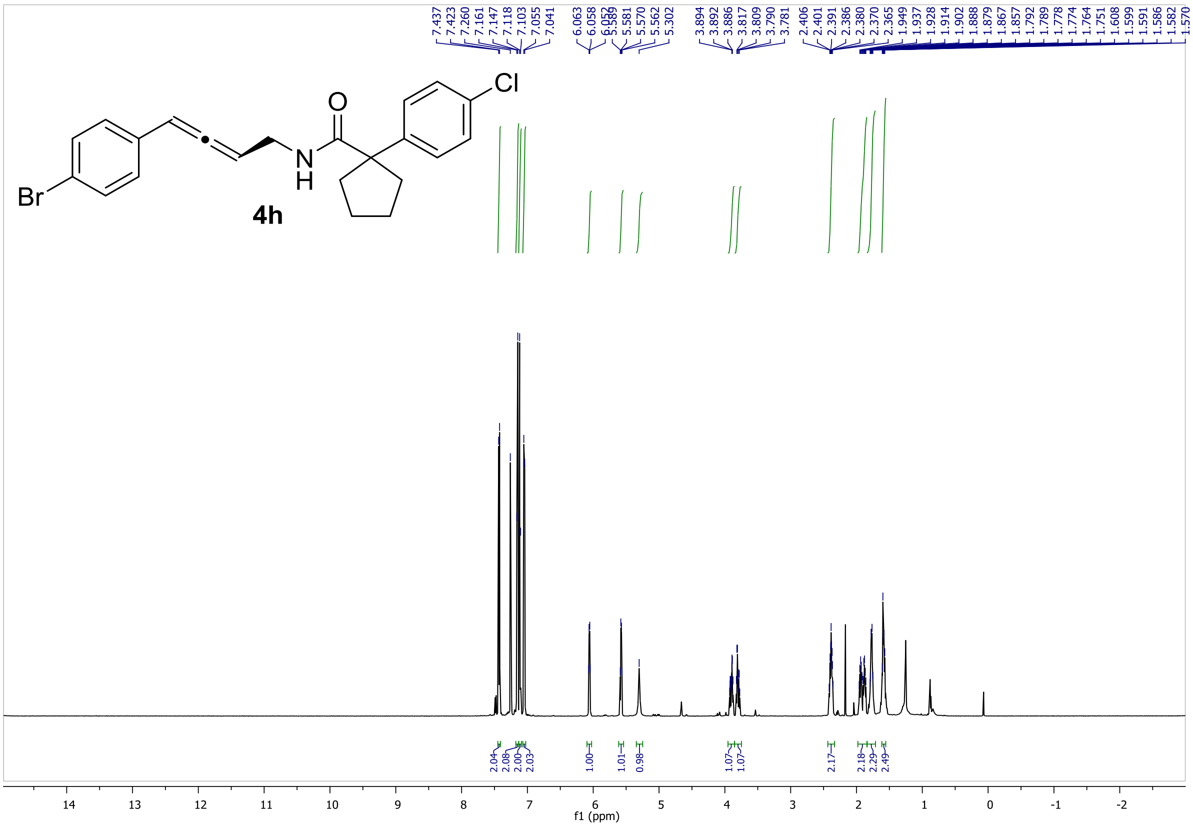

^{13}C NMR, 150 MHz, $CDCl_3$:

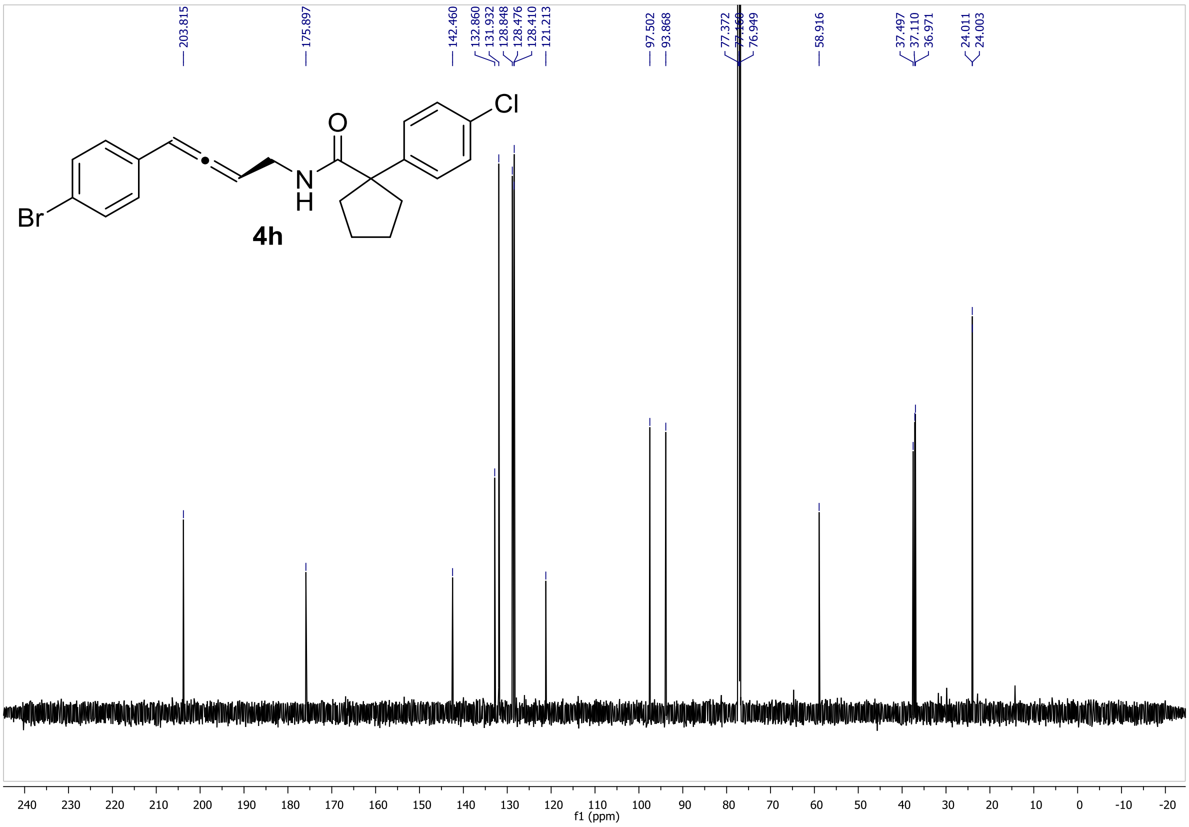

(*R*)-*N*-(4-(4-bromophenyl)buta-2,3-dien-1-yl)-4-hydroxybutanamide (4i):

^{1}H NMR, 600 MHz, $CDCl_3$:

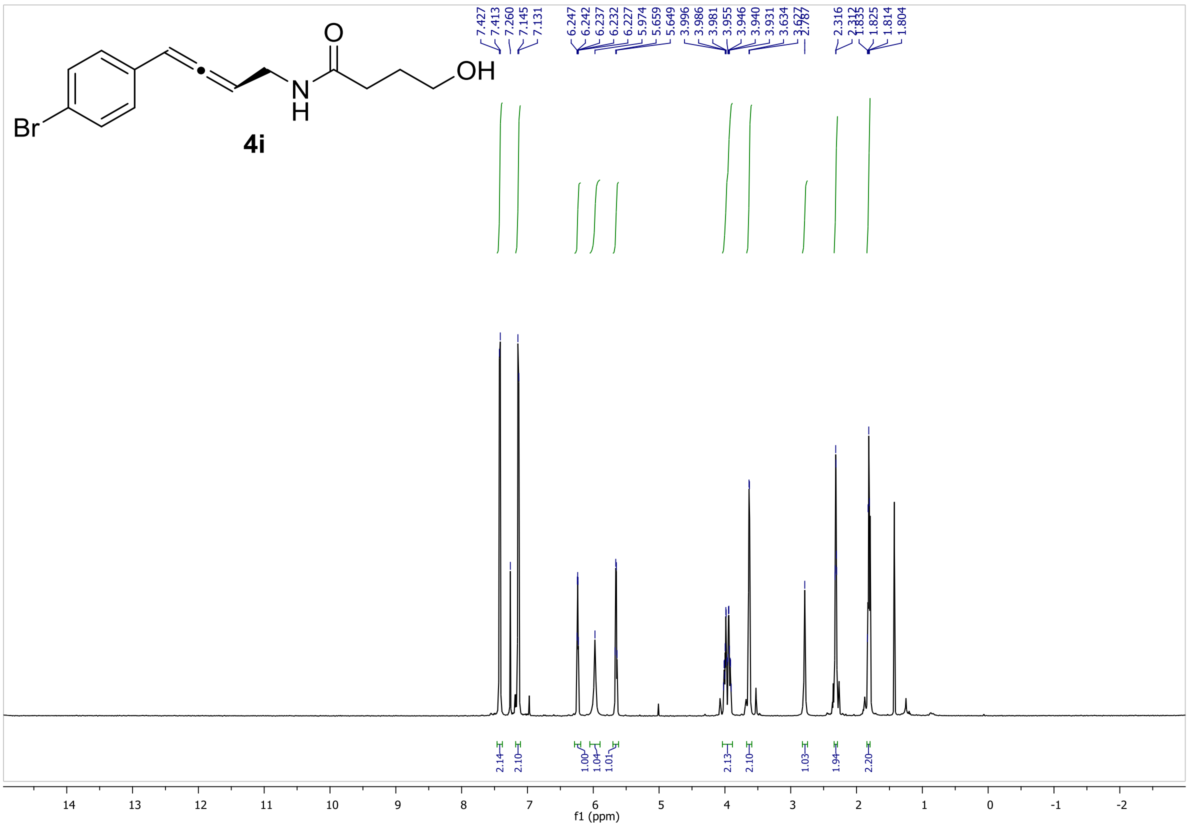

^{13}C NMR, 150 MHz, $CDCl_3$:

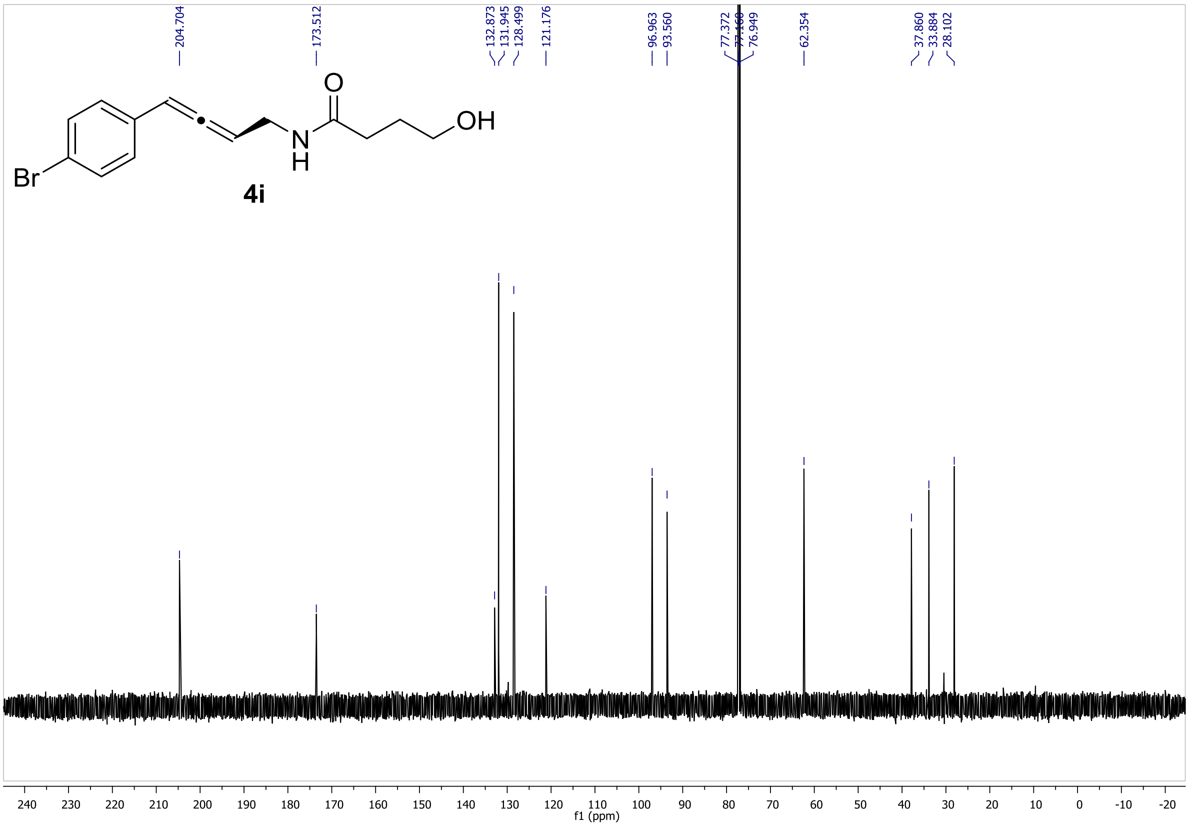

(*R*)-5-bromo-*N*-(4-(4-bromophenyl)buta-2,3-dien-1-yl)furan-2-carboxamide (4j):

^{1}H NMR, 600 MHz, $CDCl_3$:

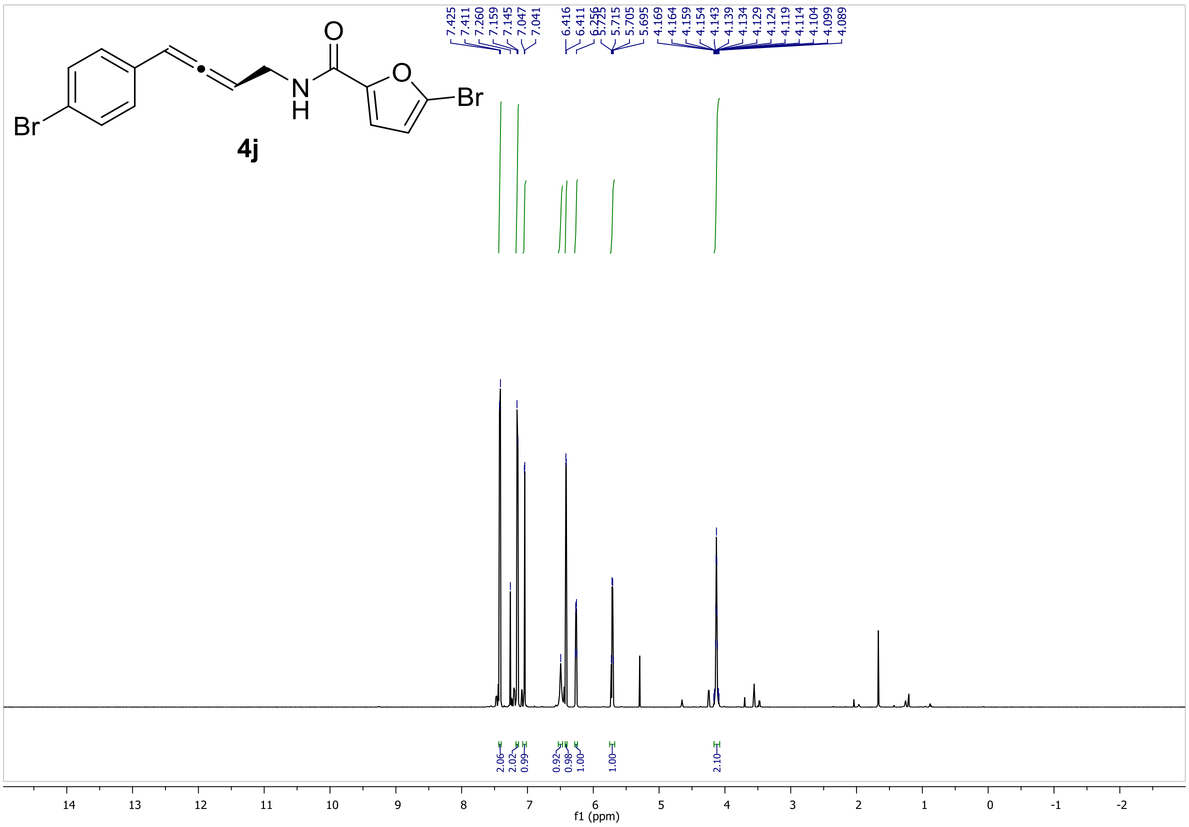

^{13}C NMR, 150 MHz, $CDCl_3$:

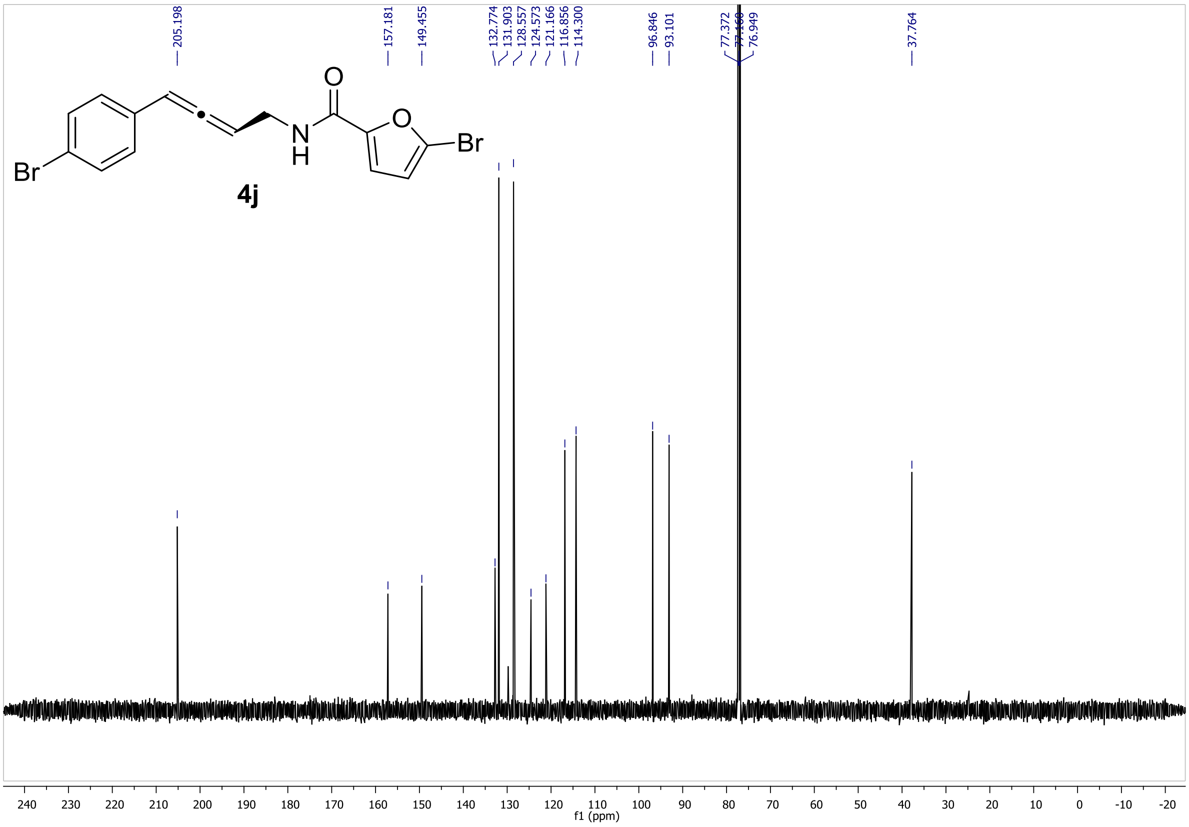

(*R*)-*N*-(4-(4-bromophenyl)buta-2,3-dien-1-yl)cyclopropanecarboxamide (4k):

^{1}H NMR, 600 MHz, CDCl$_3$:

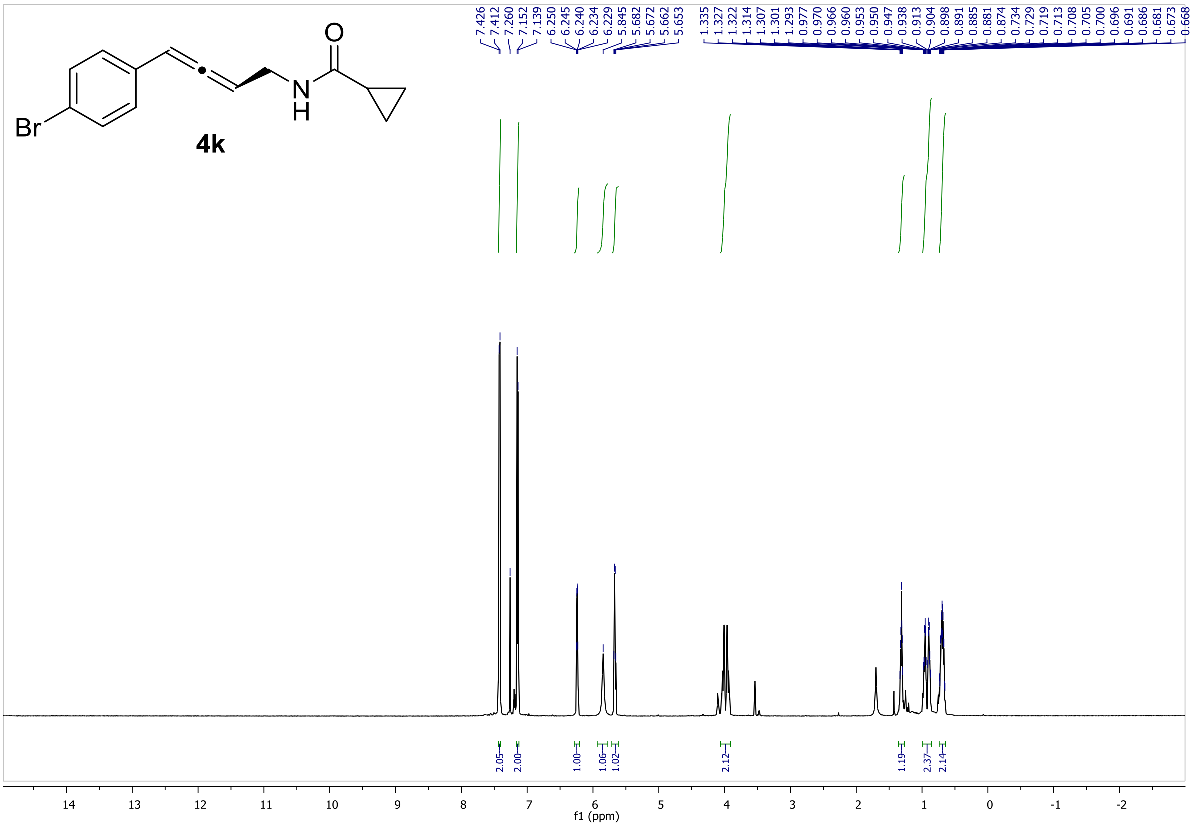

^{13}C NMR, 150 MHz, CDCl$_3$:

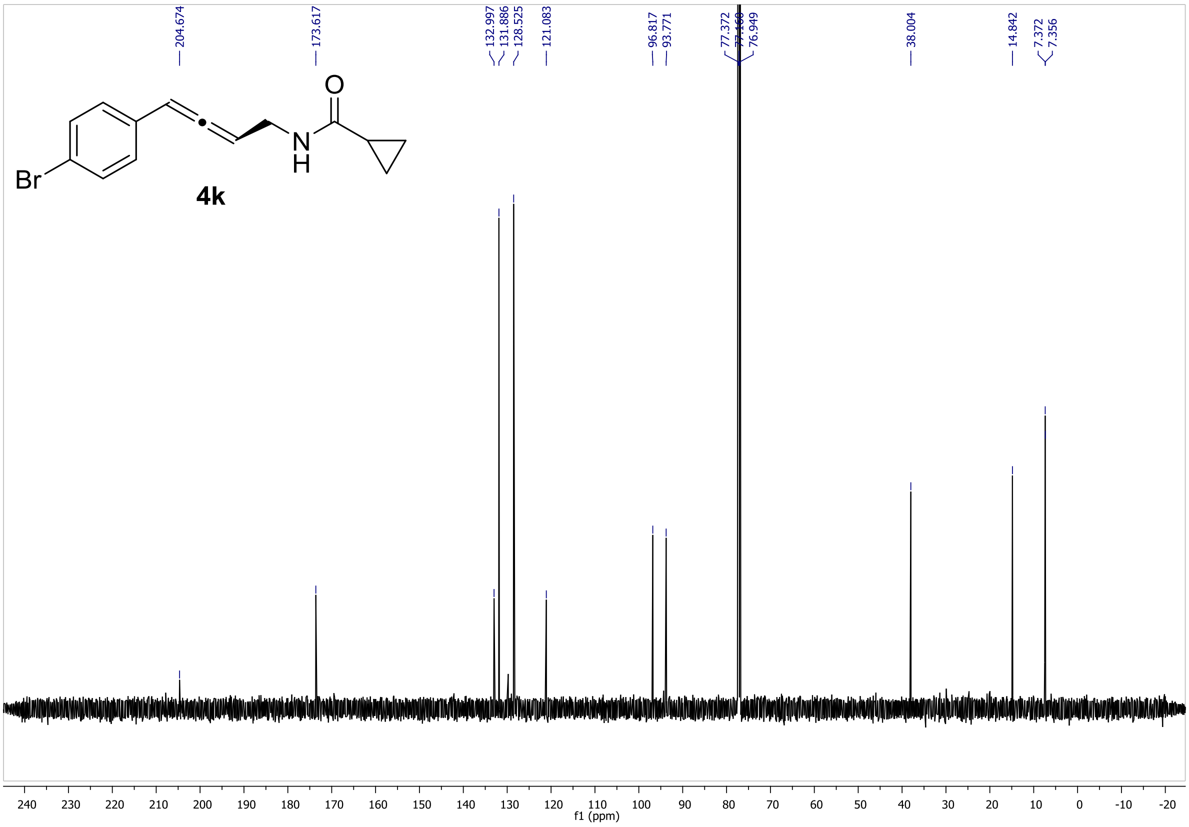

Methyl (*R*)-4-((4-(4-bromophenyl)buta-2,3-dien-1-yl)amino)-4-oxobutanoate (4l):

^{1}H NMR, 600 MHz, $CDCl_3$:

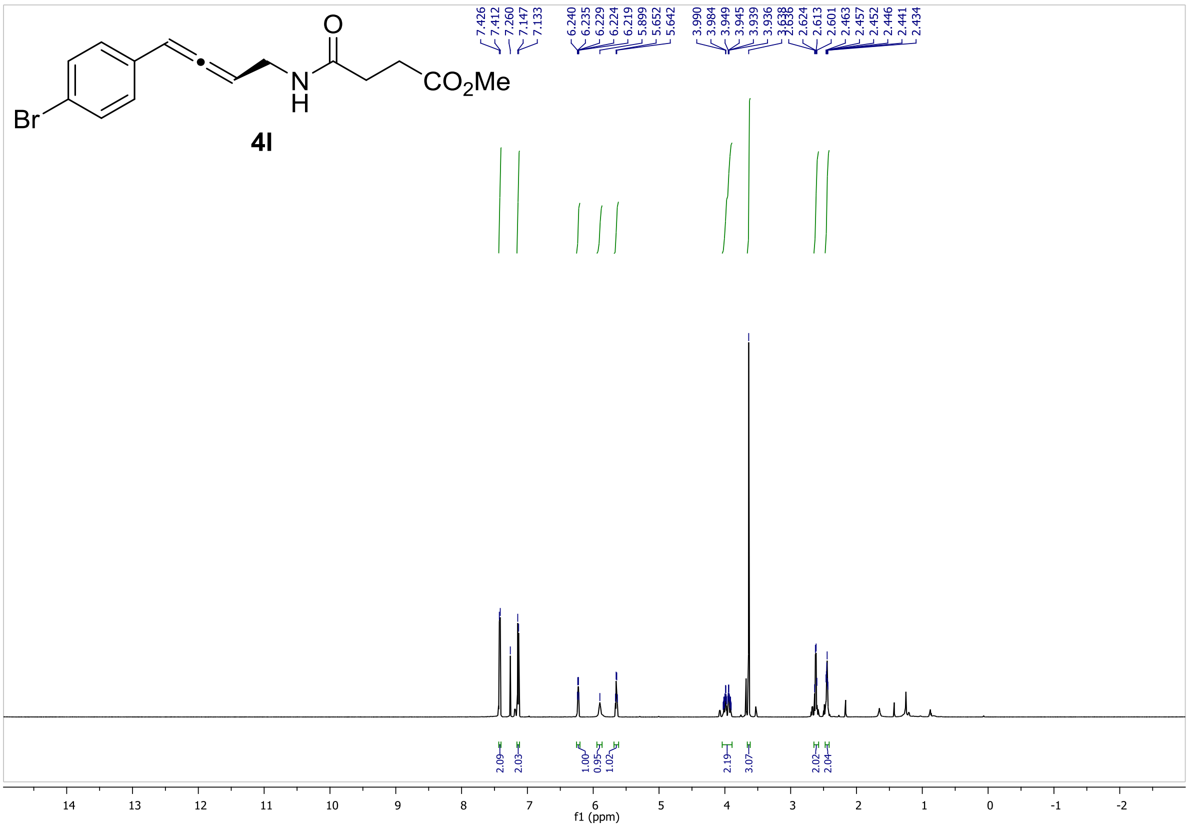

^{13}C NMR, 150 MHz, $CDCl_3$:

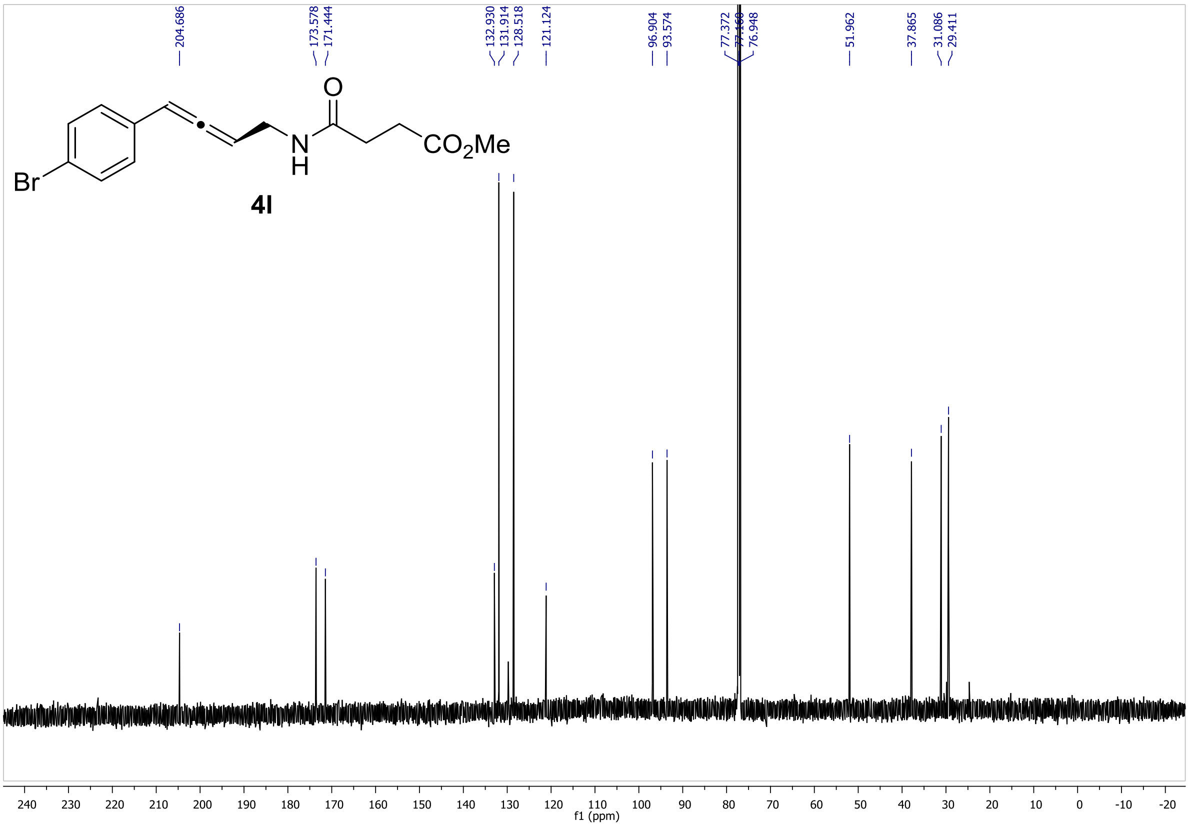

(*R*)-*N*-(4-(4-chlorophenyl)buta-2,3-dien-1-yl)acetamide (4m):

^{1}H NMR, 600 MHz, $CDCl_3$:

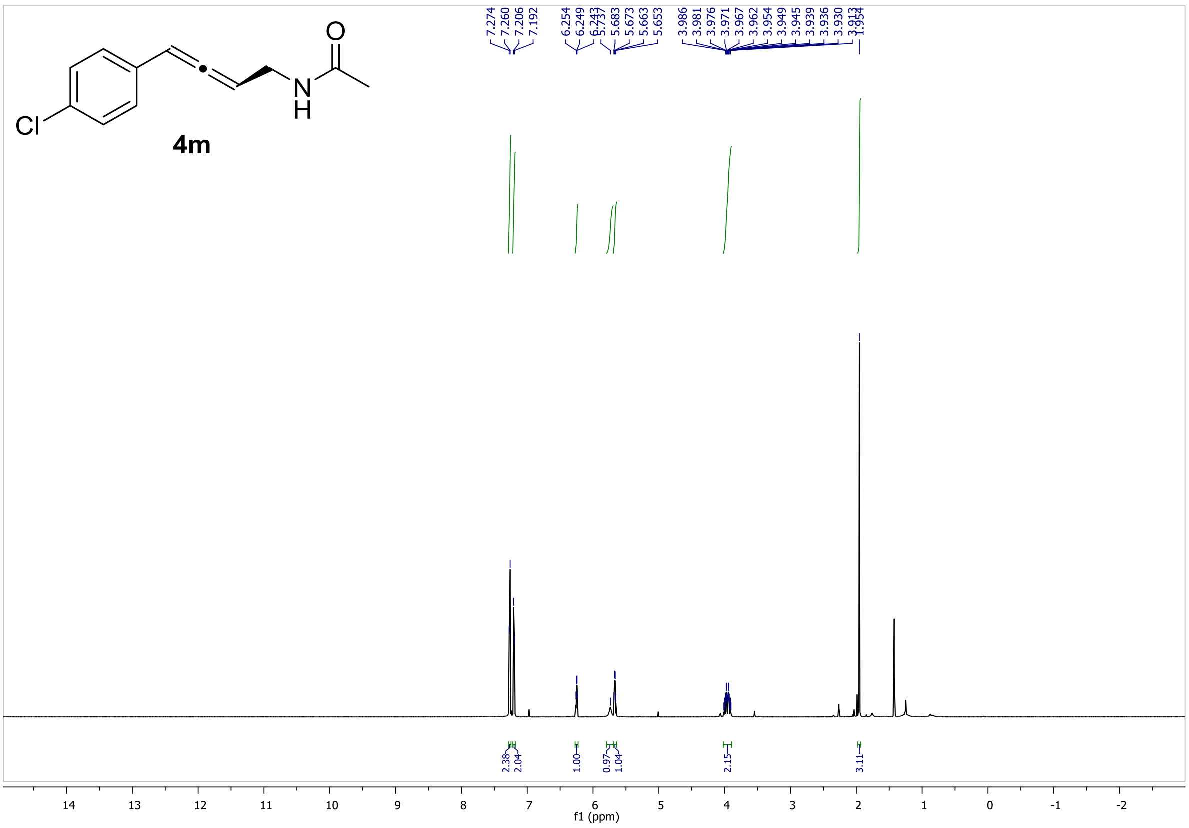

^{13}C NMR, 150 MHz, $CDCl_3$:

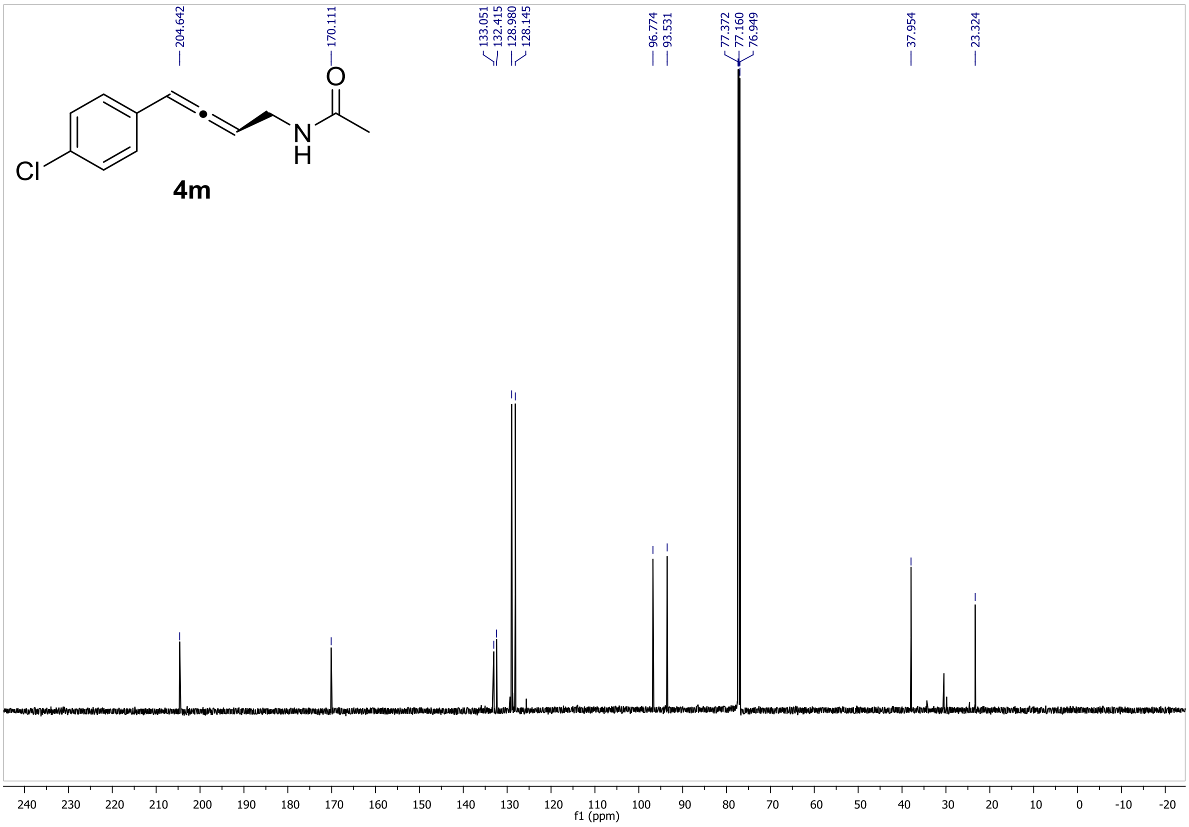

(*R*)-*N*-(4-(4-bromophenyl)buta-2,3-dien-1-yl)acetamide (4n):

^{1}H NMR, 600 MHz, $CDCl_3$:

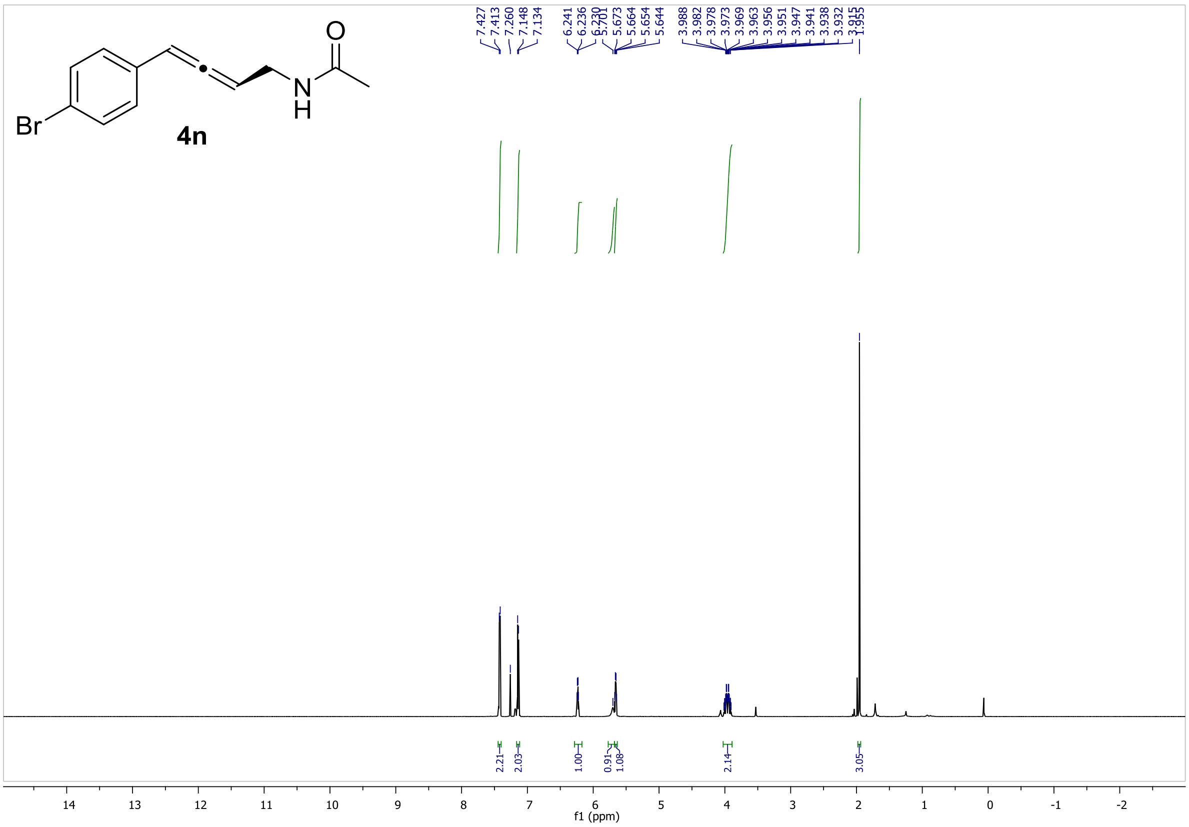

^{13}C NMR, 150 MHz, $CDCl_3$:

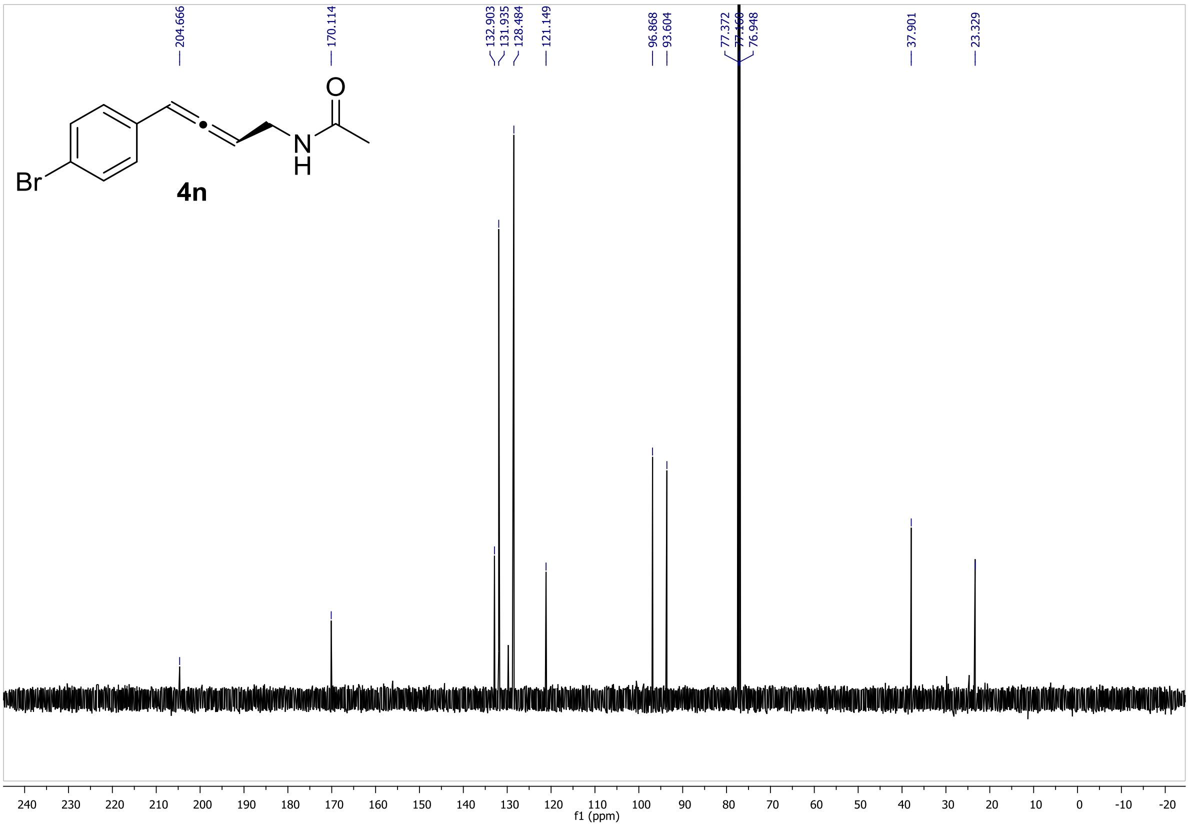

(*R*)-*N*-(4-(4-fluorophenyl)buta-2,3-dien-1-yl)acetamide (4o):

^{1}H NMR, 600 MHz, $CDCl_3$:

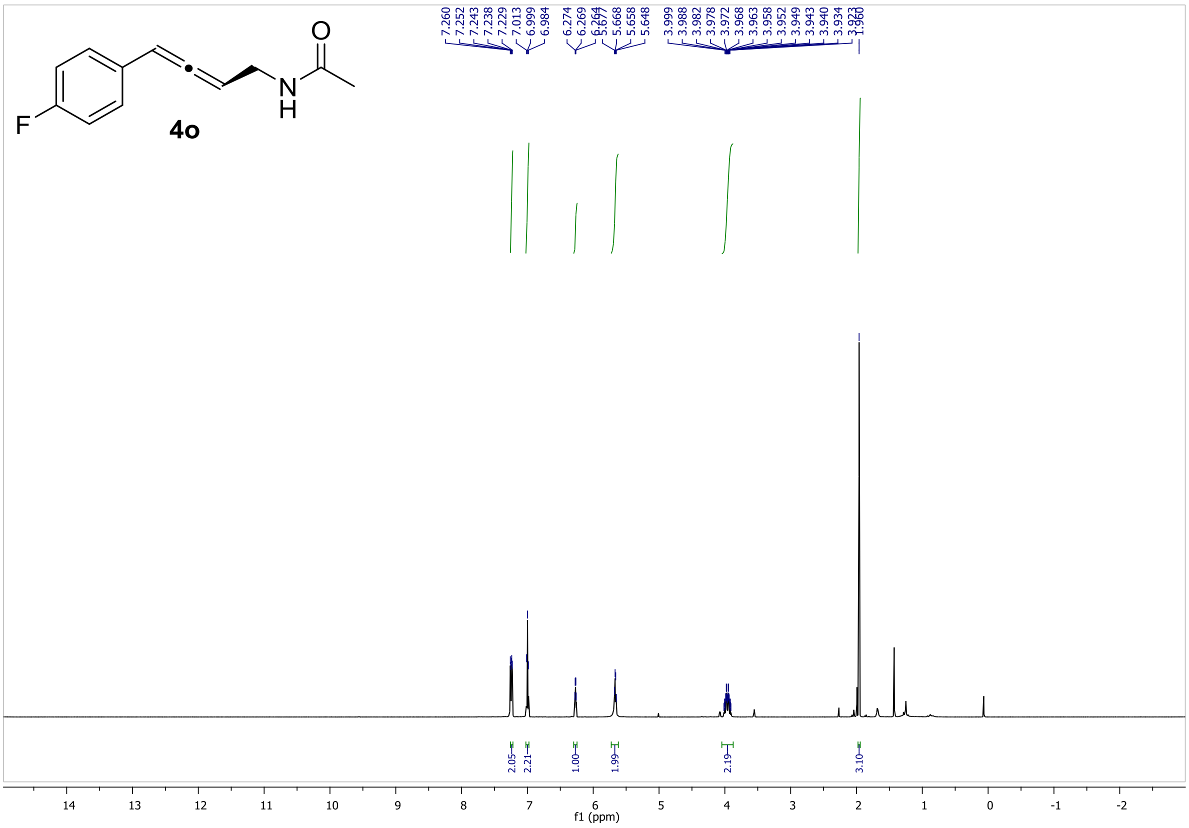

^{13}C NMR, 150 MHz, $CDCl_3$:

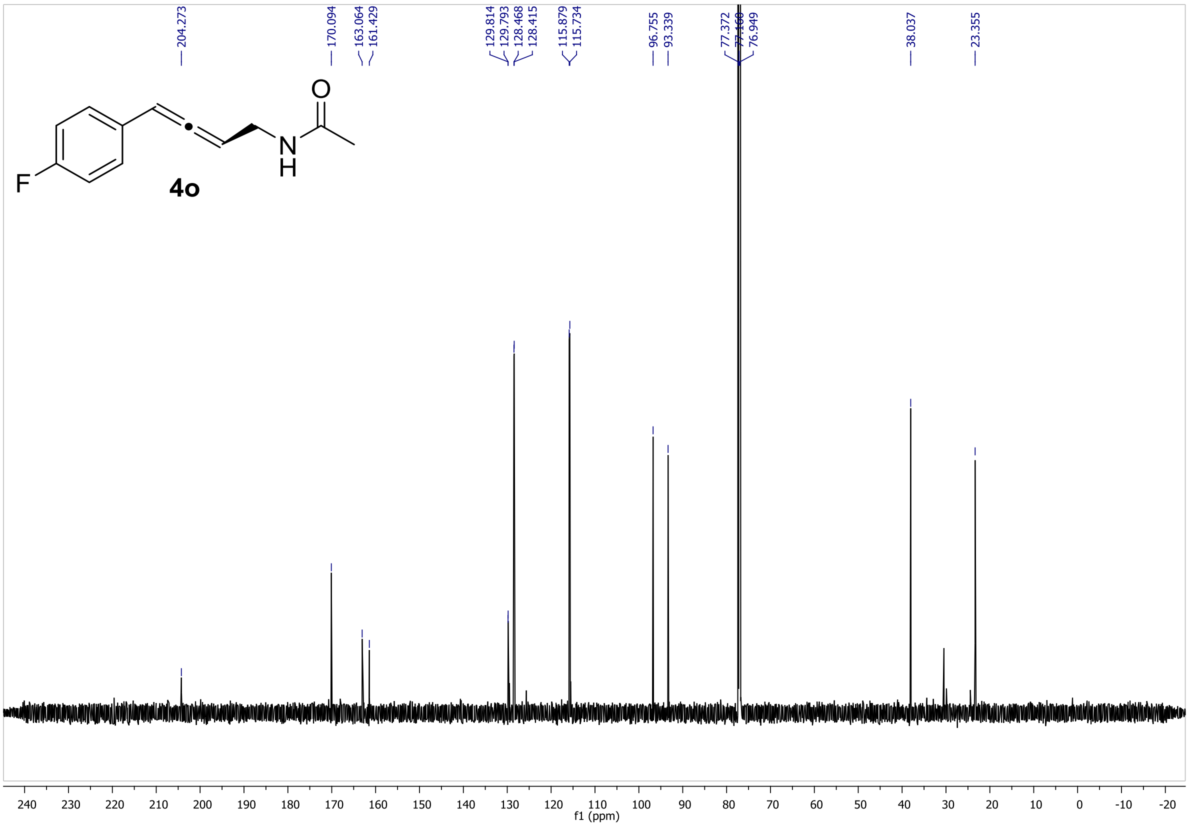

^{19}F NMR, 376 MHz, CDCl$_3$:

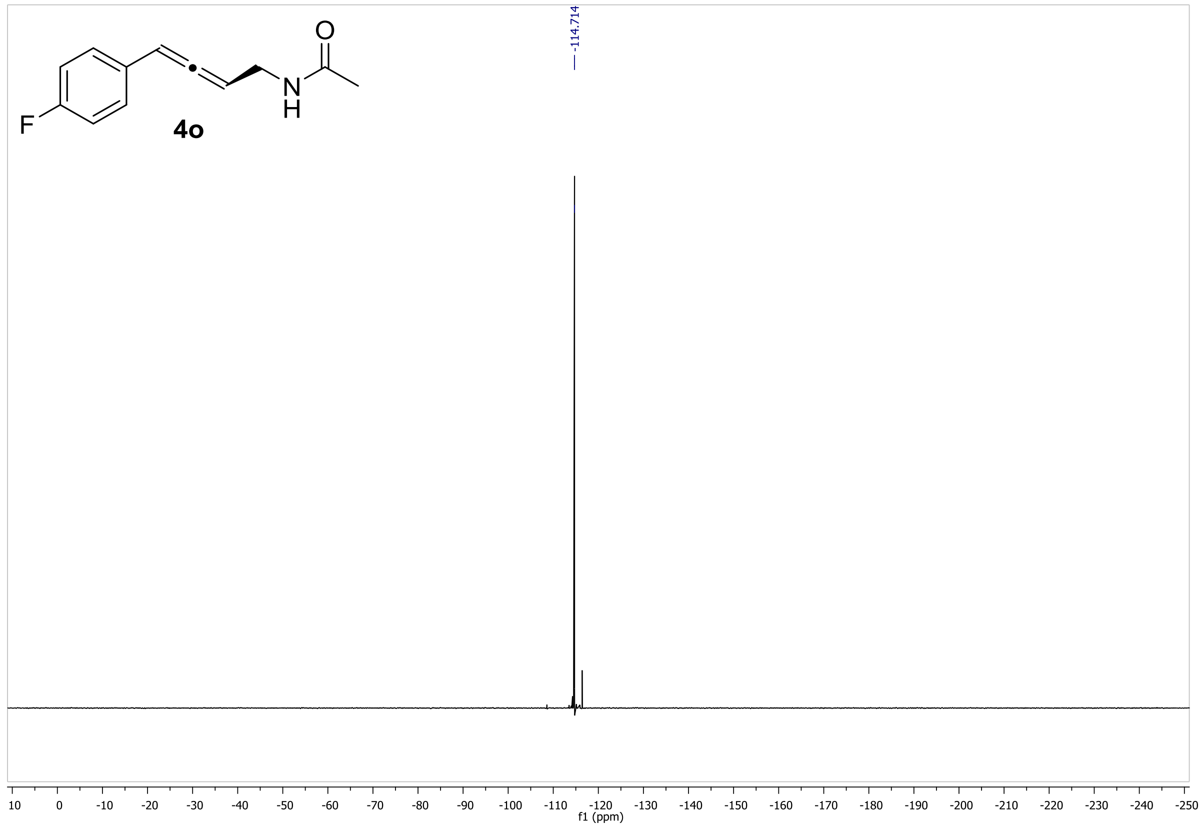

(*R*)-*N*-(4-(3-methoxyphenyl)buta-2,3-dien-1-yl)acetamide (4p):

^{1}H NMR, 600 MHz, CDCl$_3$:

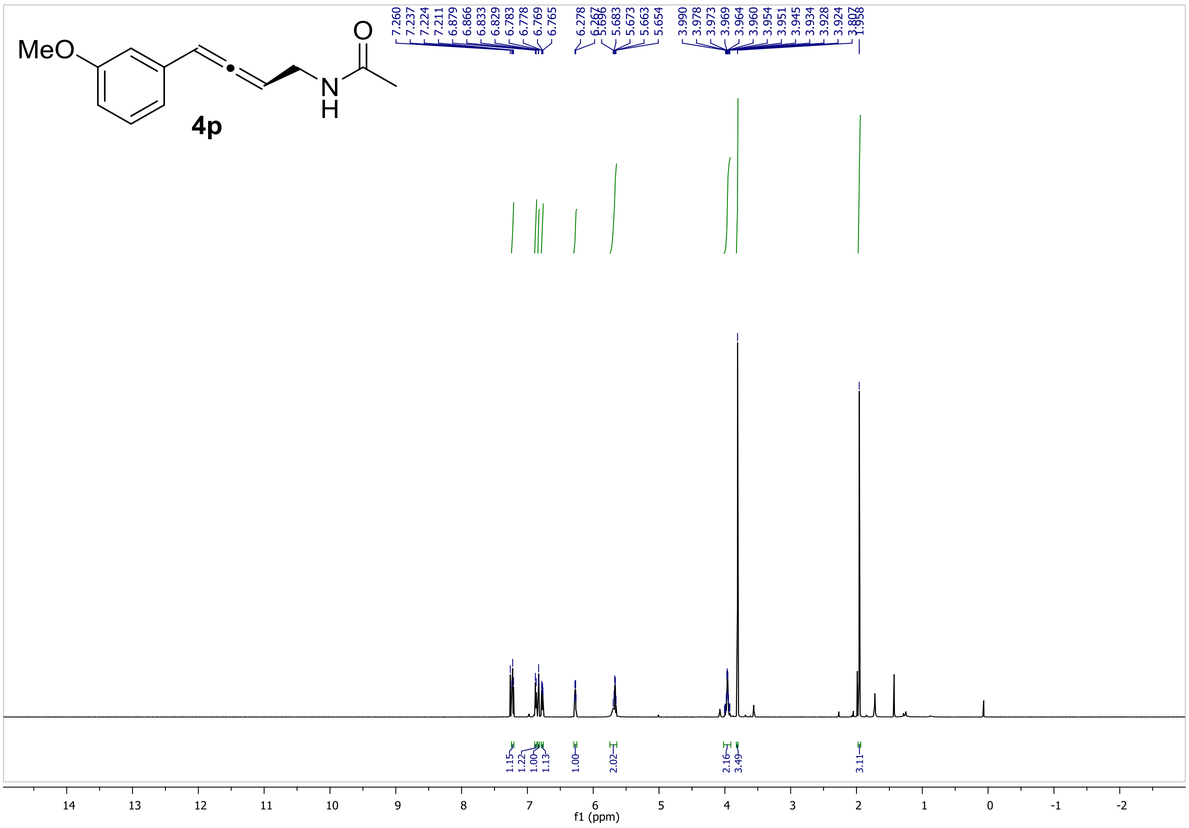

^{13}C NMR, 150 MHz, CDCl$_3$:

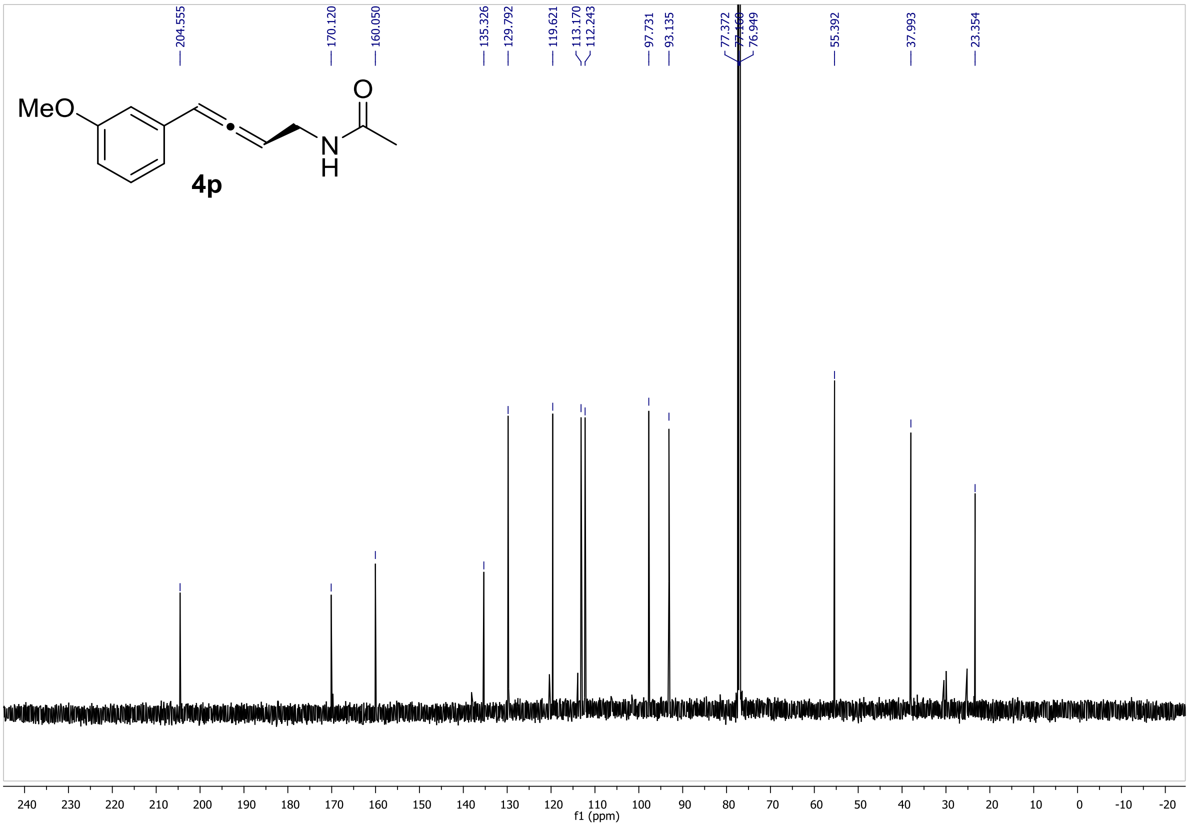

(*R*)-*N*-(4-(3-cyanophenyl)buta-2,3-dien-1-yl)acetamide (4q):

^{1}H NMR, 600 MHz, $CDCl_3$:

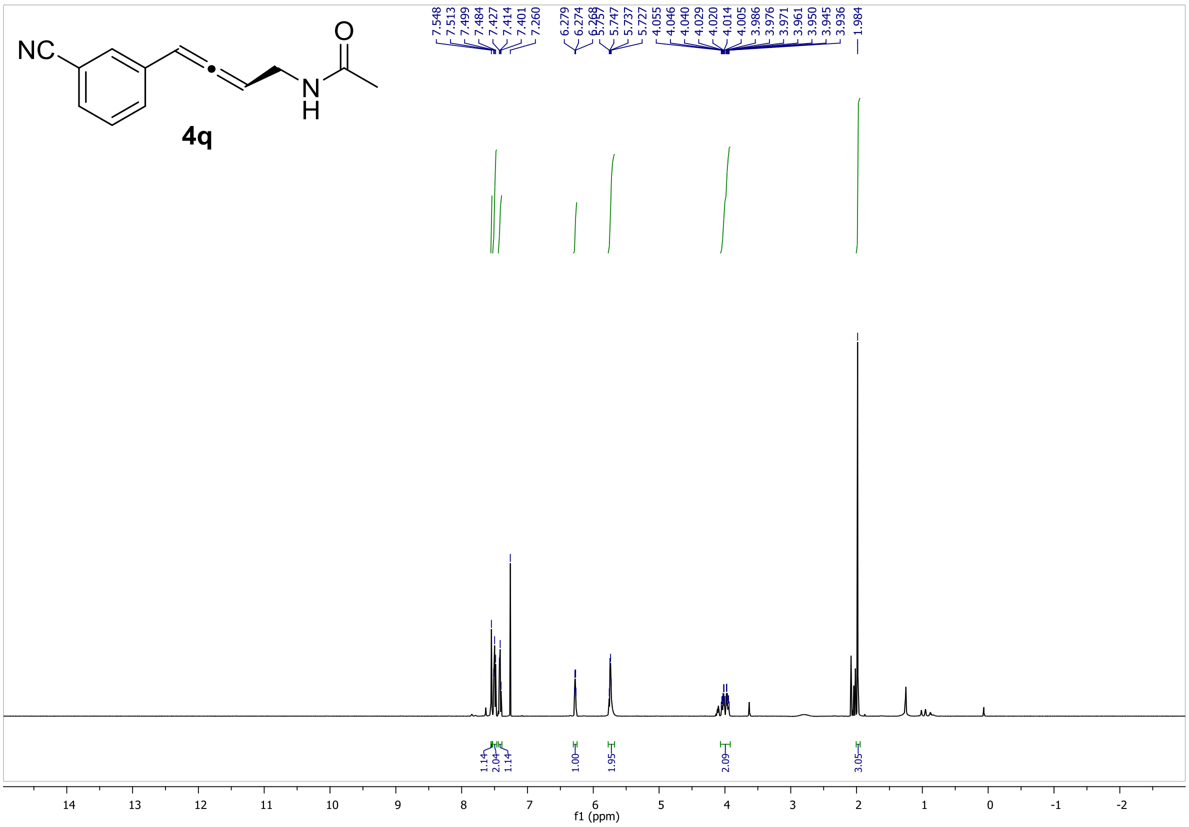

^{13}C NMR, 150 MHz, $CDCl_3$:

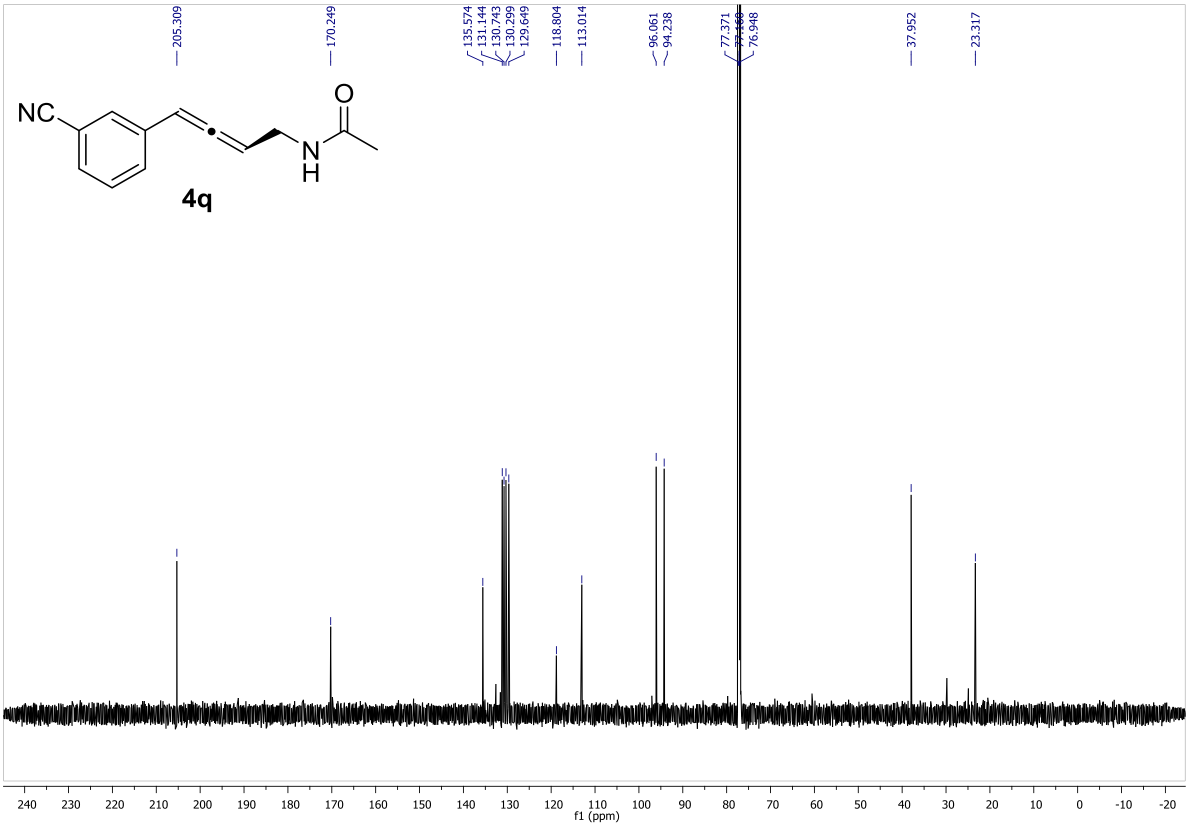

(*R*)-*N*-(4-(4-iodophenyl)buta-2,3-dien-1-yl)acetamide (4r):

^{1}H NMR, 600 MHz, $CDCl_3$:

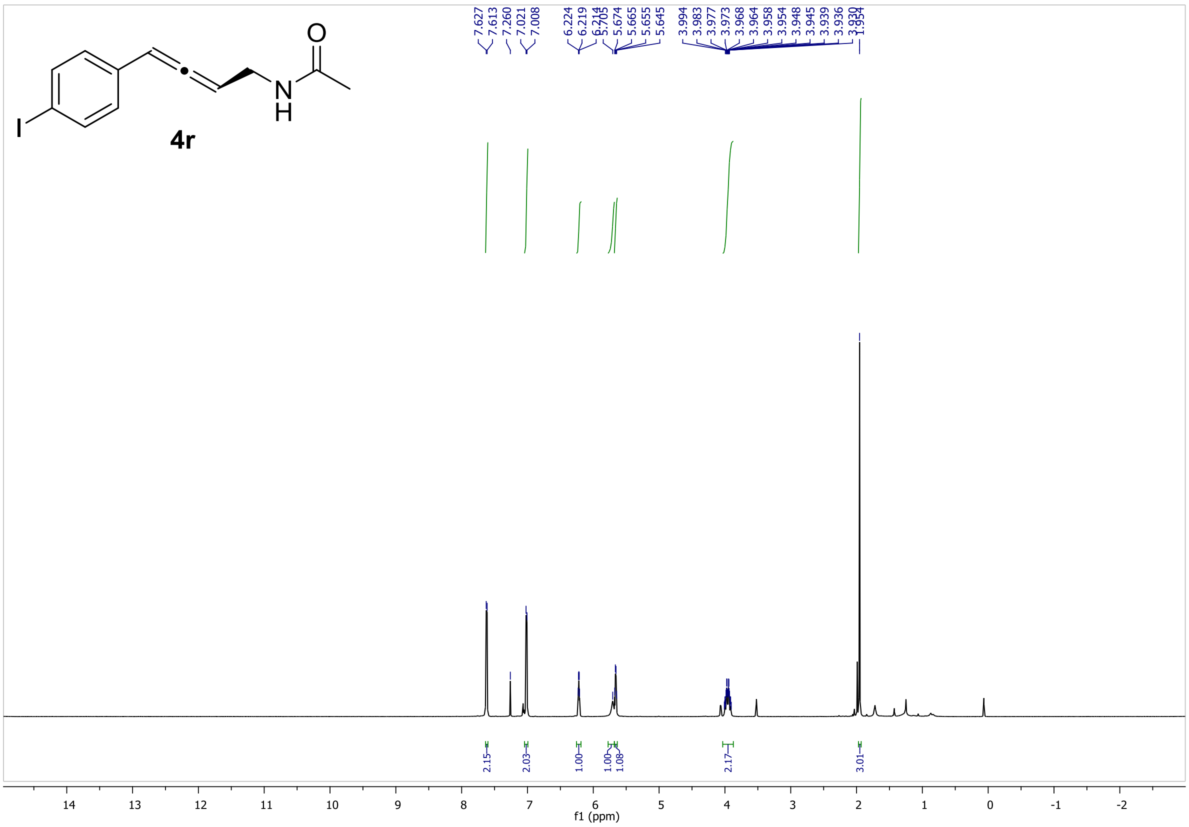

^{13}C NMR, 150 MHz, $CDCl_3$:

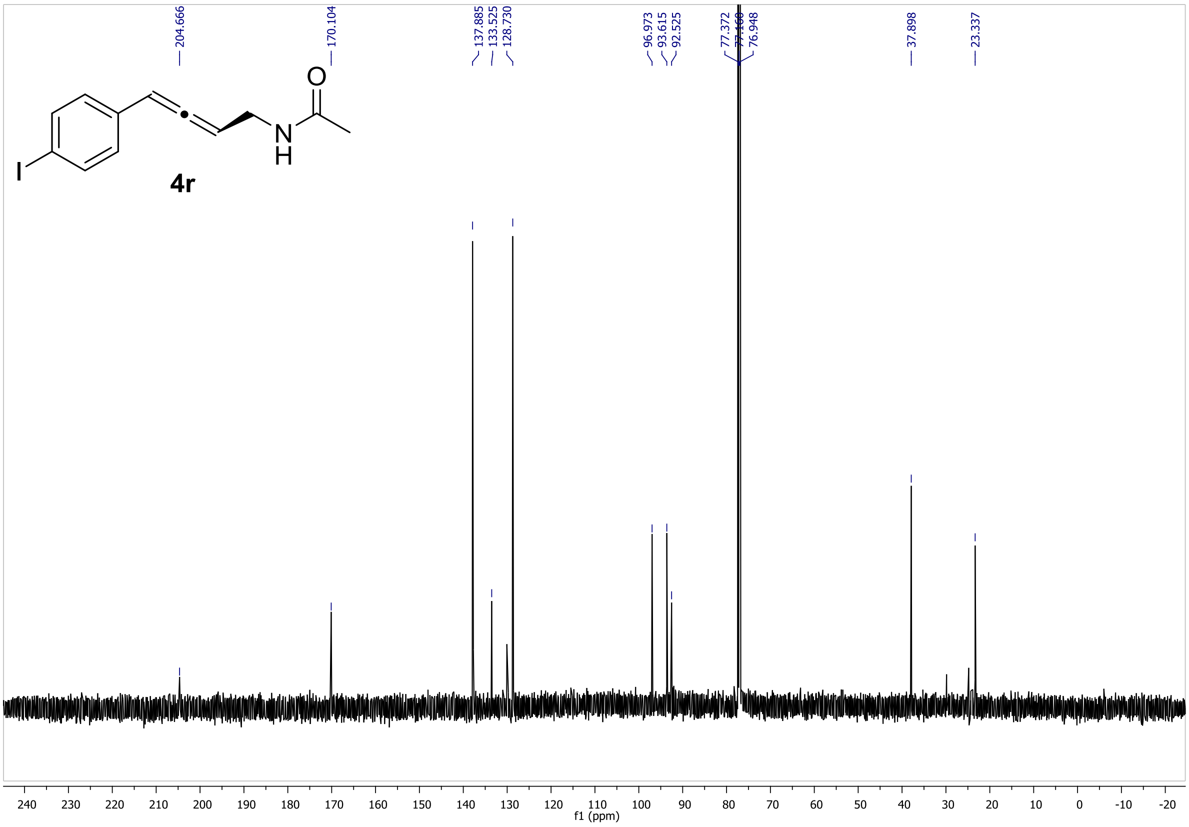

(*R*)-*N*-(4-(4-bromophenyl)buta-2,3-dien-1-yl)pent-4-enamide (4s):

^{1}H NMR, 600 MHz, $CDCl_3$:

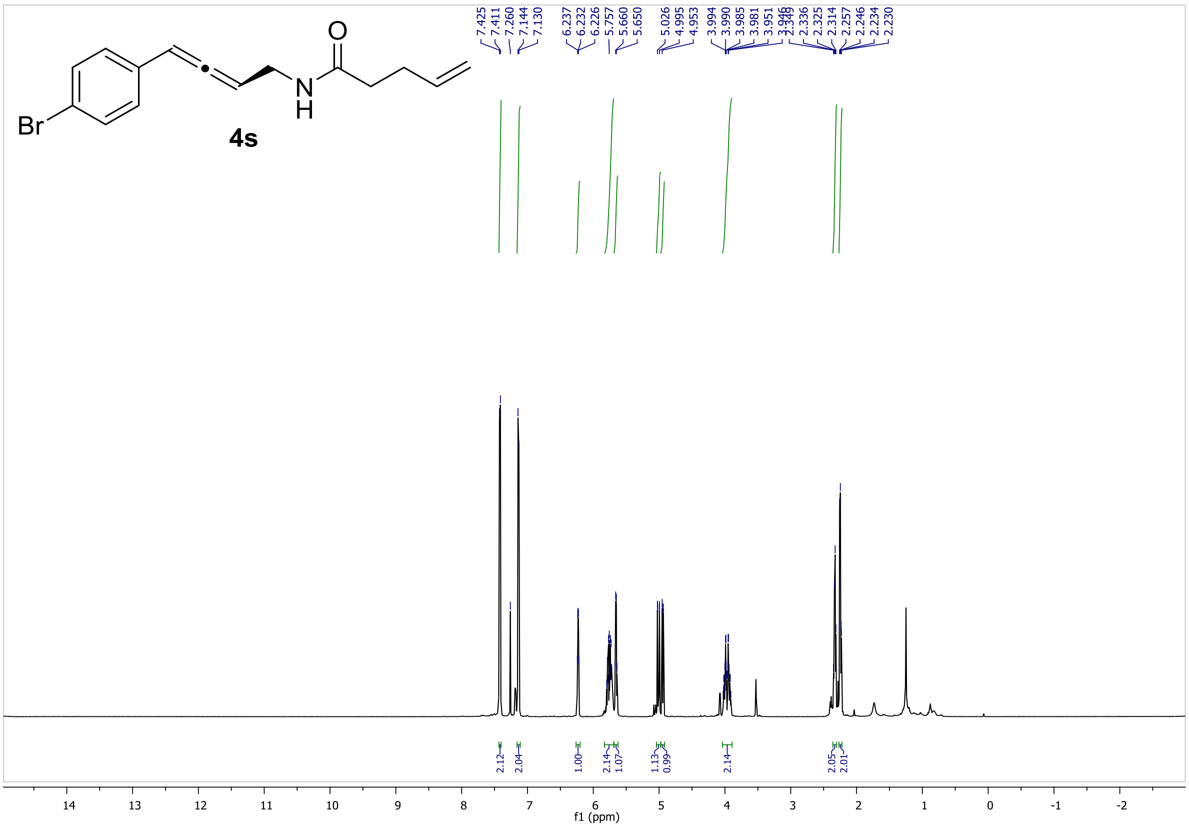

^{13}C NMR, 150 MHz, $CDCl_3$:

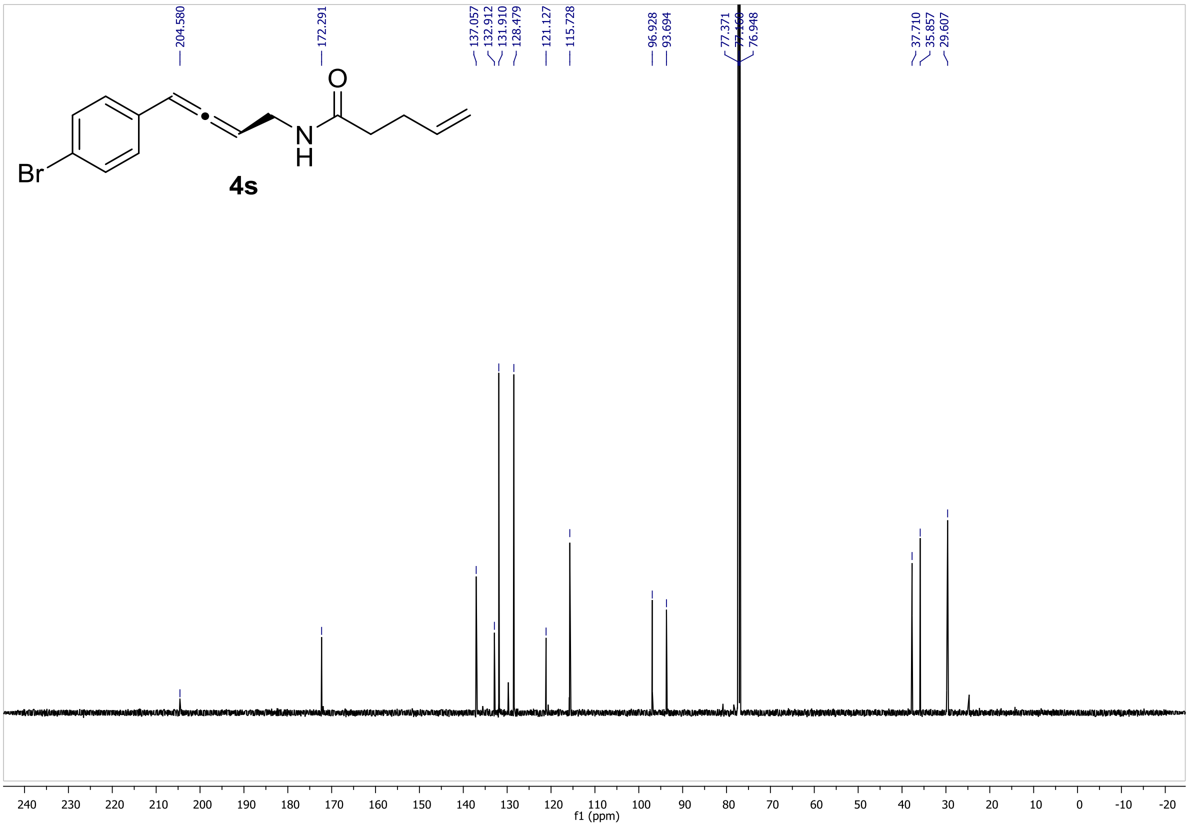

(*R*)-*N*-(4-(4-fluorophenyl)buta-2,3-dien-1-yl)pent-4-enamide (4t):

^{1}H NMR, 600 MHz, $CDCl_3$:

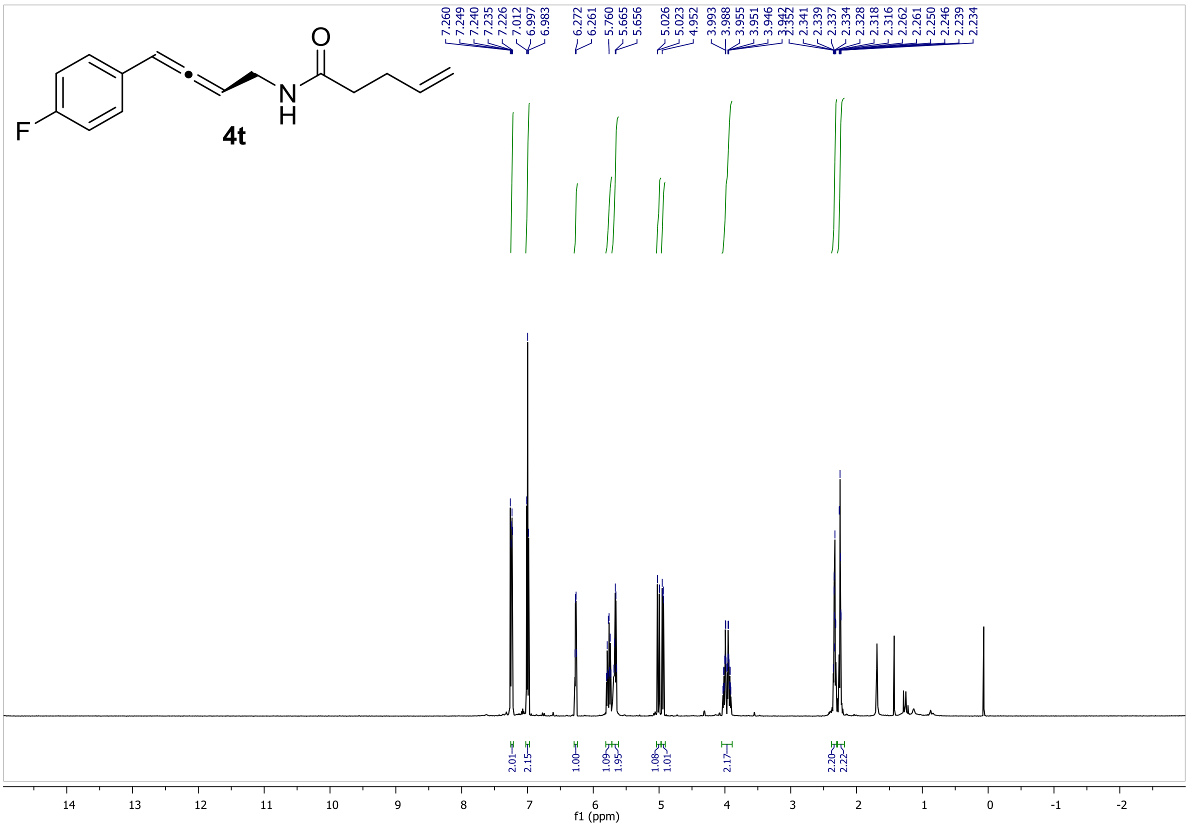

^{13}C NMR, 150 MHz, $CDCl_3$:

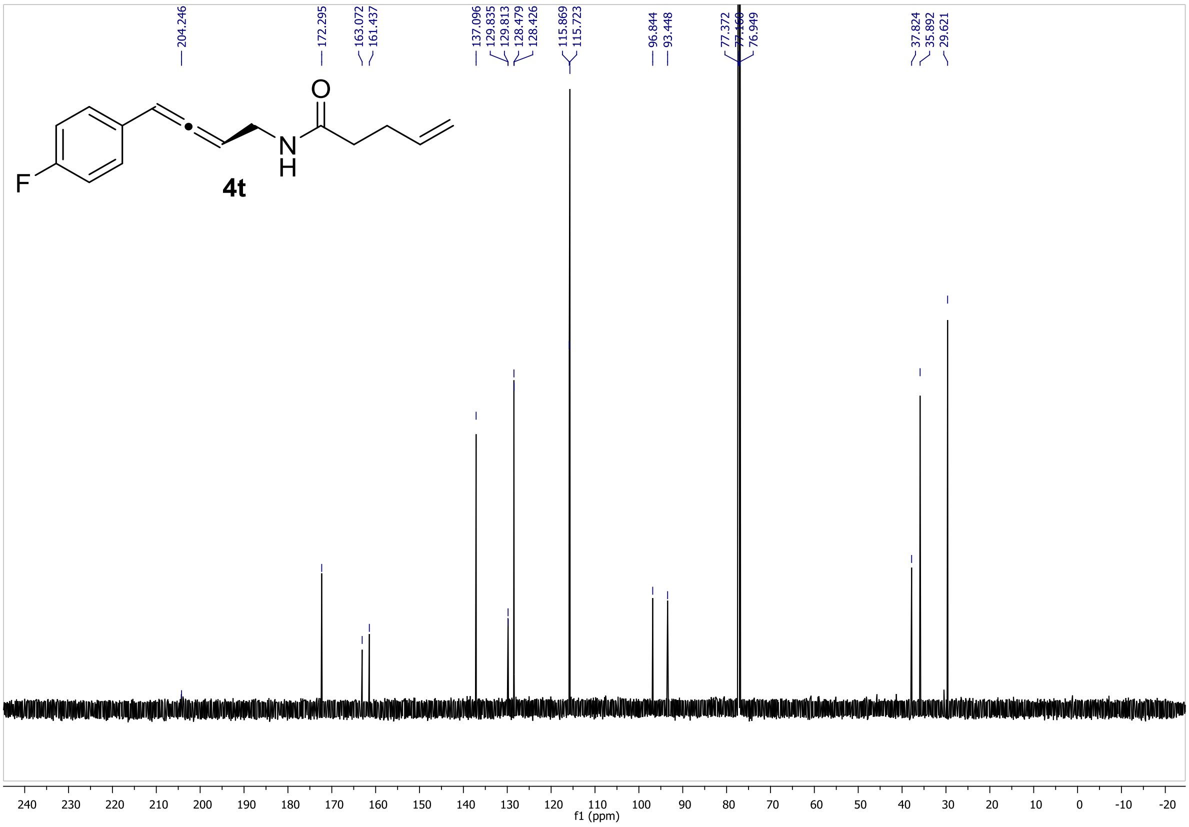

^{19}F NMR, 376 MHz, $CDCl_3$:

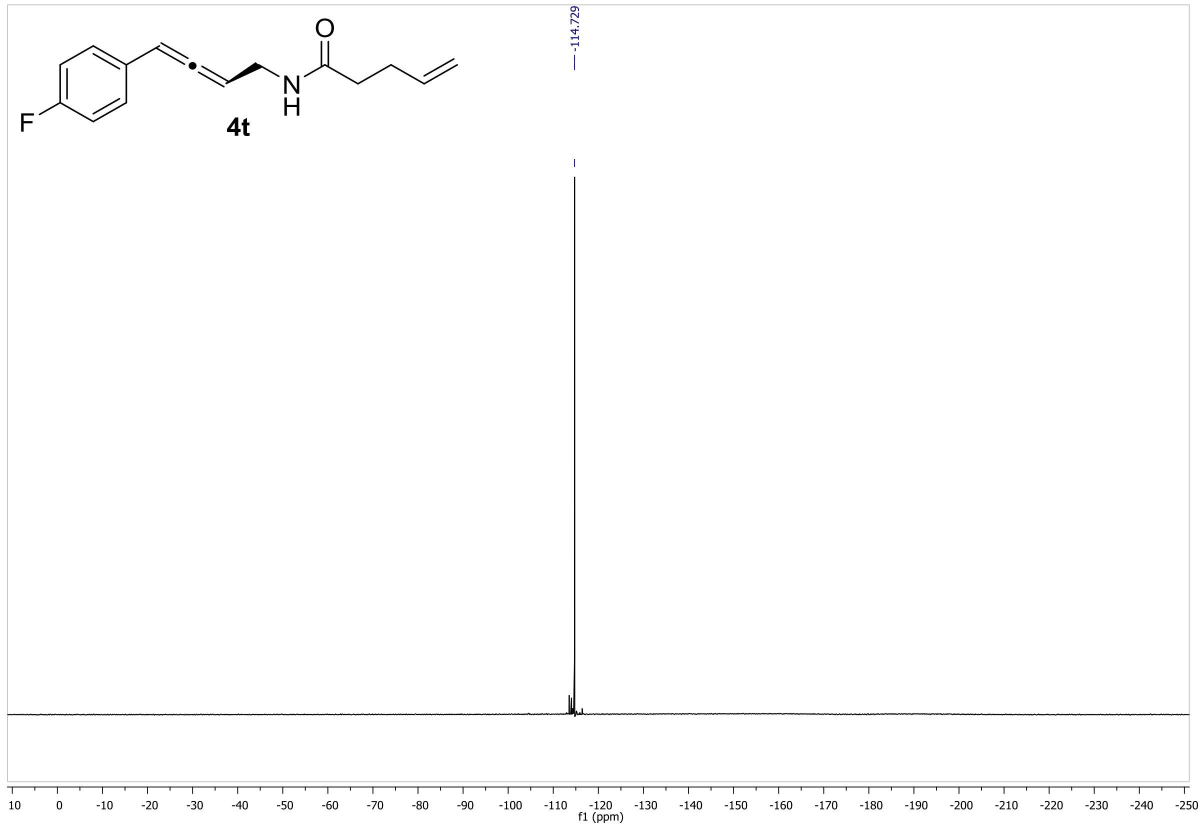

Methyl (*R*)-4-((4-(3-methoxyphenyl)buta-2,3-dien-1-yl)amino)-4-oxobutanoate (4u):

^{1}H NMR, 600 MHz, $CDCl_3$:

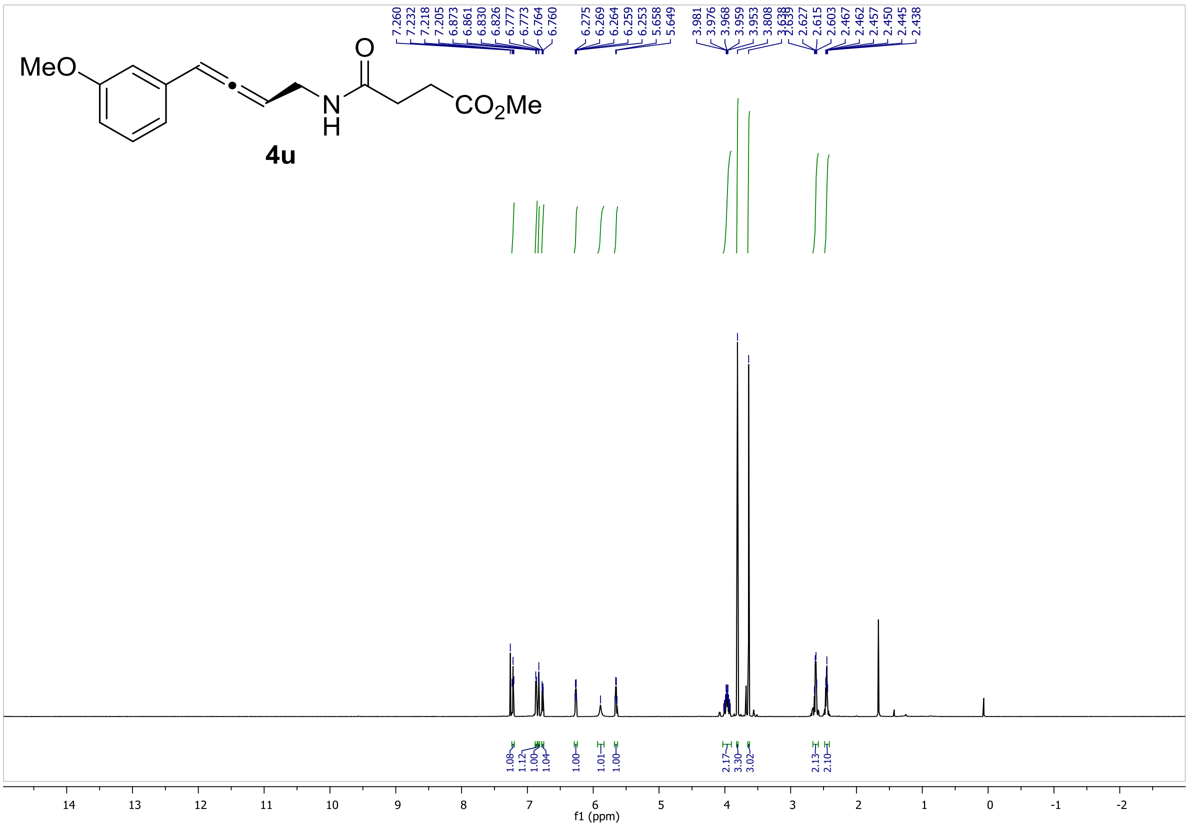

^{13}C NMR, 150 MHz, $CDCl_3$:

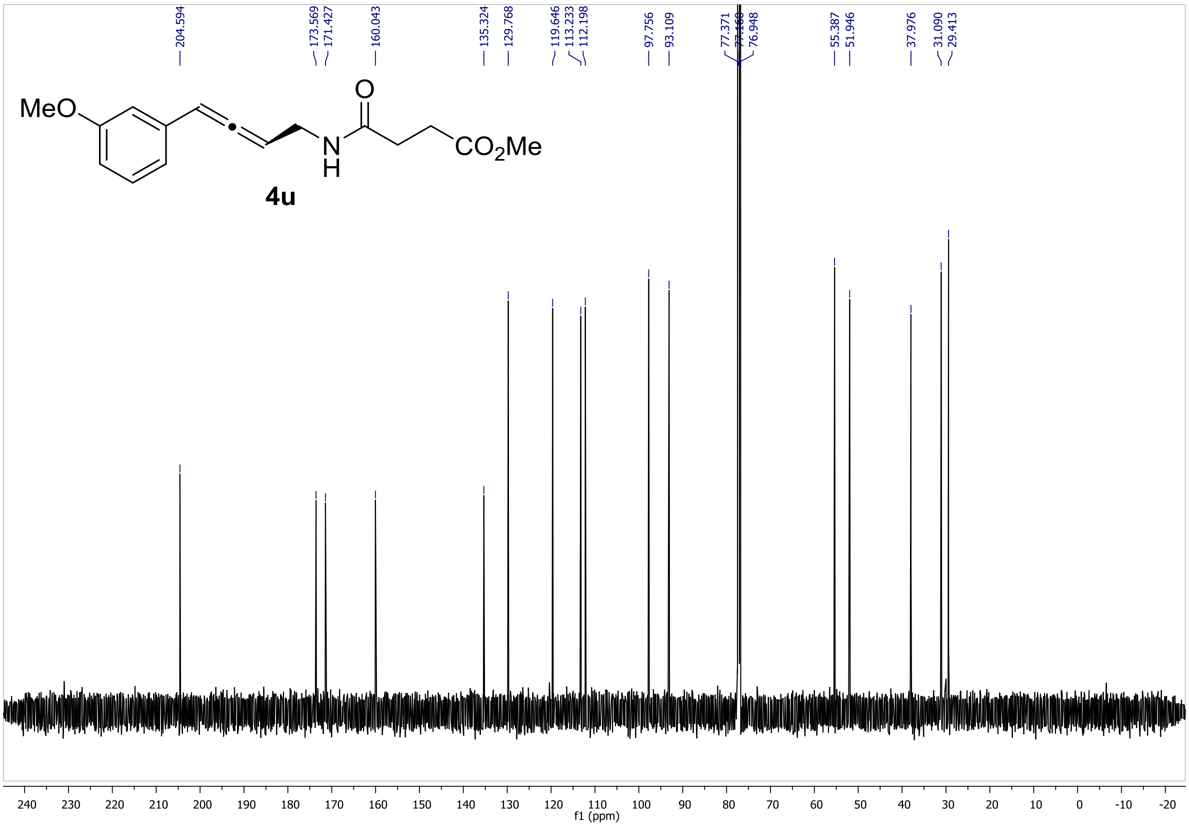

(*R*)-*N*-(4-(3-cyanophenyl)buta-2,3-dien-1-yl)cyclopent-3-ene-1-carboxamide (4v):

^{1}H NMR, 600 MHz, $CDCl_3$:

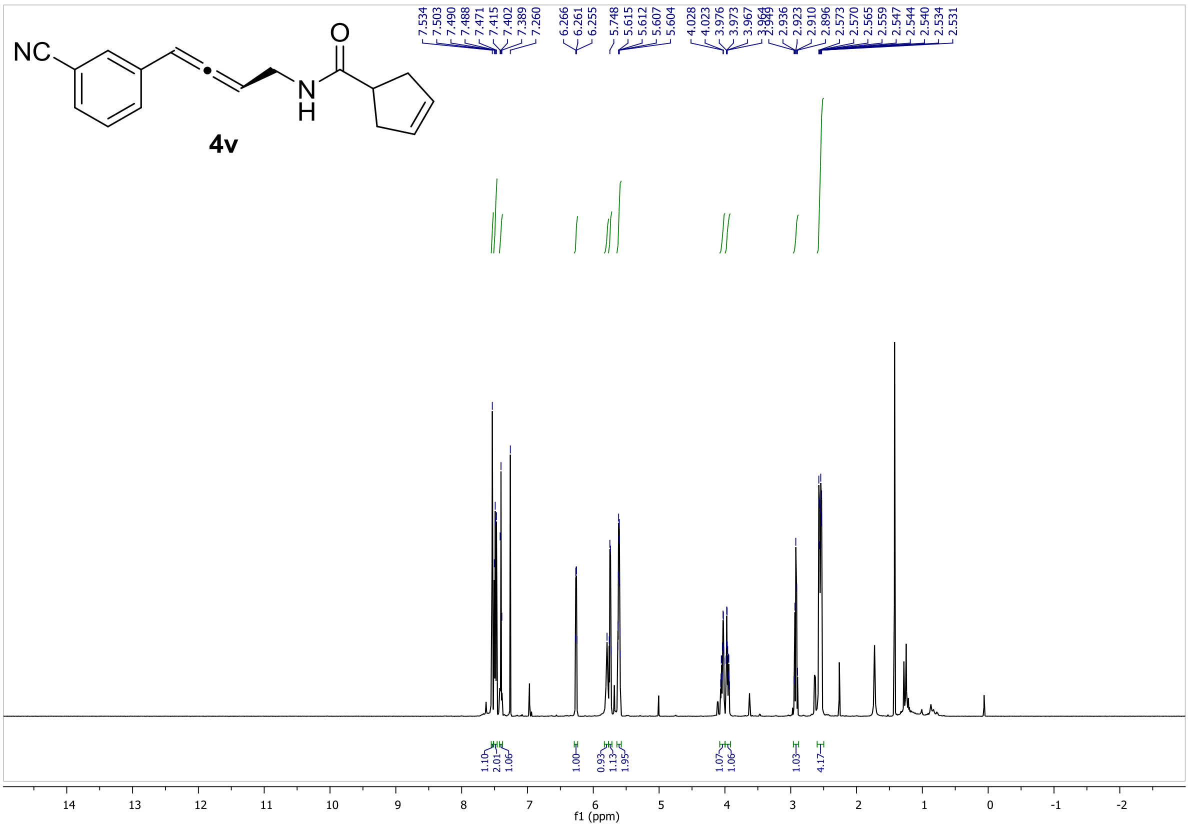

^{13}C NMR, 150 MHz, $CDCl_3$:

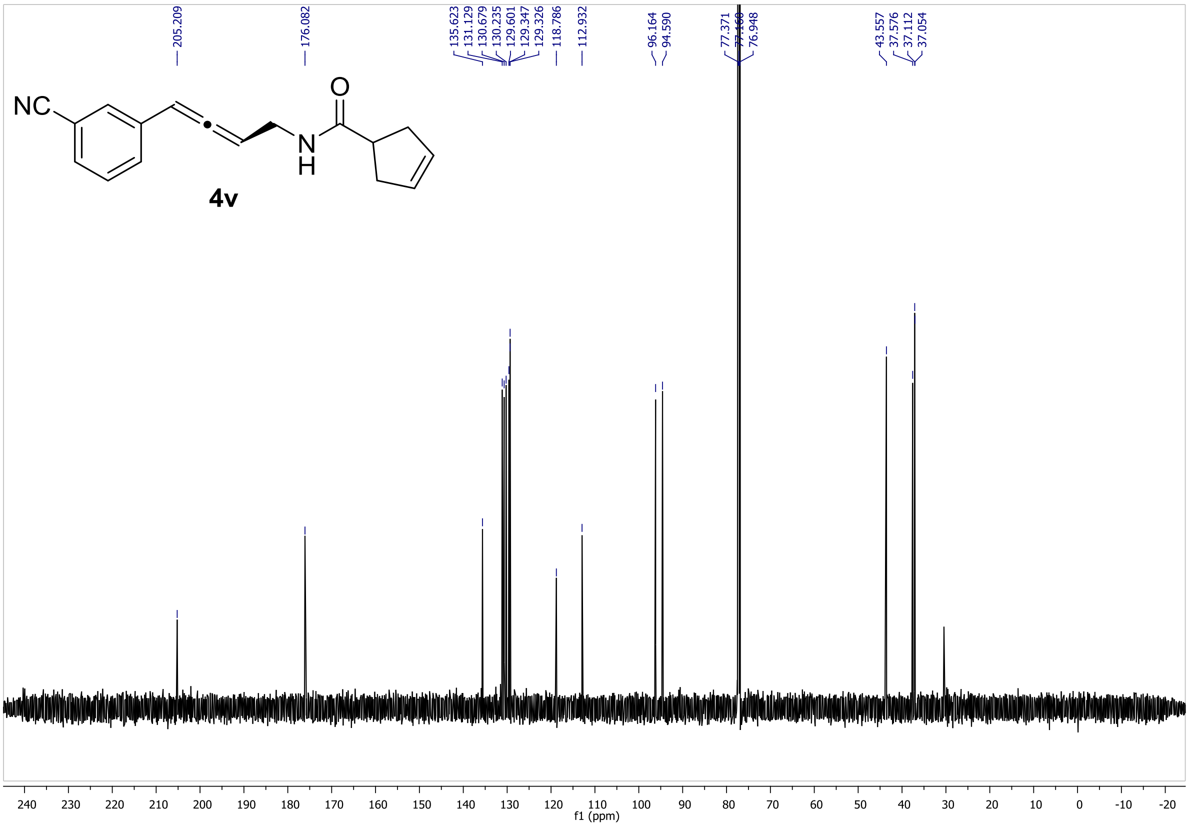

(*R*)-3,3,3-trifluoro-*N*-(4-(4-iodophenyl)buta-2,3-dien-1-yl)propanamide (4w):

^{1}H NMR, 600 MHz, $CDCl_3$:

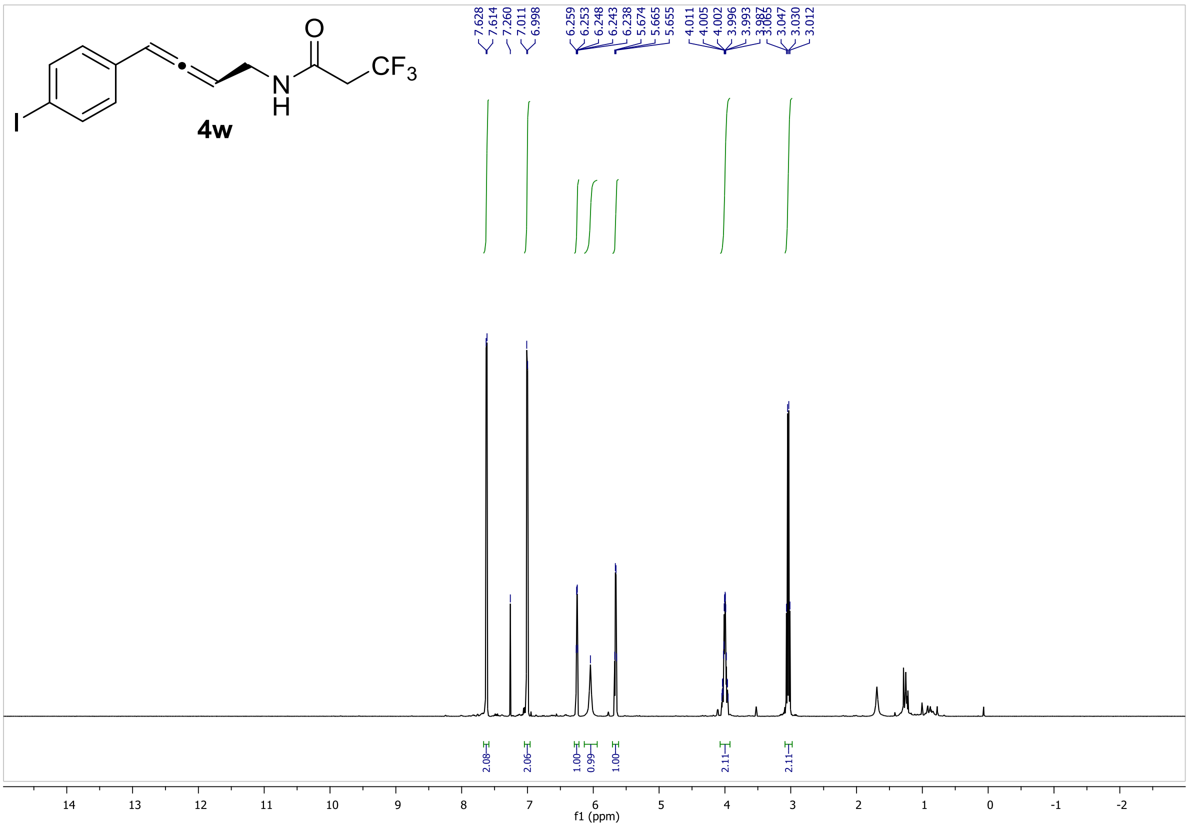

^{13}C NMR, 150 MHz, $CDCl_3$:

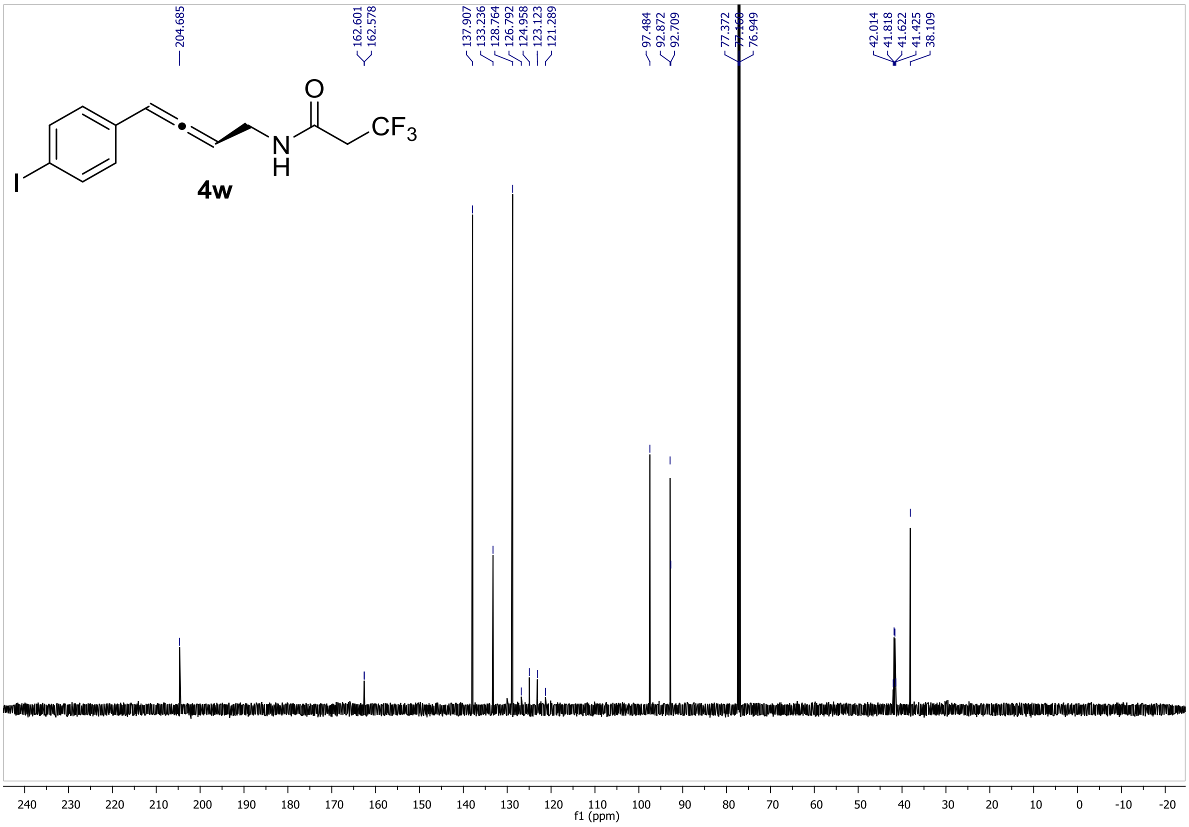

^{19}F NMR, 376 MHz, $CDCl_3$:

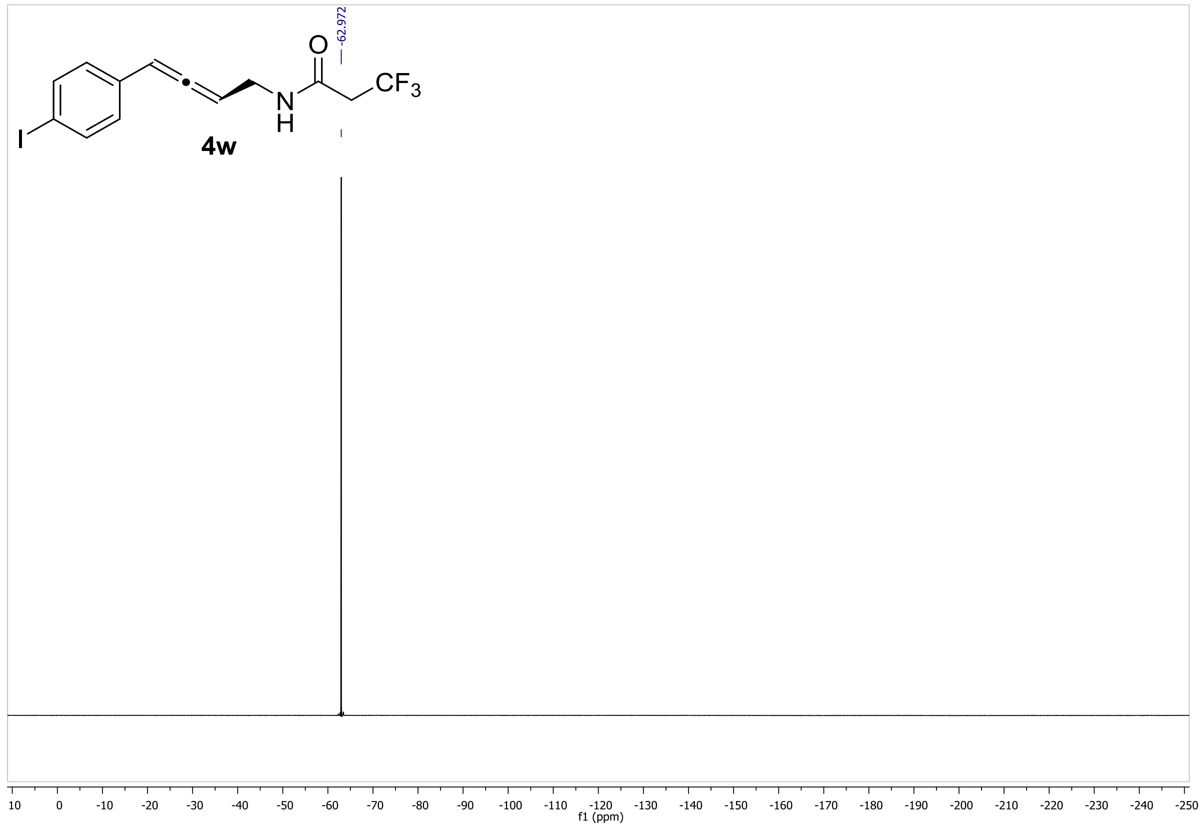

(*S*)-*N*-((*R*)-4-(4-bromophenyl)buta-2,3-dien-1-yl)-2-(4-isobutylphenyl)propanamide (4x):

^{1}H NMR, 600 MHz, $CDCl_3$:

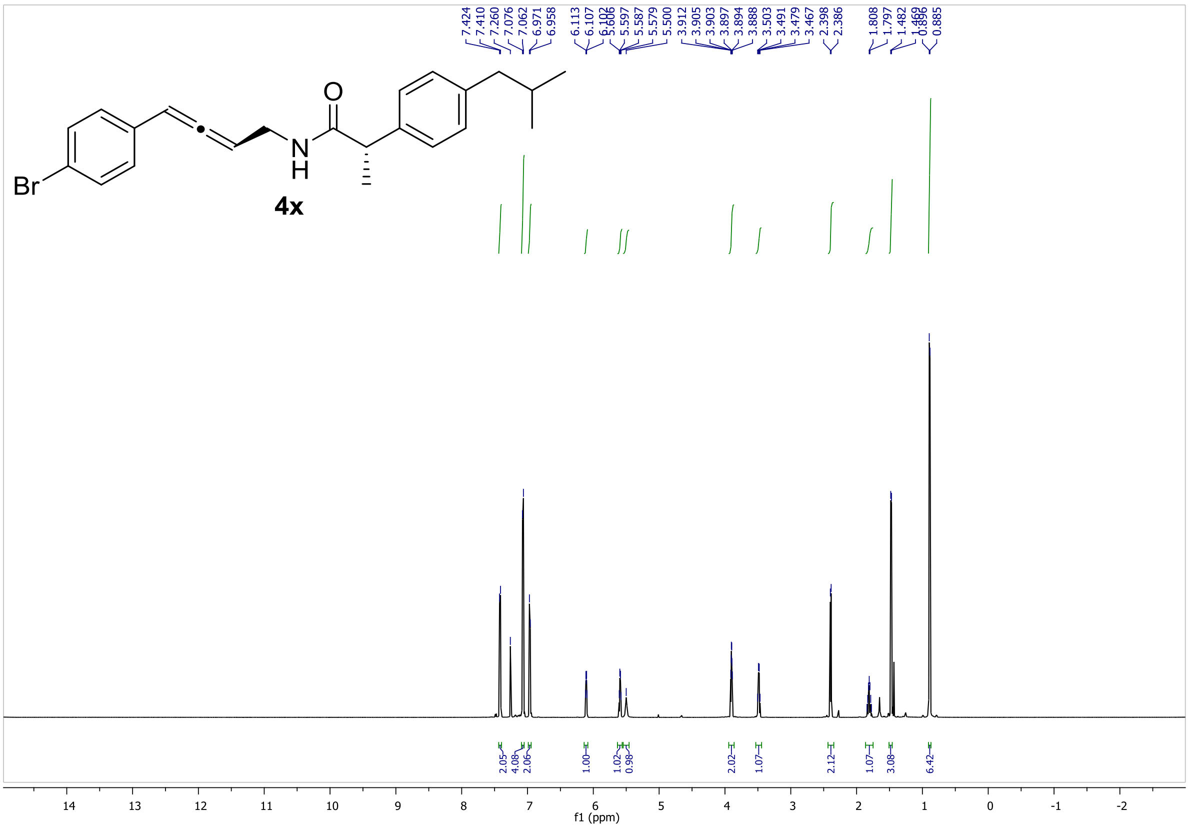

^{13}C NMR, 150 MHz, $CDCl_3$:

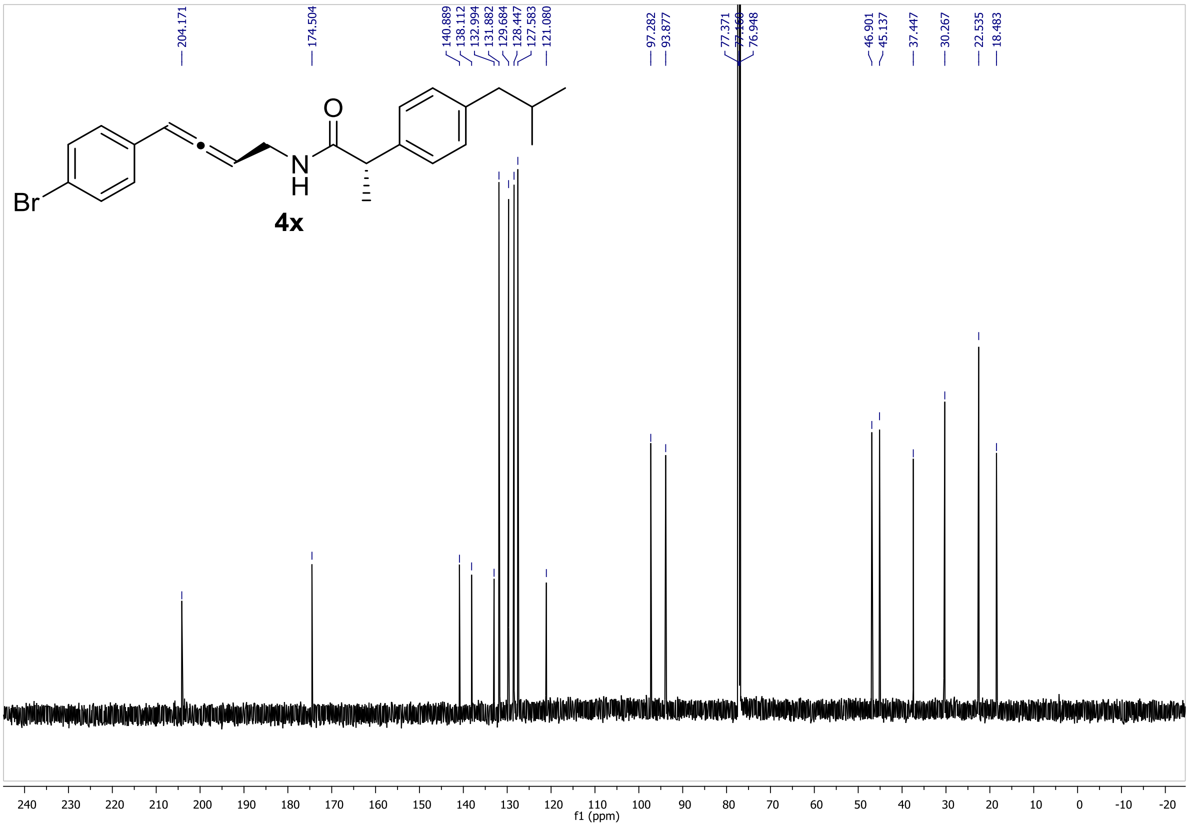

(*R*)-2-((4-(4-bromophenyl)buta-2,3-dien-1-yl)carbamoyl)phenyl acetate (4y):

^{1}H NMR, 600 MHz, $CDCl_3$:

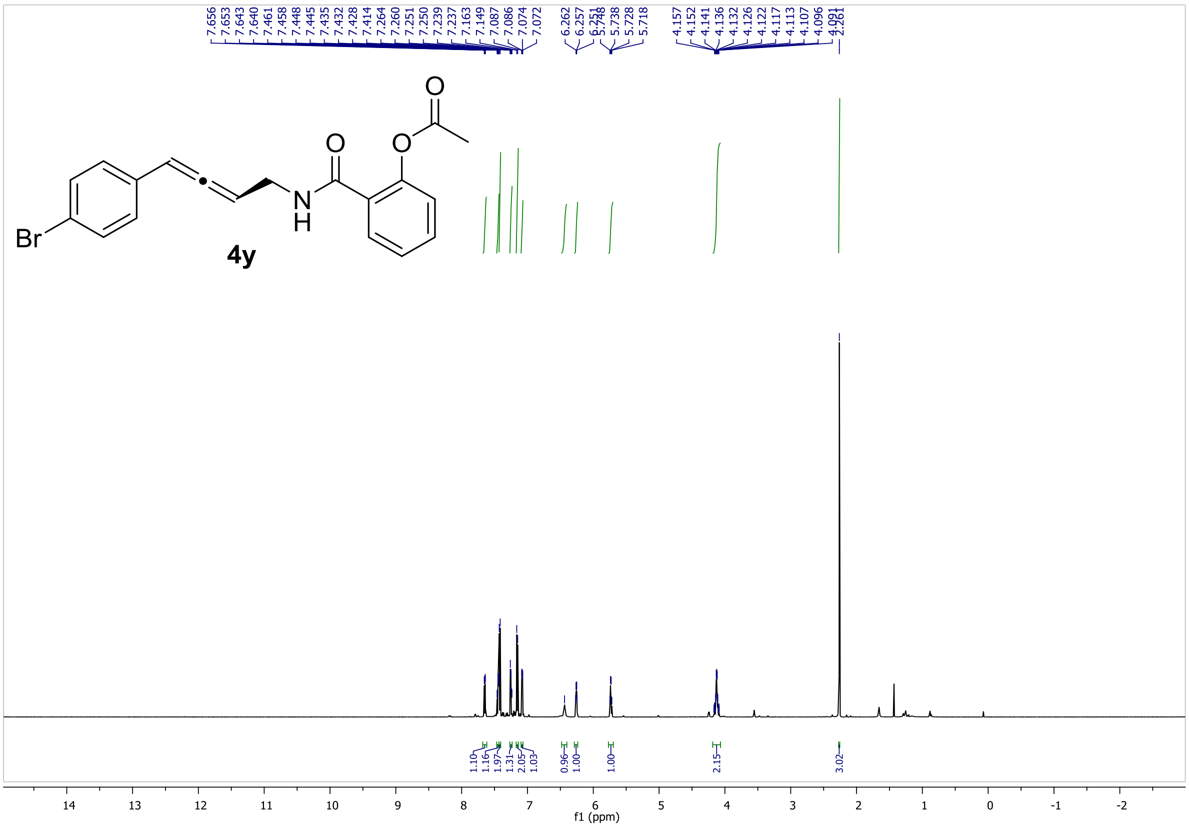

^{13}C NMR, 150 MHz, $CDCl_3$:

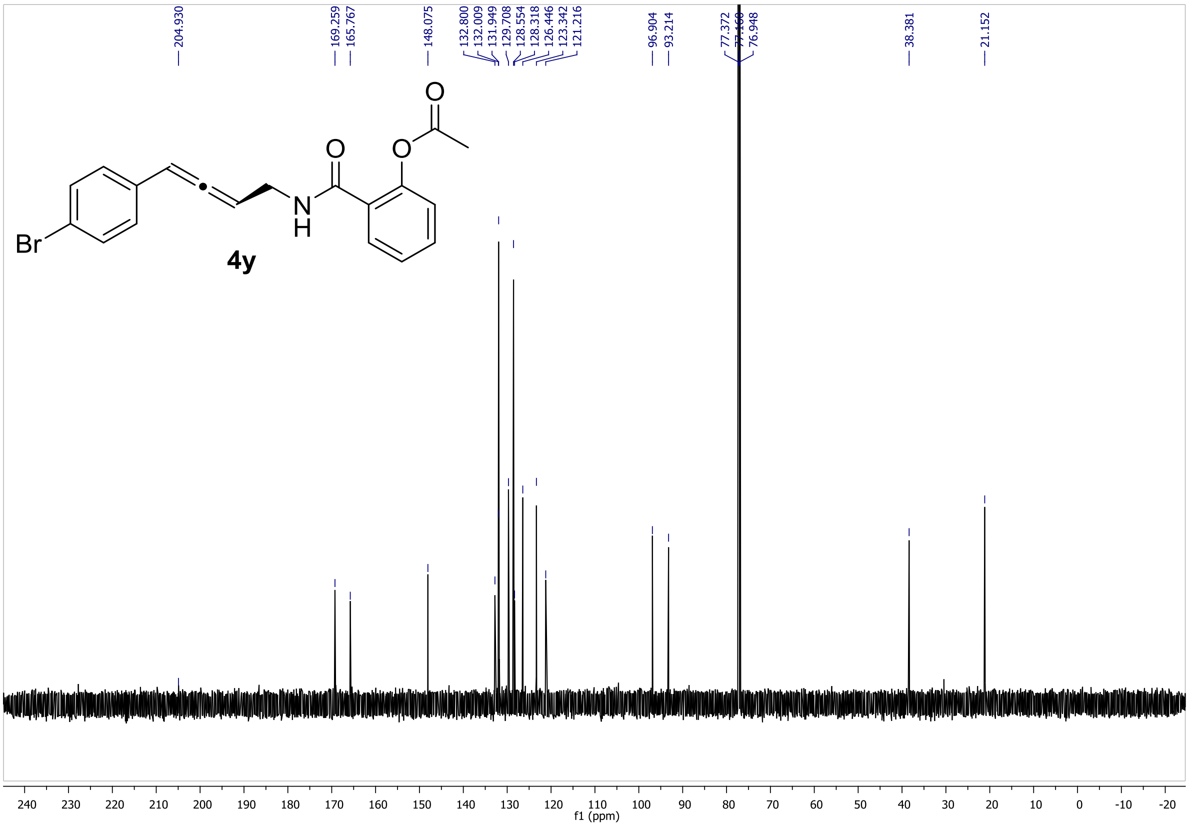

(2*S*,5*R*,6*R*)-*N*-((*R*)-4-(4-bromophenyl)buta-2,3-dien-1-yl)-3,3-dimethyl-7-oxo-6-(2-phenylacetamido)-4-thia-1-azabicyclo[3.2.0]heptane-2-carboxamide (4z):

^{1}H NMR, 600 MHz, $CDCl_3$:

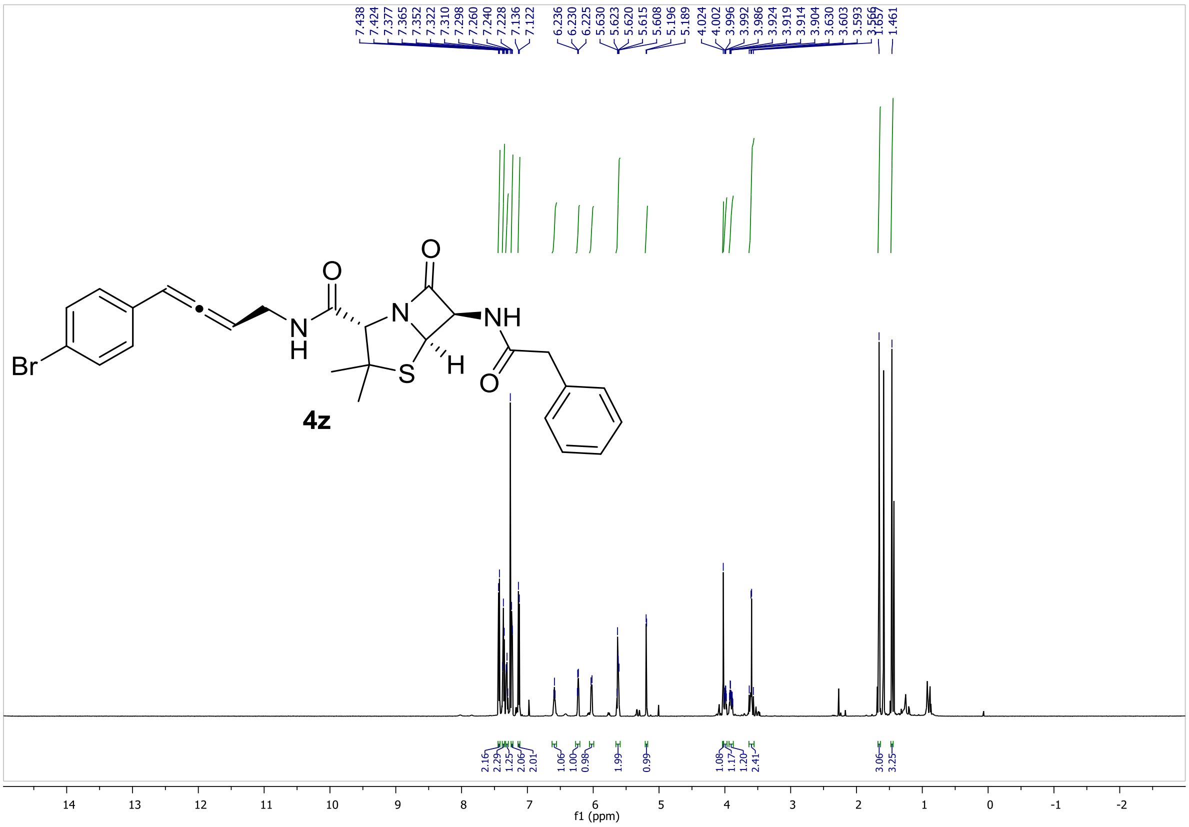

^{13}C NMR, 150 MHz, $CDCl_3$:

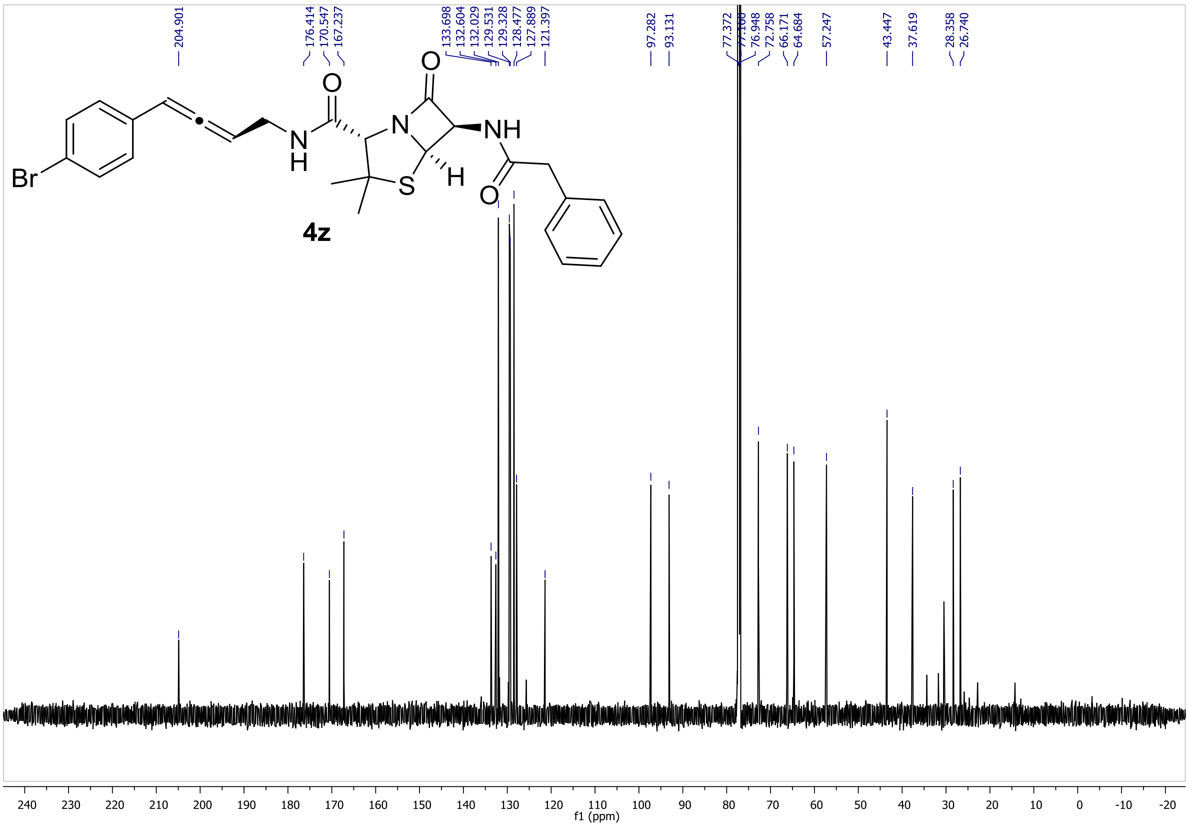

((3a*S*,5a*R*,8a*R*,8b*S*)-2,2,7,7-tetramethyltetrahydro-3a*H*-bis([1,3]dioxolo)[4,5-*b*:4',5'-*d*]pyran-3a-yl)methyl ((*R*)-4-(4-chlorophenyl)buta-2,3-dien-1-yl)sulfamate (4aa):

^{1}H NMR, 600 MHz, CDCl$_3$:

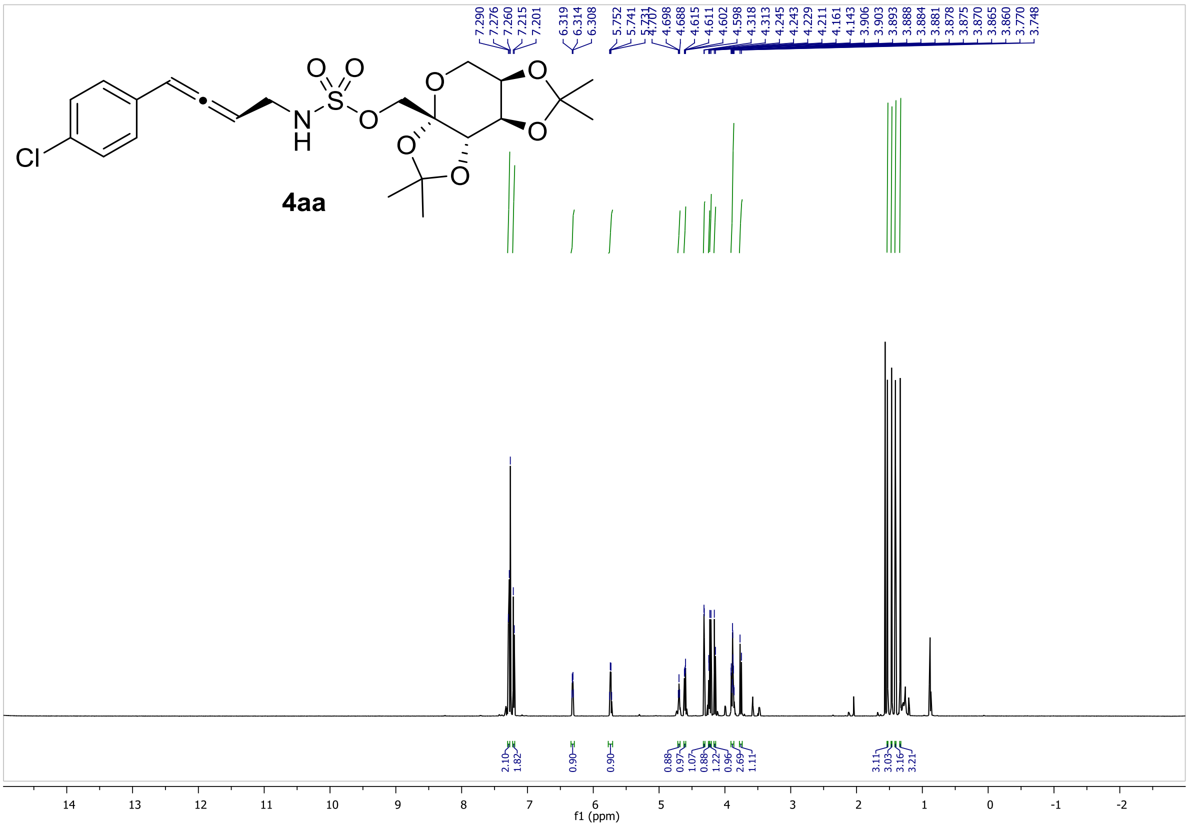

^{13}C NMR, 150 MHz, CDCl$_3$:

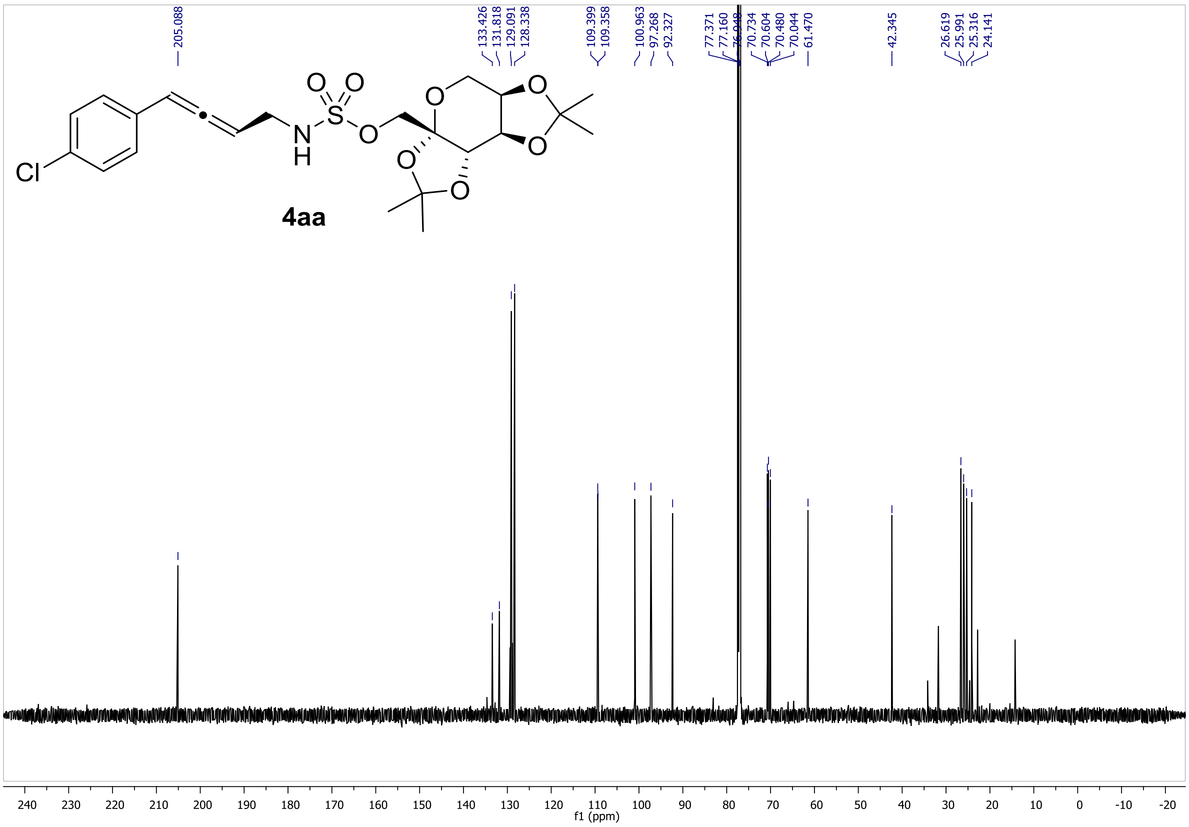

***tert*-butyl ((*S*)-2-(2-(((*R*)-4-(4-chlorophenyl)buta-2,3-dien-1-yl)amino)-2-oxoethyl)-4-methylpentyl)carbamate (4ab):**

^{1}H NMR, 600 MHz, $CDCl_3$:

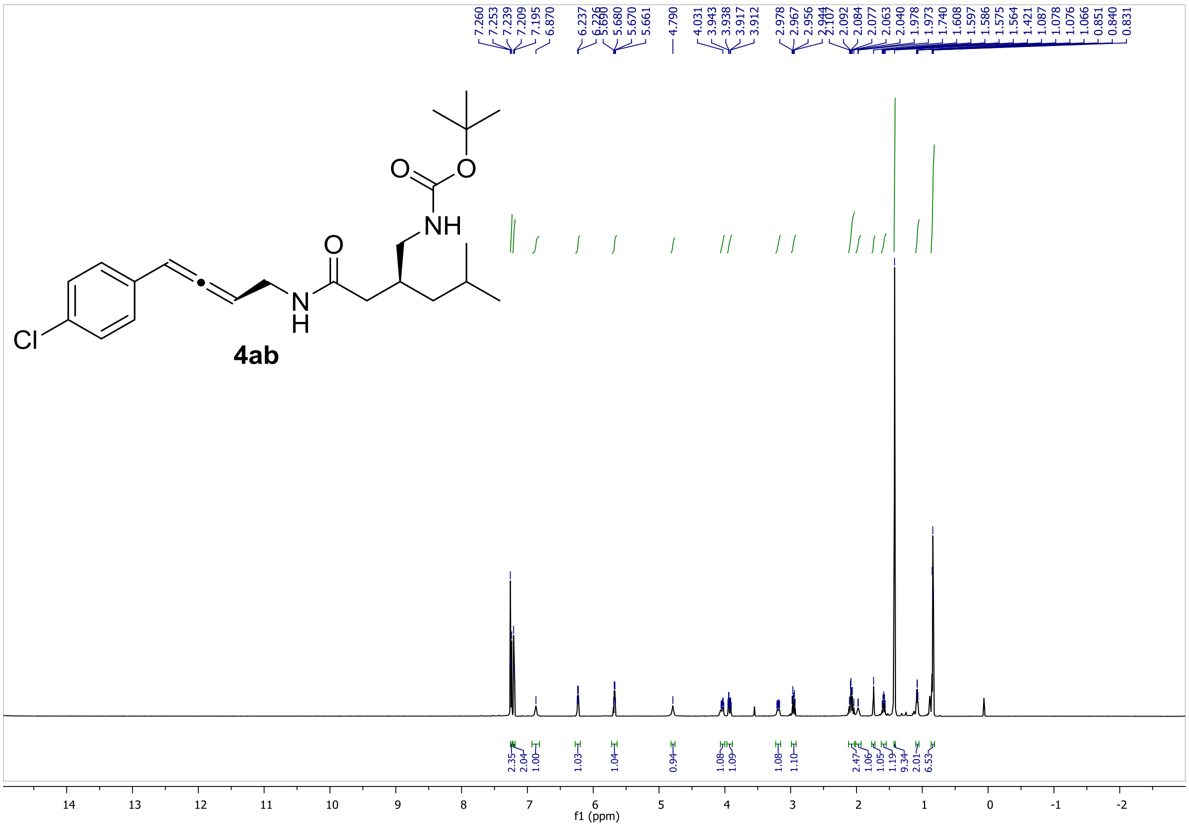

^{13}C NMR, 150 MHz, $CDCl_3$:

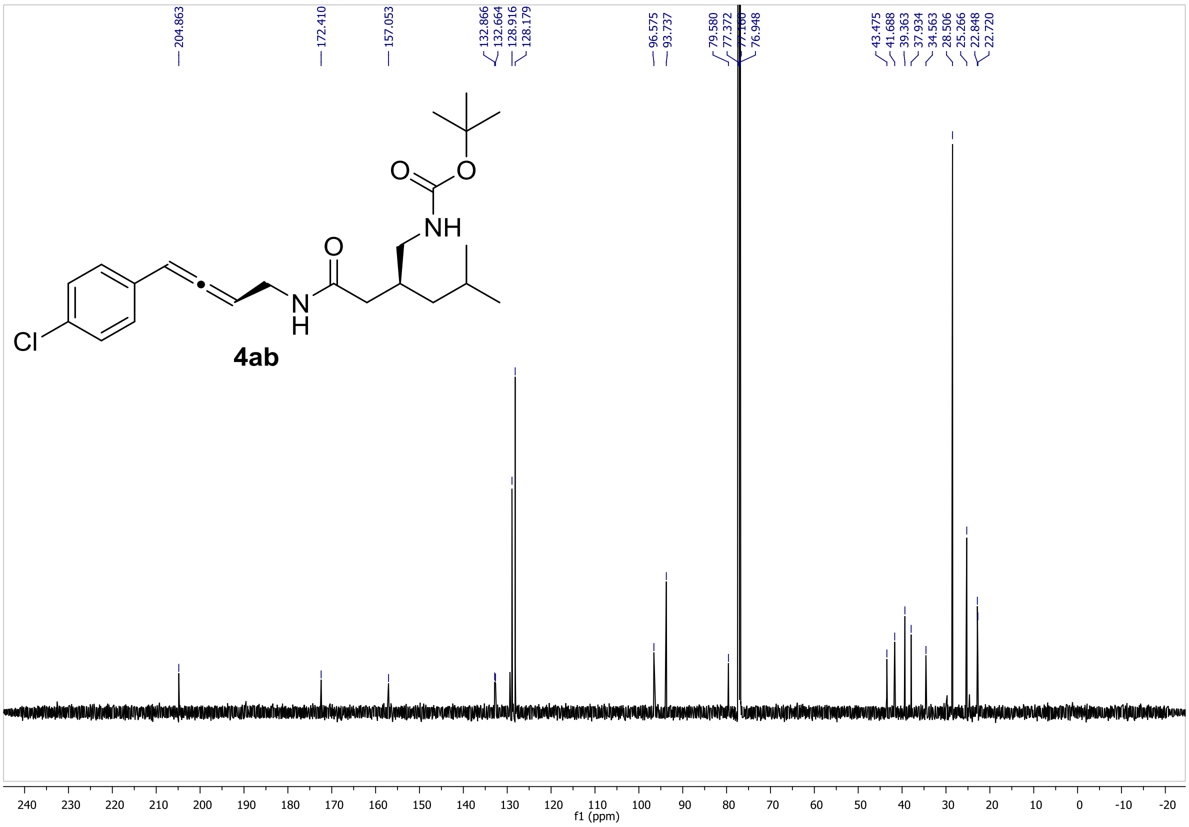

(*R*)-2-(1-(4-chlorobenzoyl)-5-methoxy-2-methyl-1*H*-indol-3-yl)-*N*-(4-(4-chloro-phenyl)buta-2,3-dien-1-yl)acetamide (4ac):

^{1}H NMR, 600 MHz, CDCl$_3$:

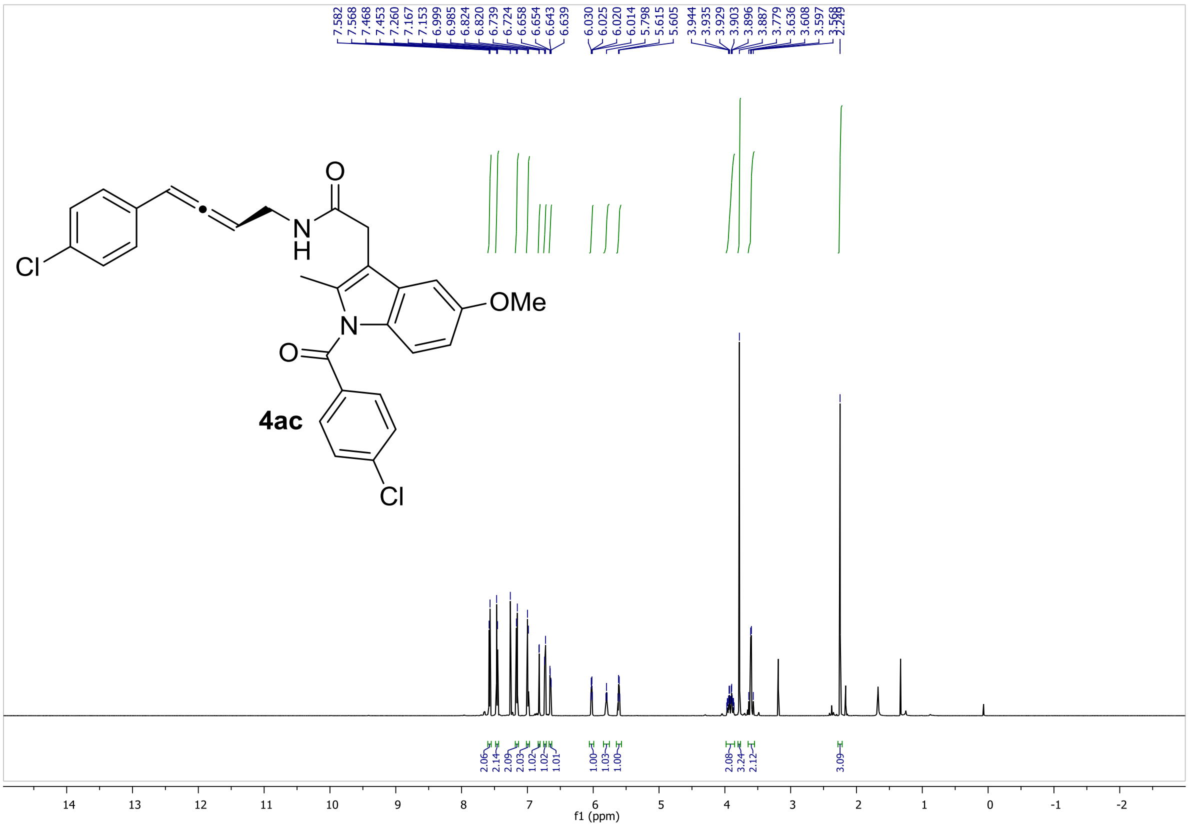

^{13}C NMR, 150 MHz, CDCl$_3$:

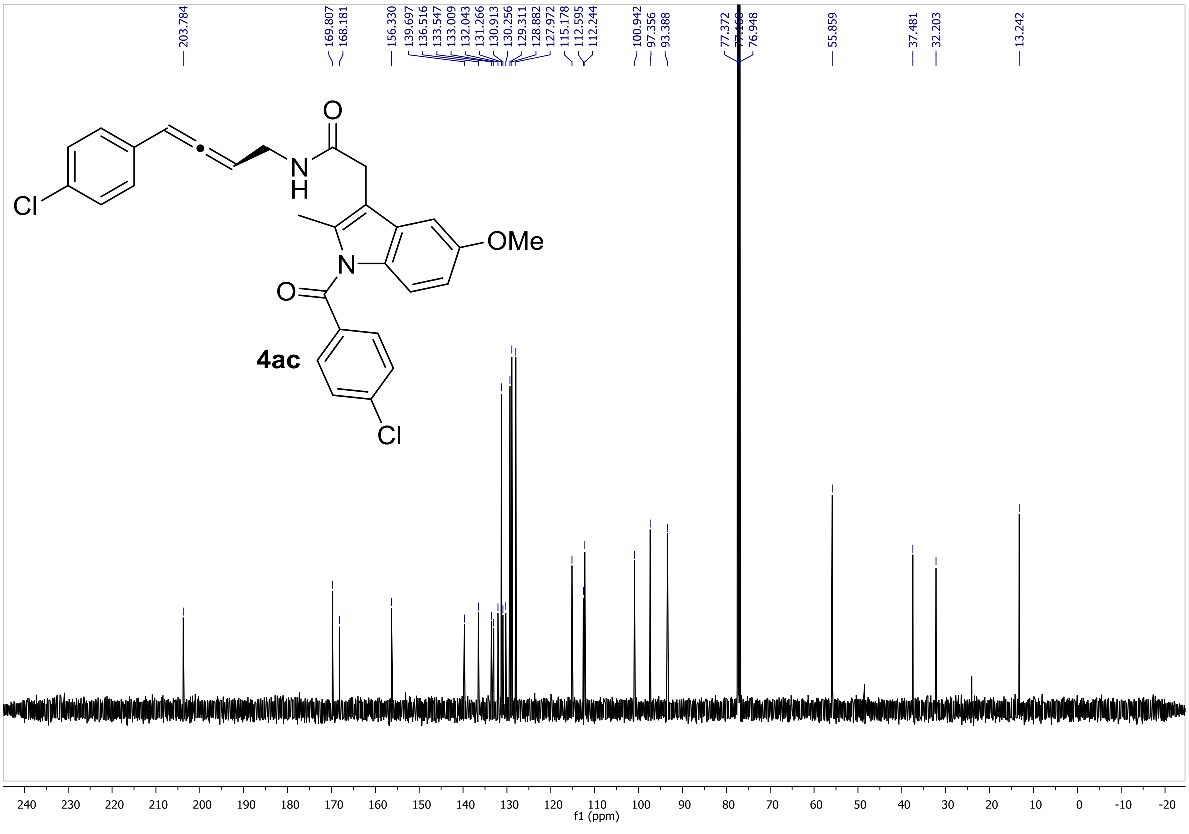

1-(2-((4*R*,6*R*)-6-(2-(((*R*)-4-(4-bromophenyl)buta-2,3-dien-1-yl)amino)-2-oxoethyl)-2,2-dimethyl-1,3-dioxan-4-yl)ethyl)-5-(4-fluorophenyl)-2-isopropyl-*N*,4-diphenyl-1*H*-pyrrole-3-carboxamide (4ad):

^{1}H NMR, 600 MHz, $CDCl_3$:

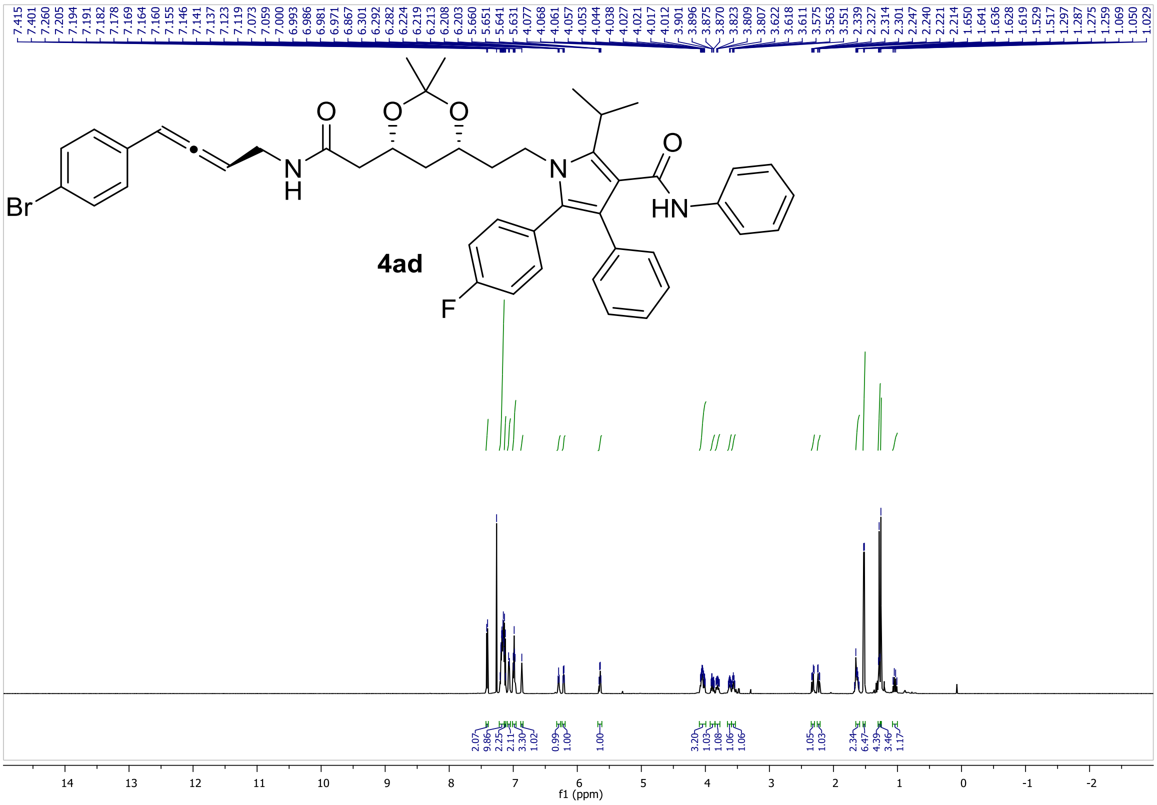

^{13}C NMR, 150 MHz, $CDCl_3$:

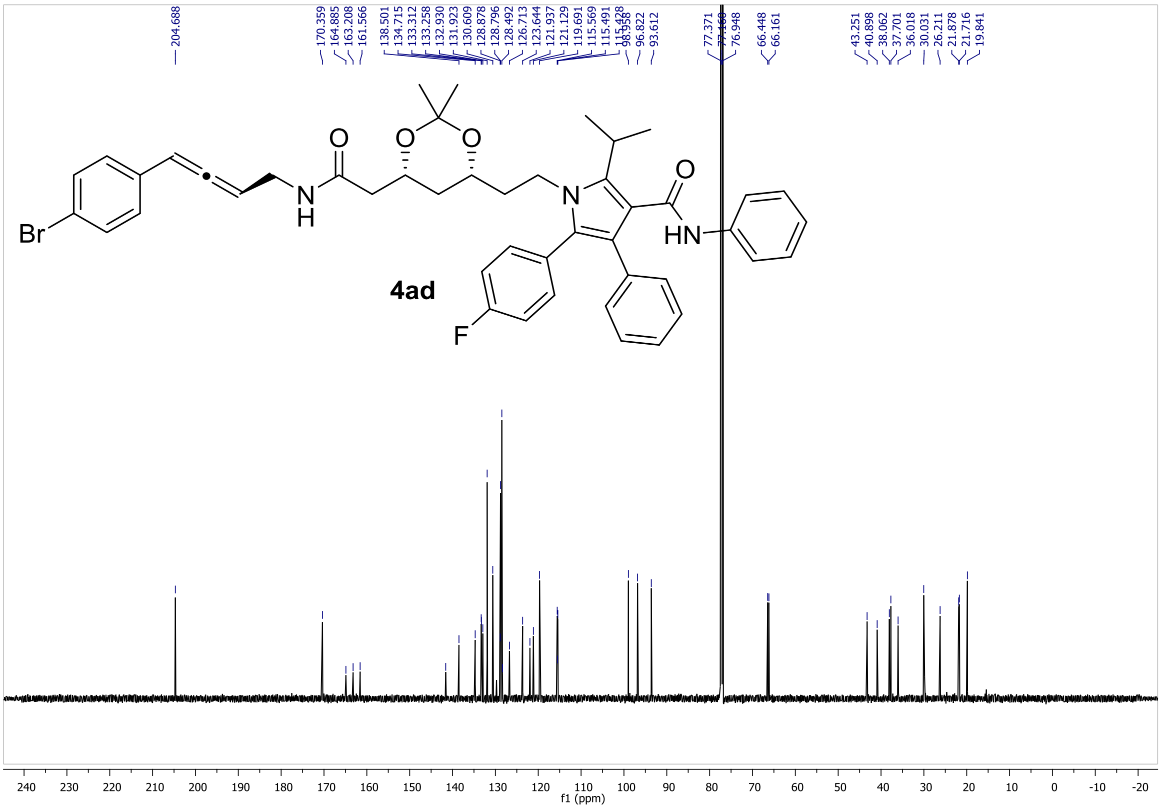

^{19}F NMR, 376 MHz, CDCl$_3$:

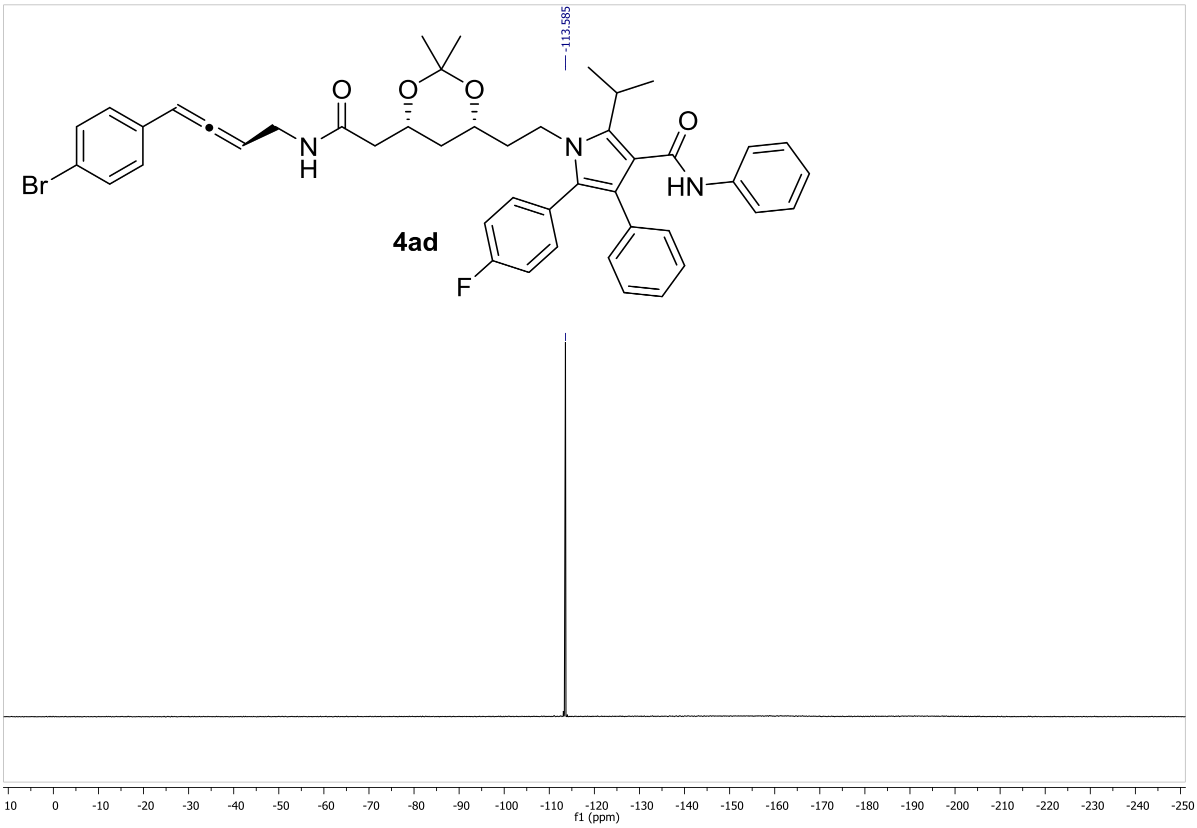

(*R*)-1-chloro-4-(3-cyclopropylpropa-1,2-dien-1-yl)benzene:

^{1}H NMR, 600 MHz, $CDCl_3$:

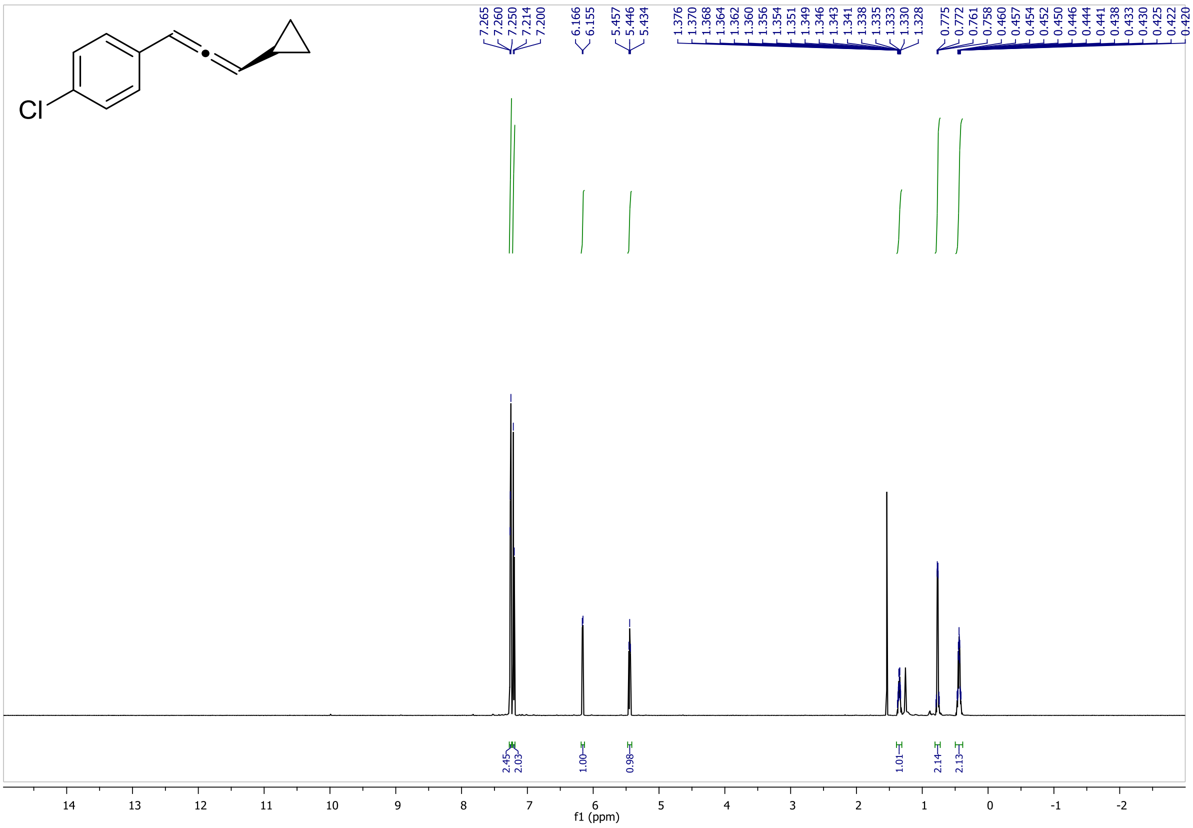

^{13}C NMR, 150 MHz, $CDCl_3$:

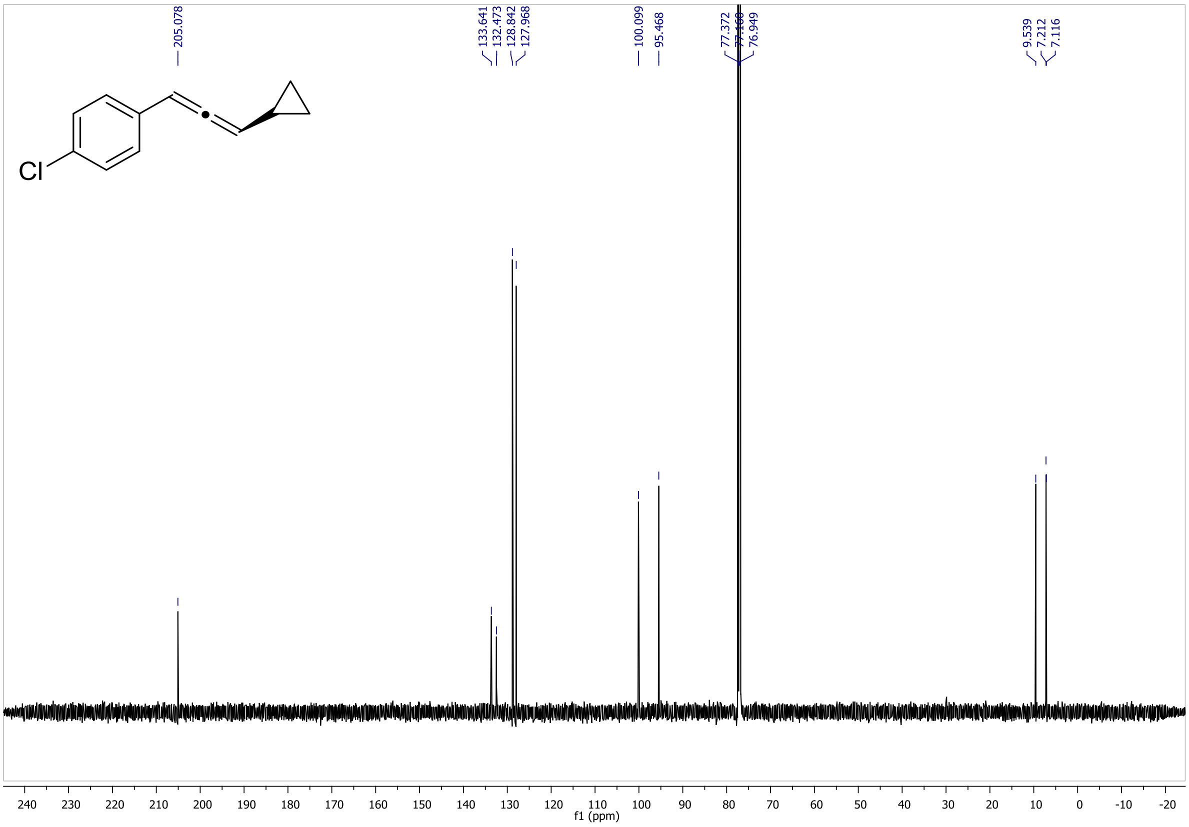

8.6. NMR spectra for alkyne cross-products

N-(4-(4-chlorophenyl)but-2-yn-1-yl)-4-methylbenzenesulfonamide (4a'):

^{1}H NMR, 600 MHz, $CDCl_3$:

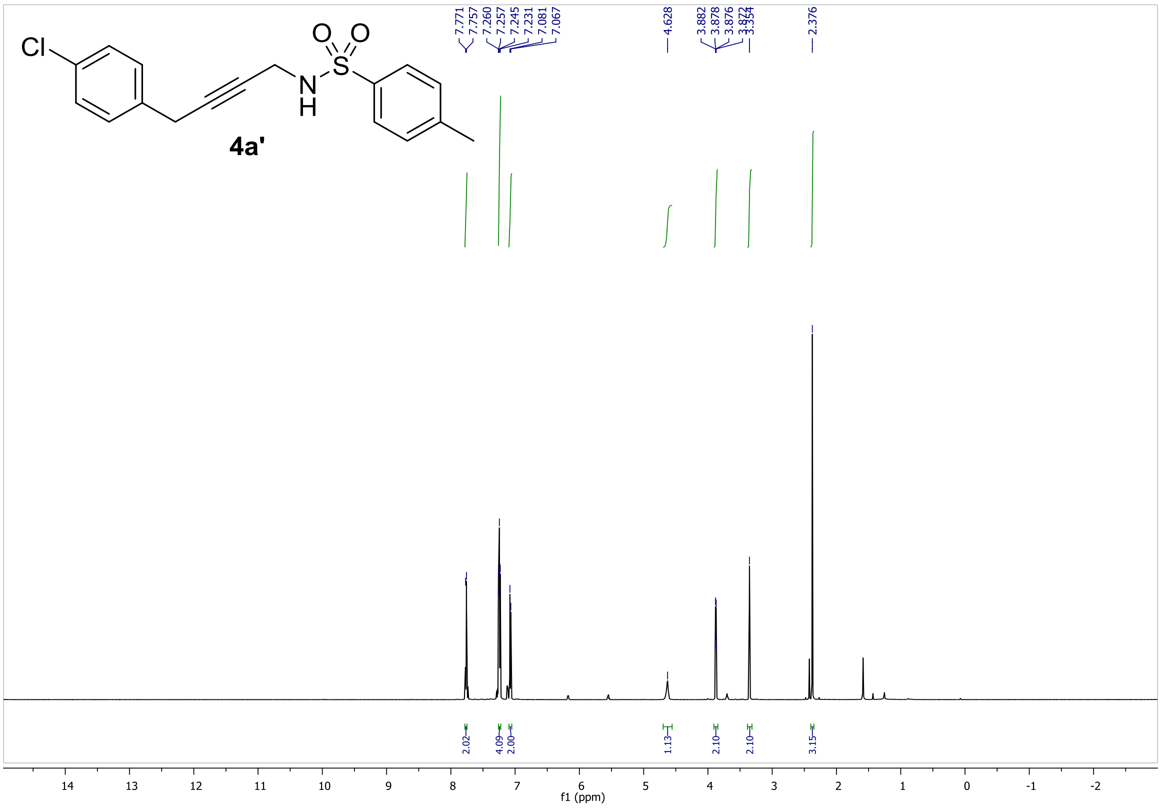

^{13}C NMR, 150 MHz, $CDCl_3$:

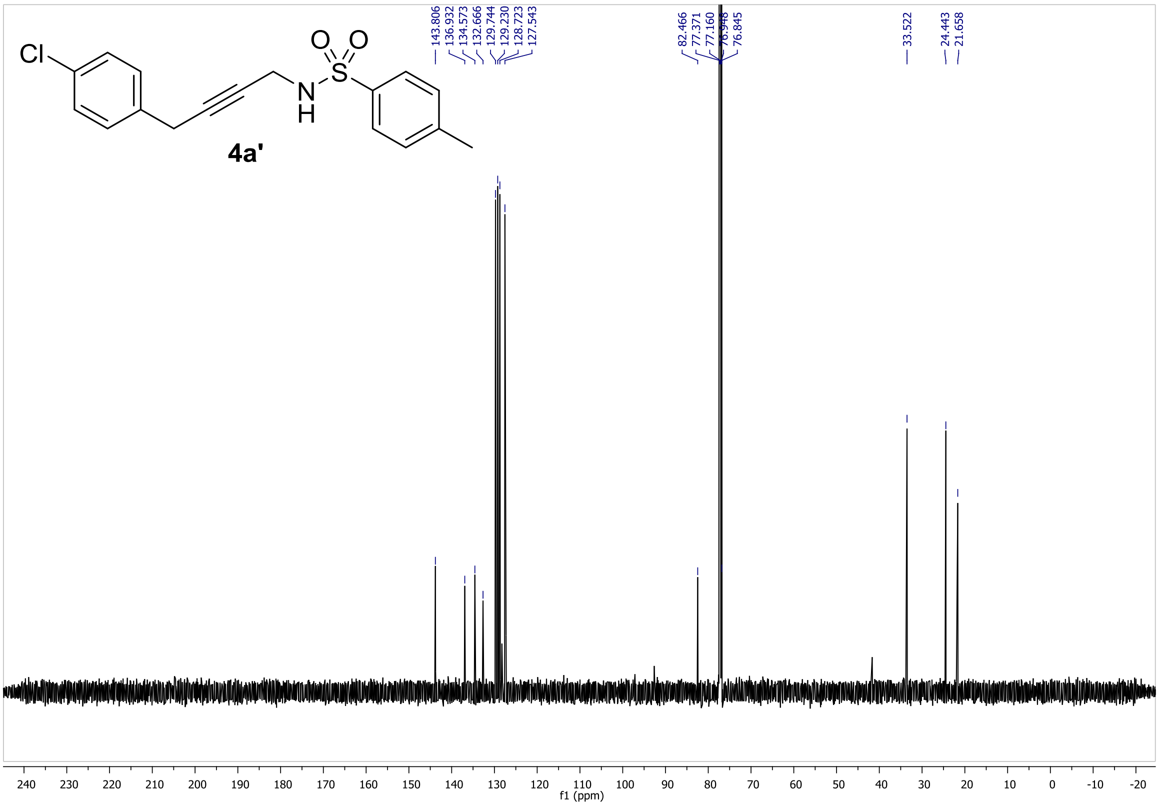

N-(4-(4-chlorophenyl)but-2-yn-1-yl)-3,3,3-trifluoropropanamide (4b'):

^{1}H NMR, 600 MHz, $CDCl_3$:

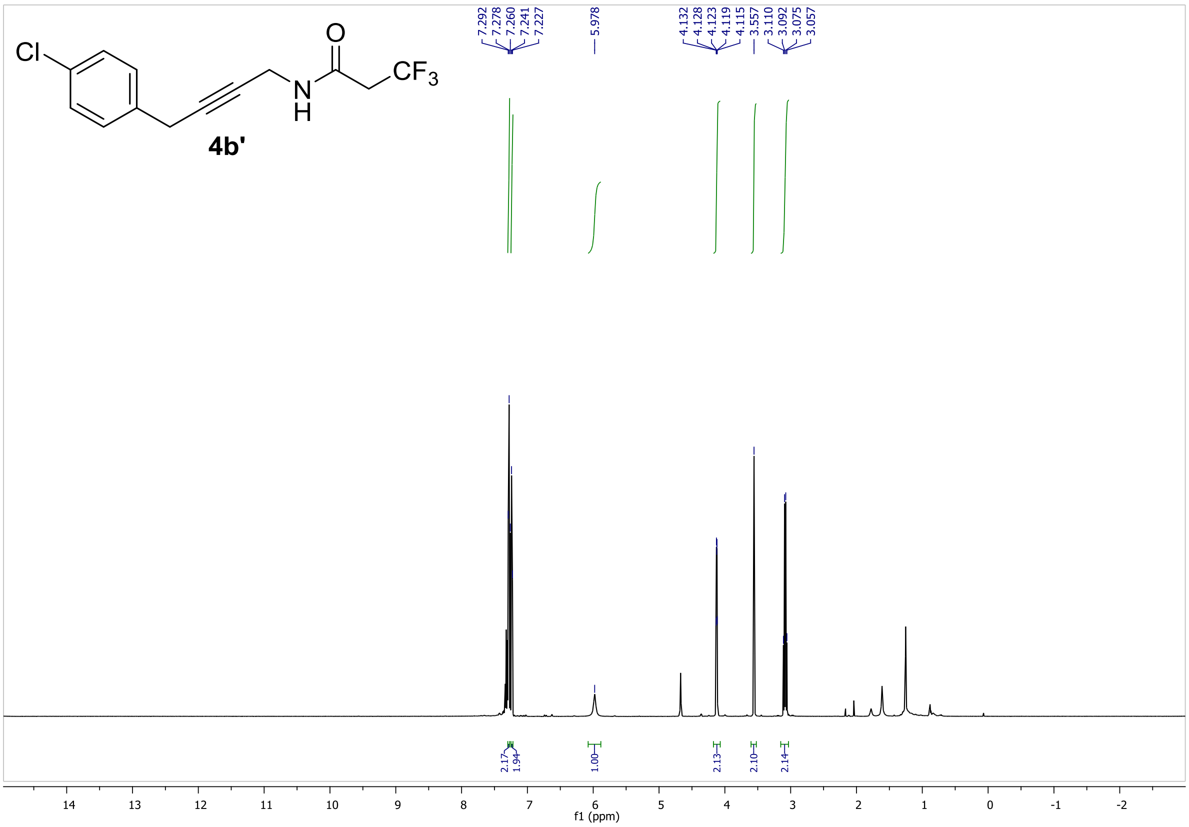

^{13}C NMR, 150 MHz, $CDCl_3$:

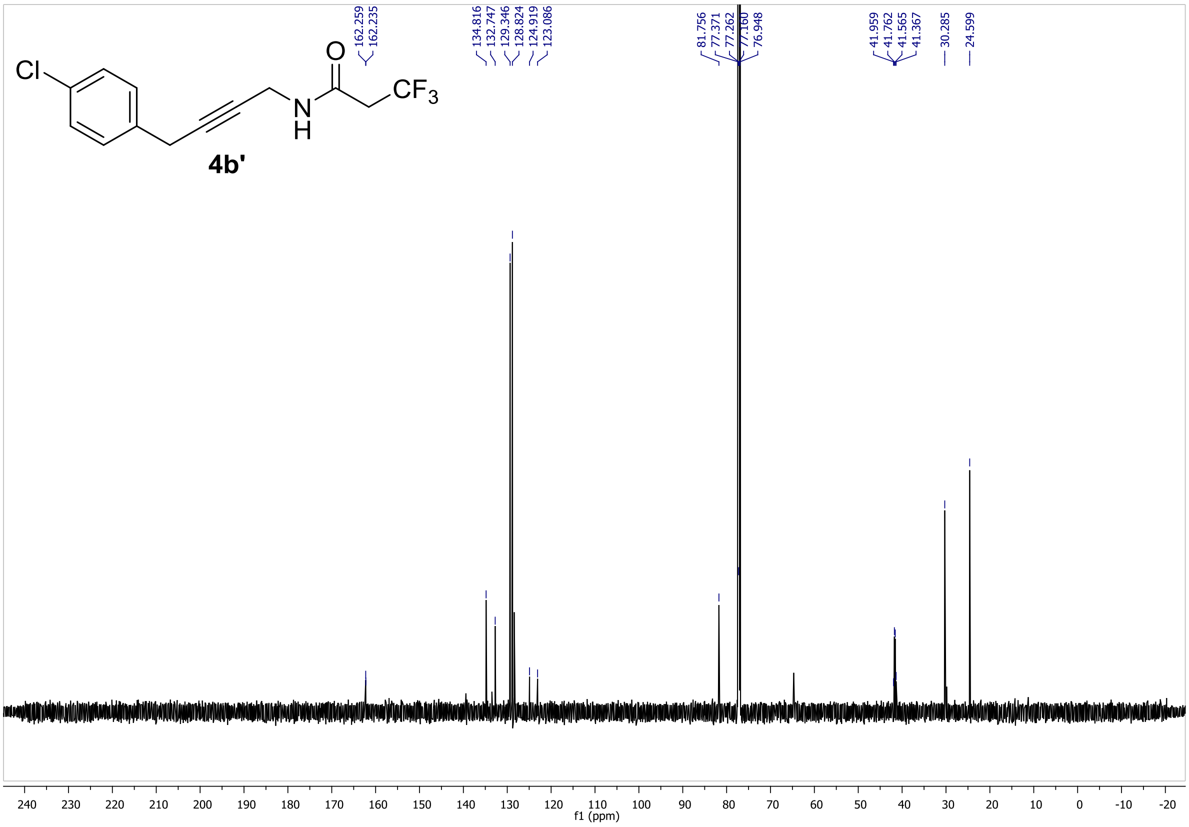

^{19}F NMR, 376 MHz, CDCl$_3$:

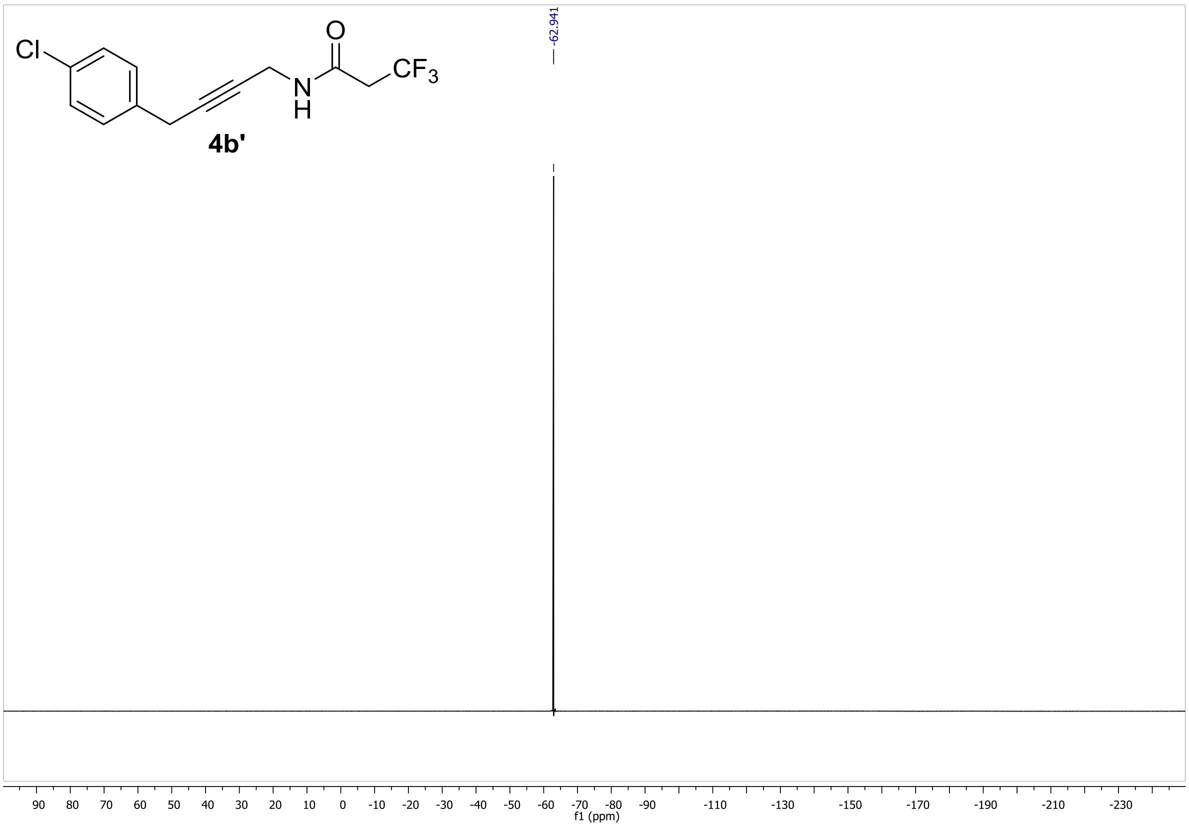

N-(4-(4-chlorophenyl)but-2-yn-1-yl)-6-oxoheptanamide (4c'):

^{1}H NMR, 600 MHz, $CDCl_3$:

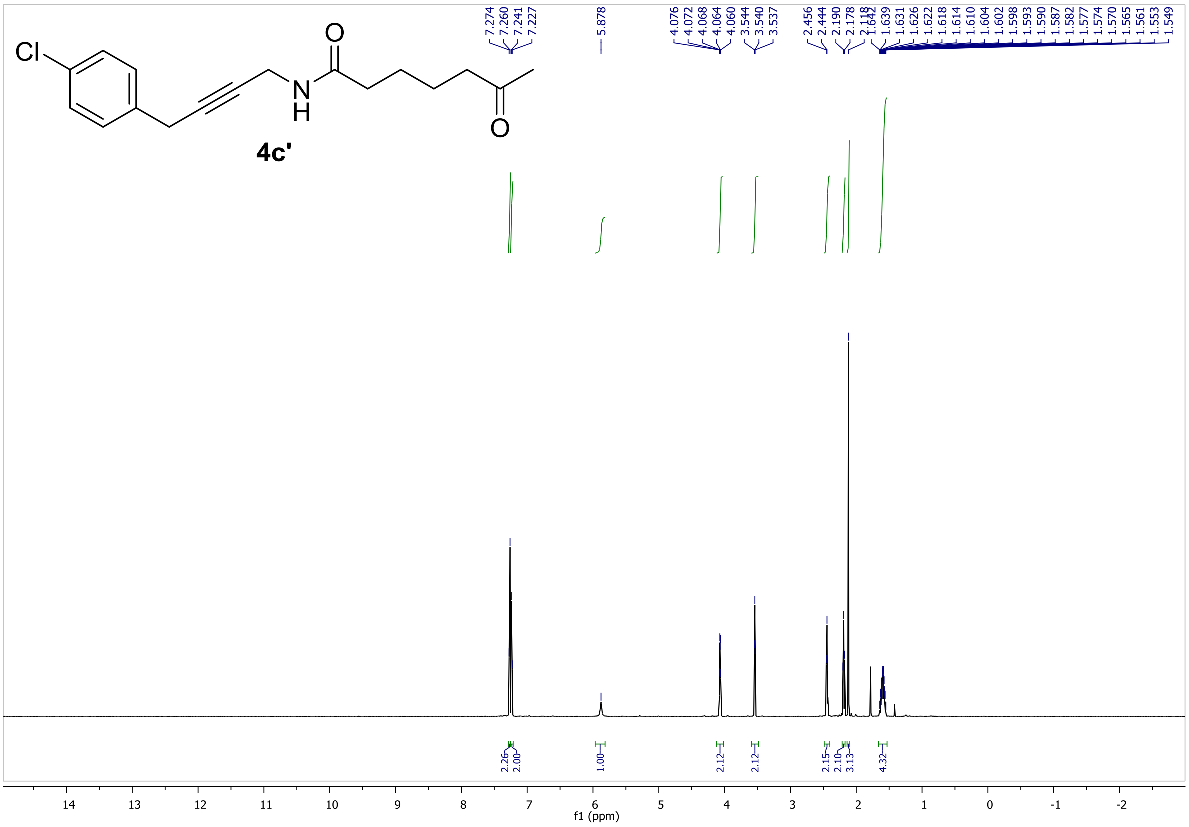

^{13}C NMR, 150 MHz, $CDCl_3$:

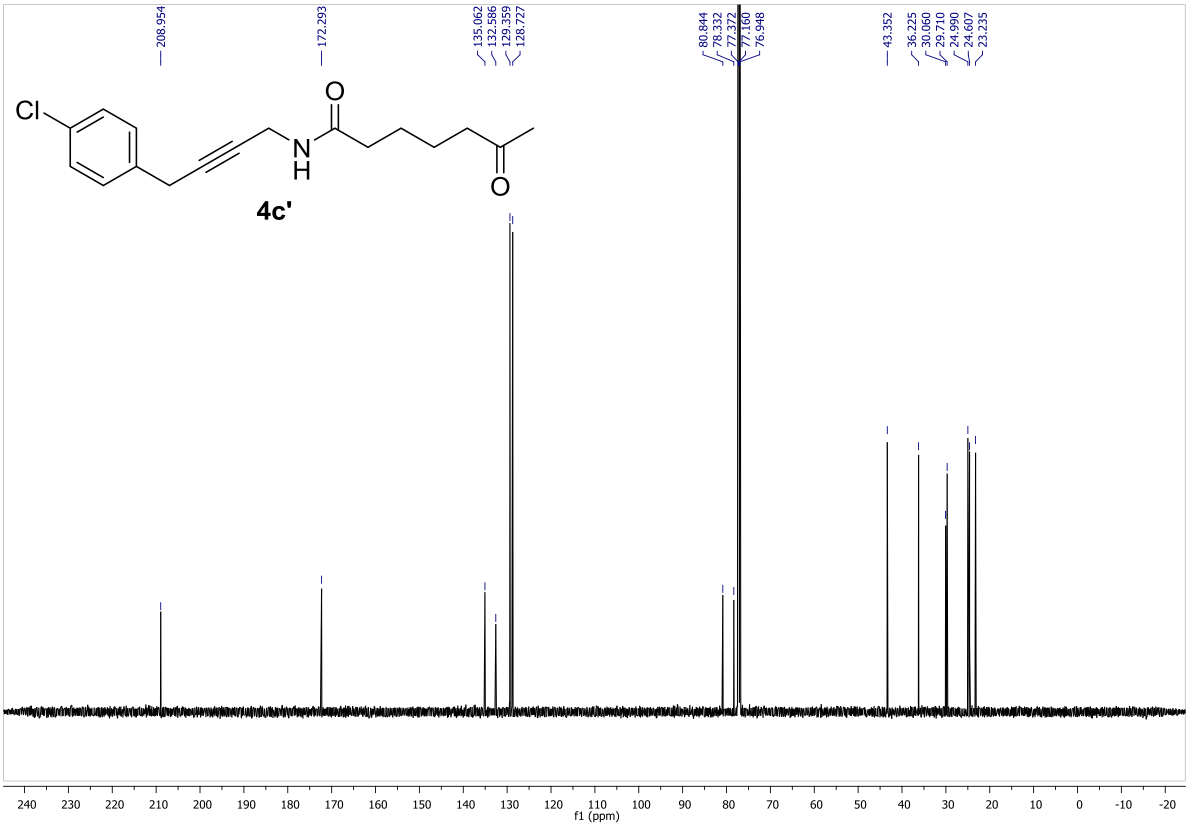

N-(4-(4-chlorophenyl)but-2-yn-1-yl)-4-formylbenzamide (4d’):

^{1}H NMR, 600 MHz, CDCl$_3$:

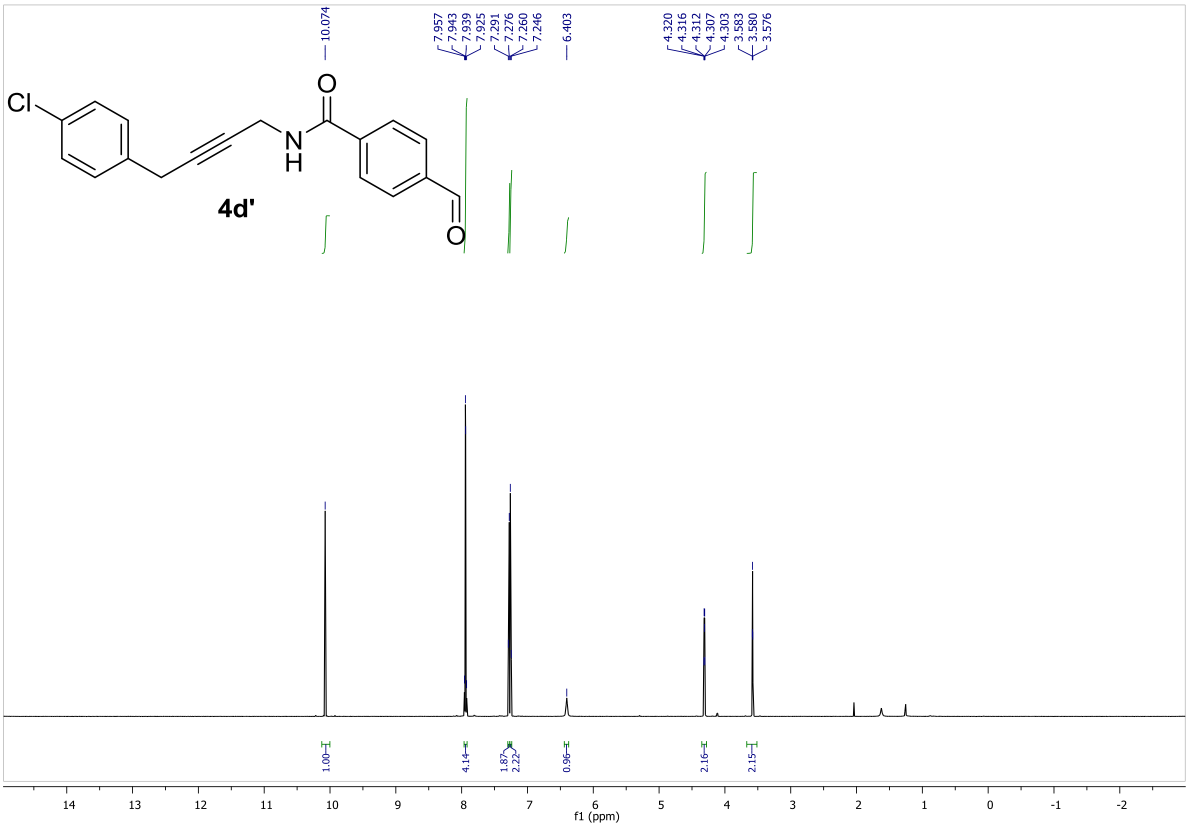

^{13}C NMR, 150 MHz, CDCl$_3$:

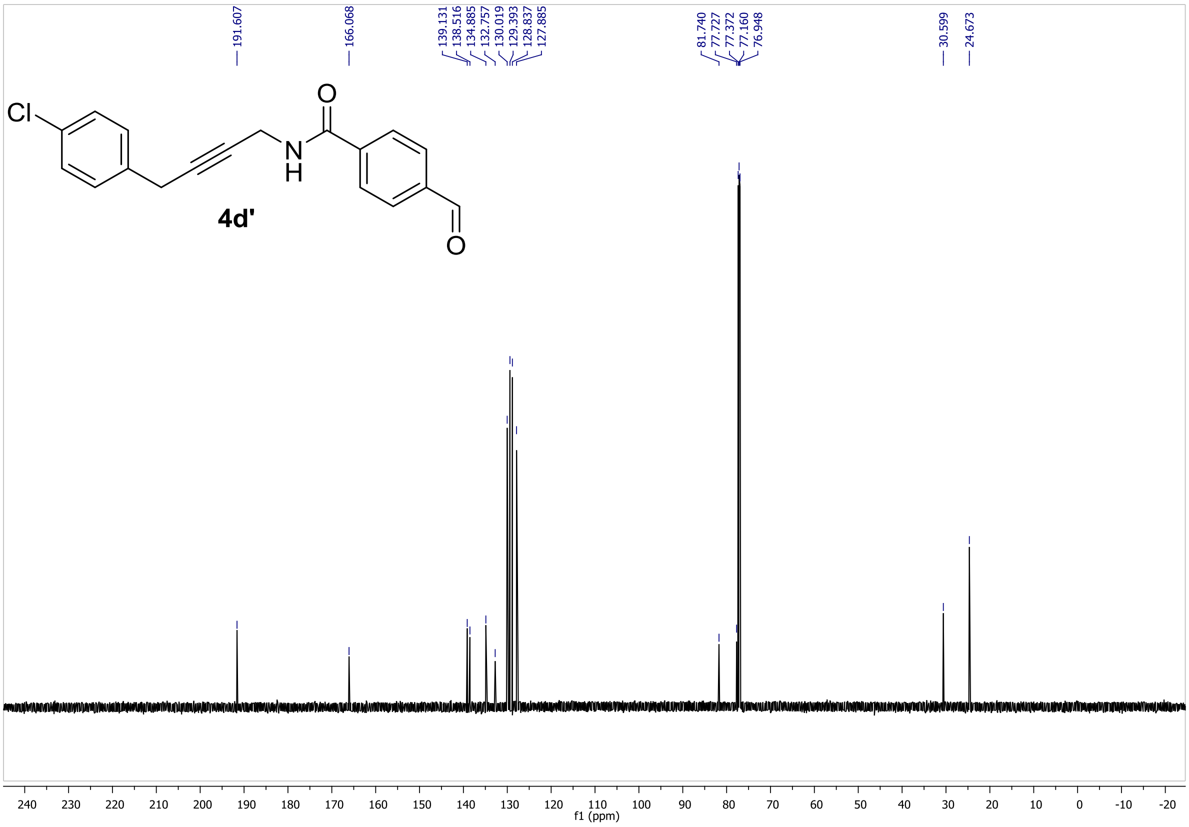

(1*R*,3*s*,5*S*)-*N*-(4-(4-chlorophenyl)but-2-yn-1-yl)-6-oxabicyclo[3.1.0]hexane-3-carboxamide (4e’):

^{1}H NMR, 600 MHz, CDCl$_3$:

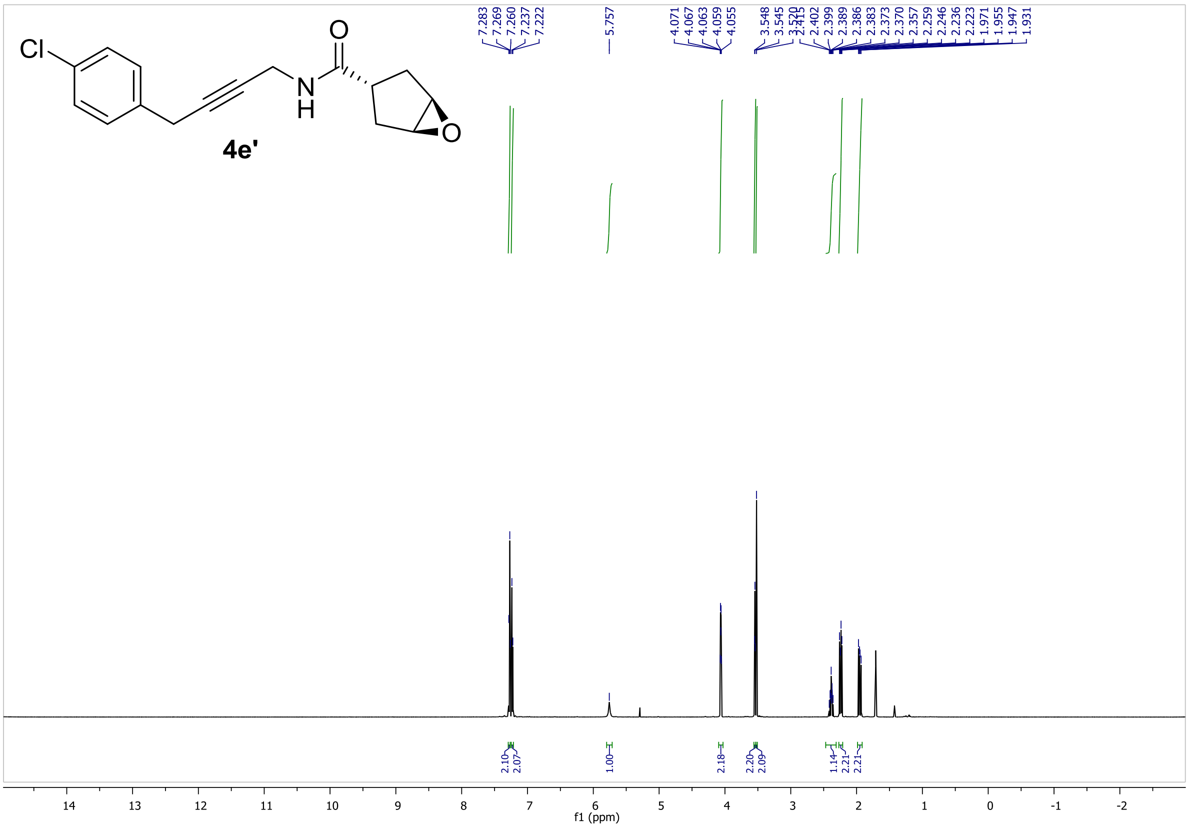

^{13}C NMR, 150 MHz, CDCl$_3$:

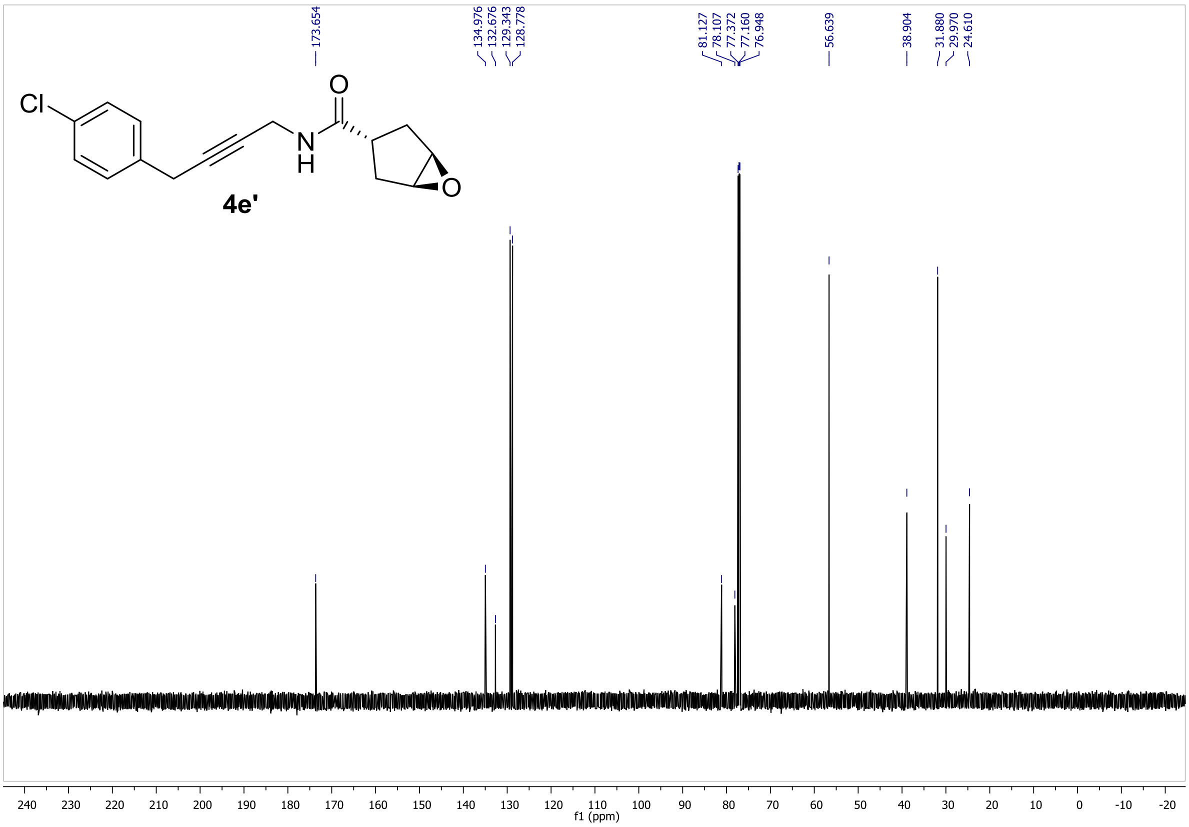

N-(4-(4-chlorophenyl)but-2-yn-1-yl)thiophene-2-carboxamide (4f'):

^{1}H NMR, 600 MHz, $CDCl_3$:

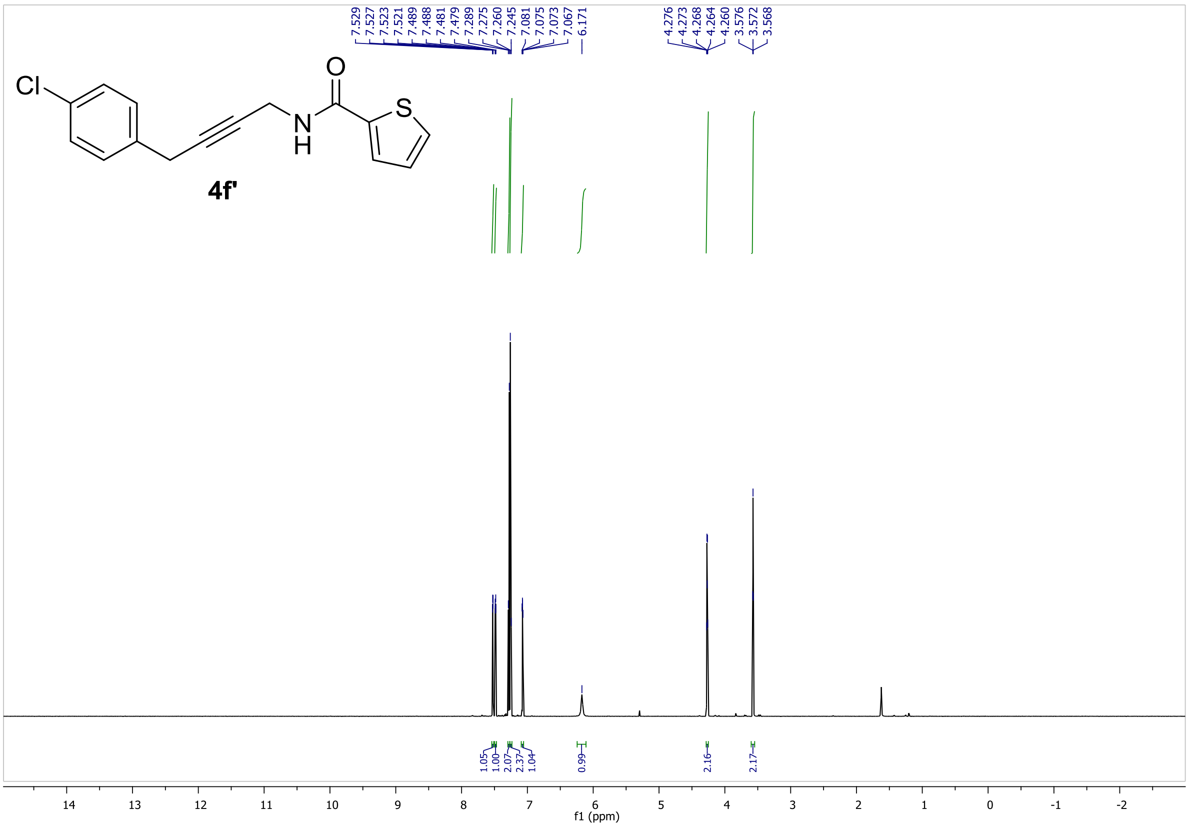

^{13}C NMR, 150 MHz, $CDCl_3$:

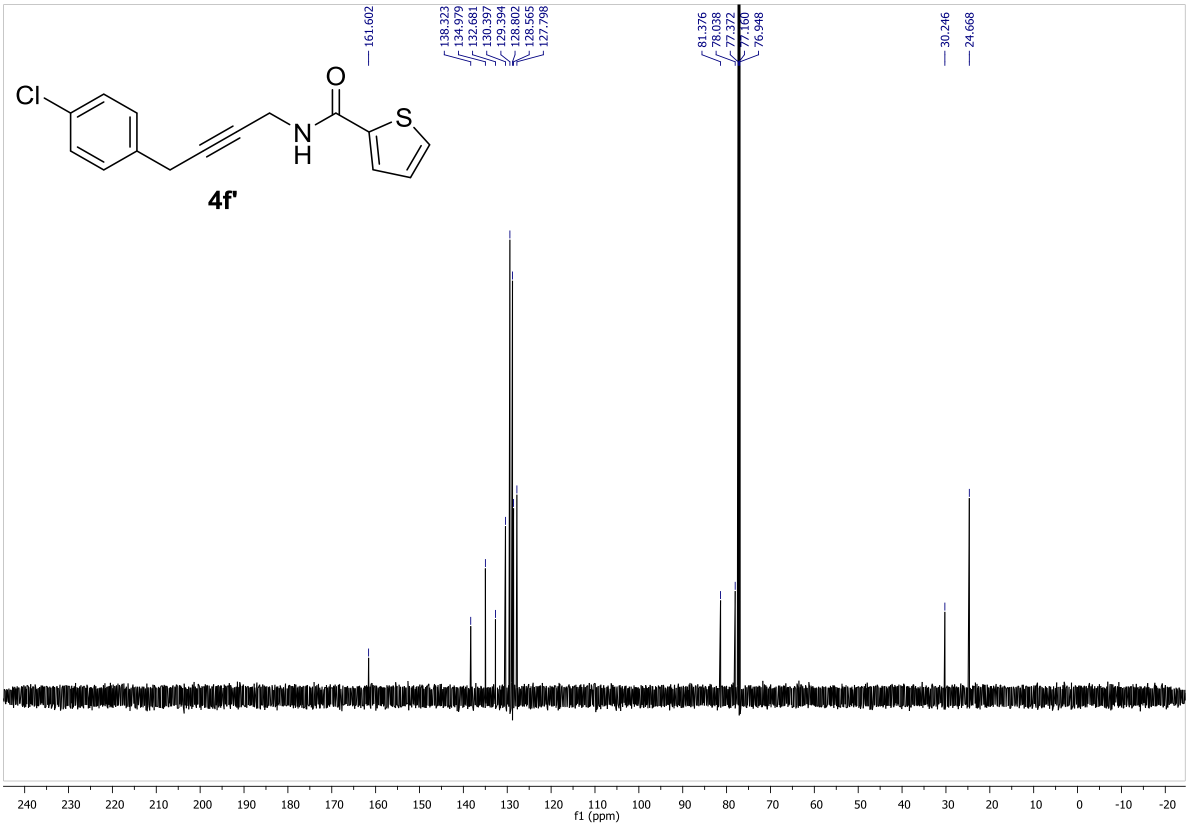

***tert*-butyl (*R*)-(4-(4-chlorophenyl)buta-2,3-dien-1-yl)carbamate (4g'):**

^{1}H NMR, 600 MHz, $CDCl_3$:

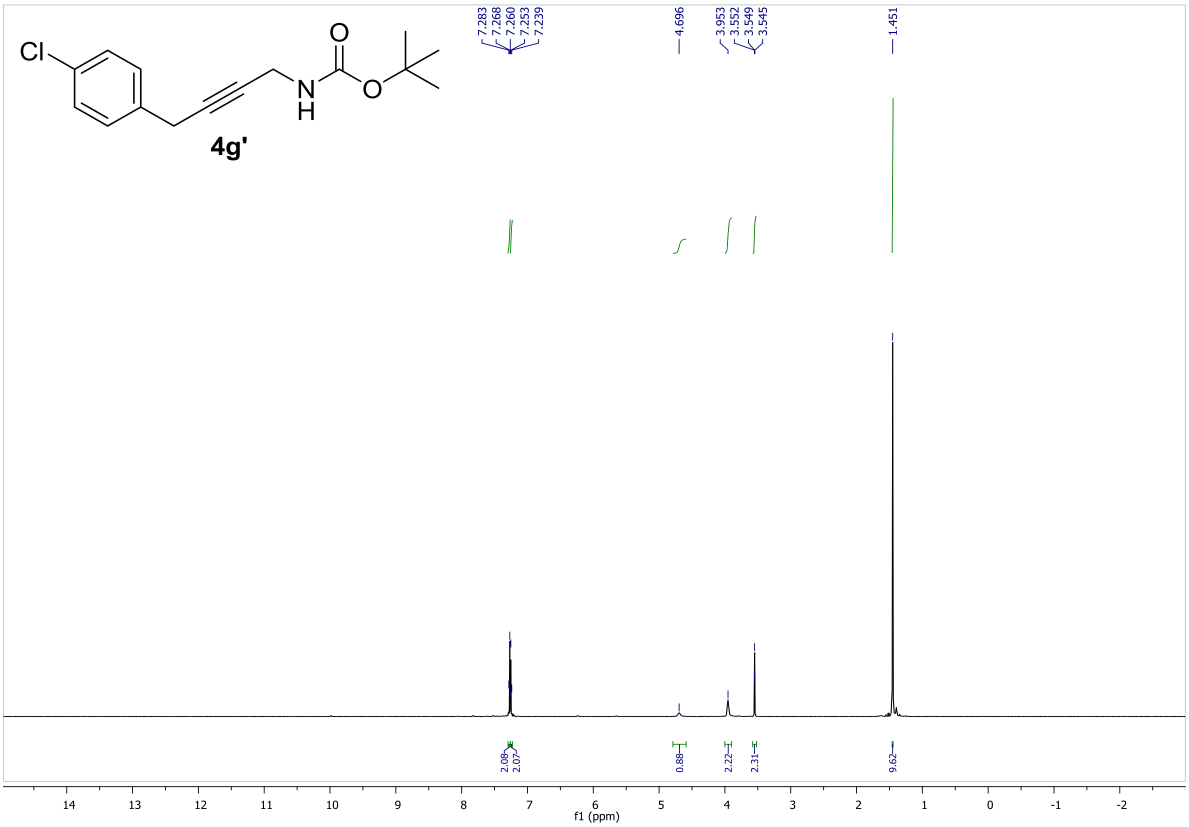

^{13}C NMR, 150 MHz, $CDCl_3$:

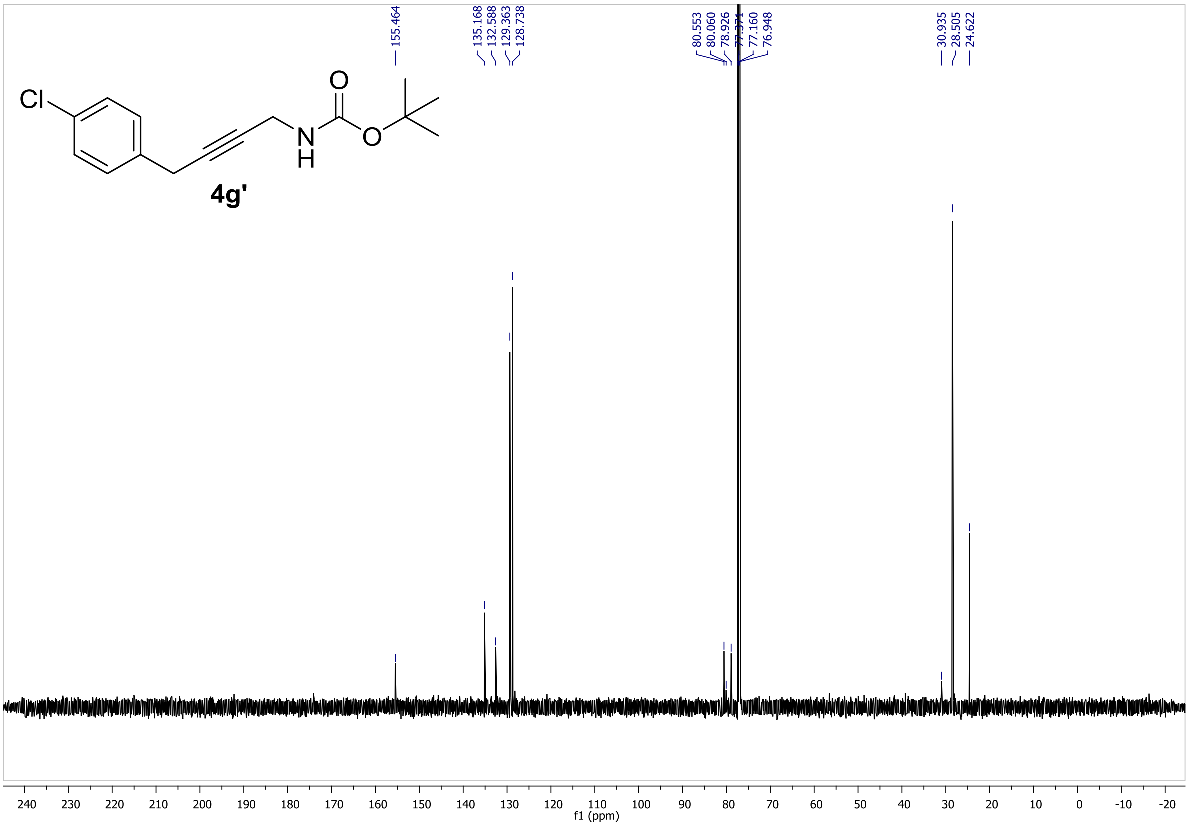

N-(4-(4-bromophenyl)but-2-yn-1-yl)-1-(4-chlorophenyl)cyclopentane-1-carboxamide (4h’):

^{1}H NMR, 600 MHz, $CDCl_3$:

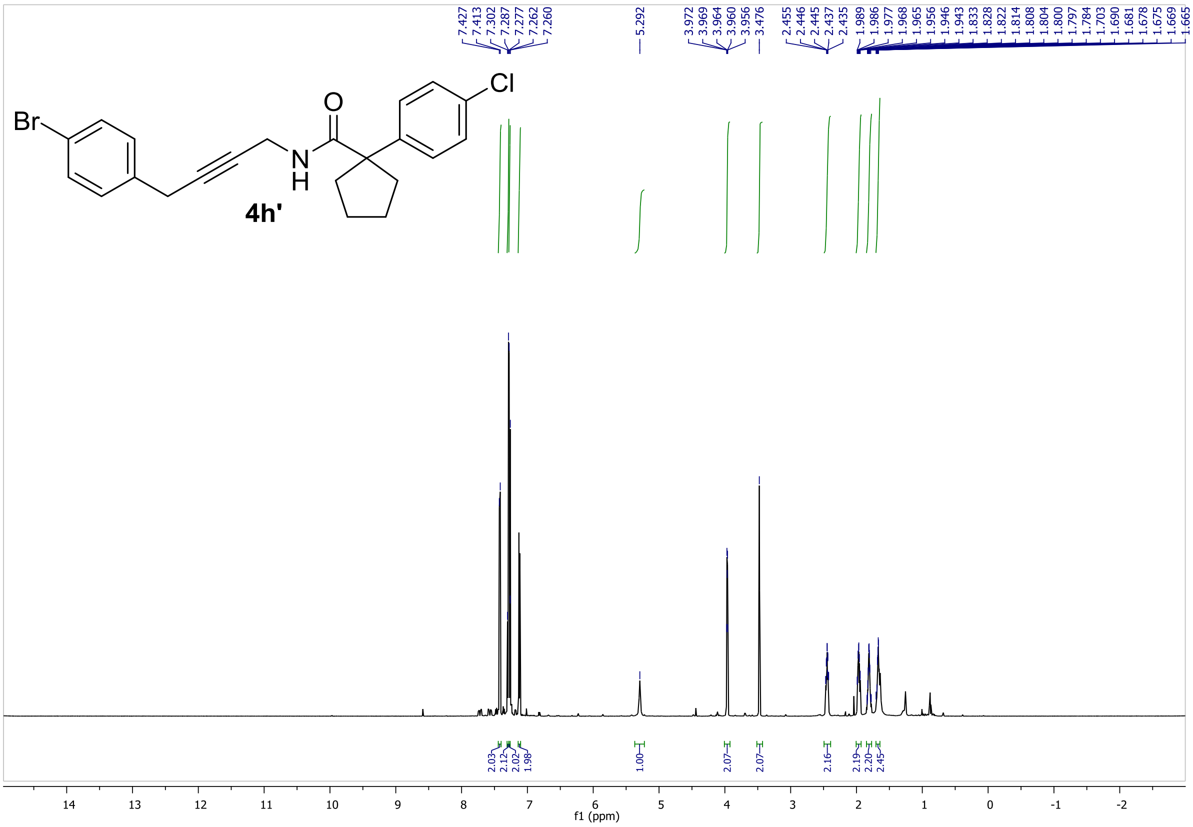

^{13}C NMR, 150 MHz, $CDCl_3$:

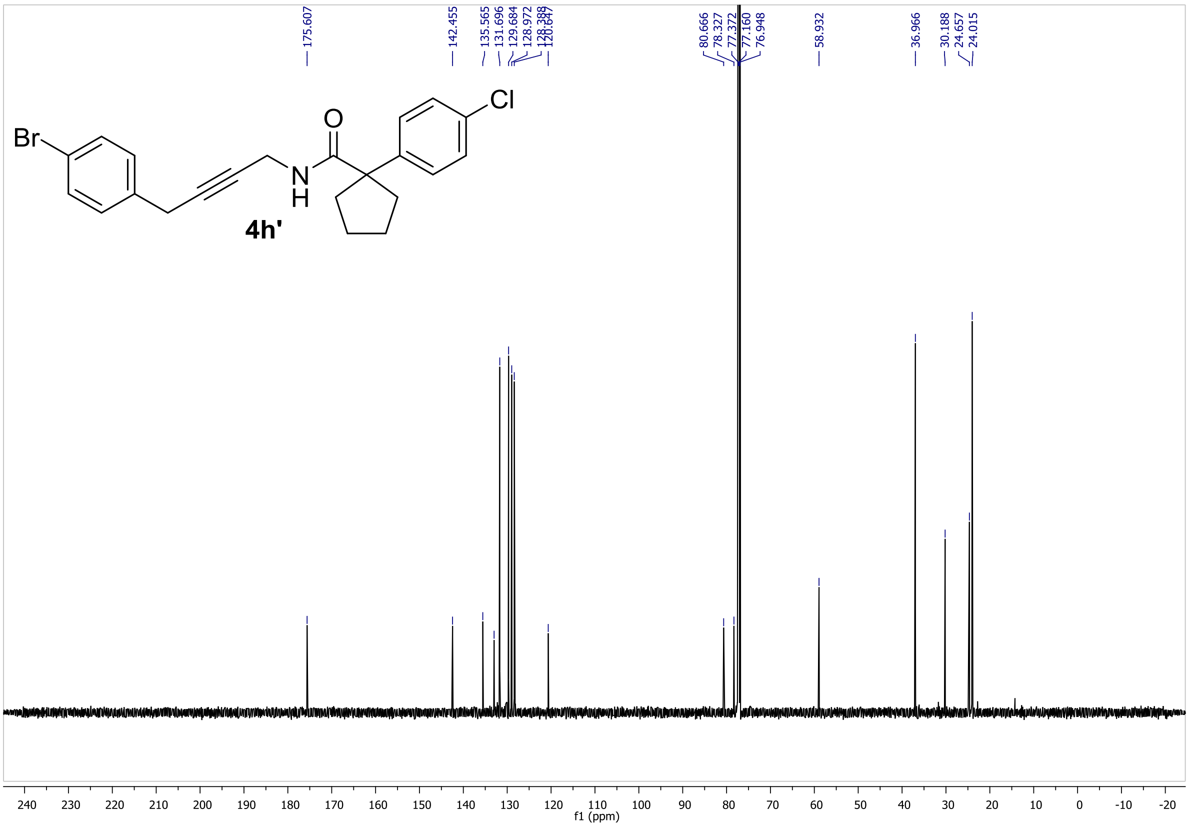

N-(4-(4-bromophenyl)but-2-yn-1-yl)-4-hydroxybutanamide (4i'):

^{1}H NMR, 600 MHz, $CDCl_3$:

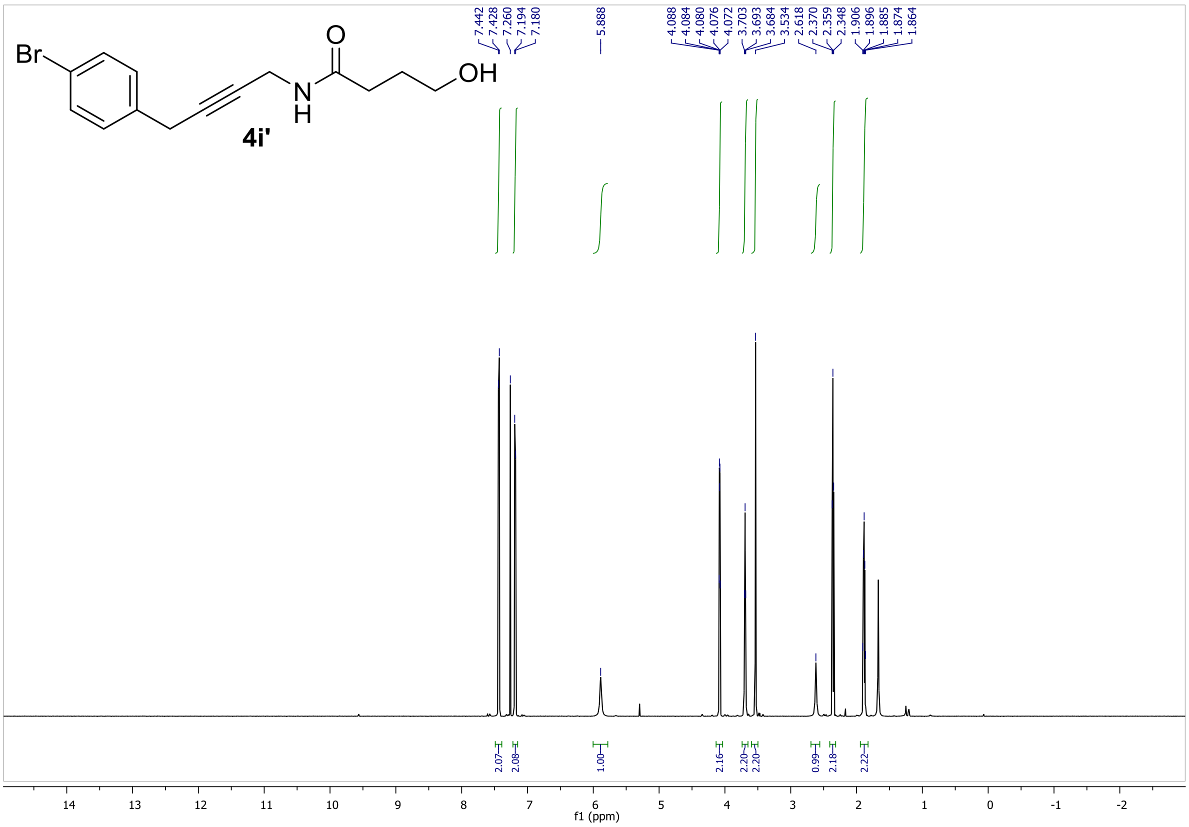

^{13}C NMR, 150 MHz, $CDCl_3$:

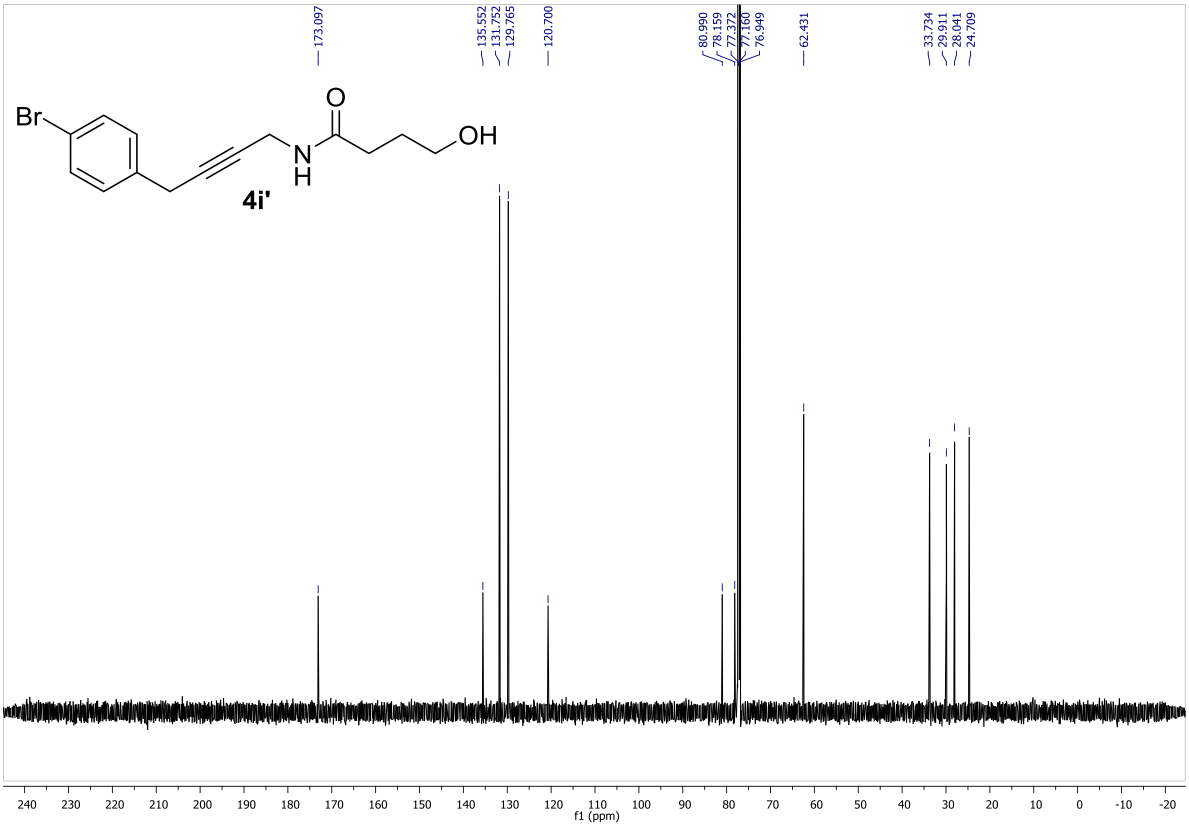

5-bromo-*N*-(4-(4-bromophenyl)but-2-yn-1-yl)furan-2-carboxamide (4j’):

^{1}H NMR, 600 MHz, $CDCl_3$:

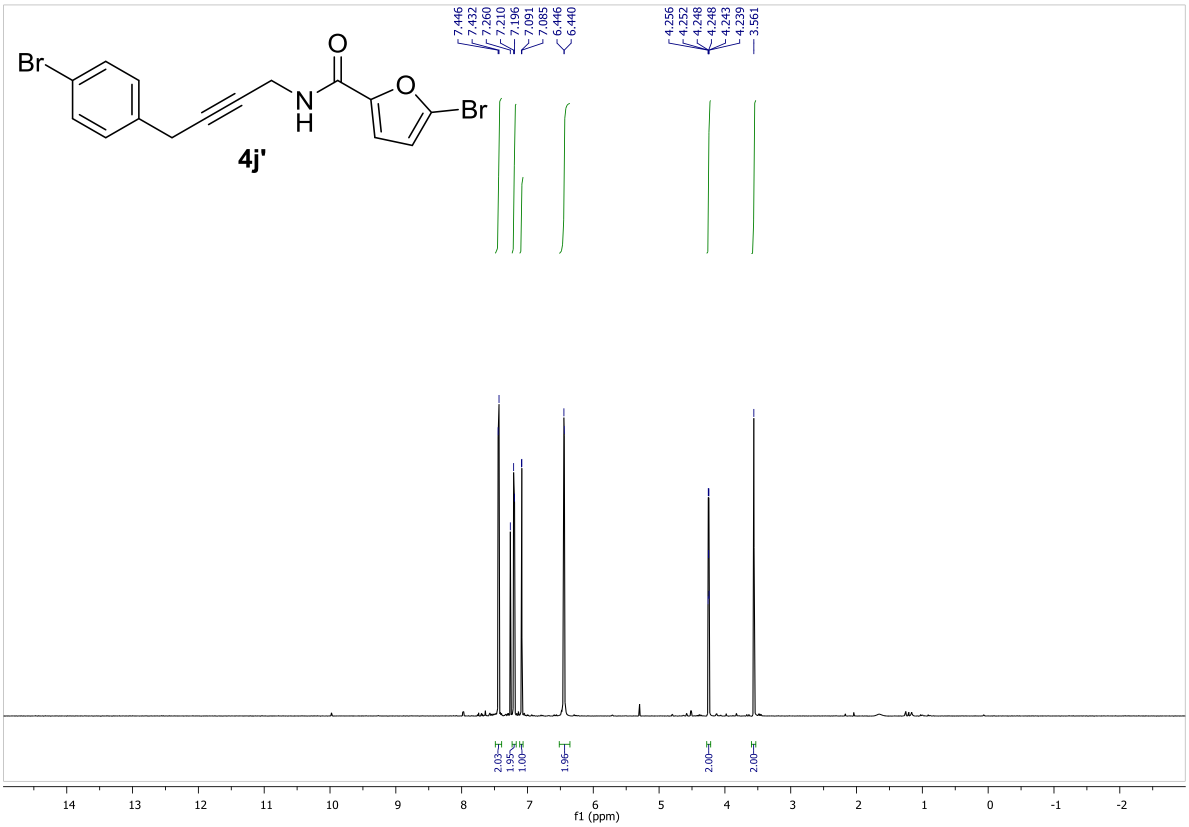

^{13}C NMR, 150 MHz, $CDCl_3$:

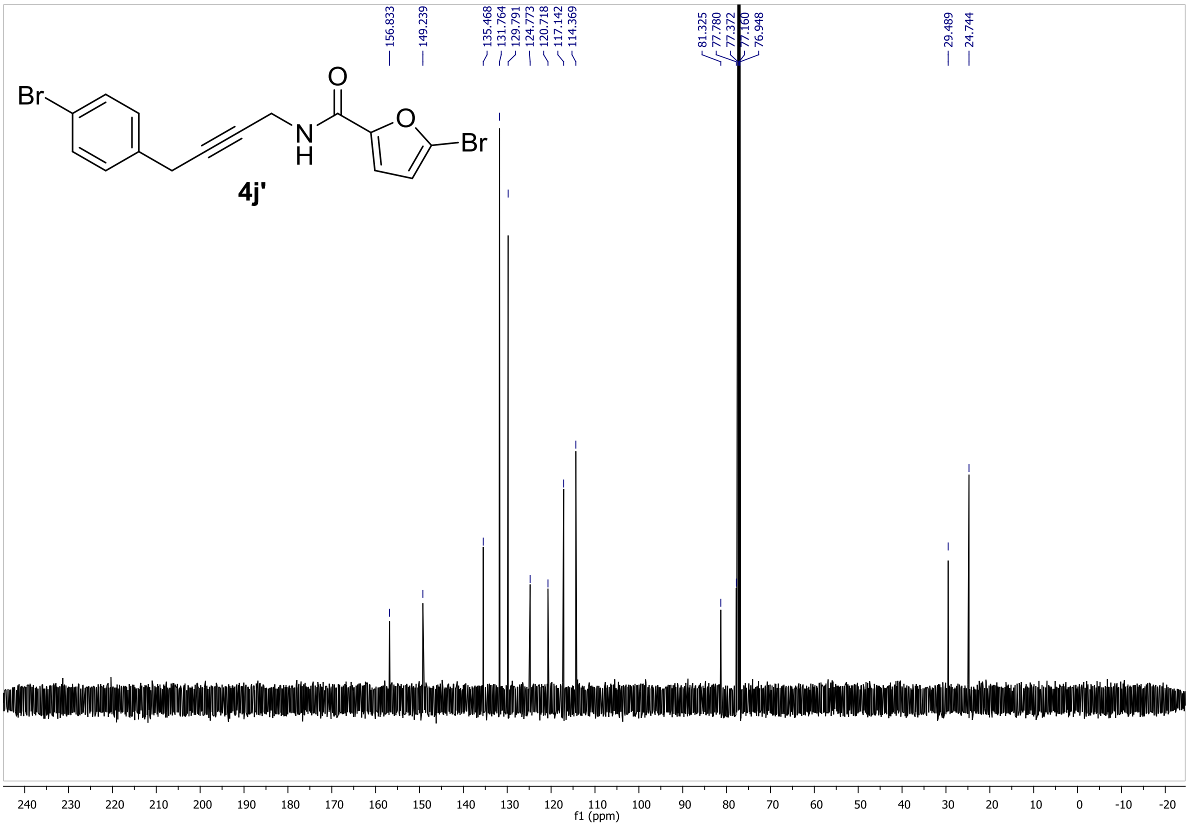

***N*-(4-(4-bromophenyl)but-2-yn-1-yl)cyclopropanecarboxamide (4k’):**

^{1}H NMR, 600 MHz, CDCl$_3$:

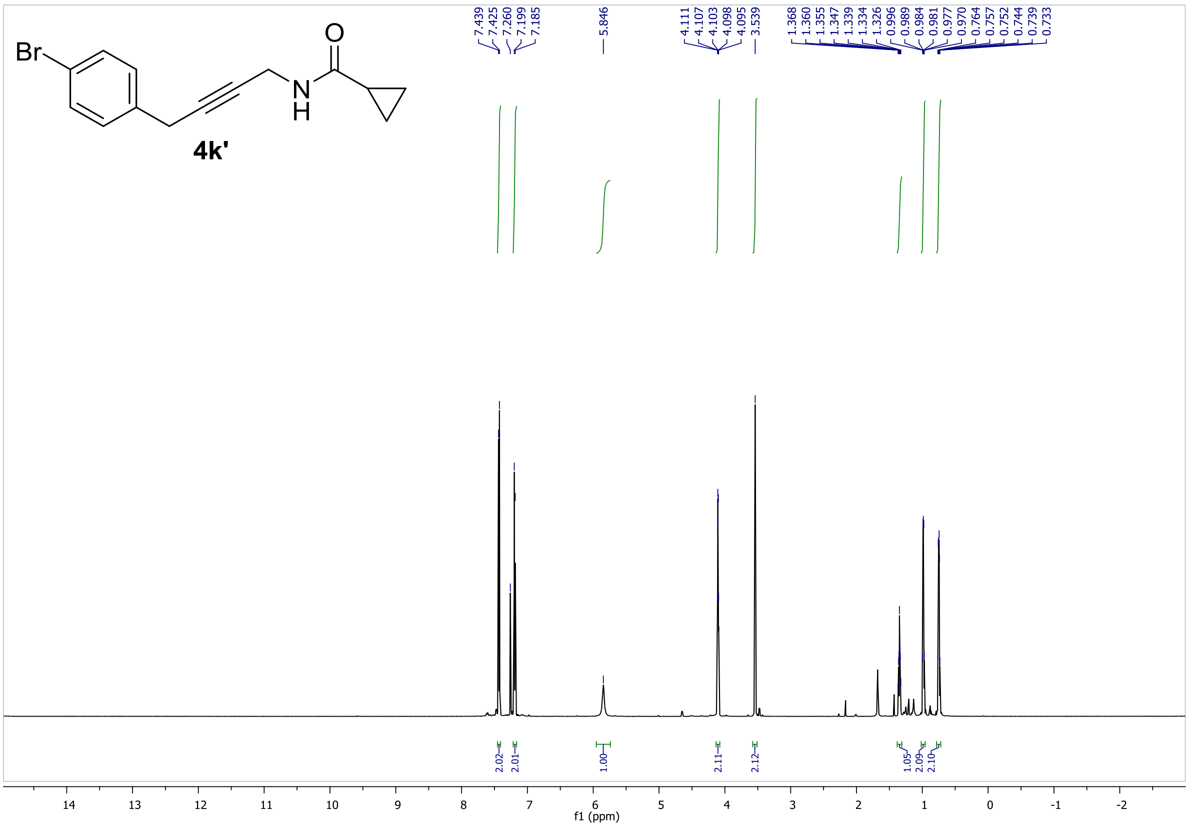

^{13}C NMR, 150 MHz, CDCl$_3$:

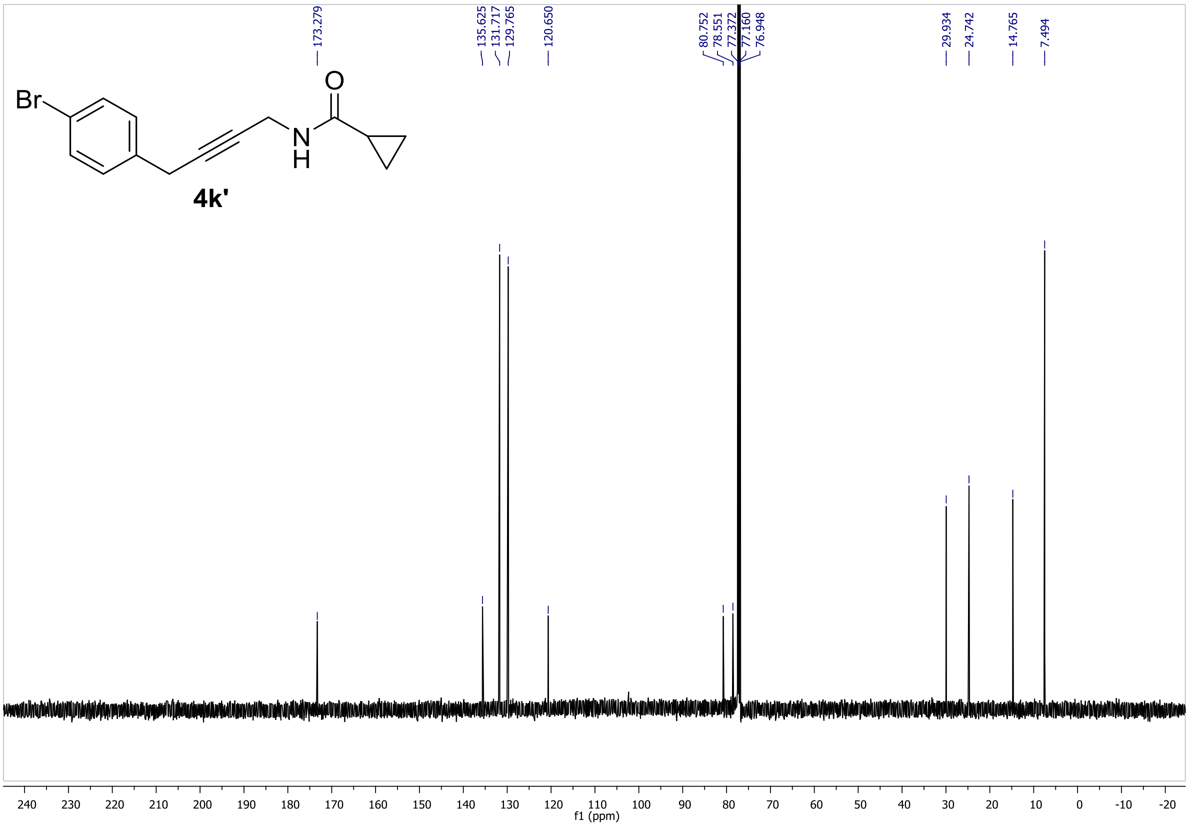

Methyl 4-((4-(4-bromophenyl)but-2-yn-1-yl)amino)-4-oxobutanoate (4l’):

^{1}H NMR, 600 MHz, $CDCl_3$:

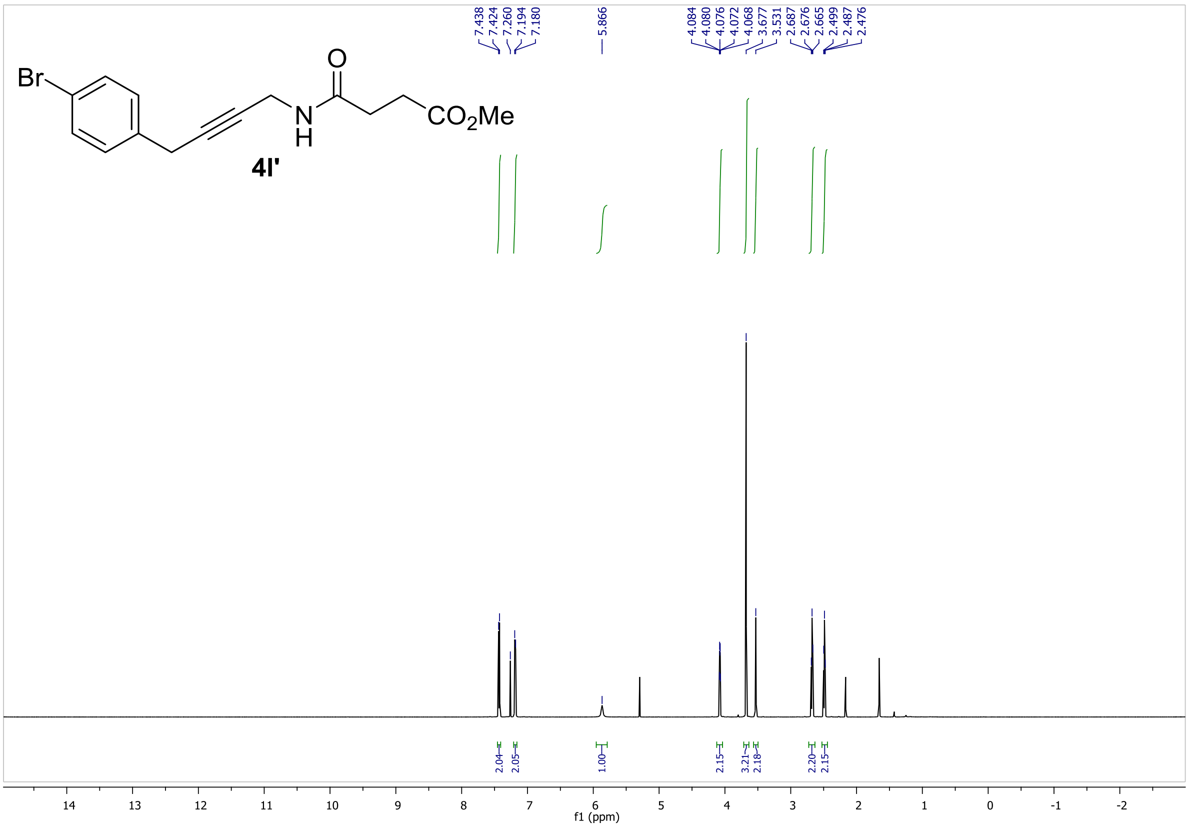

^{13}C NMR, 150 MHz, $CDCl_3$:

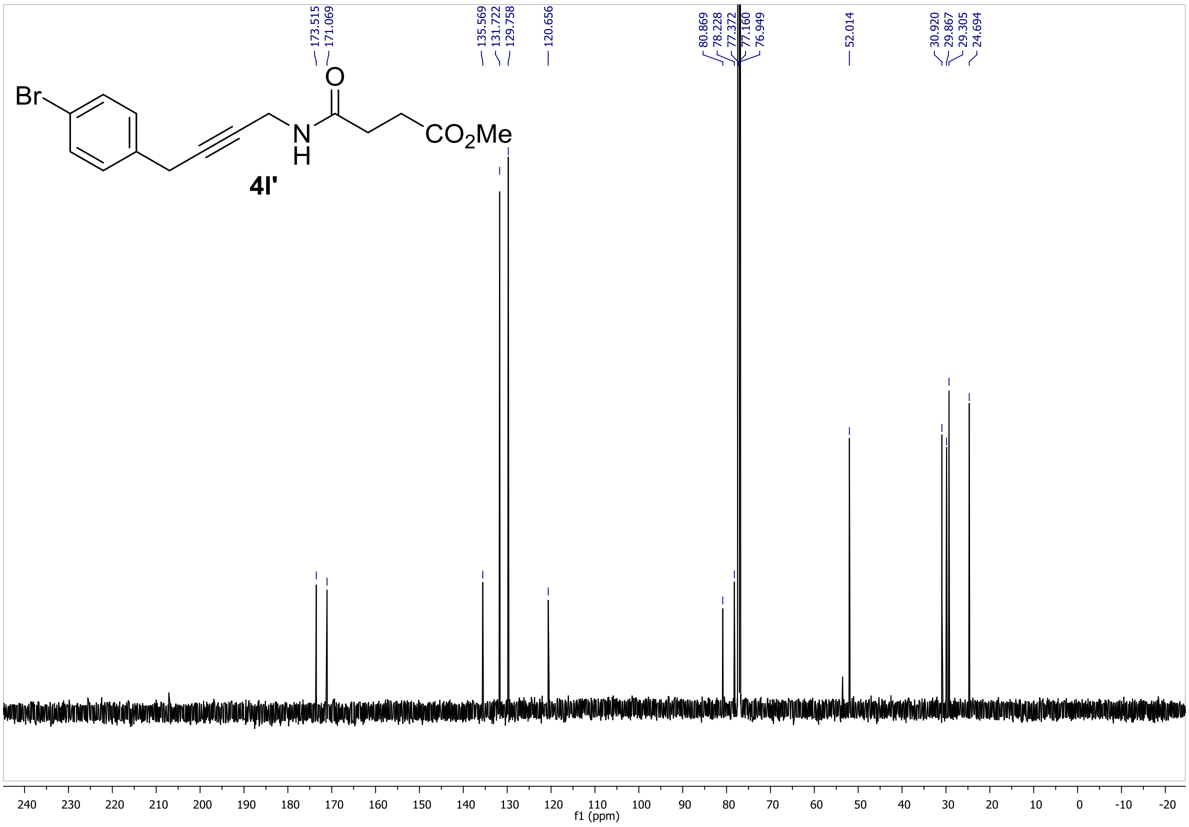

N-(4-(4-chlorophenyl)but-2-yn-1-yl)acetamide (4m’):

^{1}H NMR, 600 MHz, $CDCl_3$:

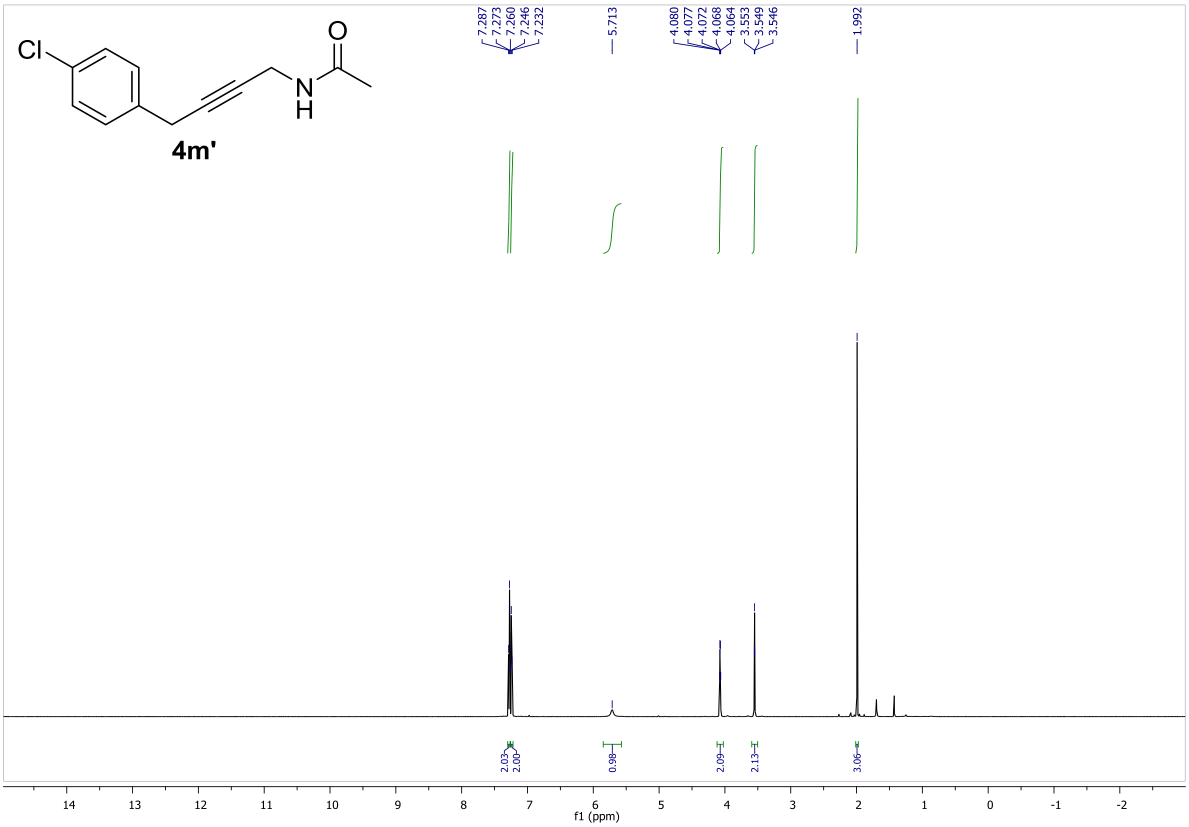

^{13}C NMR, 150 MHz, $CDCl_3$:

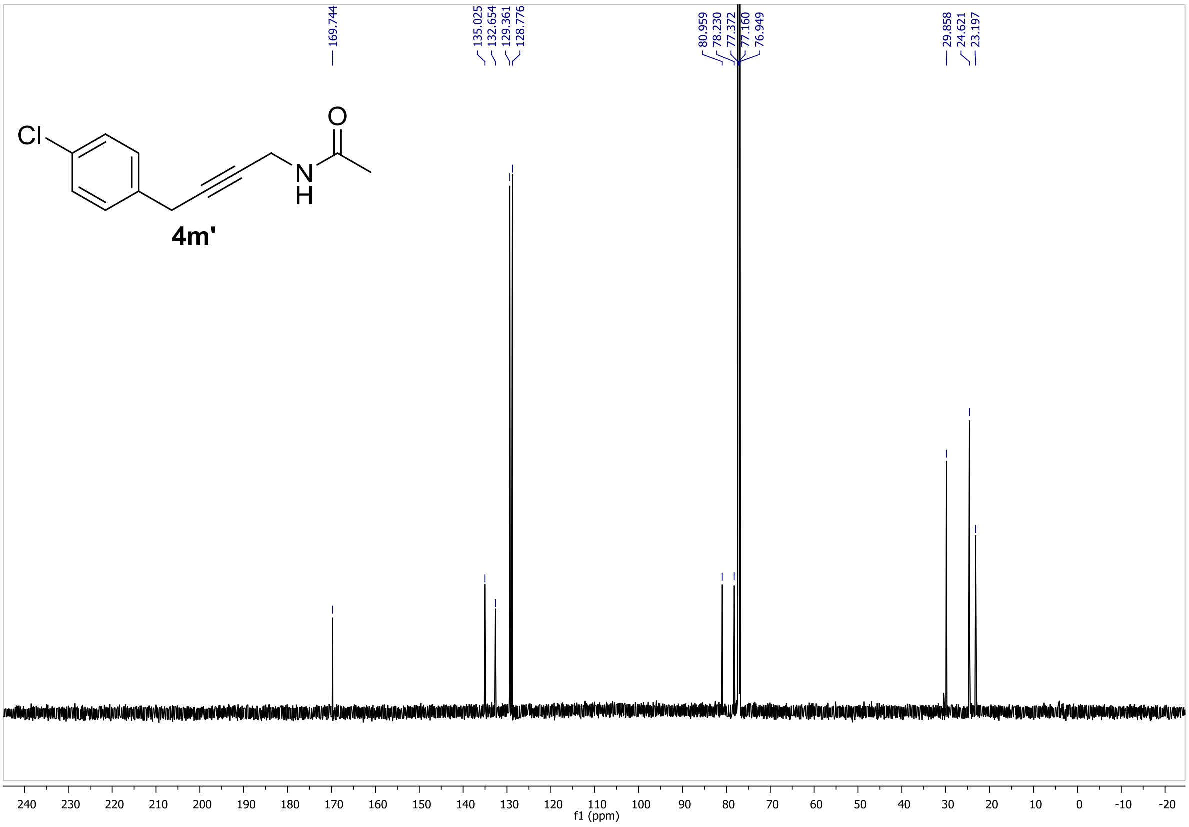

N-(4-(4-bromophenyl)but-2-yn-1-yl)acetamide (4n’):

^{1}H NMR, 600 MHz, CDCl$_3$:

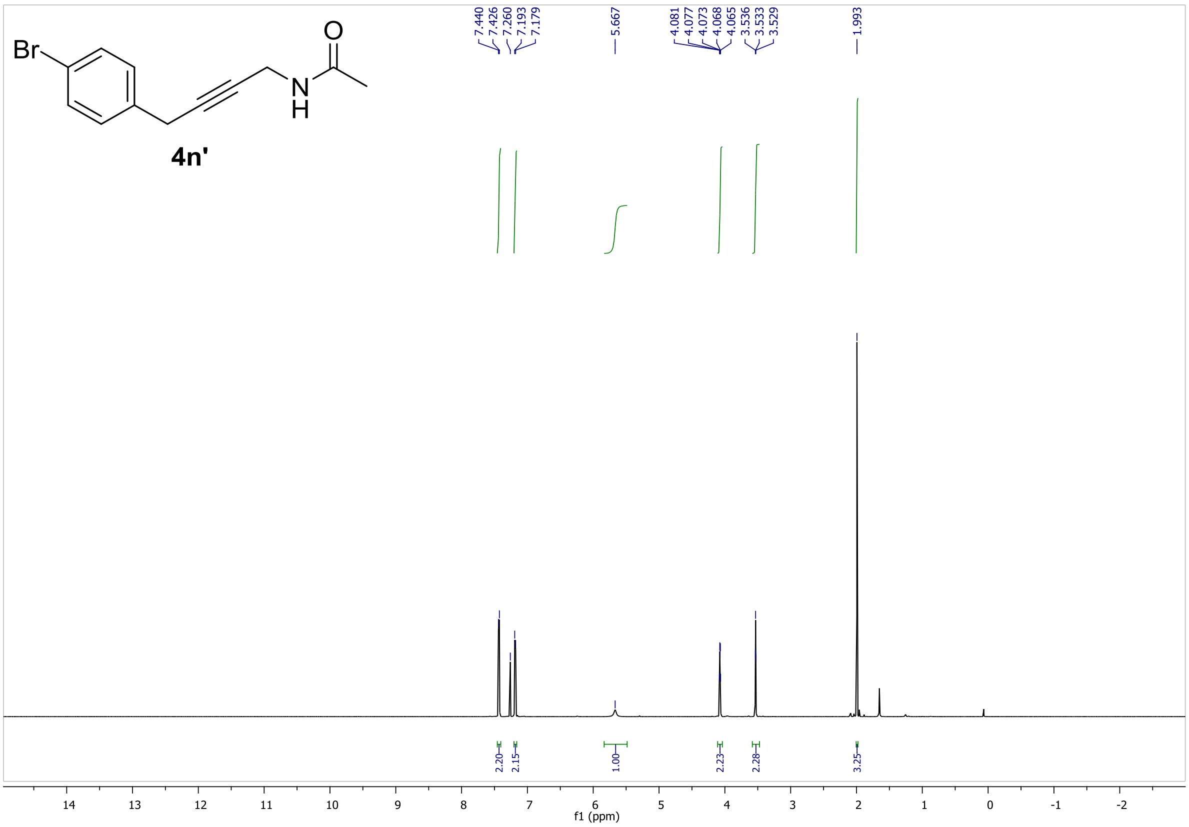

^{13}C NMR, 150 MHz, CDCl$_3$:

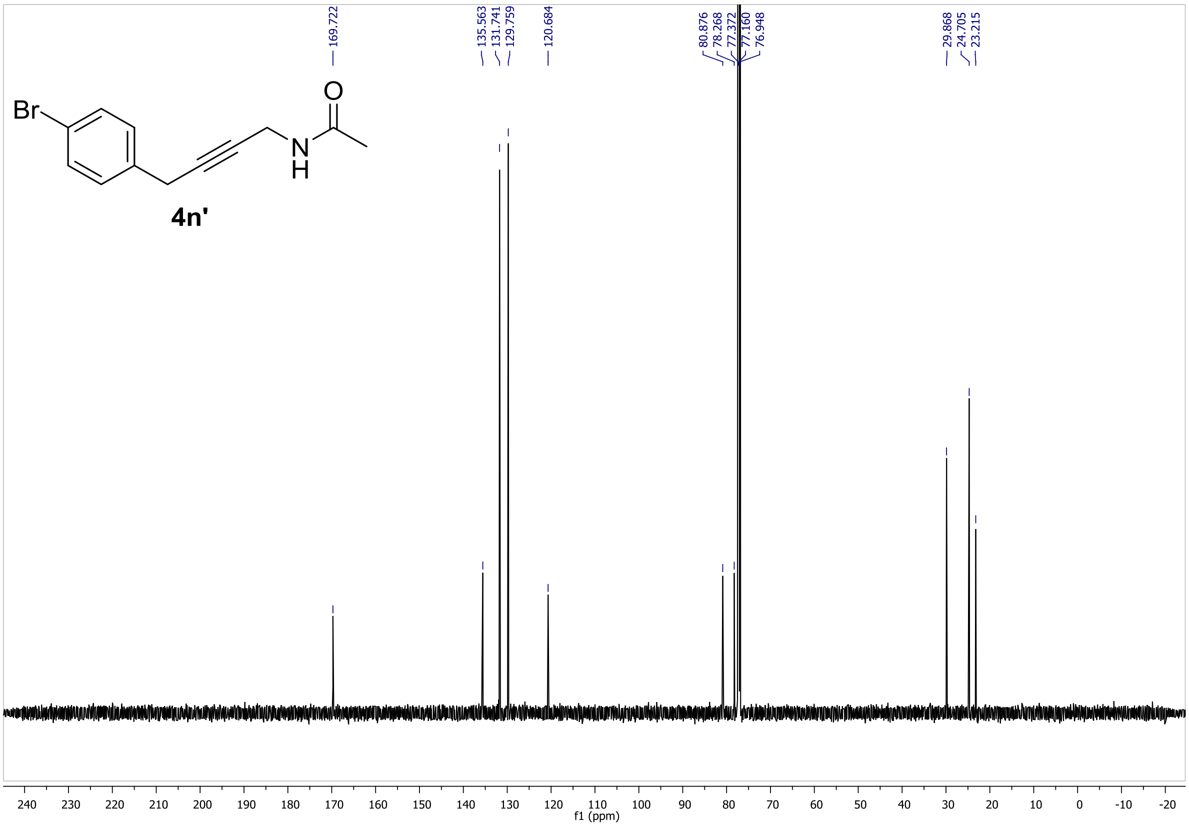

N-(4-(4-fluorophenyl)but-2-yn-1-yl)acetamide (4o'):

^{1}H NMR, 600 MHz, $CDCl_3$:

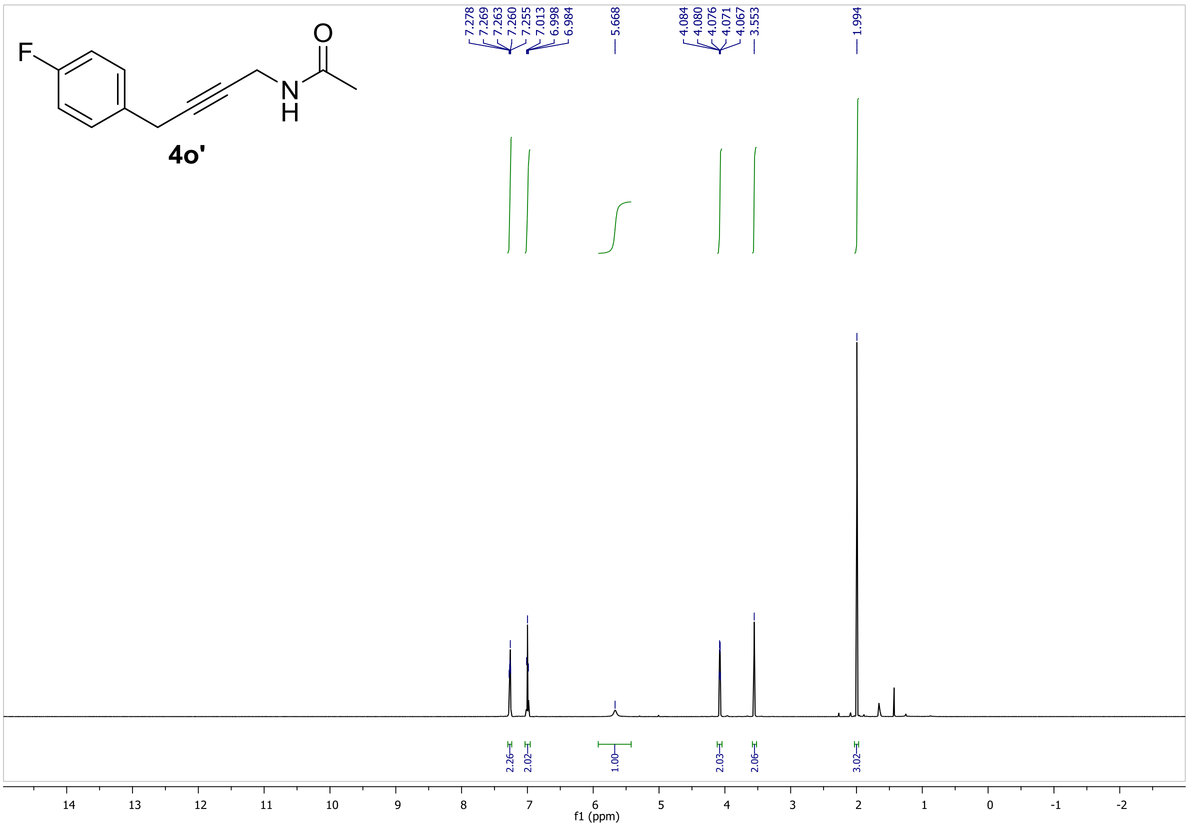

^{13}C NMR, 150 MHz, $CDCl_3$:

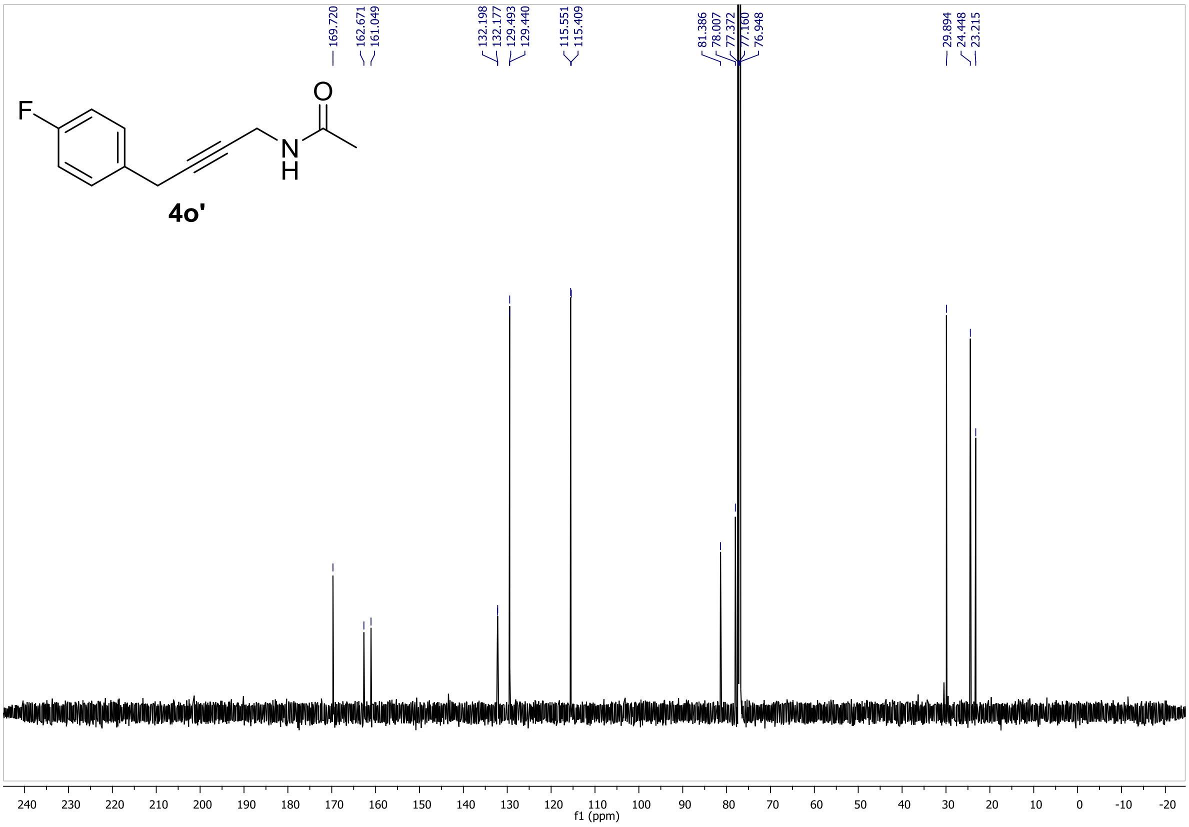

^{19}F NMR, 376 MHz, CDCl$_3$:

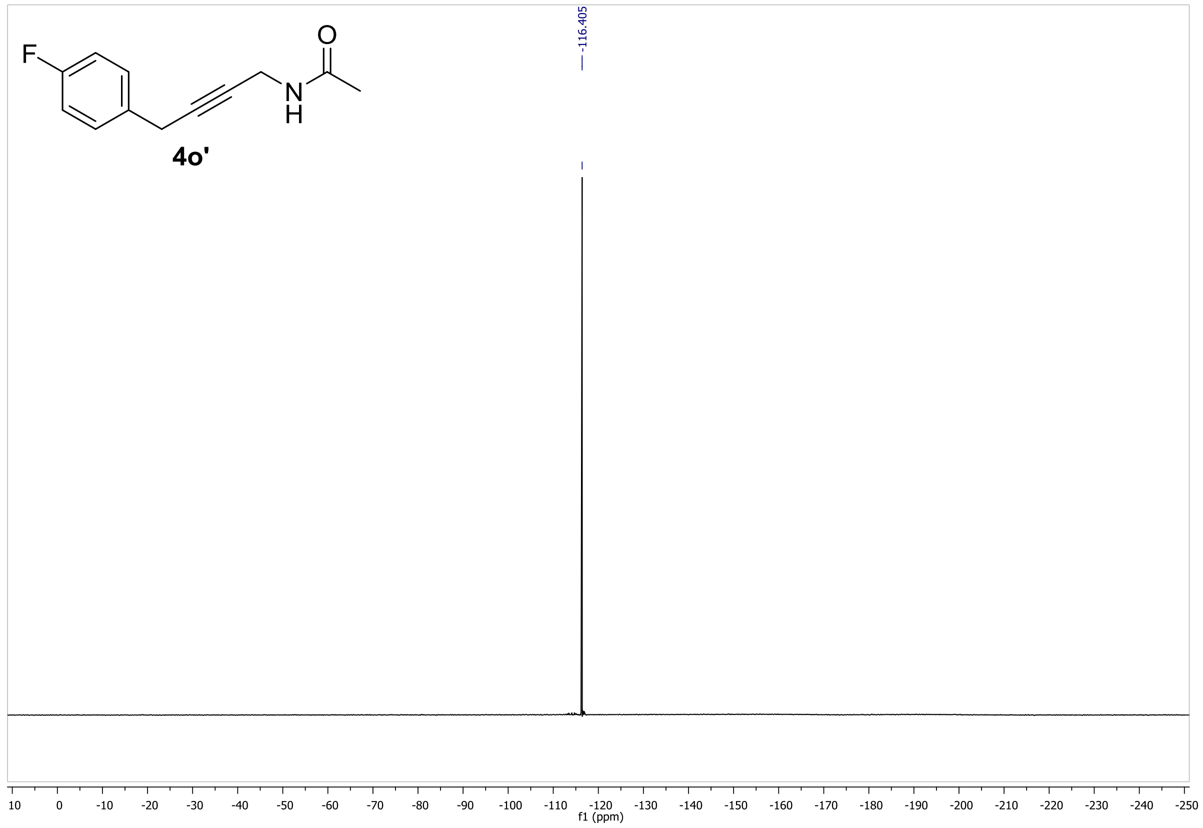

***N*-(4-(3-methoxyphenyl)but-2-yn-1-yl)acetamide (4p'):**

^{1}H NMR, 600 MHz, $CDCl_3$:

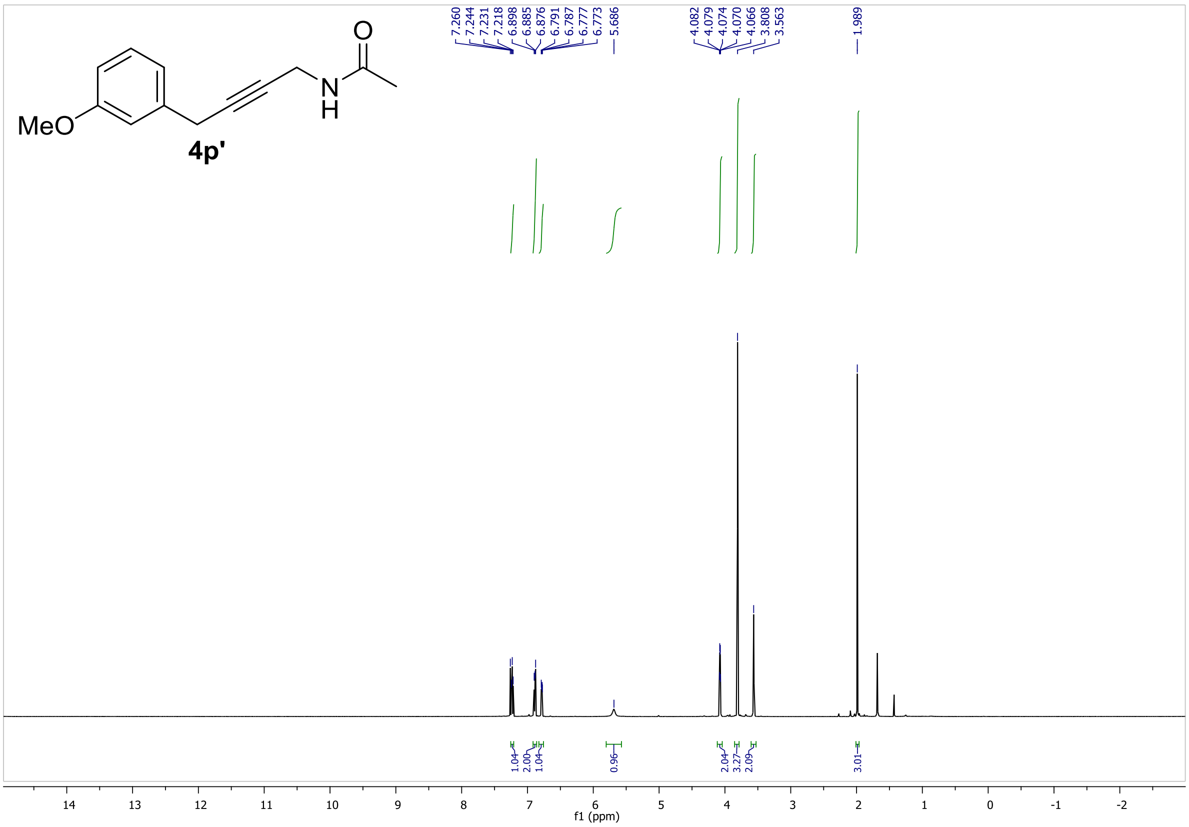

^{13}C NMR, 150 MHz, $CDCl_3$:

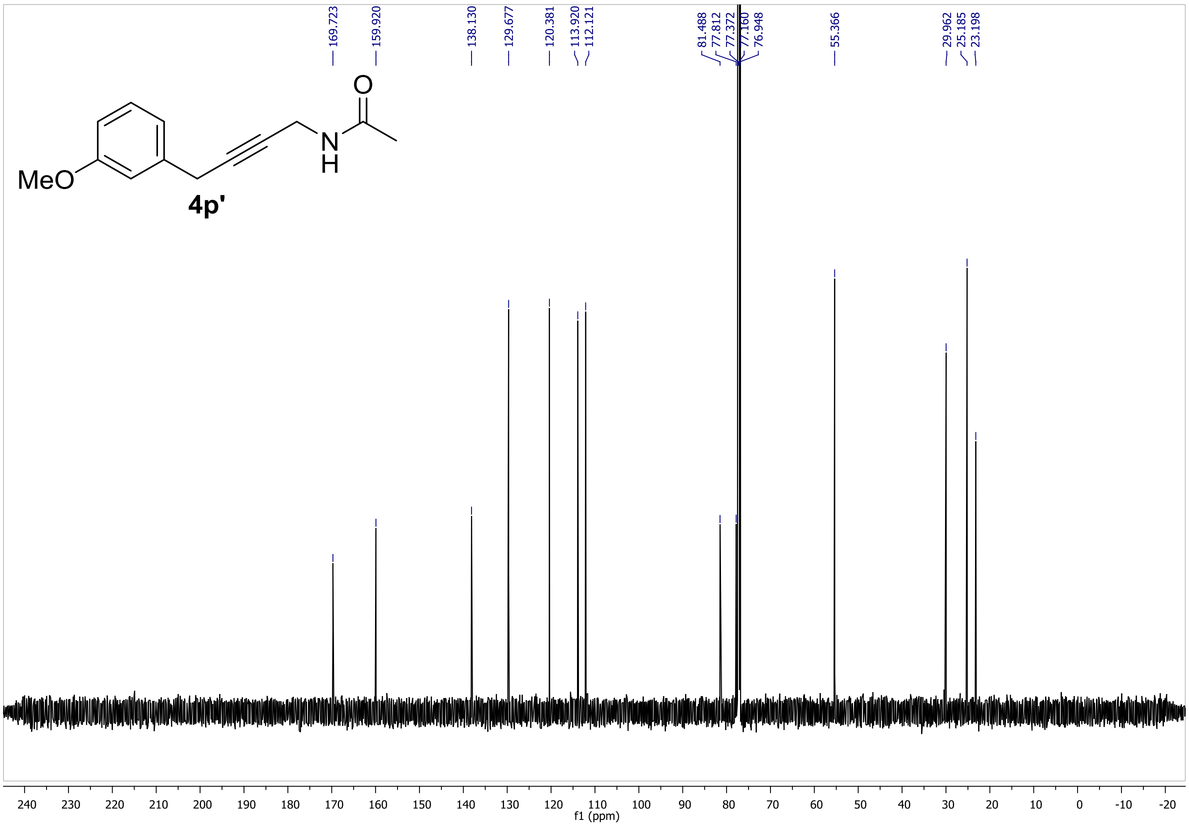

N-(4-(3-cyanophenyl)but-2-yn-1-yl)acetamide (4q'):

^{1}H NMR, 600 MHz, CDCl$_3$:

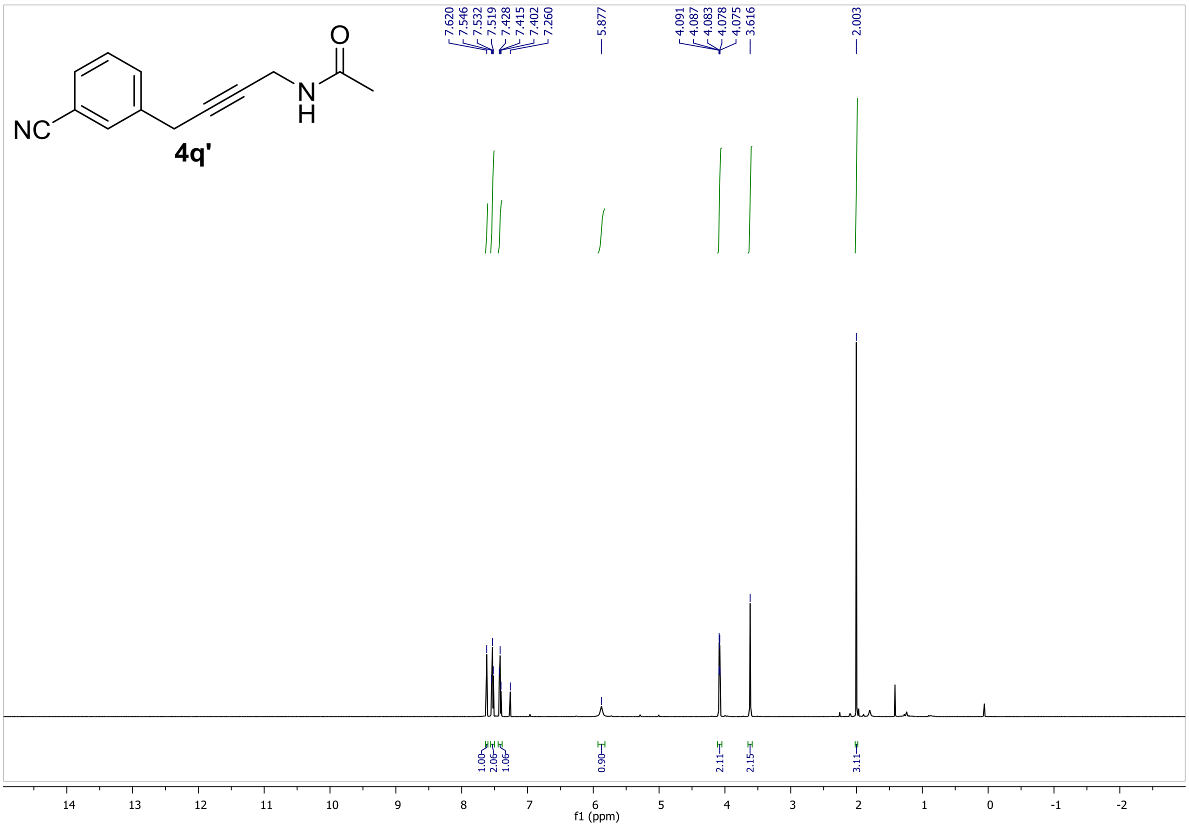

^{13}C NMR, 150 MHz, CDCl$_3$:

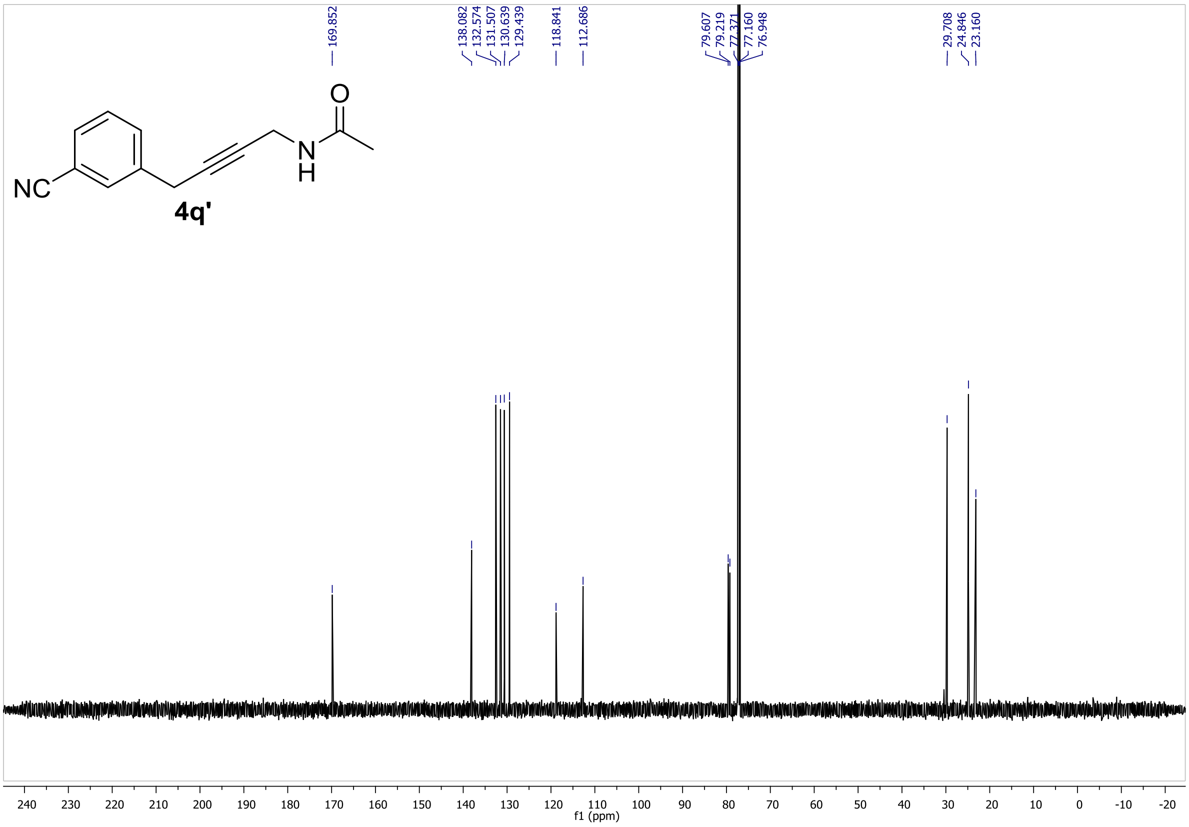

N-(4-(4-iodophenyl)but-2-yn-1-yl)acetamide (4r'):

^{1}H NMR, 600 MHz, $CDCl_3$:

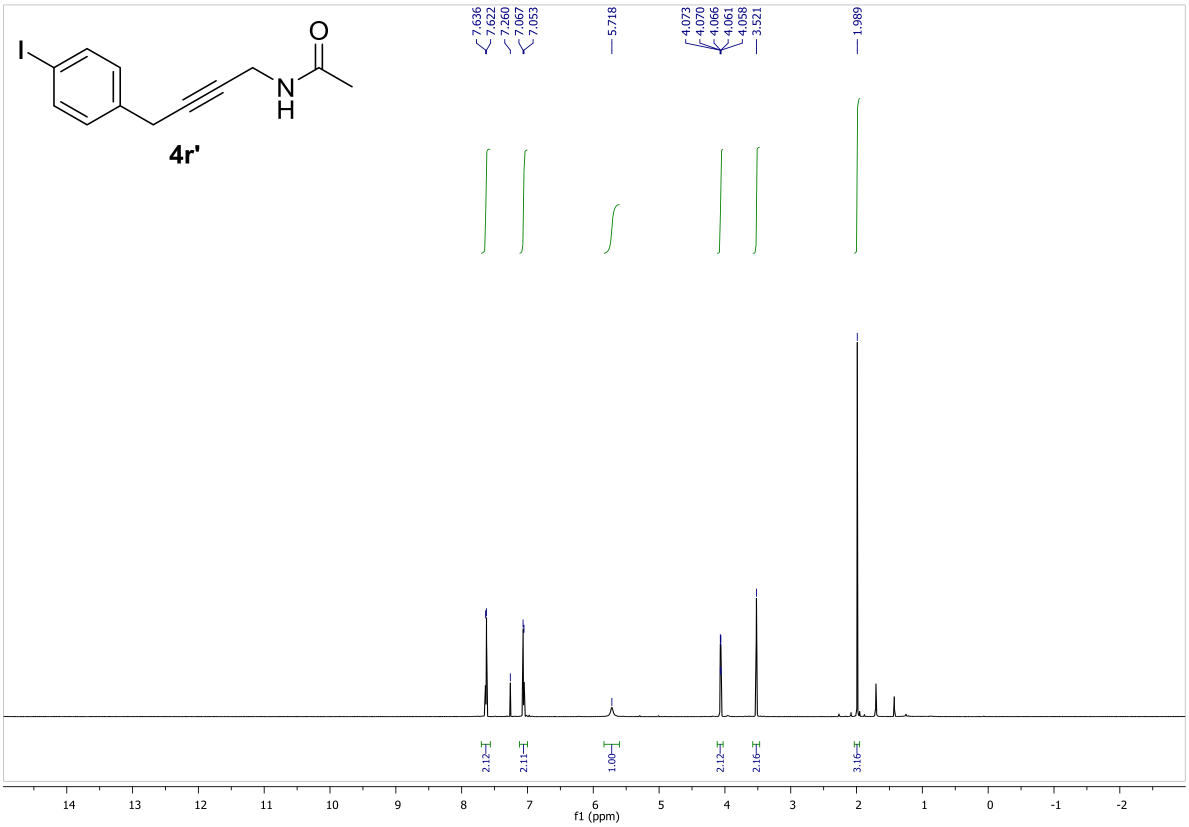

^{13}C NMR, 150 MHz, $CDCl_3$:

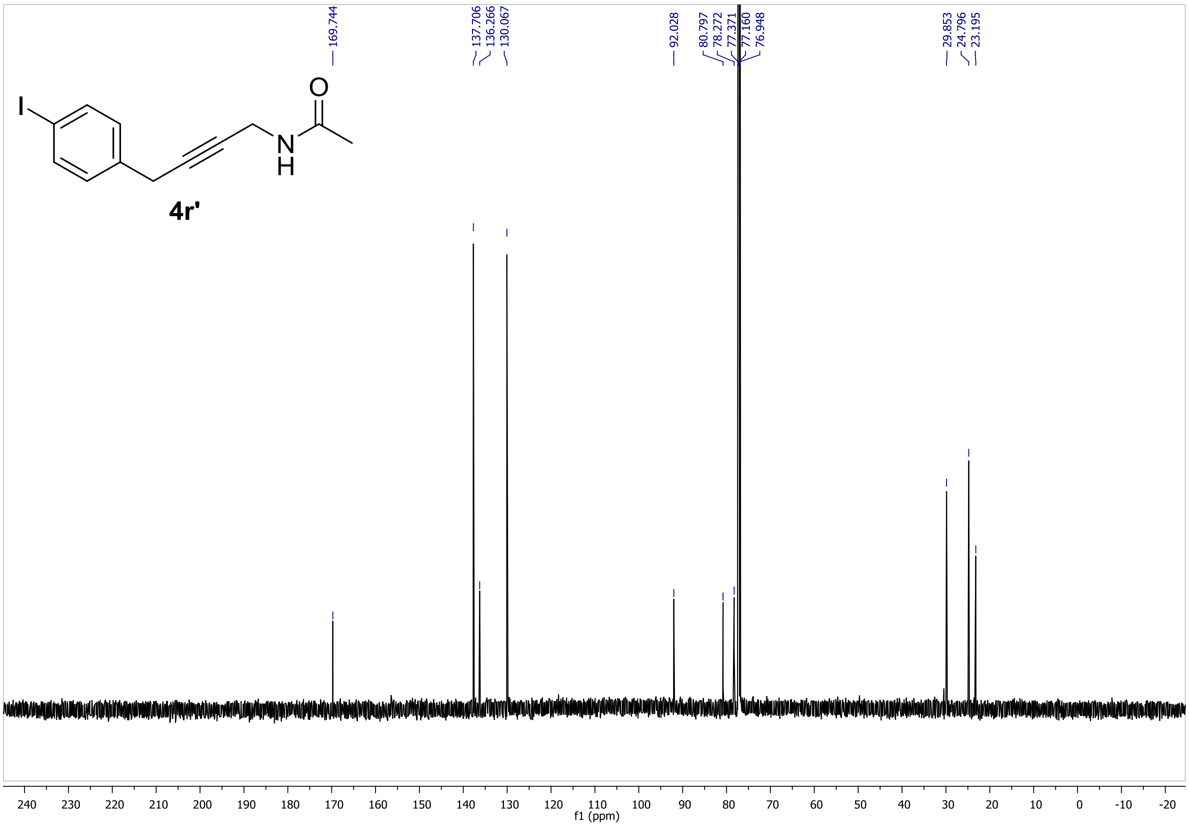

N-(4-(4-bromophenyl)but-2-yn-1-yl)pent-4-enamide (4s'):

^{1}H NMR, 600 MHz, $CDCl_3$:

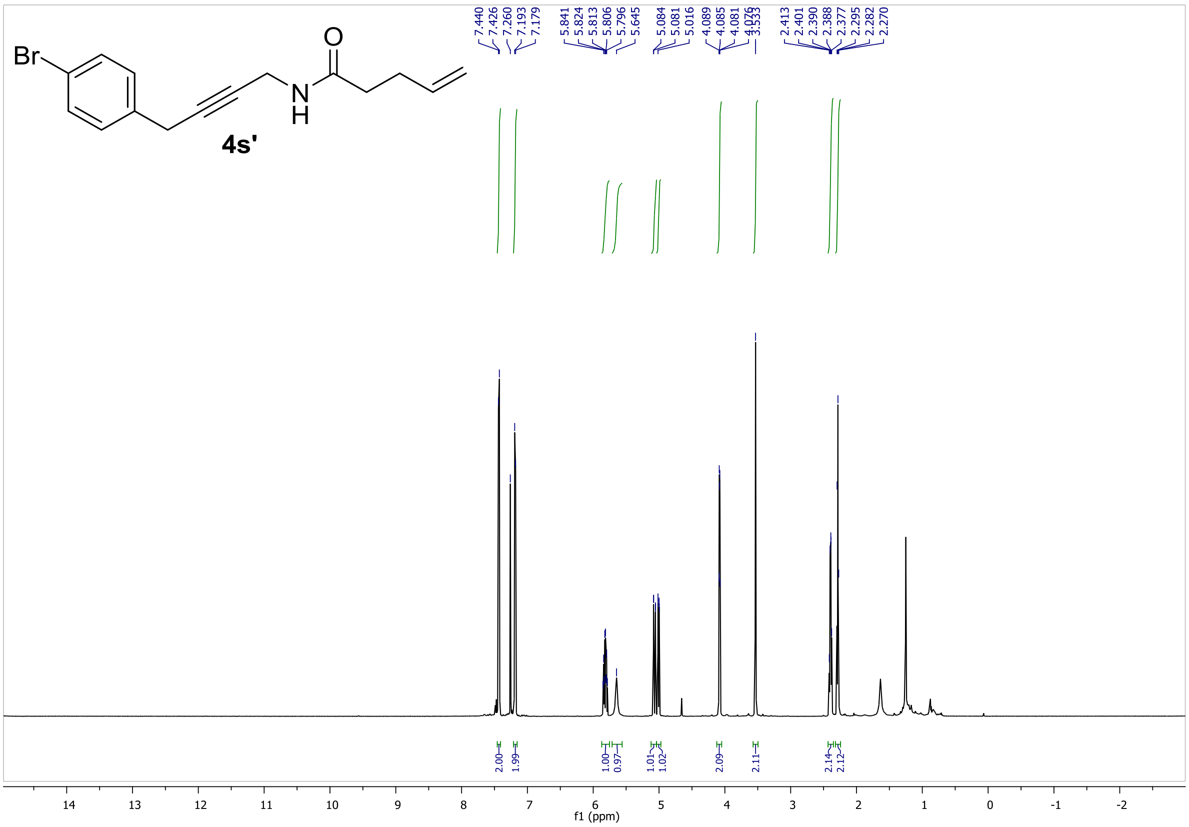

^{13}C NMR, 150 MHz, $CDCl_3$:

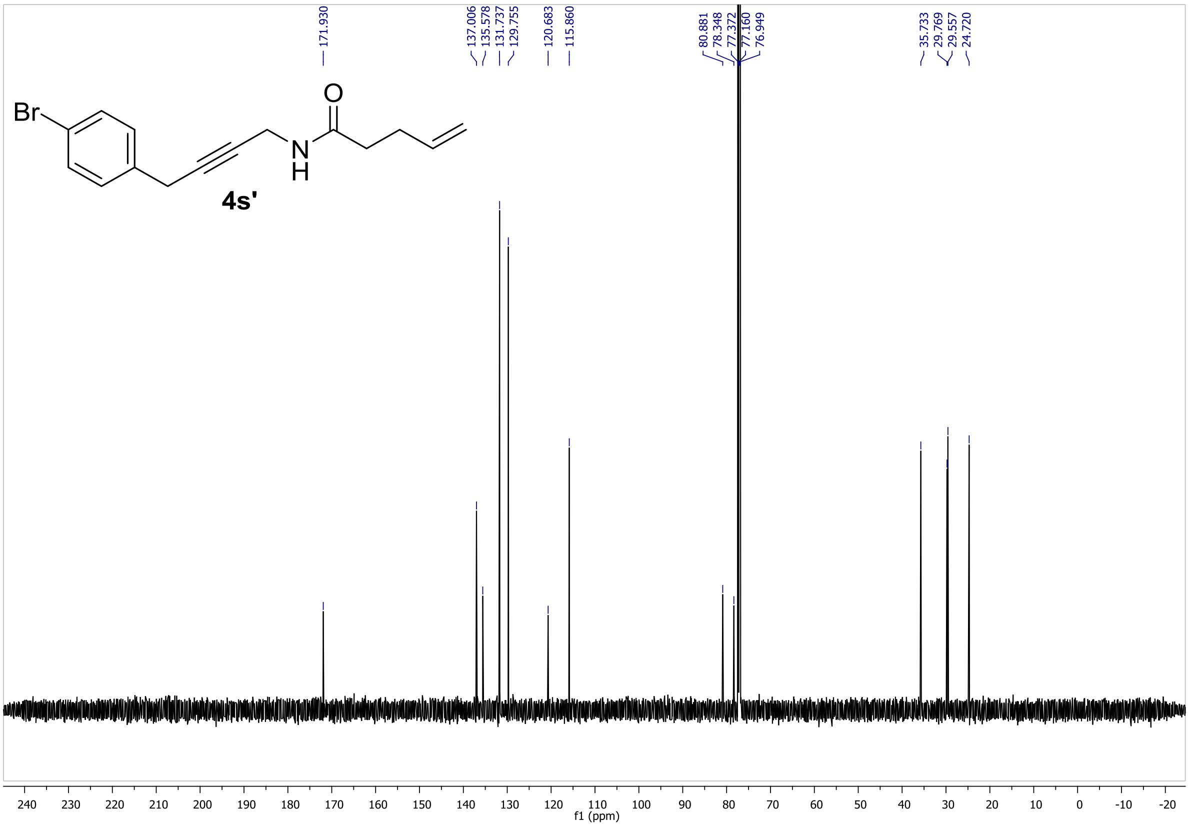

N-(4-(4-fluorophenyl)but-2-yn-1-yl)pent-4-enamide (4t'):

^{1}H NMR, 600 MHz, $CDCl_3$:

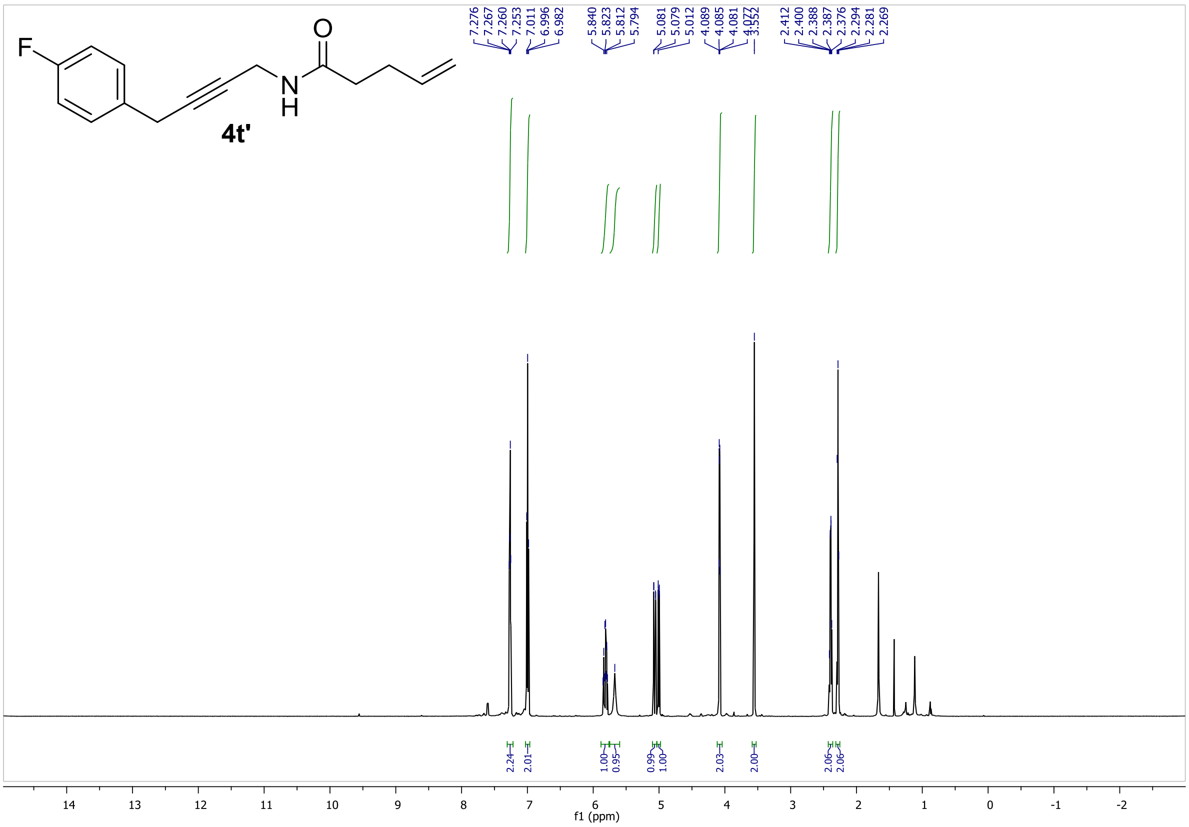

^{13}C NMR, 150 MHz, $CDCl_3$:

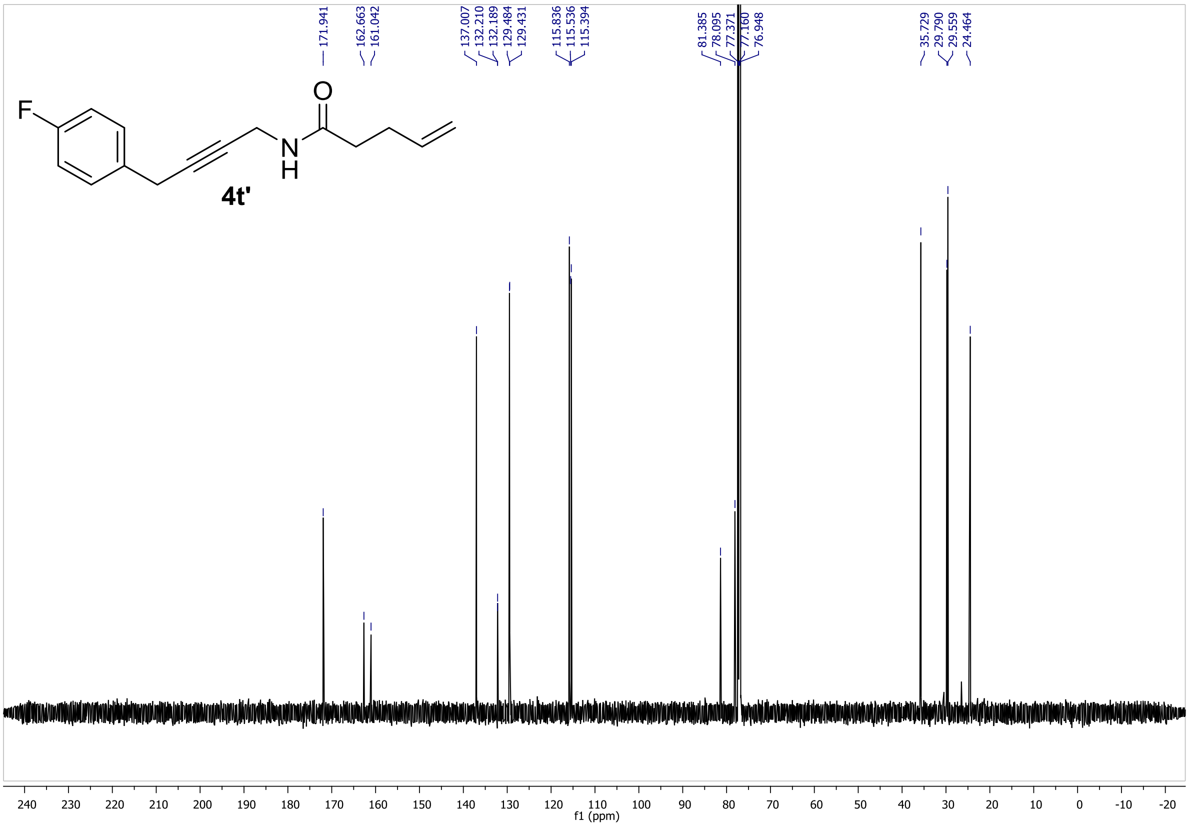

^{19}F NMR, 376 MHz, $CDCl_3$:

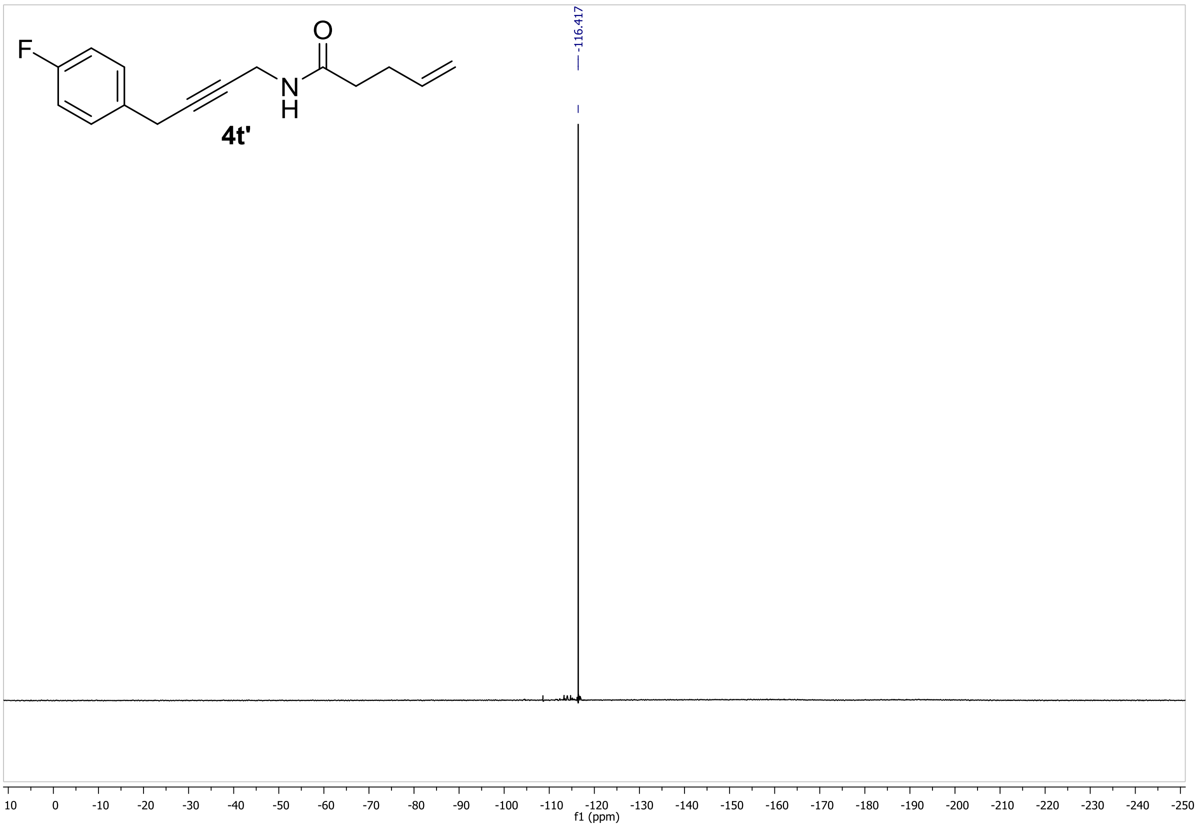

Methyl 4-((4-(3-methoxyphenyl)but-2-yn-1-yl)amino)-4-oxobutanoate (4u’):

^{1}H NMR, 600 MHz, $CDCl_3$:

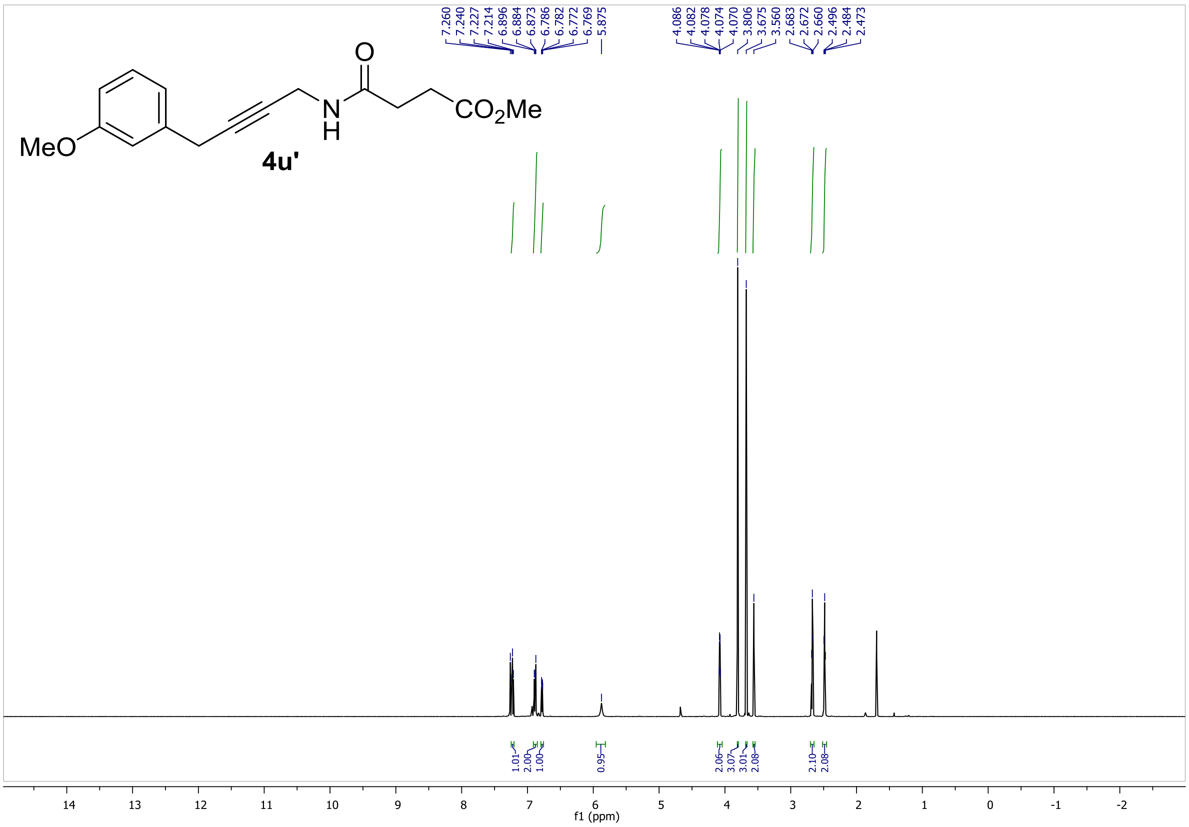

^{13}C NMR, 150 MHz, $CDCl_3$:

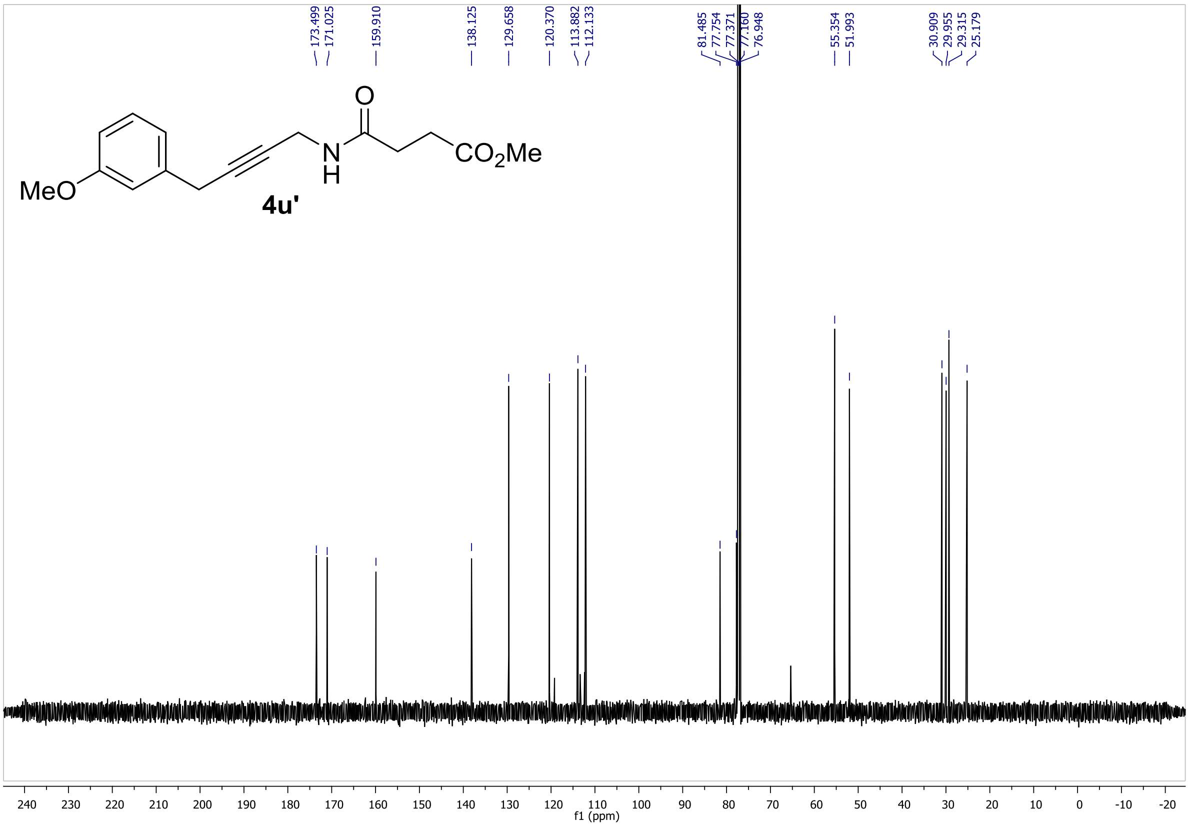

N-(4-(3-cyanophenyl)but-2-yn-1-yl)cyclopent-3-ene-1-carboxamide (4v'):

^{1}H NMR, 600 MHz, $CDCl_3$:

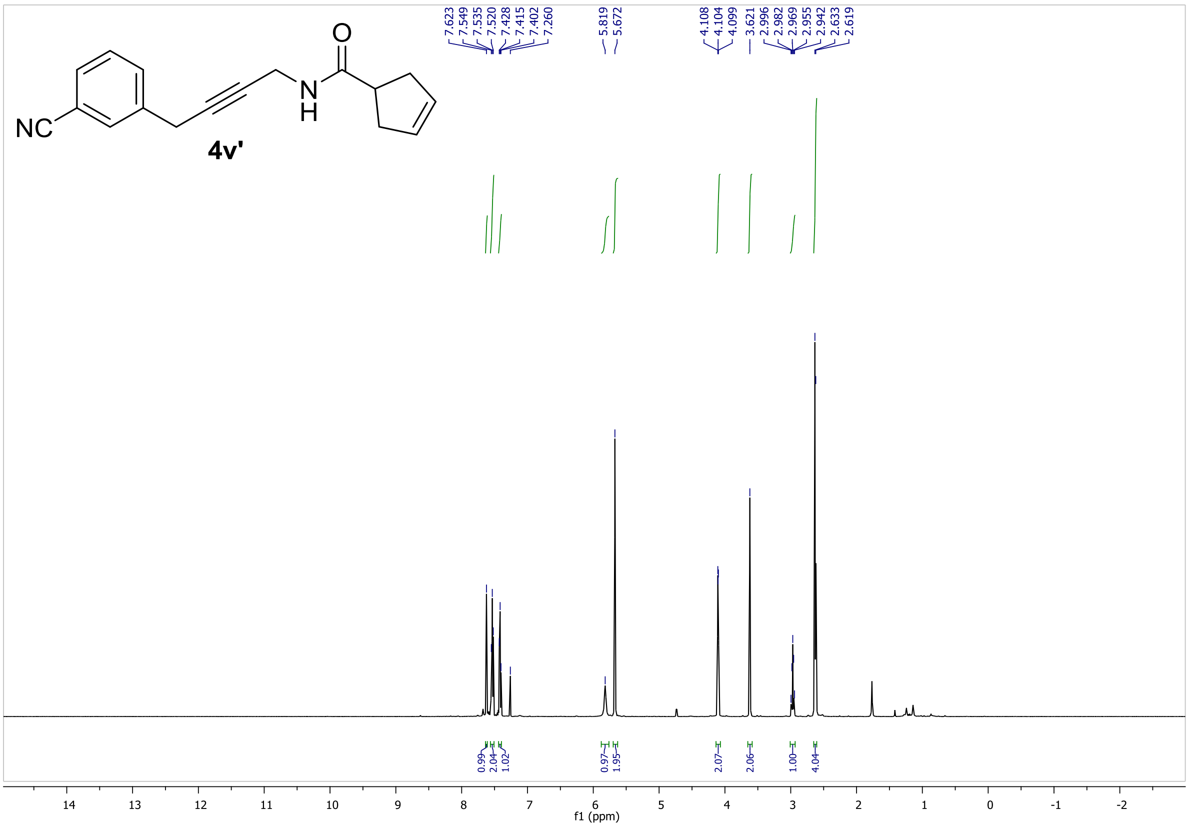

^{13}C NMR, 150 MHz, $CDCl_3$:

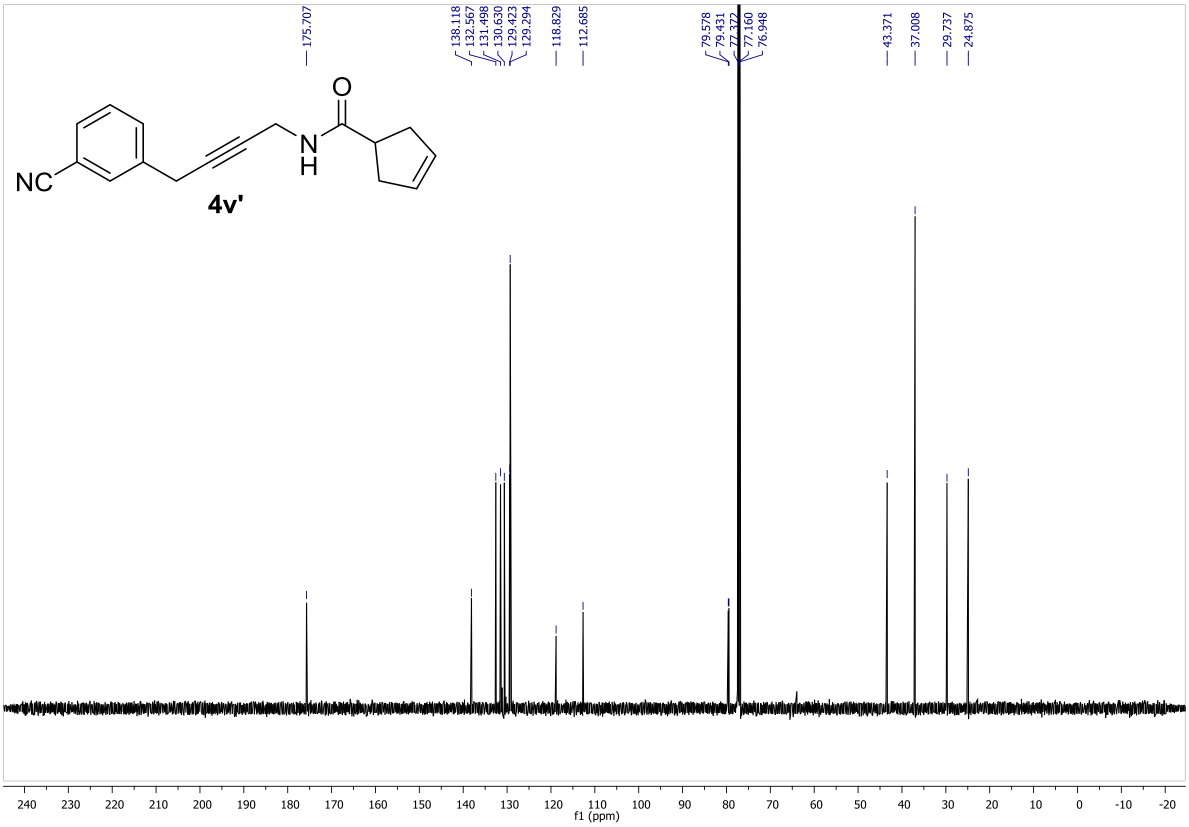

3,3,3-trifluoro-*N*-(4-(4-iodophenyl)but-2-yn-1-yl)propanamide (4w'):

^{1}H NMR, 600 MHz, $CDCl_3$:

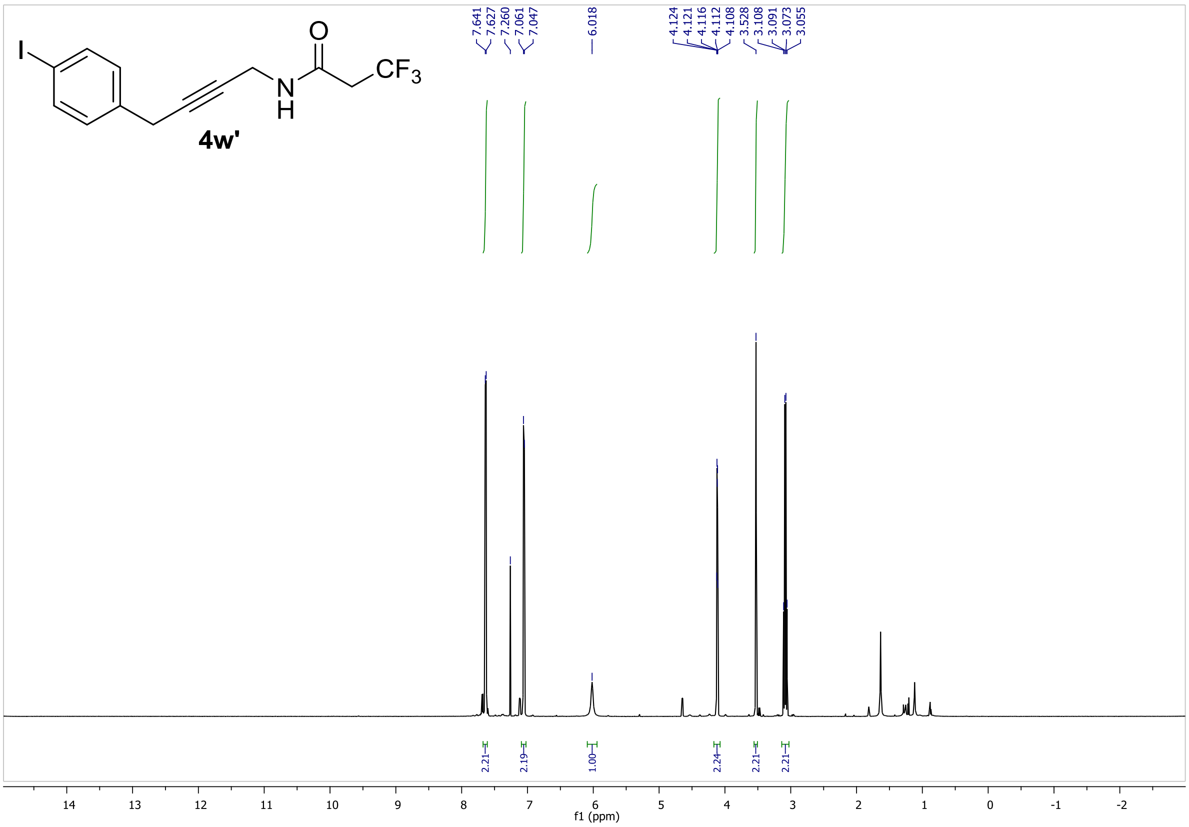

^{13}C NMR, 150 MHz, $CDCl_3$:

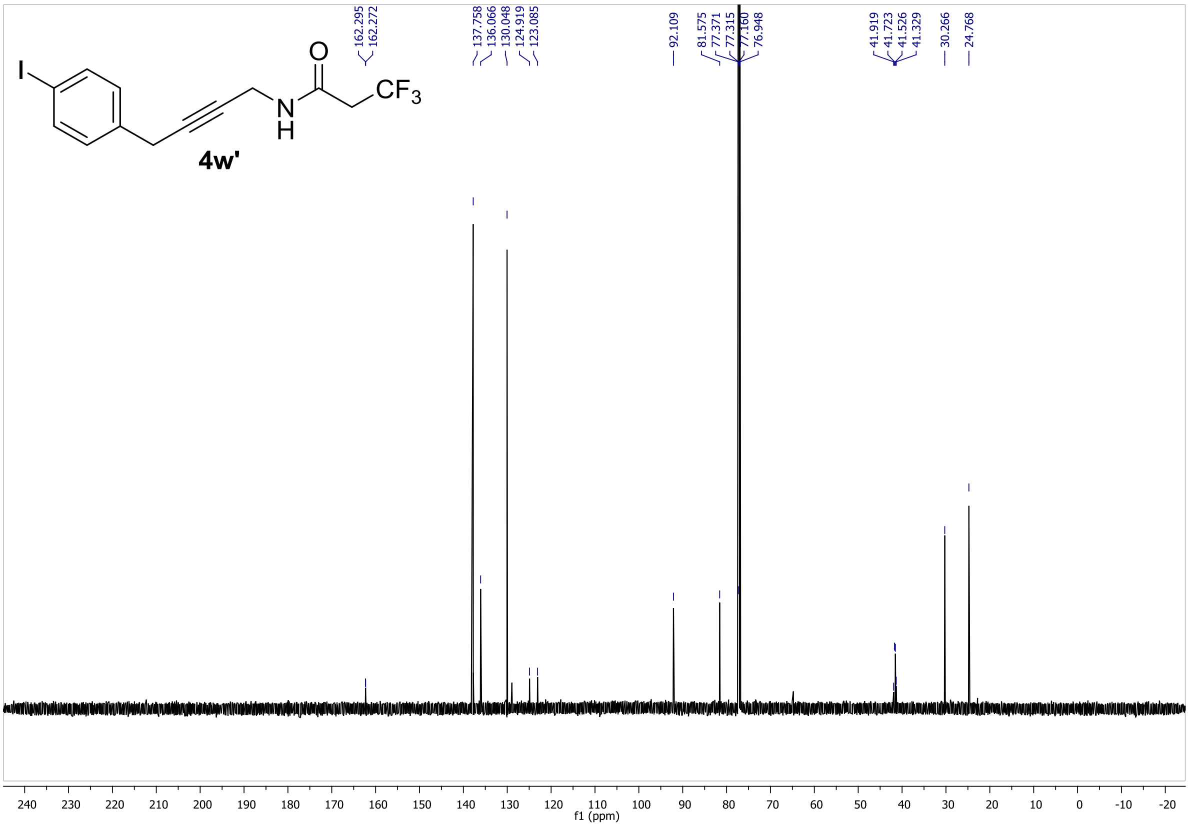

^{19}F NMR, 376 MHz, $CDCl_3$:

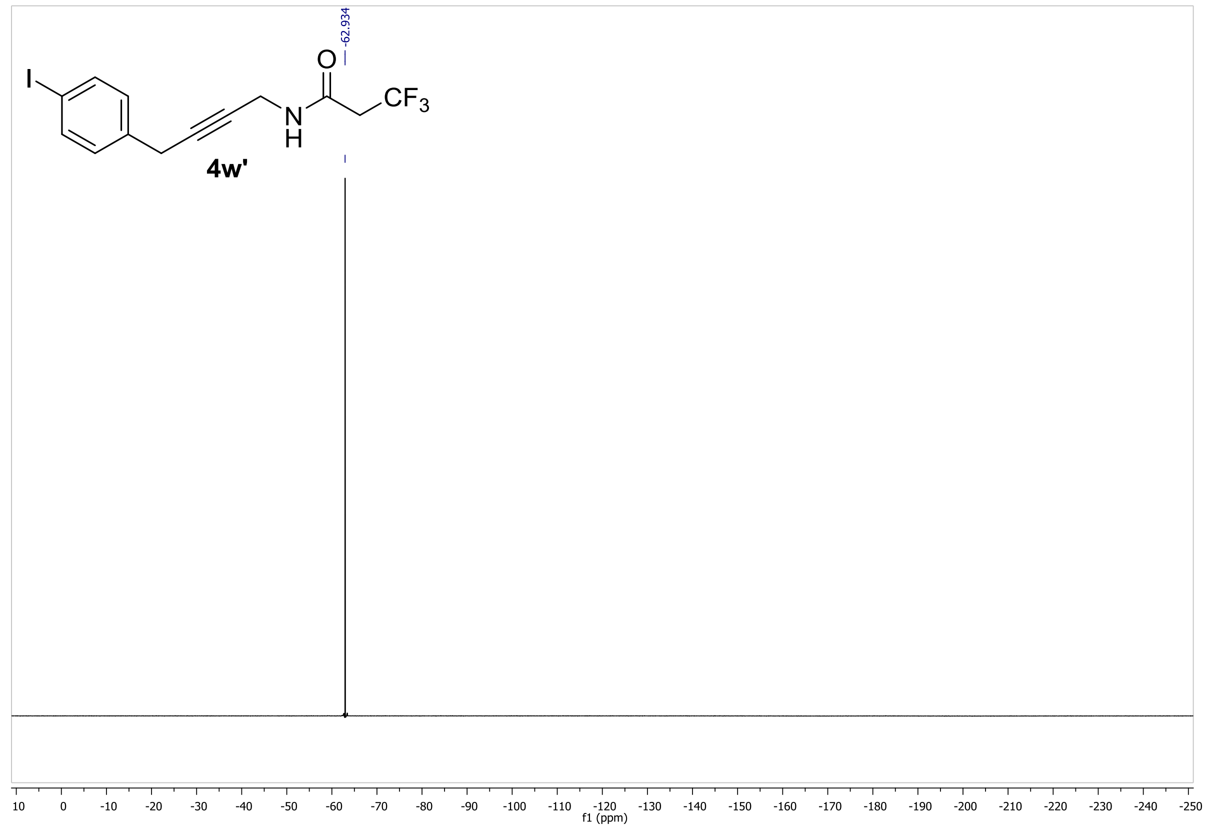

(*S*)-*N*-(4-(4-bromophenyl)but-2-yn-1-yl)-2-(4-isobutylphenyl)propanamide (4x'):

^{1}H NMR, 600 MHz, $CDCl_3$:

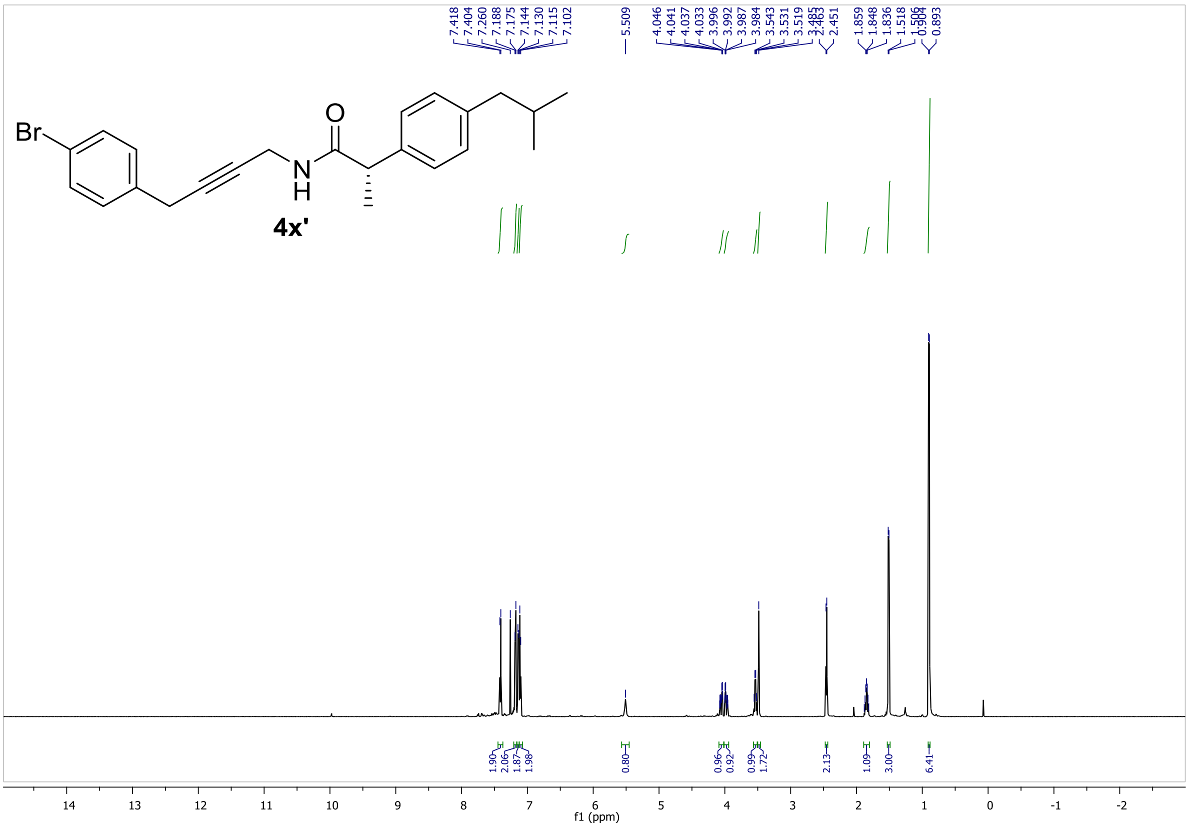

^{13}C NMR, 150 MHz, $CDCl_3$:

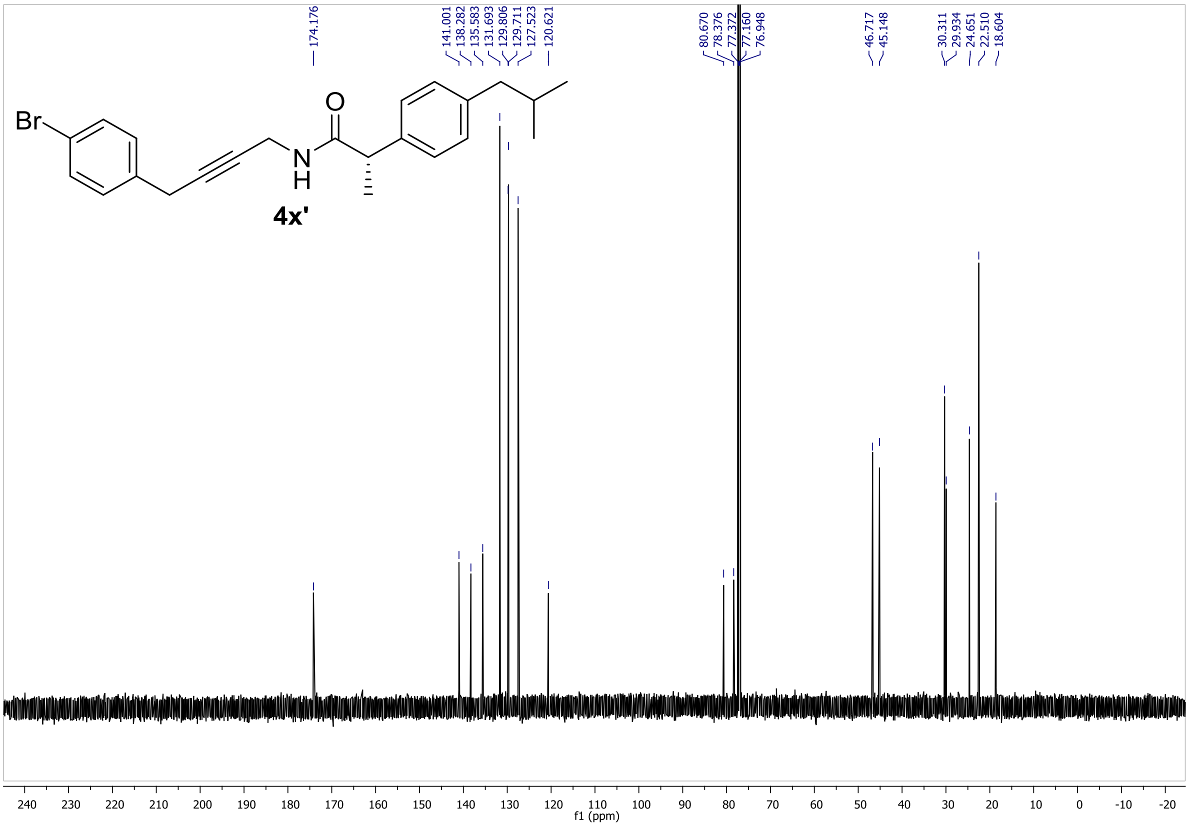

2-((4-(4-bromophenyl)but-2-yn-1-yl)carbamoyl)phenyl acetate (4y’):

^{1}H NMR, 600 MHz, $CDCl_3$:

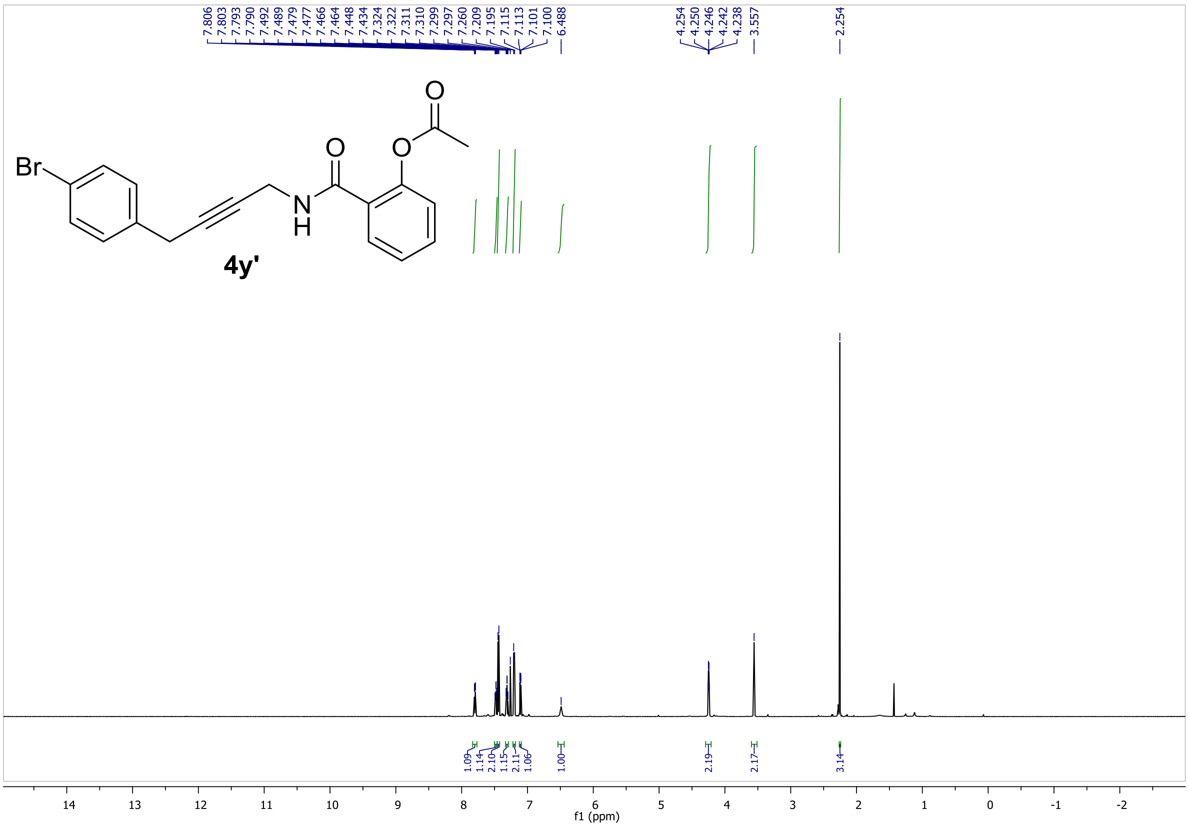

^{13}C NMR, 150 MHz, $CDCl_3$:

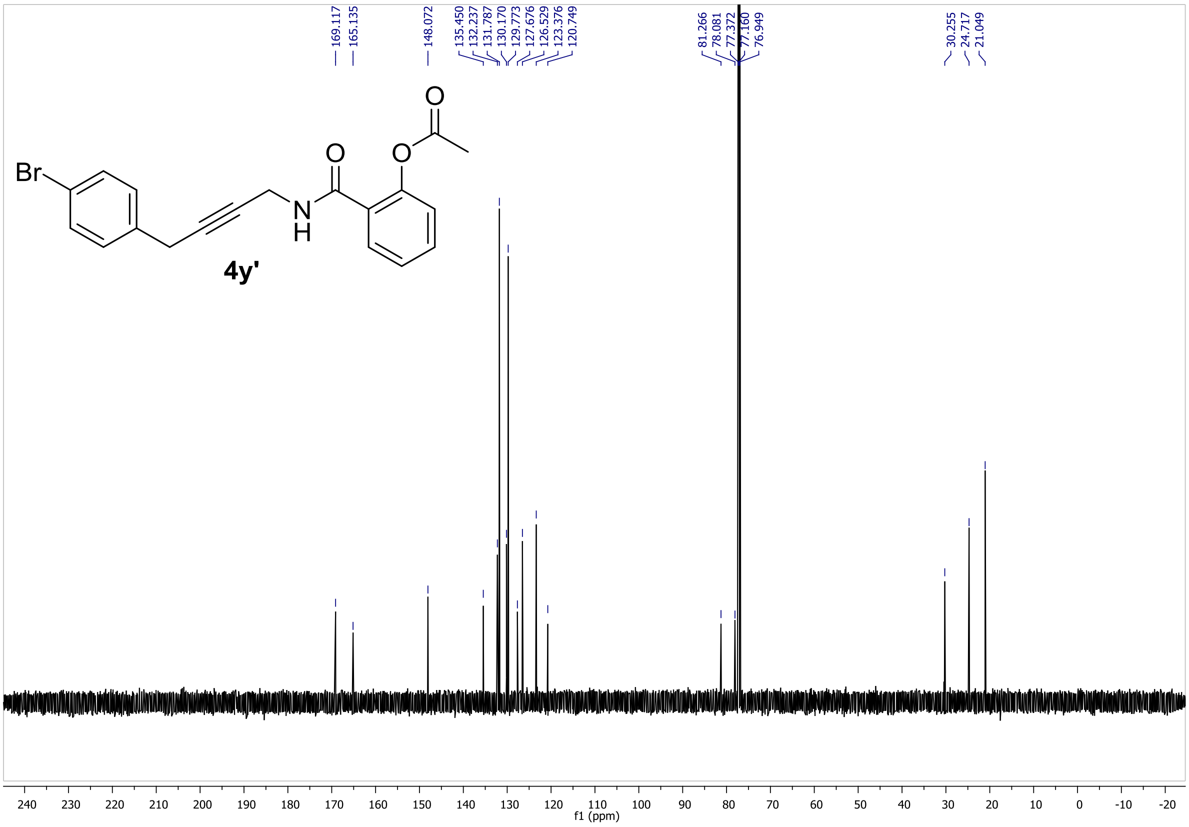

(2*S*,5*R*,6*R*)-*N*-(4-(4-bromophenyl)but-2-yn-1-yl)-3,3-dimethyl-7-oxo-6-(2-phenylacetamido)-4-thia-1-azabicyclo[3.2.0]heptane-2-carboxamide (4z'):

^{1}H NMR, 600 MHz, CDCl$_3$:

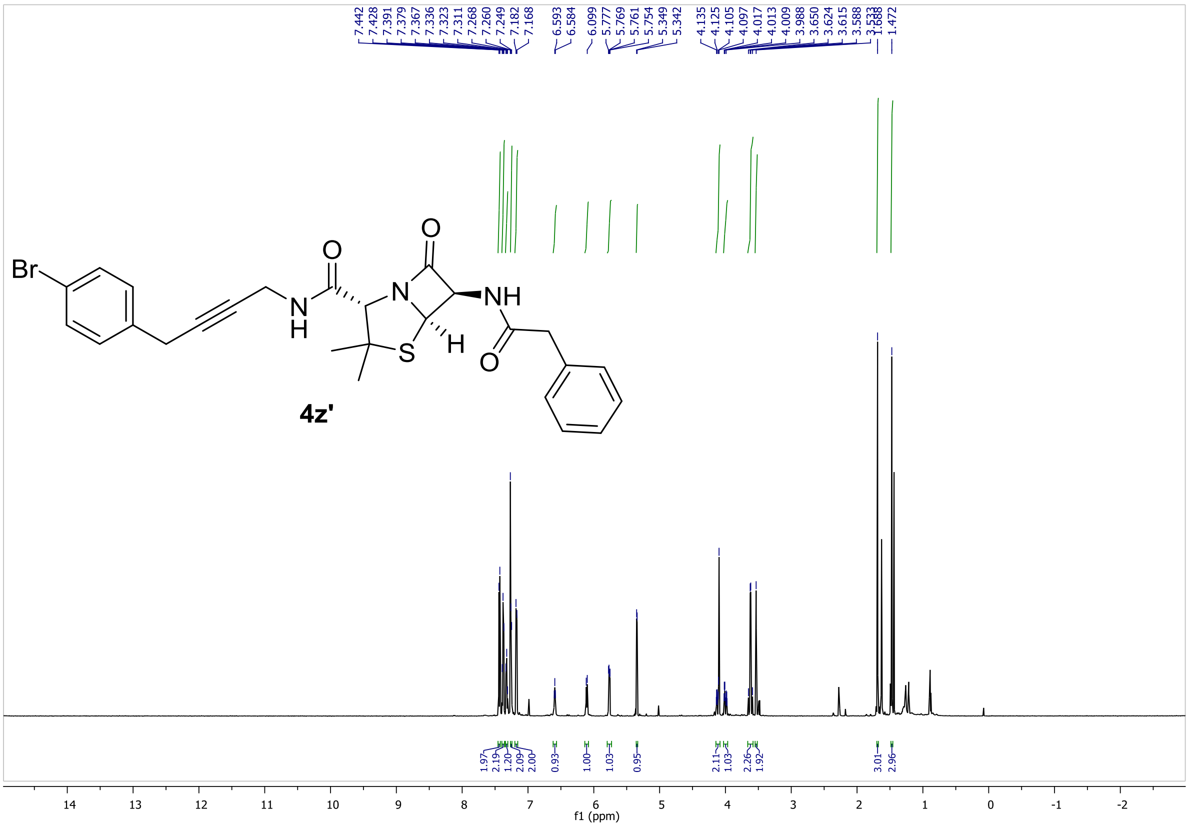

^{13}C NMR, 150 MHz, CDCl$_3$:

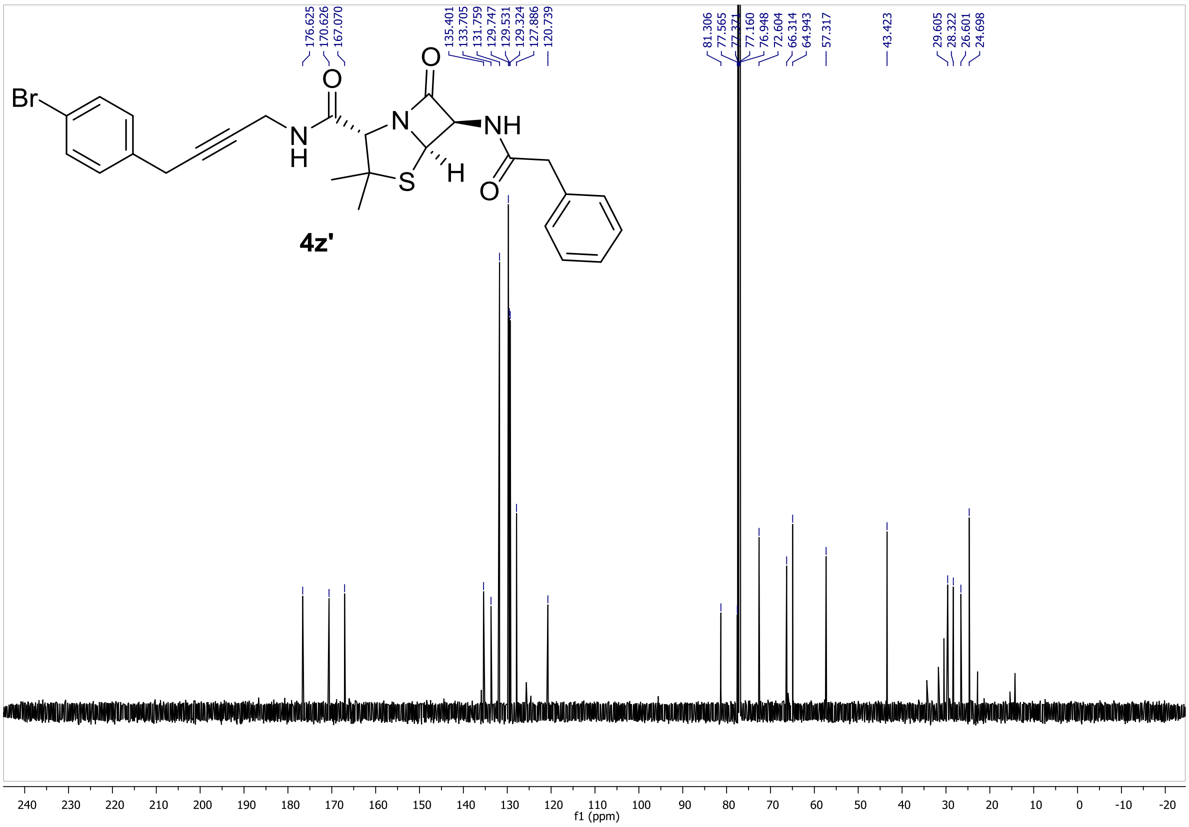

((3a*S*,5a*R*,8a*R*,8b*S*)-2,2,7,7-tetramethyltetrahydro-3a*H*-bis([1,3]dioxolo)[4,5-*b*:4',5'-*d*]pyran-3a-yl)methyl (4-(4-chlorophenyl)but-2-yn-1-yl)sulfamate (4aa'):

^{1}H NMR, 600 MHz, $CDCl_3$:

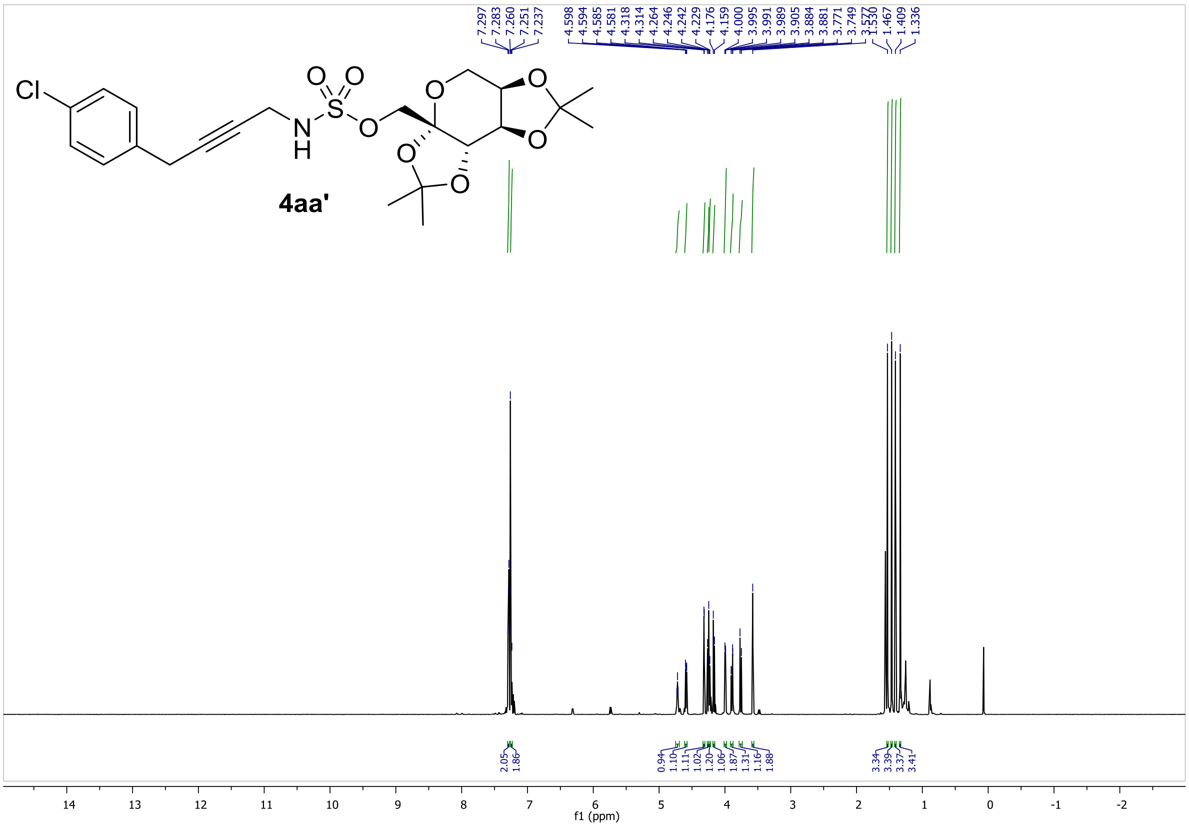

^{13}C NMR, 150 MHz, $CDCl_3$:

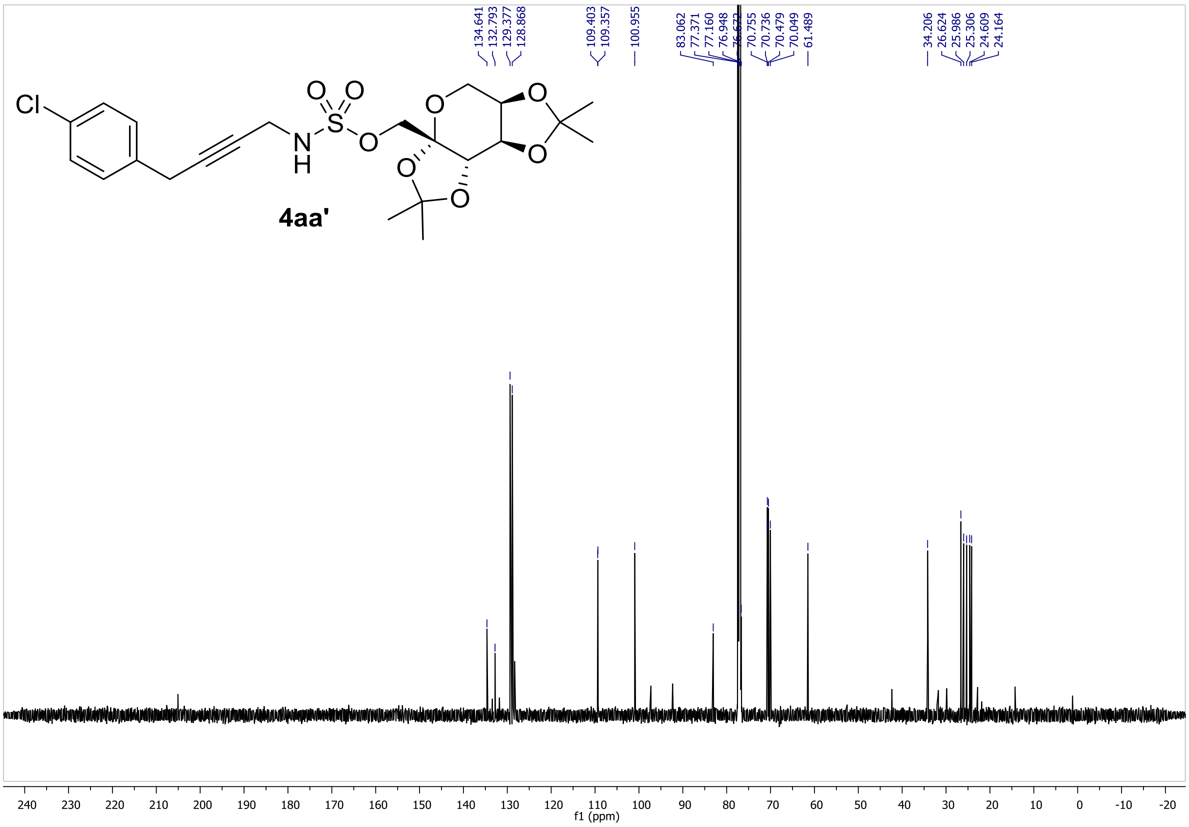

***tert*-butyl (*S*)-(2-(2-((4-(4-chlorophenyl)but-2-yn-1-yl)amino)-2-oxoethyl)-4-methylpentyl)carbamate (4ab'):**

^{1}H NMR, 600 MHz, $CDCl_3$:

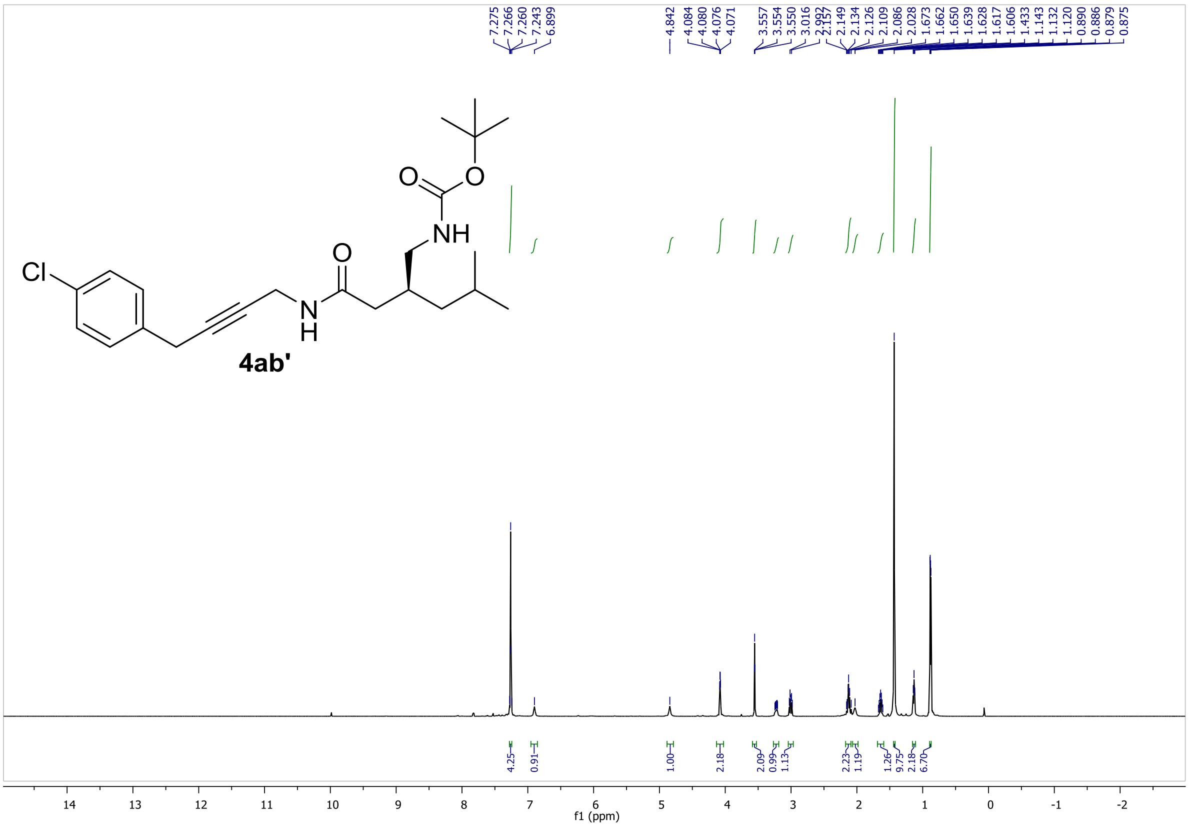

^{13}C NMR, 150 MHz, $CDCl_3$:

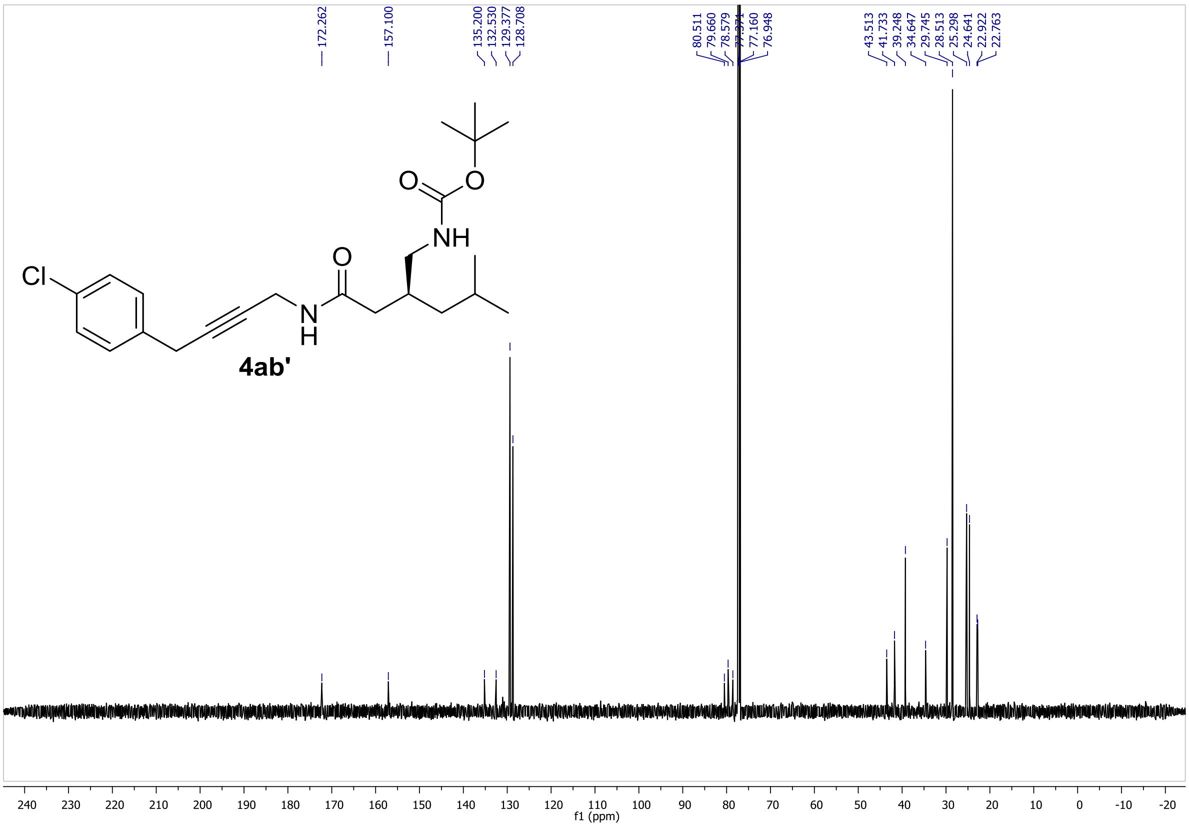

2-(1-(4-chlorobenzoyl)-5-methoxy-2-methyl-1*H*-indol-3-yl)-*N*-(4-(4-chlorophenyl)but-2-yn-1-yl)acetamide (4ac'):

^{1}H NMR, 600 MHz, $CDCl_3$:

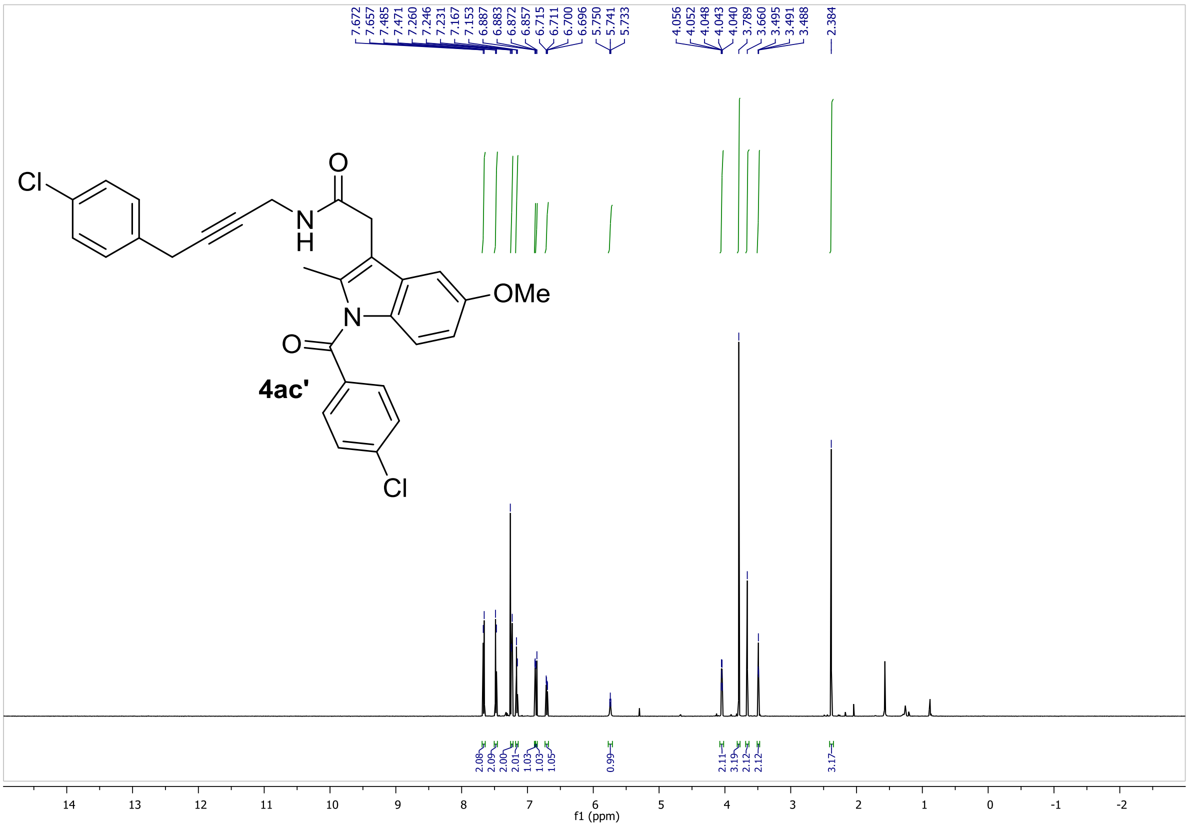

^{13}C NMR, 150 MHz, $CDCl_3$:

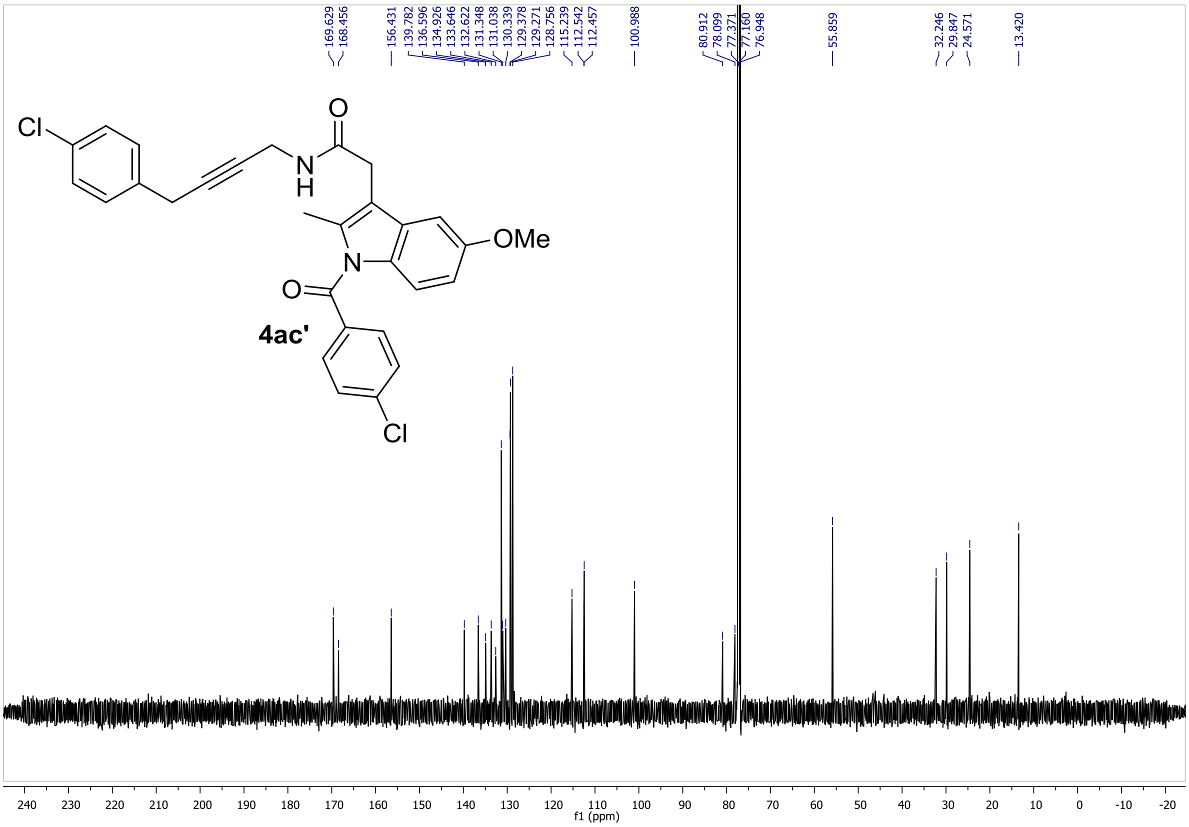

1-(2-((4*R*,6*R*)-6-(2-((4-(4-bromophenyl)but-2-yn-1-yl)amino)-2-oxoethyl)-2,2-dimethyl-1,3-dioxan-4-yl)ethyl)-5-(4-fluorophenyl)-2-isopropyl-*N*,4-diphenyl-1*H*-pyrrole-3-carboxamide (4ad'):

^{1}H NMR, 600 MHz, $CDCl_3$:

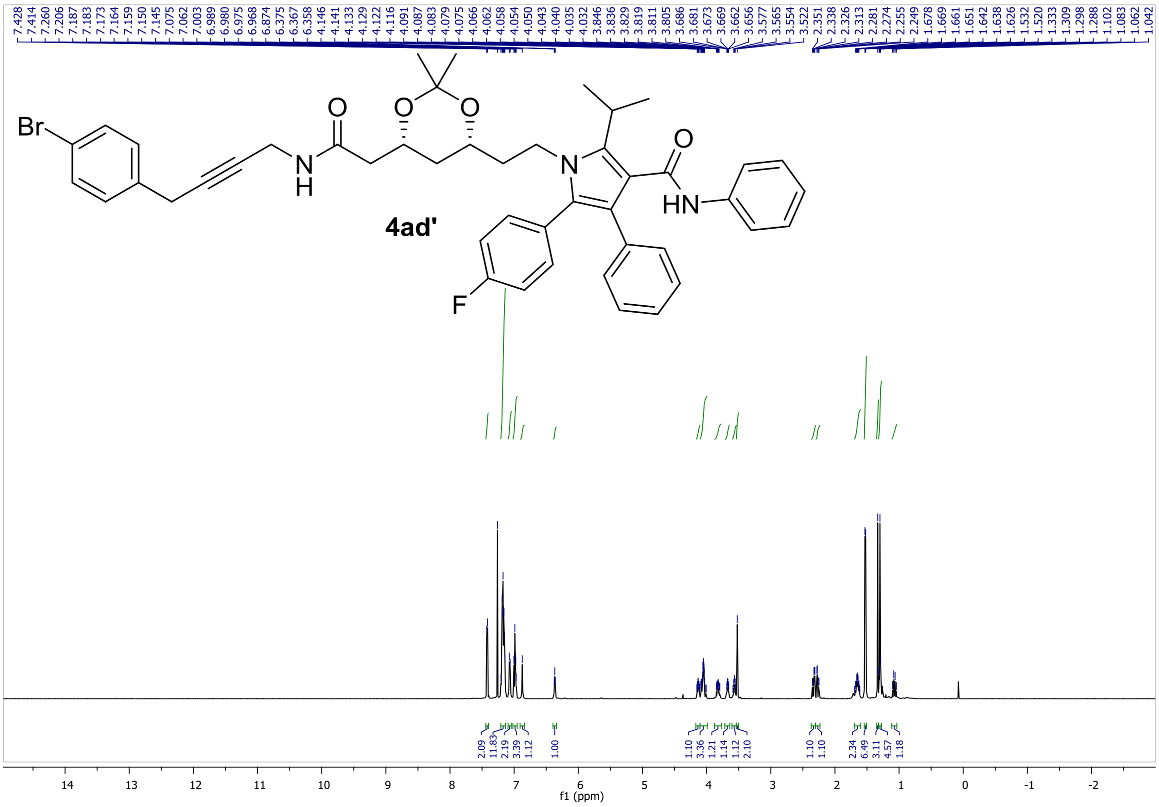

^{13}C NMR, 150 MHz, $CDCl_3$:

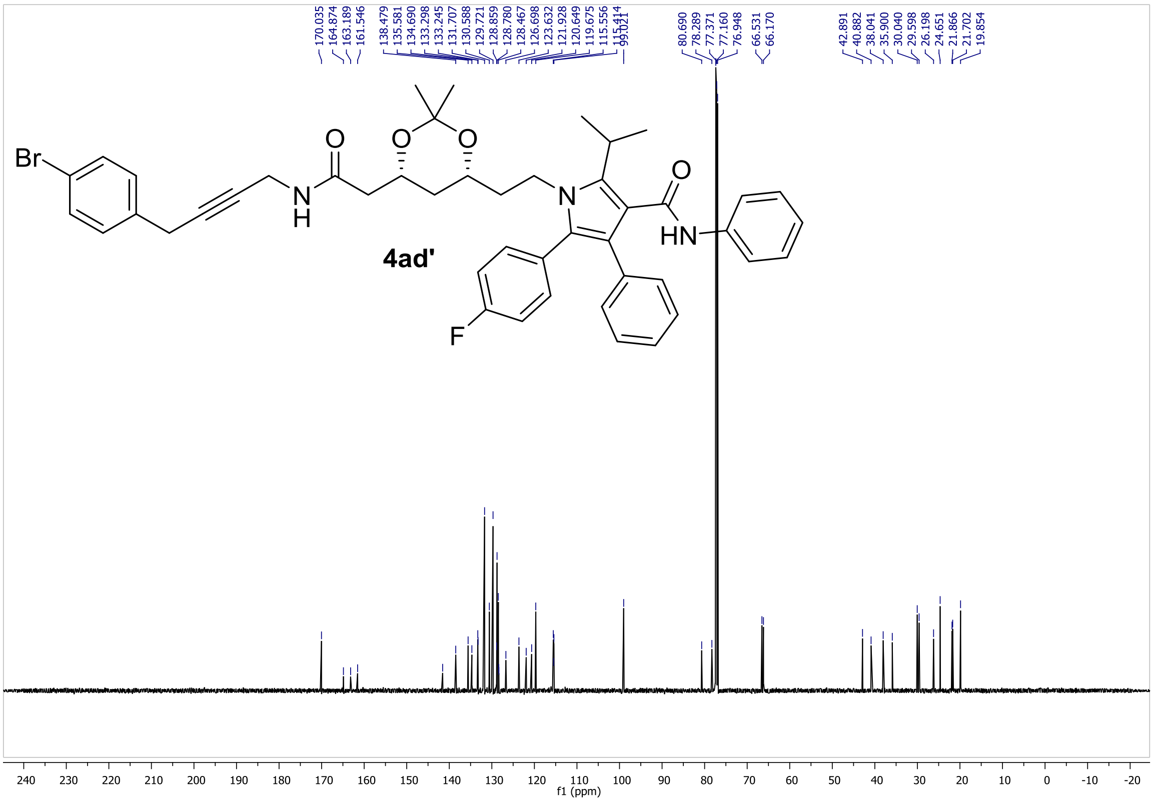

^{19}F NMR, 376 MHz, CDCl$_3$:

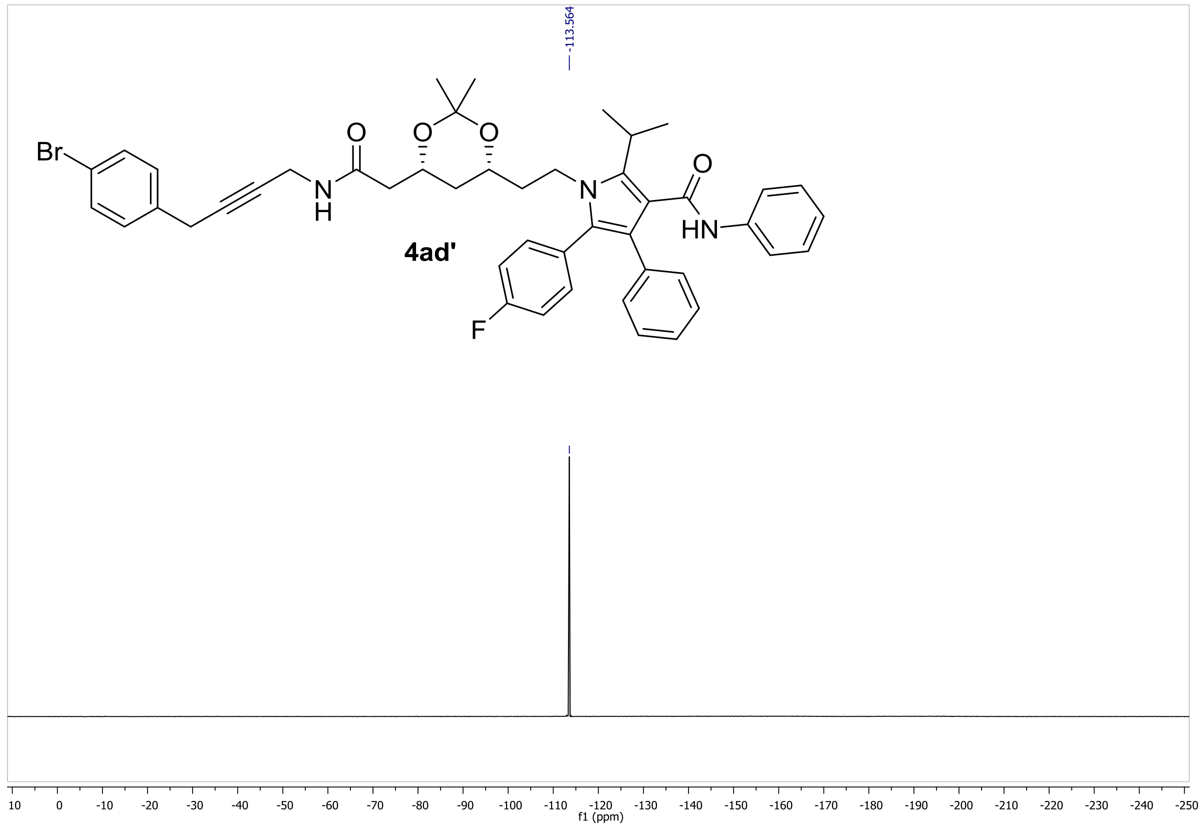

1-chloro-4-(3-cyclopropylprop-2-yn-1-yl)benzene:

^{1}H NMR, 600 MHz, $CDCl_3$:

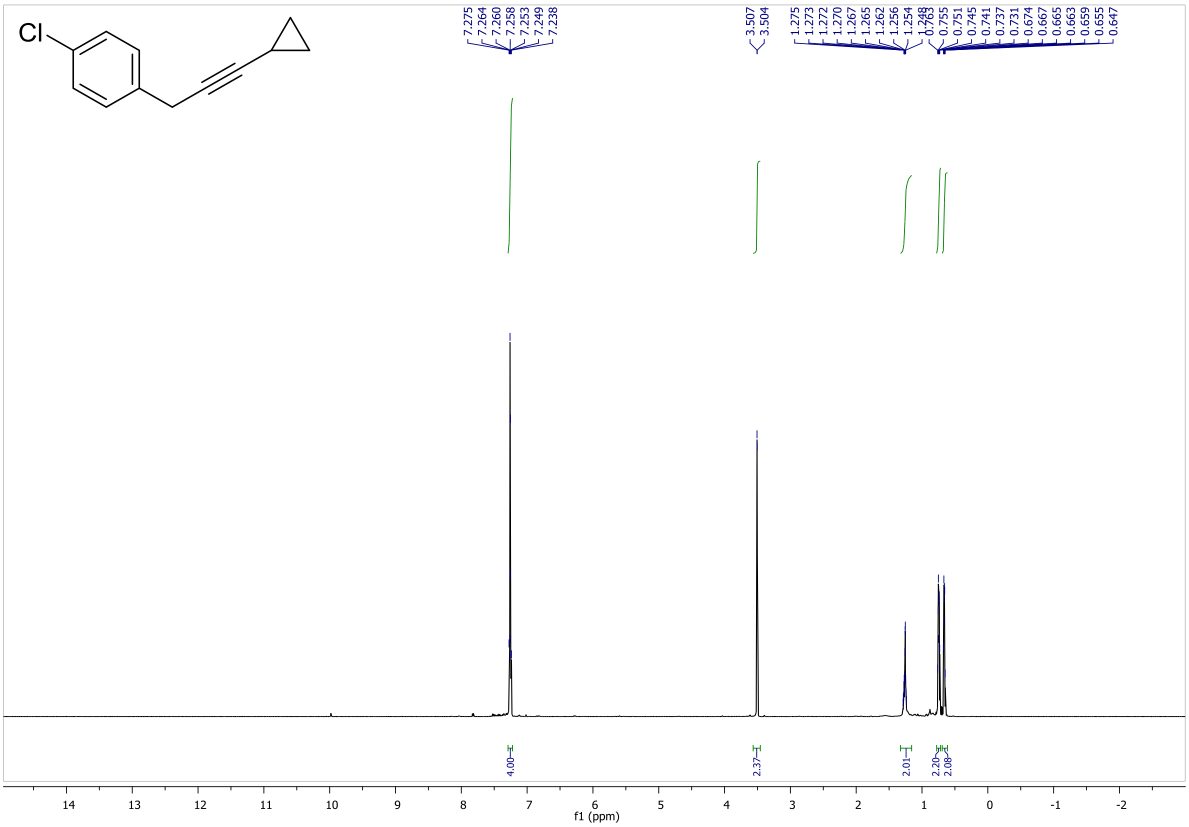

^{13}C NMR, 150 MHz, $CDCl_3$:

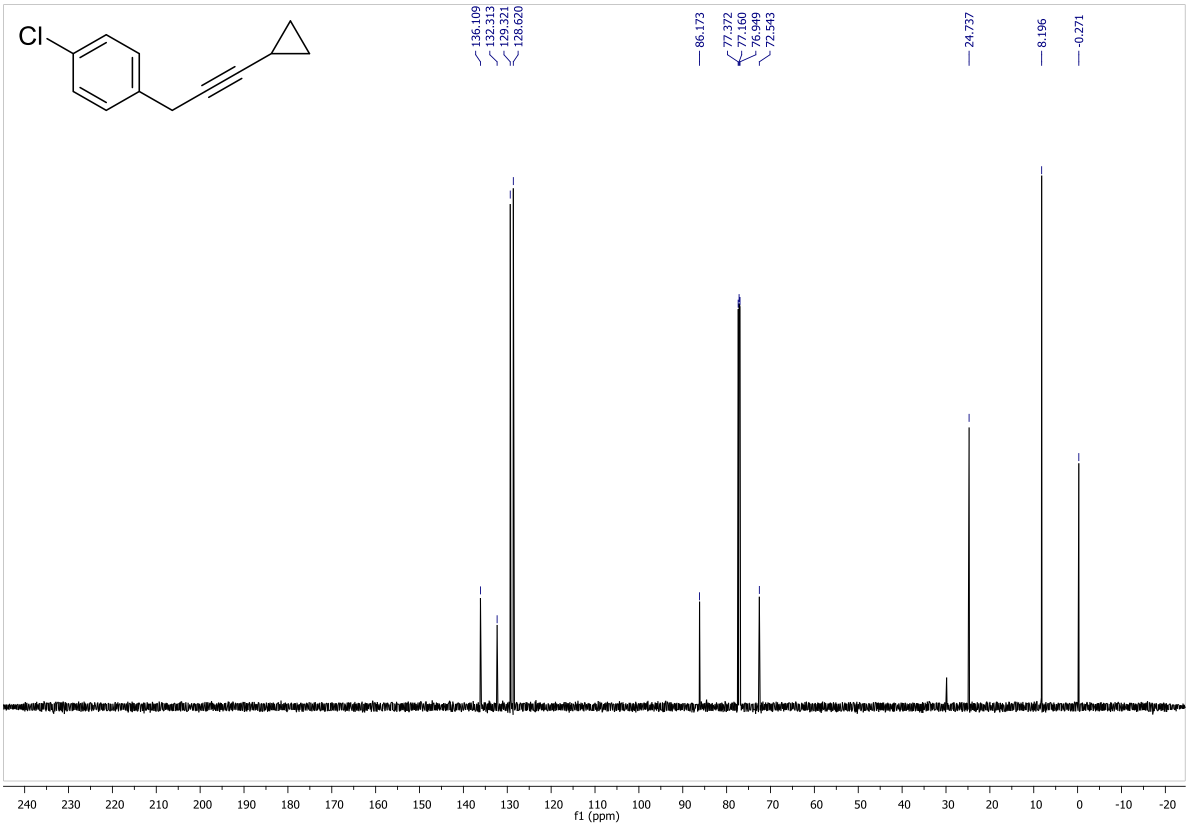

8.7. NMR spectra for pyrroline

(*S*)-2-(4-chlorophenyl)-1-tosyl-2,5-dihydro-1*H*-pyrrole (5):

^{1}H NMR, 600 MHz, $CDCl_3$:

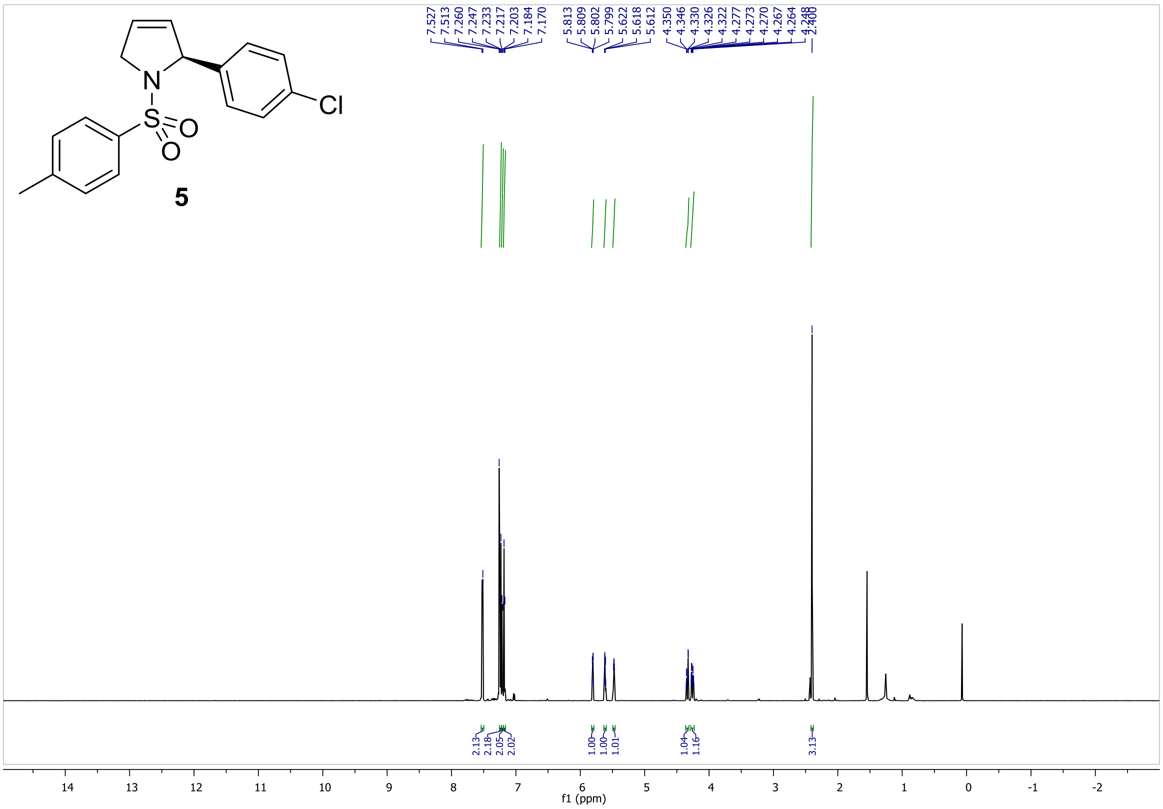

^{13}C NMR, 150 MHz, $CDCl_3$:

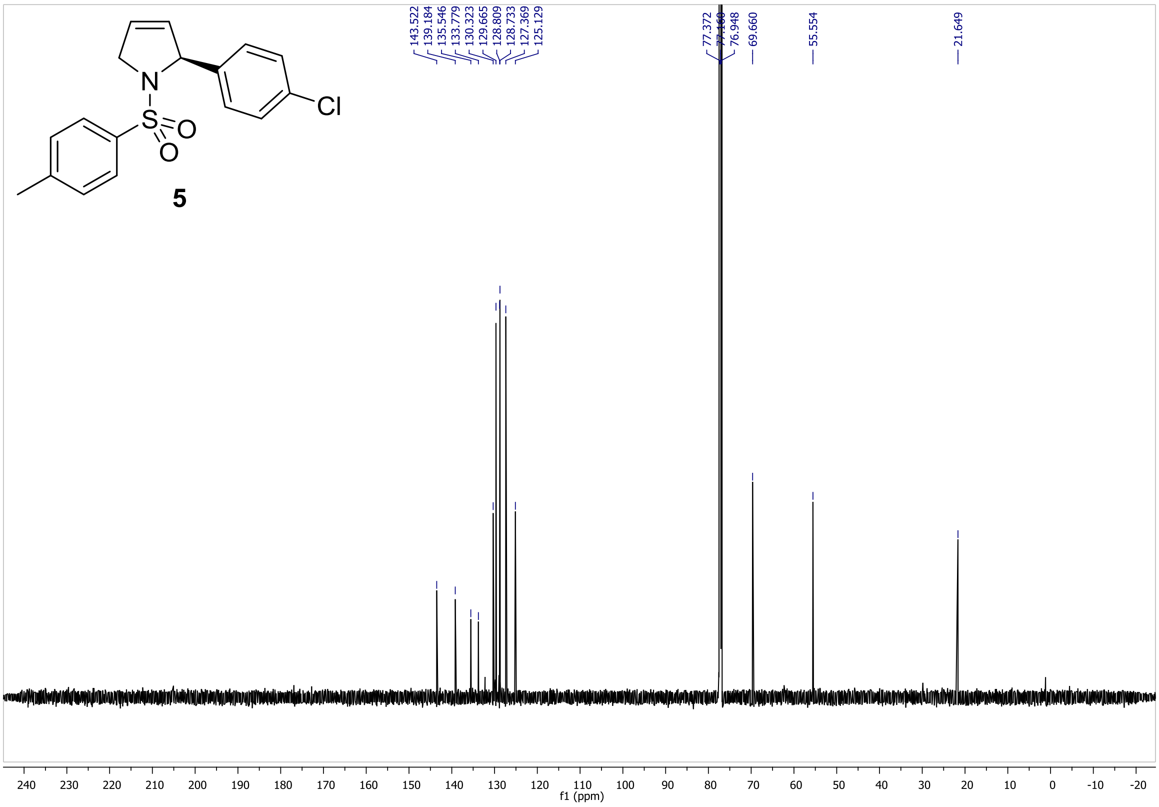

9. HPLC spectra

(*R*)-*N*-(4-(4-chlorophenyl)buta-2,3-dien-1-yl)-4-methylbenzenesulfonamide (4a):

HPLC conditions: Chiralpak AS, 90:10 hexane/isopropanol, 1.0 mL/min flow rate, T = 25 °C, λ_{max} = 254 nm; t_R (min) = 58.4 (minor), 75.5 (major).

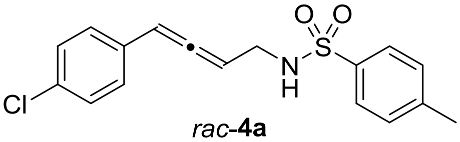

rac-**4a**

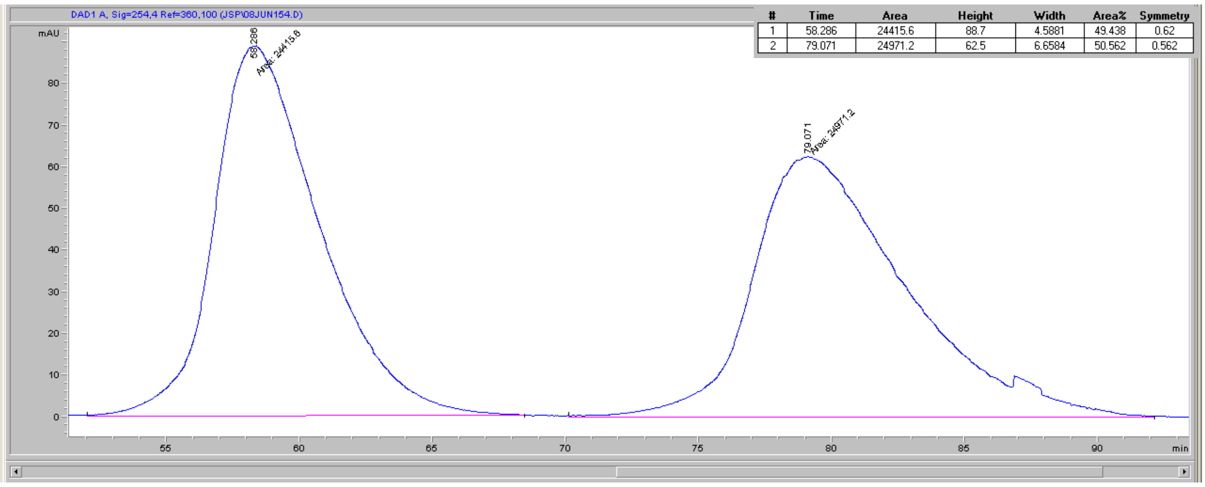

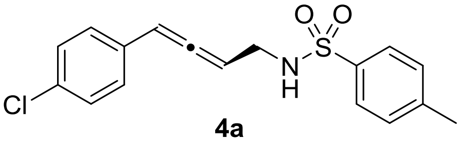

4a

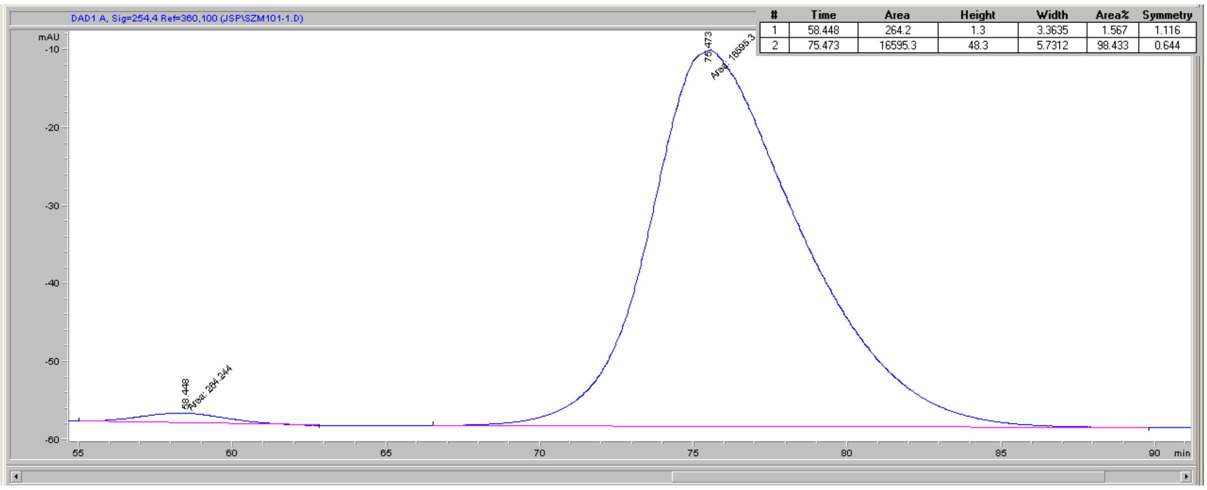

(*R*)-*N*-(4-(4-chlorophenyl)buta-2,3-dien-1-yl)-3,3,3-trifluoropropanamide (4b):

HPLC conditions: ChiralART SC, 95:5 hexane/isopropanol, 1.0 mL/min flow rate, T = 25 °C, λ_{max} = 254 nm; t_R (min) = 15.2 (minor), 16.6 (major).

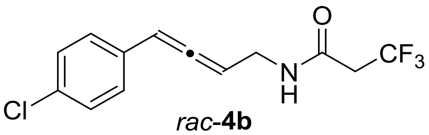

rac-**4b**

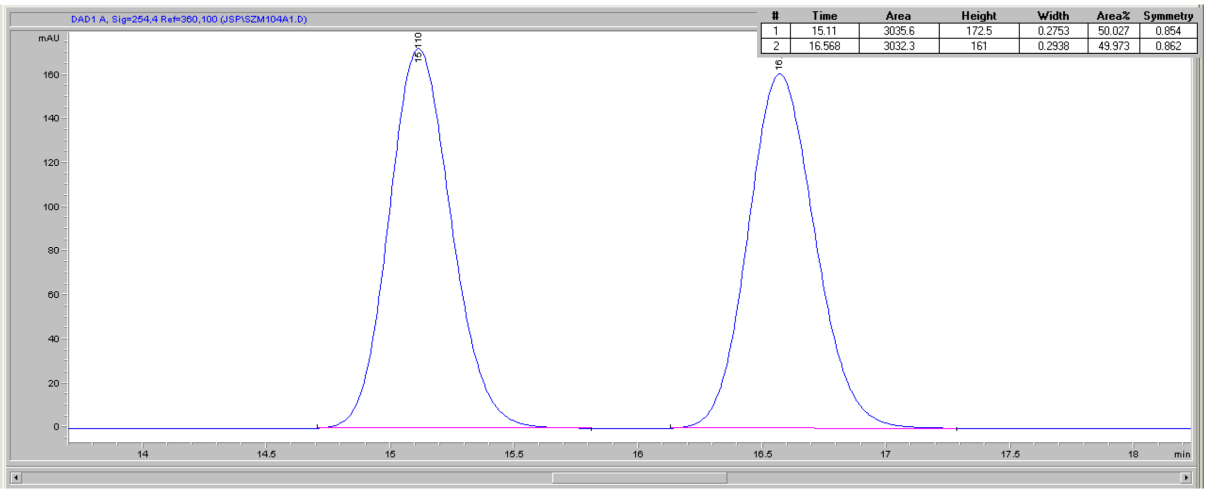

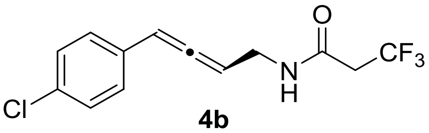

4b

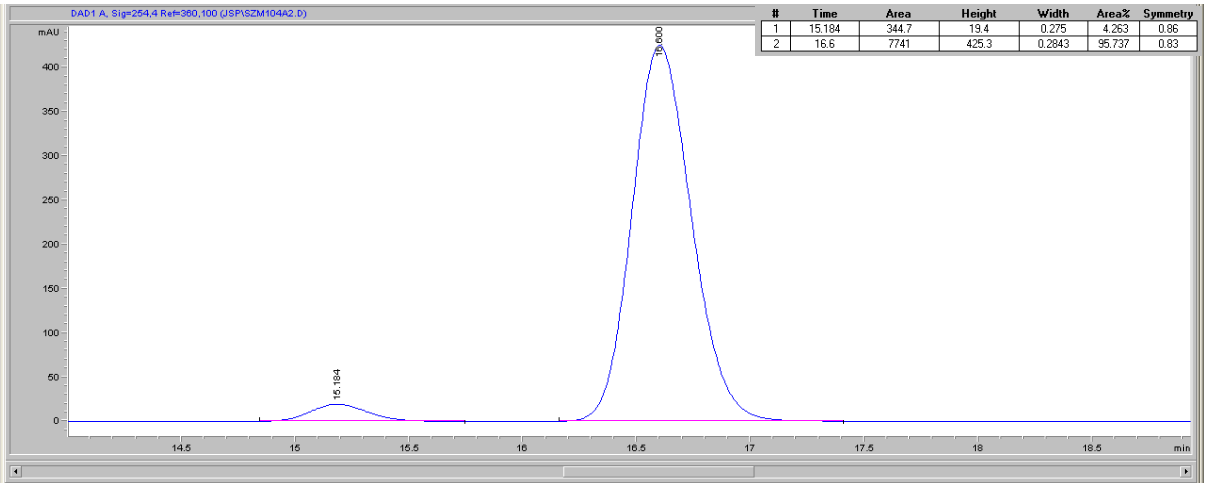

(*R*)-*N*-(4-(4-chlorophenyl)buta-2,3-dien-1-yl)-6-oxoheptanamide (4c):

HPLC conditions: ChiralART SC, 85:15 hexane/isopropanol, 1.0 mL/min flow rate, T = 25 °C, λ_{max} = 254 nm; t_R (min) = 33.8 (minor), 38.8 (major).

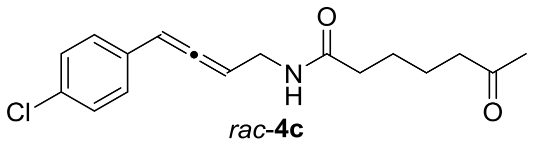

rac-**4c**

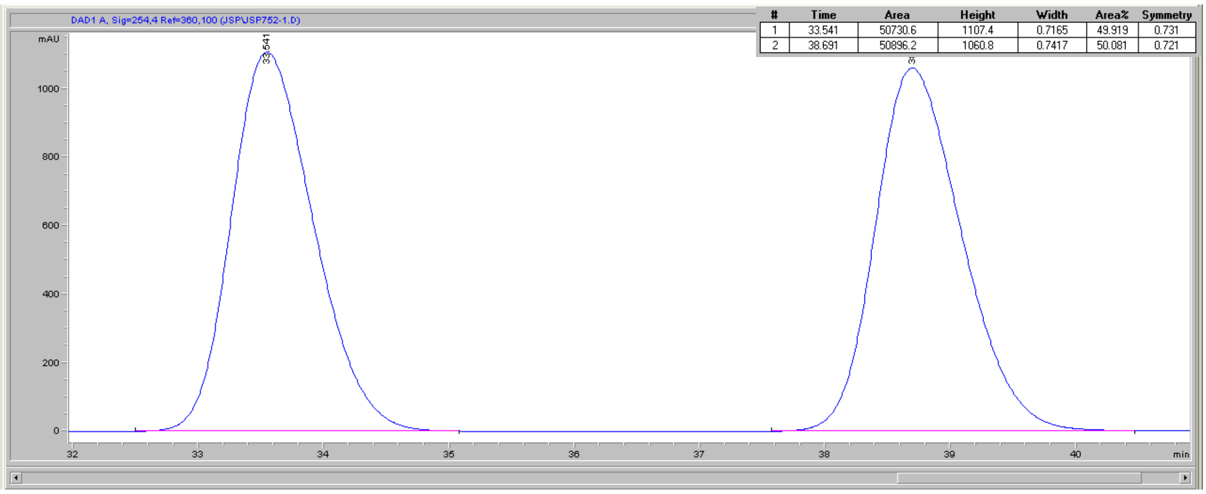

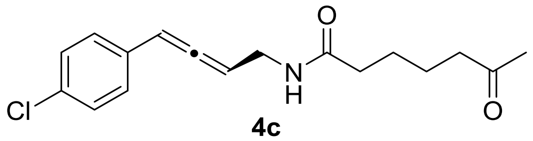

4c

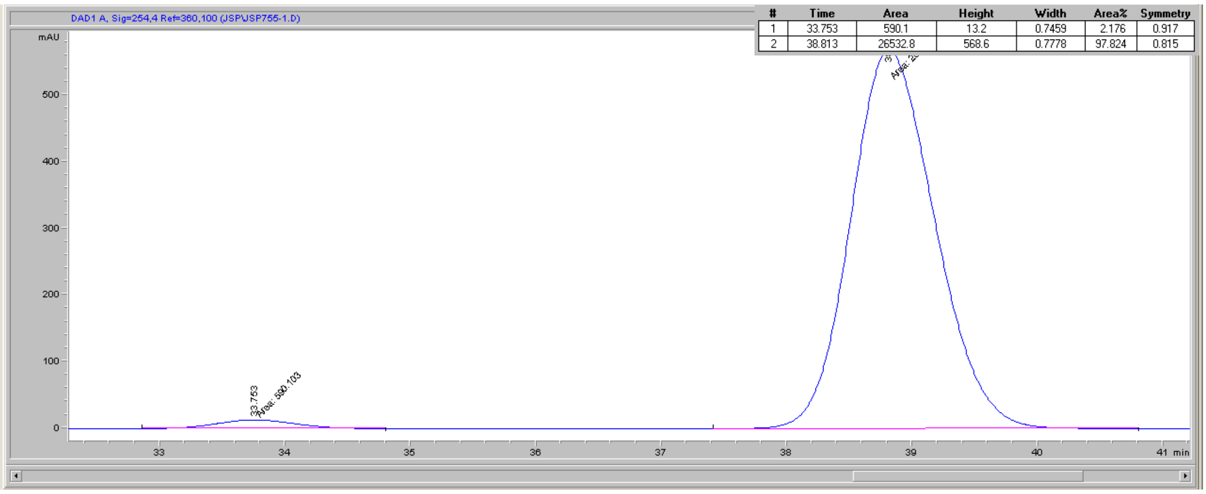

(*R*)-*N*-(4-(4-chlorophenyl)buta-2,3-dien-1-yl)-4-formylbenzamide (4d):

HPLC conditions: ChiralART SA, 93:7 hexane/isopropanol, 1.0 mL/min flow rate, T = 25 °C, λ_{max} = 254 nm; t_R (min) = 43.3 (minor), 45.0 (major).

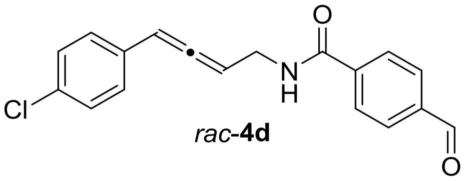

rac-**4d**

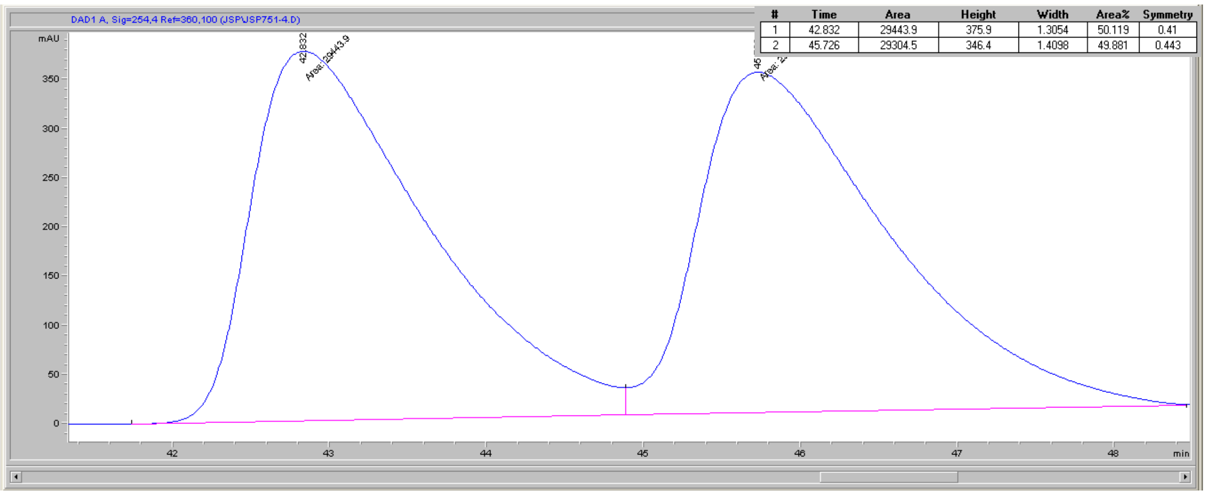

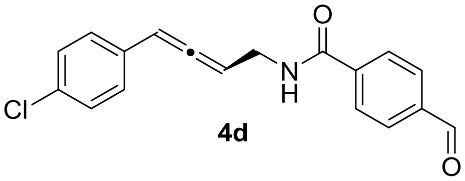

4d

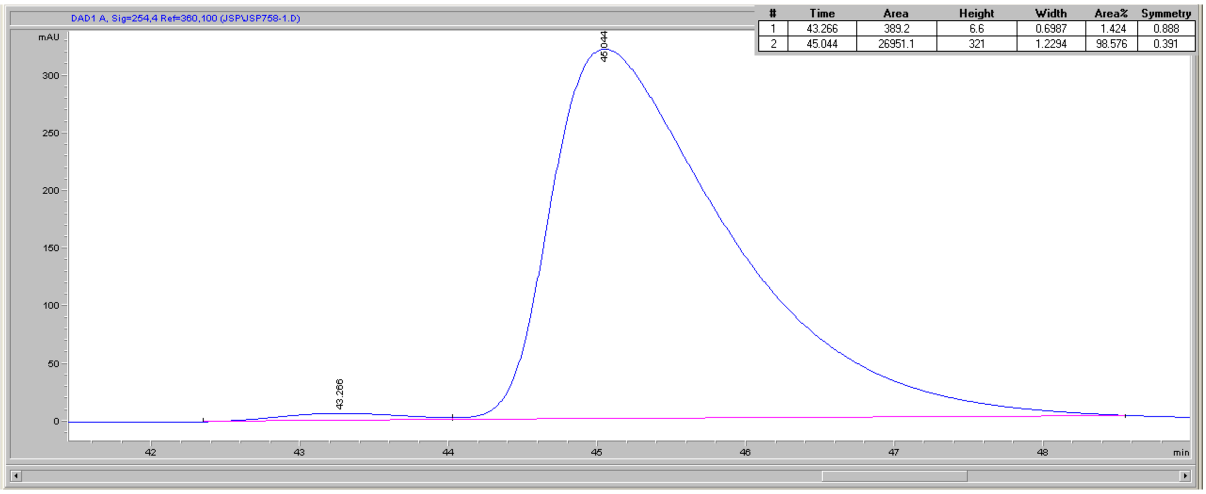

(1*R*,3*s*,5*S*)-*N*-((*R*)-4-(4-chlorophenyl)buta-2,3-dien-1-yl)-6-oxabicyclo[3.1.0]hexane-3-carboxamide (4e):

HPLC conditions: ChiralART SC, 90:10 hexane/isopropanol, 1.0 mL/min flow rate, T = 25 °C, λ_{max} = 254 nm; t_R (min) = 31.3 (minor), 36.1 (major).

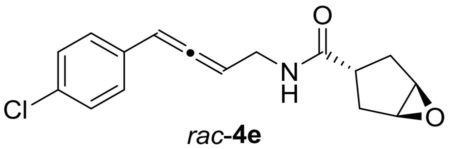

rac-**4e**

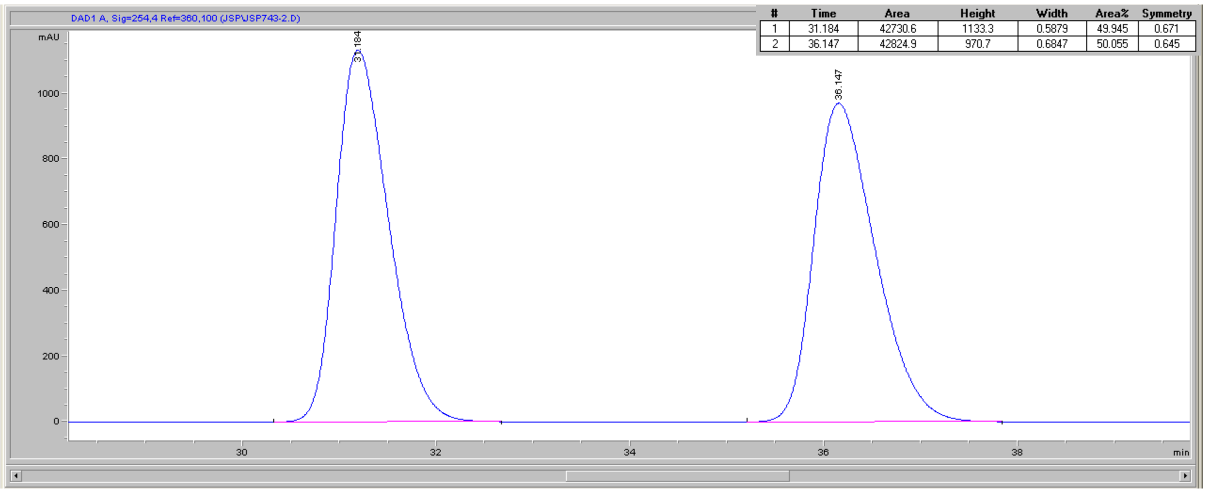

#	Time	Area	Height	Width	Area%	Symmetry
1	31.184	42730.6	1133.3	0.5879	49.945	0.671
2	36.147	42824.9	970.7	0.6847	50.055	0.645

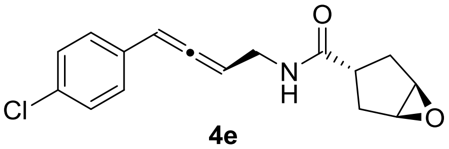

4e

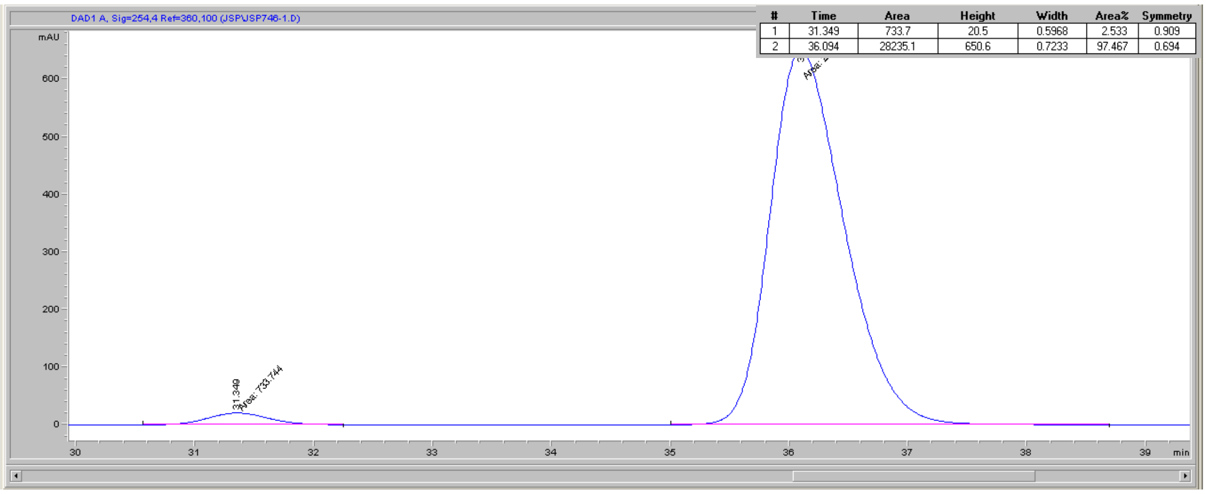

#	Time	Area	Height	Width	Area%	Symmetry
1	31.349	733.7	20.5	0.5968	2.533	0.909
2	36.094	28235.1	650.6	0.7233	97.467	0.694

(*R*)-*N*-(4-(4-chlorophenyl)buta-2,3-dien-1-yl)thiophene-2-carboxamide (4f):

HPLC conditions: ChiralART SC, 90:10 hexane/isopropanol, 1.0 mL/min flow rate, T = 25 °C, λ_{max} = 254 nm; t_R (min) = 30.6 (minor), 35.0 (major).

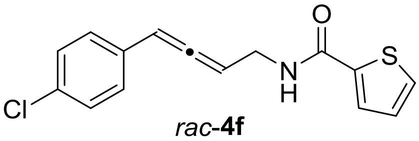

rac-**4f**

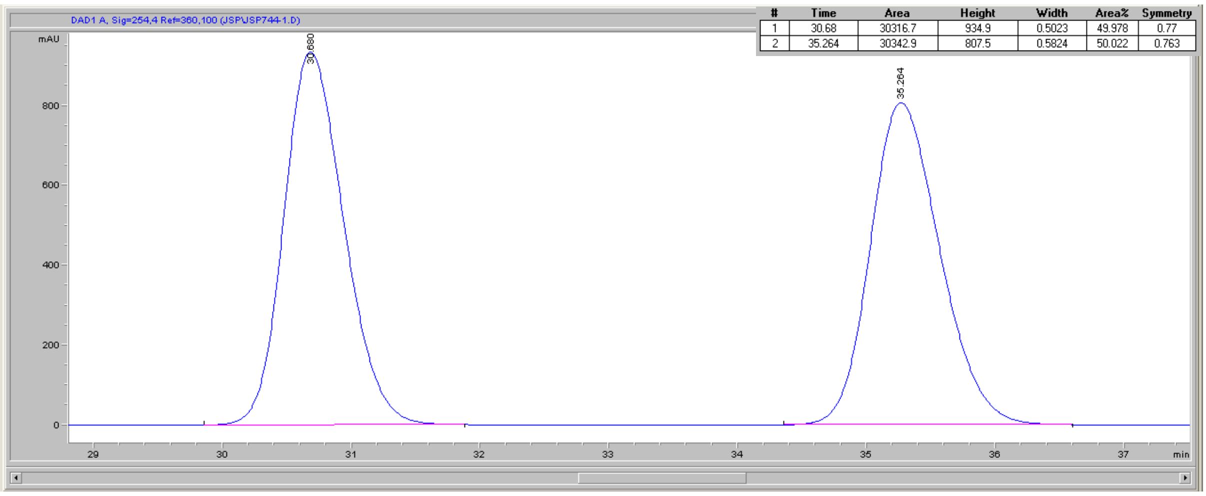

#	Time	Area	Height	Width	Area%	Symmetry
1	30.68	30316.7	934.9	0.5023	49.978	0.77
2	35.264	30342.9	807.5	0.5824	50.022	0.763

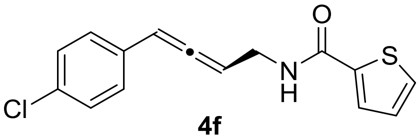

4f

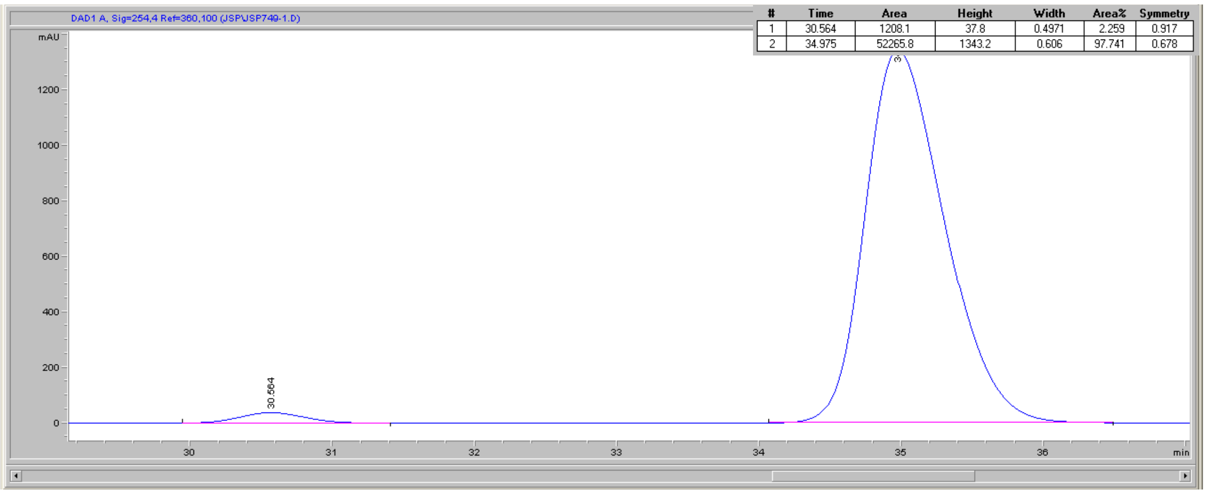

#	Time	Area	Height	Width	Area%	Symmetry
1	30.564	1208.1	37.8	0.4971	2.259	0.917
2	34.975	52265.8	1343.2	0.606	97.741	0.678

***tert*-butyl (*R*)-(4-(4-chlorophenyl)buta-2,3-dien-1-yl)carbamate (4g):**

HPLC conditions: Chiralpak OD-H, 95:5 hexane/isopropanol, 1.0 mL/min flow rate, T = 25 °C, λ_{max} = 254 nm; t_R (min) = 7.7 (minor), 8.6 (major).

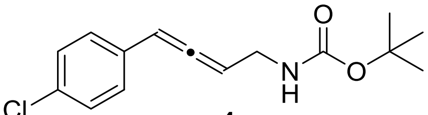

rac-**4g**

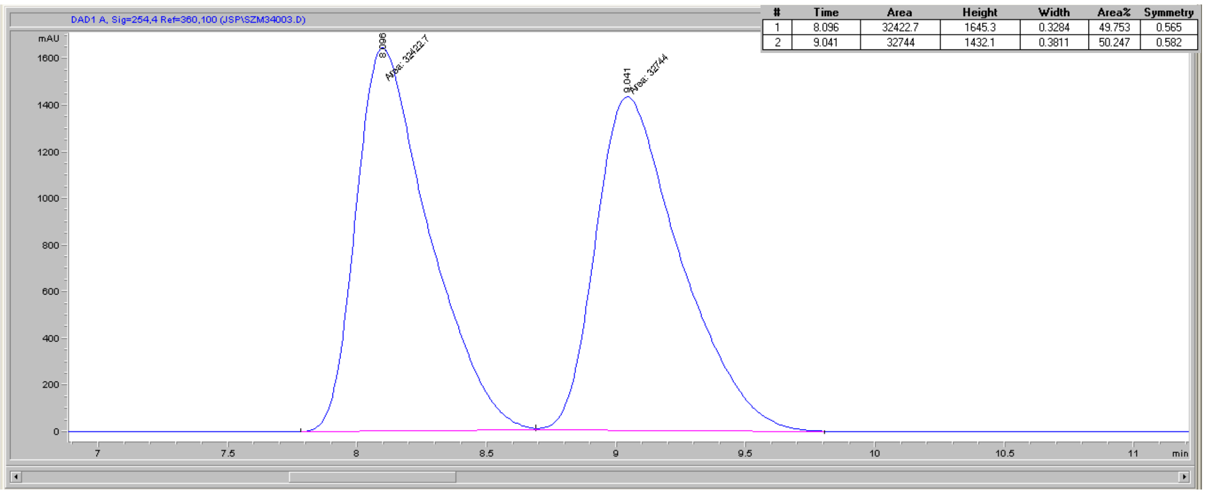

#	Time	Area	Height	Width	Area%	Symmetry
1	8.096	32422.7	1645.3	0.3284	49.753	0.565
2	9.041	32744	1432.1	0.3811	50.247	0.582

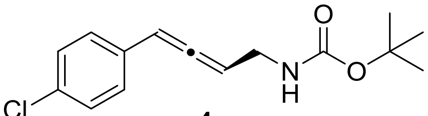

4g

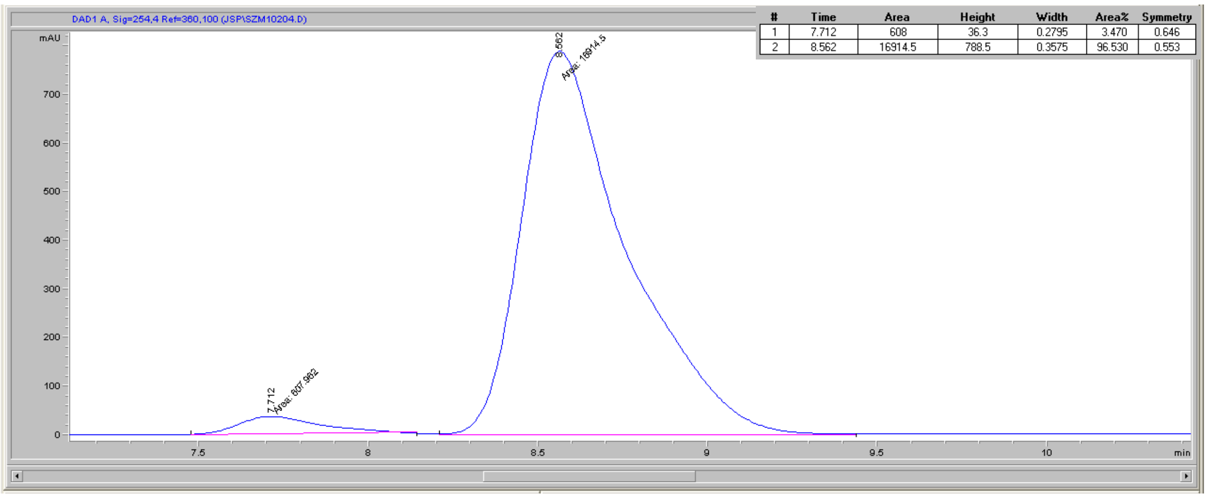

#	Time	Area	Height	Width	Area%	Symmetry
1	7.712	608	36.3	0.2795	3.470	0.646
2	8.562	16914.5	788.5	0.3575	96.530	0.553

(*R*)-*N*-(4-(4-bromophenyl)buta-2,3-dien-1-yl)-1-(4-chlorophenyl)cyclopentane-1-carboxamide (4h):

HPLC conditions: ChiralART SC, 95:5 hexane/isopropanol, 1.0 mL/min flow rate, T = 25 °C, λ_{max} = 254 nm; t_R (min) = 19.1 (minor), 19.9 (major).

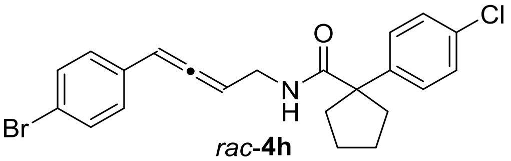

rac-**4h**

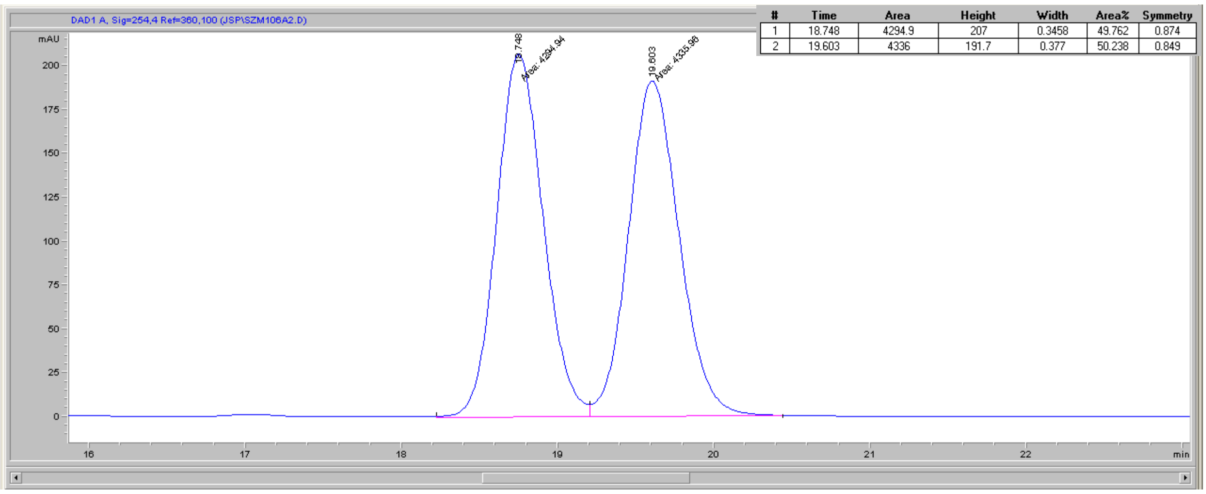

#	Time	Area	Height	Width	Area%	Symmetry
1	18.748	4294.9	207	0.3458	49.762	0.874
2	19.603	4336	191.7	0.377	50.238	0.849

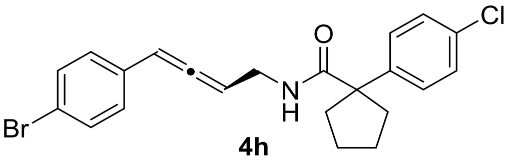

4h

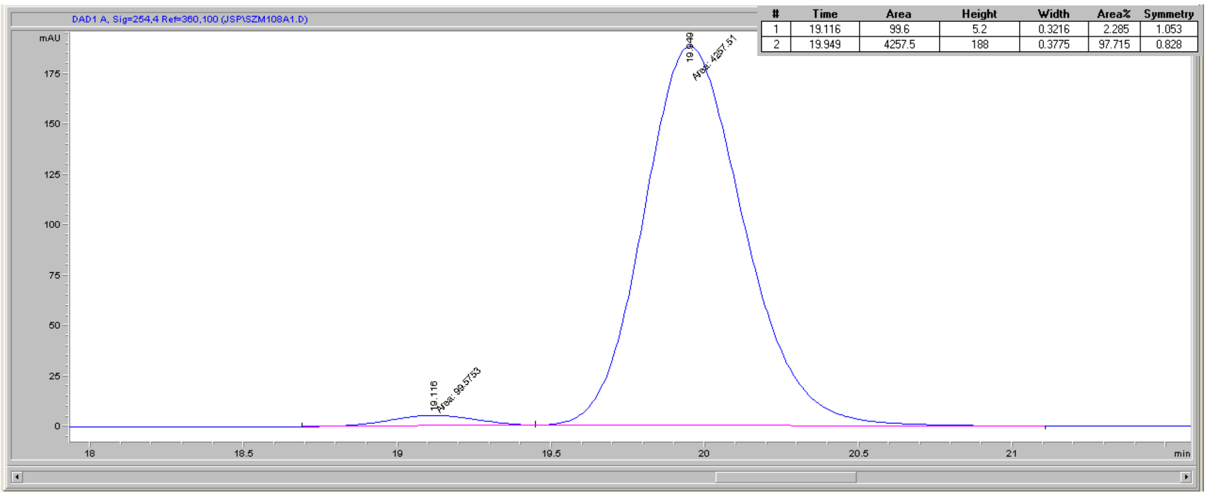

#	Time	Area	Height	Width	Area%	Symmetry
1	19.116	99.6	5.2	0.3216	2.285	1.053
2	19.949	4257.5	188	0.3775	97.715	0.828

(*R*)-*N*-(4-(4-bromophenyl)buta-2,3-dien-1-yl)-4-hydroxybutanamide (4i):

HPLC conditions: ChiralART SA, 93:7 hexane/isopropanol, 1.0 mL/min flow rate, T = 25 °C, λ_{max} = 254 nm; t_R (min) = 16.7 (minor), 18.2 (major).

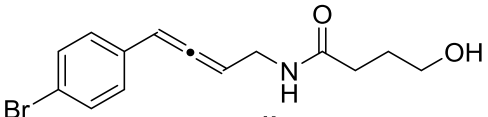

rac-**4i**

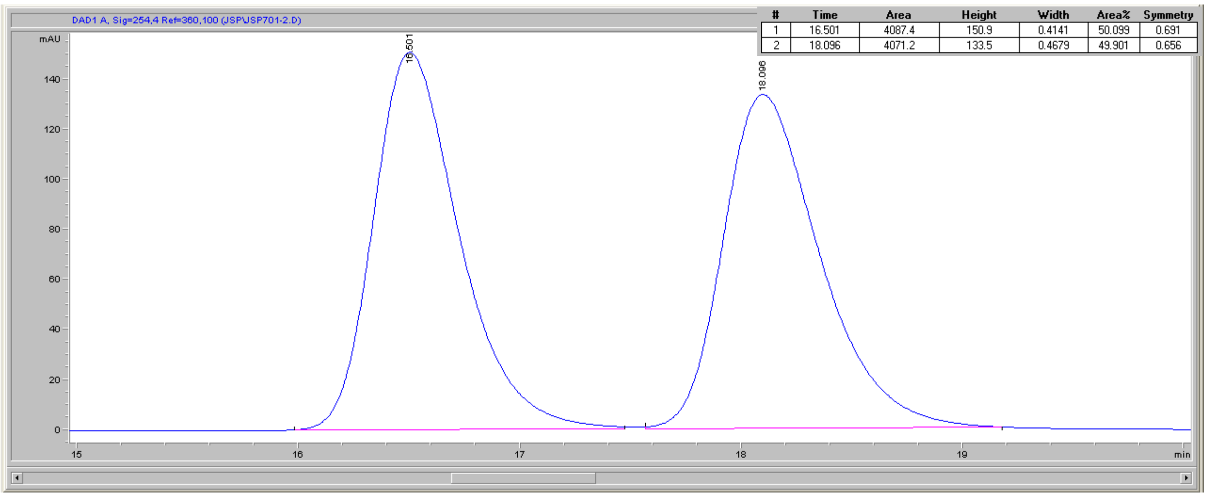

#	Time	Area	Height	Width	Area%	Symmetry
1	16.501	4087.4	150.9	0.4141	50.099	0.691
2	18.096	4071.2	133.5	0.4679	49.901	0.656

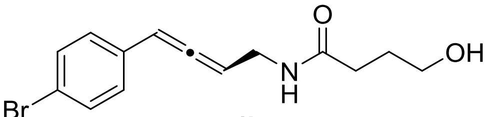

4i

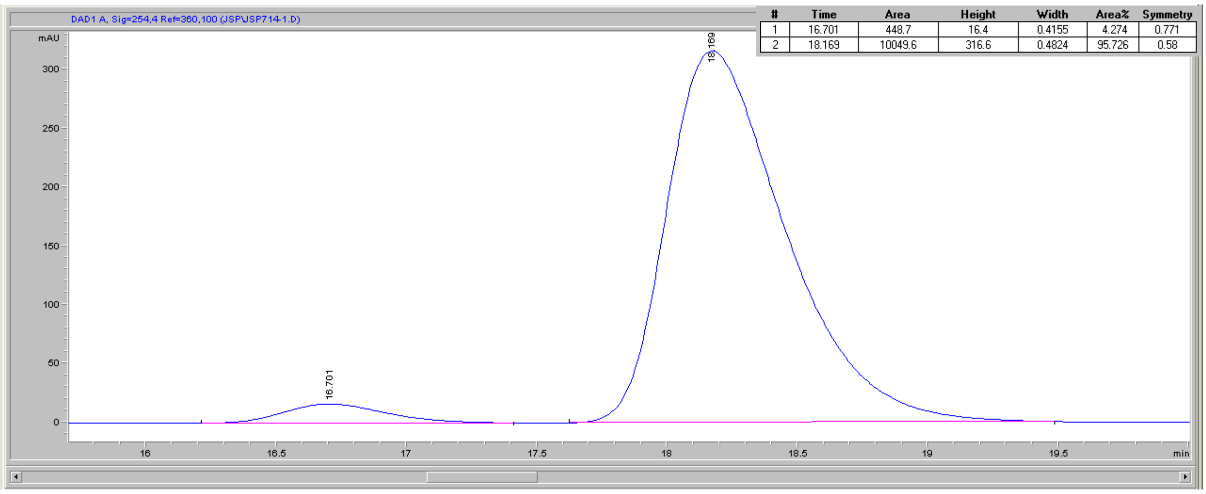

#	Time	Area	Height	Width	Area%	Symmetry
1	16.701	448.7	16.4	0.4155	4.274	0.771
2	18.169	10049.6	316.6	0.4824	95.726	0.58

(*R*)-5-bromo-*N*-(4-(4-bromophenyl)buta-2,3-dien-1-yl)furan-2-carboxamide (4j):

HPLC conditions: ChiralART SC, 90:10 hexane/isopropanol, 1.0 mL/min flow rate, T = 25 °C, λ_{max} = 254 nm; t_R (min) = 19.8 (minor), 21.8 (major).

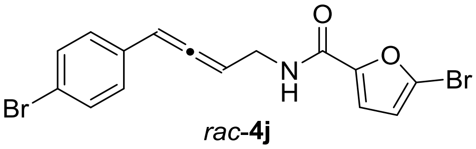

rac-**4j**

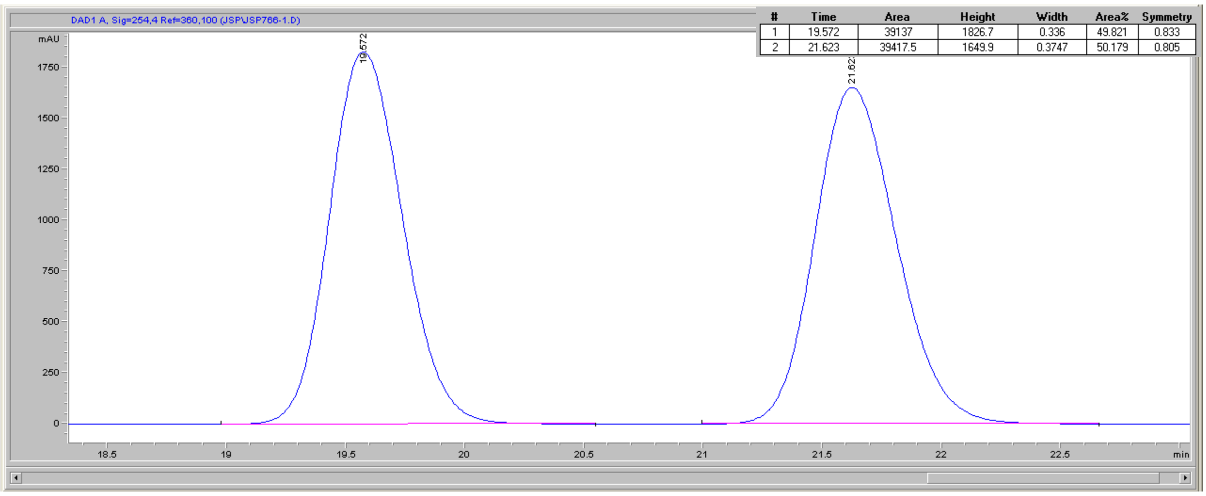

#	Time	Area	Height	Width	Area%	Symmetry
1	19.572	39137	1826.7	0.336	49.821	0.833
2	21.623	39417.5	1649.9	0.3747	50.179	0.805

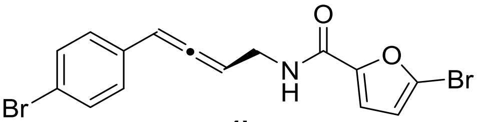

4j

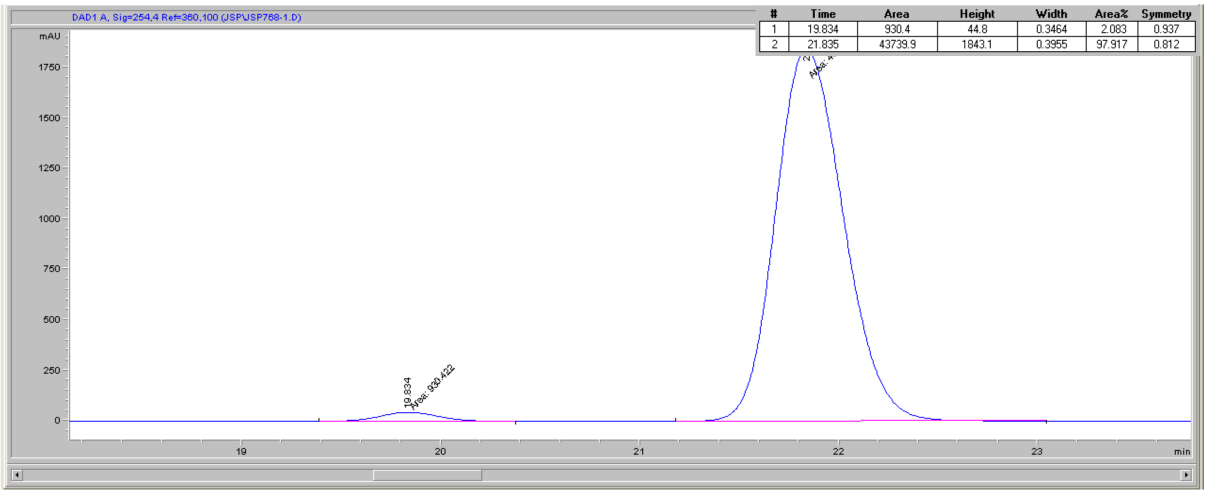

#	Time	Area	Height	Width	Area%	Symmetry
1	19.834	930.4	44.8	0.3464	2.083	0.937
2	21.835	43739.9	1843.1	0.3955	97.917	0.812

(*R*)-*N*-(4-(4-bromophenyl)buta-2,3-dien-1-yl)cyclopropanecarboxamide (4k):

HPLC conditions: ChiralART SA, 95:5 hexane/isopropanol, 1.0 mL/min flow rate, T = 25 °C, λ_{max} = 254 nm; t_R (min) = 20.1 (major), 28.3 (minor).

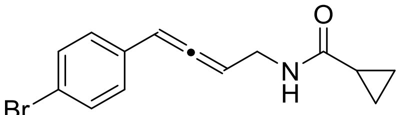

rac-**4k**

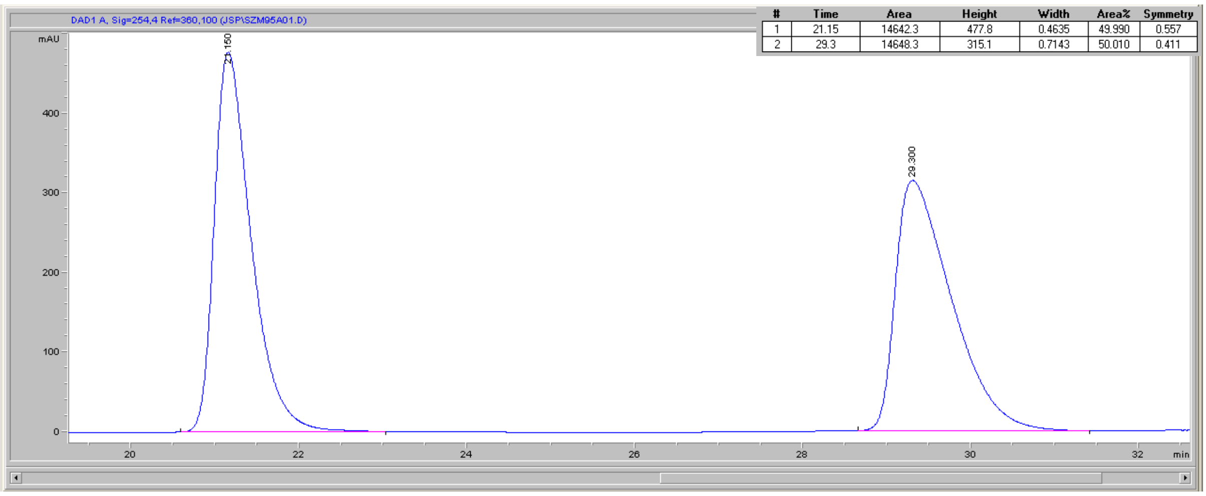

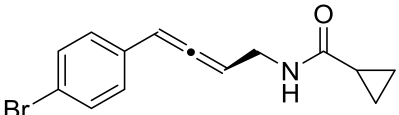

4k

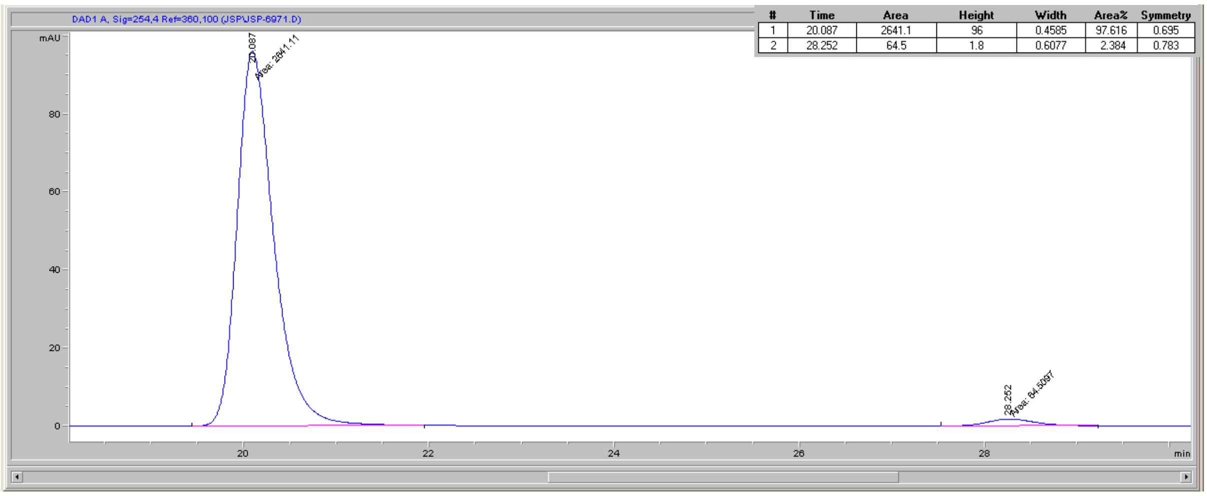

Methyl (*R*)-4-((4-(4-bromophenyl)buta-2,3-dien-1-yl)amino)-4-oxobutanoate (4l):

HPLC conditions: ChiralART SA, 90:10 hexane/isopropanol, 1.0 mL/min flow rate, T = 25 °C, λ_{max} = 254 nm; t_R (min) = 23.1 (minor), 26.3 (major).

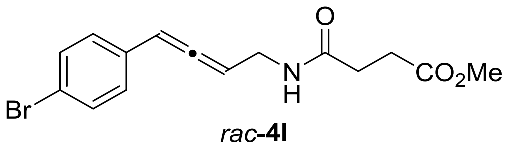

rac-**4l**

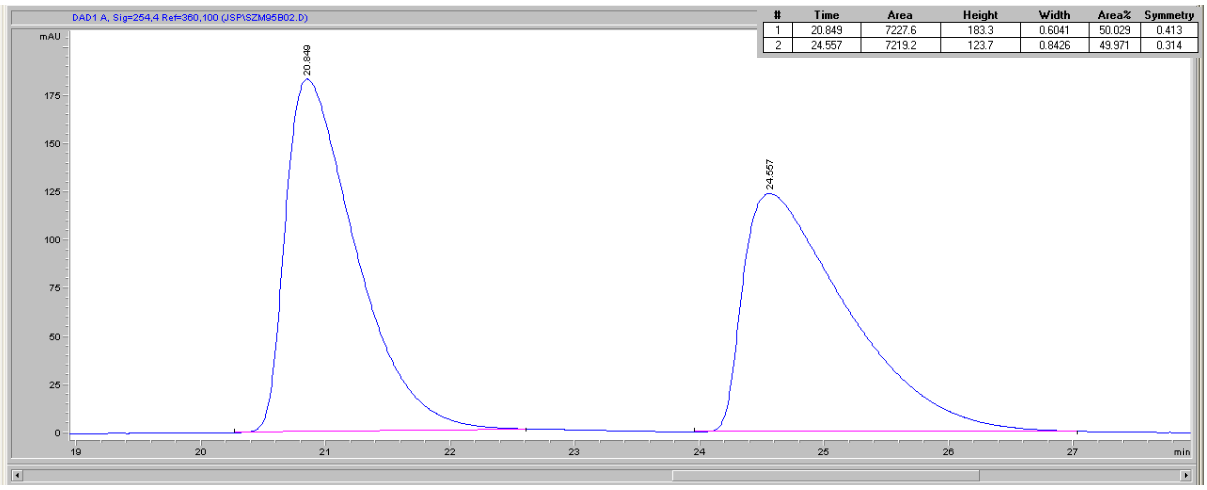

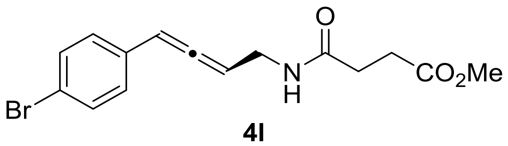

4l

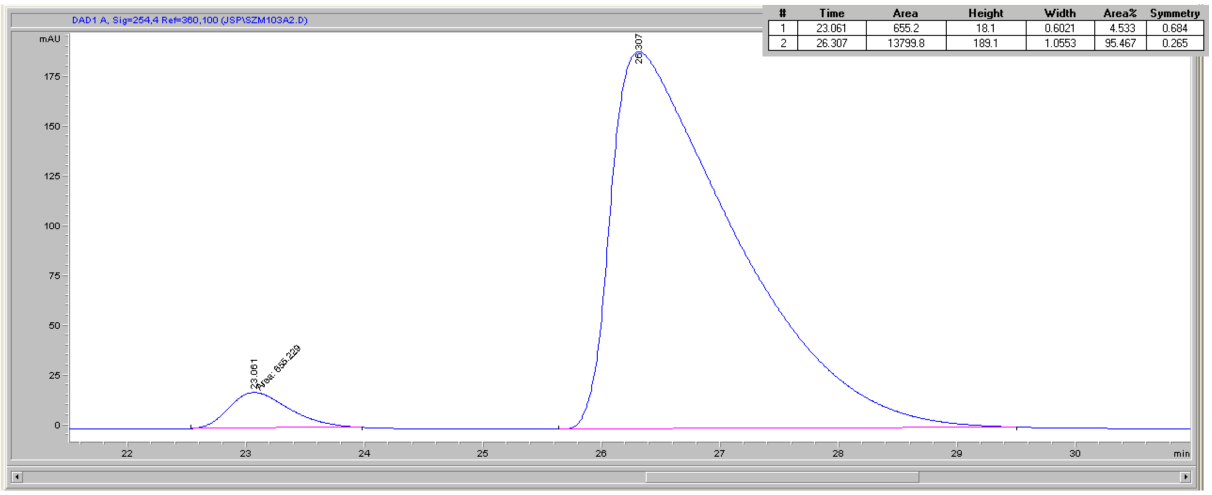

(*R*)-*N*-(4-(4-chlorophenyl)buta-2,3-dien-1-yl)acetamide (4m):

HPLC conditions: ChiralART SA, 97:3 hexane/isopropanol, 1.0 mL/min flow rate, T = 25 °C, λ_{max} = 254 nm; t_R (min) = 50.9 (minor), 54.1 (major).

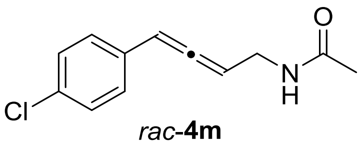

rac-**4m**

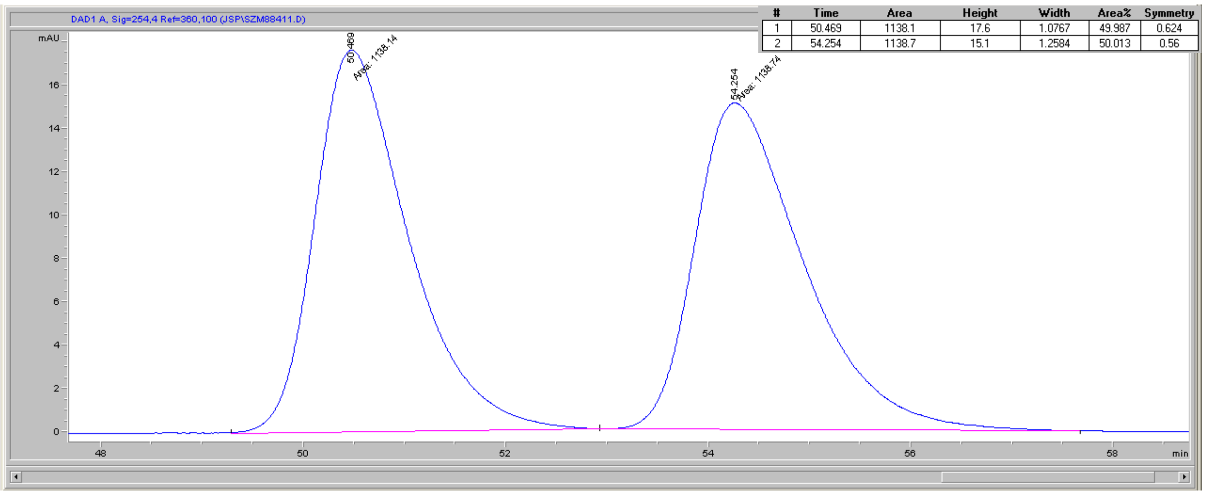

#	Time	Area	Height	Width	Area%	Symmetry
1	50.469	1138.1	17.6	1.0767	49.987	0.624
2	54.254	1138.7	15.1	1.2584	50.013	0.56

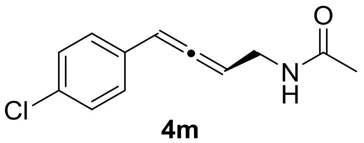

4m

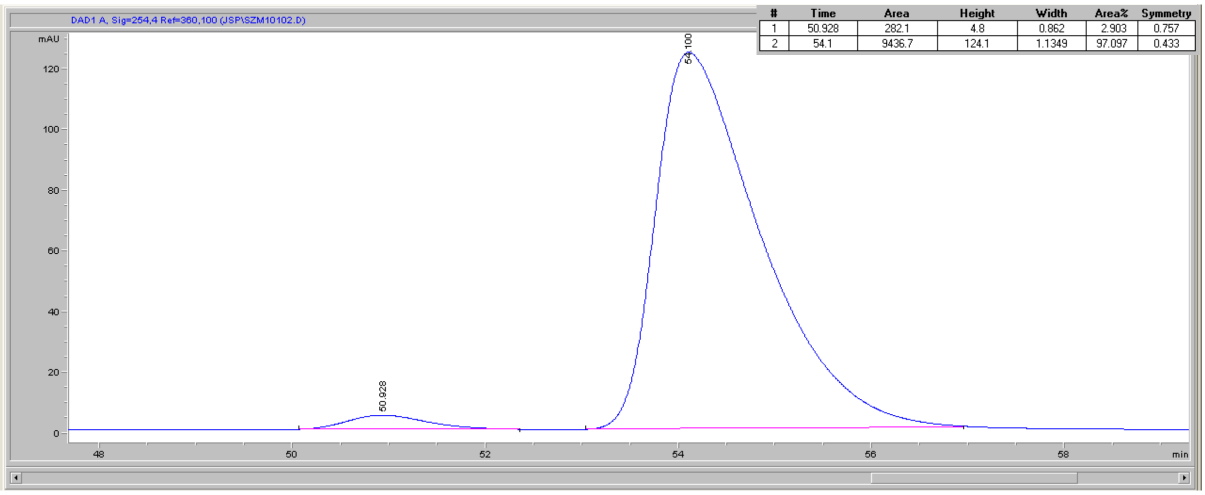

#	Time	Area	Height	Width	Area%	Symmetry
1	50.928	282.1	4.8	0.862	2.903	0.757
2	54.1	9436.7	124.1	1.1349	97.097	0.433

(*R*)-*N*-(4-(4-bromophenyl)buta-2,3-dien-1-yl)acetamide (4n):

HPLC conditions: ChiralART SA, 95:5 hexane/isopropanol, 1.0 mL/min flow rate, T = 25 °C, λ_{max} = 254 nm; t_R (min) = 24.2 (minor), 25.5 (major).

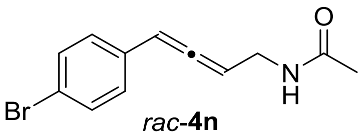

rac-**4n**

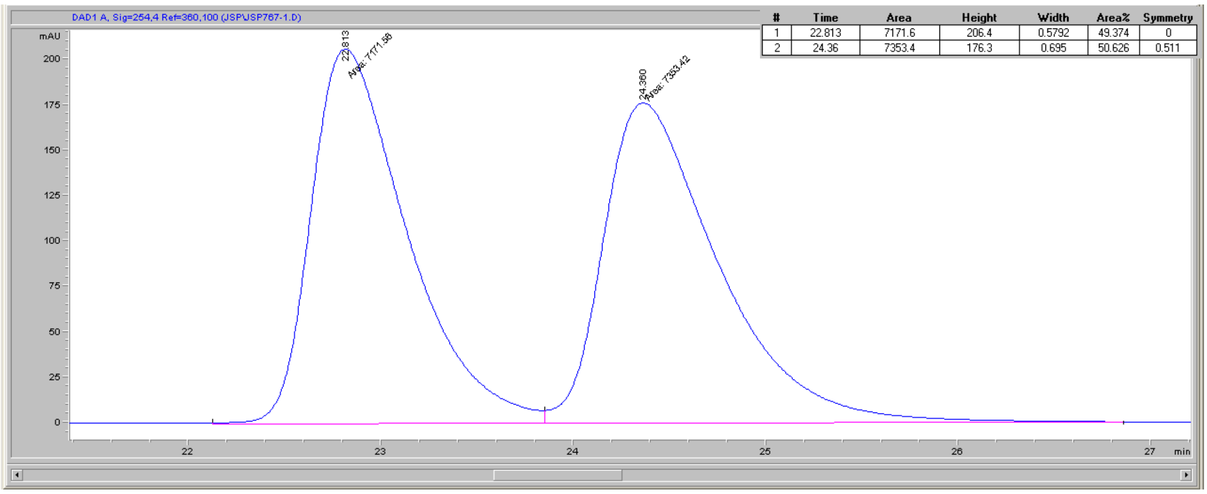

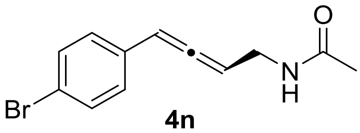

4n

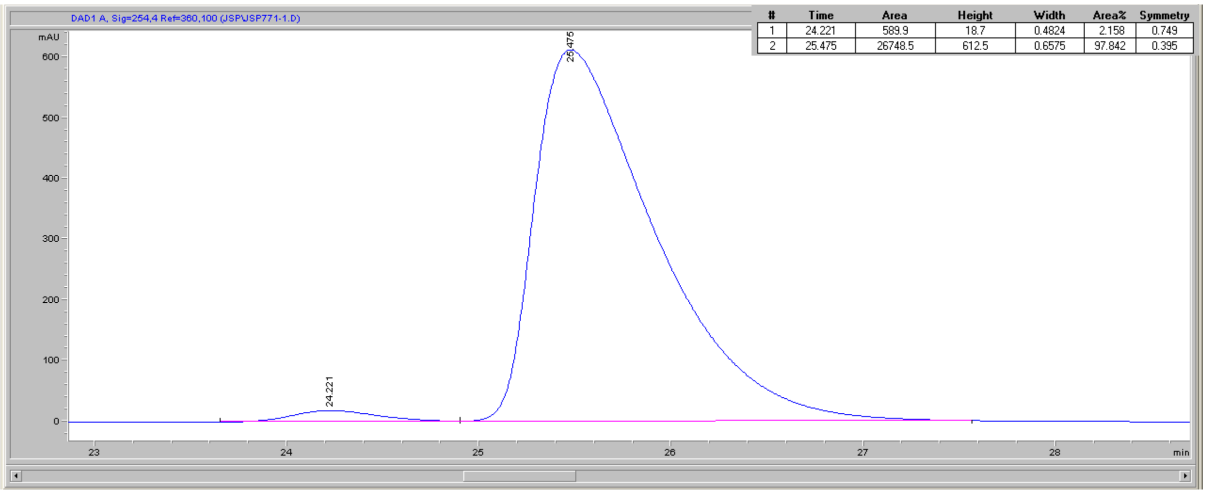

(*R*)-*N*-(4-(4-fluorophenyl)buta-2,3-dien-1-yl)acetamide (4o):

HPLC conditions: ChiralART SA, 95:5 hexane/isopropanol, 1.0 mL/min flow rate, T = 25 °C, λ_{max} = 254 nm; t_R (min) = 22.0 (minor), 22.9 (major).

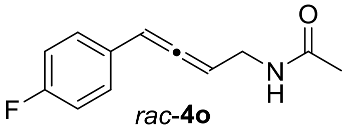

rac-**4o**

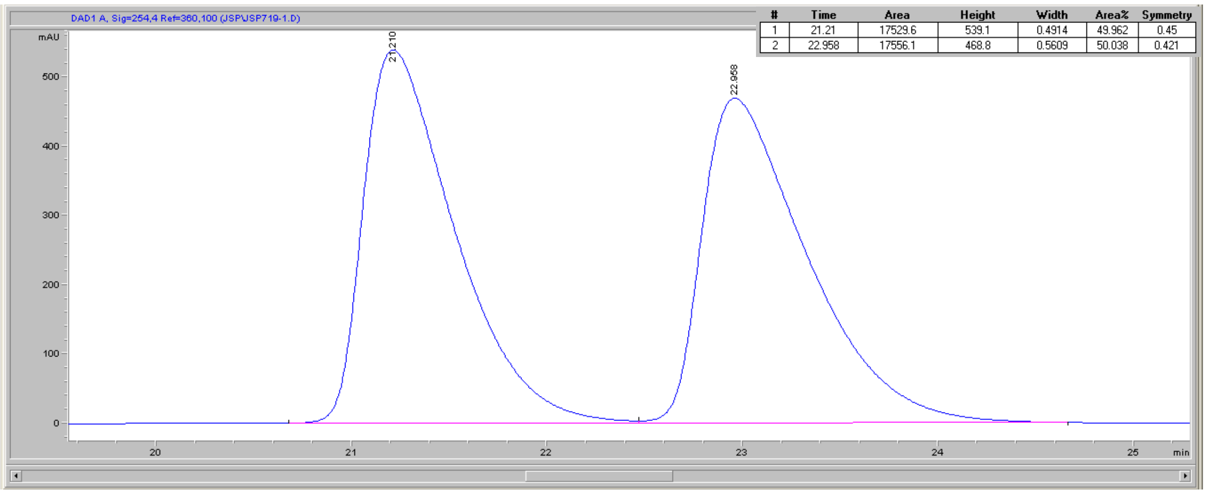

#	Time	Area	Height	Width	Area%	Symmetry
1	21.21	17529.6	539.1	0.4914	49.962	0.45
2	22.958	17556.1	468.8	0.5609	50.038	0.421

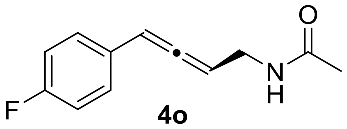

4o

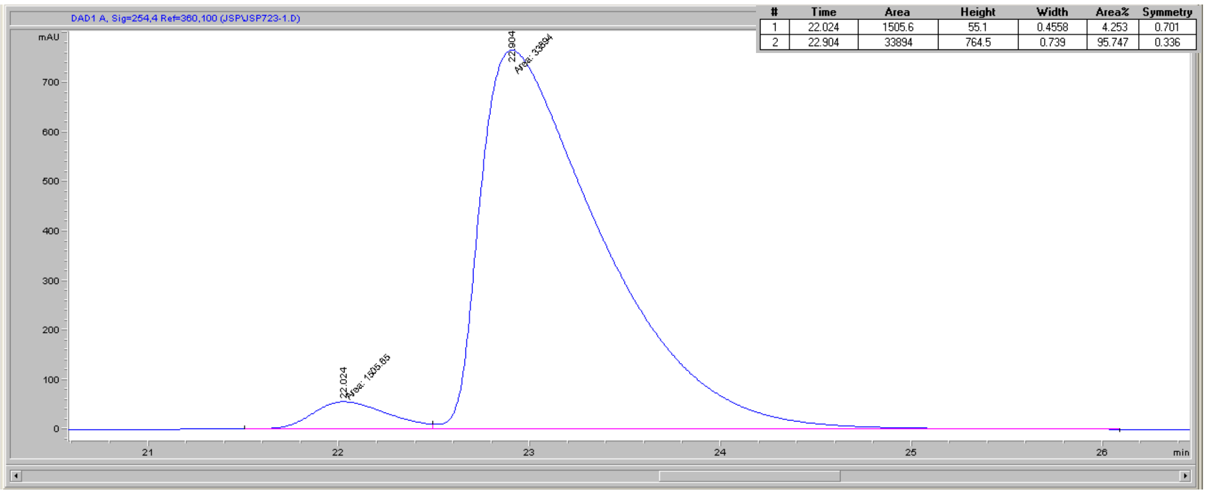

#	Time	Area	Height	Width	Area%	Symmetry
1	22.024	1505.6	55.1	0.4558	4.253	0.701
2	22.904	33894	764.5	0.739	95.747	0.336

(*R*)-*N*-(4-(3-methoxyphenyl)buta-2,3-dien-1-yl)acetamide (4p):

HPLC conditions: ChiralART SC, 85:15 hexane/isopropanol, 1.0 mL/min flow rate, T = 25 °C, λ_{max} = 254 nm; t_R (min) = 16.3 (minor), 21.1 (major).

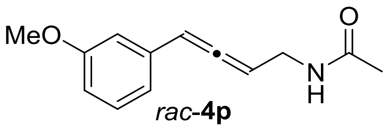

rac-**4p**

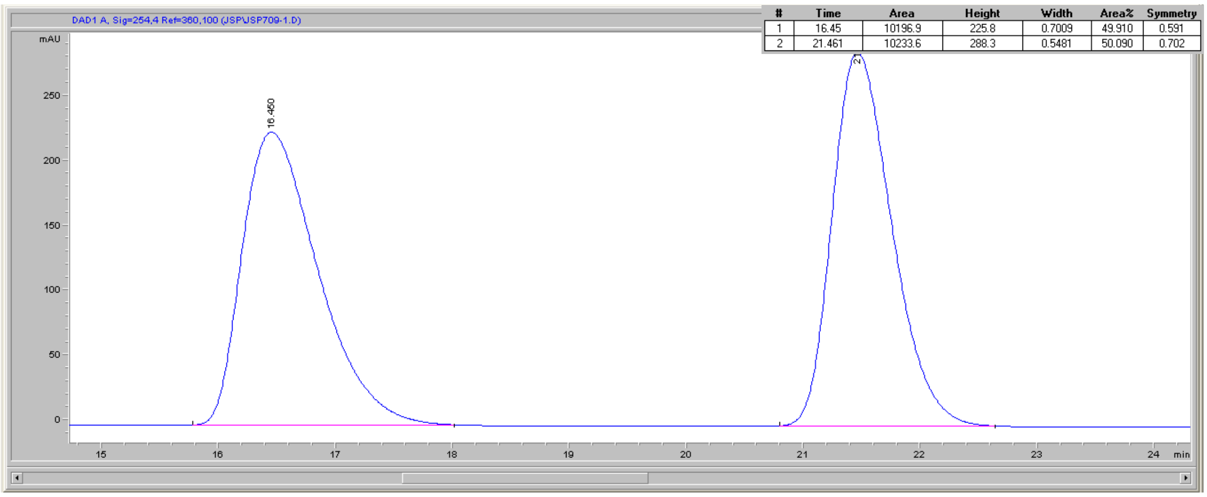

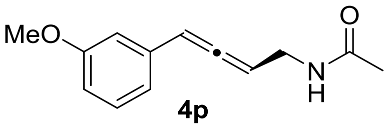

4p

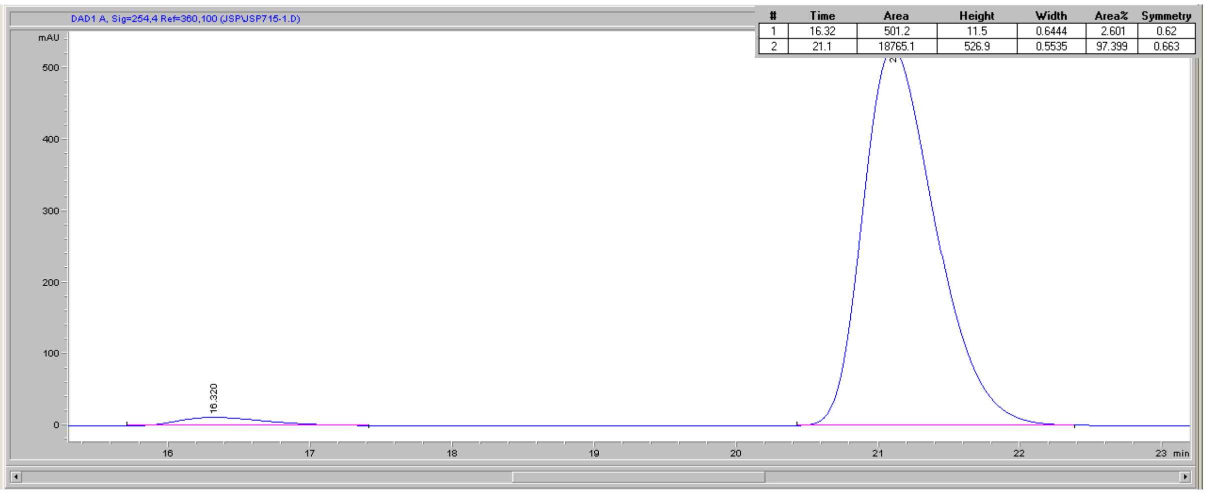

(*R*)-*N*-(4-(3-cyanophenyl)buta-2,3-dien-1-yl)acetamide (4q):

HPLC conditions: ChiralART SA, 93:7 hexane/isopropanol, 1.0 mL/min flow rate, T = 25 °C, λ_{max} = 254 nm; t_R (min) = 30.5 (minor), 32.8 (major).

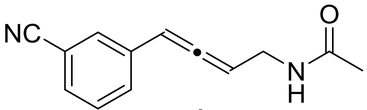

rac-**4q**

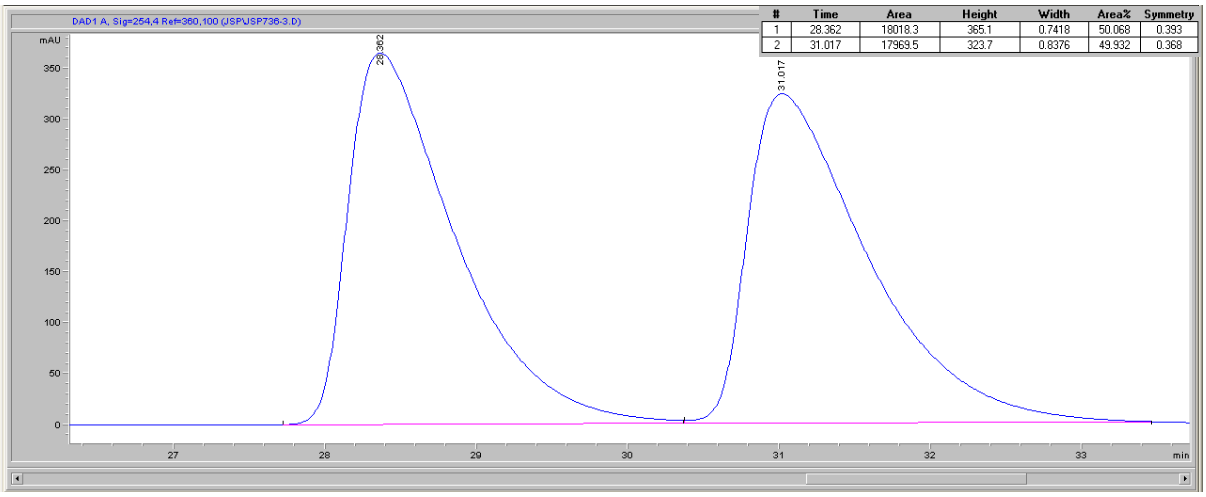

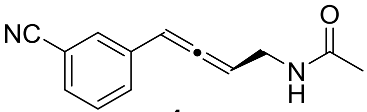

4q

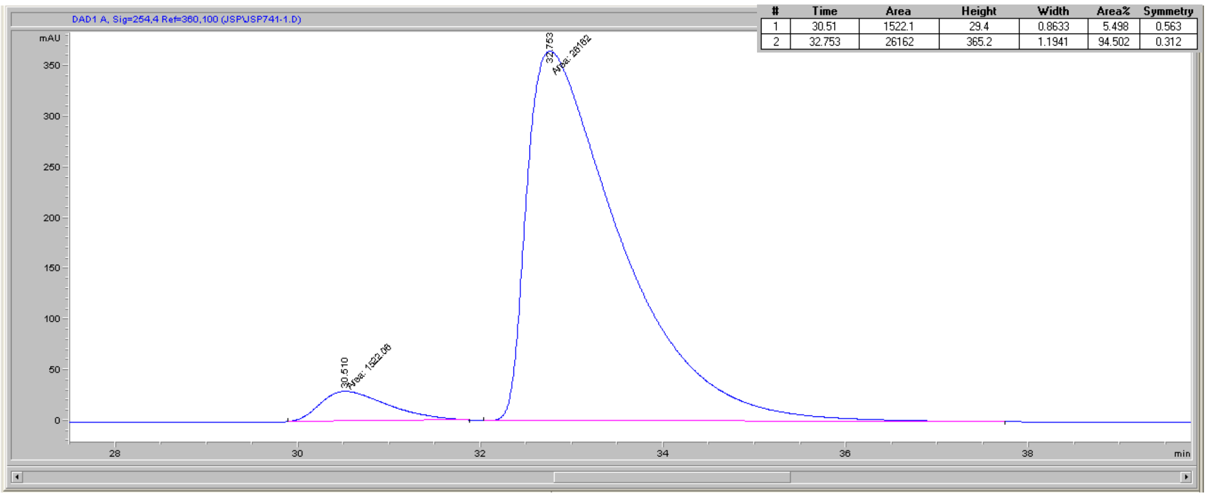

(*R*)-*N*-(4-(4-iodophenyl)buta-2,3-dien-1-yl)acetamide (4r):

HPLC conditions: ChiralART SA, 97:3 hexane/isopropanol, 1.0 mL/min flow rate, T = 25 °C, λ_{max} = 254 nm; t_R (min) = 58.0 (minor), 62.4 (major).

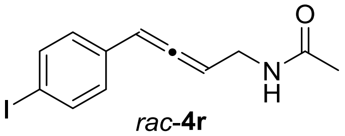

rac-**4r**

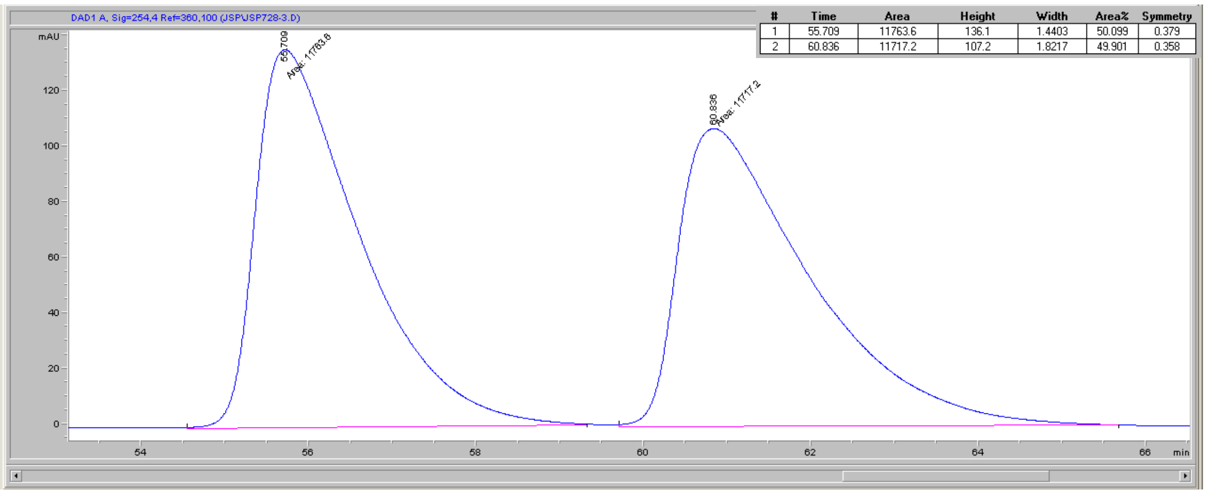

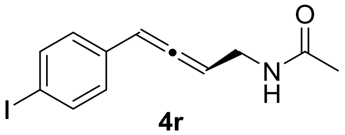

4r

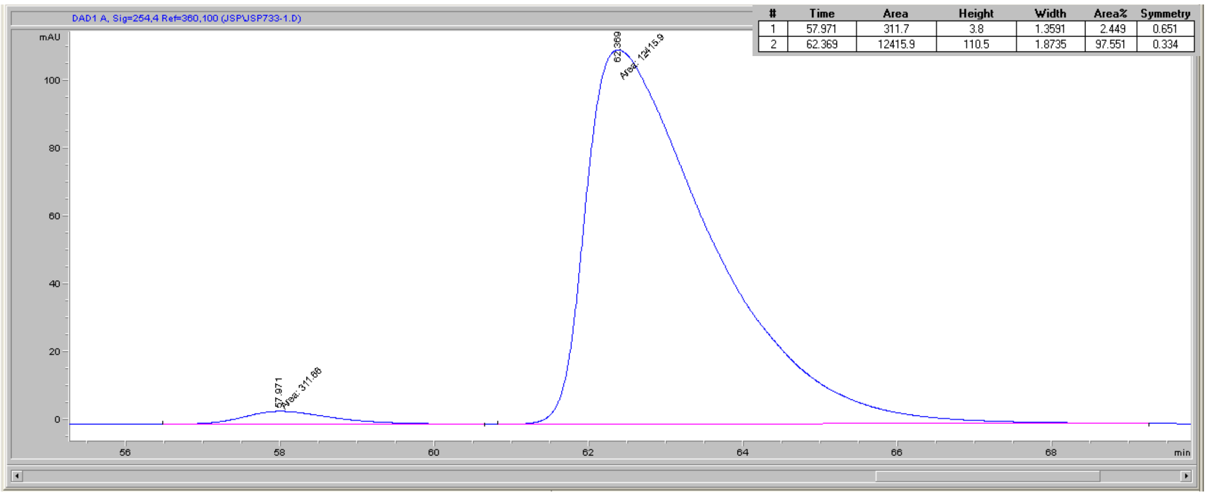

(*R*)-*N*-(4-(4-bromophenyl)buta-2,3-dien-1-yl)pent-4-enamide (4s):

HPLC conditions: ChiralART SC, 95:5 hexane/isopropanol, 1.0 mL/min flow rate, T = 25 °C, λ_{max} = 254 nm; t_R (min) = 32.1 (minor), 33.1 (major).

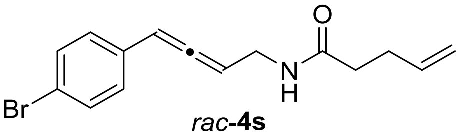

rac-**4s**

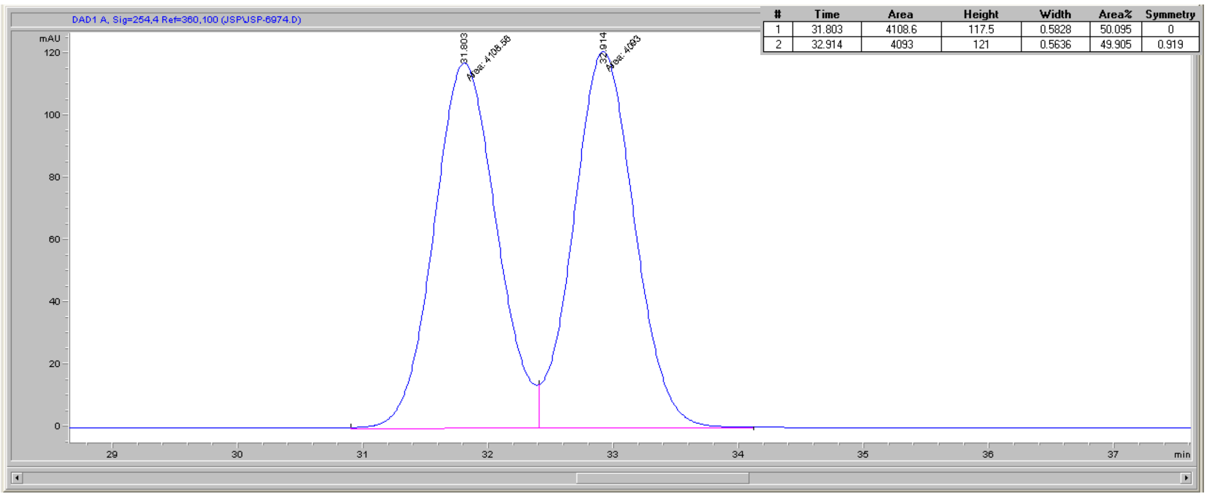

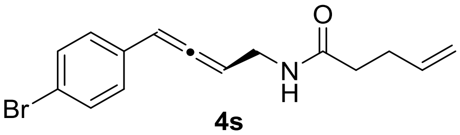

4s

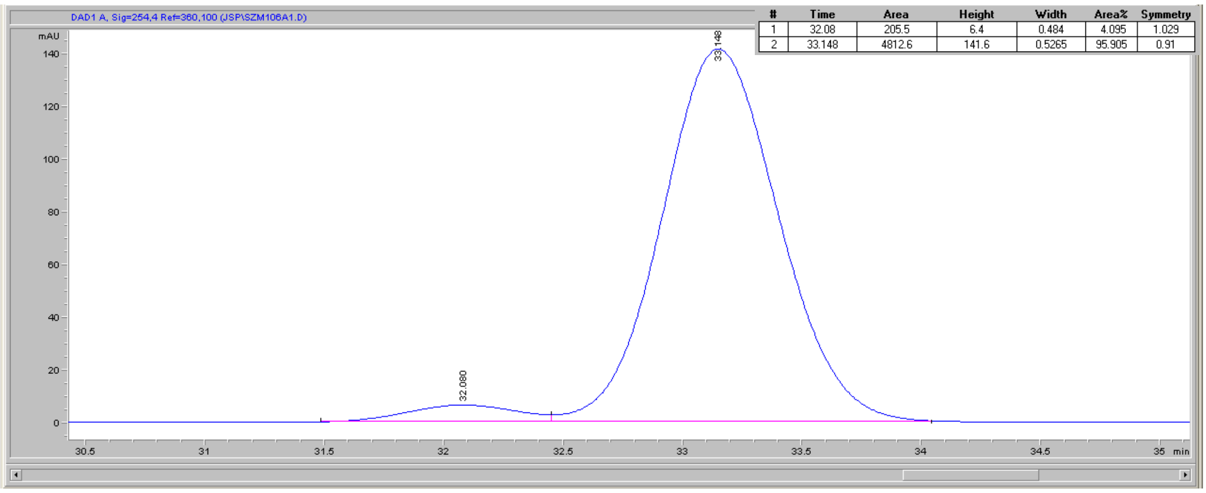

(*R*)-*N*-(4-(4-fluorophenyl)buta-2,3-dien-1-yl)pent-4-enamide (4t):

HPLC conditions: ChiralART SA, 97:3 hexane/isopropanol, 1.0 mL/min flow rate, T = 25 °C, λ_{max} = 254 nm; t_R (min) = 35.4 (major), 38.0 (minor).

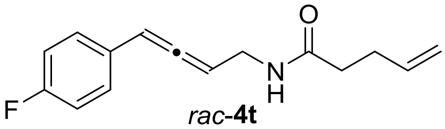

rac-**4t**

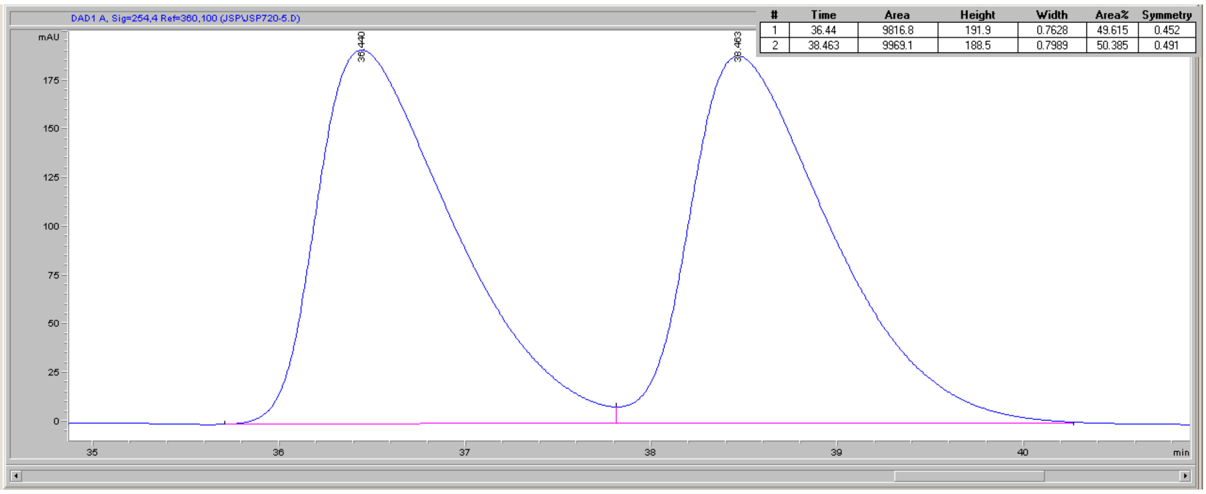

#	Time	Area	Height	Width	Area%	Symmetry
1	36.44	9816.8	191.9	0.7628	49.615	0.452
2	38.463	9969.1	188.5	0.7989	50.385	0.491

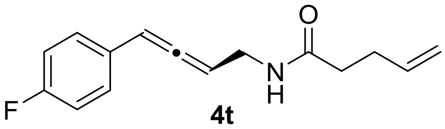

4t

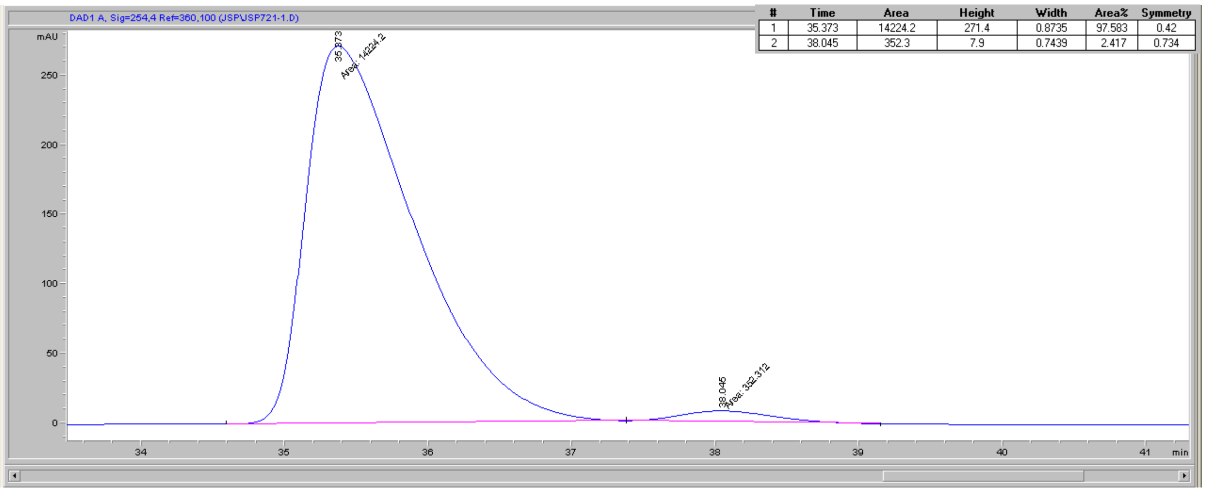

#	Time	Area	Height	Width	Area%	Symmetry
1	35.373	14224.2	271.4	0.8735	97.583	0.42
2	38.045	352.3	7.9	0.7439	2.417	0.734

Methyl (*R*)-4-((4-(3-methoxyphenyl)buta-2,3-dien-1-yl)amino)-4-oxobutanoate (4u):

HPLC conditions: ChiralART SC, 80:20 hexane/isopropanol, 1.0 mL/min flow rate, T = 25 °C, λ_{max} = 254 nm; t_R (min) = 36.1 (minor), 40.2 (major).

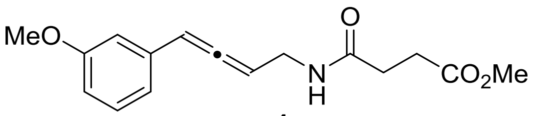

rac-**4u**

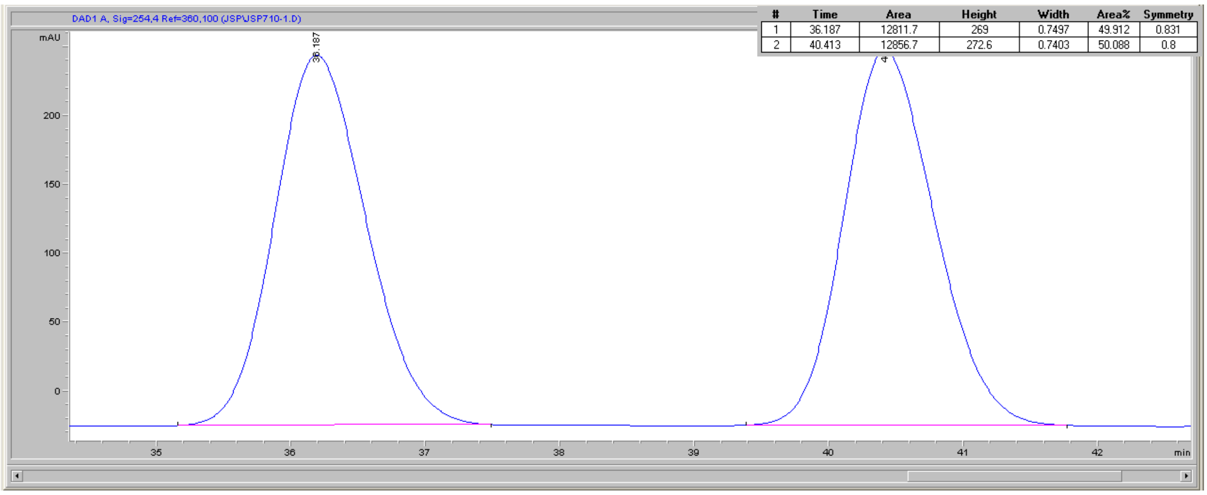

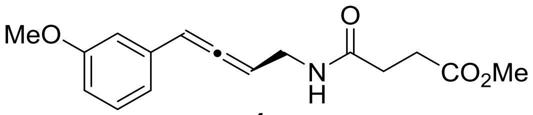

4u

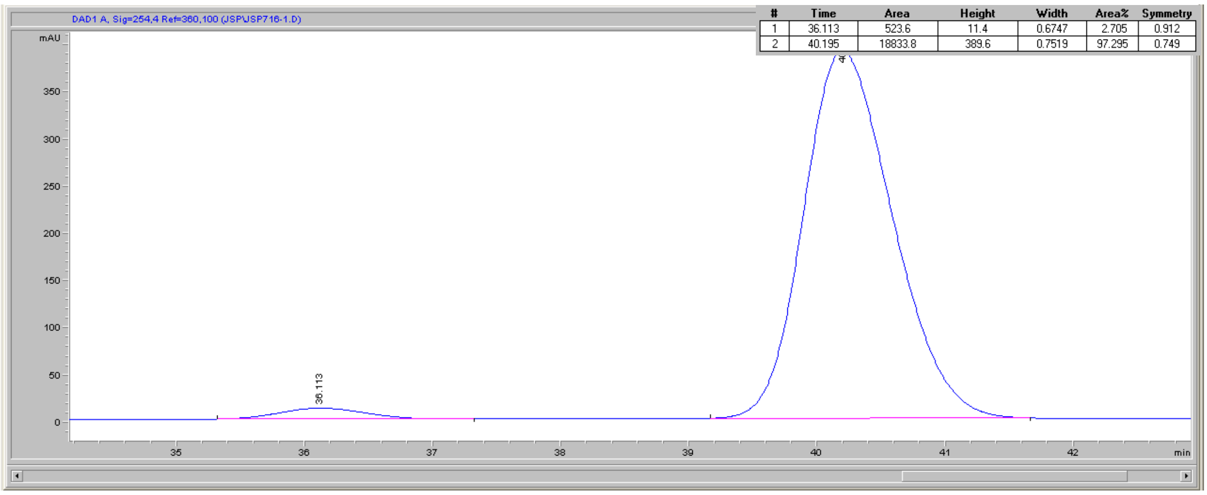

(*R*)-*N*-(4-(3-cyanophenyl)buta-2,3-dien-1-yl)cyclopent-3-ene-1-carboxamide (4v):

HPLC conditions: ChiralART SC, 80:20 hexane/isopropanol, 1.0 mL/min flow rate, T = 25 °C, λ_{max} = 254 nm; t_R (min) = 20.4 (minor), 22.4 (major).

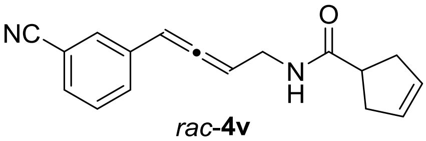

rac-**4v**

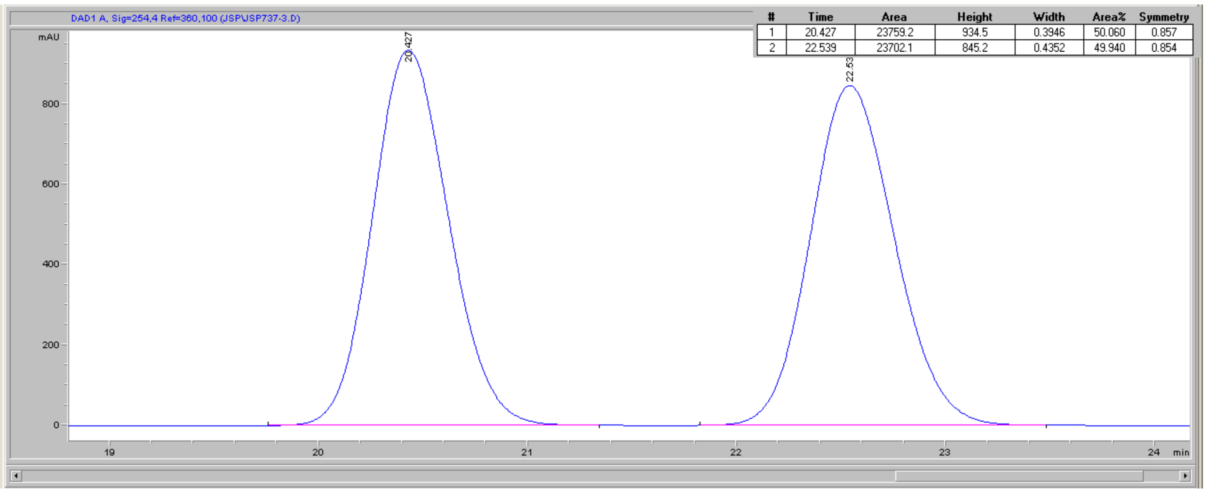

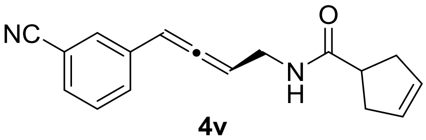

4v

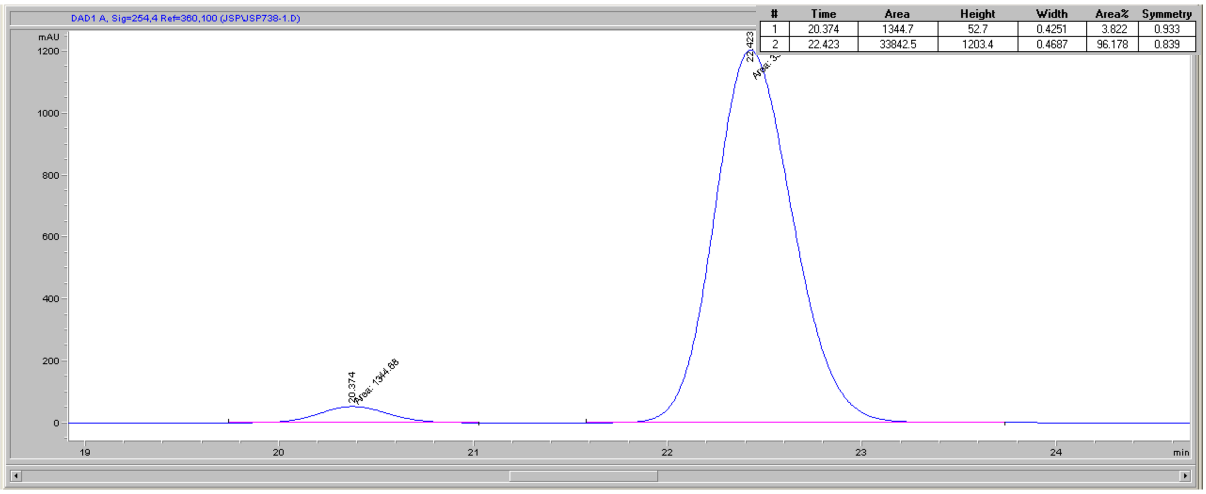

(*R*)-3,3,3-trifluoro-*N*-(4-(4-iodophenyl)buta-2,3-dien-1-yl)propanamide (4w):

HPLC conditions: ChiralART SC, 95:5 hexane/isopropanol, 1.0 mL/min flow rate, T = 25 °C, λ_{max} = 254 nm; t_R (min) = 16.9 (minor), 18.3 (major).

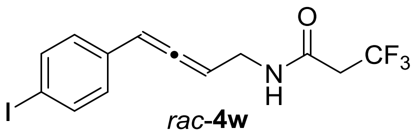

rac-**4w**

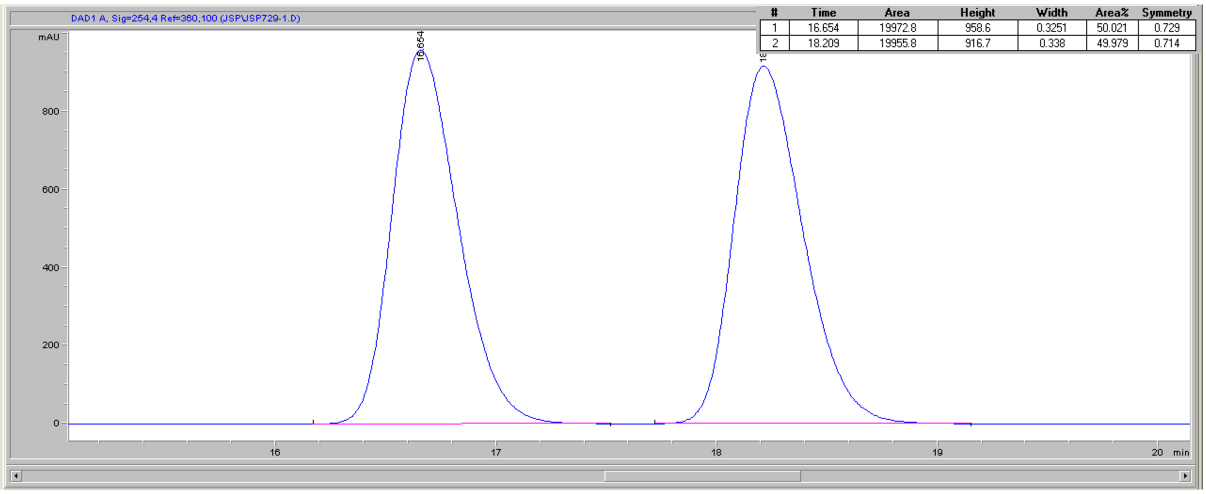

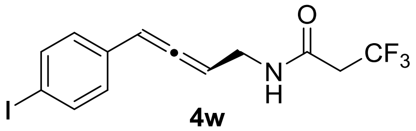

4w

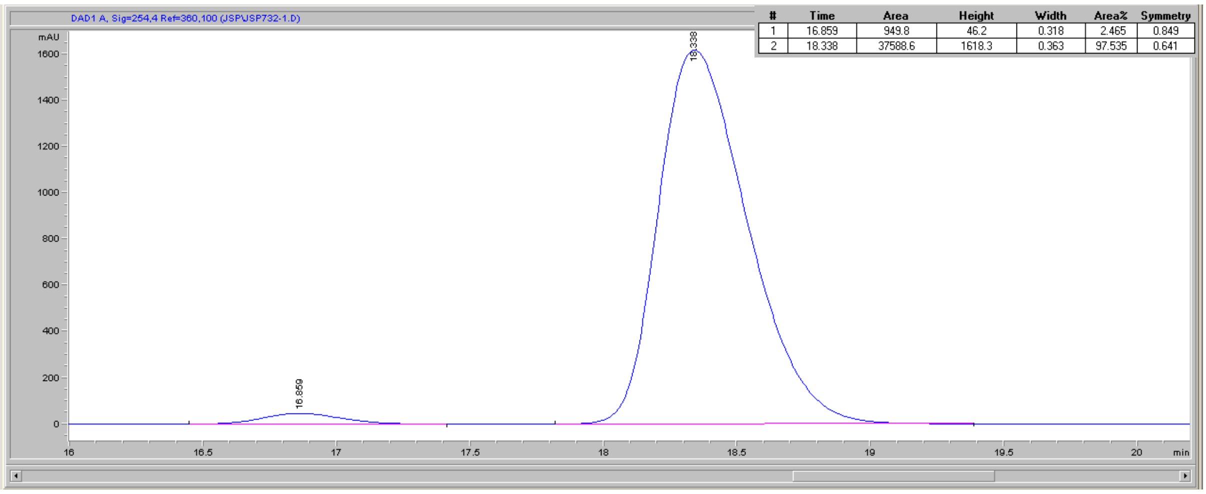

(*S*)-*N*-((*R*)-4-(4-bromophenyl)buta-2,3-dien-1-yl)-2-(4-isobutylphenyl)propanamide (4x):

HPLC conditions: ChiralART SC, 95:5 hexane/isopropanol, 1.0 mL/min flow rate, T = 25 °C, λ_{max} = 254 nm; t_R (min) = 26.8 (minor), 28.6 (major).

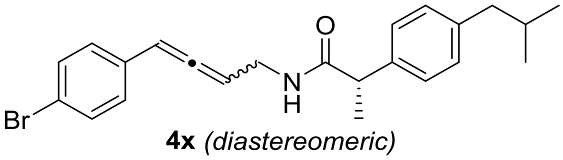

4x *(diastereomeric)*

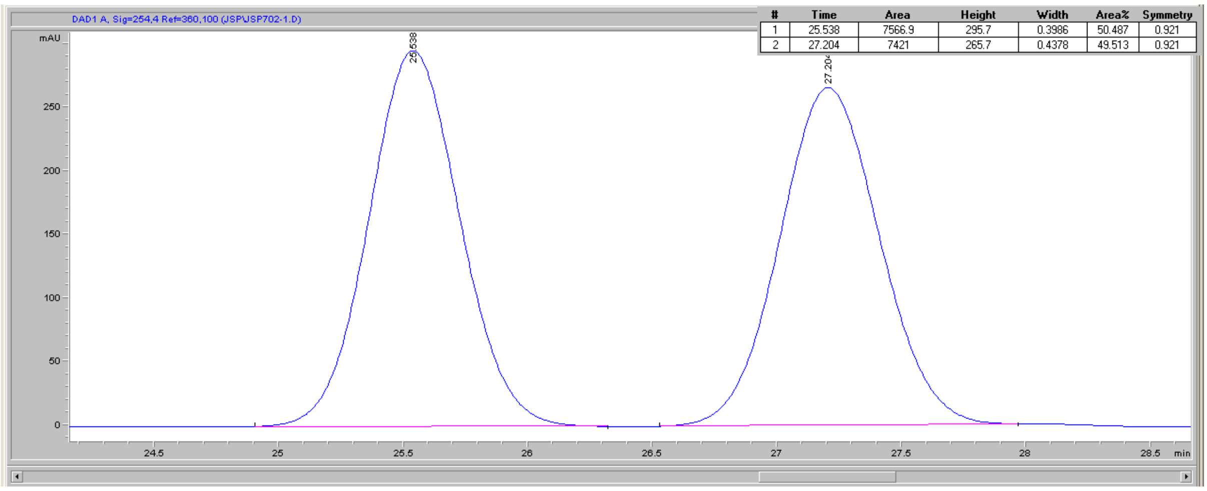

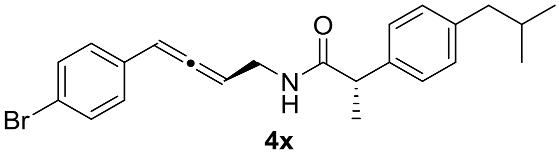

4x

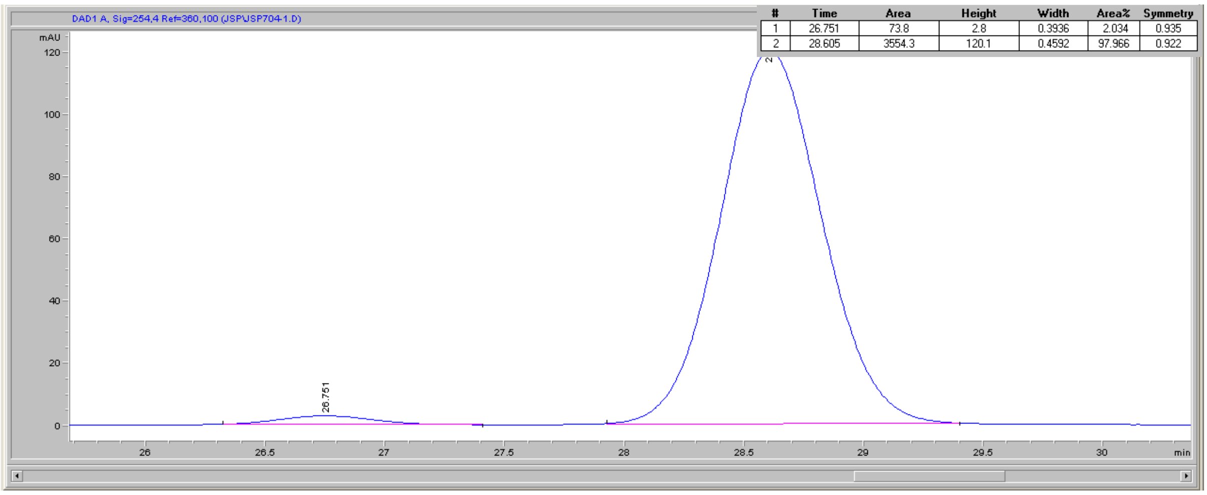

(*R*)-2-((4-(4-bromophenyl)buta-2,3-dien-1-yl)carbamoyl)phenyl acetate (4y):

HPLC conditions: ChiralART SA, 93:7 hexane/isopropanol, 1.0 mL/min flow rate, T = 25 °C, λ_{max} = 254 nm; t_R (min) = 26.2 (minor), 28.6 (major).

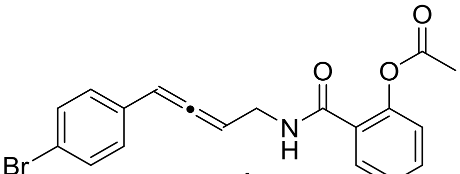

rac-**4y**

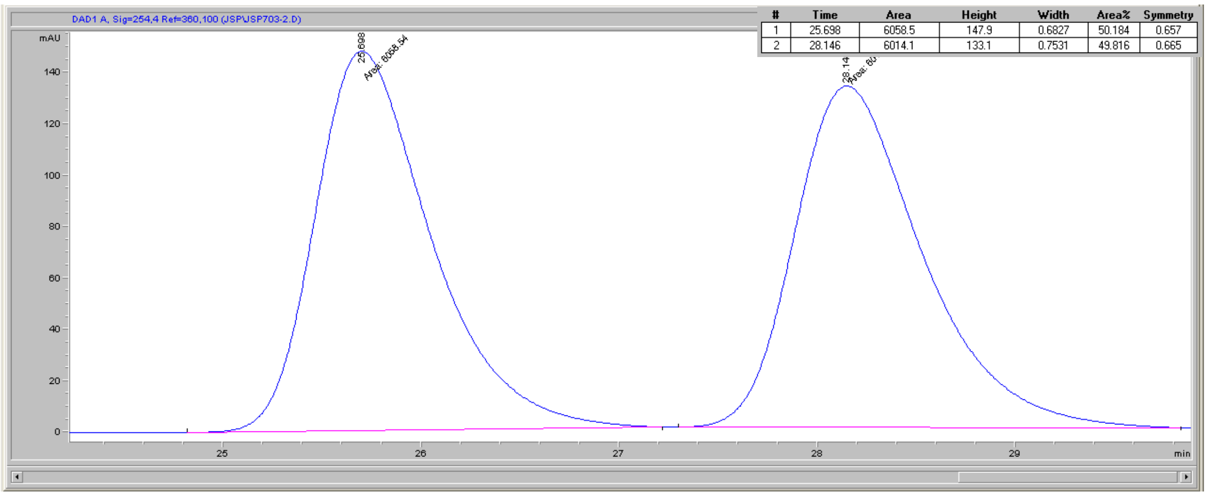

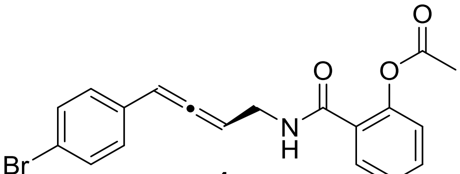

4y

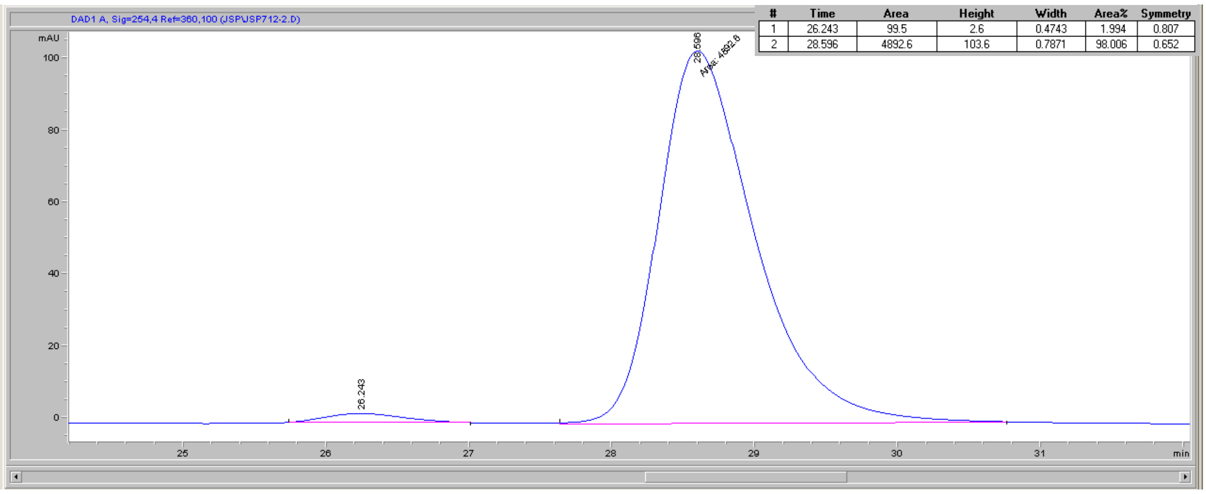

(2*S*,5*R*,6*R*)-*N*-((*R*)-4-(4-bromophenyl)buta-2,3-dien-1-yl)-3,3-dimethyl-7-oxo-6-(2-phenylacetamido)-4-thia-1-azabicyclo[3.2.0]heptane-2-carboxamide (4z):

HPLC conditions: ChiralART SC, 85:15 hexane/isopropanol, 1.0 mL/min flow rate, T = 25 °C, λ_{max} = 254 nm; t_R (min) = 30.3 (major), 33.3 (minor).

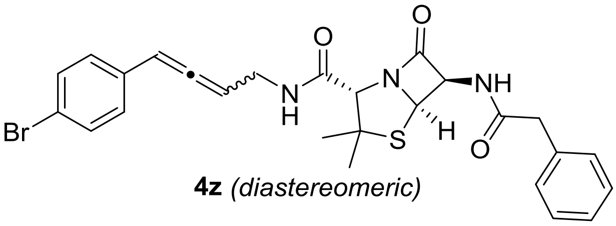

4z *(diastereomeric)*

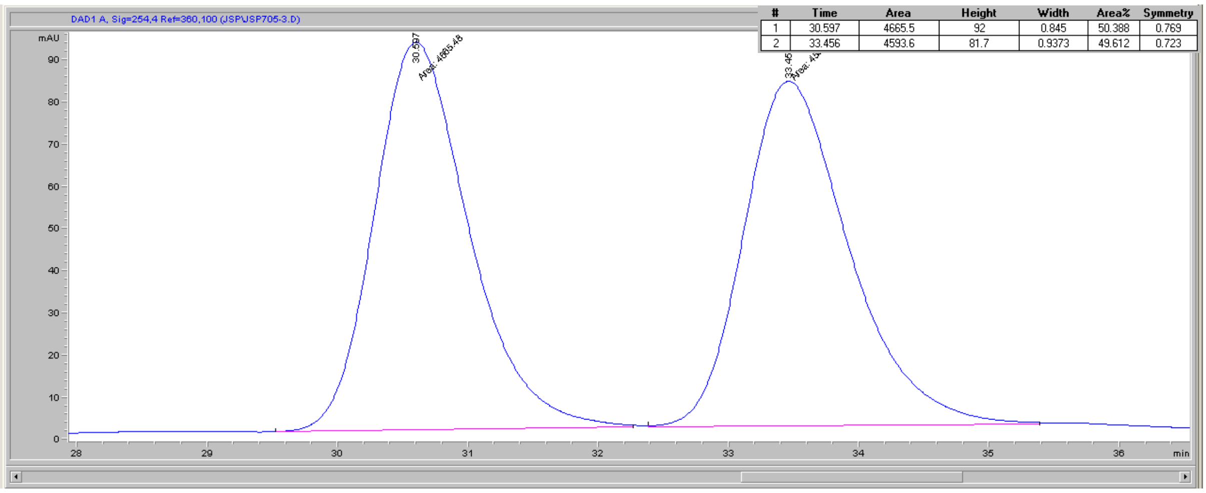

#	Time	Area	Height	Width	Area%	Symmetry
1	30.597	4665.5	92	0.845	50.388	0.769
2	33.456	4593.6	81.7	0.9373	49.612	0.723

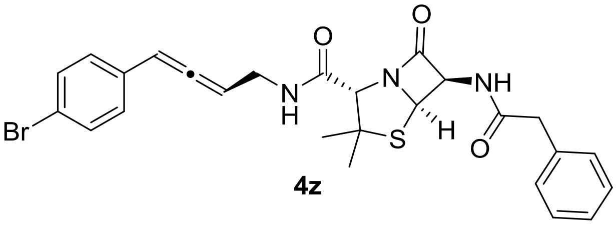

4z

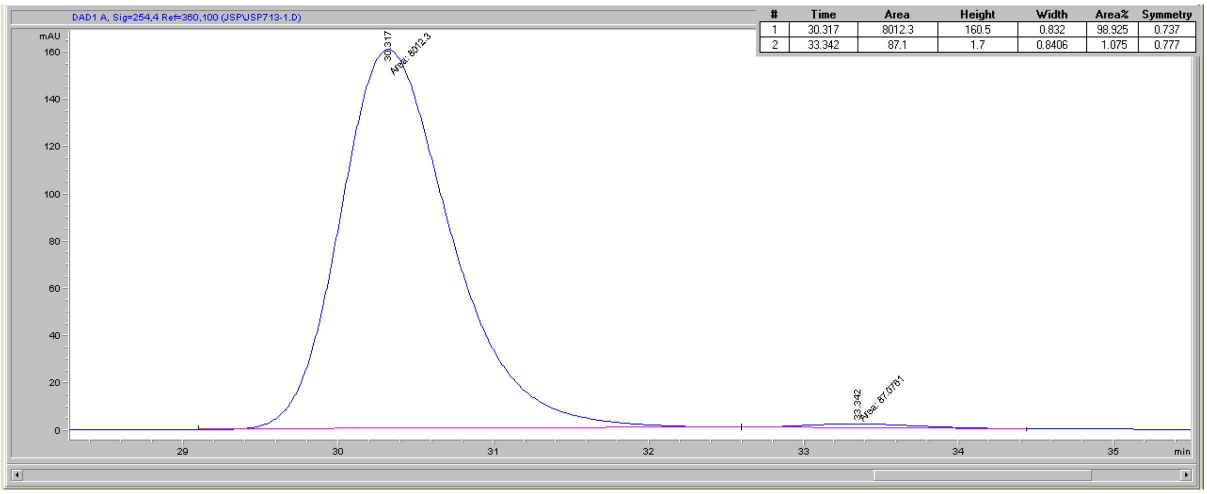

#	Time	Area	Height	Width	Area%	Symmetry
1	30.317	8012.3	160.5	0.832	98.925	0.737
2	33.342	87.1	1.7	0.8406	1.075	0.777

((3a*S*,5a*R*,8a*R*,8b*S*)-2,2,7,7-tetramethyltetrahydro-3a*H*-bis([1,3]dioxolo)[4,5-*b*:4',5'-*d*]pyran-3a-yl)methyl ((*R*)-4-(4-chlorophenyl)buta-2,3-dien-1-yl)sulfamate (4aa):

HPLC conditions: ChiralART SC, 95:5 hexane/isopropanol, 1.0 mL/min flow rate, T = 25 °C, λ_{max} = 254 nm; t_R (min) = 32.5 (minor), 34.4 (major).

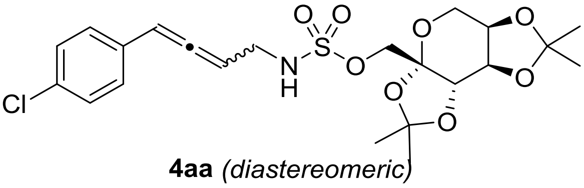

4aa *(diastereomeric)*

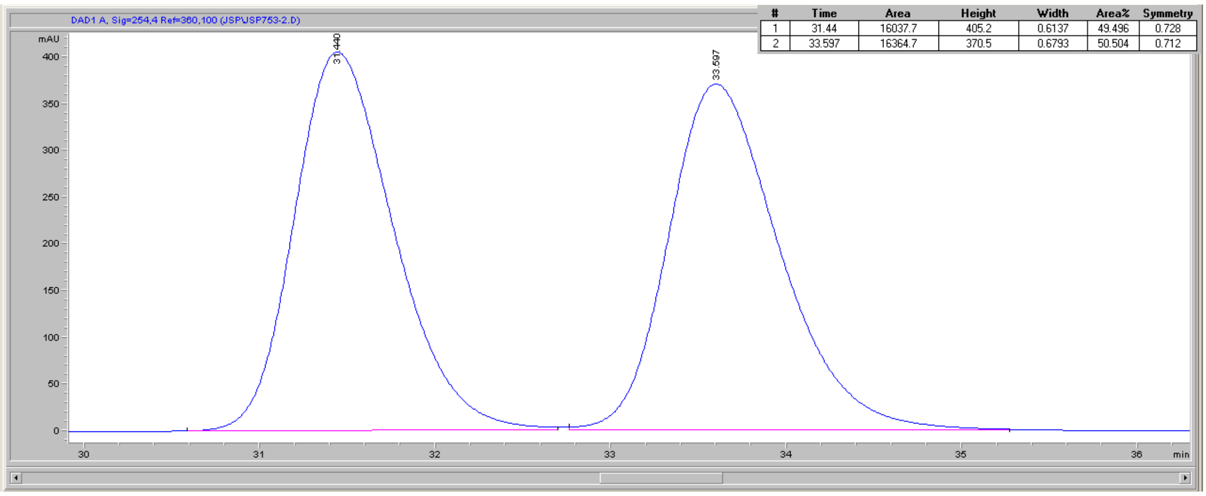

#	Time	Area	Height	Width	Area%	Symmetry
1	31.44	16037.7	405.2	0.6137	49.496	0.728
2	33.597	16364.7	370.5	0.6793	50.504	0.712

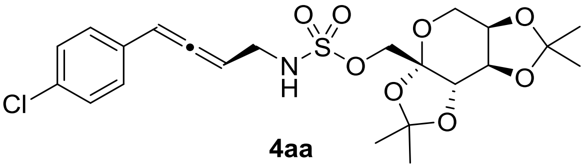

4aa

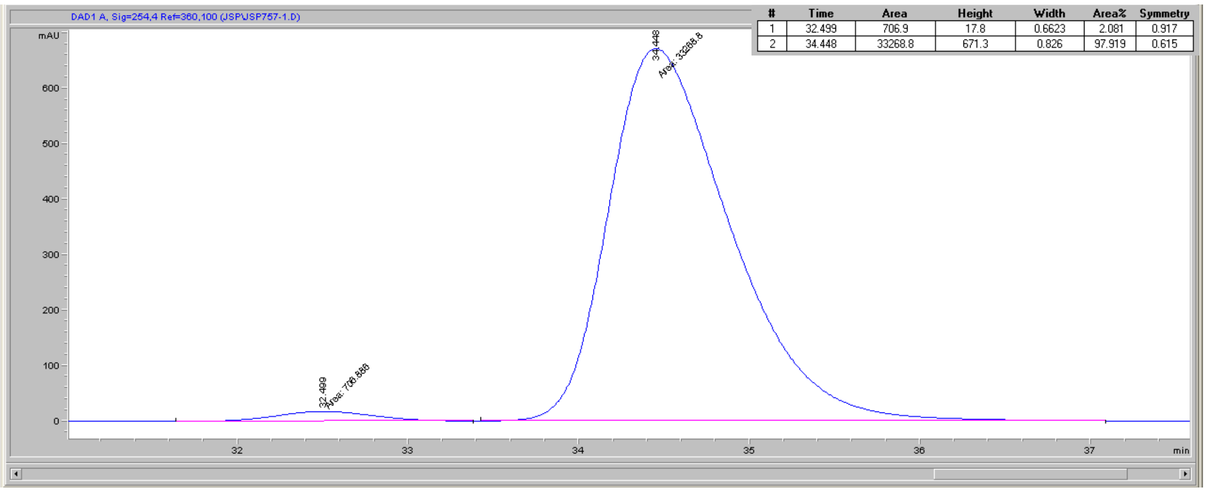

#	Time	Area	Height	Width	Area%	Symmetry
1	32.499	706.9	17.8	0.6623	2.081	0.917
2	34.448	33268.8	671.3	0.826	97.919	0.615

***tert*-butyl ((*S*)-2-(2-(((*R*)-4-(4-chlorophenyl)buta-2,3-dien-1-yl)amino)-2-oxoethyl)-4-methylpentyl)carbamate (4ab):**

HPLC conditions: ChiralART SA, 95:5 hexane/isopropanol, 1.0 mL/min flow rate, T = 25 °C, λ_{max} = 254 nm; t_R (min) = 15.9 (minor), 16.8 (major).

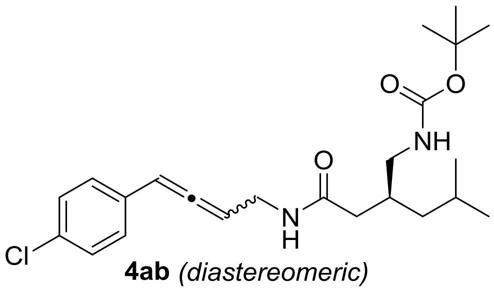

4ab *(diastereomeric)*

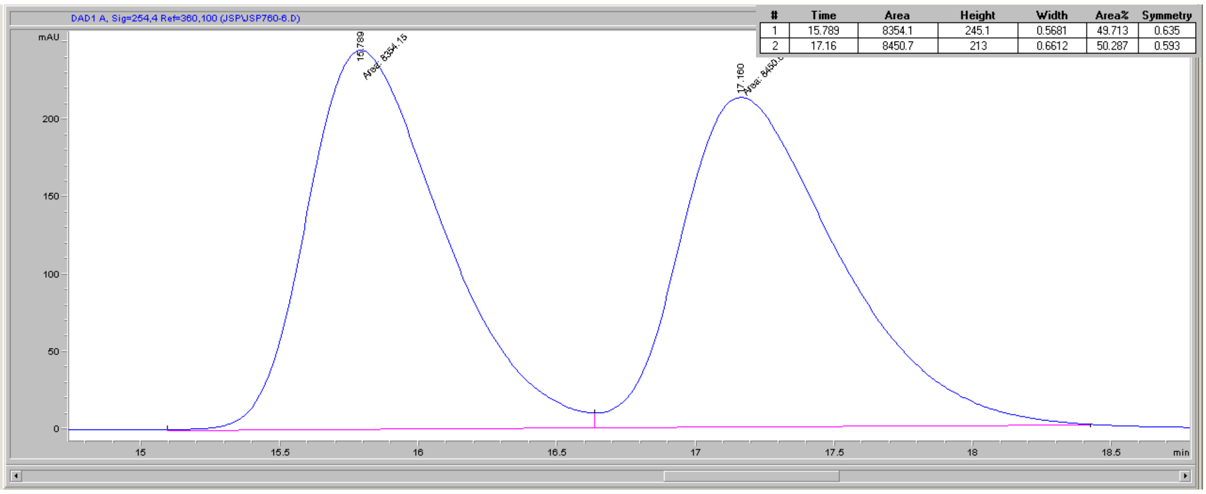

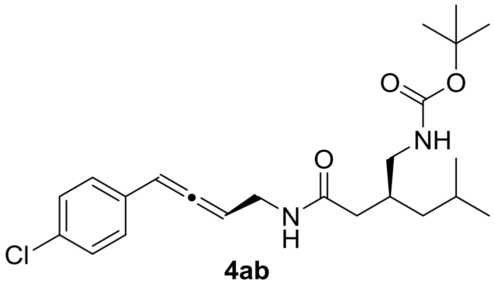

4ab

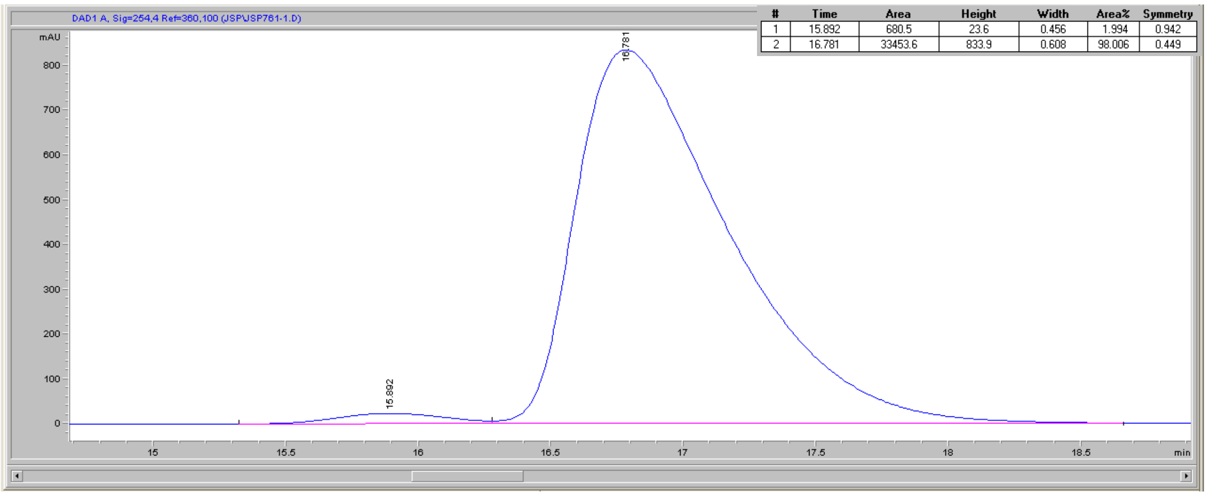

(*R*)-2-(1-(4-chlorobenzoyl)-5-methoxy-2-methyl-1*H*-indol-3-yl)-*N*-(4-(4-chlorophenyl)buta-2,3-dien-1-yl)acetamide (4ac):

HPLC conditions: ChiralART SA, 85:15 hexane/isopropanol, 1.0 mL/min flow rate, T = 25 °C, λ_{max} = 254 nm; t_R (min) = 21.2 (major), 30.1 (minor).

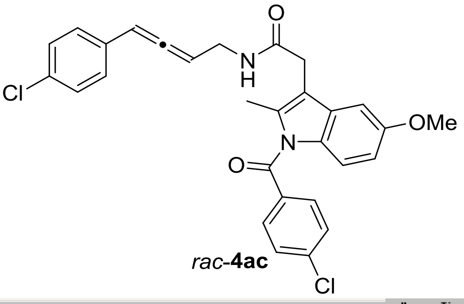

rac-**4ac**

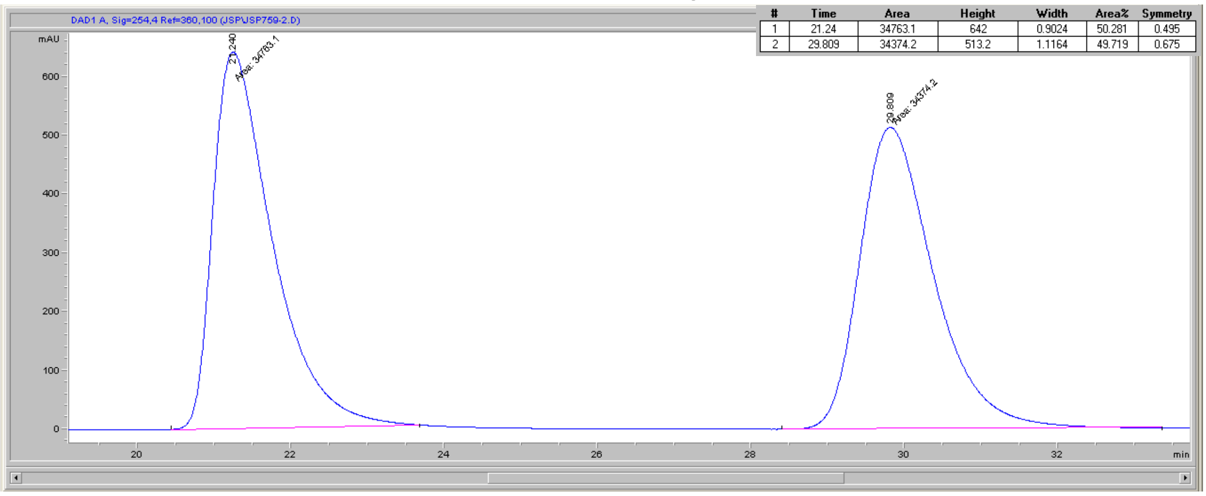

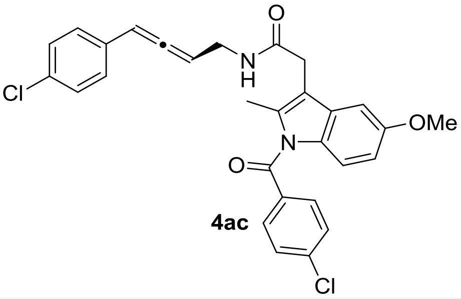

4ac

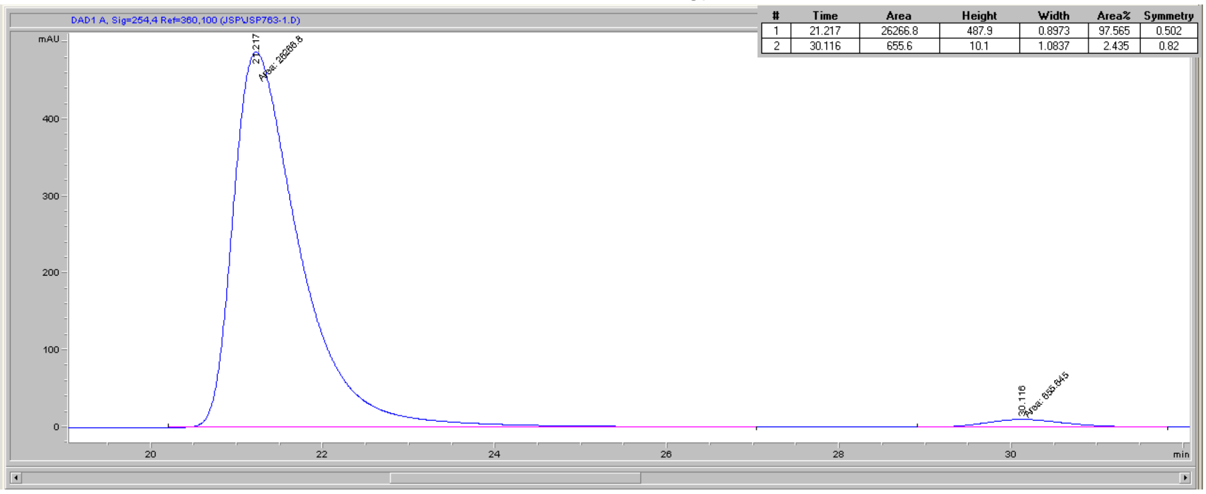

1-(2-((4*R*,6*R*)-6-(2-(((*R*)-4-(4-bromophenyl)buta-2,3-dien-1-yl)amino)-2-oxoethyl)-2,2-dimethyl-1,3-dioxan-4-yl)ethyl)-5-(4-fluorophenyl)-2-isopropyl-*N*,4-diphenyl-1*H*-pyrrole-3-carboxamide (4ad):

HPLC conditions: ChiralART SC, 85:15 hexane/isopropanol, 1.0 mL/min flow rate, T = 25 °C, λ_{max} = 254 nm; t_R (min) = 32.8 (minor), 38.0 (major).

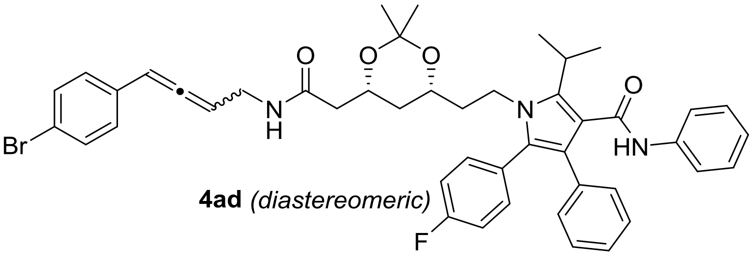

4ad *(diastereomeric)*

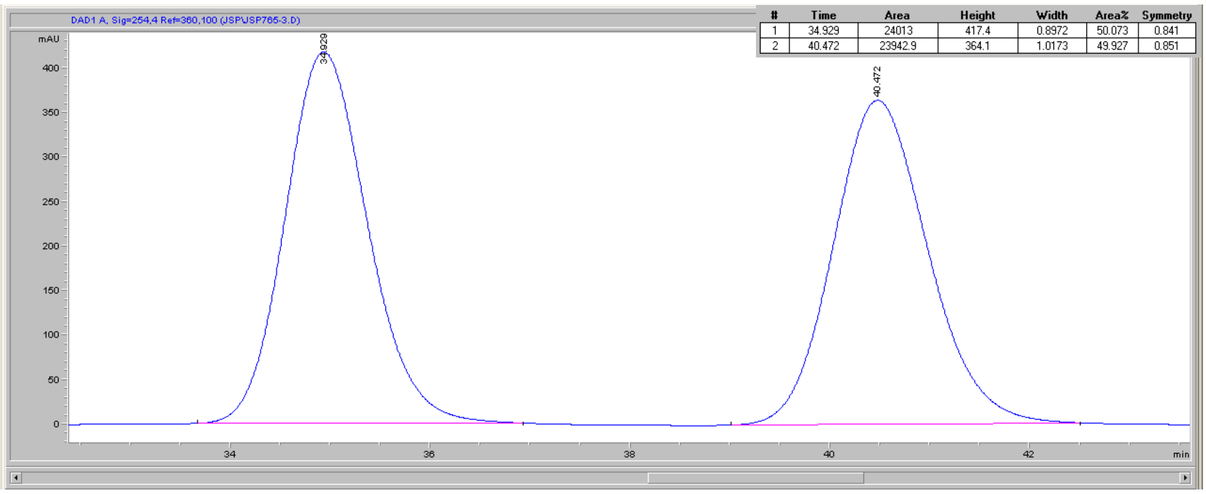

#	Time	Area	Height	Width	Area%	Symmetry
1	34.929	24013	417.4	0.8972	50.073	0.841
2	40.472	23942.9	364.1	1.0173	49.927	0.851

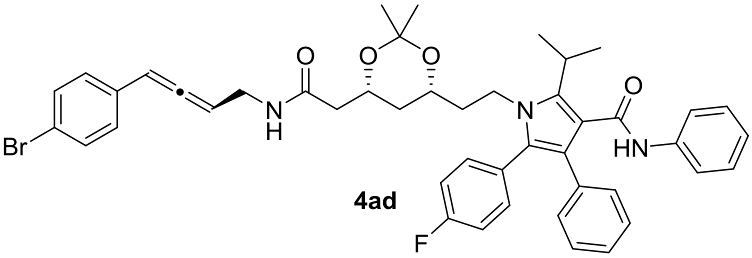

4ad

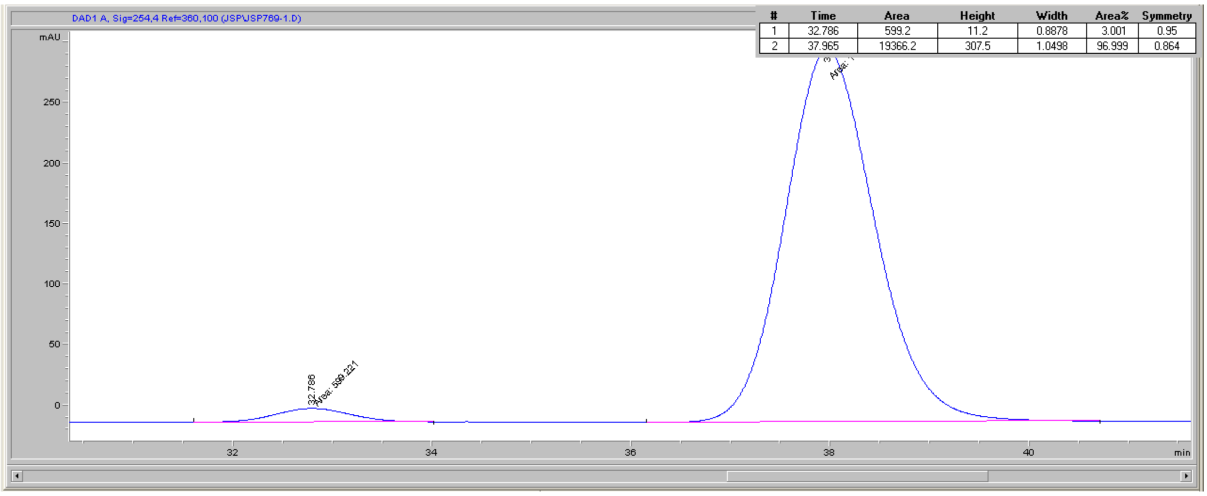

#	Time	Area	Height	Width	Area%	Symmetry
1	32.786	599.2	11.2	0.8878	3.001	0.95
2	37.965	19366.2	307.5	1.0498	96.999	0.864

(*R*)-1-chloro-4-(3-cyclopropylpropa-1,2-dien-1-yl)benzene:

HPLC conditions: Chiralpak OD-H, hexane, 1.0 mL/min flow rate, T = 25 °C, λ_{max} = 254 nm; t_R (min) = 6.7 (minor), 9.9 (major).

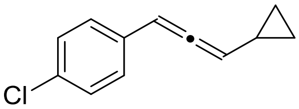

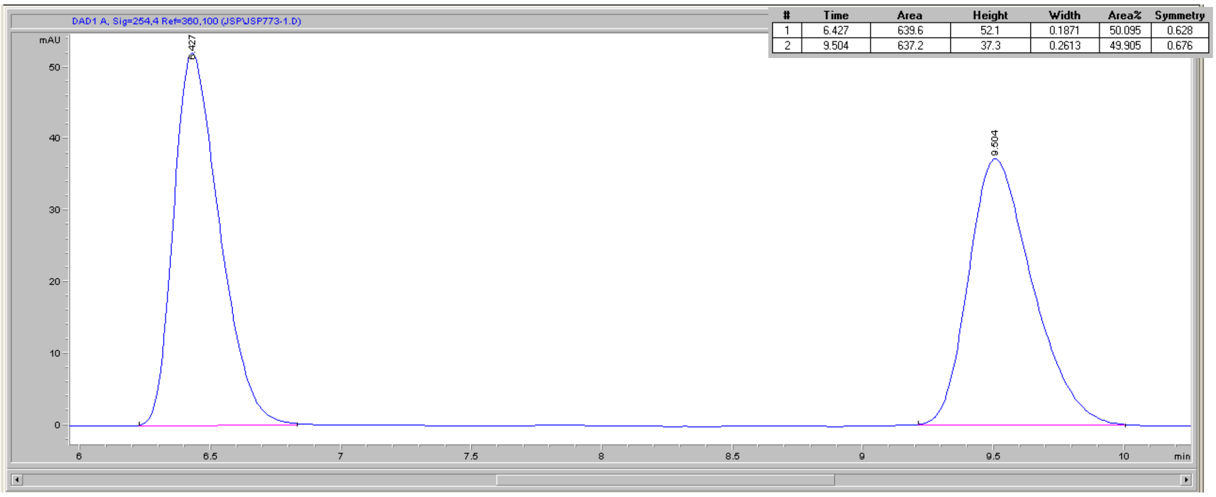

#	Time	Area	Height	Width	Area%	Symmetry
1	6.427	639.6	52.1	0.1871	50.095	0.628
2	9.504	637.2	37.3	0.2613	49.905	0.676

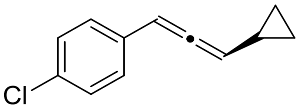

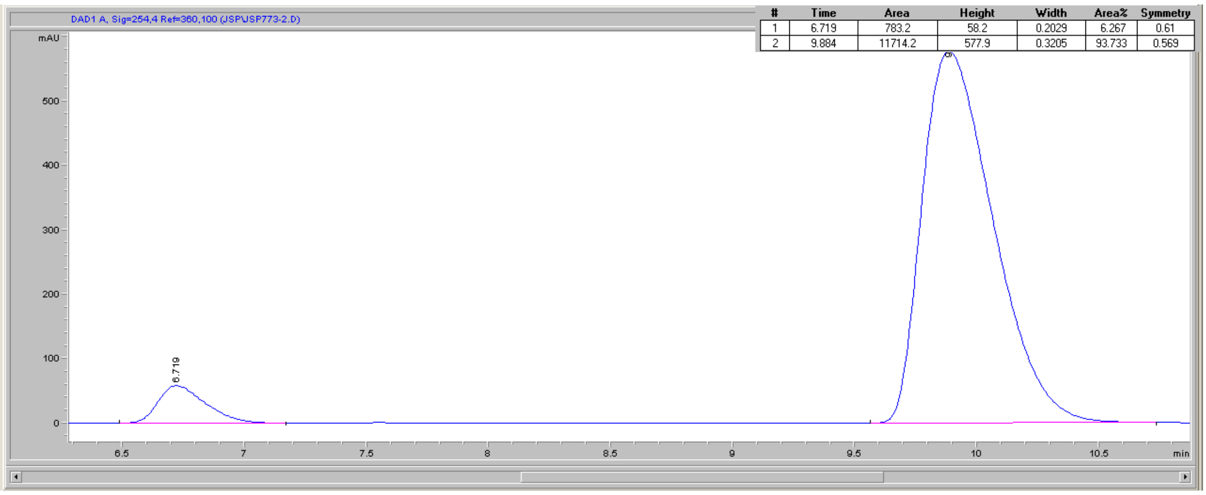

#	Time	Area	Height	Width	Area%	Symmetry
1	6.719	783.2	58.2	0.2029	6.267	0.61
2	9.884	11714.2	577.9	0.3205	93.733	0.569

(*S*)-2-(4-chlorophenyl)-1-tosyl-2,5-dihydro-1*H*-pyrrole (5):

HPLC conditions: ChiralART SC, 90:10 hexane/isopropanol, 1.0 mL/min flow rate, T = 25 °C, λ_{max} = 210 nm; t_R (min) = 33.7 (minor), 36.7 (major).

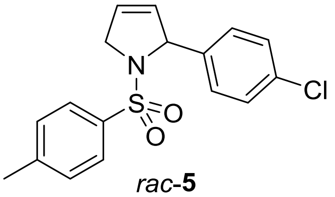

rac-**5**

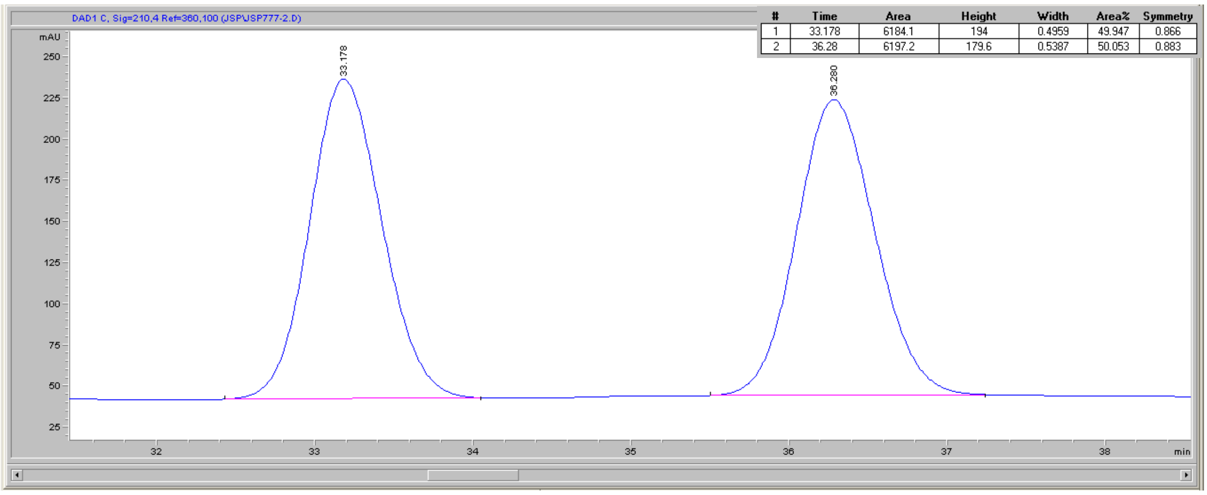

#	Time	Area	Height	Width	Area%	Symmetry
1	33.178	6184.1	194	0.4959	49.947	0.866
2	36.28	6197.2	179.6	0.5387	50.053	0.883

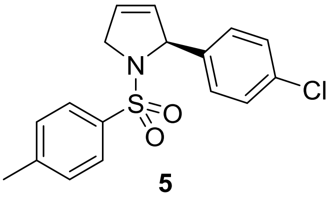

5

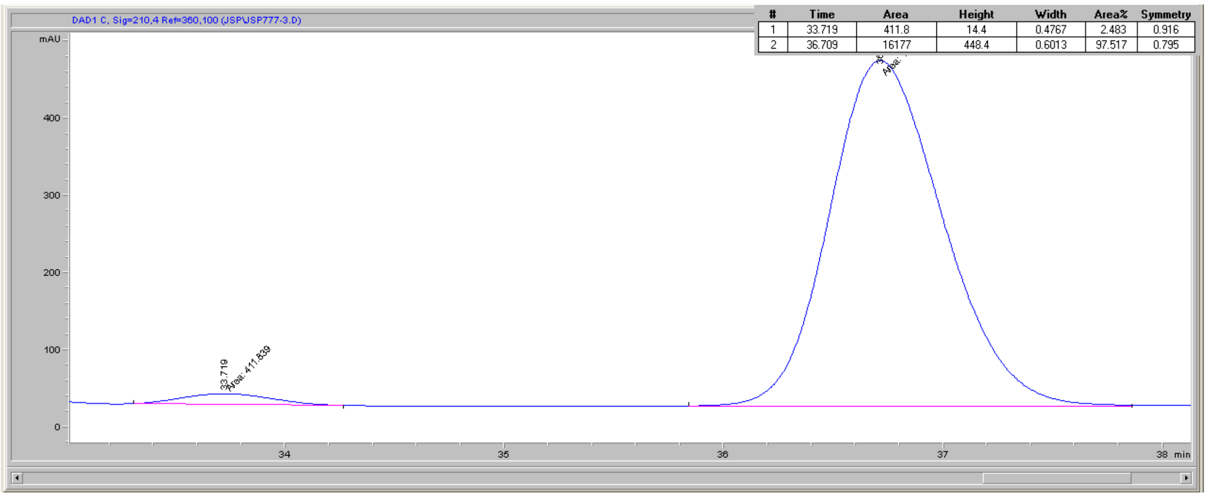

#	Time	Area	Height	Width	Area%	Symmetry
1	33.719	411.8	14.4	0.4767	2.483	0.916
2	36.709	16177	448.4	0.6013	97.517	0.795

References

[1] http://www.uniqsis.com/
[2] http://www.vapourtec.co.uk/home
[3] A. Cornejo, J. M. Fraile, J. I. García, M. J. Gil, V. Martínez-Merino, J. A. Mayoral, E. Pires, I. Villaba, *Synlett* **2015**, *15*, 2321-2324.
[4] M. K. Tse, S. Bhor, M. Klawonn, G. Anilkumar, H. Jiao, C. Döbler, A. Spannenberg, W. Mägerlein, H. Hugl, M. Beller, *Chem. Eur. J.* **2006**, *12*, 1855-1874.
[5] S. E. Schaus, E. N. Jacobsen, *Org. Lett.* **2000**, *2*, 1001-1004.
[6] J. Sanning, P. R. Ewen, L. Stegemann, J. Schmidt, C. G. Daniliuc, T. Koch, N. L. Doltsinis, D. Wenger, C. A. Strassert, *Angew. Chem. Int. Ed.* **2015**, *54*, 786-791; *Angew. Chem.* **2015**, *54*, 798-803.
[7] H. Nishiyama, S. Yamaguchi, M. Kondo, K. Itoh, *J. Org. Chem.* **1992**, *57*, 4306-4309.
[8] H. Nguyen, M. R. Gagné, *ACS Catal.* **2014**, *4*, 855-859.
[9] G. Desimoni, G. Faita, M. Guala, C. Pratelli, *Tetrahedron: Asymmetry* **2002**, *13*, 1651-1654.
[10] G. Desimoni, G. Faita, S. Filippone, M. Mella, M. G. Zampori, M. Zema, *Tetrahedron* **2001**, *57*, 10203-10212.
[11] T.-X. Métro, J. Appenzeller, D. G. Pardo, J. Cossy, *Org. Lett.* **2006**, *8*, 3509-3512.
[12] P. L. Beaulieu, D. Wernic, *J. Org. Chem.* **1996**, *61*, 3635-3645.
[13] J. You, K. Ma, *Chem. Eur. J.* **2007**, *13*, 1863-1871.
[14] M. J. Totleben, J. S. Prasad, J. H. Simpson, S. H. Chan, D. J. Vanyo, D. E. Kuehner, R. Deshpande, G. A. Kodersha, *J. Org. Chem.* **2001**, *66*, 1057-1060.
[15] J. D. Nobbs, A. K. Tomov, R. Cariou, V. C. Gibson, A. J. P. White, G. J. P. Britovesk, *Dalton Trans.* **2012**, *41*, 5949-5964.
[16] M. Z. Gao, J. H. Reibenspies, B. Wang, Z. L. Xu, R. A. Zingaro, *J. Heterocyclic Chem.* **2004**, *41*, 899-908.
[17] H. Kim, M. Stalkova, A. J. Lough, J. Chin, *Org. Lett.* **2009**, *11*, 157-160.
[18] S. Bhor, G. Anilkumar, M. K. Tse, M. Klawonn, C. Döbler, B. Bitterlich, A. Grotevendt, M. Beller, *Org. Lett.* **2005**, *7*, 3393-3396.
[19] M. S. Eno, A. Lu, J. P. Morken, *J. Am. Chem. Soc.* **2016**, *138*, 7824-7827.
[20] G. Chessa, L. Canovese, F. Visentin, C. Santo, R. Seraglia, *Tetrahedron* **2005**, *61*, 1755-1763.
[21] A. T. Parsons, J. S. Johnson, *J. Am. Chem. Soc.* **2009**, *131*, 3122-3123.
[22] D. J. Augeri, J. A. Robl, D. A. Betebenner, D. R. Magnin, A. Khanna, J. G. Robertson, A. Wang, L. M. Simpkins, P. Taunk, Q. Huang, S.-P. Han, B. Abboa-Offei, M. Cap, L. Xin, L. Tao, E. Tozzo, G. E. Welzel, D. M. Egan, J. Marcinkeviciene, S. Y. Chang, S. A. Biller, M. S. Kirby, R. A. Parker, L. G. Hamann, *J. Med. Chem.* **2005**, *48*, 5025-5037.
[23] G. Pelletier, W. S. Bechara, A. B. Charette, *J. Am. Chem. Soc.* **2010**, *132*, 12817-12819.
[24] J. Dong, Y. Gong, J. Liu, X. Chen, X. Wen, H. Sun, *Biorg. Med. Chem.* **2014**, *22*, 1383-1393.
[25] A. Baskakova, W. Frey, V. Jäger, *Synthesis* **2010**, *21*, 3693-3699.
[26] H. Song, Y. Liu, Q. Wang, *Org. Lett.* **2013**, *15*, 3274-3277.
[27] E. Trévisiol, E. Defrancq, J. Lhomme, A. Laayoun, P. Cros, *Tetrahedron* **2000**, *56*, 6501-6510.
[28] S. Bjelic, L. G. Nivón, N. Çelebi-Ölçüm, G. Kiss, C. F. Rosewall, H. M. Lovick, E. L. Ingalls, J. L. Gallaher, J. Seetharaman, S. Lew, G. T. Montelione, J. F. Hunt, F. E. Michael, K. N. Houk, D. Baker, *ACS Chem. Biol.* **2013**, *8*, 749-757.

[29] A. Lukin, T. Vedekhina, D. Tovpeko, N. Zhurilo, M. Krasavin, *RSC Adv.* **2016**, *6*, 57956-57959.
[30] E. M. Beccalli, E. Borsini, G. Broggini, G. Palmisano, S. Sottocornola, *J. Org. Chem.* **2008**, *73*, 4746-4749.
[31] A. S. K. Hashmi, J. P. Weyrauch, W. Frey, J. W. Bats, *Org. Lett.* **2004**, *6*, 4391-4394.
[32] W. R. Jackson, P. Perlmutter, A. J. Smallridge, *Aust. J. Chem.* **1988**, *41*, 1201-1208.
[33] B. Rajagopal, Y.-Y. Chen, C.-C. Chen, X.-Y. Liu, H.-R. Wang, P.-C. Lin, *J. Org. Chem.* **2014**, *79*, 1254-1264.
[34] J. Freedman, E. W. Huber, *J. Heterocyclic Chem.* **1990**, *27*, 343-346.
[35] J.-S. Poh, D. N. Tran, C. Battilocchio, J. M. Hawkins, S. V. Ley, *Angew. Chem. Int. Ed.* **2015**, *54*, 7920-7923; *Angew. Chem.* **2015**, *127*, 8031-8034.
[36] A. V. Shastin, V. N. Korotchenko, V. G. Nenajdenko, E. S. Balenkova, *Tetrahedron* **2000**, *56*, 6557-6563.
[37] V. Z. Shirinian, L. I. Belen'kii, M. M. Krayushkin, *Russ. Chem. Bull.* **1999**, *48*, 2171-2172.
[38] B. Bondzić, P. Eilbracht, *Org. Lett.* **2008**, *10*, 3433-3436.
